# Autoantibody Profiling of Glioma Serum Samples to Identify Biomarkers Using Human Proteome Arrays

**DOI:** 10.1038/srep13895

**Published:** 2015-09-15

**Authors:** Parvez Syed, Shabarni Gupta, Saket Choudhary, Narendra Goud Pandala, Apurva Atak, Annie Richharia, Manubhai KP, Heng Zhu, Sridhar Epari, Santosh B. Noronha, Aliasgar Moiyadi, Sanjeeva Srivastava

**Affiliations:** 1 Department of Biosciences and Bioengineering, Indian Institute of Technology Bombay, Powai, Mumbai 400076, India; 2 Department of Chemical Engineering, Indian Institute of Technology Bombay, Powai, Mumbai 400076, India; 3 Department of Pharmacology and Molecular Sciences/High-Throughput Biology Center, Johns Hopkins University School of Medicine, Baltimore, MD, USA; 4 Department of Pathology, Tata Memorial Centre, Mumbai 400 012, India; 5 Department of Neurosurgery, Tata Memorial Centre, Mumbai 400 012, India

## Abstract

The heterogeneity and poor prognosis associated with gliomas, makes biomarker identification imperative. Here, we report autoantibody signatures across various grades of glioma serum samples and sub-categories of glioblastoma multiforme using Human Proteome chips containing ~17000 full-length human proteins. The deduced sets of classifier proteins helped to distinguish Grade II, III and IV samples from the healthy subjects with 88, 89 and 94% sensitivity and 87, 100 and 73% specificity, respectively. Proteins namely, SNX1, EYA1, PQBP1 and IGHG1 showed dysregulation across various grades. Sub-classes of GBM, based on its proximity to the sub-ventricular zone, have been reported to have different prognostic outcomes. To this end, we identified dysregulation of NEDD9, a protein involved in cell migration, with probable prognostic potential. Another subcategory of patients where the IDH1 gene is mutated, are known to have better prognosis as compared to patients carrying the wild type gene. On a comparison of these two cohorts, we found STUB1 and YWHAH proteins dysregulated in Grade II glioma patients. In addition to common pathways associated with tumourigenesis, we found enrichment of immunoregulatory and cytoskeletal remodelling pathways, emphasizing the need to explore biochemical alterations arising due to autoimmune responses in glioma.

Gliomas are the most aggressive CNS tumours with poor prognosis^1^. World Health Organization (WHO) categorizes gliomas based on malignancy into 4 grades; where Grade I gliomas are localized and benign, whereas Grade II Gliomas are known to be diffused in nature. High Grade Gliomas include Grade III Gliomas, which are also called anaplastic gliomas while Grade IV gliomas, also termed as Glioblastoma multiforme (GBM), are the most malignant and aggressive form of glioma, known for its heterogeneous nature^2^,^3^.

Gliomas have been sub-typed based on various molecular markers like IDH1, 1p/19q co-deletion, amplification of EGFR amplification, loss of PTEN, MGMT etc. to predict the prognosis of the patients, with due consideration of parameters like patient’s age and complete histopathological profile^4^. One such sub-classification of GBMs is based on their position to the sub-ventricular zone (SVZ) in the brain^5^. The tumour positioned in proximity to the SVZ is called SVZ-positive (SVZp) while the tumour found in an area other than the SVZ, is termed SVZ-negative (SVZn). The prognosis of SVZn patients has been reported to be better than SVZp subjects, making the proximity of GBMs to the SVZ, a potential predictor of survival^6^. Similarly, IDH1 (isocitrate dehydrogenase 1) mutations have been a powerful molecular marker to predict the prognosis of glioma subjects, where subjects with IDH1 mutations referred to as positive for IDH1 mutations (IDH1p) are known to have better prognosis than those with the wild type copy of the IDH1 gene (WT)^7^. However, understanding the biological basis of this heterogeneity and its possible effect on autoantibody response, if any, is not clear.

Traditionally, gliomas have been diagnosed either by imaging techniques, histopathology or both^8^. Minimal-invasive and early diagnostic techniques can play an important role in improving the longevity and treatment of these patients^9^. The need for early diagnosis stems from the fact that, the two-year survival of the GBM patients is less than 30%^10^. The extent of invasiveness and risks involved in brain biopsies required to establish disease condition necessitates the need for novel serum based biomarkers to incorporate minimal invasive diagnosis^9^. This can be achieved with the help of autoantibody response towards certain aberrant self-proteins termed as tumour associated autoantigens (TAAs) using protein microarray based platforms. Neoplasms evoke an immune response against these TAAs, and this is often accompanied by the production of autoantibodies^11^. There are various reasons for the immunogenicity of the TAAs, such as expression of embryonic proteins in adults, expression of mutated oncogenic proteins and overexpression of proteins^12^. Such autoantibodies can be used for early diagnosis of cancers. However, for achieving higher sensitivity and specificity, a panel of autoantibodies should be targeted, instead of a single autoantibody^11^.

In this study, we performed screening of sera from healthy controls and various grades of glioma patients using human proteome arrays containing more than 17000 proteins (Fig. 1a,b). To the best of our knowledge, this is the first study performing autoantibody profiling of such a huge collection of recombinant proteins using glioma sera across various grades of glioma. The enrichment analysis of such differentially expressed proteins highlighted the underlying perturbed pathways, which may play key roles in the tumourigenesis and progression of the disease. The enriched pathways include the pathways leading to the invasiveness of the disease. We have also identified potential candidate proteins, which are not only able to distinguish the healthy controls from various grades of glioma, but also the sub-types observed in case of the aggressive GBM, which provides the necessary groundwork for minimal invasive diagnostics of this disease.

## Results

### Quality control and protocol optimization

**Figure 1:**
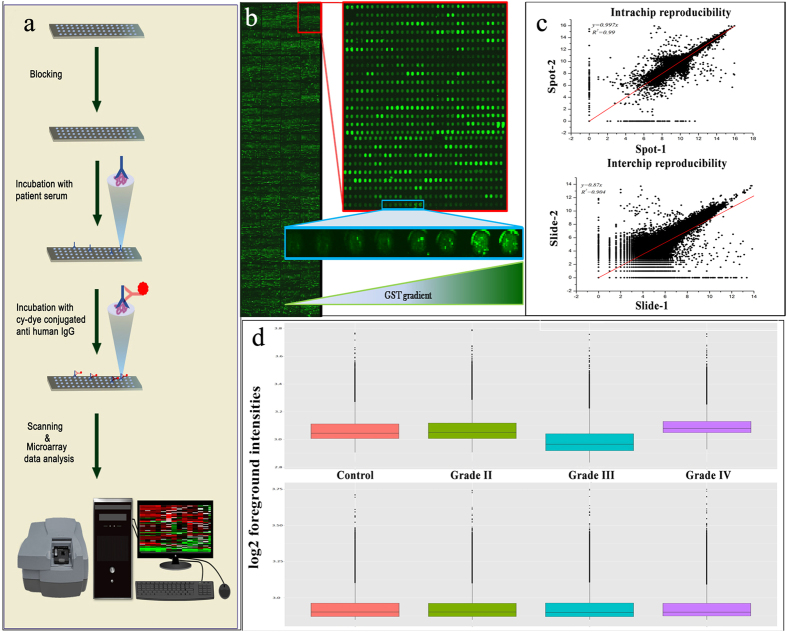
Experimental workflow and data preprocessing. (**a**) illustrates the experimental procedure involved in the microarray experiments in this study. (**b**) represents the quality of the proteins spotted on the microarray. The zoomed-in panel shows the increase in signal intensity with increase in concentration of purified GST protein spotted. Scatter plots in the top panel of (**c**) shows intrachip reproducibility, while the bottom panel represents interchip reproducibility. (**d**) represents the distribution of the data across all slides pertaining to each group of the samples. The top figure shows unnormalized data while the bottom panel shows normalized data (drawn by the authors PS and SG).

As a part of quality control experiment, we analysed the signal intensities obtained from varying concentrations of GST purified proteins spotted, which were found to be directly proportional. The reproducibility of the duplicate spots of the same proteins showed good correlation (R^2^ = 0.99) (Fig. 1c). Similarly, fairly good correlation (R^2^ = 0.904) was observed between the signal-intensities from different microarrays processed on different days using the same serum sample (Fig. 1c).

### Protein microarray data normalization

After the background correction and normalization, the data seemed to be median centric (Fig. 1d), thereby eliminating any possible technical variances. This pre-processed data was used for further analysis.

### Differential expression analysis

**Figure 2:**
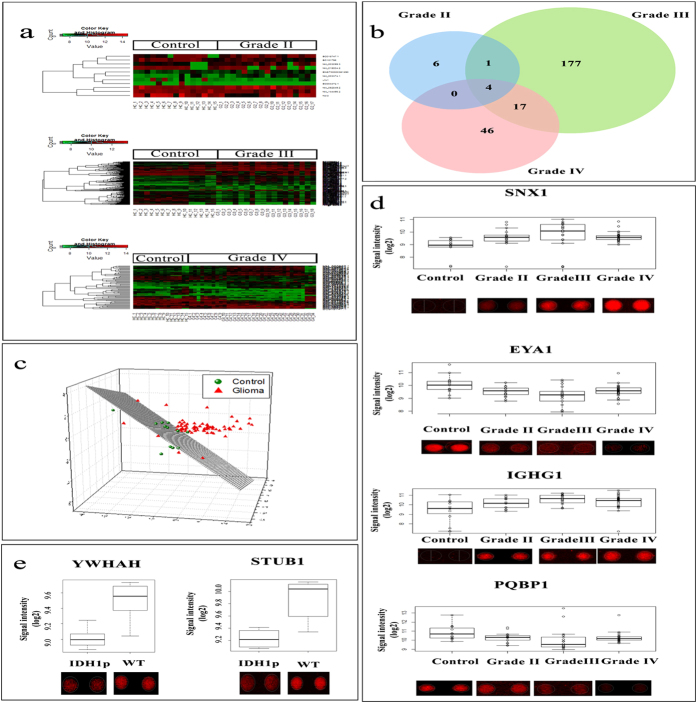
Differentially expressed proteins. (**a**) represents heat-maps of comparisons of healthy controls with various grades of glioma using their respective differentially expressed proteins. (**b**) shows the number of unique and overlapping proteins dysregulated in various grades of glioma compared to the healthy controls. (**c**) represents the MDS plot highlighting poor discrimination of healthy controls from glioma samples using 4 commonly dysregulated proteins, SNX1, EYA1, PQBP1 and IGHG1. Their expression patterns across different sample types have been shown in panel (**d**) along with their spot intensity patterns on the microarray slides. (**e**) represents the expression patterns and spot intensities of few significantly dysregulated proteins in Grade II IDH1p cohort against Grade II glioma patients with WT IDH1.

The expression values of all the proteins from the healthy controls were compared to that of different grades of gliomas (Supplementary Tables 1, 2 and 3). In this process, we identified varied numbers of proteins which showed significant dysregulation. Although, each of these proteins showed differential expression, when combined, these proteins did not show any discriminative power (Fig. 2a). Among the 11 differentially expressed proteins in Grade II samples, we found 5 up-regulated proteins and 6 down-regulated proteins. Similarly, in Grade III samples we identified a list of the 200 significant proteins, which includes 97 up-regulated proteins and 103 down-regulated proteins. When healthy controls were compared with Grade IV samples, we identified 67 significant differentially expressed proteins. Among these 67 proteins, 34 were up-regulated, while the remaining 33 proteins were down-regulated.

When the lists of differentially expressed proteins were compared with each other, we identified sets of proteins which were differentially expressed in more than one grade (Fig. 2b). For example, EXOSC7 was found to be differentially expressed in Grade II and III. Similarly there were 17 proteins, namely, RNF25, C11orf74, CALCOCO2, C14orf119, IMPDH2, TIPIN, CAMK2N1, LOC285382, MAPK3, HBG2, JUB, ZMYM3, PRPSAP2, LRFN1, DUPD1, TIRAP and ZNF397, which were dysregulated both in Grade III and IV (Fig. 2b). However, there were no common proteins which were differentially expressed in Grade II and IV with the exception of 4 proteins, SNX1, EYA1, PQBP1, and IGHG1, which were dysregulated across all the grades of gliomas (Fig. 2b). The multidimensional scaling plot of the healthy controls and gliomas using these 4 proteins (Grades II, III and IV together) showed some partial discrimination (Fig. 2c). Among these 4 proteins, SNX1 and IGHG1 were up-regulated in gliomas, while, PQBP1 and EYA1 were down-regulated in gliomas (Fig. 2d).

The comparison of SVZp versus SVZn (Supplementary Table 4) revealed dysregulation of 18 proteins. Of these, the expression of 6 proteins was found to be higher in the SVZp samples compared to that of SVZn samples. On the other hand, lower expression of 12 proteins in SVZp samples was observed in comparison with SVZn.

Another analysis with IDH1 mutation as focus, revealed 22 proteins significantly dysregulated in a comparison of Grade II WT versus IDH1p (Supplementary Table 5). Among these proteins YWHAH and STUB1 were found to be down-regulated in IDH1p (Fig. 2e). No significantly dysregulated protein was found in Grade III WT versus IDH1p and only 1 significantly dysregulated protein in case of Grade IV WT versus IDH1p was found. Considering all the WT IDH1 across all grades as a cohort against all IDH1p subjects as another cohort, we did not find any significantly dysregulated proteins.

### Enrichment Analysis

**Figure 3:**
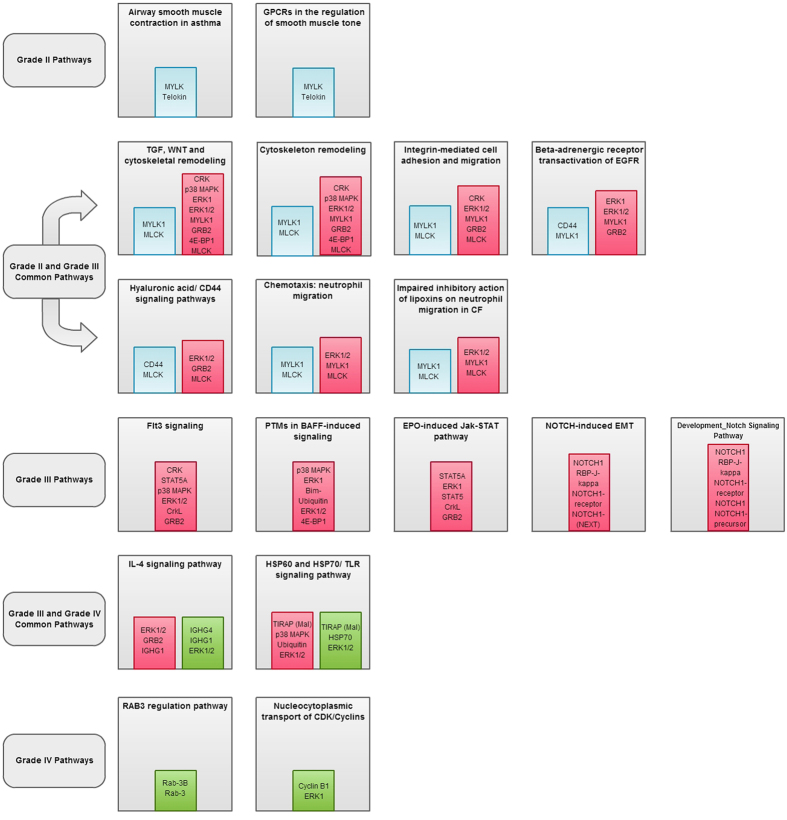
Enriched Pathways emerging from TAAs in each grade. Figure 3 schematically represents the enriched pathways emerging from deregulated TAAs in each grade. The proteins attributed to these pathways are highlighted in colour-coded panels. The pathways common between grades have also been represented in the above panels. Blue coloured panels denote proteins from Grade II, red coloured panels represent proteins from Grade III and green coloured panels signify proteins from Grade IV in any given pathway. These images were created from the data generated by GeneGoMetacore.

Grade II Gliomas were found to be enriched in invasive pathways like cytoskeleton based cell adhesion and migration, cytoskeleton remodelling, chemotaxis, etc. (Fig. 3 and Supplementary Table 6). Metabolic networks like phosphatidic acid pathway, phosphatidylinositol-4,5-diphosphate pathway (Supplementary Table 7) process networks like cell adhesion & cell matrix attractions and cytoskeleton remodelling involving actin filaments were enriched in this grade (Supplementary Table 8). Grade III Gliomas had pathways like cytoskeleton based cell adhesion and migration, cytoskeleton remodelling common with the low-grade gliomas (Fig. 3). In addition to this, differentially expressed proteins in Grade III were found to be enriched in certain cell signalling pathways like Flt3, JAK-STAT, NOTCH, interleukin signalling pathways etc. (Fig. 3 and Supplementary Table 6). Process networks were found to be enriched in NOTCH signalling pathways and haematopoiesis development and blood vessel development etc. (Supplementary Table 8). Grade IV gliomas were found to be enriched in immune regulatory pathways, cell cycle signalling pathway and RAB3 signalling pathways (Fig. 3 and Supplementary Table 6). Process networks that were found to be enriched were inflammation signalling pathways, cell cycle transition from G2 to M phase and protein folding and unfolding (Supplementary Table 8). No significant metabolic networks could emerge in case of Grade III and GBM when subjected to enrichment analysis.

TLR signalling and histone phosphorylation were among the top enriched Gene Ontology (GO) processes in case of comparison of high grade gliomas with healthy subjects while aorta morphogenesis was one of the top hits in this category, in case of low grade gliomas (Supplementary Table 9). On enriching these differentially expressed proteins for diseases by known biomarkers, intraductal papilloma was the top hit while large granular lymphocytic leukaemia emerged among the top hits in high-grade gliomas (Supplementary Table 10). The results from GeneGo Metacore (Thomson Reuters) with a complete list of FDR corrected enriched pathways, metabolic networks, process networks, GO terms, enriched diseases by known biomarkers and their implicated TAAs can be found in the supplementary Tables 5–9.

QIAGEN’s Ingenuity^®^Pathway Analysis (IPA) was used to analyse the most significant interactions of differentially expressed proteins with special focus to the involvement of classifier proteins. Interaction networks like “cell death and survival, inflammatory response, cell to cell signalling and interaction” and “developmental disorder, hereditary disorder, neurological disease” network were implicated in Grade II (Supplementary Fig. 1a,b); “cell death and survival, cancer, cellular assembly” and “organization and cancer, gastrointestinal diseases, cellular growth and proliferation” were among the significant interactions in Grade III (Supplementary Fig. 1c,d); and “cellular movement, inflammatory response” and “cell morphology, cellular movement, cell to cell signalling and interactions”, were amongst the most important networks in Grade IV when differentially expressed proteins were studied with respect to healthy controls in the above cases (Supplementary Fig. 1e,f).

### Classifiers

As the lists of all significant differentially expressed proteins together could discriminate the diseased samples from healthy ones with good efficiency and efficacy; we elucidated panels of 10 proteins for this purpose using Support Vector Machine (SVM) (Supplementary Fig. 2). SVM is known to be an extremely useful tool for deducing smaller sets of proteins that can distinguish any two desired cohorts from a larger set of significant differentially expressed proteins^13^. Such a panel of 10 proteins could differentiate the healthy controls from Grade II samples with 88% sensitivity and 87% specificity with Area Under Curve (AUC) value of 0.9 and classification accuracy of 87%. This panel included 5 up-regulated proteins which were SNX1, MYLK, VDAC1, IGHG1 and CCDC32, and 5 down-regulated proteins like EYA1, CD44, NOL3, PQBP1 and EXOSC7 (Table 1).

Another panel of 10 proteins was elucidated which differentiated healthy samples from Grade III samples with 89% sensitivity and 100% specificity at an AUC value of 1 and classification accuracy of 93%. This panel included 9 up-regulated proteins such as C14orf80, GCK, HSD17B14, LYPLAL1, MAGEA4, MLX, RTN4, SNX1 and TEX264 and 1 down-regulated protein, ARHGAP17 (Table 1).

Similarly, another set of 10 proteins, was found to differentiate the healthy samples from Grade IV samples with 94% sensitivity, 73% specificity, AUC value of 0.975 and classification accuracy of 88%. The panel included proteins like SNX1, IGHG1 and C11orf74, which were up-regulated and down-regulated proteins like C17orf57, CIB1, RCSD1, CDH26, PQBP1, EYA1 and ZHX3 (Table 1).

Through the intra-grade analysis of Grade IV i.e. SVZp versus SVZn, we identified a set of 10 classifier proteins which helped distinguish SVZp samples from SVZn with 77% sensitivity and 95% specificity and with an AUC value of 0.975. These classifier proteins included 6 proteins, PGM2, DR1, HIBADH, GPBP1, EEF1A1 and LOC339685, which were overexpressed in SVZp and 4 proteins, TMOD4, FAM120B, NEDD9 and GMEB1, which were overexpressed in SVZn (Table 1).

**Figure 4:**
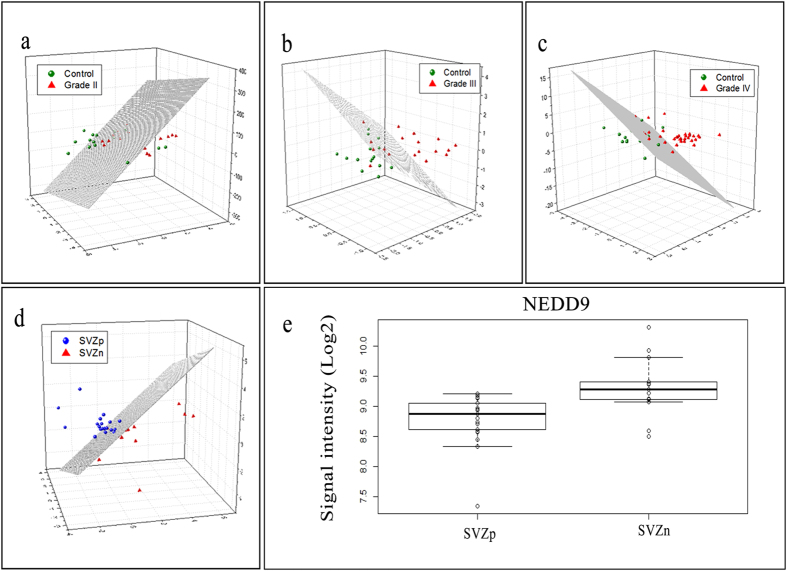
Multidimensional scaling (MDS) using classifiers. The top panel in (**a**) denotes the MDS plot of healthy controls versus Grade II samples. Similarly, panels (**b**,**c**) represent the separation of the healthy controls versus Grade III and Grade IV using MDS, respectively. (**d**) represents the MDS plot corresponding to SVZp versus SVZn. The boxplot showing the expression pattern of NEDD9 across SVZp and SVZn samples is represented in panel (e).

The multidimensional scaling (MDS) plots of healthy samples versus different grades of gliomas revealed a separation of samples albeit with some mis-classification error (Fig. 4a,b&c). Similarly, separation of SVZp samples from SVZn samples could be observed with few samples being mis-classified (Fig. 4d).

A robust classifier panel could not be deduced to distinguish IDH1p and WT subjects in any grade while maintaining stringent statistical thresholds; however few of the significant proteins exhibited interesting trends in the IDH1p and WT cohorts (Supplementary Fig. 2).

## Discussion

Glioma is one of the most common and aggressive types of primary brain tumours^1^ in which early confirmatory diagnostic examinations of subjects involve highly invasive techniques like biopsies^14^. Although surgery is the first line of therapy in most cases, sometimes, when surgery is not possible or when only a biopsy is required^14^, alternative diagnostic confirmation may be preferable. In clinical trials, especially those where the intervention being assessed is surgery (for example comparison of two different adjuncts), it is also desirable to know a priori the possible histological type in order to ensure homogeneity of the sample^15^. Another possible application is to ascertain tumour transformation during imaging surveillance of untreated low-grade gliomas. Further, in the post-treatment setting, there is often a lot of diagnostic dilemma between true progression or recurrence and treatment related changes. Presently MRI is the only clinically available diagnostic modality. Though it has tremendously improved the diagnostic capabilities, it has its limitations. In such scenarios, serum markers which reflect disease biology may be a very useful option. In this context, production of autoantibodies is one of the first reactions that the immune system elicits in response to an establishing tumour^16^. Being produced in abundance not only makes autoantibody detection an ideal aid for early diagnosis of the disease, but it could also help in confirming the histotype of the tumour depending on the nature of proteins that present aberration in that histotype. This may be because, the number of glial antigens exposed to the immune system increases with the extent of breach of the blood-brain barrier as the disease progresses, which should ideally unveil some unique signatures. A panel of autoantibody markers thus, has the potential of complementing the existing conventional diagnostic techniques for gliomas. It could also possibly address the problem of heterogeneity within grades and sub-types, allowing for individualized therapeutic decisions rather than blanket protocols in clinics. Our study fits in as a foundation to realize these goals of early diagnosis, minimal invasive diagnosis and identification of autoantibody signatures within each grade. Low-grade gliomas could potentially progress into the more aggressive grades and it is, therefore, of prime importance to find a set of early diagnostic markers. Although our panel of early diagnostic markers separates Grade II subjects and healthy controls, there is a lack of clear distinction between the two cohorts which can be attributed to the fact that the blood brain barrier is not breached at an early stage of the disease, due to which we can expect relatively few glial self-antigens to be exposed to the humoral system thereby eliciting a poor response. As the severity of the disease increases, we find that the number of TAAs detected is substantially higher than that of the low-grade gliomas which also reflects on this breach of the blood brain barrier and therefore, the autoantibody response. This can be seen from the extent of differentiation between high-grade gliomas (Grade III and Grade IV) from healthy individuals.

A holistic observation of this analysis revealed four proteins, IGHG1, EYA1, SNX1 and PQBP1, which were found to be significantly dysregulated in all the grades compared to the healthy individuals. Of these four proteins, SNX1 contributed to the classification of healthy controls and all the three grades of gliomas. On the other hand IGHG1, EYA1 and PQBP1 were found to help in the classification of Grade II and Grade IV from healthy individuals. The expression of IgG heavy region has been reported in various cancerous cell lines. However, the reasons for its expression are still not very clear^17^. Contrary to the popular belief that the immunoglobulins are produced only in mature B lymphocytes, some studies have reported carcinoma cells and nervous cells to produce immunoglobulins^18^,^19^,^20^. In a study conducted by Li *et al.*^21^, IGHG1 was found to be up-regulated in human pancreatic carcinomas and the blockage of IGHG1 was correlated with retarded tumour development and better survival. Through this study the authors have, also, hypothesized that IGHG1 might play a role in immune evasion mechanisms, thus making IGHG1 an interesting classifier candidate. EYA1 which was a classifier protein for separating healthy controls with subjects in Grade II and Grade IV belongs to eyes absent (EYA) family of evolutionarily conserved proteins and is known to have putative role in innate immunity, DNA damage repair, angiogenesis and cancer metastasis, etc.^22^. EYA1 has shown ambiguous expression profiles across a number of known cancers. Over-expression of EYAs has been observed in ovarian and breast cancers^23^ while on the other hand, Nikpour *et al.*^24^ have reported down-regulation of EYA1 in gastric cancer. SNX1 is a member of sorting nexin proteins subfamily and the members of this subfamily and is known to interact with epidermal growth factor receptor (EGFR). Earlier studies have shown that the over-expression of SNX1 leads to the degradation of EGFR^25^. Our studies have shown that the SNX1 protein is up-regulated in gliomas and we could not find differential expression of EGFR in gliomas. PQBP1 binds to BRN2, a POU-III class of neural transcription factors. PQBP1 inhibits the transcription activation of BRN2^26^. BRN2 is known to be expressed in glioblastoma and is associated with the development of neural and glial cells^27^. Although these 4 proteins collectively failed to differentiate the healthy samples from various grades of glioma, these 4 proteins in combination with other grade specific differentially expressed proteins did separate healthy samples from gliomas with reasonably good classification accuracy.

Differentiating sub-categories of a given grade of cancer is extremely important in order to understand underlying mechanisms in the manifestation of the disease. In this study, we tried to identify differentially expressed proteins between the sub-categories of Grade IV i.e. SVZp and SVZn. NEDD9 has been known to cause invasiveness in lung cancer and melanoma^28^,^29^. Such invasiveness can be attributed to its role in the regulation and activation of transcriptional pathways that are associated with cancer progression and metastasis. In the context of GBM, NEDD9 increases the migration capacity by acting as a downstream effector of FAK^30^. The interactions of NEDD9 with Src, FAK and Crk results in tyrosine phosphorylation which help in the formation of binding sites for effector proteins containing SH2 domains and such complexes triggers cell migration^31^. In this study, we found NEDD9 to be up-regulated in the SVZn samples (Fig. 4e). Studies have shown that SVZ involvement is an adverse prognostic factor in GBMs^32^. There is a lot of speculation that the SVZ region is the site of origin of most GBMs. Thus, a subset of GBMs that express NEDD9 could be the ones which migrate away from the SVZ. This migration may in some way alter the biology and make SVZn tumours more favourable in terms of prognosis. Thus, NEDD9 expression could serve as a useful marker for a favourable subgroup and may be further validated for targeted therapeutic potential. In addition to the SVZ subtyping, we tried to distinguish the WT cohorts against the IDH1p cohorts to understand any significant patterns in the autoantibody signature (Fig. 2). The mutation in *IDH1* gene is widely associated with good prognosis of glioma patients. One of the primary reasons for a prolonged survival is that the lower levels of NADPH found in the IDH1p patients. The lower levels of NADPH make the tumour more responsive to irradiation and chemotherapy^7^. Also, it is widely accepted that, *IDH1* mutation is more frequent in younger patients, compared to that of older^33^. The median age of IDH1p patients, in our study, was 29 years (standard deviation ±9 years), while the median age of WT patients was found to be 46 years (standard deviation ±16 years) (Supplementary Table 5). In this study, we could identify set of 22 proteins, which were dysregulated in the Grade II gliomas when the IDH1p samples were compared to that of WT. Among these 22 proteins, YWHAH^34^ and STUB1^35^ (also known as CHIP) are known to be involved in proliferation of glioma cells and were found to be significantly upregulated in the WT cohort when compared with the IDH1p cohort. This dysregulation may attribute to the poor prognosis of WT patients. Although, few differentially expressed proteins for IDH1p and WT among Grade II and Grade IV samples were identified but due to small sample size a set of stringent classifier protein from the WT versus IDH1p analysis could not be deduced.

Biochemical pathways form the framework, which leads the molecular and biological processes in a system to function optimally^36^. Tracing potential TAAs to biochemical pathways may give us an understanding of pathways which are more aberrant and could be the likely targets of the immune system to generate an autoimmune response which in turn reflects on the disease pathobiology^37^. If studied in greater depths, pathway analysis can also help in developing generalized therapies if core pathways are targeted. Many of the pathways traced from our list of potential TAAs have highlighted pathways which are known to have a pronounced role in tumourigenesis^38^. Pathways regulating nucleocytoplasmic transport of CDK/Cyclins in cell cycle signalling, NOTCH signalling, EGFR signalling, ERK1/2 signalling, JAK/STAT signalling, cytoskeletal remodelling pathways involving TGF and WNT etc. are few amongst them^38^. While the escalation from low grade to high grade glioma were pronounced in pathways involving cell migration and cytoskeletal remodelling, the high grade gliomas were found to be more enriched in immunoregulatory pathways. IL-4 signalling pathway is one such pathway which was found to be enriched and has been implicated to show aberrations in glioma cell lines^39^. In glioma cells, IL-4 signalling involves aberrant activation of STAT3 instead of STAT6 which are known to lead to enhanced cell proliferation, cell survival and angiogenesis in a number of human cancers^40^. Genetic alterations in PI3K pathway has been found to be one of the critical signalling pathways resulting in the manifestation of gliomas and is also one of the pathways stringently regulated by the IL-4 signalling pathway^36^,^39^,^41^ which leads us to consider the upstream effectors of such critical pathways which may be deregulated due to autoimmune responses in addition to known genetic mutations. HSP60 and HSP70/TLR signalling pathway was another immune-regulatory pathway, which was enriched in high grade glioma patients. TIRAP (MAL) is one of the adaptor proteins which transmits signals from TLRs^42^. TLR stimulation results in activation of NF-κB, MAPKs, JUN N-terminal kinases (JNKs), p38, ERKs which normally regulate the host immune responses^43^. TLR signalling pathways have also been associated with enhanced cell survival by over-expression of anti-apoptotic proteins like Bcl-2 related protein A1 (BCL2A1), inhibitors of apoptosis 1 (cIAP1), cIAP2, XIAP and Bcl-2 family members through PI3K-Akt signaling^43^,^44^. Certain proteins in the TLR family have been implicated in the pathogenesis of infectious, chronic inflammatory and autoimmune disorders^45^,^46^. Heat shock proteins (Hsp) have been reported to bring about TLR mediated pro-inflammatory cytokine production in macrophages and maturation of dendritic cells^47^. It is interesting to note that Hsps like Hsp70 are known to help in the TLR mediated accumulation of Aβ peptides in microglia resulting in downstream activation of p38 MAPKase and NF-κB activation in case of neurodegenerative disorders like Alzheimer’s disease^47^. Proteins like Hsp70, MAPKase/ERK1/2 and TIRAP were found to be amongst the TAAs in our study. Apart from immunoregulatory pathways, integrin mediated cell adhesion and migration was one of the significant pathways, which emerged in the high-grade gliomas. Integrins are also involved in the regulation of intracellular signalling pathways, which in turn control the cytoskeletal organization and survival, thus playing a major role in tumourprogression^48^. A cytoplasmic protein kinase known as focal adhesion kinase (FAK) co-localizes with the integrins and form focal adhesions. Higher levels of expression of FAK are known to be observed in aggressive forms of tumours compared to the benign tumours^49^ and FAK is linked to promote cell survival^50^ and integrin mediated cell migration^51^. Further, FAK is also linked to the activation of RAS–extracellular-signal-regulated kinase (ERK). FAK activates ERK through the recruitment of adaptor proteins like GRB2 and CAS^52^. GRB2, ERK, CRK etc. were TAAs from these pathways were detected in our study. About 15–20% of the CNS space is taken up by the extra cellular space which is made up of the extra cellular matrix (ECM)^53^. ECM has high levels of glycosaminoglycan (GAG). Hyaluronan (HA) which is one of the principal components of the GAG, binds to CD44, enhancing cell motility which correlates with the invasive nature of the disease^54^. In a histopathological study, it was also established that CD44 expression was found to be correlating with the invasiveness and aggressiveness of glioma which is attributed due to its enhanced ability of migrating through the ECM^55^. Although many of the proteins implicated in the above pathways have been individually reported in glioma, it is indeed interesting to investigate if these pathways have any crucial role in the pathobiology of glioma on the whole. In this light, it is also interesting to find that the most significant pathways, especially from the high-grade glioma data, were those which regulate essential activities of the immune system itself. This therefore, necessitates further study to understand the underlying reasons for immune system to target its own key components to elicit an autoimmune response under diseased conditions.

The findings from our data point towards the potential lists of classifiers, which are differentially expressed in various grades of gliomas compared to that of healthy control samples. To the best of our knowledge, this is a first study of its kind where more than 17,000 proteins were screened for their autoantigenicity using glioma serum samples on a protein microarray platform. The pathway analysis from potential TAAs opens a new avenue, which needs to be pursued in depth as to how and why these proteins are targeted by the immune system. This also gives us an understanding of the likely functionalities to be compromised depending on the targeted biochemical pathways. The findings from our study, thus, lay the foundation for early diagnosis of gliomas and prognostics.

## Materials and Methods

### Ethics Statement

The tumour serum samples collected and experiments conducted in this study were approved as part of an institutional review board approved study (ACTREC-TMC IEC No. 15, Advanced Centre for Treatment, Research and Education in Cancer and Tata Memorial Centre) in accordance with approved guidelines. Patients with radiologically suspected gliomas were enrolled after giving written informed consent.

### Serum sample collection

Blood was collected prior to the first surgery and before patients receiving any kind of treatment. The collected serum samples were aliquoted into smaller volumes and stored at −80 °C until further use. Prior to the assay, the serum samples were thawed on ice. For the evaluation of serum samples from Glioma grades II (*n* = *17*), III (*n* = *18*) and IV (*n* = *34*) patients and healthy individuals (*n* = *15*) we used Human Proteome arrays (HuProt™ arrays) (Johns Hopkins University). The median age of the patients with Grade II, III and IV were 32, 32.5 and 57 years with a standard deviation of 15, 12 and 11 years, respectively. Further patient details are enlisted in the Supplementary Tables 1, 2, 3 and 4. The grade IV samples comprised of 21 SVZp and 13 SVZn samples.

For the comparison of WT versus IDH1p, in Grade II, the IDH1 status for 8 out of 17 patients were known, where 4 patients were profiled IDH1p while 4 were WT. In grade III, off 18 patients, the IDH1 status of 10 patients was known where, 7 patients were IDH1p while 3 were WT. In case of Grade IV patients, off 34 patients, 3 patients were IDH1p mutated while 4 were WT. The IDH1 statuses for these patients were determined by sequencing the IDH1 gene (Supplementary Table 5).

### Microarray fabrication

The HuProt™ arrays comprised of ~17000 unique full-length proteins printed in duplicates. The expression of these 17000 GST-tagged proteins was done in *Saccharomyces cerevisiae* expression system. In addition to these full-length proteins, positive controls (H2A, H2B, H3, H4 and GST in various concentrations) and negative controls (BSA, HeLa cell lysates, p300-BHC) were spotted in duplicates on the microarrays^56^.

### Microarray assay

Prior to the screening of the serum samples for the detection of autoantibody signatures (Fig. 1a), the quality of the Glutathione S-transferase (GST) tagged recombinant proteins immobilized on the microarray slide surface was checking using anti-GST antibody. The GST purified proteins spotted on the microarray slide were of varying concentrations (Fig. 1b). In this experiment, the microarray slides were incubated with anti-GST antibody. Later, secondary antibody conjugated with Cy3 was used to check the quality of the printing on the slide.

In order to obtain a high signal-to-noise ratio one needs to optimize the assay. We used 3% non-fat milk powder in TBST (tris-buffered saline with 0.1% tween20), 3% BSA in TBST, SuperBlock (Pierce) and 3% BSA in SuperBlock. Similarly, various dilutions of serum and secondary antibody were tried.

Finally, the protocol which gave us the best intrachip and interchip reproducibility and low background and high signal-to-noise ratio was used to further evaluate the serum samples. For deducing the intrachip reproducibility, we used the signal intensities from the duplicate spots of the same protein, while the interchip reproducibility was calculated using the signal intensities from the identical spots on two different microarray slides processed using same serum sample on different days (Fig. 1c). The protocol is as follows: The microarray was blocked using 3% BSA in SuperBlock for 2 hours on a gentle shaker. Then the serum sample was diluted 1:500 in 2% BSA in TBST and incubated for 2 hours on a shaker. To this diluted serum, 1:5000 rabbit anti-GST antibody was also added. Later, a cocktail of secondary antibody, containing 1:1000 dilution of anti-human IgG conjugated with Cy5 and 1:5000 dilution anti-rabbit antibody conjugated with Cy3, was applied onto the microarray slide. All these incubation steps were followed by rinsing thrice with TBST, washing the slides for 4 × 5 min with TBST, rinsing with distilled water and drying at 900 rpm for 2 min. All the steps were performed at room temperature. After the final wash and drying of the slides, scanning was performed using GenePix 4000B Microarray Scanner (Molecular Devices).

### Statistical analysis

The processed microarrays were scanned with GenePix 4000B Microarray Scanner (Molecular Devices). The image processing and the data acquisition of microarrays were done using GenePix Pro 7 (Molecular Devices). In order to identify differentially expressed proteins, the raw data needs to be pre-processed so as to adjust variation between arrays, thus adjusting for any effects not arising due to biological differences^57^. We make use of “limma^58^” package made available as a Bioconductor^59^ package for the R programming language (https://www.r-project.org/).

Pre-processing the data involves two steps: background correction and normalization. Background correction was performed using the ‘nec’ method present in ‘limma’. ‘nec’ performs ‘normexp-by-control’ background correction utilizing the negative controls only. An offset of 100 is added to the final adjusted values in order to reduce the variability of the log ratios. This data is then normalized across arrays using ‘normalizebetweenarrays’ method with the ‘quantile normalization’ function in limma. Quantile normalization normalizes the value in two or more data sets by making the distribution of the probe intensities identical, statistically by extending the concept of Q-Q plots to n-dimensions, where n is the number of proteins, in our case n = 17000.

The data having been pre-processed is then analysed for selecting differentially expressed genes. ‘limma’ makes use of moderated t-statistic to test the null hypothesis that the genes are not differentially expressed. These tests are adjusted for multiple hypothesis testing using the ‘Benjamin-Hochberg’(BH) correction method. Though limma was primarily developed for DNA microarrays, it has been used for analysing protein microarrays^60^.

The proteins with a Log-fold change more than 0.5 or less than −0.5 and with an adjusted p value less than 0.05 were selected to be potentially differentially expressed. The chosen cut off values essentially focuses on genes with higher fold changes which are also significant statistically. Thus, in grade II versus control comparison the threshold was maintained at p value less than 0.05. For grade III versus healthy and Grade IV versus healthy comparisons the threshold was maintained at adjusted p value less than 0.05. The shortlisted proteins were then put to pathway analysis to determine if they were a part of a pathway that might have strong implications for tumourigenesis. By applying recursive feature elimination using SVMs with k-fold cross-validation we elucidated a panel of 10 classifier proteins, which enable differentiation of healthy samples from glioma samples. For visualization of the samples being discriminated using these panels, we used MDS. The 10 dimensional classifier space was mapped to a 3 dimensional space using a non-isometric MDS algorithm.

### Pathway analysis

After identifying the significant proteins from various comparisons, we proceeded further for the identification of enriched pathways and gene ontology terms using GeneGoMetacore™ software (Thomson Reuters). The significance level of 0.05 was used for this analysis and FDR corrected values at p < 0.05 were used as a threshold for these analysis. We, further, wanted to investigate the protein-protein interactions among the significant proteins identified from various grades of gliomas for which we used QIAGEN’s Ingenuity^®^Pathway Analysis (IPA^®^, QIAGEN Redwood City, www.qiagen.com/ingenuity).

## Additional Information

**How to cite this article**: Syed, P. *et al.* Autoantibody Profiling of Glioma Serum Samples to Identify Biomarkers Using Human Proteome Arrays. *Sci. Rep.* **5**, 13895; doi: 10.1038/srep13895 (2015).

## Supplementary Material

Supplementary Information

## Figures and Tables

**Table 1:** Panels of classifiers.

**Symbol**	**Full name**	**AUC**	Sensitivity(%)	Specificity(%)	**PPV**	**NPV**
HC vs Grade II						
SNX1	sorting nexin 1	0.9	88	87	0.88	0.87
MYLK	myosin light chain kinase					
VDAC1	voltage-dependent anion channel 1					
IGHG1	immunoglobulin heavy constant gamma 1 (G1m marker)					
CCDC32	coiled-coil domain containing 32					
EYA1	EYA transcriptional coactivator and phosphatase 1					
CD44	CD44 molecule (Indian blood group)					
NOL3	nucleolar protein 3 (apoptosis repressor with CARD domain)					
PQBP1	polyglutamine binding protein 1					
EXOSC7	exosome component 7					
HC vs Grade III						
C14orf80	chromosome 14 open reading frame 80	1	89	100	1	0.88
GCK	glucokinase (hexokinase 4)					
HSD17B14	hydroxysteroid (17-beta) dehydrogenase 14					
LYPLAL1	lysophospholipase-like 1					
MAGEA4	melanoma antigen family A, 4,					
MLX	MLX, MAX dimerization protein					
RTN4	reticulon 4					
SNX1	sorting nexin 1					
TEX264	testis expressed 264					
ARHGAP17	Rho GTPase activating protein 17					
HC vs Grade IV						
SNX1	sorting nexin 1	0.975	94	73	0.89	0.85
IGHG1	immunoglobulin heavy constant gamma 1 (G1m marker)					
C11orf74	chromosome 11 open reading frame 74					
C17orf57	EF-hand calcium binding domain 13					
CIB1	calcium and integrin binding 1 (calmyrin)					
RCSD1	RCSD domain containing 1					
CDH26	cadherin 26					
PQBP1	polyglutamine binding protein 1					
EYA1	EYA transcriptional coactivator and phosphatase 1					
ZHX3	zinc fingers and homeoboxes 3					
SVZp vs SVZn						
NEDD9	neural precursor cell expressed	0.975	77	95	0.91	0.87
PGM2	phosphoglucomutase 2					
DR1	down-regulator of transcription 1					
FAM120B	family with sequence similarity 120B					
TMOD4	tropomodulin 4 (muscle)					
HIBADH	3-hydroxyisobutyrate dehydrogenase					
GPBP1	GC-rich promoter binding protein 1					
GMEB1	glucocorticoid modulatory element binding protein 1 eukaryotic translation elongation factor 1 alpha 1					
EEF1A1	Eukaryotic Translation Elongation Factor 1 Alpha					
LOC339685	LOC339685					

This table shows the sensitivities, specificities and the corresponding AUC values for various comparisons (AUC: Area under curve; PPV: positive predictive value; NPV: negative predictive value).
