# Supplementary material for: Autoantibody Profiling of Glioma Serum Samples to Identify Biomarkers Using Human Proteome Arrays
Source: Sci Rep. 2015 Sep 15;5:13895. doi: 10.1038/srep13895 (PMC4570193; doi:10.1038/srep13895)
Supplement: Supplementary Information [file srep13895-s1.pdf]

Autoantibody Profiling of Glioma Serum Samples to Identify Biomarkers Using Human Proteome Arrays

Parvez Syed[1], Shabarni Gupta[1], Saket Choudhary[2], Narendra Goud Pandala[1], Apurva Atak[1], Annie Richharia[1], Manubhai KP[1], Heng Zhu[3], Sridhar Epari[4], Santosh B. Noronha[2], Aliasgar Moiyadi[5], Sanjeeva Srivastava[1*]

[1]Department of Biosciences and Bioengineering, Indian Institute of Technology Bombay, Powai, Mumbai 400076, India

[2]Department of Chemical Engineering, Indian Institute of Technology Bombay, Powai, Mumbai 400076, India

[3]Department of Pharmacology and Molecular Sciences/High-Throughput Biology Center, Johns Hopkins University School of Medicine, Baltimore, MD, USA

[4]Department of Pathology, Tata Memorial Centre, Mumbai400 012, India

[5]Department of Neurosurgery, Tata Memorial Centre, Mumbai 400 012, India

[*]Correspondence: Dr. Sanjeeva Srivastava, Department of Biosciences and Bioengineering, IIT Bombay, Mumbai 400 076, India: E-mail: sanjeeva@iitb.ac.in
Phone: +91-22-2576-7779, Fax: +91-22-2572-3480

Supplementary Information

Supplementary Tables:

**Table 1S: Experimental details.** The details of the healthy control and Grade II samples.				
Sample ID	Sample Type	File name	Age (years)	Sex
**CF 10729**	Glioma Grade II	Grade_II_CF_10729_2000154012.gpr	45	Female
**CF 20468**	Glioma Grade II	Grade_II_CF_20468_2000153947.gpr	22	Male
**CF 28254**	Glioma Grade II	Grade_II_CF_28254_2000153938.gpr	26	Male
**CF 29538**	Glioma Grade II	Grade_II_CF_29538_2000154020.gpr	32	Male
**CF 8551**	Glioma Grade II	Grade_II_CF_8551_2000153940.gpr	23	Male
**CH 12323**	Glioma Grade II	Grade_II_CH_12323_2000144444.gpr	47	Male
**CH 13717**	Glioma Grade II	Grade_II_CH_13717_2000144447.gpr	47	Male
**CH 27095**	Glioma Grade II	Grade_II_CH_27095_2000154014.gpr		
**CH 32225**	Glioma Grade II	Grade_II_CH_32225_2000154033.gpr	62	Female
**CJ 10242**	Glioma Grade II	Grade_II_CJ_10242_2000144449.gpr	22	Male
**CJ 16084**	Glioma Grade II	Grade_II_CJ_16084_2000153931.gpr	42	Female
**CJ 17734**	Glioma Grade II	Grade_II_CJ_17734_2000154015.gpr		
**CJ 33350**	Glioma Grade II	Grade_II_CJ_33350_2000144452.gpr	55	Male
**CJ 4825**	Glioma Grade II	Grade_II_CJ_4825_2000143981.gpr	17	Male
**CJ 7132**	Glioma Grade II	Grade_II_CJ_7132_2000154028.gpr		
**CJ 863**	Glioma Grade II	Grade_II_CJ_863_2000143970.gpr	32	Female
**CK 40**	Glioma Grade II	Grade_II_CK_40_2000144445.gpr	17	Male
**H-02**	Healthy Control	Control_H-02_2000153953.gpr		
**H-03**	Healthy Control	Control_H-03_2000153943.gpr		
**H-19**	Healthy Control	Control_H-19_2000154008.gpr		
**H-23**	Healthy Control	Control_H-23_2000154009.gpr		
**H-25**	Healthy Control	Control_H-25_2000153952.gpr		
**H-35**	Healthy Control	Control_H-35_2000153942.gpr		
**H-41**	Healthy Control	Control_H-41_2000154026.gpr		
**H-58**	Healthy Control	Control_H-58_2000153935.gpr		
**H-59**	Healthy Control	Control_H-59_2000154016.gpr		
**HC-25**	Healthy Control	Control_HC-25_2000153932.gpr		
**HV-56**	Healthy Control	Control_HV-56_2000155735.gpr		
**HV-59**	Healthy Control	Control_HV-59_2000155740.gpr		
**HV-64**	Healthy Control	Control_HV-64_2000144456.gpr		
**HV-70**	Healthy Control	Control_HV-70_2000144457.gpr		
**HV-71**	Healthy Control	Control_HV-71_2000144458.gpr		

Table 1S: Shortlisted proteins. List of proteins with p-value<0.05 and logFC >0.5 or <-0.5 for the healthy control and Grade II samples.

Block	Row	Column	ID	Name	logFC	AveExpr	t	P.Value	adj.P.Val	B
41	23	27	BC121798	EYA1	-0.56	9.58	-4.03	0.00009	0.55983	0.83
36	4	17	NM_003099.3	SNX1	0.78	9.47	4.00	0.00010	0.55983	0.76
35	14	21	ENST00000361290	MYLK	0.54	8.53	3.49	0.00063	0.71157	-0.67
18	4	11	BC004372.1	CD44	-0.51	7.43	-3.25	0.00139	0.72272	-1.27
16	4	21	NM_003374.1	VDAC1	0.53	8.06	3.25	0.00140	0.72272	-1.27
36	23	21	Nol3	NOL3	-1.35	11.13	-3.03	0.00282	0.91641	-1.79
36	23	23	Lhx1	LHX1	-0.66	8.01	-2.83	0.00517	0.99999	-2.24
33	2	17	BC018747.1	IGHG1	0.66	10.08	2.79	0.00591	0.99999	-2.34
23	16	5	NM_144495.2	PQBP1	-0.50	10.33	-2.59	0.01056	0.99999	-2.76
37	15	23	NM_052849.2	CCDC32	0.58	11.00	2.48	0.01423	0.99999	-2.98
46	14	17	NM_015004.2	EXOSC7	-0.67	9.37	-2.34	0.02065	0.99999	-3.24

Table 1S: Classifiers. List of classifiers and their corresponding statistics for the healthy control and Grade II samples.

NF	Brier	BrierSE	CA	CASE	AUC	IS	Sensitivity	Specificity	PPV	NPV	FeatureList
1	0.40	0.04	0.79	0.06	0.8	0.18	0.94	0.60	0.73	0.90	BC121798
2	0.34	0.04	0.69	0.09	0.825	0.33	0.82	0.53	0.67	0.73	BC121798 BC018747.1
3	0.26	0.09	0.81	0.07	0.95	0.50	0.82	0.80	0.82	0.80	BC121798 BC018747.1 NM_003099.3
4	0.24	0.06	0.87	0.05	0.9	0.54	0.88	0.87	0.88	0.87	BC121798 BC018747.1 NM_003099.3 NM_003374.1
5	0.28	0.08	0.83	0.07	0.95	0.46	0.76	0.93	0.93	0.78	BC121798 BC018747.1 NM_003099.3 NM_003374.1 NM_052849.2
6	0.27	0.05	0.83	0.07	0.95	0.48	0.88	0.80	0.83	0.86	BC121798 BC018747.1 NM_003099.3 NM_003374.1 NM_052849.2 BC004372.1
7	0.19	0.05	0.9	0.05	1	0.61	0.94	0.87	0.89	0.93	BC121798 BC018747.1 NM_003099.3 NM_003374.1 NM_052849.2 BC004372.1 ENST00000361290
8	0.21	0.05	0.83	0.06	0.95	0.59	0.88	0.80	0.83	0.86	BC121798 BC018747.1 NM_003099.3 NM_003374.1 NM_052849.2 BC004372.1 ENST00000361290 NM_144495.2
9	0.26	0.08	0.78	0.08	0.9	0.52	0.88	0.67	0.75	0.83	BC121798 BC018747.1 NM_003099.3 NM_003374.1 NM_052849.2 BC004372.1 ENST00000361290 NM_144495.2 NM_015004.2
10	0.23	0.05	0.87	0.05	0.9	0.51	0.88	0.87	0.88	0.87	BC121798 BC018747.1 NM_003099.3 NM_003374.1 NM_052849.2 BC004372.1 ENST00000361290 NM_144495.2 NM_015004.2 Nol3
11	0.28	0.08	0.8	0.07	0.9	0.49	0.88	0.73	0.79	0.85	BC121798 BC018747.1 NM_003099.3 NM_003374.1 NM_052849.2 BC004372.1 ENST00000361290 NM_144495.2 NM_015004.2 Nol3 Lhx1
12	0.28	0.08	0.8	0.07	0.9	0.49	0.88	0.73	0.79	0.85	BC121798 BC018747.1 NM_003099.3 NM_003374.1 NM_052849.2 BC004372.1 ENST00000361290 NM_144495.2 NM_015004.2 Nol3 Lhx1
13	0.28	0.08	0.8	0.07	0.9	0.49	0.88	0.73	0.79	0.85	BC121798 BC018747.1 NM_003099.3 NM_003374.1 NM_052849.2 BC004372.1 ENST00000361290 NM_144495.2 NM_015004.2 Nol3 Lhx1
14	0.28	0.08	0.8	0.07	0.9	0.49	0.88	0.73	0.79	0.85	BC121798 BC018747.1 NM_003099.3 NM_003374.1 NM_052849.2 BC004372.1 ENST00000361290 NM_144495.2 NM_015004.2 Nol3 Lhx1

15	0.28	0.08	0.8	0.07	0.9	0.49	0.88	0.73	0.79	0.85	BC121798 BC018747.1 NM_003099.3 NM_003374.1 NM_052849.2 BC004372.1 ENST00000361290 NM_144495.2 NM_015004.2 Nol3 Lhx1
16	0.28	0.08	0.8	0.07	0.9	0.49	0.88	0.73	0.79	0.85	BC121798 BC018747.1 NM_003099.3 NM_003374.1 NM_052849.2 BC004372.1 ENST00000361290 NM_144495.2 NM_015004.2 Nol3 Lhx1
17	0.28	0.08	0.8	0.07	0.9	0.49	0.88	0.73	0.79	0.85	BC121798 BC018747.1 NM_003099.3 NM_003374.1 NM_052849.2 BC004372.1 ENST00000361290 NM_144495.2 NM_015004.2 Nol3 Lhx1
18	0.28	0.08	0.8	0.07	0.9	0.49	0.88	0.73	0.79	0.85	BC121798 BC018747.1 NM_003099.3 NM_003374.1 NM_052849.2 BC004372.1 ENST00000361290 NM_144495.2 NM_015004.2 Nol3 Lhx1
19	0.28	0.08	0.8	0.07	0.9	0.49	0.88	0.73	0.79	0.85	BC121798 BC018747.1 NM_003099.3 NM_003374.1 NM_052849.2 BC004372.1 ENST00000361290 NM_144495.2 NM_015004.2 Nol3 Lhx1
20	0.28	0.08	0.8	0.07	0.9	0.49	0.88	0.73	0.79	0.85	BC121798 BC018747.1 NM_003099.3 NM_003374.1 NM_052849.2 BC004372.1 ENST00000361290 NM_144495.2 NM_015004.2 Nol3 Lhx1
21	0.28	0.08	0.8	0.07	0.9	0.49	0.88	0.73	0.79	0.85	BC121798 BC018747.1 NM_003099.3 NM_003374.1 NM_052849.2 BC004372.1 ENST00000361290 NM_144495.2 NM_015004.2 Nol3 Lhx1
22	0.28	0.08	0.8	0.07	0.9	0.49	0.88	0.73	0.79	0.85	BC121798 BC018747.1 NM_003099.3 NM_003374.1 NM_052849.2 BC004372.1 ENST00000361290 NM_144495.2 NM_015004.2 Nol3 Lhx1
23	0.28	0.08	0.8	0.07	0.9	0.49	0.88	0.73	0.79	0.85	BC121798 BC018747.1 NM_003099.3 NM_003374.1 NM_052849.2 BC004372.1 ENST00000361290 NM_144495.2 NM_015004.2 Nol3 Lhx1
24	0.28	0.08	0.8	0.07	0.9	0.49	0.88	0.73	0.79	0.85	BC121798 BC018747.1 NM_003099.3 NM_003374.1 NM_052849.2 BC004372.1 ENST00000361290 NM_144495.2 NM_015004.2 Nol3 Lhx1
25	0.28	0.08	0.8	0.07	0.9	0.49	0.88	0.73	0.79	0.85	BC121798 BC018747.1 NM_003099.3 NM_003374.1 NM_052849.2 BC004372.1 ENST00000361290 NM_144495.2 NM_015004.2 Nol3 Lhx1
26	0.28	0.08	0.8	0.07	0.9	0.49	0.88	0.73	0.79	0.85	BC121798 BC018747.1 NM_003099.3 NM_003374.1 NM_052849.2 BC004372.1 ENST00000361290 NM_144495.2

											NM_015004.2 Nol3 Lhx1
27	0.28	0.08	0.8	0.07	0.9	0.49	0.88	0.73	0.79	0.85	BC121798 BC018747.1 NM_003099.3 NM_003374.1 NM_052849.2 BC004372.1 ENST00000361290 NM_144495.2 NM_015004.2 Nol3 Lhx1
28	0.28	0.08	0.8	0.07	0.9	0.49	0.88	0.73	0.79	0.85	BC121798 BC018747.1 NM_003099.3 NM_003374.1 NM_052849.2 BC004372.1 ENST00000361290 NM_144495.2 NM_015004.2 Nol3 Lhx1
29	0.28	0.08	0.8	0.07	0.9	0.49	0.88	0.73	0.79	0.85	BC121798 BC018747.1 NM_003099.3 NM_003374.1 NM_052849.2 BC004372.1 ENST00000361290 NM_144495.2 NM_015004.2 Nol3 Lhx1
30	0.28	0.08	0.8	0.07	0.9	0.49	0.88	0.73	0.79	0.85	BC121798 BC018747.1 NM_003099.3 NM_003374.1 NM_052849.2 BC004372.1 ENST00000361290 NM_144495.2 NM_015004.2 Nol3 Lhx1

Table 1S: Classifiers. List of 10 classifier proteins for the healthy control and Grade II samples.

ID	Name	logFC	AveExpr	t	P.Value	adj.P.Val	B
BC121798	EYA1	-0.56	9.58	-4.03	0.00009	0.55983	0.83
NM_003099.3	SNX1	0.78	9.47	4.00	0.00010	0.55983	0.76
ENST00000361290	MYLK	0.54	8.53	3.49	0.00063	0.71157	-0.67
BC004372.1	CD44	-0.51	7.43	-3.25	0.00139	0.72272	-1.27
NM_003374.1	VDAC1	0.53	8.06	3.25	0.00140	0.72272	-1.27
Nol3	NOL3	-1.35	11.13	-3.03	0.00282	0.91641	-1.79
BC018747.1	IGHG1	0.66	10.08	2.79	0.00591	0.99999	-2.34
NM_144495.2	PQBP1	-0.50	10.33	-2.59	0.01056	0.99999	-2.76
NM_052849.2	CCDC32	0.58	11.00	2.48	0.01423	0.99999	-2.98
NM_015004.2	EXOSC7	-0.67	9.37	-2.34	0.02065	0.99999	-3.24

**Table 2S: Experimental details.** The details of the healthy control and Grade III samples.				
Sample ID	Sample Type	File name	Age (years)	Sex
CF 6980	Glioma Grade III	Grade_III_CF_12839_2000143982.gpr	27	Female
CF 12839	Glioma Grade III	Grade_III_CF_16216_2000144451.gpr	25	Female
CF 16216	Glioma Grade III	Grade_III_CF_22483_2000144446.gpr	28	Female
CF 22483	Glioma Grade III	Grade_III_CF_22807_2000154035.gpr	33	Female
CF 22807	Glioma Grade III	Grade_III_CF_25539_2000143971.gpr	27	Male
CF 25539	Glioma Grade III	Grade_III_CF_29446_2000144453.gpr	46	Male
CF 29446	Glioma Grade III	Grade_III_CF_6980_2000154032.gpr	55	Female
CH 15130	Glioma Grade III	Grade_III_CH_15130_2000143947.gpr	35	Male
CH 15138	Glioma Grade III	Grade_III_CH_15138_2000143946.gpr	42	Male
CH 17244	Glioma Grade III	Grade_III_CH_17244_2000144448.gpr	28	Female
CH 9010	Glioma Grade III	Grade_III_CH_9010_2000143984.gpr	23	Female
CH 9043	Glioma Grade III	Grade_III_CH_9043_2000144459.gpr	38	Male
CJ 1176	Glioma Grade III	Grade_III_CJ_1176_2000154027.gpr	32	Male
CJ 14116	Glioma Grade III	Grade_III_CJ_14116_2000144450.gpr	54	Male
CJ 16912	Glioma Grade III	Grade_III_CJ_16912_2000154006.gpr	45	Male
CJ 20721	Glioma Grade III	Grade_III_CJ_20721_2000143947.gpr	21	Male
CJ 7833	Glioma Grade III	Grade_III_CJ_7833_2000153939.gpr	25	Female
CJ 9761	Glioma Grade III	Grade_III_CJ_9761_2000143973.gpr	59	Male
H-02	Healthy Control	Control_H-02_2000153953.gpr		
H-03	Healthy Control	Control_H-03_2000153943.gpr		
H-19	Healthy Control	Control_H-19_2000154008.gpr		
H-23	Healthy Control	Control_H-23_2000154009.gpr		
H-25	Healthy Control	Control_H-25_2000153952.gpr		
H-35	Healthy Control	Control_H-35_2000153942.gpr		
H-41	Healthy Control	Control_H-41_2000154026.gpr		
H-58	Healthy Control	Control_H-58_2000153935.gpr		
H-59	Healthy Control	Control_H-59_2000154016.gpr		
HC-25	Healthy Control	Control_HC-25_2000153932.gpr		
HV-56	Healthy Control	Control_HV-56_2000155735.gpr		
HV-59	Healthy Control	Control_HV-59_2000155740.gpr		
HV-64	Healthy Control	Control_HV-64_2000144456.gpr		
HV-70	Healthy Control	Control_HV-70_2000144457.gpr		
HV-71	Healthy Control	Control_HV-71_2000144458.gpr		

Table 2S: Shortlisted proteins. List of proteins with adjusted p-value<0.05 and logFC >0.5 or <-0.5 for the healthy control and Grade III samples.

Block	Row	Column	ID	Name	logFC	AveExpr	t	P.Value	adj.P.Val	B
11	24	5	BC014024.1	TCL1A	-0.66	9.32	-7.01	5.75E-11	7.75E-07	14.51
32	13	5	BC017492.1	COG8	0.64	9.44	6.78	2.06E-10	7.75E-07	13.31
17	4	25	NM_001500.2	GMDS	0.56	8.26	6.77	2.17E-10	7.75E-07	13.26
46	14	13	NM_013347.1	RPA4	0.57	8.73	6.76	2.25E-10	7.75E-07	13.22
19	17	5	BC103840.1	MT1P3	-0.54	9.10	-6.76	2.31E-10	7.75E-07	13.20
23	14	5	ENST00000360373		-0.67	9.15	-6.74	2.57E-10	7.75E-07	13.10
42	5	9	NM_004504.3	HRB	0.69	7.59	6.68	3.44E-10	8.87E-07	12.83
8	3	29	NM_018491.3	CBWD1	0.90	9.45	6.54	7.24E-10	1.63E-06	12.13
42	13	1	NM_000100.2	CSTB	-0.80	9.30	-6.39	1.65E-09	3.21E-06	11.35
23	3	25	NM_015926.3	TEX264	0.51	8.19	6.35	2.03E-09	3.21E-06	11.16
41	23	27	BC121798	EYA1	-0.87	9.58	-6.32	2.39E-09	3.21E-06	11.00
48	13	7	NM_032361.1	THOC3	0.58	9.54	6.29	2.74E-09	3.21E-06	10.87
39	3	15	NM_022453.2	RNF25	0.82	9.32	6.29	2.75E-09	3.21E-06	10.87
36	3	23	NM_003279.2	TNNC2	-0.72	8.85	-6.29	2.76E-09	3.21E-06	10.87
46	13	21	NM_153686.4	LCORL	0.56	9.35	6.28	2.84E-09	3.21E-06	10.84
15	21	19	BC092474.1	PRR16	0.69	9.22	6.26	3.20E-09	3.34E-06	10.73
35	24	11	NM_004095.2	EIF4EBP1	-0.63	8.95	-6.19	4.70E-09	4.31E-06	10.37
19	18	31	NM_005987.2	SPRR1A	-0.76	9.54	-6.1	4.77E-09	4.31E-06	10.35

							8			
48	5	1	BC031469.1	LOC554207	0.50	8.72	6.15	5.56E-09	4.78E-06	10.21
19	15	5	NM_016370.1	RAB9B	-0.55	9.91	-6.12	6.41E-09	5.27E-06	10.07
34	1	5	BC001890.1	GCK	0.56	8.52	6.07	8.40E-09	6.07E-06	9.82
22	23	31	BC053845	HKR1	-0.68	9.43	-6.04	9.90E-09	6.88E-06	9.67
17	23	23	BC110912	IRX4	-0.88	9.21	-6.01	1.17E-08	7.83E-06	9.51
19	17	9	NM_001002848.1	LY6G5C	-0.65	8.63	-5.95	1.58E-08	1.02E-05	9.23
7	6	25	NM_152667.1	NANP	-0.57	9.16	-5.91	1.92E-08	1.20E-05	9.05
42	20	29	NM_053031.2	MYLK	-0.78	9.04	-5.90	1.99E-08	1.20E-05	9.01
5	5	1	NM_138794.1	LYPLAL1	0.51	9.04	5.84	2.69E-08	1.43E-05	8.73
40	1	5	NM_002969.3	MAPK12	0.64	8.11	5.81	3.19E-08	1.60E-05	8.57
30	22	5	BC045826.1	C20orf19	0.63	9.62	5.80	3.37E-08	1.60E-05	8.52
32	12	13	NM_014284.2	NCDN	0.88	8.93	5.79	3.43E-08	1.60E-05	8.50
32	16	7	NM_001002033.1	HN1	-0.70	8.93	-5.78	3.63E-08	1.60E-05	8.45
19	4	9	BC000719.2	ATG4B	0.50	9.27	5.77	3.85E-08	1.60E-05	8.39
32	12	27	BC007660.2	DKK3	0.52	9.40	5.76	3.94E-08	1.60E-05	8.37
15	12	17	ENST00000378470		-0.74	8.92	-5.76	3.97E-08	1.60E-05	8.36
44	5	1	NM_001037535.1	SCML1	0.67	10.05	5.76	3.98E-08	1.60E-05	8.36
10	13	9	NM_022822.1	KLC2	0.73	8.62	5.74	4.41E-08	1.73E-05	8.27
22	24	23	NM_014669.2	N.D.	-0.57	8.24	-5.7	4.61E-08	1.77E-05	8.22

							3			
17	4	29	BC000718.2	DCTN2	0.53	8.35	5.72	4.97E-08	1.78E-05	8.15
42	22	15	XM_001130991.1	LOC729447	-0.59	8.78	-5.71	5.09E-08	1.78E-05	8.13
28	4	15	BC004867.2	DOK3	0.76	9.65	5.71	5.11E-08	1.78E-05	8.13
34	4	11	ENST00000370417	--	0.80	9.23	5.70	5.50E-08	1.88E-05	8.06
35	6	23	NM_012198.2	GCA	0.51	8.81	5.69	5.75E-08	1.90E-05	8.02
46	14	17	NM_015004.2	EXOSC7	-1.62	9.37	-5.69	5.78E-08	1.90E-05	8.01
9	4	13	NM_016095.1	GINS2	0.51	8.62	5.62	8.07E-08	2.47E-05	7.70
32	2	25	BC009561.1	C11orf74	0.62	9.44	5.61	8.45E-08	2.54E-05	7.66
8	6	21	BC029858.1	LRRC23	0.61	8.74	5.58	9.73E-08	2.78E-05	7.52
39	1	13	NM_005500.1	SAE1	0.59	8.98	5.57	1.02E-07	2.78E-05	7.48
12	18	13	NM_012275.2	IL1F5	-0.77	9.46	-5.56	1.06E-07	2.78E-05	7.44
38	24	19	BC029896.1	N.D.	-0.54	8.40	-5.55	1.11E-07	2.88E-05	7.40
3	17	29	NM_001014450.1	SPRR2F	-0.51	9.14	-5.55	1.13E-07	2.88E-05	7.38
22	24	25	BC053624.1	LOC286016	-0.66	9.04	-5.53	1.26E-07	3.16E-05	7.28
10	20	11	NM_018339.3	RFK	-1.29	8.91	-5.52	1.29E-07	3.20E-05	7.26
47	3	25	NM_016246.2	HSD17B14	0.58	8.51	5.46	1.75E-07	4.13E-05	6.98
22	12	5	NM_016823.2	CRK	-0.56	8.68	-5.43	1.98E-07	4.40E-05	6.86
48	19	17	NM_015162.3	ACSBG1	0.53	9.01	5.43	2.00E-07	4.40E-05	6.85
7	17	31	NM_174928.1	N6AMT2	-0.53	9.99	-5.4	2.29E-07	4.77E-05	6.72

							0			
29	23	13	NM_198584.1	CA13	-0.66	8.67	-5.40	2.31E-07	4.77E-05	6.72
39	19	27	NM_032547.1	SCOC	-0.67	9.91	-5.40	2.32E-07	4.77E-05	6.71
36	13	11	BC021192.2	SEPT9	0.59	8.93	5.39	2.35E-07	4.77E-05	6.70
18	13	9	NM_199415.1	UBOX5	0.71	9.72	5.39	2.41E-07	4.81E-05	6.68
38	17	27	NM_138627.2	BCL2L11	-0.57	8.36	-5.39	2.42E-07	4.81E-05	6.67
6	14	23	NM_170678.1	ITGB1BP3	-0.59	9.77	-5.38	2.47E-07	4.84E-05	6.65
28	20	1	NM_001018025.2	MTCP1	-0.74	9.07	-5.38	2.52E-07	4.85E-05	6.63
25	23	13	NM_138362.1	FAM104B	-0.65	8.20	-5.36	2.75E-07	5.09E-05	6.55
4	15	25	NM_173080.1	SPRR4	-0.55	9.01	-5.36	2.79E-07	5.09E-05	6.54
40	14	29	NM_176870.1	MT1M	-0.58	9.08	-5.35	2.92E-07	5.28E-05	6.50
40	17	31	NM_181337.2	KAAG1	-0.62	9.48	-5.34	2.98E-07	5.33E-05	6.48
28	1	3	BC015547.1	UCK1	0.52	8.83	5.33	3.19E-07	5.60E-05	6.41
23	16	7	NM_176806.2	MOCS2	-0.51	8.34	-5.33	3.23E-07	5.60E-05	6.40
15	3	27	BC004130.2	CALCOCO2	0.68	9.20	5.30	3.58E-07	6.05E-05	6.31
32	13	21	BC069328.1	BMF	0.57	10.04	5.29	3.80E-07	6.25E-05	6.25
41	23	29	BC125062	ARNTL2	-0.59	8.90	-5.27	4.19E-07	6.62E-05	6.16
3	10	29	NM_002086.3	GRB2	-0.66	10.19	-5.26	4.43E-07	6.79E-05	6.11

39	19	3	NM_005335.3	HCLS1	0.76	9.40	5.24	4.84E-07	7.23E-05	6.02
46	17	27	NM_001009956.1	ZNF655	-0.64	9.45	-5.23	5.04E-07	7.46E-05	5.99
19	6	17	NM_005388.3	PDCL	0.75	9.84	5.22	5.27E-07	7.67E-05	5.95
31	20	11	NM_006148.1	LASP1	-0.59	9.24	-5.21	5.61E-07	7.92E-05	5.89
36	18	31	BC001241.1	ARHGAP17	-0.60	8.71	-5.19	5.98E-07	8.31E-05	5.83
9	14	11	BC058007.1	MUC15	0.85	8.77	5.19	6.06E-07	8.35E-05	5.82
32	1	25	BC012010.1	SAAL1	0.71	10.28	5.17	6.66E-07	9.11E-05	5.73
3	17	9	NM_175622.3	MT1JP	-0.55	9.29	-5.15	7.47E-07	9.72E-05	5.62
14	24	23	BC033854.1	N.D.	-0.60	9.09	-5.14	7.53E-07	9.72E-05	5.61
15	13	9	BC064834.1	LRBA	0.52	8.54	5.10	9.10E-07	0.000115	5.44
42	12	13	BC032412.1	BC032412.1	0.61	8.97	5.10	9.33E-07	0.000117	5.41
34	6	9	NM_006304.1	SHFM1	-0.65	8.59	-5.10	9.39E-07	0.000117	5.41
24	24	25	NM_019857.3	N.D.	-0.54	9.00	-5.10	9.43E-07	0.000117	5.40
42	14	7	NM_174895.1	PCP2	-0.52	10.15	-5.09	9.79E-07	0.000119	5.37
4	5	7	NM_005185.2	CALML3	-0.66	8.94	-5.08	1.00E-06	0.00012	5.35
41	22	1	BC075862.1	CAPN1	-0.51	8.61	-5.06	1.10E-06	0.000129	5.26
4	16	27	NM_152901.1	PYDC1	-0.52	9.73	-5.05	1.14E-06	0.000132	5.23
46	12	21	BC007439.2	DCP1A	0.52	8.97	5.04	1.20E-06	0.000137	5.18

9	24	19	NM_152308.1	C16orf75	-0.84	8.45	-5.03	1.26E-06	0.000141	5.13
42	15	7	NM_003746.2	DYNLL1	-0.53	8.63	-5.01	1.37E-06	0.000151	5.05
9	24	5	NM_198584.1	CA13	-0.75	8.93	-5.00	1.42E-06	0.000156	5.02
9	2	25	NM_006360.3	PCID1	0.63	9.68	4.99	1.53E-06	0.000164	4.95
33	2	17	BC018747.1	IGHG1	1.17	10.08	4.99	1.54E-06	0.000164	4.95
17	19	9	NM_000577.3	IL1RN	-0.64	9.29	-4.98	1.62E-06	0.00017	4.90
33	14	15	NM_012278.1	ITGB1BP2	-1.20	8.97	-4.97	1.63E-06	0.00017	4.89
17	13	21	NM_138468.3	ICA1L	-0.54	8.42	-4.95	1.80E-06	0.000183	4.80
24	12	11	BC012797.2	CNKSR1	0.59	9.01	4.94	1.94E-06	0.000194	4.73
17	23	15	NM_152678.1	FAM116A	-0.52	8.09	-4.93	1.95E-06	0.000194	4.72
31	6	23	NM_017924.2	C14orf119	0.67	9.15	4.93	1.97E-06	0.000194	4.72
14	17	1	BC045799.1	ZNF428	-0.61	8.57	-4.92	2.10E-06	0.000207	4.66
40	3	21	NM_182789.2	PAIP1	0.59	8.90	4.92	2.12E-06	0.000207	4.65
18	13	7	NM_016143.3	NSFL1C	-0.61	8.91	-4.91	2.20E-06	0.000211	4.62
28	11	11	NM_005953.2	MT2A	-0.51	9.17	-4.90	2.29E-06	0.000219	4.58
28	13	9	NM_173608.1	C14orf80	0.55	8.37	4.89	2.36E-06	0.000223	4.55
28	21	31	BC101135.1	FERD3L	-0.51	8.36	-4.88	2.49E-06	0.00023	4.50
36	23	9	NM_001001561.1	GGA1	-0.55	10.55	-4.8	2.51E-06	0.000231	4.49

							8			
22	24	19	NM_006112.2	PPIE	-0.61	8.56	-4.87	2.63E-06	0.000237	4.45
21	2	5	BC018269.1	KIAA1967	0.68	8.60	4.86	2.74E-06	0.000242	4.41
26	5	3	BC004405.1	BC004405.1	-0.54	8.68	-4.84	3.01E-06	0.00026	4.32
18	23	13	NM_032194.1	BXDC1	-0.52	10.07	-4.83	3.05E-06	0.000262	4.31
28	13	19	BC066304.1	LGALS13	-0.64	9.18	-4.82	3.20E-06	0.000272	4.26
47	2	21	NM_001031677.2	RAB24	0.59	8.77	4.79	3.65E-06	0.000304	4.14
1	4	7	NM_000790.2	DDC	0.61	8.77	4.79	3.73E-06	0.000306	4.12
30	13	25	NM_000496.2	CRYBB2	-0.54	8.53	-4.74	4.51E-06	0.000351	3.95
36	4	17	NM_003099.3	SNX1	0.91	9.47	4.73	4.71E-06	0.000359	3.91
46	1	31	NM_001878.2	CRABP2	-0.94	8.49	-4.73	4.87E-06	0.000366	3.88
5	23	9	NM_002899.2	RBP1	-0.64	8.73	-4.72	4.93E-06	0.000368	3.86
22	16	13	NM_183041.1	DTNBP1	0.59	9.90	4.72	5.06E-06	0.000376	3.84
3	17	21	NM_005950.1	MT1G	-0.61	9.33	-4.70	5.36E-06	0.000383	3.79
11	19	21	ENST00000371141		-0.53	9.62	-4.70	5.38E-06	0.000383	3.78
35	24	25	NM_032531.2	N.D.	-0.55	8.82	-4.68	6.01E-06	0.000418	3.68
40	22	21	BC014225.2	HOP	-0.53	9.08	-4.65	6.67E-06	0.000445	3.58
20	23	15	BC026965.1	ARHGEF10	-0.56	9.68	-4.61	7.86E-06	0.000499	3.43

46	13	25	NM_002822.3	TWF1	0.83	7.97	4.61	7.93E-06	0.000501	3.42
41	24	23	NM_198428.2	N.D.	-0.60	9.69	-4.60	8.23E-06	0.000513	3.39
48	21	23	BC099845.1	RAP1GDS1	0.54	9.91	4.54	1.07E-05	0.000618	3.15
13	22	5	BC063432.1	TRA@	-0.52	9.03	-4.53	1.13E-05	0.000644	3.10
30	23	3	NM_007193.3	ANXA10	-0.56	9.85	-4.51	1.21E-05	0.000676	3.04
46	15	19	NM_130844.1	WWOX	-0.78	9.37	-4.51	1.24E-05	0.000688	3.01
13	13	5	NM_130776.1	XAGE3	-0.51	8.95	-4.49	1.32E-05	0.00072	2.95
11	13	9	NM_000884.2	IMPDH2	0.55	8.62	4.47	1.43E-05	0.000759	2.88
21	21	31	BC090928.1	SLAMF6	-0.56	9.12	-4.43	1.68E-05	0.00084	2.73
44	12	25	NM_152296.3	ATP1A3	0.81	8.43	4.43	1.69E-05	0.00084	2.72
26	12	27	NM_138357.1	CCDC109A	-0.52	9.02	-4.43	1.71E-05	0.000844	2.71
2	1	21	NM_004330.1	BNIP2	0.64	8.37	4.43	1.71E-05	0.000844	2.71
33	24	3	nd	nd	-0.58	8.28	-4.43	1.73E-05	0.000848	2.70
34	4	13	BC004889.2	USP5	0.52	9.04	4.42	1.76E-05	0.000855	2.68
48	14	17	NM_006634.2	VAMP5	-0.61	8.28	-4.39	2.05E-05	0.000951	2.54
41	24	13	NM_032439.1	PHYHIPL	-0.66	9.83	-4.31	2.75E-05	0.001205	2.28
12	14	9	NM_182498.2	ZNF428	-0.66	8.82	-4.28	3.14E-05	0.00134	2.15
21	4	5	NM_000026.1	ADSL	0.51	9.02	4.28	3.16E-05	0.001344	2.15

4	14	21	NM_130467.3	PAGE5	-0.53	8.57	-4.26	3.45E-05	0.00143	2.07
46	20	31	NM_031431.2	COG3	0.50	7.64	4.26	3.45E-05	0.00143	2.07
43	2	15	BC017723.1	MAGEA4	0.55	9.83	4.26	3.48E-05	0.001431	2.06
20	10	3	BC000870.1	TIPIN	0.53	8.87	4.22	3.99E-05	0.001568	1.93
2	19	21	NM_052965.1	C1orf19	-0.66	10.45	-4.20	4.30E-05	0.001651	1.86
2	14	25	BC062684.1	SCOC	-0.68	10.24	-4.20	4.35E-05	0.001662	1.85
43	4	5	NM_002853.3	RAD1	0.52	8.47	4.20	4.40E-05	0.001675	1.84
24	4	21	BC006402.1	IGHG1	0.73	10.16	4.18	4.81E-05	0.001792	1.76
32	8	21	NM_005207.2	CRKL	-0.52	9.58	-4.16	5.04E-05	0.001852	1.72
16	20	11	NM_003152.2	STAT5A	0.50	9.24	4.14	5.60E-05	0.002011	1.62
31	4	3	NM_002857.2	PEX19	0.70	9.75	4.13	5.65E-05	0.002023	1.61
44	20	29	NM_005416.1	SPRR3	-0.61	10.58	-4.13	5.68E-05	0.002025	1.61
2	18	27	NM_001024382.1	HMBS	-0.51	8.64	-4.13	5.80E-05	0.002049	1.59
17	2	1	NM_032331.2	MGC2408	0.68	9.99	4.12	6.05E-05	0.002123	1.55
23	16	5	NM_144495.2	PQBP1	-0.79	10.33	-4.11	6.12E-05	0.002142	1.54
11	20	23	BC073755.1	ANXA8L1	-0.54	9.11	-4.10	6.54E-05	0.002265	1.48
22	24	17	BC029886.1	N.D.	-0.68	8.21	-4.08	7.10E-05	0.002396	1.40
5	3	27	BC001103.2	GULP1	0.57	8.98	4.06	7.42E-05	0.002487	1.36
10	9	5	BC015848.1	GLOD4	-1.01	8.89	-4.0	8.12E-05	0.002642	1.28

							4			
28	1	13	NM_031361.1	COL4A3BP	0.53	8.89	4.0<br>2	8.71E-<br>05	0.00276<br>4	1.21
42	12	25	NM_002482.2	NASP	0.66	8.37	4.0<br>2	8.72E-<br>05	0.00276<br>4	1.21
46	20	29	NM_020532.4	RTN4	0.60	7.74	3.9<br>6	0.00011	0.00328<br>9	1.00
47	20	17	BC004128.2	EFHD1	0.62	9.87	3.9<br>5	0.00011<br>6	0.00344<br>4	0.95
46	13	23	NM_203284.1	RBPJ	1.11	8.72	3.9<br>4	0.00012	0.00353	0.92
27	14	23	NM_018584.4	CAMK2N1	-<br>1.24	8.93	-<br>3.9<br>1	0.00013<br>3	0.00380<br>3	0.83
38	4	1	BC002660.2	TMOD1	0.66	8.57	3.8<br>8	0.00015	0.00414<br>4	0.71
35	15	13	NM_001025266.<br>1	LOC28538<br>2	0.92	8.41	3.8<br>7	0.00015<br>6	0.00424<br>8	0.68
8	2	15	BC019337.1	IGHG1	0.53	10.72	3.8<br>2	0.00018<br>9	0.00482<br>1	0.51
29	21	29	BC088364.1	RAD23A	-<br>0.50	9.14	-<br>3.8<br>2	0.00018<br>9	0.00482<br>1	0.50
36	1	15	BC013992.1	MAPK3	0.51	9.01	3.7<br>8	0.00022	0.00539<br>1	0.37
42	3	7	NM_000184.2	HBG2	0.54	9.18	3.6<br>7	0.00032<br>2	0.00715<br>5	0.02
31	13	25	NM_130809.2	PRRC1	0.52	10.03	3.6<br>6	0.00033<br>4	0.00732<br>1	-<br>0.01
16	19	21	NM_016224.3	SNX9	1.20	8.49	3.6<br>6	0.00033<br>8	0.00737<br>5	-<br>0.02
1	4	5	NM_016630.3	SPG21	0.50	8.24	3.6<br>5	0.00035	0.00756<br>3	-<br>0.05
27	15	29	NM_198086.1	JUB	-<br>0.84	8.68	-<br>3.5<br>6	0.00048<br>5	0.00953<br>9	-<br>0.35
39	1	31	BC013009.2	ZMYM3	0.76	10.13	3.5<br>5	0.00050<br>3	0.00978<br>8	-<br>0.38
6	2	11	NM_145055.2	C18orf25	0.54	8.74	3.5<br>5	0.00050<br>4	0.00979<br>4	-<br>0.38
20	22	25	BC065378.1	STK24	0.56	9.04	3.4<br>8	0.00064<br>5	0.01169<br>1	-<br>0.61
38	13	11	NM_198204.1	MLX	0.51	9.58	3.4<br>7	0.00066<br>5	0.01200<br>8	-<br>0.64
21	21	1	NM_052942.2	GBP5	0.66	10.28	3.4<br>5	0.00071<br>7	0.01266<br>8	-<br>0.70
39	14	7	BC060792.1	ACBD3	0.51	8.79	3.4	0.00074	0.01300	-

							4	3	3	0.73
17	2	5	NM_001444.1	FABP5	0.58	9.97	3.43	0.000757	0.01321	-0.75
7	15	11	XM_208990.4	LOC284067	0.61	7.99	3.35	0.000997	0.016279	-1.00
7	15	9	NM_203283.1	RBPJ	1.00	9.04	3.35	0.001005	0.01638	-1.01
25	18	9	NM_001167858.1	PPP1R12B	-0.61	9.64	-3.31	0.001132	0.018053	-1.11
28	19	31	NM_018955.2	UBB	-0.50	8.15	-3.31	0.001133	0.018056	-1.11
19	21	9	BC041857.1	R3HDM2	0.50	8.35	3.25	0.001413	0.021498	-1.31
38	20	1	NM_001032296.2	STK24	0.97	9.68	3.25	0.00142	0.021597	-1.32
36	17	21	BC067101.1	MYOM3	-0.51	8.01	-3.17	0.001823	0.025856	-1.54
48	17	25	NM_019088.2	PAF1	0.53	9.41	3.09	0.002323	0.030673	-1.76
27	14	19	NM_002767.2	PRPSAP2	-0.70	8.43	-3.04	0.00271	0.034019	-1.89
27	15	7	XM_290842.4	LRFN1	-0.55	8.19	-3.03	0.00282	0.03509	-1.93
27	21	31	NM_001003892.1	DUPD1	0.65	8.24	3.03	0.002835	0.035181	-1.93
27	14	17	NM_148910.2	TIRAP	-0.57	8.30	-3.03	0.002848	0.035281	-1.94
1	18	17	NM_004696.1	SLC16A4	0.84	9.05	2.98	0.00327	0.038783	-2.06
32	6	27	NM_032347.1	ZNF397	0.56	9.82	2.95	0.003672	0.042248	-2.16

Table 2S: Classifiers. List of classifiers and their corresponding statistics for the healthy control and Grade III samples.

NF	Brier	BrierSE	CA	CASE	AUC	IS	Sensitivity	Specificity	PPV	NPV	FeatureList
1	0.30	0.07	0.73	0.07	0.88	0.38	0.78	0.67	0.74	0.71	NM_138794.1
2	0.25	0.06	0.79	0.07	0.88	0.48	0.83	0.73	0.79	0.79	NM_138794.1<br>BC001890.1
3	0.20	0.06	0.94	0.04	0.90	0.54	0.89	1.00	1.00	0.88	NM_138794.1<br>BC001890.1<br>NM_003099.3
4	0.18	0.07	0.84	0.07	0.95	0.62	0.78	0.93	0.93	0.78	NM_138794.1<br>BC001890.1<br>NM_003099.3<br>NM_015926.3
5	0.14	0.06	0.90	0.07	0.95	0.67	0.89	0.93	0.94	0.88	NM_138794.1<br>BC001890.1<br>NM_003099.3<br>NM_015926.3<br>NM_016246.2
6	0.13	0.05	0.97	0.03	0.95	0.70	0.94	1.00	1.00	0.94	NM_138794.1<br>BC001890.1<br>NM_003099.3<br>NM_015926.3<br>NM_016246.2<br>NM_198204.1
7	0.13	0.06	0.93	0.04	0.95	0.69	0.89	1.00	1.00	0.88	NM_138794.1<br>BC001890.1<br>NM_003099.3<br>NM_015926.3<br>NM_016246.2<br>NM_198204.1<br>NM_020532.4
8	0.11	0.04	1.00	0.00	1.00	0.69	1.00	1.00	1.00	1.00	NM_138794.1<br>BC001890.1<br>NM_003099.3<br>NM_015926.3<br>NM_016246.2<br>NM_198204.1<br>NM_020532.4<br>BC017723.1
9	0.11	0.04	0.93	0.04	1.00	0.71	0.89	1.00	1.00	0.88	NM_138794.1<br>BC001890.1<br>NM_003099.3<br>NM_015926.3<br>NM_016246.2<br>NM_198204.1

											NM_020532.4<br>BC017723.1<br>BC001241.1
10	0.10	0.03	0.93	0.04	1.00	0.72	0.89	1.00	1.00	0.88	NM_138794.1<br>BC001890.1<br>NM_003099.3<br>NM_015926.3<br>NM_016246.2<br>NM_198204.1<br>NM_020532.4<br>BC017723.1<br>BC001241.1<br>NM_173608.1
11	0.07	0.02	1.00	0.00	1.00	0.78	1.00	1.00	1.00	1.00	NM_138794.1<br>BC001890.1<br>NM_003099.3<br>NM_015926.3<br>NM_016246.2<br>NM_198204.1<br>NM_020532.4<br>BC017723.1<br>BC001241.1<br>NM_173608.1<br>BC121798
12	0.07	0.02	0.97	0.03	1.00	0.77	0.94	1.00	1.00	0.94	NM_138794.1<br>BC001890.1<br>NM_003099.3<br>NM_015926.3<br>NM_016246.2<br>NM_198204.1<br>NM_020532.4<br>BC017723.1<br>BC001241.1<br>NM_173608.1<br>BC121798<br>NM_005500.1
13	0.06	0.02	0.97	0.03	1.00	0.81	0.94	1.00	1.00	0.94	NM_138794.1<br>BC001890.1<br>NM_003099.3<br>NM_015926.3<br>NM_016246.2<br>NM_198204.1<br>NM_020532.4<br>BC017723.1<br>BC001241.1<br>NM_173608.1<br>BC121798<br>NM_005500.1

											NM_006634.2
14	0.06	0.02	0.97	0.03	1.00	0.<br>80	0.94	1.00	1.0<br>0	0.94	NM_138794.1<br>BC001890.1<br>NM_003099.3<br>NM_015926.3<br>NM_016246.2<br>NM_198204.1<br>NM_020532.4<br>BC017723.1<br>BC001241.1<br>NM_173608.1<br>BC121798<br>NM_005500.1<br>NM_006634.2<br>BC014024.1
15	0.06	0.02	0.97	0.03	1.00	0.<br>79	0.94	1.00	1.0<br>0	0.94	NM_138794.1<br>BC001890.1<br>NM_003099.3<br>NM_015926.3<br>NM_016246.2<br>NM_198204.1<br>NM_020532.4<br>BC017723.1<br>BC001241.1<br>NM_173608.1<br>BC121798<br>NM_005500.1<br>NM_006634.2<br>BC014024.1<br>BC021192.2
16	0.05	0.02	1.00	0.00	1.00	0.<br>82	1.00	1.00	1.0<br>0	1.00	NM_138794.1<br>BC001890.1<br>NM_003099.3<br>NM_015926.3<br>NM_016246.2<br>NM_198204.1<br>NM_020532.4<br>BC017723.1<br>BC001241.1<br>NM_173608.1<br>BC121798<br>NM_005500.1<br>NM_006634.2<br>BC014024.1<br>BC021192.2<br>ENST0000036037<br>3

17	0.05	0.01	1.00	0.00	1.00	0. 82	1.00	1.00	1.0 0	1.00	NM_138794.1 BC001890.1 NM_003099.3 NM_015926.3 NM_016246.2 NM_198204.1 NM_020532.4 BC017723.1 BC001241.1 NM_173608.1 BC121798 NM_005500.1 NM_006634.2 BC014024.1 BC021192.2 ENST0000036037 3 NM_032361.1
18	0.04	0.01	1.00	0.00	1.00	0. 84	1.00	1.00	1.0 0	1.00	NM_138794.1 BC001890.1 NM_003099.3 NM_015926.3 NM_016246.2 NM_198204.1 NM_020532.4 BC017723.1 BC001241.1 NM_173608.1 BC121798 NM_005500.1 NM_006634.2 BC014024.1 BC021192.2 ENST0000036037 3 NM_032361.1 BC045799.1
19	0.03	0.01	1.00	0.00	1.00	0. 85	1.00	1.00	1.0 0	1.00	NM_138794.1 BC001890.1 NM_003099.3 NM_015926.3 NM_016246.2 NM_198204.1 NM_020532.4 BC017723.1 BC001241.1 NM_173608.1 BC121798 NM_005500.1 NM_006634.2

											BC014024.1 BC021192.2 ENST0000036037 3 NM_032361.1 BC045799.1 NM_005953.2
20	0.04	0.01	1.00	0.00	1.00	0. 84	1.00	1.00	1.0 0	1.00	NM_138794.1 BC001890.1 NM_003099.3 NM_015926.3 NM_016246.2 NM_198204.1 NM_020532.4 BC017723.1 BC001241.1 NM_173608.1 BC121798 NM_005500.1 NM_006634.2 BC014024.1 BC021192.2 ENST0000036037 3 NM_032361.1 BC045799.1 NM_005953.2 NM_032547.1
21	0.04	0.01	1.00	0.00	1.00	0. 84	1.00	1.00	1.0 0	1.00	NM_138794.1 BC001890.1 NM_003099.3 NM_015926.3 NM_016246.2 NM_198204.1 NM_020532.4 BC017723.1 BC001241.1 NM_173608.1 BC121798 NM_005500.1 NM_006634.2 BC014024.1 BC021192.2 ENST0000036037 3 NM_032361.1 BC045799.1 NM_005953.2 NM_032547.1 BC026965.1

22	0.04	0.01	1.00	0.00	1.00	0.83	1.00	1.00	1.00	1.00	NM_138794.1 BC001890.1 NM_003099.3 NM_015926.3 NM_016246.2 NM_198204.1 NM_020532.4 BC017723.1 BC001241.1 NM_173608.1 BC121798 NM_005500.1 NM_006634.2 BC014024.1 BC021192.2 ENST00000360373 NM_032361.1 BC045799.1 NM_005953.2 NM_032547.1 BC026965.1 BC018747.1
23	0.04	0.01	1.00	0.00	1.00	0.83	1.00	1.00	1.00	1.00	NM_138794.1 BC001890.1 NM_003099.3 NM_015926.3 NM_016246.2 NM_198204.1 NM_020532.4 BC017723.1 BC001241.1 NM_173608.1 BC121798 NM_005500.1 NM_006634.2 BC014024.1 BC021192.2 ENST00000360373 NM_032361.1 BC045799.1 NM_005953.2 NM_032547.1 BC026965.1 BC018747.1 BC009561.1
24	0.05	0.02	1.00	0.00	1.00	0.80	1.00	1.00	1.00	1.00	NM_138794.1 BC001890.1 NM_003099.3

											NM_015926.3<br>NM_016246.2<br>NM_198204.1<br>NM_020532.4<br>BC017723.1<br>BC001241.1<br>NM_173608.1<br>BC121798<br>NM_005500.1<br>NM_006634.2<br>BC014024.1<br>BC021192.2<br>ENST0000036037<br>3 NM_032361.1<br>BC045799.1<br>NM_005953.2<br>NM_032547.1<br>BC026965.1<br>BC018747.1<br>BC009561.1<br>NM_032439.1
25	0.04	0.01	1.00	0.00	1.00	0.82	1.00	1.00	1.00	1.00	NM_138794.1<br>BC001890.1<br>NM_003099.3<br>NM_015926.3<br>NM_016246.2<br>NM_198204.1<br>NM_020532.4<br>BC017723.1<br>BC001241.1<br>NM_173608.1<br>BC121798<br>NM_005500.1<br>NM_006634.2<br>BC014024.1<br>BC021192.2<br>ENST0000036037<br>3 NM_032361.1<br>BC045799.1<br>NM_005953.2<br>NM_032547.1<br>BC026965.1<br>BC018747.1<br>BC009561.1<br>NM_032439.1<br>BC012010.1
26	0.04	0.01	1.00	0.00	1.00	0.84	1.00	1.00	1.00	1.00	NM_138794.1<br>BC001890.1

											NM_003099.3 NM_015926.3 NM_016246.2 NM_198204.1 NM_020532.4 BC017723.1 BC001241.1 NM_173608.1 BC121798 NM_005500.1 NM_006634.2 BC014024.1 BC021192.2 ENST0000036037 3 NM_032361.1 BC045799.1 NM_005953.2 NM_032547.1 BC026965.1 BC018747.1 BC009561.1 NM_032439.1 BC012010.1 NM_175622.3
27	0.04	0.01	1.00	0.00	1.00	0. 83	1.00	1.00	1.0 0	1.00	NM_138794.1 BC001890.1 NM_003099.3 NM_015926.3 NM_016246.2 NM_198204.1 NM_020532.4 BC017723.1 BC001241.1 NM_173608.1 BC121798 NM_005500.1 NM_006634.2 BC014024.1 BC021192.2 ENST0000036037 3 NM_032361.1 BC045799.1 NM_005953.2 NM_032547.1 BC026965.1 BC018747.1 BC009561.1 NM_032439.1

											BC012010.1 NM_175622.3 BC125062
28	0.04	0.01	1.00	0.00	1.00	0. 82	1.00	1.00	1.0 0	1.00	NM_138794.1 BC001890.1 NM_003099.3 NM_015926.3 NM_016246.2 NM_198204.1 NM_020532.4 BC017723.1 BC001241.1 NM_173608.1 BC121798 NM_005500.1 NM_006634.2 BC014024.1 BC021192.2 ENST0000036037 3 NM_032361.1 BC045799.1 NM_005953.2 NM_032547.1 BC026965.1 BC018747.1 BC009561.1 NM_032439.1 BC012010.1 NM_175622.3 BC125062 BC004405.1
29	0.04	0.01	1.00	0.00	1.00	0. 82	1.00	1.00	1.0 0	1.00	NM_138794.1 BC001890.1 NM_003099.3 NM_015926.3 NM_016246.2 NM_198204.1 NM_020532.4 BC017723.1 BC001241.1 NM_173608.1 BC121798 NM_005500.1 NM_006634.2 BC014024.1 BC021192.2 ENST0000036037 3 NM_032361.1

											BC045799.1 NM_005953.2 NM_032547.1 BC026965.1 BC018747.1 BC009561.1 NM_032439.1 BC012010.1 NM_175622.3 BC125062 BC004405.1 NM_016224.3
30	0.04	0.01	1.00	0.00	1.00	0.83	1.00	1.00	1.00	1.00	NM_138794.1 BC001890.1 NM_003099.3 NM_015926.3 NM_016246.2 NM_198204.1 NM_020532.4 BC017723.1 BC001241.1 NM_173608.1 BC121798 NM_005500.1 NM_006634.2 BC014024.1 BC021192.2 ENST00000360373 NM_032361.1 BC045799.1 NM_005953.2 NM_032547.1 BC026965.1 BC018747.1 BC009561.1 NM_032439.1 BC012010.1 NM_175622.3 BC125062 BC004405.1 NM_016224.3 NM_001001561.1

**Table 2S: Classifiers.** List of 10 classifier proteins for the healthy control and Grade III samples.								
ID	Name	logFC	AveExpr	t	P.Value	adj.P.Val	B	Regulation
NM_003099.3	SNX1	0.91	9.47	4.73	4.71E-06	0.000359	3.91	Up
NM_020532.4	RTN4	0.60	7.74	3.96	0.00011	0.003289	1.00	Up
NM_016246.2	HSD17B14	0.58	8.51	5.46	1.75E-07	4.13E-05	6.98	Up
BC001890.1	GCK	0.56	8.52	6.07	8.40E-09	6.07E-06	9.82	Up
BC017723.1	MAGEA4	0.55	9.83	4.26	3.48E-05	0.001431	2.06	Up
NM_173608.1	C14orf80	0.55	8.37	4.89	2.36E-06	0.000223	4.55	Up
NM_198204.1	MLX	0.51	9.58	3.47	0.000665	0.012008	-0.64	Up
NM_138794.1	LYPLAL1	0.51	9.04	5.84	2.69E-08	1.43E-05	8.73	Up
NM_015926.3	TEX264	0.51	8.19	6.35	2.03E-09	3.21E-06	11.16	Up
BC001241.1	ARHGAP17	-0.60	8.71	-5.19	5.98E-07	8.31E-05	5.83	Down

**Table 3S: Experimental details.** The details of the healthy control and Grade IV samples.				
Sample ID	Sample Type	File name	Age (years)	Sex
**CF 11518**	Glioma Grade IV SVZ negative	Grade_IV_CF_11518_2000153942.gpr	57	Female
**CF 11940**	Glioma Grade IV SVZ positive	Grade_IV_CF_11940_2000153779.gpr	59	Male
**CF 12166**	Glioma Grade IV SVZ positive	Grade_IV_CF_12166_2000153934.gpr	64	Male
**CF 19257**	Glioma Grade IV SVZ negative	Grade_IV_CF_19257_2000155723.gpr	60	Male
**CF 27891**	Glioma Grade IV SVZ negative	Grade_IV_CF_27891_2000153776.gpr	64	Male
**CF 7522**	Glioma Grade IV SVZ positive	Grade_IV_CF_7522_2000154010.gpr	20	Female
**CF 7916**	Glioma Grade IV SVZ positive	Grade_IV_CF_7916_2000154021.gpr	56	Male
**CH 12724**	Glioma Grade IV SVZ negative	Grade_IV_CH_12724_2000153778.gpr	60	Male
**CH 14820**	Glioma Grade IV SVZ positive	Grade_IV_CH_14820_2000153796.gpr	57	Male
**CH 18016**	Glioma Grade IV SVZ negative	Grade_IV_CH_18016_2000153791.gpr	40	Male
**CH 18426**	Glioma Grade IV SVZ positive	Grade_IV_CH_18426_2000153757.gpr	56	Male
**CH 21783**	Glioma Grade IV SVZ positive	Grade_IV_CH_21783_2000153785.gpr	47	Male
**CH 22618**	Glioma Grade IV SVZ positive	Grade_IV_CH_22618_2000153782.gpr	53	Male
**CH 28569**	Glioma Grade IV SVZ positive	Grade_IV_CH_28569_2000153799.gpr	38	Female
**CH 29697**	Glioma Grade IV SVZ positive	Grade_IV_CH_29697_2000153759.gpr	60	Male
**CH 29738**	Glioma Grade IV SVZ positive	Grade_IV_CH_29738_2000153798.gpr	46	Male
**CH 30203**	Glioma Grade IV SVZ positive	Grade_IV_CH_30203_2000153793.gpr	61	Female
**CH 3969**	Glioma Grade IV SVZ positive	Grade_IV_CH_3969_2000153950.gpr	46	Female
**CH 4996**	Glioma Grade IV SVZ positive	Grade_IV_CH_4996_2000153933.gpr	57	Male
**CH 5261**	Glioma Grade IV SVZ negative	Grade_IV_CH_5261_2000155722.gpr	30	Male
**CH 6643**	Glioma Grade IV SVZ positive	Grade_IV_CH_6643_2000153758.gpr	47	Male
**CH 8498**	Glioma Grade IV SVZ negative	Grade_IV_CH_8498_2000153777.gpr	52	Male
**CH 9002**	Glioma Grade IV SVZ positive	Grade_IV_CH_9002_2000153954.gpr	69	Male
**CJ 10048**	Glioma Grade IV SVZ negative	Grade_IV_CJ_10048_2000155725.gpr	37	Male
**CJ 11291**	Glioma Grade IV SVZ positive	Grade_IV_CJ_11291_2000154025.gpr	51	Female
**CJ 12441**	Glioma Grade IV SVZ	Grade_IV_CJ_12441_2000153760.g	58	Male

	negative	pr		
**CJ 13655**	Glioma Grade IV SVZ positive	Grade_IV_CJ_13655_2000153783.gpr	68	Female
**CJ 1519**	Glioma Grade IV SVZ positive	Grade_IV_CJ_1519_2000153951.gpr	55	Female
**CJ 18704**	Glioma Grade IV SVZ negative	Grade_IV_CJ_18704_2000153784.gpr	65	Male
**CJ 2041**	Glioma Grade IV SVZ negative	Grade_IV_CJ_2041_2000154019.gpr	58	Male
**CJ 22776**	Glioma Grade IV SVZ positive	Grade_IV_CJ_22776_2000153756.gpr	61	Male
**CJ 30014**	Glioma Grade IV SVZ positive	Grade_IV_CJ_30014_2000153805.gpr	64	Male
**CJ 32705**	Glioma Grade IV SVZ negative	Grade_IV_CJ_32705_2000153797.gpr	59	Male
**CJ 6254**	Glioma Grade IV SVZ negative	Grade_IV_CJ_6254_2000153800.gpr	67	Male
**H-02**	Healthy Control	Control_H-02_2000153953.gpr		
**H-03**	Healthy Control	Control_H-03_2000153943.gpr		
**H-19**	Healthy Control	Control_H-19_2000154008.gpr		
**H-23**	Healthy Control	Control_H-23_2000154009.gpr		
**H-25**	Healthy Control	Control_H-25_2000153952.gpr		
**H-35**	Healthy Control	Control_H-35_2000153942.gpr		
**H-41**	Healthy Control	Control_H-41_2000154026.gpr		
**H-58**	Healthy Control	Control_H-58_2000153935.gpr		
**H-59**	Healthy Control	Control_H-59_2000154016.gpr		
**HC-25**	Healthy Control	Control_HC-25_2000153932.gpr		
**HV-56**	Healthy Control	Control_HV-56_2000155735.gpr		
**HV-59**	Healthy Control	Control_HV-59_2000155740.gpr		
**HV-64**	Healthy Control	Control_HV-64_2000144456.gpr		
**HV-70**	Healthy Control	Control_HV-70_2000144457.gpr		
**HV-71**	Healthy Control	Control_HV-71_2000144458.gpr		

Table 3S: Shortlisted proteins. List of proteins with adjusted p-value<0.05 and logFC >0.5 or <-0.5 for the healthy control and Grade IV samples.

Block	Row	Column	ID	Name	logFC	AveExpr	t	P.Value	adj.P.Val	B
41	5	11	BC025985.1	IGHG4	-1.75	9.37	-8.97	6.26E-16	1.13E-11	25.26
27	12	25	NM_001005465.1	OR10G3	-1.36	9.79	-7.08	3.89E-11	1.76E-07	14.86
27	13	15	BC065370.1	C20orf112	-1.30	9.41	-6.18	4.87E-09	5.86E-06	10.33
27	13	1	NM_024928.3	OBFC1	-1.27	10.02	-5.32	3.33E-07	7.42E-05	6.38
25	11	23	BC041037.1	IGHM	0.98	8.13	3.94	0.000119	0.00366	0.94
36	4	17	NM_003099.3	SNX1	0.84	9.47	4.86	2.70E-06	0.000292	4.43
27	14	23	NM_018584.4	CAMK2N1	-0.80	8.93	-2.84	0.005077	0.045225	-2.44
44	7	13	NM_001157.2	ANXA11	0.78	9.92	5.37	2.67E-07	6.52E-05	6.58
27	22	17	NM_001033515.1	LOC389833	-0.74	8.16	-5.78	3.69E-08	1.71E-05	8.43
27	21	1	BC037876.1	C17orf57	-0.74	8.64	-2.89	0.004319	0.040515	-2.29
43	1	27	NM_006857.1	RY1	0.74	8.94	5.60	8.58E-08	2.98E-05	7.64
27	2	15	NM_014372.3	RNF11	-0.72	8.67	-3.52	0.000565	0.010522	-0.48
27	15	29	NM_198086.1	JUB	-0.71	8.68	-3.36	0.000969	0.014872	-0.96
27	6	11	NM_024745.2	SHCBP1	-0.71	9.11	-3.05	0.002626	0.029	-1.86
38	19	7	NM_000689.3	ALDH1A1	-0.70	9.50	-5.49	1.51E-07	4.52E-05	7.11
40	7	13	BC015738.1	ZFYVE19	0.70	8.79	5.98	1.37E-08	9.57E-06	9.36

35	15	13	NM_001025266.1	LOC285382	0.69	8.41	3.27	0.001321	0.01842	-1.24
3	3	21	NM_032328.1	EFCAB2	0.69	9.22	6.05	9.16E-09	7.20E-06	9.73
27	14	19	NM_002767.2	PRPSAP2	-0.68	8.43	-3.30	0.001177	0.017153	-1.14
20	10	3	BC000870.1	TIPIN	0.68	8.87	6.10	7.15E-09	6.46E-06	9.97
3	3	11	NM_001001394.2	HCG3	0.68	8.86	5.55	1.10E-07	3.68E-05	7.41
39	1	31	BC013009.2	ZMYM3	0.67	10.13	3.49	0.00062	0.011082	-0.56
26	19	17	NM_018379.3	FAM63A	-0.67	8.50	-5.43	1.95E-07	5.41E-05	6.87
12	1	15	NM_001827.1	CKS2	0.67	8.11	6.57	6.21E-10	1.40E-06	12.26
41	18	15	BC000846.2	CIB1	-0.65	10.15	-3.58	0.000449	0.008851	-0.27
33	2	17	BC018747.1	IGHG1	0.65	10.08	3.11	0.002217	0.025956	-1.70
20	22	23	BC075842.1	IGHG1	0.64	10.39	4.98	1.61E-06	0.000202	4.91
8	16	11	NM_139204.1	EPS8L1	0.64	8.19	3.72	0.000275	0.006395	0.17
48	1	19	NM_178815.3	ARL5B	0.63	8.27	3.96	0.00011	0.003466	1.01
27	15	7	XM_290842.4	LRFN1	-0.63	8.19	-3.88	0.000151	0.004295	0.72
38	10	23	BC013186.1	RCSD1	-0.63	8.01	-4.25	3.52E-05	0.001621	2.05
11	1	19	NM_031966.2	CCNB1	0.62	8.61	6.13	6.24E-09	6.46E-06	10.09
32	6	27	NM_032347.1	ZNF397	0.62	9.82	3.63	0.00038	0.007969	-0.12
27	20	11	NM_021810.3	CDH26	-0.61	8.44	-3.84	0.000176	0.00477	0.58
38	18	5	NM_031304.2	DOHH	-0.60	9.92	-5.81	3.08E-08	1.63E-05	8.60
27	21	31	NM_001003892.1	DUPD1	-0.59	8.24	-3.0	0.002491	0.028025	-1.81

							7			
42	15	31	BC067254.1	COASY	-0.58	9.29	-6.21	4.17E-09	5.74E-06	10.47
27	18	31	NM_006541.1	TXNL2	-0.58	8.21	-3.62	0.000386	0.008067	-0.14
27	14	17	NM_148910.2	TIRAP	-0.57	8.30	-3.40	0.000848	0.013593	-0.85
46	23	31	BC000446	CLP1	-0.57	9.51	-4.85	2.84E-06	0.000304	4.38
16	2	1	NM_007198.2	PROSC	0.57	8.28	7.02	5.56E-11	2.01E-07	14.53
19	9	19	ENST00000435033		0.57	9.04	7.68	1.30E-12	1.17E-08	18.07
36	1	15	BC013992.1	MAPK3	0.56	9.01	4.58	9.18E-06	0.000691	3.29
36	7	13	BC010450.1	PRCC	0.55	8.56	5.26	4.50E-07	8.61E-05	6.09
23	16	5	NM_144495.2	PQBP1	-0.55	10.33	-3.23	0.001507	0.020135	-1.36
34	20	11	NM_022158.2	FN3K	-0.55	8.70	-6.92	9.67E-11	2.91E-07	14.01
41	14	19	NM_024826.1	ASAP	0.55	10.16	3.05	0.002637	0.029	-1.86
5	1	15	NM_001549.2	IFIT3	0.55	8.91	4.51	1.20E-05	0.000814	3.04
38	18	7	NM_021979.2	HSPA2	-0.54	9.12	-5.80	3.28E-08	1.65E-05	8.54
41	23	27	BC121798	EYA1	-0.53	9.58	-4.33	2.60E-05	0.001332	2.33
42	18	3	NM_024815.3	NUDT18	-0.53	9.20	-5.25	4.72E-07	8.89E-05	6.05
38	20	25	NM_001801.2	CDO1	-0.53	9.08	-5.95	1.52E-08	9.80E-06	9.26
31	6	23	NM_017924.2	C14orf119	0.53	9.15	4.32	2.67E-05	0.00135	2.31
23	2	11	BC016854.2	C11orf67	0.53	8.71	6.54	7.23E-10	1.45E-06	12.12

18	19	1	NM_006541.2	TXNL2	-0.52	9.69	-4.60	8.50E-06	0.000667	3.36
11	13	9	NM_000884.2	IMPDH2	0.51	8.62	4.67	6.30E-06	0.000547	3.64
15	3	27	BC004130.2	CALCOCO2	0.51	9.20	4.46	1.50E-05	0.000947	2.84
42	3	7	NM_000184.2	HBG2	0.51	9.18	3.93	0.000123	0.003725	0.91
11	1	9	NM_002867.2	RAB3B	0.51	8.92	4.66	6.45E-06	0.000552	3.62
39	3	15	NM_022453.2	RNF25	0.51	9.32	4.37	2.23E-05	0.00118	2.47
32	2	25	BC009561.1	C11orf74	0.51	9.44	5.19	6.20E-07	0.000105	5.79
4	2	13	NM_003123.3	SPN	0.51	7.84	3.08	0.002399	0.027327	-1.77
38	20	13	BC068569.1	ZHX3	-0.51	9.16	-3.89	0.000146	0.004214	0.75
46	19	9	NM_001008491.1	SEPT2	-0.51	9.82	-5.12	8.52E-07	0.000129	5.50
44	9	19	BC032665.1	EEF2K	0.51	8.78	4.57	9.69E-06	0.00071	3.24
20	16	3	ENST00000362035		0.51	8.15	5.70	5.28E-08	2.22E-05	8.09
34	23	9	NM_001155.3	ANXA6	-0.51	8.67	-5.33	3.12E-07	7.23E-05	6.43

Table 3S: Classifiers. List of classifiers and their corresponding statistics for the healthy control and Grade IV samples.

NF	Brier	BrierSE	CA	CASE	AUC	IS	Sensitivity	Specificity	PPV	NPV	FeatureList
1	0.24	0.04	0.84	0.05	0.88	0.35	0.91	0.67	0.86	0.77	NM_003099.3
2	0.21	0.03	0.84	0.04	0.95	0.42	0.91	0.67	0.86	0.77	NM_003099.3 NM_021810.3
3	0.24	0.06	0.82	0.06	0.94	0.39	0.91	0.60	0.84	0.75	NM_003099.3 NM_021810.3 BC068569.1
4	0.20	0.04	0.86	0.04	0.91	0.45	0.85	0.87	0.94	0.72	NM_003099.3 NM_021810.3 BC068569.1 BC009561.1
5	0.26	0.05	0.80	0.04	0.92	0.36	0.88	0.60	0.83	0.69	NM_003099.3 NM_021810.3 BC068569.1 BC009561.1 BC018747.1
6	0.21	0.04	0.82	0.05	0.94	0.40	0.88	0.67	0.86	0.71	NM_003099.3 NM_021810.3 BC068569.1 BC009561.1 BC018747.1 BC000846.2
7	0.23	0.05	0.80	0.07	0.88	0.40	0.91	0.53	0.82	0.73	NM_003099.3 NM_021810.3 BC068569.1 BC009561.1 BC018747.1 BC000846.2 BC121798
8	0.13	0.04	0.92	0.04	1.00	0.55	0.97	0.80	0.92	0.92	NM_003099.3 NM_021810.3 BC068569.1 BC009561.1 BC018747.1 BC000846.2 BC121798 BC037876.1
9	0.16	0.04	0.90	0.05	0.98	0.49	0.94	0.80	0.91	0.86	NM_003099.3 NM_021810.3 BC068569.1 BC009561.1 BC018747.1 BC000846.2 BC121798 BC037876.1 NM_144495.2
1	0	0	0	0	0	0	0.	0.	0	0	NM_003099.3 NM_021810.3 BC068569.1 BC009561.1

0	.16	.03	.88	.04	.98	.47	94	73	.89	.85	BC018747.1 BC000846.2 BC121798 BC037876.1 NM_144495.2 BC013186.1
11	0.15	0.03	0.92	0.04	0.98	0.50	0.97	0.80	0.92	0.92	NM_003099.3 NM_021810.3 BC068569.1 BC009561.1 BC018747.1 BC000846.2 BC121798 BC037876.1 NM_144495.2 BC013186.1 NM_003123.3
12	0.12	0.04	0.92	0.04	0.98	0.59	0.97	0.80	0.92	0.92	NM_003099.3 NM_021810.3 BC068569.1 BC009561.1 BC018747.1 BC000846.2 BC121798 BC037876.1 NM_144495.2 BC013186.1 NM_003123.3 NM_006541.1
13	0.12	0.03	0.88	0.04	0.98	0.60	0.94	0.73	0.89	0.85	NM_003099.3 NM_021810.3 BC068569.1 BC009561.1 BC018747.1 BC000846.2 BC121798 BC037876.1 NM_144495.2 BC013186.1 NM_003123.3 NM_006541.1 NM_000689.3
14	0.13	0.03	0.88	0.03	1.00	0.57	0.91	0.80	0.91	0.80	NM_003099.3 NM_021810.3 BC068569.1 BC009561.1 BC018747.1 BC000846.2 BC121798 BC037876.1 NM_144495.2 BC013186.1 NM_003123.3 NM_006541.1 NM_000689.3 NM_022158.2
15	0.17	0.03	0.84	0.04	0.97	0.50	0.88	0.73	0.88	0.73	NM_003099.3 NM_021810.3 BC068569.1 BC009561.1 BC018747.1 BC000846.2 BC121798 BC037876.1 NM_144495.2 BC013186.1 NM_003123.3 NM_006541.1 NM_000689.3 NM_022158.2 NM_018584.4
16	0.14	0.03	0.88	0.03	0.98	0.56	0.91	0.80	0.91	0.80	NM_003099.3 NM_021810.3 BC068569.1 BC009561.1 BC018747.1 BC000846.2 BC121798 BC037876.1 NM_144495.2 BC013186.1 NM_003123.3 NM_006541.1 NM_000689.3 NM_022158.2 NM_018584.4 NM_002867.2
17	0.12	0.03	0.92	0.03	0.98	0.58	0.97	0.80	0.92	0.92	NM_003099.3 NM_021810.3 BC068569.1 BC009561.1 BC018747.1 BC000846.2 BC121798 BC037876.1 NM_144495.2 BC013186.1 NM_003123.3 NM_006541.1 NM_000689.3 NM_022158.2 NM_018584.4 NM_002867.2 NM_006541.2
18	0.10	0.03	0.92	0.03	0.98	0.61	0.97	0.80	0.92	0.92	NM_003099.3 NM_021810.3 BC068569.1 BC009561.1 BC018747.1 BC000846.2 BC121798 BC037876.1 NM_144495.2 BC013186.1 NM_003123.3 NM_006541.1 NM_000689.3 NM_022158.2 NM_018584.4 NM_002867.2 NM_006541.2 NM_018379.3
19	0.11	0.03	0.90	0.03	1.00	0.62	0.97	0.73	0.89	0.92	NM_003099.3 NM_021810.3 BC068569.1 BC009561.1 BC018747.1 BC000846.2 BC121798 BC037876.1 NM_144495.2 BC013186.1 NM_003123.3 NM_006541.1 NM_000689.3 NM_022158.2 NM_018584.4 NM_002867.2 NM_006541.2 NM_018379.3 NM_001005465.1
20	0.11	0.03	0.92	0.03	1.00	0.60	0.97	0.80	0.92	0.92	NM_003099.3 NM_021810.3 BC068569.1 BC009561.1 BC018747.1 BC000846.2 BC121798 BC037876.1 NM_144495.2 BC013186.1 NM_003123.3 NM_006541.1 NM_000689.3 NM_022158.2 NM_018584.4 NM_002867.2 NM_006541.2 NM_018379.3 NM_001005465.1 NM_001801.2
2	0	0	0	0	1	0	0.	0.	0	0	NM_003099.3 NM_021810.3 BC068569.1 BC009561.1

1	.10	.03	.94	.03	.00	.61	97	87	.94	.93	BC018747.1 BC000846.2 BC121798 BC037876.1 NM_144495.2 BC013186.1 NM_003123.3 NM_006541.1 NM_000689.3 NM_022158.2 NM_018584.4 NM_002867.2 NM_006541.2 NM_018379.3 NM_001005465.1 NM_001801.2 NM_031966.2
22	0.12	0.03	0.90	0.04	1.00	0.59	0.97	0.73	0.89	0.92	NM_003099.3 NM_021810.3 BC068569.1 BC009561.1 BC018747.1 BC000846.2 BC121798 BC037876.1 NM_144495.2 BC013186.1 NM_003123.3 NM_006541.1 NM_000689.3 NM_022158.2 NM_018584.4 NM_002867.2 NM_006541.2 NM_018379.3 NM_001005465.1 NM_001801.2 NM_031966.2 NM_001025266.1
23	0.10	0.03	0.94	0.03	0.98	0.61	0.97	0.87	0.94	0.93	NM_003099.3 NM_021810.3 BC068569.1 BC009561.1 BC018747.1 BC000846.2 BC121798 BC037876.1 NM_144495.2 BC013186.1 NM_003123.3 NM_006541.1 NM_000689.3 NM_022158.2 NM_018584.4 NM_002867.2 NM_006541.2 NM_018379.3 NM_001005465.1 NM_001801.2 NM_031966.2 NM_001025266.1 NM_032328.1
24	0.09	0.02	0.94	0.03	0.98	0.63	0.97	0.87	0.94	0.93	NM_003099.3 NM_021810.3 BC068569.1 BC009561.1 BC018747.1 BC000846.2 BC121798 BC037876.1 NM_144495.2 BC013186.1 NM_003123.3 NM_006541.1 NM_000689.3 NM_022158.2 NM_018584.4 NM_002867.2 NM_006541.2 NM_018379.3 NM_001005465.1 NM_001801.2 NM_031966.2 NM_001025266.1 NM_032328.1 NM_178815.3
25	0.09	0.03	0.96	0.03	0.98	0.63	0.97	0.93	0.97	0.93	NM_003099.3 NM_021810.3 BC068569.1 BC009561.1 BC018747.1 BC000846.2 BC121798 BC037876.1 NM_144495.2 BC013186.1 NM_003123.3 NM_006541.1 NM_000689.3 NM_022158.2 NM_018584.4 NM_002867.2 NM_006541.2 NM_018379.3 NM_001005465.1 NM_001801.2 NM_031966.2 NM_001025266.1 NM_032328.1 NM_178815.3 BC000870.1
26	0.12	0.02	0.92	0.04	0.98	0.55	0.94	0.87	0.94	0.87	NM_003099.3 NM_021810.3 BC068569.1 BC009561.1 BC018747.1 BC000846.2 BC121798 BC037876.1 NM_144495.2 BC013186.1 NM_003123.3 NM_006541.1 NM_000689.3 NM_022158.2 NM_018584.4 NM_002867.2 NM_006541.2 NM_018379.3 NM_001005465.1 NM_001801.2 NM_031966.2 NM_001025266.1 NM_032328.1 NM_178815.3 BC000870.1 BC016854.2
27	0.09	0.01	0.96	0.03	1.00	0.61	0.94	1.00	1.00	0.88	NM_003099.3 NM_021810.3 BC068569.1 BC009561.1 BC018747.1 BC000846.2 BC121798 BC037876.1 NM_144495.2 BC013186.1 NM_003123.3 NM_006541.1 NM_000689.3 NM_022158.2 NM_018584.4 NM_002867.2 NM_006541.2 NM_018379.3 NM_001005465.1 NM_001801.2 NM_031966.2 NM_001025266.1 NM_032328.1 NM_178815.3 BC000870.1 BC016854.2 NM_001033515.1
28	0.08	0.01	0.96	0.03	1.00	0.62	0.97	0.93	0.97	0.93	NM_003099.3 NM_021810.3 BC068569.1 BC009561.1 BC018747.1 BC000846.2 BC121798 BC037876.1 NM_144495.2 BC013186.1 NM_003123.3 NM_006541.1 NM_000689.3 NM_022158.2 NM_018584.4 NM_002867.2 NM_006541.2 NM_018379.3 NM_001005465.1 NM_001801.2 NM_031966.2

											NM_001025266.1 NM_032328.1 NM_178815.3 BC000870.1 BC016854.2 NM_001033515.1 NM_000184.2
29	0.10	0.02	0.94	0.03	0.98	0.61	1.00	0.80	0.92	1.00	NM_003099.3 NM_021810.3 BC068569.1 BC009561.1 BC018747.1 BC000846.2 BC121798 BC037876.1 NM_144495.2 BC013186.1 NM_003123.3 NM_006541.1 NM_000689.3 NM_022158.2 NM_018584.4 NM_002867.2 NM_006541.2 NM_018379.3 NM_001005465.1 NM_001801.2 NM_031966.2 NM_001025266.1 NM_032328.1 NM_178815.3 BC000870.1 BC016854.2 NM_001033515.1 NM_000184.2 NM_001003892.1
30	0.10	0.02	0.94	0.03	1.00	0.60	0.97	0.87	0.94	0.93	NM_003099.3 NM_021810.3 BC068569.1 BC009561.1 BC018747.1 BC000846.2 BC121798 BC037876.1 NM_144495.2 BC013186.1 NM_003123.3 NM_006541.1 NM_000689.3 NM_022158.2 NM_018584.4 NM_002867.2 NM_006541.2 NM_018379.3 NM_001005465.1 NM_001801.2 NM_031966.2 NM_001025266.1 NM_032328.1 NM_178815.3 BC000870.1 BC016854.2 NM_001033515.1 NM_000184.2 NM_001003892.1 NM_007198.2

**Table 3S: Classifiers.** List of 10 classifier proteins for the healthy control and Grade IV samples.								
ID	Name	logFC	AveExpr	t	P.Value	adj.P.Val	B	Regulation
NM_003099.3	SNX1	0.84	9.47	4.86	2.70E-06	0.000292	4.43	Up
BC018747.1	IGHG1	0.65	10.08	3.11	0.002217	0.025956	-1.70	Up
BC009561.1	C11orf74	0.51	9.44	5.19	6.20E-07	0.000105	5.79	Up
BC068569.1	ZHX3	-0.51	9.16	-3.89	0.000146	0.004214	0.75	Down
BC121798	EYA1	-0.53	9.58	-4.33	2.60E-05	0.001332	2.33	Down
NM_144495.2	PQBP1	-0.55	10.33	-3.23	0.001507	0.020135	-1.36	Down
NM_021810.3	CDH26	-0.61	8.44	-3.84	0.000176	0.00477	0.58	Down
BC013186.1	RCSD1	-0.63	8.01	-4.25	3.52E-05	0.001621	2.05	Down
BC000846.2	CIB1	-0.65	10.15	-3.58	0.000449	0.008851	-0.27	Down
BC037876.1	C17orf57	-0.74	8.64	-2.89	0.004319	0.040515	-2.29	Down

**Table 4S: Experimental details.** The details of the SVZp and SVZn samples.				
**Sample ID**	**Sample Type**	**File name**	**Age**	**Sex**
**CF 11518**	SVZn	Grade_IV_CF_11518_2000153942.gpr	57	Female
**CF 19257**	SVZn	Grade_IV_CF_19257_2000155723.gpr	60	Male
**CF 27891**	SVZn	Grade_IV_CF_27891_2000153776.gpr	64	Male
**CH 12724**	SVZn	Grade_IV_CH_12724_2000153778.gpr	60	Male
**CH 18016**	SVZn	Grade_IV_CH_18016_2000153791.gpr	40	Male
**CH 5261**	SVZn	Grade_IV_CH_5261_2000155722.gpr	30	Male
**CH 8498**	SVZn	Grade_IV_CH_8498_2000153777.gpr	52	Male
**CJ 10048**	SVZn	Grade_IV_CJ_10048_2000155725.gpr	37	Male
**CJ 12441**	SVZn	Grade_IV_CJ_12441_2000153760.gpr	58	Male
**CJ 18704**	SVZn	Grade_IV_CJ_18704_2000153784.gpr	65	Male
**CJ 2041**	SVZn	Grade_IV_CJ_2041_2000154019.gpr	58	Male
**CJ 32705**	SVZn	Grade_IV_CJ_32705_2000153797.gpr	59	Male
**CJ 6254**	SVZn	Grade_IV_CJ_6254_2000153800.gpr	67	Male
**CF 11940**	SVZp	Grade_IV_CF_11940_2000153779.gpr	59	Male
**CF 12166**	SVZp	Grade_IV_CF_12166_2000153934.gpr	64	Male
**CF 7522**	SVZp	Grade_IV_CF_7522_2000154010.gpr	20	Female
**CF 7916**	SVZp	Grade_IV_CF_7916_2000154021.gpr	56	Male
**CH 14820**	SVZp	Grade_IV_CH_14820_2000153796.gpr	57	Male
**CH 18426**	SVZp	Grade_IV_CH_18426_2000153757.gpr	56	Male
**CH 21783**	SVZp	Grade_IV_CH_21783_2000153785.gpr	47	Male
**CH 22618**	SVZp	Grade_IV_CH_22618_2000153782.gpr	53	Male
**CH 28569**	SVZp	Grade_IV_CH_28569_2000153799.gpr	38	Female
**CH 29697**	SVZp	Grade_IV_CH_29697_2000153759.gpr	60	Male
**CH 29738**	SVZp	Grade_IV_CH_29738_2000153798.gpr	46	Male
**CH 30203**	SVZp	Grade_IV_CH_30203_2000153793.gpr	61	Female
**CH 3969**	SVZp	Grade_IV_CH_3969_2000153950.gpr	46	Female
**CH 4996**	SVZp	Grade_IV_CH_4996_2000153933.gpr	57	Male
**CH 6643**	SVZp	Grade_IV_CH_6643_2000153758.gpr	47	Male
**CH 9002**	SVZp	Grade_IV_CH_9002_2000153954.gpr	69	Male
**CJ 11291**	SVZp	Grade_IV_CJ_11291_2000154025.gpr	51	Female
**CJ 13655**	SVZp	Grade_IV_CJ_13655_2000153783.gpr	68	Female
**CJ 1519**	SVZp	Grade_IV_CJ_1519_2000153951.gpr	55	Female
**CJ 22776**	SVZp	Grade_IV_CJ_22776_2000153756.gpr	61	Male
**CJ 30014**	SVZp	Grade_IV_CJ_30014_2000153805.gpr	64	Male

Table 4S: Shortlisted proteins. List of proteins with adjusted p-value<0.05 and logFC >0.5 or <-0.5 for the SVZp and SVZn samples.

Block	Row	Column	ID	Name	logFC	AveExpr	t	P.Value	adj.P.Val	B
44	14	21	NM_006403.2	NEDD9	-0.52	8.85	-5.13	8.02E-07	0.003623	5.43
22	19	29	NM_018290.2	PGM2	0.55	9.42	4.75	4.49E-06	0.007368	3.90
2	23	3	NM_001938.2	DR1	0.85	11.25	4.69	5.67E-06	0.008156	3.69
8	8	11	BC035031.2	GABPA	-0.65	7.41	-4.63	7.25E-06	0.008736	3.47
27	12	1	NM_032448.1	FAM120B	-0.53	7.95	-4.61	8.04E-06	0.009008	3.38
5	7	9	NM_020313.2	CIAPIN1	-0.70	7.48	-4.53	1.14E-05	0.009129	3.07
27	17	11	NM_001007275.1	DUSP13	-0.67	7.65	-4.52	1.15E-05	0.009129	3.06
10	8	9	NM_178471.1	GPR119	-0.51	7.38	-4.50	1.28E-05	0.009129	2.97
16	7	19	BC033711.1	ATXN3	-0.67	7.45	-4.35	2.36E-05	0.011504	2.43
29	7	29	NM_000409.2	GUCA1A	-0.51	7.41	-4.31	2.80E-05	0.012491	2.27
48	7	13	NM_000666.1	ACY1	-0.51	7.42	-4.30	2.93E-05	0.012588	2.23
6	7	19	BC017810.1	TMOD4	-0.65	7.48	-4.04	8.04E-05	0.026405	1.34
43	8	1	NM_152740.2	HIBADH	1.09	10.74	4.03	8.61E-05	0.027251	1.28
3	4	1	BC000267.1	GPBP1	0.92	10.53	4.01	9.05E-05	0.027251	1.24
30	15	27	XM_379060.1		0.53	9.29	3.95	0.00011461	0.031813	1.03
16	12	7	NM_024482.1	GMEB1	-0.52	8.38	-3.92	0.00012774	0.033444	0.94

21	21	21	BC094687.1	EEF1A1	0.75	8.42	3.82	0.00018923	0.044396	0.59
44	12	3	BC019883.1	CLEC2D	-0.52	7.43	-3.80	0.00019996	0.045719	0.55

Table 4S: Classifiers. List of classifiers and their corresponding statistics for the SVZp and SVZn samples.

NF	Brier	BrierSE	CA	CASE	AUC	IS	Sensitivity	Specificity	PPV	NPV	FeatureList
1	0.373511	0.035588	0.766667	0.055277	0.9	0.203306	0.538462	0.904762	0.777778	0.76	NM_001938.2
2	0.283782	0.040356	0.8	0.045134	0.875	0.357254	0.692308	0.857143	0.75	0.818182	NM_001938.2 NM_018290.2
3	0.195261	0.054879	0.866667	0.058531	0.95	0.521466	0.692308	0.952381	0.9	0.833333	NM_001938.2 NM_018290.2 NM_032448.1
4	0.174262	0.051162	0.866667	0.058531	0.95	0.576657	0.692308	0.952381	0.9	0.833333	NM_001938.2 NM_018290.2 NM_032448.1 NM_006403.2
5	0.154755	0.037581	0.925	0.038188	0.975	0.586843	0.846154	0.952381	0.916667	0.909091	NM_001938.2 NM_018290.2 NM_032448.1 NM_006403.2 NM_024482.1
6	0.1	0.0	0.8	0.0	0.	0.5	0.7	0.9	0.8	0.8	NM_001938.2 NM_018290.2 NM_032448.1 NM_006403.2 NM_024482.1 BC000267.1

	63792	50467	66667	58531	95	78927	69231	04762	33333	63636	
7	0.1656554	0.0451331	0.9	0.0552777	0.925	0.5747719	0.7692331	0.9523881	0.9090991	0.8695665	NM_001938.2 NM_018290.2 NM_032448.1 NM_006403.2 NM_024482.1 BC000267.1 NM_152740.2
8	0.1827758	0.0464778	0.8583333	0.0609221	0.925	0.5259928	0.7692331	0.9047762	0.8333333	0.8636363	NM_001938.2 NM_018290.2 NM_032448.1 NM_006403.2 NM_024482.1 BC000267.1 NM_152740.2 BC017810.1
9	0.1562661	0.0356777	0.925	0.0381888	0.975	0.5408006	0.8461554	0.9523881	0.9166667	0.9090991	NM_001938.2 NM_018290.2 NM_032448.1 NM_006403.2 NM_024482.1 BC000267.1 NM_152740.2 BC017810.1 BC094687.1
10	0.1752265	0.0393884	0.8916667	0.0448776	0.975	0.5161622	0.7692331	0.9523881	0.9090991	0.8695665	NM_001938.2 NM_018290.2 NM_032448.1 NM_006403.2 NM_024482.1 BC000267.1 NM_152740.2 BC017810.1 BC094687.1 XM_379060.1
11	0.1711111	0.0385733	0.95	0.0333333	0.95	0.5218777	0.9230777	0.9523881	0.9230777	0.9523881	NM_001938.2 NM_018290.2 NM_032448.1 NM_006403.2 NM_024482.1 BC000267.1 NM_152740.2 BC017810.1 BC094687.1 XM_379060.1 NM_020313.2
12	0.1913443	0.0405548	0.9166667	0.0430033	0.975	0.50732	0.8461554	0.9523881	0.9166667	0.9090991	NM_001938.2 NM_018290.2 NM_032448.1 NM_006403.2 NM_024482.1 BC000267.1 NM_152740.2 BC017810.1 BC094687.1 XM_379060.1 NM_020313.2 BC019883.1

13	0.172282	0.038669	0.916667	0.043033	0.975	0.513029	0.923077	0.904762	0.857143	0.95	NM_001938.2 NM_018290.2 NM_032448.1 NM_006403.2 NM_024482.1 BC000267.1 NM_152740.2 BC017810.1 BC094687.1 XM_379060.1 NM_020313.2 BC019883.1 BC035031.2
14	0.180885	0.040585	0.883333	0.048432	0.975	0.512617	0.846154	0.904762	0.846154	0.904762	NM_001938.2 NM_018290.2 NM_032448.1 NM_006403.2 NM_024482.1 BC000267.1 NM_152740.2 BC017810.1 BC094687.1 XM_379060.1 NM_020313.2 BC019883.1 BC035031.2 NM_000409.2
15	0.215493	0.047816	0.883333	0.048432	0.95	0.474121	0.846154	0.904762	0.846154	0.904762	NM_001938.2 NM_018290.2 NM_032448.1 NM_006403.2 NM_024482.1 BC000267.1 NM_152740.2 BC017810.1 BC094687.1 XM_379060.1 NM_020313.2 BC019883.1 BC035031.2 NM_000409.2 NM_001007275.1
16	0.19312	0.041666	0.916667	0.043033	0.975	0.490698	0.923077	0.904762	0.857143	0.95	NM_001938.2 NM_018290.2 NM_032448.1 NM_006403.2 NM_024482.1 BC000267.1 NM_152740.2 BC017810.1 BC094687.1 XM_379060.1 NM_020313.2 BC019883.1 BC035031.2 NM_000409.2 NM_001007275.1 BC033711.1
17	0.173153	0.036882	0.916667	0.043033	0.975	0.526884	0.846154	0.952381	0.916667	0.909091	NM_001938.2 NM_018290.2 NM_032448.1 NM_006403.2 NM_024482.1 BC000267.1 NM_152740.2 BC017810.1 BC094687.1 XM_379060.1 NM_020313.2 BC019883.1 BC035031.2 NM_000409.2 NM_001007275.1 BC033711.1 NM_000666.1
18	0.17972	0.039064	0.95	0.033333	0.975	0.511815	0.923077	0.952381	0.923077	0.952381	NM_001938.2 NM_018290.2 NM_032448.1 NM_006403.2 NM_024482.1 BC000267.1 NM_152740.2 BC017810.1 BC094687.1 XM_379060.1 NM_020313.2 BC019883.1 BC035031.2 NM_000409.2 NM_001007275.1 BC033711.1 NM_000666.1 NM_178471.1
19	0.1797	0.0390	0.95	0.0333	0.975	0.5118	0.9230	0.9523	0.9230	0.9523	NM_001938.2 NM_018290.2 NM_032448.1 NM_006403.2 NM_024482.1 BC000267.1 NM_152740.2 BC017810.1 BC094687.1 XM_379060.1 NM_020313.2 BC019883.1 BC035031.2 NM_000409.2 NM_001007275.1 BC033711.1 NM_000666.1

	2	64		33		15	77	81	77	81	NM_178471.1
20	0.17972	0.039064	0.95	0.033333	0.975	0.511815	0.923077	0.952381	0.923077	0.952381	NM_001938.2 NM_018290.2 NM_032448.1 NM_006403.2 NM_024482.1 BC000267.1 NM_152740.2 BC017810.1 BC094687.1 XM_379060.1 NM_020313.2 BC019883.1 BC035031.2 NM_000409.2 NM_001007275.1 BC033711.1 NM_000666.1 NM_178471.1
21	0.17972	0.039064	0.95	0.033333	0.975	0.511815	0.923077	0.952381	0.923077	0.952381	NM_001938.2 NM_018290.2 NM_032448.1 NM_006403.2 NM_024482.1 BC000267.1 NM_152740.2 BC017810.1 BC094687.1 XM_379060.1 NM_020313.2 BC019883.1 BC035031.2 NM_000409.2 NM_001007275.1 BC033711.1 NM_000666.1 NM_178471.1
22	0.17972	0.039064	0.95	0.033333	0.975	0.511815	0.923077	0.952381	0.923077	0.952381	NM_001938.2 NM_018290.2 NM_032448.1 NM_006403.2 NM_024482.1 BC000267.1 NM_152740.2 BC017810.1 BC094687.1 XM_379060.1 NM_020313.2 BC019883.1 BC035031.2 NM_000409.2 NM_001007275.1 BC033711.1 NM_000666.1 NM_178471.1
23	0.17972	0.039064	0.95	0.033333	0.975	0.511815	0.923077	0.952381	0.923077	0.952381	NM_001938.2 NM_018290.2 NM_032448.1 NM_006403.2 NM_024482.1 BC000267.1 NM_152740.2 BC017810.1 BC094687.1 XM_379060.1 NM_020313.2 BC019883.1 BC035031.2 NM_000409.2 NM_001007275.1 BC033711.1 NM_000666.1 NM_178471.1
24	0.17972	0.039064	0.95	0.033333	0.975	0.511815	0.923077	0.952381	0.923077	0.952381	NM_001938.2 NM_018290.2 NM_032448.1 NM_006403.2 NM_024482.1 BC000267.1 NM_152740.2 BC017810.1 BC094687.1 XM_379060.1 NM_020313.2 BC019883.1 BC035031.2 NM_000409.2 NM_001007275.1 BC033711.1 NM_000666.1 NM_178471.1
25	0.17972	0.039064	0.95	0.033333	0.975	0.511815	0.923077	0.952381	0.923077	0.952381	NM_001938.2 NM_018290.2 NM_032448.1 NM_006403.2 NM_024482.1 BC000267.1 NM_152740.2 BC017810.1 BC094687.1 XM_379060.1 NM_020313.2 BC019883.1 BC035031.2 NM_000409.2 NM_001007275.1 BC033711.1 NM_000666.1 NM_178471.1
26	0.17	0.03	0.95	0.03	0.9	0.51	0.92	0.95	0.92	0.95	NM_001938.2 NM_018290.2 NM_032448.1 NM_006403.2 NM_024482.1 BC000267.1 NM_152740.2 BC017810.1 BC094687.1 XM_379060.1 NM_020313.2

	972	9064		3333	75	1815	3077	2381	3077	2381	BC019883.1 BC035031.2 NM_000409.2 NM_001007275.1 BC033711.1 NM_000666.1 NM_178471.1
27	0.17972	0.039064	0.95	0.033333	0.975	0.511815	0.923077	0.952381	0.923077	0.952381	NM_001938.2 NM_018290.2 NM_032448.1 NM_006403.2 NM_024482.1 BC000267.1 NM_152740.2 BC017810.1 BC094687.1 XM_379060.1 NM_020313.2 BC019883.1 BC035031.2 NM_000409.2 NM_001007275.1 BC033711.1 NM_000666.1 NM_178471.1
28	0.17972	0.039064	0.95	0.033333	0.975	0.511815	0.923077	0.952381	0.923077	0.952381	NM_001938.2 NM_018290.2 NM_032448.1 NM_006403.2 NM_024482.1 BC000267.1 NM_152740.2 BC017810.1 BC094687.1 XM_379060.1 NM_020313.2 BC019883.1 BC035031.2 NM_000409.2 NM_001007275.1 BC033711.1 NM_000666.1 NM_178471.1
29	0.17972	0.039064	0.95	0.033333	0.975	0.511815	0.923077	0.952381	0.923077	0.952381	NM_001938.2 NM_018290.2 NM_032448.1 NM_006403.2 NM_024482.1 BC000267.1 NM_152740.2 BC017810.1 BC094687.1 XM_379060.1 NM_020313.2 BC019883.1 BC035031.2 NM_000409.2 NM_001007275.1 BC033711.1 NM_000666.1 NM_178471.1
30	0.17972	0.039064	0.95	0.033333	0.975	0.511815	0.923077	0.952381	0.923077	0.952381	NM_001938.2 NM_018290.2 NM_032448.1 NM_006403.2 NM_024482.1 BC000267.1 NM_152740.2 BC017810.1 BC094687.1 XM_379060.1 NM_020313.2 BC019883.1 BC035031.2 NM_000409.2 NM_001007275.1 BC033711.1 NM_000666.1 NM_178471.1

**Table 4S: Classifiers.** List of 10 classifier proteins for the SVZp and SVZn samples.								
ID	Name	logFC	AveExpr	t	P.Value	adj.P.Val	B	Regulation with respect to SVZp
NM_006403.2	NEDD9	-0.5231	8.850901	-5.13073	8.02E-07	0.003623	5.427753	Down
NM_018290.2	PGM2	0.553421	9.424367	4.745176	4.49E-06	0.007368	3.895634	Up
NM_001938.2	DR1	0.84551	11.25256	4.691203	5.67E-06	0.008156	3.688151	Up
NM_032448.1	FAM120B	-0.53461	7.94502	-4.60948	8.04E-06	0.009008	3.377387	Down
BC017810.1	TMOD4	-0.64824	7.482631	-4.04427	8.04E-05	0.026405	1.344158	Down
NM_152740.2	HIBADH	1.091449	10.74323	4.026714	8.61E-05	0.027251	1.284368	Up
BC000267.1	GPBP1	0.917842	10.53046	4.013646	9.05E-05	0.027251	1.240006	Up
XM_379060.1	LOC339685	0.531831	9.292159	3.952141	0.000115	0.031813	1.03279	Up
NM_024482.1	GMEB1	-0.51622	8.383436	-3.92365	0.000128	0.033444	0.937675	Down
BC094687.1	EEF1A1	0.753808	8.419964	3.819124	0.000189	0.044396	0.593586	Up

Table 5S: Experimental details. The patient details with the IDHp, WT and 1p and 19q status for different grades of glioma.

FileName	Patient ID	Condition	IDH status	1p	19q
GradeII	CF 8551	Grade2	NA	NA	NA
GradeII	CF 10729	Grade2	WT	NA	NA
GradeII	CF 20468	Grade2	WT	not deleted	not deleted
GradeII	CF 28254	Grade2	NA	NA	NA
GradeII	CF 29538	Grade2	IDHp	NA	NA
GradeII	CH 12323	Grade2	NA	not deleted	not deleted
GradeII	CH 13717	Grade2	NA	not deleted	not deleted
GradeII	CH 27095	Grade2	NA	NA	NA
GradeII	CH 32225	Grade2	NA	not deleted	not deleted
GradeII	CJ 863	Grade2	NA	NA	NA
GradeII	CJ 4825	Grade2	IDHp	not deleted	uninterpretable
GradeII	CJ 7132	Grade2	IDHp	NA	NA
GradeII	CJ 10242	Grade2	WT	not deleted	not deleted
GradeII	CJ 16084	Grade2	NA	NA	NA
GradeII	CJ 17734	Grade2	NA	NA	NA
GradeII	CJ 33350	Grade2	IDHp	deleted	deleted
GradeII	CK 40	Grade2	WT	not deleted	not deleted
GradeIII	CF 12839	Grade3	IDHp	NA	NA
GradeIII	CF 16216	Grade3	IDHp	NA	NA
GradeIII	CF 22483	Grade3	NA	not deleted	not deleted
GradeIII	CF 25539	Grade3	WT	not deleted	not deleted
GradeIII	CF 29446	Grade3	WT	NA	NA
GradeIII	CH 9010	Grade3	NA	NA	NA
GradeIII	CH 9043	Grade3	WT	NA	NA
GradeIII	CH 17244	Grade3	IDHp	not deleted	not deleted
GradeIII	CJ 1176	Grade3	NA	NA	NA
GradeIII	CJ 7833	Grade3	IDHp	deleted	uninterpretable
GradeIII	CJ 9761	Grade3	IDHp	not deleted	not deleted
GradeIII	CJ 14116	Grade3	IDHp	not deleted	not deleted
GradeIII	CJ 16912	Grade3	NA	not deleted	not deleted
GradeIII	CF 22807	Grade3	NA	NA	NA
GradeIII	CF 6980	Grade3	NA	NA	NA
GradeIII	CH 15130	Grade3	IDHp	NA	NA
GradeIII	CH 15138	Grade3	NA	NA	NA
GradeIII	CJ 20721	Grade3	NA	NA	NA
GradeIV	CF 7522	Grade4SVZp	NA	NA	NA
GradeIV	CF 7916	Grade4SVZp	IDHp	NA	NA
GradeIV	CF 11518	Grade4SVZn	NA	NA	NA
GradeIV	CF 11940	Grade4SVZp	NA	NA	NA

GradeIV	CF 12166	Grade4SVZp	IDHp	NA	NA
GradeIV	CF 19257	Grade4SVZn	NA	NA	NA
GradeIV	CF 27891	Grade4SVZn	NA	NA	NA
GradeIV	CH 3969	Grade4SVZp	WT	NA	NA
GradeIV	CH 4996	Grade4SVZp	NA	NA	NA
GradeIV	CH 5261	Grade4SVZn	NA	NA	NA
GradeIV	CH 6643	Grade4SVZp	NA	NA	NA
GradeIV	CH 8498	Grade4SVZn	NA	not deleted	not deleted
GradeIV	CH 9002	Grade4SVZp	NA	NA	NA
GradeIV	CH 12724	Grade4SVZn	NA	NA	NA
GradeIV	CH 14820	Grade4SVZp	NA	NA	NA
GradeIV	CH 18016	Grade4SVZn	WT	NA	NA
GradeIV	CH 18426	Grade4SVZp	NA	NA	NA
GradeIV	CH 21783	Grade4SVZp	NA	NA	NA
GradeIV	CH 22618	Grade4SVZp	NA	NA	NA
GradeIV	CH 28569	Grade4SVZp	NA	NA	NA
GradeIV	CH 29697	Grade4SVZp	NA	NA	NA
GradeIV	CH 29738	Grade4SVZp	NA	NA	NA
GradeIV	CH 30203	Grade4SVZp	NA	NA	NA
GradeIV	CJ 1519	Grade4SVZp	NA	NA	NA
GradeIV	CJ 2041	Grade4SVZn	NA	NA	NA
GradeIV	CJ 6254	Grade4SVZn	NA	NA	NA
GradeIV	CJ 10048	Grade4SVZn	NA	NA	NA
GradeIV	CJ 11291	Grade4SVZp	NA	NA	NA
GradeIV	CJ 12441	Grade4SVZn	WT	NA	NA
GradeIV	CJ 13655	Grade4SVZp	NA	NA	NA
GradeIV	CJ 18704	Grade4SVZn	NA	NA	NA
GradeIV	CJ 22776	Grade4SVZp	IDHp	NA	NA
GradeIV	CJ 30014	Grade4SVZp	WT	NA	NA
GradeIV	CJ 32705	Grade4SVZn	NA	NA	NA

Table 5S: Shortlisted proteins (Grade II: IDHp vs WT). List of proteins with p-value<0.05 and logFC >0.5 or <-0.5 for Grade II: IDHp vs WT samples.

Block	Row	Column	ID	Name	logFC	AveExpr	t	P.Value	adj.P.Val	B	abslogFC
41	18	15	BC000846.2	CIB1	1.338358	10.63468	10.37573	2.39E-08	0.000432	8.278787	1.338358
16	14	3	NM_017735.3	TTC27	-0.99635	10.56595	-8.8276	2.05E-07	0.001851	6.679011	0.996354
17	6	7	BC034483.1	HSPA1L	0.583475	10.30696	7.776351	1.03E-06	0.005928	5.400163	0.583475
24	16	7	ENST00000389266		-1.11001	9.715674	-7.54565	1.49E-06	0.005928	5.096484	1.110015
24	17	29	BC026060.2	CAMKK2	-0.60396	9.656103	-7.48834	1.64E-06	0.005928	5.019704	0.603956
11	6	31	NM_016401.2	C11orf73	-0.7173	9.651959	-6.89773	4.42E-06	0.010616	4.196858	0.717295
37	19	15	NM_001888.2	CRYM	0.547707	9.170732	6.861579	4.70E-06	0.010616	4.144592	0.547707
36	24	17	NM_007026.1	N.D.	2.056038	9.521931	6.627491	7.06E-06	0.013357	3.800801	2.056038
37	19	13	NM_005861.2	STUB1	0.712943	9.58449	6.601235	7.39E-06	0.013357	3.761659	0.712943
24	13	17	NM_000519.3	HBD	-0.54912	11.25092	-6.34832	1.16E-05	0.017435	3.378595	0.549118
16	16	5	NM_017863.2	CXorf48	-0.78121	10.04777	-5.99034	2.22E-05	0.021231	2.817781	0.781214
23	21	27	BC089418.1	IGLV3-25	-0.64768	9.815037	-5.90079	2.62E-05	0.021231	2.674105	0.64768
13	23	9	NM_014440.1	IL1F6	0.547456	10.21958	5.853503	2.86E-05	0.021231	2.597692	0.547456
5	17	1	NM_130897.1	DYNLRB2	0.551471	8.905261	5.738918	3.54E-05	0.02452	2.410984	0.551471

33	4	15	BC001394.2	SERPINB6	0.517334	8.670526	5.643051	4.24E-05	0.026773	2.25311	0.517334
9	22	15	BC000967.2	NUDCD1	0.553045	9.580231	5.635919	4.30E-05	0.026773	2.241304	0.553045
19	13	3	BC064512.1	UBR2	-0.61139	9.859295	-5.60749	4.54E-05	0.027309	2.194173	0.611391
30	21	27	NM_001106.3	ACVR2B	0.866547	8.113489	5.506809	5.49E-05	0.031998	2.026167	0.866547
41	22	1	BC075862.1	CAPN1	0.582924	8.843983	5.404513	6.68E-05	0.036555	1.853808	0.582924
8	12	15	BC002508.2	ASMTL	-0.65339	10.3223	-5.34555	7.48E-05	0.037532	1.753713	0.653389
29	24	25	NM_003405.3	YWHAH	0.548356	9.285129	5.326232	7.76E-05	0.037651	1.720797	0.548356
8	13	9	NM_013330.3	NME7	-0.50224	9.435105	-5.24761	9.04E-05	0.040819	1.586246	0.502244

**Table 5S: Shortlisted proteins (Grade IV: IDHp vs WT).** List of proteins with p-value<0.05 and logFC >0.5 or <-0.5 for Grade II: IDHp vs WT samples.											
Block	Row	Column	ID	Name	logFC	AveExpr	t	P.Value	adj.P.Val	B	abslogFC
22	13	3	NM_152734.3	C6orf89	0.636265	9.988923	7.544568	5.46E-07	0.009868	3.97153	0.636265

**Table 6S: Enrichment analysis report.** Enrichment by Pathway Maps (healthy control vs Grade II)								
Enrichment by Pathway Maps					GRADE II_INPUT_genelist			
**#**	**Maps**	**Total**	**pValue**	**Min FDR**	**p-value**	**FDR**	**In Data**	**Network Objects from Active Data**
1	Development_Beta-adrenergic receptors transactivation of EGFR	37	4.345E-04	8.636E-03	4.345E-04	8.636E-03	2	CD44, MYLK1
2	Cytoskeleton remodeling_Hyaluronic acid/ CD44 signaling pathways	43	5.876E-04	8.636E-03	5.876E-04	8.636E-03	2	CD44, MLCK
3	Cell adhesion_Integrin-mediated cell adhesion and migration	48	7.324E-04	8.636E-03	7.324E-04	8.636E-03	2	MYLK1, MLCK
4	Chemotaxis_Inhibitory action of lipoxins on IL-8- and Leukotriene B4-induced neutrophil migration	51	8.268E-04	8.636E-03	8.268E-04	8.636E-03	2	MYLK1, MLCK
5	Airway smooth muscle contraction in asthma	56	9.964E-04	8.636E-03	9.964E-04	8.636E-03	2	MLCK, Telokin
6	Impaired inhibitory action of lipoxins on neutrophil migration in CF	56	9.964E-04	8.636E-03	9.964E-04	8.636E-03	2	MYLK1, MLCK
7	Muscle contraction_GPCRs in the regulation of smooth muscle tone	83	2.176E-03	1.616E-02	2.176E-03	1.616E-02	2	MLCK, Telokin
8	Cytoskeleton remodeling_Cytoskeleton remodeling	102	3.267E-03	2.123E-02	3.267E-03	2.123E-02	2	MYLK1, MLCK
9	Cytoskeleton remodeling_TGF, WNT and cytoskeletal remodeling	111	3.856E-03	2.228E-02	3.856E-03	2.228E-02	2	MYLK1, MLCK

Table 6S: Enrichment analysis report. Enrichment by Pathway Maps (healthy control vs Grade III)

Enrichment by Pathway Maps					GRADE III_INPUT_4_genelist			
**#**	**Maps**	**Total**	**pValue**	**Min FDR**	**p-value**	**FDR**	**In Data**	**Network Objects from Active Data**
1	Development_Flt3 signaling	44	5.139E-07	1.978E-04	5.139E-07	1.978E-04	6	CRK, STAT5A, p38 MAPK, ERK1/2, CrkL, GRB2
2	Cytoskeleton remodeling_TGF, WNT and cytoskeletal remodeling	111	8.832E-07	1.978E-04	8.832E-07	1.978E-04	8	CRK, p38 MAPK, ERK1 (MAPK3), ERK1/2, MYLK1, GRB2, 4E-BP1, MLCK
3	Signal transduction_PTMs in BAFF-induced signaling	51	1.262E-06	1.978E-04	1.262E-06	1.978E-04	6	p38 MAPK, ERK1 (MAPK3), Bim, Ubiquitin, ERK1/2, 4E-BP1
4	Development_EPO-induced Jak-STAT pathway	35	3.852E-06	4.526E-04	3.852E-06	4.526E-04	5	STAT5A, ERK1 (MAPK3), STAT5, CrkL, GRB2
5	Cytoskeleton remodeling_Cytoskeleton remodeling	102	6.217E-06	5.844E-04	6.217E-06	5.844E-04	7	CRK, p38 MAPK, ERK1/2, MYLK1, GRB2, 4E-BP1, MLCK
6	Development_NOTCH-induced EMT	19	7.649E-06	5.992E-04	7.649E-06	5.992E-04	4	NOTCH1 (NICD), RBP-J kappa (CBF1), NOTCH1 receptor, NOTCH1 (NEXT)
7	Development_Notch Signaling Pathway	43	1.094E-05	7.347E-04	1.094E-05	7.347E-04	5	NOTCH1 (NICD), RBP-J kappa (CBF1), NOTCH1 receptor, NOTCH1 (NEXT), NOTCH1 precursor
8	Cell adhesion_Integrin-mediated cell adhesion and migration	48	1.895E-05	1.113E-03	1.895E-05	1.113E-03	5	CRK, ERK1/2, MYLK1, GRB2, MLCK
9	Development_FGFR signaling pathway	54	3.390E-05	1.770E-03	3.390E-05	1.770E-03	5	CRK, p38 MAPK, Ubiquitin, ERK1/2, GRB2
10	Immune response_IL-15 signaling	64	7.747	3.191	7.747	3.191	5	p38 MAPK, ERK1 (MAPK3), ERK1/2, GRB2, 4E-BP1

			E-05	E-03	E-05	E-03		
11	Chemotaxis_CXCR4 signaling pathway	34	8.463E-05	3.191E-03	8.463E-05	3.191E-03	4	CRK, ERK1 (MAPK3), CrkL, GRB2
12	Development_NOTCH1-mediated pathway for NF-KB activity modulation	34	8.463E-05	3.191E-03	8.463E-05	3.191E-03	4	NOTCH1 (NICD), RBP-J kappa (CBF1), NOTCH1 receptor, NOTCH1 (NEXT)
13	Role of Tissue factor in cancer independent of coagulation protease signaling	35	9.505E-05	3.191E-03	9.505E-05	3.191E-03	4	p38 MAPK, ERK1 (MAPK3), ERK1/2, GRB2
14	Immune response_Oncostatin M signaling via MAPK in mouse cells	35	9.505E-05	3.191E-03	9.505E-05	3.191E-03	4	p38 MAPK, ERK1 (MAPK3), ERK1/2, GRB2
15	DNA damage_Nucleotide excision repair	36	1.064E-04	3.280E-03	1.064E-04	3.280E-03	4	Ubiquitin, XPF, RAD23A, RPA4
16	Immune response_Oncostatin M signaling via MAPK in human cells	37	1.187E-04	3.280E-03	1.187E-04	3.280E-03	4	p38 MAPK, ERK1 (MAPK3), ERK1/2, GRB2
17	Development_Beta-adrenergic receptors transactivation of EGFR	37	1.187E-04	3.280E-03	1.187E-04	3.280E-03	4	ERK1 (MAPK3), ERK1/2, MYLK1, GRB2
18	Immune response_IL-7 signaling in B lymphocytes	43	2.149E-04	5.611E-03	2.149E-04	5.611E-03	4	STAT5A, STAT5, ERK1/2, GRB2
19	Immune response_TNF-R2 signaling pathways	45	2.567E-04	6.351E-03	2.567E-04	6.351E-03	4	p38 MAPK, Bim, ERK1/2, BMF
20	Immune response_IL-5 signalling	46	2.797E-04	6.498E-03	2.797E-04	6.498E-03	4	STAT5, ERK1/2, CrkL, GRB2
21	Development_TGF-beta-dependent induction of EMT via MAPK	47	3.042E-04	6.498E-03	3.042E-04	6.498E-03	4	p38 MAPK, ERK1/2, GRB2, Occludin

22	Development_HGF signaling pathway	47	3.042E-04	6.498E-03	3.042E-04	6.498E-03	4	CRK, ERK1/2, CrkL, GRB2
23	Immune response_IL-2 activation and signaling pathway	49	3.576E-04	7.003E-03	3.576E-04	7.003E-03	4	STAT5A, STAT5, ERK1/2, GRB2
24	Development_G-CSF signaling	49	3.576E-04	7.003E-03	3.576E-04	7.003E-03	4	p38 MAPK, STAT5, ERK1/2, GRB2
25	Development_GM-CSF signaling	50	3.867E-04	7.269E-03	3.867E-04	7.269E-03	4	STAT5A, STAT5, ERK1/2, GRB2
26	Development_Oligodendrocyte differentiation from adult stem cells	51	4.174E-04	7.545E-03	4.174E-04	7.545E-03	4	NOTCH1 (NICD), p38 MAPK, NOTCH1 receptor, ERK1/2
27	Translation_Regulation of EIF4F activity	53	4.840E-04	8.147E-03	4.840E-04	8.147E-03	4	p38 MAPK, ERK1/2, GRB2, 4E-BP1
28	Immune response_IL-12 signaling pathway	23	5.157E-04	8.147E-03	5.157E-04	8.147E-03	3	STAT5A, STAT5, Ubiquitin
29	Immune response_HSP60 and HSP70/ TLR signaling pathway	54	5.200E-04	8.147E-03	5.200E-04	8.147E-03	4	TIRAP (Mal), p38 MAPK, Ubiquitin, ERK1/2
30	Neurophysiological process_Dynein-dynactin motor complex in axonal transport in neurons	54	5.200E-04	8.147E-03	5.200E-04	8.147E-03	4	Ubiquitin, Kinesin light chain, ERK1/2, DYNLL
31	Aberrant B-Raf signaling in melanoma progression	55	5.579E-04	8.458E-03	5.579E-04	8.458E-03	4	NOTCH1 (NICD), Bim, ERK1/2, BMF
32	Immune response_TLR2 and TLR4 signaling pathways	57	6.395E-04	9.108E-03	6.395E-04	9.108E-03	4	TIRAP (Mal), p38 MAPK, Ubiquitin, ERK1/2
33	Development_Cytokine-mediated regulation of megakaryopoiesis	57	6.395E-	9.108E-	6.395E-	9.108E-	4	STAT5, ERK1/2, GRB2, 4E-BP1

			04	03	04	03		
34	Effect of H. pylori infection on gastric epithelial cell proliferation	58	6.833E-04	9.175E-03	6.833E-04	9.175E-03	4	CRK, ERK1/2, CrkL, GRB2
35	Development_Prolactin receptor signaling	58	6.833E-04	9.175E-03	6.833E-04	9.175E-03	4	STAT5A, STAT5, ERK1/2, GRB2
36	IGF family signaling in colorectal cancer	60	7.772E-04	1.015E-02	7.772E-04	1.015E-02	4	ERK1 (MAPK3), ERK1/2, GRB2, 4E-BP1
37	Development_c-Kit ligand signaling pathway during hemopoiesis	61	8.274E-04	1.051E-02	8.274E-04	1.051E-02	4	STAT5, ERK1/2, CrkL, GRB2
38	Immune response_CD137 signaling in immune cell	29	1.033E-03	1.277E-02	1.033E-03	1.277E-02	3	p38 MAPK, Bim, ERK1/2
39	Immune response_Mast cell proliferation, differentiation and survival	30	1.142E-03	1.376E-02	1.142E-03	1.376E-02	3	STAT5, ERK1/2, GRB2
40	Immune response_IL-3 activation and signaling pathway	31	1.258E-03	1.442E-02	1.258E-03	1.442E-02	3	STAT5, ERK1/2, GRB2
41	Immune response_ETV3 affect on CSF1-promoted macrophage differentiation	31	1.258E-03	1.442E-02	1.258E-03	1.442E-02	3	p38 MAPK, ERK1/2, GRB2
42	Signal transduction_ERK1/2 signaling pathway	32	1.381E-03	1.510E-02	1.381E-03	1.510E-02	3	ERK1 (MAPK3), ERK1/2, GRB2
43	Signal transduction_PTMs in IL-17-induced CIKS-dependent MAPK signaling pathways	32	1.381E-03	1.510E-02	1.381E-03	1.510E-02	3	p38 MAPK, Ubiquitin, ERK1/2
44	Development_EGFR signaling pathway	71	1.463E-03	1.562E-02	1.463E-03	1.562E-02	4	p38 MAPK, ERK1 (MAPK3), ERK1/2, GRB2

45	Development_Epigenetic and transcriptional regulation of oligodendrocyte precursor cell differentiation and myelination	34	1.650E-03	1.616E-02	1.650E-03	1.616E-02	3	NOTCH1 (NICD), RBP-J kappa (CBF1), NOTCH1 receptor
46	Development_S1P1 receptor signaling via beta-arrestin	34	1.650E-03	1.616E-02	1.650E-03	1.616E-02	3	ERK1 (MAPK3), ERK1/2, GRB2
47	Apoptosis and survival_Cytoplasmic/mitochondrial transport of proapoptotic proteins Bid, Bmf and Bim	34	1.650E-03	1.616E-02	1.650E-03	1.616E-02	3	DLC1 (Dynein LC8a), Bim, BMF
48	Immune response_IL-22 signaling pathway	34	1.650E-03	1.616E-02	1.650E-03	1.616E-02	3	p38 MAPK, STAT5, ERK1/2
49	Development_Angiopoietin - Tie2 signaling	35	1.796E-03	1.722E-02	1.796E-03	1.722E-02	3	CRK, STAT5, GRB2
50	Immune response_HMGB1/TLR signaling pathway	36	1.949E-03	1.796E-02	1.949E-03	1.796E-02	3	TIRAP (Mal), Ubiquitin, IL1RN
51	Immune response_IL-9 signaling pathway	36	1.949E-03	1.796E-02	1.949E-03	1.796E-02	3	STAT5, ERK1/2, GRB2
52	Neurophysiological process_NMDA-dependent postsynaptic long-term potentiation in CA1 hippocampal neurons	80	2.272E-03	1.880E-02	2.272E-03	1.880E-02	4	ERK1 (MAPK3), ERK1/2, GRB2, 4E-BP1
53	Immune response_Role of integrins in NK cells cytotoxicity	38	2.280E-03	1.880E-02	2.280E-03	1.880E-02	3	p38 MAPK, ERK1 (MAPK3), GRB2
54	Development_SDF-1 signaling in hematopoietic stem cell homing	38	2.280E-03	1.880E-02	2.280E-03	1.880E-02	3	CRK, ERK1/2, CrkL
55	Role of tumor microenvironment in plexiform neurofibroma formation in neurofibromatosis type 1	38	2.280E-03	1.880E-02	2.280E-03	1.880E-02	3	p38 MAPK, ERK1/2, GRB2
56	Immune response_Human NKG2D signaling	38	2.280E-	1.880E-	2.280E-	1.880E-	3	STAT5, ERK1/2, GRB2

			03	02	03	02		
57	Signal transduction_Soluble CXCL16 signaling	38	2.280E-03	1.880E-02	2.280E-03	1.880E-02	3	p38 MAPK, ERK1/2, 4E-BP1
58	Regulation of degradation of deltaF508-CFTR in CF	39	2.458E-03	1.926E-02	2.458E-03	1.926E-02	3	SAE1, Ubiquitin, Sti1
59	Immune response_TSLP signalling	39	2.458E-03	1.926E-02	2.458E-03	1.926E-02	3	p38 MAPK, STAT5, ERK1/2
60	Transcription_Receptor-mediated HIF regulation	39	2.458E-03	1.926E-02	2.458E-03	1.926E-02	3	ERK1/2, GRB2, 4E-BP1
61	G-protein signaling_Rap1B regulation pathway	11	2.519E-03	1.941E-02	2.519E-03	1.941E-02	2	GRB2, Rap1GDS1
62	Development_VEGF signaling via VEGFR2 - generic cascades	84	2.715E-03	2.058E-02	2.715E-03	2.058E-02	4	p38 MAPK, ERK1 (MAPK3), ERK1/2, GRB2
63	Development_Neurotrophin family signaling	41	2.839E-03	2.118E-02	2.839E-03	2.118E-02	3	ERK1/2, CrkL, GRB2
64	Translation_Insulin regulation of translation	42	3.043E-03	2.167E-02	3.043E-03	2.167E-02	3	ERK1/2, GRB2, 4E-BP1
65	Immune response_Murine NKG2D signaling	42	3.043E-03	2.167E-02	3.043E-03	2.167E-02	3	STAT5, ERK1/2, GRB2
66	Signal transduction_JNK pathway	42	3.043E-03	2.167E-02	3.043E-03	2.167E-02	3	CRK, GRB2, GCK(MAP4K2)
67	Immune response_HTR2A-induced activation of cPLA2	43	3.255E-03	2.250E-02	3.255E-03	2.250E-02	3	p38 MAPK, ERK1/2, GRB2

68	Cytoskeleton remodeling_Hyaluronic acid/ CD44 signaling pathways	43	3.255E-03	2.250E-02	3.255E-03	2.250E-02	3	ERK1/2, GRB2, MLCK
69	Immune response_IL-4 signaling pathway	44	3.476E-03	2.334E-02	3.476E-03	2.334E-02	3	ERK1/2, GRB2, IGHG1
70	Influence of smoking on activation of EGFR signaling in lung cancer cells	44	3.476E-03	2.334E-02	3.476E-03	2.334E-02	3	ERK1 (MAPK3), ERK1/2, GRB2
71	Development_EPO-induced MAPK pathway	45	3.705E-03	2.386E-02	3.705E-03	2.386E-02	3	ERK1/2, CrkL, GRB2
72	Development_Ligand-independent activation of ESR1 and ESR2	45	3.705E-03	2.386E-02	3.705E-03	2.386E-02	3	ERK1 (MAPK3), ERK1/2, GRB2
73	Development_Thrombopoietin-regulated cell processes	45	3.705E-03	2.386E-02	3.705E-03	2.386E-02	3	ERK1/2, CrkL, GRB2
74	Development_Endothelin-1/EDNRA transactivation of EGFR	46	3.944E-03	2.505E-02	3.944E-03	2.505E-02	3	STAT5, ERK1/2, GRB2
75	Regulation of lipid metabolism_Insulin signaling:generic cascades	47	4.193E-03	2.627E-02	4.193E-03	2.627E-02	3	ERK1/2, GRB2, 4E-BP1
76	Signal transduction_PTMs in IL-12 signaling pathway	48	4.450E-03	2.716E-02	4.450E-03	2.716E-02	3	STAT5A, STAT5, Ubiquitin
77	Immune response_TLR5, TLR7, TLR8 and TLR9 signaling pathways	48	4.450E-03	2.716E-02	4.450E-03	2.716E-02	3	p38 MAPK, Ubiquitin, ERK1/2
78	Transcription_CREB pathway	49	4.717E-03	2.771E-02	4.717E-03	2.771E-02	3	p38 MAPK, ERK1/2, GRB2
79	Role and regulation of Prostaglandin E2 in gastric cancer	49	4.717E-	2.771E-	4.717E-	2.771E-	3	NOTCH1 receptor, ERK1/2, GRB2

			03	02	03	02		
80	Tissue Factor signaling in cancer via PAR1 and PAR2	49	4.717E-03	2.771E-02	4.717E-03	2.771E-02	3	STAT5A, ERK1/2, GRB2
81	Development_A2B receptor: action via G-protein alpha s	50	4.993E-03	2.897E-02	4.993E-03	2.897E-02	3	p38 MAPK, ERK1/2, GRB2
82	Chemotaxis_Inhibitory action of lipoxins on IL-8- and Leukotriene B4-induced neutrophil migration	51	5.280E-03	3.026E-02	5.280E-03	3.026E-02	3	ERK1/2, MYLK1, MLCK
83	Development_IGF-1 receptor signaling	52	5.575E-03	3.119E-02	5.575E-03	3.119E-02	3	ERK1/2, GRB2, 4E-BP1
84	Development_FGF-family signaling	52	5.575E-03	3.119E-02	5.575E-03	3.119E-02	3	p38 MAPK, ERK1 (MAPK3), GRB2
85	Development_A1 receptor signaling	53	5.881E-03	3.141E-02	5.881E-03	3.141E-02	3	p38 MAPK, ERK1/2, GRB2
86	Immune response_T cell receptor signaling pathway	53	5.881E-03	3.141E-02	5.881E-03	3.141E-02	3	Ubiquitin, ERK1/2, GRB2
87	Immune response_HMGB1/RAGE signaling pathway	53	5.881E-03	3.141E-02	5.881E-03	3.141E-02	3	p38 MAPK, ERK1/2, IL1RN
88	Immune response_T regulatory cell-mediated modulation of effector T cell and NK cell functions	53	5.881E-03	3.141E-02	5.881E-03	3.141E-02	3	NOTCH1 (NICD), RBP-J kappa (CBF1), NOTCH1 receptor
89	DNA damage_Role of SUMO in p53 regulation	17	6.064E-03	3.200E-02	6.064E-03	3.200E-02	2	SAE1, Ubiquitin
90	Immune response_Role of DAP12 receptors in NK cells	54	6.196E-03	3.200E-02	6.196E-03	3.200E-02	3	ERK1/2, CrkL, GRB2

91	Immune response _IFN gamma signaling pathway	54	6.196E-03	3.200E-02	6.196E-03	3.200E-02	3	p38 MAPK, ERK1/2, CrkL
92	Immune response_Fc epsilon RI pathway	55	6.521E-03	3.332E-02	6.521E-03	3.332E-02	3	p38 MAPK, ERK1/2, GRB2
93	Chemotaxis_CCL2-induced chemotaxis	56	6.857E-03	3.357E-02	6.857E-03	3.357E-02	3	p38 MAPK, ERK1/2, GRB2
94	Development_Keratinocyte differentiation	56	6.857E-03	3.357E-02	6.857E-03	3.357E-02	3	NOTCH1 (NICD), RBP-J kappa (CBF1), ERK1/2
95	Impaired inhibitory action of lipoxins on neutrophil migration in CF	56	6.857E-03	3.357E-02	6.857E-03	3.357E-02	3	ERK1/2, MYLK1, MLCK
96	Regulation of lipid metabolism_Insulin regulation of glycogen metabolism	56	6.857E-03	3.357E-02	6.857E-03	3.357E-02	3	ERK1/2, GRB2, HXK4
97	Cytoskeleton remodeling_FAK signaling	57	7.202E-03	3.490E-02	7.202E-03	3.490E-02	3	CRK, ERK1/2, GRB2
98	Immune response_TREM1 signaling pathway	59	7.923E-03	3.800E-02	7.923E-03	3.800E-02	3	Ubiquitin, ERK1/2, GRB2
99	Development_TGF-beta-induction of EMT via ROS	20	8.359E-03	3.929E-02	8.359E-03	3.929E-02	2	p38 MAPK, GRB2
100	Translation_IL-2 regulation of translation	20	8.359E-03	3.929E-02	8.359E-03	3.929E-02	2	GRB2, 4E-BP1

Table 6S: Enrichment analysis report. Enrichment by Pathway Maps (healthy control vs Grade IV)

Enrichment by Pathway Maps					GRADE IV_INPUT(1)_genelist			
**#**	**Maps**	**Total**	**pValue**	**Min FDR**	**p-value**	**FDR**	**In Data**	**Network Objects from Active Data**
1	Immune response_IL-4 signaling pathway	44	1.280 E-04	3.435 E-02	1.280 E-04	3.435 E-02	3	IGHG4, IGHG1, ERK1/2
2	Immune response_HSP60 and HSP70/ TLR signaling pathway	54	2.363 E-04	3.435 E-02	2.363 E-04	3.435 E-02	3	TIRAP (Mal), HSP70, ERK1/2
3	Transport_RAB3 regulation pathway	14	4.447 E-04	3.435 E-02	4.447 E-04	3.435 E-02	2	Rab-3B, Rab-3
4	Cell cycle_Nucleocytoplasmic transport of CDK/Cyclins	14	4.447 E-04	3.435 E-02	4.447 E-04	3.435 E-02	2	Cyclin B1, ERK1 (MAPK3)

Table 6S: Enrichment analysis report. Enrichment by Pathway Maps (healthy control vs SVZp)

Enrichment by Pathway Maps					SVZp Limma Input_genelist			
**#**	**Maps**	**Total**	**pValue**	**Min FDR**	**p-value**	**FDR**	**In Data**	**Network Objects from Active Data**
1	Development_Notch Signaling Pathway	43	6.729E-06	2.214E-03	6.729E-06	2.214E-03	4	NOTCH1 (NICD), NOTCH1 (NEXT), NOTCH1 precursor, NOTCH1 receptor
2	Development_NOTCH-induced EMT	19	2.121E-05	3.488E-03	2.121E-05	3.488E-03	3	NOTCH1 (NICD), NOTCH1 (NEXT), NOTCH1 receptor
3	Development_NOTCH1-mediated pathway for NF-KB activity modulation	34	1.272E-04	1.395E-02	1.272E-04	1.395E-02	3	NOTCH1 (NICD), NOTCH1 (NEXT), NOTCH1 receptor
4	Immune response_IL-4 signaling pathway	44	2.760E-04	2.270E-02	2.760E-04	2.270E-02	3	IGHG4, IGHG1, ERK1/2
5	Development_Oligodendrocyte differentiation from adult stem cells	51	4.281E-04	2.817E-02	4.281E-04	2.817E-02	3	NOTCH1 (NICD), NOTCH1 receptor, ERK1/2
6	Transport_RAB3 regulation pathway	14	7.394E-04	3.475E-02	7.394E-04	3.475E-02	2	Rab-3B, Rab-3
7	Cell cycle_Nucleocytoplasmic transport of CDK/Cyclins	14	7.394E-04	3.475E-02	7.394E-04	3.475E-02	2	Cyclin B1, ERK1 (MAPK3)

No significant pathways enriched in healthy control vs SVZp comparison by Pathway maps.

**Table 6S: Enrichment analysis report.** Enrichment by Pathway Maps (SVZp vs SVZn)								
Enrichment by Pathway Maps					SVZp VS SVZn Limma List_genelist			
**#**	**Maps**	**Total**	**pValue**	**Min FDR**	**p-value**	**FDR**	**In Data**	**Network Objects from Active Data**
1	Regulation of degradation of wtCFTR	20	1.298E-02	3.504E-02	1.298E-02	3.504E-02	1	MJD (ataxin-3)
2	Proteolysis_Putative ubiquitin pathway	23	1.492E-02	3.504E-02	1.492E-02	3.504E-02	1	MJD (ataxin-3)
3	Neurophysiological process _Visual perception	30	1.942E-02	3.504E-02	1.942E-02	3.504E-02	1	GCAP1
4	LRRK2 in neurons in Parkinson's disease	33	2.135E-02	3.504E-02	2.135E-02	3.504E-02	1	eEF1A
5	Glycogen metabolism	37	2.391E-02	3.504E-02	2.391E-02	3.504E-02	1	PGMU
6	Immune response_Generation of memory CD4+ T cells	37	2.391E-02	3.504E-02	2.391E-02	3.504E-02	1	GABP alpha
7	Regulation of degradation of deltaF508-CFTR in CF	39	2.519E-02	3.504E-02	2.519E-02	3.504E-02	1	MJD (ataxin-3)
8	Propionate metabolism p.1	39	2.519E-02	3.504E-02	2.519E-02	3.504E-02	1	3HIDH
9	Pentose phosphate pathway/ Rodent version	43	2.774E-02	3.504E-02	2.774E-02	3.504E-02	1	PGMU
10	Pentose phosphate pathway	44	2.838E-02	3.504E-02	2.838E-02	3.504E-02	1	PGMU
11	Glycolysis and gluconeogenesis p. 1	46	2.965E-02	3.504E-02	2.965E-02	3.504E-02	1	PGMU
12	Galactose metabolism	59	3.790E-02	4.105E-02	3.790E-02	4.105E-02	1	PGMU
13	Galactose metabolism/ Rodent version	64	4.105E-02	4.105E-02	4.105E-02	4.105E-02	1	PGMU

**Table 7S: Enrichment analysis report.** Enrichment by Metabolic Networks (healthy control vs Grade II)								
Enrichment by Metabolic Networks					GRADE II_INPUT_genelist			
**#**	**Networks**	**Total**	**pValue**	**Min FDR**	**p-value**	**FDR**	**In Data**	**Network Objects from Active Data**
1	Phosphatidic acid pathway	13	4.118E-03	1.235E-02	4.118E-03	1.235E-02	1	SNX1
2	Phosphatidylethanolamine pathway	91	2.865E-02	2.896E-02	2.865E-02	2.896E-02	1	CD44
3	Phosphatidylinositol-4,5-diphosphate pathway	92	2.896E-02	2.896E-02	2.896E-02	2.896E-02	1	SNX1

No significant pathways enriched in healthy control vs Grade III comparison by Metabolic Networks.

No significant pathways enriched in healthy control vs Grade IV comparison by Metabolic Networks.

**Table 7S: Enrichment analysis report.** Enrichment by Metabolic Networks (healthy control vs SVZp)								
Enrichment by Metabolic Networks					SVZp Limma Input_genelist			
**#**	**Networks**	**Total**	**pValue**	**Min FDR**	**p-value**	**FDR**	**In Data**	**Network Objects from Active Data**
1	Phosphatidylethanolamine pathway	91	1.837E-05	4.409E-04	1.837E-05	4.409E-04	4	Tissue kallikreins, Kallikrein 1, Annexin XI, Kallikrein 3 (PSA)
2	2-arachidonoyl-glycerol_3-phosphocholine_pathway	140	2.032E-03	2.439E-02	2.032E-03	2.439E-02	3	Tissue kallikreins, OATP-C, ERK1/2

No significant pathways enriched in healthy control vs SVZn comparison by Metabolic Networks.

No significant pathways enriched in SVZp vs SVZn comparison by Metabolic Networks.

**Table 8S: Enrichment analysis report.** Enrichment by Process Networks (healthy control vs Grade II)								
Enrichment by Process Networks					GRADE II_INPUT_genelist			
**#**	**Networks**	**Total**	**pValue**	**Min FDR**	**p-value**	**FDR**	**In Data**	**Network Objects from Active Data**
1	Cell adhesion_Cell-matrix interactions	211	2.071 E-04	5.178 E-03	2.071 E-04	5.178 E-03	4	CD44 (EXT), CD44, CD44 soluble, CD44 (ICD)
2	Cytoskeleton_Actin filaments	176	2.095 E-03	2.618 E-02	2.095 E-03	2.618 E-02	3	CD44, MYLK1, MLCK

Table 8S: Enrichment analysis report. Enrichment by Process Networks (healthy control vs Grade III)

Enrichment by Process Networks					GRADE III_INPUT_4(1)_genelist			
**#**	**Networks**	**Total**	**pValue**	**Min FDR**	**p-value**	**FDR**	**In Data**	**Network Objects from Active Data**
1	Development_Blood vessel morphogenesis	228	8.403E-06	8.058E-04	8.403E-06	8.058E-04	12	CRK, NOTCH1 (NICD), ERK1 (MAPK3), STAT5, RBP-J kappa (CBF1), NOTCH1 receptor, ERK1/2, Guanylate cyclase A (NPR1), GRB2, Notch, NOTCH1 (NEXT), NOTCH1 precursor
2	Signal transduction_NOTCH signaling	236	1.194E-05	8.058E-04	1.194E-05	8.058E-04	12	NOTCH1 (NICD), p38 MAPK, ERK1 (MAPK3), RBP-J kappa (CBF1), NOTCH1 receptor, p38gamma (MAPK12), Ubiquitin, ERK1/2, GRB2, NOTCH1 (NEXT), NOTCH1 precursor, 4E-BP1
3	Development_Hemopoiesis, Erythropoietin pathway	136	2.054E-05	9.242E-04	2.054E-05	9.242E-04	9	STAT5A, p38 MAPK, ERK1 (MAPK3), Bim, STAT5, p38gamma (MAPK12), ERK1/2, CrkL, GRB2
4	Development_EMT_ Regulation of epithelial-to-mesenchymal transition	225	4.108E-05	1.386E-03	4.108E-05	1.386E-03	11	CRK, NOTCH1 (NICD), p38 MAPK, RBP-J kappa (CBF1), NOTCH1 receptor, ERK1/2, CrkL, GRB2, NOTCH1 (NEXT), 4E-BP1, Occludin
5	Cytoskeleton_Actin filaments	176	1.543E-04	4.167E-03	1.543E-04	4.167E-03	9	CRK, ERK1 (MAPK3), LASP1, Twinfilin, ERK1/2, MYLK1, GRB2, Tropomodulin, MLCK
6	Cell adhesion_Glycoconjugates	162	4.631E-04	1.042E-02	4.631E-04	1.042E-02	8	Reticulon 4, Siglec-8, ERK1 (MAPK3), ERK1/2, Siglec-E, SAP, Galectin-13, SIGLEC5
7	Immune response_IL-5 signalling	38	8.437E-04	1.512E-02	8.437E-04	1.512E-02	4	STAT5, ERK1/2, CrkL, GRB2
8	Apoptosis_Anti-Apoptosis mediated by external signals via MAPK and JAK/STAT	179	8.962E-	1.512E-	8.962E-	1.512E-	8	CRK, STAT5A, p38 MAPK, Bim, STAT5, ERK1/2, CrkL, GRB2

			04	02	04	02		
9	Inflammation_IL-2 signaling	104	1.104E-03	1.655E-02	1.104E-03	1.655E-02	6	STAT5A, p38 MAPK, ERK1 (MAPK3), STAT5, ERK1/2, GRB2
10	Cell adhesion_Amyloid proteins	195	1.557E-03	2.102E-02	1.557E-03	2.102E-02	8	Reticulon 4, Siglec-8, NOTCH1 (NICD), ERK1 (MAPK3), NOTCH1 receptor, ERK1/2, GRB2, Notch
11	DNA damage_Checkpoint	124	2.715E-03	3.332E-02	2.715E-03	3.332E-02	6	RAD1, p38 MAPK, ERK1 (MAPK3), p38gamma (MAPK12), Ubiquitin, ERK1/2
12	Translation_Regulation of initiation	127	3.060E-03	3.442E-02	3.060E-03	3.442E-02	6	p38 MAPK, ERK1 (MAPK3), p38gamma (MAPK12), ERK1/2, GRB2, 4E-BP1
13	Immune response_TCR signaling	174	3.437E-03	3.569E-02	3.437E-03	3.569E-02	7	p38 MAPK, ERK1 (MAPK3), Bim, p38gamma (MAPK12), Ubiquitin, ERK1/2, GRB2

**Table 8S: Enrichment analysis report.** Enrichment by Process Networks (healthy control vs Grade IV)								
Enrichment by Process Networks					GRADE IV_INPUT_genelist			
**#**	**Networks**	**Total**	**pValue**	**Min FDR**	**p-value**	**FDR**	**In Data**	**Network Objects from Active Data**
1	Protein folding_Response to unfolded proteins	69	8.643E-05	8.789E-03	8.643E-05	8.789E-03	4	HSPA1B, HSP70, HSPA1A, HSPA2
2	Reproduction_Male sex differentiation	246	1.598E-04	8.789E-03	1.598E-04	8.789E-03	6	Olfactory receptor, HSP70, ERK1 (MAPK3), HSPA1A, HSPA2, ERK1/2
3	Inflammation_IL-4 signaling	115	6.201E-04	1.552E-02	6.201E-04	1.552E-02	4	IGHG4, IGHG1, ERK1 (MAPK3), ERK1/2
4	Cell cycle_G2-M	206	6.291E-04	1.552E-02	6.291E-04	1.552E-02	5	Cyclin B1, ERK1 (MAPK3), CKS2, Cyclin B, ERK1/2
5	Protein folding_Folding in normal condition	119	7.056E-04	1.552E-02	7.056E-04	1.552E-02	4	HSPA1B, HSP70, HSPA1A, HSPA2

Table 8S: Enrichment analysis report. Enrichment by Process Networks (healthy control vs SVZp)

Enrichment by Process Networks					SVZp Limma Input_genelist			
**#**	**Networks**	**Total**	**pValue**	**Min FDR**	**p-value**	**FDR**	**In Data**	**Network Objects from Active Data**
1	Development_Blood vessel morphogenesis	228	6.013E-08	6.674E-06	6.013E-08	6.674E-06	10	Tissue kallikreins, Kallikrein 1, NOTCH1 (NICD), Notch, NOTCH1 (NEXT), ERK1 (MAPK3), NOTCH1 precursor, NOTCH1 receptor, Kallikrein 3 (PSA), ERK1/2
2	Development_Neurogenesis in general	192	2.634E-05	1.462E-03	2.634E-05	1.462E-03	7	LHX1, NOTCH1 (NICD), Notch, NOTCH1 (NEXT), NOTCH1 precursor, NOTCH1 receptor, ERK1/2
3	Cell cycle_G2-M	206	4.144E-05	1.533E-03	4.144E-05	1.533E-03	7	Cyclin B1, ERK1 (MAPK3), 14-3-3 zeta/delta, CKS2, Cyclin B, ERK1/2, 14-3-3
4	Development_Hedgehog signaling	254	1.558E-04	4.324E-03	1.558E-04	4.324E-03	7	Cyclin B1, LHX1, NOTCH1 (NICD), NOTCH1 (NEXT), ERK1 (MAPK3), NOTCH1 precursor, NOTCH1 receptor
5	DNA damage_Checkpoint	124	2.698E-04	5.989E-03	2.698E-04	5.989E-03	5	ERK1 (MAPK3), 14-3-3 zeta/delta, Cyclin B, ERK1/2, 14-3-3
6	Inflammation_TREM1 signaling	145	5.558E-04	1.028E-02	5.558E-04	1.028E-02	5	TIRAP (Mal), ERK1 (MAPK3), 14-3-3 zeta/delta, ERK1/2, 14-3-3
7	Signal transduction_NOTCH signaling	236	7.529E-04	1.194E-02	7.529E-04	1.194E-02	6	NOTCH1 (NICD), NOTCH1 (NEXT), ERK1 (MAPK3), NOTCH1 precursor, NOTCH1 receptor, ERK1/2
8	Cytoskeleton_Regulation of cytoskeleton rearrangement	183	1.587E-03	2.202E-02	1.587E-03	2.202E-02	5	ERK1 (MAPK3), CD43, 14-3-3 zeta/delta, ERK1/2, 14-3-3
9	Inflammation_IL-4 signaling	115	2.034E-03	2.326E-02	2.034E-03	2.326E-02	4	IGHG4, IGHG1, ERK1 (MAPK3), ERK1/2
10	Cell adhesion_Amyloid proteins	19	2.100	2.326	2.100	2.326	5	NOTCH1 (NICD), Notch, ERK1 (MAPK3), NOTCH1 receptor, ERK1/2

		5	E-03	E-02	E-03	E-02		
11	Inflammation_IL-6 signaling	119	2.305E-03	2.326E-02	2.305E-03	2.326E-02	4	ERK1 (MAPK3), 14-3-3 zeta/delta, ERK1/2, 14-3-3
12	Reproduction_Progesterone signaling	214	3.149E-03	2.913E-02	3.149E-03	2.913E-02	5	Cyclin B1, ERK1 (MAPK3), NOTCH1 precursor, SULT1E1, CKS2

**Table 8S: Enrichment analysis report.** Enrichment by Process Networks (healthy control vs SVZn)								
Enrichment by Process Networks					SVZn Limma Input_genelist			
#	Networks	Total	pValue	Min FDR	p-value	FDR	In Data	Network Objects from Active Data
1	Protein folding_Response to unfolded proteins	69	6.564 E-05	6.171 E-03	6.564 E-05	6.171 E-03	5	HSPA1B, HSP70, HSPA2, HSPA1A, HSC70
2	Protein folding_Folding in normal condition	119	8.447 E-04	3.970 E-02	8.447 E-04	3.970 E-02	5	HSPA1B, HSP70, HSPA2, HSPA1A, HSC70

No significant pathways enriched in SVZp vs SVZn comparison by Process Networks.

Table 9S: Enrichment analysis report. Enrichment by GO Processes (healthy control vs Grade II)

Enrichment by GO Processes					GRADE II_INPUT_genelist			
**#**	**Processes**	**Total**	**pValue**	**Min FDR**	**p-value**	**FDR**	**In Data**	**Network Objects from Active Data**
1	aorta morphogenesis	30	1.751E-09	6.483E-07	1.751E-09	6.483E-07	4	MYLK1, MLCK, EYA1, Telokin
2	aorta development	33	2.613E-09	6.483E-07	2.613E-09	6.483E-07	4	MYLK1, MLCK, EYA1, Telokin
3	aorta smooth muscle tissue morphogenesis	6	2.964E-09	6.483E-07	2.964E-09	6.483E-07	3	MYLK1, MLCK, Telokin
4	cellular hypotonic response	6	2.964E-09	6.483E-07	2.964E-09	6.483E-07	3	MYLK1, MLCK, Telokin
5	hypotonic response	8	8.293E-09	1.451E-06	8.293E-09	1.451E-06	3	MYLK1, MLCK, Telokin
6	bleb assembly	12	3.254E-08	4.745E-06	3.254E-08	4.745E-06	3	MYLK1, MLCK, Telokin
7	artery morphogenesis	78	8.982E-08	1.123E-05	8.982E-08	1.123E-05	4	MYLK1, MLCK, EYA1, Telokin
8	artery development	87	1.398E-07	1.390E-05	1.398E-07	1.390E-05	4	MYLK1, MLCK, EYA1, Telokin
9	tonic smooth muscle contraction	19	1.430E-07	1.390E-05	1.430E-07	1.390E-05	3	MYLK1, MLCK, Telokin
10	positive regulation of wound healing	22	2.270	1.986	2.270	1.986	3	MYLK1, MLCK, Telokin

			E-07	E-05	E-07	E-05		
11	smooth muscle tissue development	35	9.607E-07	7.642E-05	9.607E-07	7.642E-05	3	MYLK1, MLCK, Telokin
12	tissue morphogenesis	693	1.064E-06	7.761E-05	1.064E-06	7.761E-05	6	CD44, MYLK1, MLCK, LHX1, EYA1, Telokin
13	cellular response to osmotic stress	42	1.681E-06	1.132E-04	1.681E-06	1.132E-04	3	MYLK1, MLCK, Telokin
14	branching involved in ureteric bud morphogenesis	78	1.101E-05	6.882E-04	1.101E-05	6.882E-04	3	CD44, LHX1, EYA1
15	smooth muscle contraction	84	1.376E-05	8.029E-04	1.376E-05	8.029E-04	3	MYLK1, MLCK, Telokin
16	blood vessel development	623	1.606E-05	8.430E-04	1.606E-05	8.430E-04	5	CD44, MYLK1, MLCK, EYA1, Telokin
17	ureteric bud morphogenesis	89	1.638E-05	8.430E-04	1.638E-05	8.430E-04	3	CD44, LHX1, EYA1
18	organ morphogenesis	1127	1.776E-05	8.634E-04	1.776E-05	8.634E-04	6	CD44, MYLK1, MLCK, LHX1, EYA1, Telokin
19	vasculature development	668	2.249E-05	1.036E-03	2.249E-05	1.036E-03	5	CD44, MYLK1, MLCK, EYA1, Telokin
20	muscle tissue morphogenesis	104	2.614E-05	1.091E-03	2.614E-05	1.091E-03	3	MYLK1, MLCK, Telokin
21	tissue development	1879	2.619E-05	1.091E-03	2.619E-05	1.091E-03	7	CD44, VDAC 1, MYLK1, MLCK, LHX1, EYA1, Telokin

22	muscle organ morphogenesis	112	3.263E-05	1.298E-03	3.263E-05	1.298E-03	3	MYLK1, MLCK, Telokin
23	response to osmotic stress	116	3.623E-05	1.321E-03	3.623E-05	1.321E-03	3	MYLK1, MLCK, Telokin
24	positive regulation of calcium ion transport	116	3.623E-05	1.321E-03	3.623E-05	1.321E-03	3	MYLK1, MLCK, Telokin
25	ureteric bud development	138	6.082E-05	2.129E-03	6.082E-05	2.129E-03	3	CD44, LHX1, EYA1
26	regulation of wound healing	141	6.485E-05	2.182E-03	6.485E-05	2.182E-03	3	MYLK1, MLCK, Telokin
27	muscle tissue development	425	7.576E-05	2.455E-03	7.576E-05	2.455E-03	4	MYLK1, MLCK, EYA1, Telokin
28	positive regulation of response to wounding	160	9.440E-05	2.950E-03	9.440E-05	2.950E-03	3	MYLK1, MLCK, Telokin
29	actin filament organization	180	1.338E-04	4.038E-03	1.338E-04	4.038E-03	3	MYLK1, MLCK, Telokin
30	blood vessel morphogenesis	529	1.765E-04	5.148E-03	1.765E-04	5.148E-03	4	MYLK1, MLCK, EYA1, Telokin
31	circulatory system development	1074	2.160E-04	5.906E-03	2.160E-04	5.906E-03	5	CD44, MYLK1, MLCK, EYA1, Telokin
32	cardiovascular system development	1074	2.160E-04	5.906E-03	2.160E-04	5.906E-03	5	CD44, MYLK1, MLCK, EYA1, Telokin
33	branching morphogenesis of an epithelial tube	226	2.618E-	6.942E-	2.618E-	6.942E-	3	CD44, LHX1, EYA1

			04	03	04	03		
34	cell projection assembly	246	3.358E-04	8.642E-03	3.358E-04	8.642E-03	3	MYLK1, MLCK, Telokin
35	regulation of calcium ion transport	254	3.688E-04	8.938E-03	3.688E-04	8.938E-03	3	MYLK1, MLCK, Telokin
36	morphogenesis of a branching epithelium	254	3.688E-04	8.938E-03	3.688E-04	8.938E-03	3	CD44, LHX1, EYA1
37	positive regulation of response to stimulus	1943	3.784E-04	8.938E-03	3.784E-04	8.938E-03	6	CD44, IGHG1, MYLK1, MLCK, EYA1, Telokin
38	positive regulation of ion transport	259	3.905E-04	8.938E-03	3.905E-04	8.938E-03	3	MYLK1, MLCK, Telokin
39	morphogenesis of a branching structure	263	4.084E-04	8.938E-03	4.084E-04	8.938E-03	3	CD44, LHX1, EYA1
40	cell projection organization	1231	4.086E-04	8.938E-03	4.086E-04	8.938E-03	5	CD44, MYLK1, MLCK, LHX1, Telokin
41	regulation of response to stress	1242	4.258E-04	9.088E-03	4.258E-04	9.088E-03	5	CD44, MYLK1, MLCK, EYA1, Telokin
42	muscle contraction	289	5.379E-04	1.033E-02	5.379E-04	1.033E-02	3	MYLK1, MLCK, Telokin
43	regulation of ion transport	712	5.485E-04	1.033E-02	5.485E-04	1.033E-02	4	VDAC 1, MYLK1, MLCK, Telokin
44	horizontal cell localization	1	5.790E-04	1.033E-02	5.790E-04	1.033E-02	1	LHX1

45	uterine epithelium development	1	5.790E-04	1.033E-02	5.790E-04	1.033E-02	1	LHX1
46	oviduct epithelium development	1	5.790E-04	1.033E-02	5.790E-04	1.033E-02	1	LHX1
47	nephric duct elongation	1	5.790E-04	1.033E-02	5.790E-04	1.033E-02	1	LHX1
48	response to injury involved in regulation of muscle adaptation	1	5.790E-04	1.033E-02	5.790E-04	1.033E-02	1	NOL3
49	macrophage fusion	1	5.790E-04	1.033E-02	5.790E-04	1.033E-02	1	CD44
50	response to stress	4156	5.901E-04	1.033E-02	5.901E-04	1.033E-02	8	CD44, VDAC 1, IGHG1, MYLK1, NOL3, MLCK, EYA1, Telokin
51	regulation of metal ion transport	354	9.696E-04	1.654E-02	9.696E-04	1.654E-02	3	MYLK1, MLCK, Telokin
52	muscle system process	357	9.935E-04	1.654E-02	9.935E-04	1.654E-02	3	MYLK1, MLCK, Telokin
53	cellular response to abiotic stimulus	358	1.002E-03	1.654E-02	1.002E-03	1.654E-02	3	MYLK1, MLCK, Telokin
54	positive regulation of anterior head development	2	1.158E-03	1.842E-02	1.158E-03	1.842E-02	1	LHX1
55	regulation of anterior head development	2	1.158E-03	1.842E-02	1.158E-03	1.842E-02	1	LHX1
56	response to abiotic stimulus	155	1.183E-	1.848E-	1.183E-	1.848E-	5	MYLK1, NOL3, MLCK, EYA1, Telokin

		1	03	02	03	02		
57	renal system development	386	1.245E-03	1.911E-02	1.245E-03	1.911E-02	3	CD44, LHX1, EYA1
58	muscle organ development	399	1.369E-03	1.919E-02	1.369E-03	1.919E-02	3	MYLK1, MLCK, Telokin
59	epithelial tube morphogenesis	419	1.576E-03	1.919E-02	1.576E-03	1.919E-02	3	CD44, LHX1, EYA1
60	positive regulation of cell migration	419	1.576E-03	1.919E-02	1.576E-03	1.919E-02	3	MYLK1, MLCK, Telokin
61	negative regulation of cysteine-type endopeptidase activity involved in apoptotic process	104	1.593E-03	1.919E-02	1.593E-03	1.919E-02	2	CD44, NOL3
62	formation of primary germ layer	107	1.685E-03	1.919E-02	1.685E-03	1.919E-02	2	LHX1, EYA1
63	cytoskeleton organization	961	1.686E-03	1.919E-02	1.686E-03	1.919E-02	4	MYLK1, MLCK, EYA1, Telokin
64	regulation of anatomical structure morphogenesis	962	1.693E-03	1.919E-02	1.693E-03	1.919E-02	4	MLCK, PQBP-1, LHX1, EYA1
65	positive regulation of cell motility	430	1.697E-03	1.919E-02	1.697E-03	1.919E-02	3	MYLK1, MLCK, Telokin
66	negative regulation of cysteine-type endopeptidase activity	108	1.716E-03	1.919E-02	1.716E-03	1.919E-02	2	CD44, NOL3
67	positive regulation of fast-twitch skeletal muscle fiber contraction	3	1.736E-03	1.919E-02	1.736E-03	1.919E-02	1	MLCK

68	positive regulation of epithelial cell differentiation involved in kidney development	3	1.736E-03	1.919E-02	1.736E-03	1.919E-02	1	LHX1
69	positive regulation of nephron tubule epithelial cell differentiation	3	1.736E-03	1.919E-02	1.736E-03	1.919E-02	1	LHX1
70	regulation of twitch skeletal muscle contraction	3	1.736E-03	1.919E-02	1.736E-03	1.919E-02	1	MLCK
71	regulation of fast-twitch skeletal muscle fiber contraction	3	1.736E-03	1.919E-02	1.736E-03	1.919E-02	1	MLCK
72	positive regulation of neutrophil apoptotic process	3	1.736E-03	1.919E-02	1.736E-03	1.919E-02	1	CD44
73	positive regulation of leukocyte cell-cell adhesion	3	1.736E-03	1.919E-02	1.736E-03	1.919E-02	1	CD44
74	regulation of vascular permeability involved in acute inflammatory response	3	1.736E-03	1.919E-02	1.736E-03	1.919E-02	1	MLCK
75	positive regulation of monocyte aggregation	3	1.736E-03	1.919E-02	1.736E-03	1.919E-02	1	CD44
76	monocyte aggregation	3	1.736E-03	1.919E-02	1.736E-03	1.919E-02	1	CD44
77	cervix development	3	1.736E-03	1.919E-02	1.736E-03	1.919E-02	1	LHX1
78	regulation of monocyte aggregation	3	1.736E-03	1.919E-02	1.736E-03	1.919E-02	1	CD44
79	lateral motor column neuron migration	3	1.736E-	1.919E-	1.736E-	1.919E-	1	LHX1

			03	02	03	02		
80	tube morphogenesis	435	1.755E-03	1.919E-02	1.755E-03	1.919E-02	3	CD44, LHX1, EYA1
81	positive regulation of cellular component movement	443	1.849E-03	1.997E-02	1.849E-03	1.997E-02	3	MYLK1, MLCK, Telokin
82	anatomical structure morphogenesis	2618	1.890E-03	2.000E-02	1.890E-03	2.000E-02	6	CD44, MYLK1, MLCK, LHX1, EYA1, Telokin
83	urogenital system development	447	1.897E-03	2.000E-02	1.897E-03	2.000E-02	3	CD44, LHX1, EYA1
84	positive regulation of locomotion	449	1.921E-03	2.001E-02	1.921E-03	2.001E-02	3	MYLK1, MLCK, Telokin
85	metanephros development	117	2.010E-03	2.069E-02	2.010E-03	2.069E-02	2	LHX1, EYA1
86	metanephric part of ureteric bud development	4	2.314E-03	2.199E-02	2.314E-03	2.199E-02	1	LHX1
87	ectoderm formation	4	2.314E-03	2.199E-02	2.314E-03	2.199E-02	1	LHX1
88	spinal cord motor neuron migration	4	2.314E-03	2.199E-02	2.314E-03	2.199E-02	1	LHX1
89	sarcomerogenesis	4	2.314E-03	2.199E-02	2.314E-03	2.199E-02	1	MLCK
90	positive regulation of secondary heart field cardioblast proliferation	4	2.314E-03	2.199E-02	2.314E-03	2.199E-02	1	EYA1

91	oviduct development	4	2.314E-03	2.199E-02	2.314E-03	2.199E-02	1	LHX1
92	motor neuron migration	4	2.314E-03	2.199E-02	2.314E-03	2.199E-02	1	LHX1
93	actin cytoskeleton organization	482	2.352E-03	2.199E-02	2.352E-03	2.199E-02	3	MYLK1, MLCK, Telokin
94	embryonic skeletal system morphogenesis	127	2.362E-03	2.199E-02	2.362E-03	2.199E-02	2	LHX1, EYA1
95	epithelium development	1063	2.446E-03	2.253E-02	2.446E-03	2.253E-02	4	CD44, VDAC 1, LHX1, EYA1
96	regulation of response to wounding	491	2.479E-03	2.260E-02	2.479E-03	2.260E-02	3	MYLK1, MLCK, Telokin
97	regulation of response to stimulus	3878	2.611E-03	2.345E-02	2.611E-03	2.345E-02	7	CD44, IGHG1, MYLK1, NOL3, MLCK, EYA1, Telokin
98	macromolecule metabolic process	8229	2.627E-03	2.345E-02	2.627E-03	2.345E-02	10	CD44, IGHG1, MYLK1, NOL3, MLCK, PQBP-1, LHX1, RRP42, EYA1, Telokin
99	anatomical structure formation involved in morphogenesis	1091	2.690E-03	2.378E-02	2.690E-03	2.378E-02	4	CD44, MLCK, LHX1, EYA1
100	regulation of leukocyte cell-cell adhesion	5	2.892E-03	2.457E-02	2.892E-03	2.457E-02	1	CD44
101	paramesonephric duct development	5	2.892E-03	2.457E-02	2.892E-03	2.457E-02	1	LHX1
102	histone dephosphorylation	5	2.892E-	2.457E-	2.892E-	2.457E-	1	EYA1

			03	02	03	02		
103	early endosome to Golgi transport	5	2.892E-03	2.457E-02	2.892E-03	2.457E-02	1	SNX1
104	positive regulation of biological process	5249	3.034E-03	2.552E-02	3.034E-03	2.552E-02	8	CD44, IGHG1, MYLK1, MLCK, LHX1, EYA1, Telokin, SNX1
105	morphogenesis of an epithelium	529	3.063E-03	2.553E-02	3.063E-03	2.553E-02	3	CD44, LHX1, EYA1
106	actin filament-based process	532	3.113E-03	2.570E-02	3.113E-03	2.570E-02	3	MYLK1, MLCK, Telokin
107	satellite cell differentiation	6	3.470E-03	2.711E-02	3.470E-03	2.711E-02	1	MLCK
108	positive regulation of sarcomere organization	6	3.470E-03	2.711E-02	3.470E-03	2.711E-02	1	MLCK
109	negative regulation of mature B cell apoptotic process	6	3.470E-03	2.711E-02	3.470E-03	2.711E-02	1	CD44
110	regulation of mature B cell apoptotic process	6	3.470E-03	2.711E-02	3.470E-03	2.711E-02	1	CD44
111	regulation of neutrophil apoptotic process	6	3.470E-03	2.711E-02	3.470E-03	2.711E-02	1	CD44
112	regulation of muscle filament sliding	6	3.470E-03	2.711E-02	3.470E-03	2.711E-02	1	MLCK
113	regulation of response to DNA damage stimulus	160	3.716E-03	2.877E-02	3.716E-03	2.877E-02	2	CD44, EYA1

114	skeletal system development	578	3.934E-03	3.020E-02	3.934E-03	3.020E-02	3	CD44, LHX1, EYA1
115	metanephric comma-shaped body morphogenesis	7	4.047E-03	3.053E-02	4.047E-03	3.053E-02	1	LHX1
116	cerebellar Purkinje cell-granule cell precursor cell signaling involved in regulation of granule cell precursor cell proliferation	7	4.047E-03	3.053E-02	4.047E-03	3.053E-02	1	LHX1
117	embryonic skeletal system development	169	4.135E-03	3.092E-02	4.135E-03	3.092E-02	2	LHX1, EYA1
118	gastrulation	175	4.426E-03	3.282E-02	4.426E-03	3.282E-02	2	LHX1, EYA1
119	cellular component organization or biogenesis	5557	4.475E-03	3.289E-02	4.475E-03	3.289E-02	8	CD44, MYLK1, MLCK, PQBP-1, LHX1, RRP42, EYA1, Telokin
120	embryonic retina morphogenesis in camera-type eye	8	4.624E-03	3.289E-02	4.624E-03	3.289E-02	1	LHX1
121	positive regulation of homotypic cell-cell adhesion	8	4.624E-03	3.289E-02	4.624E-03	3.289E-02	1	CD44
122	outer ear morphogenesis	8	4.624E-03	3.289E-02	4.624E-03	3.289E-02	1	EYA1
123	metanephric S-shaped body morphogenesis	8	4.624E-03	3.289E-02	4.624E-03	3.289E-02	1	LHX1
124	mesodermal cell fate specification	9	5.200E-03	3.611E-02	5.200E-03	3.611E-02	1	EYA1
125	positive regulation of gastrulation	9	5.200E-	3.611E-	5.200E-	3.611E-	1	LHX1

			03	02	03	02		
126	semicircular canal morphogenesis	9	5.200E-03	3.611E-02	5.200E-03	3.611E-02	1	EYA1
127	muscle structure development	648	5.420E-03	3.709E-02	5.420E-03	3.709E-02	3	MYLK1, MLCK, Telokin
128	anatomical structure development	5718	5.426E-03	3.709E-02	5.426E-03	3.709E-02	8	CD44, VDAC 1, MYLK1, MLCK, PQBP-1, LHX1, EYA1, Telokin
129	tube development	660	5.705E-03	3.744E-02	5.705E-03	3.744E-02	3	CD44, LHX1, EYA1
130	regulation of secondary heart field cardioblast proliferation	10	5.777E-03	3.744E-02	5.777E-03	3.744E-02	1	EYA1
131	regulation of cardioblast proliferation	10	5.777E-03	3.744E-02	5.777E-03	3.744E-02	1	EYA1
132	comma-shaped body morphogenesis	10	5.777E-03	3.744E-02	5.777E-03	3.744E-02	1	LHX1
133	stress granule assembly	10	5.777E-03	3.744E-02	5.777E-03	3.744E-02	1	PQBP-1
134	positive regulation of heterotypic cell-cell adhesion	10	5.777E-03	3.744E-02	5.777E-03	3.744E-02	1	CD44
135	leukocyte aggregation	10	5.777E-03	3.744E-02	5.777E-03	3.744E-02	1	CD44
136	cellular component biogenesis	2239	6.043E-03	3.887E-02	6.043E-03	3.887E-02	5	MYLK1, MLCK, PQBP-1, RRP42, Telokin

137	regulation of organ morphogenesis	209	6.250E-03	3.887E-02	6.250E-03	3.887E-02	2	LHX1, EYA1
138	blood vessel maturation	11	6.353E-03	3.887E-02	6.353E-03	3.887E-02	1	CD44
139	pronephros development	11	6.353E-03	3.887E-02	6.353E-03	3.887E-02	1	LHX1
140	S-shaped body morphogenesis	11	6.353E-03	3.887E-02	6.353E-03	3.887E-02	1	LHX1
141	semicircular canal development	11	6.353E-03	3.887E-02	6.353E-03	3.887E-02	1	EYA1
142	nephric duct morphogenesis	11	6.353E-03	3.887E-02	6.353E-03	3.887E-02	1	LHX1
143	wound healing involved in inflammatory response	11	6.353E-03	3.887E-02	6.353E-03	3.887E-02	1	CD44
144	regulation of cell migration	698	6.666E-03	4.051E-02	6.666E-03	4.051E-02	3	MYLK1, MLCK, Telokin
145	mesonephric duct development	12	6.928E-03	4.069E-02	6.928E-03	4.069E-02	1	LHX1
146	positive regulation of cerebellar granule cell precursor proliferation	12	6.928E-03	4.069E-02	6.928E-03	4.069E-02	1	LHX1
147	positive regulation of myeloid cell apoptotic process	12	6.928E-03	4.069E-02	6.928E-03	4.069E-02	1	CD44
148	regulation of skeletal muscle contraction	12	6.928E-	4.069E-	6.928E-	4.069E-	1	MLCK

			03	02	03	02		
149	mitochondrial calcium ion transport	12	6.928E-03	4.069E-02	6.928E-03	4.069E-02	1	VDAC 1
150	embryonic viscerocranium morphogenesis	13	7.504E-03	4.291E-02	7.504E-03	4.291E-02	1	LHX1
151	metanephric renal vesicle morphogenesis	13	7.504E-03	4.291E-02	7.504E-03	4.291E-02	1	LHX1
152	primitive streak formation	13	7.504E-03	4.291E-02	7.504E-03	4.291E-02	1	LHX1
153	negative regulation of B cell apoptotic process	13	7.504E-03	4.291E-02	7.504E-03	4.291E-02	1	CD44
154	cellular response to fibroblast growth factor stimulus	232	7.649E-03	4.346E-02	7.649E-03	4.346E-02	2	CD44, LHX1
155	regulation of cell motility	737	7.749E-03	4.374E-02	7.749E-03	4.374E-02	3	MYLK1, MLCK, Telokin
156	response to fibroblast growth factor	236	7.905E-03	4.434E-02	7.905E-03	4.434E-02	2	CD44, LHX1
157	positive regulation of striated muscle contraction	14	8.079E-03	4.474E-02	8.079E-03	4.474E-02	1	MLCK
158	negative regulation of DNA damage response, signal transduction by p53 class mediator	14	8.079E-03	4.474E-02	8.079E-03	4.474E-02	1	CD44
159	negative regulation of apoptotic signaling pathway	242	8.297E-03	4.561E-02	8.297E-03	4.561E-02	2	CD44, EYA1

160	otic vesicle morphogenesis	15	8.653E-03	4.561E-02	8.653E-03	4.561E-02	1	EYA1
161	regulation of cerebellar granule cell precursor proliferation	15	8.653E-03	4.561E-02	8.653E-03	4.561E-02	1	LHX1
162	nephric duct development	15	8.653E-03	4.561E-02	8.653E-03	4.561E-02	1	LHX1
163	renal vesicle morphogenesis	15	8.653E-03	4.561E-02	8.653E-03	4.561E-02	1	LHX1
164	regulation of sarcomere organization	15	8.653E-03	4.561E-02	8.653E-03	4.561E-02	1	MLCK
165	somite rostral/caudal axis specification	15	8.653E-03	4.561E-02	8.653E-03	4.561E-02	1	LHX1
166	response to stimulus involved in regulation of muscle adaptation	15	8.653E-03	4.561E-02	8.653E-03	4.561E-02	1	NOL3
167	regulation of muscle system process	253	9.038E-03	4.736E-02	9.038E-03	4.736E-02	2	NOL3, MLCK
168	positive regulation of embryonic development	16	9.228E-03	4.778E-02	9.228E-03	4.778E-02	1	LHX1
169	hyaluronan catabolic process	16	9.228E-03	4.778E-02	9.228E-03	4.778E-02	1	CD44
170	regulation of cysteine-type endopeptidase activity involved in apoptotic process	259	9.455E-03	4.866E-02	9.455E-03	4.866E-02	2	CD44, NOL3
171	pharyngeal system development	17	9.802E-	4.894E-	9.802E-	4.894E-	1	EYA1

			03	02	03	02		
172	regulation of nephron tubule epithelial cell differentiation	17	9.802E-03	4.894E-02	9.802E-03	4.894E-02	1	LHX1
173	renal vesicle development	17	9.802E-03	4.894E-02	9.802E-03	4.894E-02	1	LHX1
174	branching involved in prostate gland morphogenesis	17	9.802E-03	4.894E-02	9.802E-03	4.894E-02	1	CD44
175	vagina development	17	9.802E-03	4.894E-02	9.802E-03	4.894E-02	1	LHX1
176	regulation of locomotion	804	9.843E-03	4.894E-02	9.843E-03	4.894E-02	3	MYLK1, MLCK, Telokin
177	metanephric glomerulus development	18	1.038E-02	4.990E-02	1.038E-02	4.990E-02	1	LHX1
178	cerebellar Purkinje cell layer formation	18	1.038E-02	4.990E-02	1.038E-02	4.990E-02	1	LHX1
179	regulation of cell proliferation involved in heart morphogenesis	18	1.038E-02	4.990E-02	1.038E-02	4.990E-02	1	EYA1
180	mesonephric tubule development	18	1.038E-02	4.990E-02	1.038E-02	4.990E-02	1	LHX1
181	cerebellar Purkinje cell differentiation	18	1.038E-02	4.990E-02	1.038E-02	4.990E-02	1	LHX1
182	regulation of cellular component movement	820	1.039E-02	4.990E-02	1.039E-02	4.990E-02	3	MYLK1, MLCK, Telokin

183	regulation of cysteine-type endopeptidase activity	274	1.053E-02	4.990E-02	1.053E-02	4.990E-02	2	CD44, NOL3
184	skeletal system morphogenesis	276	1.068E-02	4.990E-02	1.068E-02	4.990E-02	2	LHX1, EYA1
185	positive regulation of gene expression	1607	1.075E-02	4.990E-02	1.075E-02	4.990E-02	4	CD44, MLCK, LHX1, EYA1
186	system process	2568	1.086E-02	4.990E-02	1.086E-02	4.990E-02	5	VDAC 1, MYLK1, MLCK, EYA1, Telokin
187	single-organism developmental process	6348	1.086E-02	4.990E-02	1.086E-02	4.990E-02	8	CD44, VDAC 1, MYLK1, MLCK, PQBP-1, LHX1, EYA1, Telokin
188	protein phosphorylation	835	1.092E-02	4.990E-02	1.092E-02	4.990E-02	3	MYLK1, MLCK, Telokin
189	mesonephric epithelium development	19	1.095E-02	4.990E-02	1.095E-02	4.990E-02	1	LHX1
190	regulation of epithelial cell differentiation involved in kidney development	19	1.095E-02	4.990E-02	1.095E-02	4.990E-02	1	LHX1
191	negative regulation of intrinsic apoptotic signaling pathway in response to DNA damage by p53 class mediator	19	1.095E-02	4.990E-02	1.095E-02	4.990E-02	1	CD44
192	spinal cord association neuron differentiation	19	1.095E-02	4.990E-02	1.095E-02	4.990E-02	1	LHX1
193	organ development	3691	1.101E-02	4.991E-02	1.101E-02	4.991E-02	6	CD44, MYLK1, MLCK, LHX1, EYA1, Telokin

Table 9S: Enrichment analysis report. Enrichment by GO Processes (healthy control vs Grade III)								
Enrichment by GO Processes					GRADE III_INPUT_4(1)_genelist			
**#**	**Processes**	**Total**	**pValue**	**MinFDR**	**p-value**	**FDR**	**In Data**	**Network Objects from Active Data**
1	Notch receptor processing	29	2.399E-07	3.496E-04	2.399E-07	3.496E-04	6	RPS27A, Ubiquitin, UBC, Notch, UBB, NOTCH1 precursor
2	transport	4365	7.810E-07	3.496E-04	7.810E-07	3.496E-04	70	Annexin VIII, PEX2, KLC2, DLC1 (Dynein LC8a), CRK, HBG, DCTN2, VAMP5, STAT5A, GGA1, NASP, ERK1 (MAPK3), Carbonic anhydrase XIII, Bim, LASP1, CERT, STAT5, RBP-J kappa (CBF1), RBP1, MCT4, Autophagin-1, Tex1, KLC3, RPS27A, Ccdc109a, Ubiquitin, CDC4L, SNX9, Kinesin light chain, Phosphatase regulator (inhibitor), ATP1A3, Fatty acid-binding protein, ERK1/2, CRABP2, Syntaxin 16, Guanylate cyclase A (NPR1), GRB2, Rab-9B, PEX19, HXK4, DDC, BBS9, COG3, UBC, HBGA, SNX1, CED-6, NUP93, ANXA8L2, Dysbindin, Notch, UBB, Guanylate cyclase, IGHG1, TRS85 homolog, NOTCH1 precursor, GCK(MAP4K2), GGA, RAB24, IL1RN, DYNLL, E-FABP, Adult hemoglobin, GOCAP1, MLX, Rich1, COG8, APOBEC-1, RIP, MLCK
3	stress-activated MAPK cascade	175	7.811E-07	3.496E-04	7.811E-07	3.496E-04	11	CRK, TIRAP (Mal), p38 MAPK, ERK1 (MAPK3), RPS27A, Ubiquitin, ERK1/2, CrkL, UBC, UBB, GCK(MAP4K2)
4	glycogen biosynthetic process	35	7.824E	3.496E	7.824E	3.496E	6	RPS27A, Ubiquitin, Phosphatase regulator (inhibitor), HXK4, UBC, UBB

			-07	-04	-07	-04		
5	glucan biosynthetic process	35	7.824E-07	3.496E-04	7.824E-07	3.496E-04	6	RPS27A, Ubiquitin, Phosphatase regulator (inhibitor), HXK4, UBC, UBB
6	regulation of cell adhesion involved in heart morphogenesis	3	7.953E-07	3.496E-04	7.953E-07	3.496E-04	3	RBP-J kappa (CBF1), Notch, NOTCH1 precursor
7	positive regulation of ephrin receptor signaling pathway	3	7.953E-07	3.496E-04	7.953E-07	3.496E-04	3	RBP-J kappa (CBF1), Notch, NOTCH1 precursor
8	establishment of localization	4467	8.743E-07	3.496E-04	8.743E-07	3.496E-04	71	Annexin VIII, PEX2, KLC2, DLC1 (Dynein LC8a), CRK, HBG, DCTN2, VAMP5, STAT5A, GGA1, NASP, ERK1 (MAPK3), Carbonic anhydrase XIII, Bim, LASP1, CERT, STAT5, RBP-J kappa (CBF1), RBP1, MCT4, Autophagin-1, Tex1, KLC3, RPS27A, Ccdc109a, Ubiquitin, CDC4L, EYA1, SNX9, Kinesin light chain, Phosphatase regulator (inhibitor), ATP1A3, Fatty acid-binding protein, ERK1/2, CRABP2, Syntaxin 16, Guanylate cyclase A (NPR1), GRB2, Rab-9B, PEX19, HXK4, DDC, BBS9, COG3, UBC, HBGA, SNX1, CED-6, NUP93, ANXA8L2, Dysbindin, Notch, UBB, Guanylate cyclase, IGHG1, TRS85 homolog, NOTCH1 precursor, GCK(MAP4K2), GGA, RAB24, IL1RN, DYNLL, E-FABP, Adult hemoglobin, GOCAP1, MLX, Rich1, COG8, APOBEC-1, RIP, MLCK
9	stress-activated protein kinase signaling cascade	181	1.0	3.8	1.0	3.8	11	CRK, TIRAP (Mal), p38 MAPK, ERK1 (MAPK3), RPS27A, Ubiquitin, ERK1/2, CrkL, UBC, UBB, GCK(MAP4K2)

			90E-06	75E-04	90E-06	75E-04		
10	cellular response to growth factor stimulus	753	2.209E-06	6.407E-04	2.209E-06	6.407E-04	22	Reticulon 4, CRK, p38 MAPK, ERK1 (MAPK3), Bim, Metallothionein-I, STAT5, SMIF, RPS27A, p38gamma (MAPK12), Ubiquitin, Phosphatase regulator (inhibitor), ERK1/2, Metallothionein-IG, CrkL, GRB2, DDC, UBC, Notch, UBB, NOTCH1 precursor, WWOX
11	toll-like receptor 5 signaling pathway	94	2.748E-06	6.407E-04	2.748E-06	6.407E-04	8	TIRAP (Mal), p38 MAPK, ERK1 (MAPK3), RPS27A, Ubiquitin, ERK1/2, UBC, UBB
12	apoptotic process involved in embryonic digit morphogenesis	4	3.159E-06	6.407E-04	3.159E-06	6.407E-04	3	Bim, Notch, NOTCH1 precursor
13	regulation of ephrin receptor signaling pathway	4	3.159E-06	6.407E-04	3.159E-06	6.407E-04	3	RBP-J kappa (CBF1), Notch, NOTCH1 precursor
14	ubiquitin homeostasis	4	3.159	6.407	3.159	6.407	3	Ubiquitin, UBC, UBB

			E-06	E-04	E-06	E-04		
15	establishment of localization in cell	2015	3.171E-06	6.407E-04	3.171E-06	6.407E-04	40	Annexin VIII, PEX2, KLC2, DCTN2, VAMP5, GGA1, ERK1 (MAPK3), Bim, CERT, RBP-J kappa (CBF1), Tex1, KLC3, RPS27A, Ccdc109a, Ubiquitin, CDC4L, EYA1, SNX9, Kinesin light chain, Phosphatase regulator (inhibitor), ERK1/2, Syntaxin 16, PEX19, DDC, COG3, UBC, SNX1, ANXA8L2, Dysbindin, Notch, UBB, NOTCH1 precursor, GCK(MAP4K2), GGA, IL1RN, DYNLL, MLX, Rich1, RIP, MLCK
16	DNA damage induced protein phosphorylation	12	3.405E-06	6.407E-04	3.405E-06	6.407E-04	4	p38 MAPK, ERK1 (MAPK3), p38gamma (MAPK12), ERK1/2
17	cellular response to granulocyte macrophage colony-stimulating factor stimulus	12	3.405E-06	6.407E-04	3.405E-06	6.407E-04	4	STAT5A, STAT5, ERK1/2, Guanylate cyclase
18	toll-like receptor TLR6:TLR2 signaling pathway	99	4.063E-06	6.840E-04	4.063E-06	6.840E-04	8	TIRAP (Mal), p38 MAPK, ERK1 (MAPK3), RPS27A, Ubiquitin, ERK1/2, UBC, UBB
19	toll-like receptor TLR1:TLR2 signaling pathway	99	4.063E-	6.840E-	4.063E-	6.840E-	8	TIRAP (Mal), p38 MAPK, ERK1 (MAPK3), RPS27A, Ubiquitin, ERK1/2, UBC, UBB

			06	04	06	04		
20	toll-like receptor 2 signaling pathway	101	4.721E-06	7.437E-04	4.721E-06	7.437E-04	8	TIRAP (Mal), p38 MAPK, ERK1 (MAPK3), RPS27A, Ubiquitin, ERK1/2, UBC, UBB
21	response to granulocyte macrophage colony-stimulating factor	13	4.882E-06	7.437E-04	4.882E-06	7.437E-04	4	STAT5A, STAT5, ERK1/2, Guanylate cyclase
22	regulation of transcription from RNA polymerase II promoter in response to hypoxia	48	5.350E-06	7.638E-04	5.350E-06	7.638E-04	6	RPS27A, Ubiquitin, UBC, Notch, UBB, NOTCH1 precursor
23	response to growth factor	797	5.492E-06	7.638E-04	5.492E-06	7.638E-04	22	Reticulon 4, CRK, p38 MAPK, ERK1 (MAPK3), Bim, Metallothionein-I, STAT5, SMIF, RPS27A, p38gamma (MAPK12), Ubiquitin, Phosphatase regulator (inhibitor), ERK1/2, Metallothionein-IG, CrkL, GRB2, DDC, UBC, Notch, UBB, NOTCH1 precursor, WWOX
24	intracellular signal transduction	1918	6.185E-06	8.244E-04	6.185E-06	8.244E-04	38	MST3, DLC1 (Dynein LC8a), CRK, STAT5A, TIRAP (Mal), p38 MAPK, ERK1 (MAPK3), Bim, Metallothionein-I, STAT5, RPS27A, p38gamma (MAPK12), Ccdc109a, Ubiquitin, PhLP, Phosphatase regulator (inhibitor), ERK1/2, LCKBP1, Guanylate cyclase A (NPR1), CrkL, GRB2, Rab-9B, CNK1, HXK4, Metallothionein-II, UBC, Siglec-E, UBB, BMF, Guanylate cyclase, GCK(MAP4K2), 4E-BP1, RRAD, RAB24, DYNLL, WWOX, Rich1, DOK3

25	aorta morphogenesis	30	7.844E-06	1.004E-03	7.844E-06	1.004E-03	5	RBP-J kappa (CBF1), EYA1, MYLK1, Telokin, MLCK
26	artery morphogenesis	78	8.236E-06	1.013E-03	8.236E-06	1.013E-03	7	RBP-J kappa (CBF1), EYA1, MYLK1, Telokin, Notch, NOTCH1 precursor, MLCK
27	positive regulation of MAP kinase activity	270	9.134E-06	1.082E-03	9.134E-06	1.082E-03	12	CRK, TIRAP (Mal), p38 MAPK, ERK1 (MAPK3), RPS27A, Ubiquitin, ERK1/2, Ajuba, UBC, UBB, GCK(MAP4K2), IL1RN
28	MyD88-dependent toll-like receptor signaling pathway	112	1.018E-05	1.163E-03	1.018E-05	1.163E-03	8	TIRAP (Mal), p38 MAPK, ERK1 (MAPK3), RPS27A, Ubiquitin, ERK1/2, UBC, UBB
29	viral protein processing	16	1.216E-05	1.238E-03	1.216E-05	1.238E-03	4	RPS27A, Ubiquitin, UBC, UBB
30	activation of MAPK activity	19	1.	1.	1.	1.	10	CRK, TIRAP (Mal), p38 MAPK, ERK1 (MAPK3), RPS27A, Ubiquitin, ERK1/2, UBC, UBB, GCK(MAP4K2)

		1	268E-05	238E-03	268E-05	238E-03		
31	aorta development	33	1.277E-05	1.238E-03	1.277E-05	1.238E-03	5	RBP-J kappa (CBF1), EYA1, MYLK1, Telokin, MLCK
32	cellular response to stress	1685	1.430E-05	1.238E-03	1.430E-05	1.238E-03	34	MST3, TIPIN, CRK, TIRAP (Mal), RAD1, p38 MAPK, ERK1 (MAPK3), Bim, CERT, Autophagin-1, RPS27A, p38gamma (MAPK12), Ubiquitin, EYA1, XPF, Phosphatase regulator (inhibitor), Fatty acid-binding protein, ERK1/2, MYLK1, CrkL, GRB2, HXK4, UBC, Telokin, Notch, RAD23A, UBB, NOTCH1 precursor, GCK(MAP4K2), DBC1, RPA4, DSS1, Adult hemoglobin, MLCK
33	MAPK cascade	283	1.463E-05	1.238E-03	1.463E-05	1.238E-03	12	CRK, TIRAP (Mal), p38 MAPK, ERK1 (MAPK3), RPS27A, p38gamma (MAPK12), Ubiquitin, ERK1/2, CrkL, UBC, UBB, GCK(MAP4K2)
34	cellular polysaccharide biosynthetic process	57	1.476E-05	1.238E-03	1.476E-05	1.238E-03	6	RPS27A, Ubiquitin, Phosphatase regulator (inhibitor), HXK4, UBC, UBB
35	signal transduction by phosphorylation	332	1.49	1.23	1.49	1.23	13	MST3, CRK, TIRAP (Mal), p38 MAPK, ERK1 (MAPK3), RPS27A, p38gamma (MAPK12), Ubiquitin, ERK1/2, CrkL, UBC, UBB, GCK(MAP4K2)

			7E-05	8E-03	7E-05	8E-03		
36	aorta smooth muscle tissue morphogenesis	6	1.558E-05	1.238E-03	1.558E-05	1.238E-03	3	MYLK1, Telokin, MLCK
37	positive regulation of transcription of Notch receptor target	6	1.558E-05	1.238E-03	1.558E-05	1.238E-03	3	RBP-J kappa (CBF1), Notch, NOTCH1 precursor
38	arterial endothelial cell differentiation	6	1.558E-05	1.238E-03	1.558E-05	1.238E-03	3	RBP-J kappa (CBF1), Notch, NOTCH1 precursor
39	interleukin-4 secretion	6	1.558E-05	1.238E-03	1.558E-05	1.238E-03	3	RBP-J kappa (CBF1), Notch, NOTCH1 precursor
40	cellular hypotonic response	6	1.558E	1.238E	1.558E	1.238E	3	MYLK1, Telokin, MLCK

			-05	-03	-05	-03		
41	endosomal transport	196	1.587E-05	1.238E-03	1.587E-05	1.238E-03	10	Annexin VIII, RPS27A, Ubiquitin, CDC4L, SNX9, Syntaxin 16, UBC, SNX1, ANXA8L2, UBB
42	polysaccharide biosynthetic process	58	1.633E-05	1.244E-03	1.633E-05	1.244E-03	6	RPS27A, Ubiquitin, Phosphatase regulator (inhibitor), HXK4, UBC, UBB
43	artery development	87	1.698E-05	1.263E-03	1.698E-05	1.263E-03	7	RBP-J kappa (CBF1), EYA1, MYLK1, Telokin, Notch, NOTCH1 precursor, MLCK
44	G2/M transition of mitotic cell cycle	200	1.890E-05	1.315E-03	1.890E-05	1.315E-03	10	DLC1 (Dynein LC8a), DCTN2, PPP1R12B, RPS27A, Ubiquitin, Phosphatase regulator (inhibitor), Ajuba, UBC, UBB, DYNLL
45	cell cycle G2/M phase transition	200	1.890E-0	1.315E-0	1.890E-0	1.315E-0	10	DLC1 (Dynein LC8a), DCTN2, PPP1R12B, RPS27A, Ubiquitin, Phosphatase regulator (inhibitor), Ajuba, UBC, UBB, DYNLL

			5	3	5	3		
46	positive regulation of innate immune response	245	1.975E-05	1.315E-03	1.975E-05	1.315E-03	11	STAT5A, TIRAP (Mal), p38 MAPK, ERK1 (MAPK3), STAT5, RPS27A, Ubiquitin, ERK1/2, UBC, UBB, SAP
47	cellular response to vascular endothelial growth factor stimulus	36	1.983E-05	1.315E-03	1.983E-05	1.315E-03	5	p38 MAPK, Metallothionein-I, Metallothionein-IG, Notch, NOTCH1 precursor
48	neurotrophin TRK receptor signaling pathway	341	1.983E-05	1.315E-03	1.983E-05	1.315E-03	13	Reticulon 4, CRK, p38 MAPK, ERK1 (MAPK3), Bim, RPS27A, p38gamma (MAPK12), Ubiquitin, ERK1/2, CrkL, GRB2, UBC, UBB
49	regulation of cell proliferation involved in heart morphogenesis	18	2.014E-05	1.315E-03	2.014E-05	1.315E-03	4	RBP-J kappa (CBF1), EYA1, Notch, NOTCH1 precursor
50	regulation of transcription from RNA polymerase II promoter in response to stress	61	2.189E-05	1.401E-03	2.189E-05	1.401E-03	6	RPS27A, Ubiquitin, UBC, Notch, UBB, NOTCH1 precursor

51	JNK cascade	125	2.272E-05	1.421E-03	2.272E-05	1.421E-03	8	CRK, TIRAP (Mal), RPS27A, Ubiquitin, CrkL, UBC, UBB, GCK(MAP4K2)
52	neurotrophin signaling pathway	346	2.309E-05	1.421E-03	2.309E-05	1.421E-03	13	Reticulon 4, CRK, p38 MAPK, ERK1 (MAPK3), Bim, RPS27A, p38gamma (MAPK12), Ubiquitin, ERK1/2, CrkL, GRB2, UBC, UBB
53	virion assembly	19	2.533E-05	1.518E-03	2.533E-05	1.518E-03	4	RPS27A, Ubiquitin, UBC, UBB
54	toll-like receptor 10 signaling pathway	93	2.626E-05	1.518E-03	2.626E-05	1.518E-03	7	p38 MAPK, ERK1 (MAPK3), RPS27A, Ubiquitin, ERK1/2, UBC, UBB
55	response to zinc ion	63	2.638E-05	1.518E-03	2.638E-05	1.518E-03	6	Metallothionein-I, Metallothionein-IG, Metallothionein-II, HMBS, Metallothionein-1M, APOBEC-1
56	nucleotide biosynthetic process	25	2.	1.	2.	1.	11	MIBP, GDP-mannose 4,6 dehydratase, UCK1, IMD2, ATP1A3, ADSL, Guanylate cyclase A (NPR1), RIFK, PAP41,

		3	657E-05	518E-03	657E-05	518E-03		Guanylate cyclase, CTP synthase II
57	nucleoside phosphate biosynthetic process	254	2.755E-05	1.546E-03	2.755E-05	1.546E-03	11	MIBP, GDP-mannose 4,6 dehydratase, UCK1, IMD2, ATP1A3, ADSL, Guanylate cyclase A (NPR1), RIFK, PAP41, Guanylate cyclase, CTP synthase II
58	cellular localization	2374	2.926E-05	1.614E-03	2.926E-05	1.614E-03	42	Annexin VIII, PEX2, KLC2, DCTN2, VAMP5, GGA1, ERK1 (MAPK3), Bim, CERT, RBP-J kappa (CBF1), Tex1, KLC3, RPS27A, Ccdc109a, Ubiquitin, CDC4L, EYA1, SNX9, Kinesin light chain, Phosphatase regulator (inhibitor), ERK1/2, Syntaxin 16, Ajuba, PTP-2, PEX19, DDC, COG3, UBC, SNX1, ANXA8L2, Dysbindin, Notch, UBB, NOTCH1 precursor, GCK(MAP4K2), GGA, IL1RN, DYNLL, MLX, Rich1, RIP, MLCK
59	localization	5388	3.039E-05	1.648E-03	3.039E-05	1.648E-03	76	Annexin VIII, PEX2, KLC2, Reticulon 4, DLC1 (Dynein LC8a), CRK, HBG, DCTN2, VAMP5, STAT5A, GGA1, NASP, ERK1 (MAPK3), Carbonic anhydrase XIII, Bim, LASP1, CERT, STAT5, RBP-J kappa (CBF1), RBP1, Twinfilin, MCT4, Autophagin-1, Tex1, KLC3, RPS27A, Ccdc109a, Ubiquitin, CDC4L, EYA1, SNX9, Kinesin light chain, Phosphatase regulator (inhibitor), ATP1A3, Fatty acid-binding protein, ERK1/2, CRABP2, Syntaxin 16, Guanylate cyclase A (NPR1), NEPH2, Ajuba, GRB2, Rab-9B, PTP-2, PEX19, HXK4, DDC, BBS9, COG3, UBC, HBGA, SNX1, CED-6, NUP93, ANXA8L2, Dysbindin, Notch, UBB, Guanylate cyclase, IGHG1, TRS85 homolog, NOTCH1 precursor, GCK(MAP4K2), GGA, RAB24, IL1RN, DYNLL, E-FABP, Adult hemoglobin, GOCAP1, MLX, Rich1, COG8, APOBEC-1, RIP, MLCK
60	cellular response to zinc ion	20	3.143E-0	1.672E-0	3.143E-0	1.672E-0	4	Metallothionein-I, Metallothionein-IG, Metallothionein-II, Metallothionein-1M

			5	3	5	3		
61	cellular response to cytokine stimulus	701	3.189E-05	1.672E-03	3.189E-05	1.672E-03	19	STAT5A, TIRAP (Mal), ERK1 (MAPK3), Metallothionein-I, STAT5, RPS27A, Ubiquitin, IMD2, ERK1/2, LCKBP1, Metallothionein-II, HMBS, UBC, GBP5, NUP93, UBB, Guanylate cyclase, PYDC1 (POP1), MLCK
62	toll-like receptor 4 signaling pathway	132	3.365E-05	1.736E-03	3.365E-05	1.736E-03	8	TIRAP (Mal), p38 MAPK, ERK1 (MAPK3), RPS27A, Ubiquitin, ERK1/2, UBC, UBB
63	toll-like receptor 9 signaling pathway	100	4.201E-05	1.995E-03	4.201E-05	1.995E-03	7	p38 MAPK, ERK1 (MAPK3), RPS27A, Ubiquitin, ERK1/2, UBC, UBB
64	interleukin-4 production	8	4.303E-05	1.995E-03	4.303E-05	1.995E-03	3	RBP-J kappa (CBF1), Notch, NOTCH1 precursor
65	regulation of transcription from RNA polymerase II promoter involved in myocardial precursor cell differentiation	8	4.303E-05	1.995E-03	4.303E-05	1.995E-03	3	RBP-J kappa (CBF1), Notch, NOTCH1 precursor

66	endocardium morphogenesis	8	4.303E-05	1.995E-03	4.303E-05	1.995E-03	3	RBP-J kappa (CBF1), Notch, NOTCH1 precursor
67	auditory receptor cell fate commitment	8	4.303E-05	1.995E-03	4.303E-05	1.995E-03	3	RBP-J kappa (CBF1), Notch, NOTCH1 precursor
68	inner ear receptor cell fate commitment	8	4.303E-05	1.995E-03	4.303E-05	1.995E-03	3	RBP-J kappa (CBF1), Notch, NOTCH1 precursor
69	hypotonic response	8	4.303E-05	1.995E-03	4.303E-05	1.995E-03	3	MYLK1, Telokin, MLCK
70	regulation of DNA-templated transcription in response to stress	69	4.444E-05	2.031E-03	4.444E-05	2.031E-03	6	RPS27A, Ubiquitin, UBC, Notch, UBB, NOTCH1 precursor
71	regulation of muscle cell differentiation	17	4.	2.	4.	2.	9	LAGY, MIBP, p38 MAPK, RBP-J kappa (CBF1), p38gamma (MAPK12), NIP2, Notch, NOTCH1 precursor, MLCK

		8	551E-05	051E-03	551E-05	051E-03		
72	positive regulation of response to stimulus	1943	4.778E-05	2.072E-03	4.778E-05	2.072E-03	36	MST3, CRK, STAT5A, TIRAP (Mal), p38 MAPK, ERK1 (MAPK3), Bim, LASP1, STAT5, RBP-J kappa (CBF1), RPS27A, Ubiquitin, EYA1, SCOCO, Fatty acid-binding protein, ERK1/2, NIP2, MYLK1, LCKBP1, CrkL, Ajuba, GRB2, UBC, Telokin, Notch, UBB, BMF, IGHG1, NOTCH1 precursor, GCK(MAP4K2), DBC1, IL1RN, WWOX, ACP33, SAP, MLCK
73	myoblast differentiation	43	4.803E-05	2.072E-03	4.803E-05	2.072E-03	5	p38 MAPK, RBP-J kappa (CBF1), p38gamma (MAPK12), Notch, NOTCH1 precursor
74	cellular carbohydrate biosynthetic process	70	4.824E-05	2.072E-03	4.824E-05	2.072E-03	6	RPS27A, Ubiquitin, Phosphatase regulator (inhibitor), HXK4, UBC, UBB
75	response to stress	4156	4.857E-05	2.072E-03	4.857E-05	2.072E-03	62	MST3, Annexin VIII, KLC2, TIPIN, CRK, RNASE6, HBG, TIRAP (Mal), RAD1, p38 MAPK, ERK1 (MAPK3), Bim, Metallothionein-I, CERT, STAT5, RBP-J kappa (CBF1), Autophagin-1, RPS27A, p38gamma (MAPK12), Ubiquitin, Sti1, EYA1, Kinesin light chain, XPF, Phosphatase regulator (inhibitor), Fatty acid-binding protein, ERK1/2, ADSL, MYLK1, CrkL, Ajuba, GRB2, HXK4, Metallothionein-II, HMBS, UBC, HBGA, Telokin, SAAL1, GBP5, ANXA8L2, Dysbindin, Notch, RAD23A, UBB, Guanylate cyclase, IGHG1, CALCOCO2, NOTCH1 precursor, GCK(MAP4K2), 4E-BP1, DBC1, IL1RN, RPA4, DSS1, E-FABP, PYDC1 (POP1), Adult hemoglobin, SAP, APOBEC-1, SPRR3, MLCK

76	intracellular transport	1491	5.187E-05	2.151E-03	5.187E-05	2.151E-03	30	Annexin VIII, PEX2, KLC2, VAMP5, GGA1, ERK1 (MAPK3), Bim, CERT, Tex1, KLC3, RPS27A, Ccdc109a, Ubiquitin, CDC4L, SNX9, Kinesin light chain, Phosphatase regulator (inhibitor), ERK1/2, Syntaxin 16, PEX19, COG3, UBC, SNX1, ANXA8L2, Dysbindin, UBB, GGA, DYNLL, MLX, RIP
77	transport of virus	44	5.379E-05	2.151E-03	5.379E-05	2.151E-03	5	Tex1, RPS27A, Ubiquitin, UBC, UBB
78	intracellular transport of virus	44	5.379E-05	2.151E-03	5.379E-05	2.151E-03	5	Tex1, RPS27A, Ubiquitin, UBC, UBB
79	multi-organism intracellular transport	44	5.379E-05	2.151E-03	5.379E-05	2.151E-03	5	Tex1, RPS27A, Ubiquitin, UBC, UBB
80	multi-organism transport	44	5.379E-05	2.151E-03	5.379E-05	2.151E-03	5	Tex1, RPS27A, Ubiquitin, UBC, UBB
81	TRIF-dependent toll-like receptor signaling pathway	10	5.	2.	5.	2.	7	p38 MAPK, ERK1 (MAPK3), RPS27A, Ubiquitin, ERK1/2, UBC, UBB

		5	744E-05	268E-03	744E-05	268E-03		
82	blood vessel endothelial cell differentiation	9	6.410E-05	2.501E-03	6.410E-05	2.501E-03	3	RBP-J kappa (CBF1), Notch, NOTCH1 precursor
83	cellular component organization	5419	7.021E-05	2.706E-03	7.021E-05	2.706E-03	75	MST3, Neurochondrin, Annexin VIII, PEX2, LAGY, Reticulon 4, ZNF261, DLC1 (Dynein LC8a), TIPIN, SCMH1, HBG, DCTN2, VAMP5, Calpain 1(mu), p38 MAPK, NASP, ERK1 (MAPK3), Bim, CERT, EFHD1, RBP-J kappa (CBF1), Twinfilin, Autophagin-1, KLC3, RPS27A, Ccdc109a, Ubiquitin, IMD2, EYA1, PhLP, SNX9, Kinesin light chain, XPF, Phosphatase regulator (inhibitor), PDXP, Fatty acid-binding protein, ERK1/2, NIP2, ADSL, ARHGEF10, MYLK1, PQBP-1, Kizuna (C20orf19), LCKBP1, NEPH2, GA17, Ajuba, GRB2, PEX19, Grancalcin, Rap1GDS1, BBS9, Tropomodulin, UBC, HBGA, Telokin, ST13 (Hip), CED-6, NUP93, ANXA8L2, Dysbindin, Notch, UBB, ARID4A, NOTCH1 precursor, DBC1, BMP1, DYNLL, Occludin, Adult hemoglobin, Rich1, p47, RIP, MLCK, PAF1
84	toll-like receptor 3 signaling pathway	109	7.288E-05	2.743E-03	7.288E-05	2.743E-03	7	p38 MAPK, ERK1 (MAPK3), RPS27A, Ubiquitin, ERK1/2, UBC, UBB
85	MyD88-independent toll-like receptor signaling pathway	109	7.288E-05	2.743E-03	7.288E-05	2.743E-03	7	p38 MAPK, ERK1 (MAPK3), RPS27A, Ubiquitin, ERK1/2, UBC, UBB

86	organic substance transport	2147	7.589E-05	2.823E-03	7.589E-05	2.823E-03	38	PEX2, VAMP5, GGA1, NASP, ERK1 (MAPK3), Carbonic anhydrase XIII, CERT, RBP-J kappa (CBF1), MCT4, Autophagin-1, Tex1, RPS27A, SNX9, Kinesin light chain, Phosphatase regulator (inhibitor), Fatty acid-binding protein, ERK1/2, CRABP2, Syntaxin 16, Rab-9B, PEX19, HXK4, DDC, BBS9, COG3, SNX1, CED-6, NUP93, Notch, NOTCH1 precursor, GGA, RAB24, IL1RN, E-FABP, Adult hemoglobin, COG8, APOBEC-1, RIP
87	cardiac epithelial to mesenchymal transition	25	7.913E-05	2.910E-03	7.913E-05	2.910E-03	4	Reticulon 4, RBP-J kappa (CBF1), Notch, NOTCH1 precursor
88	response to cytokine	884	8.151E-05	2.963E-03	8.151E-05	2.963E-03	21	STAT5A, TIRAP (Mal), ERK1 (MAPK3), Metallothionein-I, STAT5, RPS27A, Ubiquitin, IMD2, ERK1/2, LCKBP1, Metallothionein-II, HMBS, UBC, GBP5, NUP93, UBB, Guanylate cyclase, CALCOCO2, IL1RN, PYDC1 (POP1), MLCK
89	Notch signaling pathway involved in regulation of secondary heart field cardioblast proliferation	2	8.628E-05	2.968E-03	8.628E-05	2.968E-03	2	Notch, NOTCH1 precursor
90	regulation of apolipoprotein binding	2	8.628E-05	2.968E-03	8.628E-05	2.968E-03	2	ERK1 (MAPK3), ERK1/2
91	positive regulation of mast cell differentiation	2	8.	2.	8.	2.	2	STAT5A, STAT5

			628E-05	968E-03	628E-05	968E-03		
92	venous endothelial cell differentiation	2	8.628E-05	2.968E-03	8.628E-05	2.968E-03	2	Notch, NOTCH1 precursor
93	negative regulation of apolipoprotein binding	2	8.628E-05	2.968E-03	8.628E-05	2.968E-03	2	ERK1 (MAPK3), ERK1/2
94	regulation of secondary heart field cardioblast proliferation	10	9.094E-05	3.031E-03	9.094E-05	3.031E-03	3	EYA1, Notch, NOTCH1 precursor
95	regulation of cardioblast proliferation	10	9.094E-05	3.031E-03	9.094E-05	3.031E-03	3	EYA1, Notch, NOTCH1 precursor
96	regulation of response to stress	1242	9.17	3.03	9.17	3.03	26	MST3, LAGY, Reticulon 4, CRK, STAT5A, TIRAP (Mal), p38 MAPK, ERK1 (MAPK3), STAT5, RPS27A, Ubiquitin, EYA1, SCOCO, Fatty acid-binding protein, ERK1/2, MYLK1, Ajuba, UBC, Telokin, UBB, GCK(MAP4K2), DBC1, IL1RN,

			0E-05	1E-03	0E-05	1E-03		Adult hemoglobin, SAP, MLCK
97	cellular component organization or biogenesis	557	9.200E-05	3.031E-03	9.200E-05	3.031E-03	76	MST3, Neurochondrin, Annexin VIII, PEX2, LAGY, Reticulon 4, ZNF261, DLC1 (Dynein LC8a), TIPIN, SCMH1, HBG, DCTN2, VAMP5, Calpain 1(mu), p38 MAPK, NASP, ERK1 (MAPK3), Bim, CERT, EFHD1, RBP-J kappa (CBF1), Twinfilin, Autophagin-1, KLC3, RPS27A, Ccdc109a, Ubiquitin, IMD2, EYA1, PhLP, SNX9, Kinesin light chain, XPF, Phosphatase regulator (inhibitor), PDXP, Fatty acid-binding protein, ERK1/2, NIP2, ADSL, ARHGEF10, MYLK1, PQBP-1, Kizuna (C20orf19), LCKBP1, NEPH2, GA17, Ajuba, GRB2, PEX19, Grancalcin, Rap1GDS1, BBS9, Tropomodulin, RRP42, UBC, HBGA, Telokin, ST13 (Hip), CED-6, NUP93, ANXA8L2, Dysbindin, Notch, UBB, ARID4A, NOTCH1 precursor, DBC1, BMP1, DYNLL, Occludin, Adult hemoglobin, Rich1, p47, RIP, MLCK, PAF1
98	regulation of heart morphogenesis	26	9.284E-05	3.031E-03	9.284E-05	3.031E-03	4	RBP-J kappa (CBF1), EYA1, Notch, NOTCH1 precursor
99	positive regulation of protein serine/threonine kinase activity	346	1.025E-04	3.313E-03	1.025E-04	3.313E-03	12	CRK, TIRAP (Mal), p38 MAPK, ERK1 (MAPK3), RPS27A, Ubiquitin, ERK1/2, Ajuba, UBC, UBB, GCK(MAP4K2), IL1RN
100	single-organism biosynthetic process	1405	1.118E-04	3.551E-03	1.118E-04	3.551E-03	28	PEX2, MIBP, GDP-mannose 4,6 dehydratase, CERT, RBP1, UCK1, RPS27A, Ubiquitin, IMD2, NANP, Phosphatase regulator (inhibitor), ATP1A3, Fatty acid-binding protein, ADSL, CRABP2, Guanylate cyclase A (NPR1), Ajuba, HXK4, RIFK, HMBS, UBC, UBB, PAP41, Guanylate cyclase, CTP synthase II, RPA4, E-FABP, GOCAP1

101	phosphorylation	1331	1.121E-04	3.551E-03	1.121E-04	3.551E-03	27	MST3, MIBP, CRK, STAT5A, TIRAP (Mal), p38 MAPK, ERK1 (MAPK3), CERT, STAT5, Twinfilin, UCK1, RPS27A, p38gamma (MAPK12), Ubiquitin, ERK1/2, MYLK1, Guanylate cyclase A (NPR1), CrkL, CNK1, HXK4, RIFK, UBC, Telokin, UBB, Guanylate cyclase, GCK(MAP4K2), MLCK
102	phosphate-containing compound metabolic process	2859	1.165E-04	3.654E-03	1.165E-04	3.654E-03	46	MST3, MIBP, GDP-mannose 4,6 dehydratase, CRK, STAT5A, TIRAP (Mal), p38 MAPK, ERK1 (MAPK3), CERT, STAT5, Twinfilin, UCK1, RPS27A, p38gamma (MAPK12), Ubiquitin, IMD2, EYA1, NANP, DUPD1, Phosphatase regulator (inhibitor), PDXP, ATP1A3, Fatty acid-binding protein, ERK1/2, ADSL, MYLK1, Guanylate cyclase A (NPR1), MOCS2(small), CrkL, Ajuba, PTP-2, CNK1, HXK4, RIFK, UBC, Telokin, GBP5, UBB, PAP41, Guanylate cyclase, GCK(MAP4K2), RRAD, CTP synthase II, E-FABP, MLCK, Galectin-13
103	cardiac muscle cell myoblast differentiation	11	1.242E-04	3.784E-03	1.242E-04	3.784E-03	3	RBP-J kappa (CBF1), Notch, NOTCH1 precursor
104	Notch signaling involved in heart development	11	1.242E-04	3.784E-03	1.242E-04	3.784E-03	3	RBP-J kappa (CBF1), Notch, NOTCH1 precursor
105	epithelial to mesenchymal transition involved in endocardial cushion formation	11	1.242E-04	3.784E-03	1.242E-04	3.784E-03	3	RBP-J kappa (CBF1), Notch, NOTCH1 precursor

106	positive regulation of response to wounding	160	1.306E-04	3.943E-03	1.306E-04	3.943E-03	8	MST3, STAT5A, p38 MAPK, STAT5, Fatty acid-binding protein, MYLK1, Telokin, MLCK
107	macromolecule localization	2206	1.339E-04	3.977E-03	1.339E-04	3.977E-03	38	PEX2, VAMP5, STAT5A, GGA1, NASP, ERK1 (MAPK3), CERT, STAT5, RBP-J kappa (CBF1), Twinfilin, Autophagin-1, Tex1, RPS27A, CDC4L, EYA1, SNX9, Kinesin light chain, Phosphatase regulator (inhibitor), Fatty acid-binding protein, ERK1/2, CRABP2, Syntaxin 16, Ajuba, Rab-9B, PTP-2, PEX19, BBS9, COG3, SNX1, CED-6, NUP93, Notch, NOTCH1 precursor, GGA, RAB24, COG8, APOBEC-1, RIP
108	glycogen metabolic process	84	1.343E-04	3.977E-03	1.343E-04	3.977E-03	6	RPS27A, Ubiquitin, Phosphatase regulator (inhibitor), HXK4, UBC, UBB
109	toll-like receptor signaling pathway	161	1.364E-04	3.981E-03	1.364E-04	3.981E-03	8	TIRAP (Mal), p38 MAPK, ERK1 (MAPK3), RPS27A, Ubiquitin, ERK1/2, UBC, UBB
110	regulation of metabolic process	7302	1.369E-04	3.981E-03	1.369E-04	3.981E-03	93	SAE1, Annexin VIII, PEX2, LAGY, DLC1 (Dynein LC8a), LCORL, TIPIN, SCMH1, CRK, HBG, PPP1R12B, STAT5A, TIRAP (Mal), GGA1, p38 MAPK, ERK1 (MAPK3), Bim, STAT5, RBP-J kappa (CBF1), FERD3L, SMIF, Twinfilin, BMAL2, Autophagin-1, RPS27A, p38gamma (MAPK12), ZNF397, Ubiquitin, EYA1, PhLP, SNX9, SCOCO, XPF, Phosphatase regulator (inhibitor), Fatty acid-binding protein, ERK1/2, NIP2, ARHGEF10, CRABP2, PQBP-1, LCKBP1, Guanylate cyclase A (NPR1), GA17, CrkL, Ajuba, GRB2, PEX19, HXK4, RIFK, Rap1GDS1, ZNF655, COG3, UBC, HBGA, SNX1, PCP2, ST13 (Hip), HKR1, ANXA8L2, Dysbindin, Notch, RAD23A, UBB, PAP41, IRX4, Guanylate

								cyclase, ARID4A, Cyclophilin E, NOTCH1 precursor, GCK(MAP4K2), 4E-BP1, SCML1, DBC1, USP5, AO7, GGA, IL1RN, FAM116A, ZNF397OS, DYNLL, Cystatin B, WWOX, DKK3, PYDC1 (POP1), Adult hemoglobin, PAIP1, MLX, CAMK2N1, Rich1, APOBEC-1, RIP, MLCK, PAF1
111	[glucan metabolic process]()	85	1.434E-04	4.095E-03	1.434E-04	4.095E-03	6	RPS27A, Ubiquitin, Phosphatase regulator (inhibitor), HXK4, UBC, UBB
112	[cellular glucan metabolic process]()	85	1.434E-04	4.095E-03	1.434E-04	4.095E-03	6	RPS27A, Ubiquitin, Phosphatase regulator (inhibitor), HXK4, UBC, UBB
113	[cell cycle]()	1580	1.466E-04	4.149E-03	1.466E-04	4.149E-03	30	DLC1 (Dynein LC8a), TIPIN, DCTN2, PPP1R12B, RAD1, p38 MAPK, NASP, ERK1 (MAPK3), RPS27A, p38gamma (MAPK12), Ubiquitin, EYA1, SNX9, XPF, Phosphatase regulator (inhibitor), Fatty acid-binding protein, ERK1/2, ARHGEF10, Kizuna (C20orf19), Ajuba, ZNF655, UBC, NUP93, Notch, UBB, 4E-BP1, DBC1, GINS2, RPA4, DYNLL
114	[cell cycle phase transition]()	416	1.496E-04	4.175E-03	1.496E-04	4.175E-03	13	DLC1 (Dynein LC8a), TIPIN, DCTN2, PPP1R12B, RPS27A, Ubiquitin, Phosphatase regulator (inhibitor), Ajuba, UBC, UBB, 4E-BP1, RPA4, DYNLL
115	[glucose metabolic process]()	208	1.501E	4.175E	1.501E	4.175E	9	p38 MAPK, RPS27A, Ubiquitin, Phosphatase regulator (inhibitor), Fatty acid-binding protein, HXK4, UBC, UBB, E-FABP

			-04	-03	-04	-03		
116	single-organism transport	3506	1.555E-04	4.276E-03	1.555E-04	4.276E-03	53	Annexin VIII, PEX2, KLC2, CRK, HBG, DCTN2, VAMP5, STAT5A, ERK1 (MAPK3), Carbonic anhydrase XIII, Bim, LASP1, CERT, STAT5, RBP-J kappa (CBF1), MCT4, KLC3, RPS27A, Ccdc109a, Ubiquitin, CDC4L, SNX9, Kinesin light chain, Phosphatase regulator (inhibitor), ATP1A3, Fatty acid-binding protein, ERK1/2, CRABP2, Syntaxin 16, Guanylate cyclase A (NPR1), GRB2, PEX19, HXK4, DDC, COG3, UBC, HBGA, SNX1, CED-6, NUP93, ANXA8L2, Dysbindin, Notch, UBB, Guanylate cyclase, IGHG1, NOTCH1 precursor, IL1RN, E-FABP, Adult hemoglobin, Rich1, APOBEC-1, MLCK
117	positive regulation of defense response	362	1.564E-04	4.276E-03	1.564E-04	4.276E-03	12	STAT5A, TIRAP (Mal), p38 MAPK, ERK1 (MAPK3), STAT5, RPS27A, Ubiquitin, Fatty acid-binding protein, ERK1/2, UBC, UBB, SAP
118	organophosphate biosynthetic process	538	1.639E-04	4.407E-03	1.639E-04	4.407E-03	15	MIBP, GDP-mannose 4,6 dehydratase, UCK1, IMD2, ATP1A3, Fatty acid-binding protein, ADSL, Guanylate cyclase A (NPR1), MOCS2(small), Ajuba, RIFK, PAP41, Guanylate cyclase, CTP synthase II, E-FABP
119	bleb assembly	12	1.644E-04	4.407E-03	1.644E-04	4.407E-03	3	MYLK1, Telokin, MLCK
120	JAK-STAT cascade involved in growth hormone signaling pathway	30	1.653E	4.407E	1.653E	4.407E	4	STAT5A, ERK1 (MAPK3), STAT5, ERK1/2

			-04	-03	-04	-03		
121	negative regulation of epidermal growth factor receptor signaling pathway	56	1.728E-04	4.570E-03	1.728E-04	4.570E-03	5	RPS27A, Ubiquitin, GRB2, UBC, UBB
122	enzyme linked receptor protein signaling pathway	1007	1.826E-04	4.789E-03	1.826E-04	4.789E-03	22	Reticulon 4, CRK, STAT5A, p38 MAPK, ERK1 (MAPK3), Bim, STAT5, SMIF, RPS27A, p38gamma (MAPK12), Ubiquitin, Phosphatase regulator (inhibitor), ERK1/2, Guanylate cyclase A (NPR1), CrkL, GRB2, PTP-2, CNK1, UBC, UBB, Guanylate cyclase, 4E-BP1
123	negative regulation of ERBB signaling pathway	57	1.880E-04	4.891E-03	1.880E-04	4.891E-03	5	RPS27A, Ubiquitin, GRB2, UBC, UBB
124	phosphorus metabolic process	2922	1.945E-04	5.019E-03	1.945E-04	5.019E-03	46	MST3, MIBP, GDP-mannose 4,6 dehydratase, CRK, STAT5A, TIRAP (Mal), p38 MAPK, ERK1 (MAPK3), CERT, STAT5, Twinfilin, UCK1, RPS27A, p38gamma (MAPK12), Ubiquitin, IMD2, EYA1, NANP, DUPD1, Phosphatase regulator (inhibitor), PDXP, ATP1A3, Fatty acid-binding protein, ERK1/2, ADSL, MYLK1, Guanylate cyclase A (NPR1), MOCS2(small), CrkL, Ajuba, PTP-2, CNK1, HXK4, RIFK, UBC, Telokin, GBP5, UBB, PAP41, Guanylate cyclase, GCK(MAP4K2), RRAD, CTP synthase II, E-FABP, MLCK, Galectin-13
125	regulation of MAP kinase activity	371	1.962E-	5.021E-	1.962E-	5.021E-	12	CRK, TIRAP (Mal), p38 MAPK, ERK1 (MAPK3), RPS27A, Ubiquitin, ERK1/2, Ajuba, UBC, UBB, GCK(MAP4K2), IL1RN

			04	03	04	03		
126	negative regulation of transmembrane receptor protein serine/threonine kinase signaling pathway	129	2.091E-04	5.306E-03	2.091E-04	5.306E-03	7	RPS27A, Ubiquitin, Phosphatase regulator (inhibitor), UBC, Notch, UBB, NOTCH1 precursor
127	cellular response to DNA damage stimulus	810	2.120E-04	5.306E-03	2.120E-04	5.306E-03	19	TIPIN, RAD1, p38 MAPK, ERK1 (MAPK3), Bim, RPS27A, p38gamma (MAPK12), Ubiquitin, EYA1, XPF, Phosphatase regulator (inhibitor), ERK1/2, GRB2, UBC, RAD23A, UBB, DBC1, RPA4, DSS1
128	ventricular trabecula myocardium morphogenesis	13	2.123E-04	5.306E-03	2.123E-04	5.306E-03	3	RBP-J kappa (CBF1), Notch, NOTCH1 precursor
129	cell cycle process	1237	2.176E-04	5.396E-03	2.176E-04	5.396E-03	25	DLC1 (Dynein LC8a), TIPIN, DCTN2, PPP1R12B, RAD1, p38 MAPK, RPS27A, p38gamma (MAPK12), Ubiquitin, EYA1, SNX9, XPF, Phosphatase regulator (inhibitor), Fatty acid-binding protein, ARHGEF10, Kizuna (C20orf19), Ajuba, ZNF655, UBC, NUP93, Notch, UBB, 4E-BP1, RPA4, DYNLL
130	regulation of striated muscle cell differentiation	132	2.409E-04	5.928E-03	2.409E-04	5.928E-03	7	LAGY, MIBP, p38 MAPK, RBP-J kappa (CBF1), Notch, NOTCH1 precursor, MLCK

131	DNA damage checkpoint	176	2.510E-04	6.007E-03	2.510E-04	6.007E-03	8	TIPIN, RAD1, p38 MAPK, RPS27A, Ubiquitin, UBC, UBB, RPA4
132	negative regulation of protein refolding	3	2.573E-04	6.007E-03	2.573E-04	6.007E-03	2	PhLP, ST13 (Hip)
133	stress response to copper ion	3	2.573E-04	6.007E-03	2.573E-04	6.007E-03	2	Metallothionein-I, Metallothionein-II
134	detoxification of copper ion	3	2.573E-04	6.007E-03	2.573E-04	6.007E-03	2	Metallothionein-I, Metallothionein-II
135	regulation of protein refolding	3	2.573E-04	6.007E-03	2.573E-04	6.007E-03	2	PhLP, ST13 (Hip)
13	coronary vein morphogenesis	3	2.	6.	2.	6.	2	Notch, NOTCH1 precursor

6			573E-04	007E-03	573E-04	007E-03		
137	regulation of extracellular matrix assembly	3	2.573E-04	6.007E-03	2.573E-04	6.007E-03	2	Notch, NOTCH1 precursor
138	keratinocyte differentiation	134	2.642E-04	6.123E-03	2.642E-04	6.123E-03	7	SPRR1A, RBP-J kappa (CBF1), SPRR4, Notch, NOTCH1 precursor, Sprr2f, SPRR3
139	endocardial cushion formation	14	2.684E-04	6.132E-03	2.684E-04	6.132E-03	3	RBP-J kappa (CBF1), Notch, NOTCH1 precursor
140	endocardium development	14	2.684E-04	6.132E-03	2.684E-04	6.132E-03	3	RBP-J kappa (CBF1), Notch, NOTCH1 precursor
141	ribonucleotide biosynthetic process	179	2.81	6.38	2.81	6.38	8	UCK1, IMD2, ATP1A3, ADSL, Guanylate cyclase A (NPR1), RIFK, Guanylate cyclase, CTP synthase II

			4E-04	3E-03	4E-04	3E-03		
142	growth hormone receptor signaling pathway	35	3.046E-04	6.719E-03	3.046E-04	6.719E-03	4	STAT5A, ERK1 (MAPK3), STAT5, ERK1/2
143	mRNA stabilization	35	3.046E-04	6.719E-03	3.046E-04	6.719E-03	4	TIRAP (Mal), p38 MAPK, PAIP1, APOBEC-1
144	RNA stabilization	35	3.046E-04	6.719E-03	3.046E-04	6.719E-03	4	TIRAP (Mal), p38 MAPK, PAIP1, APOBEC-1
145	epithelial cell differentiation involved in prostate gland development	35	3.046E-04	6.719E-03	3.046E-04	6.719E-03	4	STAT5A, STAT5, Notch, NOTCH1 precursor
146	pattern recognition receptor signaling pathway	182	3.147E	6.895E	3.147E	6.895E	8	TIRAP (Mal), p38 MAPK, ERK1 (MAPK3), RPS27A, Ubiquitin, ERK1/2, UBC, UBB

			-04	-03	-04	-03		
147	Notch signaling pathway	183	3.265E-04	7.105E-03	3.265E-04	7.105E-03	8	GDP-mannose 4,6 dehydratase, RBP-J kappa (CBF1), RPS27A, Ubiquitin, UBC, Notch, UBB, NOTCH1 precursor
148	regulation of transcription from RNA polymerase II promoter involved in heart development	15	3.332E-04	7.201E-03	3.332E-04	7.201E-03	3	RBP-J kappa (CBF1), Notch, NOTCH1 precursor
149	ribose phosphate biosynthetic process	184	3.387E-04	7.271E-03	3.387E-04	7.271E-03	8	UCK1, IMD2, ATP1A3, ADSL, Guanylate cyclase A (NPR1), RIFK, Guanylate cyclase, CTP synthase II
150	macromolecular complex subunit organization	1823	3.559E-04	7.591E-03	3.559E-04	7.591E-03	32	HBG, DCTN2, NASP, ERK1 (MAPK3), RPS27A, Ccdc109a, IMD2, PhLP, SNX9, Kinesin light chain, Phosphatase regulator (inhibitor), PDXP, ERK1/2, ADSL, ARHGEF10, MYLK1, PQBP-1, Kizuna (C20orf19), LCKBP1, GA17, GRB2, Rap1GDS1, Tropomodulin, HBGA, Telokin, ST13 (Hip), NUP93, Occludin, Adult hemoglobin, Rich1, RIP, MLCK
151	innate immune response-activating signal transduction	186	3.641E-0	7.714E-0	3.641E-0	7.714E-0	8	TIRAP (Mal), p38 MAPK, ERK1 (MAPK3), RPS27A, Ubiquitin, ERK1/2, UBC, UBB

			4	3	4	3		
152	regulation of ERBB signaling pathway	101	3.674E-04	7.732E-03	3.674E-04	7.732E-03	6	RBP-J kappa (CBF1), RPS27A, Ubiquitin, GRB2, UBC, UBB
153	cellular response to organic substance	2404	3.707E-04	7.751E-03	3.707E-04	7.751E-03	39	Reticulon 4, CRK, STAT5A, TIRAP (Mal), p38 MAPK, ERK1 (MAPK3), Bim, Metallothionein-I, STAT5, SMIF, RPS27A, p38gamma (MAPK12), Ubiquitin, IMD2, Phosphatase regulator (inhibitor), PDXP, ERK1/2, Metallothionein-IG, LCKBP1, CrkL, GRB2, HXK4, DDC, Metallothionein-II, HMBS, UBC, GBP5, NUP93, Notch, UBB, Guanylate cyclase, NOTCH1 precursor, 4E-BP1, IL1RN, WWOX, PYDC1 (POP1), APOBEC-1, MLCK, PAF1
154	DNA integrity checkpoint	187	3.774E-04	7.840E-03	3.774E-04	7.840E-03	8	TIPIN, RAD1, p38 MAPK, RPS27A, Ubiquitin, UBC, UBB, RPA4
155	cardioblast differentiation	16	4.072E-04	8.373E-03	4.072E-04	8.373E-03	3	RBP-J kappa (CBF1), Notch, NOTCH1 precursor
156	activation of protein kinase activity	345	4.083E-04	8.373E-03	4.083E-04	8.373E-03	11	CRK, TIRAP (Mal), p38 MAPK, ERK1 (MAPK3), RPS27A, Ubiquitin, ERK1/2, CrkL, UBC, UBB, GCK(MAP4K2)

157	muscle tissue morphogenesis	104	4.299E-04	8.759E-03	4.299E-04	8.759E-03	6	RBP-J kappa (CBF1), MYLK1, Telokin, Notch, NOTCH1 precursor, MLCK
158	regulation of innate immune response	348	4.390E-04	8.889E-03	4.390E-04	8.889E-03	11	STAT5A, TIRAP (Mal), p38 MAPK, ERK1 (MAPK3), STAT5, RPS27A, Ubiquitin, ERK1/2, UBC, UBB, SAP
159	transmembrane receptor protein tyrosine kinase signaling pathway	725	4.614E-04	9.234E-03	4.614E-04	9.234E-03	17	Reticulon 4, CRK, STAT5A, p38 MAPK, ERK1 (MAPK3), Bim, STAT5, RPS27A, p38gamma (MAPK12), Ubiquitin, ERK1/2, CrkL, GRB2, CNK1, UBC, UBB, 4E-BP1
160	prostate gland epithelium morphogenesis	39	4.647E-04	9.234E-03	4.647E-04	9.234E-03	4	STAT5A, STAT5, Notch, NOTCH1 precursor
161	nucleotide-binding oligomerization domain containing signaling pathway	39	4.647E-04	9.234E-03	4.647E-04	9.234E-03	4	RPS27A, Ubiquitin, UBC, UBB
16	interleukin-1-mediated signaling pathway	17	4.	9.	4.	9.	3	TIRAP (Mal), ERK1 (MAPK3), ERK1/2

2			911E-04	541E-03	911E-04	541E-03		
163	single-organism intracellular transport	1231	4.927E-04	9.541E-03	4.927E-04	9.541E-03	24	Annexin VIII, PEX2, KLC2, VAMP5, ERK1 (MAPK3), Bim, CERT, KLC3, RPS27A, Ccdc109a, Ubiquitin, CDC4L, SNX9, Kinesin light chain, Phosphatase regulator (inhibitor), ERK1/2, Syntaxin 16, PEX19, COG3, UBC, SNX1, ANXA8L2, Dysbindin, UBB
164	atrioventricular node development	4	5.114E-04	9.541E-03	5.114E-04	9.541E-03	2	Notch, NOTCH1 precursor
165	negative regulation of pro-B cell differentiation	4	5.114E-04	9.541E-03	5.114E-04	9.541E-03	2	Notch, NOTCH1 precursor
166	cardiac right atrium morphogenesis	4	5.114E-04	9.541E-03	5.114E-04	9.541E-03	2	Notch, NOTCH1 precursor
167	cell migration involved in endocardial cushion formation	4	5.11	9.54	5.11	9.54	2	Notch, NOTCH1 precursor

			4E-04	1E-03	4E-04	1E-03		
168	regulation of mast cell differentiation	4	5.114E-04	9.541E-03	5.114E-04	9.541E-03	2	STAT5A, STAT5
169	prolactin signaling pathway	4	5.114E-04	9.541E-03	5.114E-04	9.541E-03	2	STAT5A, STAT5
170	negative regulation of endothelial cell chemotaxis	4	5.114E-04	9.541E-03	5.114E-04	9.541E-03	2	Notch, NOTCH1 precursor
171	cellular response to growth hormone stimulus	40	5.126E-04	9.541E-03	5.126E-04	9.541E-03	4	STAT5A, ERK1 (MAPK3), STAT5, ERK1/2
172	regulation of primary metabolic process	6540	5.130E	9.541E	5.130E	9.541E	83	Annexin VIII, PEX2, LAGY, DLC1 (Dynein LC8a), LCORL, TIPIN, SCMH1, CRK, HBG, STAT5A, TIRAP (Mal), GGA1, p38 MAPK, ERK1 (MAPK3), Bim, STAT5, RBP-J kappa (CBF1), FERD3L, SMIF, Twinfilin, BMAL2, Autophagin-1, RPS27A, p38gamma (MAPK12), ZNF397, Ubiquitin, EYA1, PhLP, SNX9, XPF, Phosphatase regulator (inhibitor), Fatty

			-04	-03	-04	-03		acid-binding protein, ERK1/2, NIP2, ARHGEF10, CRABP2, PQBP-1, LCKBP1, Guanylate cyclase A (NPR1), GA17, CrkL, Ajuba, HXK4, Rap1GDS1, ZNF655, UBC, HBGA, SNX1, PCP2, ST13 (Hip), HKR1, ANXA8L2, Notch, RAD23A, UBB, IRX4, Guanylate cyclase, ARID4A, Cyclophilin E, NOTCH1 precursor, GCK(MAP4K2), 4E-BP1, SCML1, DBC1, USP5, AO7, GGA, IL1RN, FAM116A, ZNF397OS, DYNLL, Cystatin B, WWOX, DKK3, PYDC1 (POP1), Adult hemoglobin, PAIP1, MLX, CAMK2N1, Rich1, APOBEC-1, RIP, PAF1
173	mitotic cell cycle phase transition	413	5.162E-04	9.545E-03	5.162E-04	9.545E-03	12	DLC1 (Dynein LC8a), DCTN2, PPP1R12B, RPS27A, Ubiquitin, Phosphatase regulator (inhibitor), Ajuba, UBC, UBB, 4E-BP1, RPA4, DYNLL
174	activation of innate immune response	197	5.334E-04	9.806E-03	5.334E-04	9.806E-03	8	TIRAP (Mal), p38 MAPK, ERK1 (MAPK3), RPS27A, Ubiquitin, ERK1/2, UBC, UBB
175	antigen processing and presentation of exogenous peptide antigen	248	5.475E-04	1.001E-02	5.475E-04	1.001E-02	9	KLC2, DLC1 (Dynein LC8a), DCTN2, RPS27A, Ubiquitin, Kinesin light chain, UBC, UBB, DYNLL
176	immune response-activating cell surface receptor signaling pathway	302	5.572E-04	1.002E-02	5.572E-04	1.002E-02	10	CRK, ERK1 (MAPK3), RPS27A, Ubiquitin, ERK1/2, GRB2, UBC, UBB, IGHG1, ACP33
17	response to abiotic stimulus	15	5.	1.	5.	1.	28	TIPIN, RAD1, p38 MAPK, ERK1 (MAPK3), STAT5, RBP1, RPS27A, Ubiquitin, EYA1, XPF, ATP1A3, Fatty acid-binding

7		51	603E-04	002E-02	603E-04	002E-02		protein, ERK1/2, ADSL, MYLK1, Ajuba, GRB2, HMBS, UBC, Telokin, PCP2, Notch, UBB, Guanylate cyclase, NOTCH1 precursor, DBC1, APOBEC-1, MLCK
178	positive regulation of protein insertion into mitochondrial membrane involved in apoptotic signaling pathway	41	5.640E-04	1.002E-02	5.640E-04	1.002E-02	4	DLC1 (Dynein LC8a), Bim, BMF, DYNLL
179	regulation of protein insertion into mitochondrial membrane involved in apoptotic signaling pathway	41	5.640E-04	1.002E-02	5.640E-04	1.002E-02	4	DLC1 (Dynein LC8a), Bim, BMF, DYNLL
180	prostate gland morphogenesis	41	5.640E-04	1.002E-02	5.640E-04	1.002E-02	4	STAT5A, STAT5, Notch, NOTCH1 precursor
181	organ development	3691	5.669E-04	1.002E-02	5.669E-04	1.002E-02	53	LAGY, Reticulon 4, DLC1 (Dynein LC8a), Beta crystallin B2, VAMP5, STAT5A, SPRR1A, TIRAP (Mal), RAD1, p38 MAPK, ERK1 (MAPK3), Bim, Metallothionein-I, CERT, STAT5, RBP-J kappa (CBF1), NHS, p38gamma (MAPK12), Ubiquitin, IMD2, EYA1, Fatty acid-binding protein, ERK1/2, MYLK1, CRABP2, Metallothionein-IG, LCKBP1, SPRR4, NEPH2, CrkL, GRB2, PTP-2, HXK4, HMBS, Telokin, Dysbindin, Notch, ACSBG1, UBB, IRX4, ARID4A, NOTCH1 precursor, 4E-BP1, Sprr2f, BMP1, DYNLL, WWOX, E-FABP, DKK3, Adult hemoglobin, Melusin, SPRR3, MLCK
182	cellular polysaccharide metabolic process	110	5.7	1.0	5.7	1.0	6	RPS27A, Ubiquitin, Phosphatase regulator (inhibitor), HXK4, UBC, UBB

			95E-04	19E-02	95E-04	19E-02		
183	negative regulation of myoblast differentiation	18	5.853E-04	1.023E-02	5.853E-04	1.023E-02	3	MIBP, Notch, NOTCH1 precursor
184	single-organism organelle organization	1878	5.958E-04	1.032E-02	5.958E-04	1.032E-02	32	PEX2, LAGY, DLC1 (Dynein LC8a), TIPIN, DCTN2, VAMP5, Bim, CERT, Ubiquitin, EYA1, SNX9, XPF, Phosphatase regulator (inhibitor), PDXP, Fatty acid-binding protein, NIP2, ARHGEF10, MYLK1, Kizuna (C20orf19), LCKBP1, PEX19, Rap1GDS1, Tropomodulin, Telokin, Dysbindin, UBB, ARID4A, DYNLL, Rich1, RIP, MLCK, PAF1
185	hexose metabolic process	251	5.969E-04	1.032E-02	5.969E-04	1.032E-02	9	p38 MAPK, RPS27A, Ubiquitin, Phosphatase regulator (inhibitor), Fatty acid-binding protein, HXK4, UBC, UBB, E-FABP
186	negative regulation of cellular process	4254	6.073E-04	1.041E-02	6.073E-04	1.041E-02	59	MST3, PEX2, LAGY, Reticulon 4, MIBP, DLC1 (Dynein LC8a), TIPIN, SCMH1, HBG, STAT5A, TCL1A, p38 MAPK, IL-1F5, Metallothionein-I, STAT5, RBP-J kappa (CBF1), FERD3L, Twinfilin, RPS27A, p38gamma (MAPK12), Ubiquitin, EYA1, PhLP, XPF, Phosphatase regulator (inhibitor), LYPLAL1, Fatty acid-binding protein, ERK1/2, NIP2, LCKBP1, Guanylate cyclase A (NPR1), Ajuba, GRB2, PTP-2, HXK4, ZNF655, UBC, HBGA, ST13 (Hip), Notch, UBB, BMF, Guanylate cyclase, ARID4A, NOTCH1 precursor, 4E-BP1, RRAD, DBC1, IL1RN, DYNLL, Cystatin B, WWOX, DKK3, PYDC1 (POP1), Adult hemoglobin, MLX, CAMK2N1, APOBEC-1, PAF1
18	Ras protein signal transduction	20	6.	1.	6.	1.	8	p38 MAPK, ERK1 (MAPK3), p38gamma (MAPK12), ERK1/2, CrkL, GRB2, CNK1, DOK3

7		1	088E-04	041E-02	088E-04	041E-02		
188	muscle organ morphogenesis	112	6.375E-04	1.085E-02	6.375E-04	1.085E-02	6	RBP-J kappa (CBF1), MYLK1, Telokin, Notch, NOTCH1 precursor, MLCK
189	regulation of cellular response to stress	485	6.475E-04	1.096E-02	6.475E-04	1.096E-02	13	MST3, Reticulon 4, CRK, TIRAP (Mal), ERK1 (MAPK3), EYA1, SCOCO, ERK1/2, Ajuba, GCK(MAP4K2), DBC1, IL1RN, Adult hemoglobin
190	regulation of myoblast differentiation	43	6.774E-04	1.133E-02	6.774E-04	1.133E-02	4	MIBP, p38 MAPK, Notch, NOTCH1 precursor
191	positive regulation of signal transduction	1336	6.801E-04	1.133E-02	6.801E-04	1.133E-02	25	CRK, TIRAP (Mal), p38 MAPK, ERK1 (MAPK3), Bim, LASP1, RBP-J kappa (CBF1), RPS27A, Ubiquitin, EYA1, ERK1/2, NIP2, LCKBP1, CrkL, Ajuba, GRB2, UBC, Notch, UBB, BMF, NOTCH1 precursor, GCK(MAP4K2), IL1RN, WWOX, SAP
192	positive regulation of catabolic process	256	6.87	1.13	6.87	1.13	9	GGA1, Autophagin-1, SCOCO, Fatty acid-binding protein, ARHGEF10, HXK4, SNX1, USP5, GGA

			1E-04	3E-02	1E-04	3E-02		
193	tonic smooth muscle contraction	19	6.903E-04	1.133E-02	6.903E-04	1.133E-02	3	MYLK1, Telokin, MLCK
194	positive regulation of protein oligomerization	19	6.903E-04	1.133E-02	6.903E-04	1.133E-02	3	Bim, SNX9, BMF
195	endocardial cushion morphogenesis	19	6.903E-04	1.133E-02	6.903E-04	1.133E-02	3	RBP-J kappa (CBF1), Notch, NOTCH1 precursor
196	I-kappaB kinase/NF-kappaB signaling	76	7.195E-04	1.174E-02	7.195E-04	1.174E-02	5	TIRAP (Mal), RPS27A, Ubiquitin, UBC, UBB
197	antigen processing and presentation of exogenous antigen	258	7.262E	1.179E	7.262E	1.179E	9	KLC2, DLC1 (Dynein LC8a), DCTN2, RPS27A, Ubiquitin, Kinesin light chain, UBC, UBB, DYNLL

			-04	-02	-04	-02	
198	anatomical structure development	5718	7.305E-04	1.180E-02	7.305E-04	1.180E-02	74	MST3, Neurochondrin, PEX2, LAGY, Reticulon 4, DLC1 (Dynein LC8a), SCMH1, Beta crystallin B2, VAMP5, STAT5A, SPRR1A, TIRAP (Mal), RAD1, TCL1A, p38 MAPK, NASP, ERK1 (MAPK3), Bim, Metallothionein-I, CERT, STAT5, EFHD1, RBP-J kappa (CBF1), FERD3L, NHS, p38gamma (MAPK12), Ubiquitin, IMD2, EYA1, Phosphatase regulator (inhibitor), Fatty acid-binding protein, ERK1/2, NIP2, ARHGEF10, MYLK1, CRABP2, Metallothionein-IG, PQBP-1, LCKBP1, SPRR4, NEPH2, CrkL, Ajuba, GRB2, PTP-2, HXK4, BBS9, Metallothionein-II, Tropomodulin, HMBS, Telokin, Dysbindin, Notch, ACSBG1, UBB, PAP41, IRX4, Guanylate cyclase, ARID4A, NOTCH1 precursor, 4E-BP1, Sprr2f, SCML1, BMP1, DYNLL, WWOX, E-FABP, DKK3, Adult hemoglobin, Melusin, RIP, SPRR3, MLCK, PAF1
199	positive regulation of cell communication	1424	7.594E-04	1.221E-02	7.594E-04	1.221E-02	26	CRK, TIRAP (Mal), p38 MAPK, ERK1 (MAPK3), Bim, LASP1, RBP-J kappa (CBF1), RPS27A, Ubiquitin, EYA1, SCOCO, ERK1/2, NIP2, LCKBP1, CrkL, Ajuba, GRB2, UBC, Notch, UBB, BMF, NOTCH1 precursor, GCK(MAP4K2), IL1RN, WWOX, SAP
200	negative regulation of biological process	4668	7.645E-04	1.221E-02	7.645E-04	1.221E-02	63	MST3, PEX2, LAGY, Reticulon 4, MIBP, DLC1 (Dynein LC8a), TIPIN, SCMH1, HBG, STAT5A, TIRAP (Mal), TCL1A, p38 MAPK, IL-1F5, Metallothionein-I, STAT5, RBP-J kappa (CBF1), FERD3L, Twinfilin, RPS27A, p38gamma (MAPK12), Ubiquitin, EYA1, PhLP, XPF, Phosphatase regulator (inhibitor), LYPLAL1, Fatty acid-binding protein, ERK1/2, NIP2, Metallothionein-IG, LCKBP1, Guanylate cyclase A (NPR1), Ajuba, GRB2, PTP-2, HXK4, Metallothionein-II, ZNF655, UBC, HBGA, ST13 (Hip), Notch, UBB, BMF, Guanylate cyclase, ARID4A, NOTCH1 precursor, 4E-BP1, RRAD, DBC1, Metallothionein-1M, IL1RN, DYNLL, Cystatin B, WWOX, DKK3, PYDC1 (POP1), Adult hemoglobin, MLX, CAMK2N1, APOBEC-1, PAF1
201	positive regulation of MAPK cascade	494	7.671E-0	1.221E-0	7.671E-0	1.221E-0	13	CRK, TIRAP (Mal), p38 MAPK, ERK1 (MAPK3), RPS27A, Ubiquitin, ERK1/2, NIP2, Ajuba, UBC, UBB, GCK(MAP4K2), IL1RN

			4	2	4	2		
202	positive regulation of biological process	5249	7.985E-04	1.265E-02	7.985E-04	1.265E-02	69	MST3, LAGY, Reticulon 4, DLC1 (Dynein LC8a), TIPIN, CRK, Calpain 1(mu), STAT5A, TIRAP (Mal), GGA1, p38 MAPK, ERK1 (MAPK3), Bim, LASP1, STAT5, RBP-J kappa (CBF1), SMIF, BMAL2, Autophagin-1, RPS27A, p38gamma (MAPK12), Ccdc109a, Ubiquitin, EYA1, SNX9, SCOCO, PDXP, Fatty acid-binding protein, ERK1/2, NIP2, ARHGEF10, MYLK1, LCKBP1, Guanylate cyclase A (NPR1), CrkL, Ajuba, GRB2, HXK4, UBC, Telokin, SNX1, Dysbindin, Notch, RAD23A, UBB, BMF, Guanylate cyclase, IGHG1, Cyclophilin E, NOTCH1 precursor, GCK(MAP4K2), 4E-BP1, DBC1, USP5, GGA, IL1RN, FAM116A, BMP1, DYNLL, WWOX, ACP33, PYDC1 (POP1), Adult hemoglobin, PAIP1, MLX, SAP, APOBEC-1, MLCK, PAF1
203	oxygen transport	20	8.066E-04	1.265E-02	8.066E-04	1.265E-02	3	HBG, HBGA, Adult hemoglobin
204	response to muramyl dipeptide	20	8.066E-04	1.265E-02	8.066E-04	1.265E-02	3	p38 MAPK, Notch, NOTCH1 precursor
205	creatinine metabolic process	5	8.470E-04	1.266E-02	8.470E-04	1.266E-02	2	STAT5A, STAT5
206	response to erythropoietin	5	8.470	1.266	8.470	1.266	2	Metallothionein-I, Metallothionein-II

			E-04	E-02	E-04	E-02		
207	cellular response to erythropoietin	5	8.470E-04	1.266E-02	8.470E-04	1.266E-02	2	Metallothionein-I, Metallothionein-II
208	hypothalamus gonadotrophin-releasing hormone neuron differentiation	5	8.470E-04	1.266E-02	8.470E-04	1.266E-02	2	Ubiquitin, UBB
209	stress response to metal ion	5	8.470E-04	1.266E-02	8.470E-04	1.266E-02	2	Metallothionein-I, Metallothionein-II
210	negative regulation of photoreceptor cell differentiation	5	8.470E-04	1.266E-02	8.470E-04	1.266E-02	2	Notch, NOTCH1 precursor
211	hypothalamus gonadotrophin-releasing hormone neuron development	5	8.470E-	1.266E-	8.470E-	1.266E-	2	Ubiquitin, UBB

			04	02	04	02		
212	development of secondary male sexual characteristics	5	8.470E-04	1.266E-02	8.470E-04	1.266E-02	2	STAT5A, STAT5
213	mitral valve formation	5	8.470E-04	1.266E-02	8.470E-04	1.266E-02	2	Notch, NOTCH1 precursor
214	isoleucine metabolic process	5	8.470E-04	1.266E-02	8.470E-04	1.266E-02	2	STAT5A, STAT5
215	protein complex subunit organization	1596	8.725E-04	1.298E-02	8.725E-04	1.298E-02	28	HBG, DCTN2, NASP, ERK1 (MAPK3), RPS27A, Ccdc109a, IMD2, PhLP, Phosphatase regulator (inhibitor), PDXP, ERK1/2, ADSL, ARHGEF10, MYLK1, Kizuna (C20orf19), LCKBP1, GRB2, Rap1GDS1, Tropomodulin, HBGA, Telokin, ST13 (Hip), NUP93, Occludin, Adult hemoglobin, Rich1, RIP, MLCK
216	cytoplasmic pattern recognition receptor signaling pathway	46	8.764E-04	1.298E-02	8.764E-04	1.298E-02	4	RPS27A, Ubiquitin, UBC, UBB

217	cellular response to hypoxia	165	9.183E-04	1.354E-02	9.183E-04	1.354E-02	7	RPS27A, Ubiquitin, Fatty acid-binding protein, UBC, Notch, UBB, NOTCH1 precursor
218	cellular biosynthetic process	4991	9.448E-04	1.378E-02	9.448E-04	1.378E-02	66	PEX2, MUC15, LAGY, MIBP, DLC1 (Dynein LC8a), LCORL, GDP-mannose 4,6 dehydratase, TIPIN, SCMH1, STAT5A, RAD1, p38 MAPK, NASP, ERK1 (MAPK3), CERT, STAT5, RBP-J kappa (CBF1), FERD3L, RBP1, BMAL2, UCK1, RPS27A, p38gamma (MAPK12), ZNF397, Ubiquitin, IMD2, EYA1, NANP, Phosphatase regulator (inhibitor), ATP1A3, Fatty acid-binding protein, ERK1/2, ADSL, CRABP2, PQBP-1, Guanylate cyclase A (NPR1), MOCS2(small), GA17, Ajuba, HXK4, DDC, RIFK, ZNF655, COG3, HMBS, UBC, C16orf75, HKR1, Notch, UBB, PAP41, Guanylate cyclase, ARID4A, NOTCH1 precursor, 4E-BP1, SCML1, GINS2, CTP synthase II, RPA4, ZNF397OS, DYNLL, E-FABP, PAIP1, MLX, APOBEC-1, PAF1
219	organic substance biosynthetic process	5087	9.451E-04	1.378E-02	9.451E-04	1.378E-02	67	PEX2, MUC15, LAGY, MIBP, DLC1 (Dynein LC8a), LCORL, GDP-mannose 4,6 dehydratase, TIPIN, SCMH1, STAT5A, RAD1, p38 MAPK, NASP, ERK1 (MAPK3), CERT, STAT5, RBP-J kappa (CBF1), FERD3L, RBP1, BMAL2, UCK1, RPS27A, p38gamma (MAPK12), ZNF397, Ubiquitin, IMD2, EYA1, NANP, Phosphatase regulator (inhibitor), ATP1A3, Fatty acid-binding protein, ERK1/2, ADSL, CRABP2, PQBP-1, Guanylate cyclase A (NPR1), MOCS2(small), GA17, Ajuba, HXK4, DDC, RIFK, ZNF655, COG3, HMBS, UBC, C16orf75, HKR1, Notch, UBB, PAP41, Guanylate cyclase, ARID4A, NOTCH1 precursor, 4E-BP1, SCML1, GINS2, CTP synthase II, RPA4, ZNF397OS, DYNLL, E-FABP, GOCAP1, PAIP1, MLX, APOBEC-1, PAF1
220	negative regulation of type I interferon production	47	9.510E-04	1.378E-02	9.510E-04	1.378E-02	4	RPS27A, Ubiquitin, UBC, UBB
221	polysaccharide metabolic process	121	9.56	1.37	9.56	1.37	6	RPS27A, Ubiquitin, Phosphatase regulator (inhibitor), HXK4, UBC, UBB

			0E-04	8E-02	0E-04	8E-02		
222	immune system process	2697	9.562E-04	1.378E-02	9.562E-04	1.378E-02	41	KLC2, DLC1 (Dynein LC8a), CRK, DCTN2, STAT5A, TIRAP (Mal), p38 MAPK, ERK1 (MAPK3), Bim, Metallothionein-I, CERT, STAT5, RBP-J kappa (CBF1), RPS27A, Ubiquitin, IMD2, KAAG1, Kinesin light chain, ERK1/2, Metallothionein-IG, LCKBP1, NEPH2, CrkL, GRB2, Metallothionein-II, UBC, GBP5, Notch, UBB, IGHG1, ARID4A, CALCOCO2, NOTCH1 precursor, GCK(MAP4K2), IL1RN, DYNLL, ACP33, PYDC1 (POP1), Adult hemoglobin, SAP, APOBEC-1
223	cellular response to decreased oxygen levels	167	9.853E-04	1.406E-02	9.853E-04	1.406E-02	7	RPS27A, Ubiquitin, Fatty acid-binding protein, UBC, Notch, UBB, NOTCH1 precursor
224	response to wounding	1450	9.882E-04	1.406E-02	9.882E-04	1.406E-02	26	Annexin VIII, KLC2, CRK, HBG, TIRAP (Mal), p38 MAPK, ERK1 (MAPK3), STAT5, RBP-J kappa (CBF1), Kinesin light chain, Fatty acid-binding protein, ERK1/2, Ajuba, GRB2, HBGA, SAAL1, ANXA8L2, Dysbindin, Notch, Guanylate cyclase, NOTCH1 precursor, IL1RN, E-FABP, Adult hemoglobin, SPRR3, MLCK
225	immune response-activating signal transduction	445	9.888E-04	1.406E-02	9.888E-04	1.406E-02	12	CRK, TIRAP (Mal), p38 MAPK, ERK1 (MAPK3), RPS27A, Ubiquitin, ERK1/2, GRB2, UBC, UBB, IGHG1, ACP33
226	mitotic cell cycle process	850	1.026E	1.452E	1.026E	1.452E	18	DLC1 (Dynein LC8a), TIPIN, DCTN2, PPP1R12B, RPS27A, Ubiquitin, EYA1, SNX9, Phosphatase regulator (inhibitor), Fatty acid-binding protein, ARHGEF10, Ajuba, UBC, NUP93, UBB, 4E-BP1, RPA4, DYNLL

			-03	-02	-03	-02		
227	signal transduction in response to DNA damage	123	1.041E-03	1.467E-02	1.041E-03	1.467E-02	6	p38 MAPK, RPS27A, Ubiquitin, GRB2, UBC, UBB
228	organelle organization	2976	1.064E-03	1.493E-02	1.064E-03	1.493E-02	44	Annexin VIII, PEX2, LAGY, Reticulon 4, ZNF261, DLC1 (Dynein LC8a), TIPIN, SCMH1, DCTN2, VAMP5, NASP, Bim, CERT, Autophagin-1, Ubiquitin, EYA1, SNX9, XPF, Phosphatase regulator (inhibitor), PDXP, Fatty acid-binding protein, NIP2, ARHGEF10, MYLK1, PQBP-1, Kizuna (C20orf19), LCKBP1, PEX19, Rap1GDS1, BBS9, Tropomodulin, Telokin, NUP93, ANXA8L2, Dysbindin, UBB, ARID4A, DBC1, DYNLL, Rich1, p47, RIP, MLCK, PAF1
229	positive regulation of wound healing	22	1.075E-03	1.498E-02	1.075E-03	1.498E-02	3	MYLK1, Telokin, MLCK
230	protein transport	1380	1.077E-03	1.498E-02	1.077E-03	1.498E-02	25	PEX2, VAMP5, GGA1, NASP, ERK1 (MAPK3), RBP-J kappa (CBF1), Autophagin-1, RPS27A, SNX9, Kinesin light chain, Phosphatase regulator (inhibitor), ERK1/2, Syntaxin 16, Rab-9B, PEX19, BBS9, COG3, SNX1, NUP93, Notch, NOTCH1 precursor, GGA, RAB24, COG8, APOBEC-1
231	astrocyte differentiation	49	1.113E-0	1.542E-0	1.113E-0	1.542E-0	4	Metallothionein-II, HMBS, Notch, NOTCH1 precursor

			3	2	3	2		
232	wound healing	857	1.125E-03	1.551E-02	1.125E-03	1.551E-02	18	Annexin VIII, KLC2, CRK, HBG, p38 MAPK, ERK1 (MAPK3), Kinesin light chain, ERK1/2, Ajuba, GRB2, HBGA, ANXA8L2, Dysbindin, Notch, Guanylate cyclase, NOTCH1 precursor, Adult hemoglobin, SPRR3
233	mitotic cell cycle	930	1.134E-03	1.558E-02	1.134E-03	1.558E-02	19	DLC1 (Dynein LC8a), TIPIN, DCTN2, PPP1R12B, RPS27A, Ubiquitin, EYA1, SNX9, Phosphatase regulator (inhibitor), Fatty acid-binding protein, ARHGEF10, Ajuba, UBC, NUP93, UBB, 4E-BP1, GINS2, RPA4, DYNLL
234	antigen processing and presentation of peptide antigen	276	1.166E-03	1.594E-02	1.166E-03	1.594E-02	9	KLC2, DLC1 (Dynein LC8a), DCTN2, RPS27A, Ubiquitin, Kinesin light chain, UBC, UBB, DYNLL
235	positive regulation of organelle organization	394	1.213E-03	1.622E-02	1.213E-03	1.622E-02	11	DLC1 (Dynein LC8a), ERK1 (MAPK3), Bim, PDXP, ERK1/2, ARHGEF10, LCKBP1, GRB2, BMF, DYNLL, MLCK
236	regulation of MAPK cascade	722	1.226E-03	1.622E-02	1.226E-03	1.622E-02	16	CRK, TIRAP (Mal), p38 MAPK, ERK1 (MAPK3), RPS27A, Ubiquitin, ERK1/2, NIP2, CrkL, Ajuba, GRB2, UBC, UBB, GCK(MAP4K2), IL1RN, MLCK

237	mesenchyme morphogenesis	23	1.228E-03	1.622E-02	1.228E-03	1.622E-02	3	RBP-J kappa (CBF1), Notch, NOTCH1 precursor
238	multicellular organismal development	5723	1.256E-03	1.622E-02	1.256E-03	1.622E-02	73	MST3, Neurochondrin, PEX2, LAGY, Reticulon 4, ZNF261, DLC1 (Dynein LC8a), SCMH1, Beta crystallin B2, VAMP5, STAT5A, SPRR1A, TIRAP (Mal), RAD1, TCL1A, p38 MAPK, NASP, ERK1 (MAPK3), Bim, Metallothionein-I, CERT, STAT5, EFHD1, RBP-J kappa (CBF1), FERD3L, NHS, p38gamma (MAPK12), Ubiquitin, IMD2, EYA1, Phosphatase regulator (inhibitor), Fatty acid-binding protein, ERK1/2, NIP2, ARHGEF10, MYLK1, CRABP2, Metallothionein-IG, PQBP-1, LCKBP1, SPRR4, NEPH2, CrkL, GRB2, PTP-2, HXK4, DDC, Metallothionein-II, HMBS, Telokin, HKR1, Dysbindin, Notch, ACSBG1, UBB, PAP41, IRX4, Guanylate cyclase, ARID4A, NOTCH1 precursor, 4E-BP1, Sprr2f, BMP1, DYNLL, WWOX, E-FABP, DKK3, Adult hemoglobin, Melusin, RIP, SPRR3, MLCK, PAF1
239	regulation of transmembrane receptor protein serine/threonine kinase signaling pathway	225	1.262E-03	1.622E-02	1.262E-03	1.622E-02	8	RBP-J kappa (CBF1), RPS27A, Ubiquitin, Phosphatase regulator (inhibitor), UBC, Notch, UBB, NOTCH1 precursor
240	negative regulation of phospholipase A2 activity	6	1.263E-03	1.622E-02	1.263E-03	1.622E-02	2	Annexin VIII, ANXA8L2
241	osteoblast fate commitment	6	1.263E-	1.622E-	1.263E-	1.622E-	2	Notch, NOTCH1 precursor

			03	02	03	02		
242	regulation of Golgi inheritance	6	1.263E-03	1.622E-02	1.263E-03	1.622E-02	2	ERK1 (MAPK3), ERK1/2
243	MAPK import into nucleus	6	1.263E-03	1.622E-02	1.263E-03	1.622E-02	2	ERK1 (MAPK3), ERK1/2
244	positive regulation of transcription from RNA polymerase II promoter in response to hypoxia	6	1.263E-03	1.622E-02	1.263E-03	1.622E-02	2	Notch, NOTCH1 precursor
245	regulation of pro-B cell differentiation	6	1.263E-03	1.622E-02	1.263E-03	1.622E-02	2	Notch, NOTCH1 precursor
246	positive regulation of mast cell proliferation	6	1.263E-03	1.622E-02	1.263E-03	1.622E-02	2	STAT5A, STAT5

247	cellular lactam metabolic process	6	1.263E-03	1.622E-02	1.263E-03	1.622E-02	2	STAT5A, STAT5
248	regulation of mast cell proliferation	6	1.263E-03	1.622E-02	1.263E-03	1.622E-02	2	STAT5A, STAT5
249	negative regulation of mast cell apoptotic process	6	1.263E-03	1.622E-02	1.263E-03	1.622E-02	2	STAT5A, STAT5
250	negative regulation of transforming growth factor beta receptor signaling pathway	87	1.325E-03	1.696E-02	1.325E-03	1.696E-02	5	RPS27A, Ubiquitin, Phosphatase regulator (inhibitor), UBC, UBB
251	positive regulation of myeloid cell differentiation	129	1.331E-03	1.696E-02	1.331E-03	1.696E-02	6	STAT5A, p38 MAPK, STAT5, LCKBP1, Notch, Adult hemoglobin
25	muscle organ development	39	1.	1.	1.	1.	11	VAMP5, p38 MAPK, RBP-J kappa (CBF1), p38gamma (MAPK12), MYLK1, Telokin, Dysbindin, Notch, NOTCH1

2		9	341E-03	702E-02	341E-03	702E-02		precursor, Melusin, MLCK
253	positive regulation of cellular process	4675	1.362E-03	1.721E-02	1.362E-03	1.721E-02	62	MST3, LAGY, Reticulon 4, DLC1 (Dynein LC8a), TIPIN, CRK, Calpain 1(mu), STAT5A, TIRAP (Mal), p38 MAPK, ERK1 (MAPK3), Bim, LASP1, STAT5, RBP-J kappa (CBF1), SMIF, BMAL2, Autophagin-1, RPS27A, p38gamma (MAPK12), Ccdc109a, Ubiquitin, EYA1, SCOCO, PDXP, Fatty acid-binding protein, ERK1/2, NIP2, ARHGEF10, MYLK1, LCKBP1, Guanylate cyclase A (NPR1), CrkL, Ajuba, GRB2, HXK4, UBC, Telokin, Dysbindin, Notch, RAD23A, UBB, BMF, Guanylate cyclase, Cyclophilin E, NOTCH1 precursor, GCK(MAP4K2), 4E-BP1, DBC1, USP5, IL1RN, FAM116A, DYNLL, WWOX, PYDC1 (POP1), Adult hemoglobin, PAIP1, MLX, SAP, APOBEC-1, MLCK, PAF1
254	cytokine-mediated signaling pathway	463	1.385E-03	1.721E-02	1.385E-03	1.721E-02	12	STAT5A, TIRAP (Mal), ERK1 (MAPK3), STAT5, RPS27A, Ubiquitin, ERK1/2, Metallothionein-II, UBC, NUP93, UBB, PYDC1 (POP1)
255	inflammatory response to antigenic stimulus	52	1.392E-03	1.721E-02	1.392E-03	1.721E-02	4	RBP-J kappa (CBF1), Notch, NOTCH1 precursor, IL1RN
256	axon cargo transport	52	1.392E-03	1.721E-02	1.392E-03	1.721E-02	4	KLC2, KLC3, Kinesin light chain, Dysbindin

257	apoptotic process involved in morphogenesis	24	1.393E-03	1.721E-02	1.393E-03	1.721E-02	3	Bim, Notch, NOTCH1 precursor
258	cardiac left ventricle morphogenesis	24	1.393E-03	1.721E-02	1.393E-03	1.721E-02	3	RBP-J kappa (CBF1), Notch, NOTCH1 precursor
259	positive regulation of cardiac muscle cell proliferation	24	1.393E-03	1.721E-02	1.393E-03	1.721E-02	3	RBP-J kappa (CBF1), Notch, NOTCH1 precursor
260	regulation of cellular metabolic process	646	1.457E-03	1.793E-02	1.457E-03	1.793E-02	80	PEX2, LAGY, DLC1 (Dynein LC8a), LCORL, TIPIN, SCMH1, CRK, HBG, STAT5A, TIRAP (Mal), p38 MAPK, ERK1 (MAPK3), STAT5, RBP-J kappa (CBF1), FERD3L, SMIF, Twinfilin, BMAL2, Autophagin-1, RPS27A, p38gamma (MAPK12), ZNF397, Ubiquitin, EYA1, PhLP, SNX9, SCOCO, XPF, Phosphatase regulator (inhibitor), Fatty acid-binding protein, ERK1/2, NIP2, ARHGEF10, CRABP2, PQBP-1, LCKBP1, Guanylate cyclase A (NPR1), GA17, CrkL, Ajuba, GRB2, HXK4, Rap1GDS1, ZNF655, UBC, HBGA, PCP2, ST13 (Hip), HKR1, Notch, RAD23A, UBB, IRX4, Guanylate cyclase, ARID4A, Cyclophilin E, NOTCH1 precursor, GCK(MAP4K2), 4E-BP1, SCML1, DBC1, USP5, AO7, IL1RN, FAM116A, ZNF397OS, DYNLL, Cystatin B, WWOX, DKK3, PYDC1 (POP1), Adult hemoglobin, PAIP1, MLX, CAMK2N1, Rich1, APOBEC-1, RIP, MLCK, PAF1
261	regulation of skeletal muscle fiber development	53	1.495E	1.828E	1.495E	1.828E	4	MIBP, p38 MAPK, Notch, NOTCH1 precursor

			-03	-02	-03	-02		
262	actin filament-based process	532	1.497E-03	1.828E-02	1.497E-03	1.828E-02	13	DLC1 (Dynein LC8a), Phosphatase regulator (inhibitor), PDXP, MYLK1, LCKBP1, Rap1GDS1, Tropomodulin, Telokin, Troponin C, skeletal muscle, Dysbindin, DYNLL, Rich1, MLCK
263	negative regulation of cellular biosynthetic process	1494	1.514E-03	1.841E-02	1.514E-03	1.841E-02	26	PEX2, LAGY, DLC1 (Dynein LC8a), TIPIN, SCMH1, HBG, RBP-J kappa (CBF1), FERD3L, RPS27A, Ubiquitin, Fatty acid-binding protein, LCKBP1, Ajuba, HXK4, UBC, HBGA, Notch, UBB, ARID4A, NOTCH1 precursor, 4E-BP1, DYNLL, DKK3, Adult hemoglobin, MLX, PAF1
264	actin filament organization	180	1.520E-03	1.841E-02	1.520E-03	1.841E-02	7	Phosphatase regulator (inhibitor), PDXP, MYLK1, LCKBP1, Telokin, Rich1, MLCK
265	biosynthetic process	5178	1.542E-03	1.861E-02	1.542E-03	1.861E-02	67	PEX2, MUC15, LAGY, MIBP, DLC1 (Dynein LC8a), LCORL, GDP-mannose 4,6 dehydratase, TIPIN, SCMH1, STAT5A, RAD1, p38 MAPK, NASP, ERK1 (MAPK3), CERT, STAT5, RBP-J kappa (CBF1), FERD3L, RBP1, BMAL2, UCK1, RPS27A, p38gamma (MAPK12), ZNF397, Ubiquitin, IMD2, EYA1, NANP, Phosphatase regulator (inhibitor), ATP1A3, Fatty acid-binding protein, ERK1/2, ADSL, CRABP2, PQBP-1, Guanylate cyclase A (NPR1), MOCS2(small), GA17, Ajuba, HXK4, DDC, RIFK, ZNF655, COG3, HMBS, UBC, C16orf75, HKR1, Notch, UBB, PAP41, Guanylate cyclase, ARID4A, NOTCH1 precursor, 4E-BP1, SCML1, GINS2, CTP synthase II, RPA4, ZNF397OS, DYNLL, E-FABP, GOCAP1, PAIP1, MLX, APOBEC-1, PAF1
266	cell surface receptor signaling pathway involved in heart development	25	1.57	1.86	1.57	1.86	3	RBP-J kappa (CBF1), Notch, NOTCH1 precursor

			3E-03	8E-02	3E-03	8E-02		
267	gas transport	25	1.573E-03	1.868E-02	1.573E-03	1.868E-02	3	HBG, HBGA, Adult hemoglobin
268	positive regulation of immune response	670	1.574E-03	1.868E-02	1.574E-03	1.868E-02	15	CRK, STAT5A, TIRAP (Mal), p38 MAPK, ERK1 (MAPK3), STAT5, RPS27A, Ubiquitin, ERK1/2, GRB2, UBC, UBB, IGHG1, ACP33, SAP
269	regulation of macromolecule metabolic process	6163	1.576E-03	1.868E-02	1.576E-03	1.868E-02	77	Annexin VIII, PEX2, LAGY, DLC1 (Dynein LC8a), LCORL, TIPIN, SCMH1, CRK, HBG, STAT5A, TIRAP (Mal), GGA1, p38 MAPK, ERK1 (MAPK3), Bim, STAT5, RBP-J kappa (CBF1), FERD3L, SMIF, Twinfilin, BMAL2, Autophagin-1, RPS27A, p38gamma (MAPK12), ZNF397, Ubiquitin, EYA1, PhLP, XPF, Phosphatase regulator (inhibitor), Fatty acid-binding protein, ERK1/2, CRABP2, PQBP-1, LCKBP1, GA17, CrkL, Ajuba, PEX19, HXK4, ZNF655, COG3, UBC, HBGA, SNX1, ST13 (Hip), HKR1, ANXA8L2, Dysbindin, Notch, RAD23A, UBB, IRX4, ARID4A, Cyclophilin E, NOTCH1 precursor, GCK(MAP4K2), 4E-BP1, SCML1, DBC1, USP5, AO7, GGA, IL1RN, ZNF397OS, DYNLL, Cystatin B, WWOX, DKK3, PYDC1 (POP1), Adult hemoglobin, PAIP1, MLX, CAMK2N1, APOBEC-1, MLCK, PAF1
270	cellular response to chemical stimulus	2945	1.576E-03	1.868E-02	1.576E-03	1.868E-02	43	MST3, Reticulon 4, CRK, STAT5A, TIRAP (Mal), p38 MAPK, ERK1 (MAPK3), Bim, Metallothionein-I, STAT5, SMIF, RPS27A, p38gamma (MAPK12), Ubiquitin, IMD2, Phosphatase regulator (inhibitor), PDXP, Fatty acid-binding protein, ERK1/2, Metallothionein-IG, LCKBP1, CrkL, GRB2, HXK4, DDC, Metallothionein-II, HMBS, UBC, GBP5, NUP93, Notch, UBB, Guanylate cyclase, NOTCH1 precursor, 4E-BP1, Metallothionein-1M, IL1RN, WWOX, PYDC1 (POP1), Adult hemoglobin, APOBEC-1, MLCK, PAF1

271	system development	4990	1.583E-03	1.869E-02	1.583E-03	1.869E-02	65	MST3, Neurochondrin, PEX2, LAGY, Reticulon 4, DLC1 (Dynein LC8a), Beta crystallin B2, VAMP5, STAT5A, SPRR1A, TIRAP (Mal), RAD1, p38 MAPK, ERK1 (MAPK3), Bim, Metallothionein-I, CERT, STAT5, EFHD1, RBP-J kappa (CBF1), FERD3L, NHS, p38gamma (MAPK12), Ubiquitin, IMD2, EYA1, Phosphatase regulator (inhibitor), Fatty acid-binding protein, ERK1/2, NIP2, ARHGEF10, MYLK1, CRABP2, Metallothionein-IG, PQBP-1, LCKBP1, SPRR4, NEPH2, CrkL, GRB2, PTP-2, HXK4, Metallothionein-II, HMBS, Telokin, Dysbindin, Notch, ACSBG1, UBB, PAP41, IRX4, Guanylate cyclase, ARID4A, NOTCH1 precursor, 4E-BP1, Sprr2f, BMP1, DYNLL, WWOX, E-FABP, DKK3, Adult hemoglobin, Melusin, SPRR3, MLCK
272	positive regulation of signaling	1420	1.599E-03	1.872E-02	1.599E-03	1.872E-02	25	CRK, TIRAP (Mal), p38 MAPK, ERK1 (MAPK3), Bim, LASP1, RBP-J kappa (CBF1), RPS27A, Ubiquitin, EYA1, ERK1/2, NIP2, LCKBP1, CrkL, Ajuba, GRB2, UBC, Notch, UBB, BMF, NOTCH1 precursor, GCK(MAP4K2), IL1RN, WWOX, SAP
273	negative regulation of growth	289	1.602E-03	1.872E-02	1.602E-03	1.872E-02	9	Reticulon 4, TIRAP (Mal), Metallothionein-I, Metallothionein-IG, Guanylate cyclase A (NPR1), Metallothionein-II, Guanylate cyclase, RRAD, Metallothionein-1M
274	regulation of mRNA stability	54	1.603E-03	1.872E-02	1.603E-03	1.872E-02	4	TIRAP (Mal), p38 MAPK, PAIP1, APOBEC-1
275	cellular response to oxygen levels	182	1.619E-0	1.883E-0	1.619E-0	1.883E-0	7	RPS27A, Ubiquitin, Fatty acid-binding protein, UBC, Notch, UBB, NOTCH1 precursor

			3	2	3	2		
276	cytoskeleton organization	961	1.654E-03	1.917E-02	1.654E-03	1.917E-02	19	ZNF261, DLC1 (Dynein LC8a), DCTN2, EYA1, Phosphatase regulator (inhibitor), PDXP, NIP2, ARHGEF10, MYLK1, Kizuna (C20orf19), LCKBP1, Rap1GDS1, Tropomodulin, Telokin, Dysbindin, DYNLL, Rich1, RIP, MLCK
277	transmembrane receptor protein serine/threonine kinase signaling pathway	291	1.679E-03	1.939E-02	1.679E-03	1.939E-02	9	p38 MAPK, ERK1 (MAPK3), SMIF, RPS27A, Ubiquitin, Phosphatase regulator (inhibitor), ERK1/2, UBC, UBB
278	regulation of phosphate metabolic process	2257	1.718E-03	1.945E-02	1.718E-03	1.945E-02	35	DLC1 (Dynein LC8a), CRK, TIRAP (Mal), p38 MAPK, ERK1 (MAPK3), Twinfilin, RPS27A, Ubiquitin, SNX9, Phosphatase regulator (inhibitor), Fatty acid-binding protein, ERK1/2, NIP2, ARHGEF10, LCKBP1, Guanylate cyclase A (NPR1), CrkL, Ajuba, GRB2, HXK4, Rap1GDS1, UBC, PCP2, UBB, Guanylate cyclase, GCK(MAP4K2), IL1RN, FAM116A, DYNLL, PYDC1 (POP1), Adult hemoglobin, CAMK2N1, Rich1, RIP, MLCK
279	growth involved in heart morphogenesis	7	1.757E-03	1.945E-02	1.757E-03	1.945E-02	2	Notch, NOTCH1 precursor
280	allantoin metabolic process	7	1.757E-03	1.945E-02	1.757E-03	1.945E-02	2	STAT5A, STAT5

281	pericardium morphogenesis	7	1.757E-03	1.945E-02	1.757E-03	1.945E-02	2	Notch, NOTCH1 precursor
282	cellular response to copper ion	7	1.757E-03	1.945E-02	1.757E-03	1.945E-02	2	Metallothionein-I, Metallothionein-IG
283	endocardial cell differentiation	7	1.757E-03	1.945E-02	1.757E-03	1.945E-02	2	Notch, NOTCH1 precursor
284	cardiac right ventricle formation	7	1.757E-03	1.945E-02	1.757E-03	1.945E-02	2	Notch, NOTCH1 precursor
285	fat pad development	7	1.757E-03	1.945E-02	1.757E-03	1.945E-02	2	Ubiquitin, UBB
28	aortic valve morphogenesis	7	1.	1.	1.	1.	2	Notch, NOTCH1 precursor

6			757E-03	945E-02	757E-03	945E-02		
287	[regulation of mast cell apoptotic process]()	7	1.757E-03	1.945E-02	1.757E-03	1.945E-02	2	STAT5A, STAT5
288	[caveolin-mediated endocytosis]()	7	1.757E-03	1.945E-02	1.757E-03	1.945E-02	2	ERK1 (MAPK3), ERK1/2
289	[aortic valve development]()	7	1.757E-03	1.945E-02	1.757E-03	1.945E-02	2	Notch, NOTCH1 precursor
290	[DNA-templated transcription, initiation]()	297	1.930E-03	2.125E-02	1.930E-03	2.125E-02	9	ERK1 (MAPK3), RBP-J kappa (CBF1), RPS27A, Ubiquitin, ERK1/2, UBC, Notch, UBB, NOTCH1 precursor
291	[actin cytoskeleton organization]()	482	1.93	2.12	1.93	2.12	12	DLC1 (Dynein LC8a), Phosphatase regulator (inhibitor), PDXP, MYLK1, LCKBP1, Rap1GDS1, Tropomodulin, Telokin, Dysbindin, DYNLL, Rich1, MLCK

			8E-03	5E-02	8E-03	5E-02		
292	regulation of RNA stability	57	1.959E-03	2.125E-02	1.959E-03	2.125E-02	4	TIRAP (Mal), p38 MAPK, PAIP1, APOBEC-1
293	nucleotide-binding domain, leucine rich repeat containing receptor signaling pathway	57	1.959E-03	2.125E-02	1.959E-03	2.125E-02	4	RPS27A, Ubiquitin, UBC, UBB
294	regulation of erythrocyte differentiation	57	1.959E-03	2.125E-02	1.959E-03	2.125E-02	4	STAT5A, p38 MAPK, STAT5, Adult hemoglobin
295	regulation of phosphorus metabolic process	2275	1.965E-03	2.125E-02	1.965E-03	2.125E-02	35	DLC1 (Dynein LC8a), CRK, TIRAP (Mal), p38 MAPK, ERK1 (MAPK3), Twinfilin, RPS27A, Ubiquitin, SNX9, Phosphatase regulator (inhibitor), Fatty acid-binding protein, ERK1/2, NIP2, ARHGEF10, LCKBP1, Guanylate cyclase A (NPR1), CrkL, Ajuba, GRB2, HXK4, Rap1GDS1, UBC, PCP2, UBB, Guanylate cyclase, GCK(MAP4K2), IL1RN, FAM116A, DYNLL, PYDC1 (POP1), Adult hemoglobin, CAMK2N1, Rich1, RIP, MLCK
296	epithelial cell fate commitment	27	1.973E	2.125E	1.973E	2.125E	3	RBP-J kappa (CBF1), Notch, NOTCH1 precursor

			-03	-02	-03	-02		
297	hair follicle maturation	27	1.973E-03	2.125E-02	1.973E-03	2.125E-02	3	RBP-J kappa (CBF1), Notch, NOTCH1 precursor
298	cellular response to abiotic stimulus	358	2.002E-03	2.149E-02	2.002E-03	2.149E-02	10	RAD1, p38 MAPK, ERK1 (MAPK3), ERK1/2, MYLK1, GRB2, Telokin, PCP2, Guanylate cyclase, MLCK
299	monosaccharide metabolic process	299	2.019E-03	2.160E-02	2.019E-03	2.160E-02	9	p38 MAPK, RPS27A, Ubiquitin, Phosphatase regulator (inhibitor), Fatty acid-binding protein, HXK4, UBC, UBB, E-FABP
300	negative regulation of nitrogen compound metabolic process	1446	2.046E-03	2.179E-02	2.046E-03	2.179E-02	25	PEX2, LAGY, DLC1 (Dynein LC8a), TIPIN, SCMH1, HBG, RBP-J kappa (CBF1), FERD3L, RPS27A, Ubiquitin, XPF, Fatty acid-binding protein, LCKBP1, Ajuba, UBC, HBGA, Notch, UBB, ARID4A, NOTCH1 precursor, DYNLL, DKK3, Adult hemoglobin, MLX, PAF1
301	ribonucleoside monophosphate biosynthetic process	96	2.050E-0	2.179E-0	2.050E-0	2.179E-0	5	UCK1, IMD2, ATP1A3, ADSL, RIFK

			3	2	3	2		
302	response to growth hormone	58	2.089E-03	2.213E-02	2.089E-03	2.213E-02	4	STAT5A, ERK1 (MAPK3), STAT5, ERK1/2
303	apoptotic process involved in development	28	2.195E-03	2.317E-02	2.195E-03	2.317E-02	3	Bim, Notch, NOTCH1 precursor
304	regulation of mitochondrial outer membrane permeabilization involved in apoptotic signaling pathway	59	2.225E-03	2.342E-02	2.225E-03	2.342E-02	4	DLC1 (Dynein LC8a), Bim, BMF, DYNLL
305	regulation of epidermal growth factor receptor signaling pathway	98	2.244E-03	2.350E-02	2.244E-03	2.350E-02	5	RPS27A, Ubiquitin, GRB2, UBC, UBB
306	negative regulation of molecular function	1218	2.248E-03	2.350E-02	2.248E-03	2.350E-02	22	Annexin VIII, DLC1 (Dynein LC8a), ERK1 (MAPK3), RPS27A, Ubiquitin, Phosphatase regulator (inhibitor), Fatty acid-binding protein, ERK1/2, Ajuba, PEX19, UBC, ANXA8L2, Notch, UBB, PAP41, NOTCH1 precursor, DBC1, IL1RN, DYNLL, Cystatin B, PYDC1 (POP1), CAMK2N1

307	positive regulation of intracellular signal transduction	913	2.256E-03	2.351E-02	2.256E-03	2.351E-02	18	CRK, TIRAP (Mal), p38 MAPK, ERK1 (MAPK3), Bim, RPS27A, Ubiquitin, ERK1/2, NIP2, LCKBP1, Ajuba, UBC, Notch, UBB, BMF, NOTCH1 precursor, GCK(MAP4K2), IL1RN
308	negative regulation of biosynthetic process	1538	2.265E-03	2.353E-02	2.265E-03	2.353E-02	26	PEX2, LAGY, DLC1 (Dynein LC8a), TIPIN, SCMH1, HBG, RBP-J kappa (CBF1), FERD3L, RPS27A, Ubiquitin, Fatty acid-binding protein, LCKBP1, Ajuba, HXK4, UBC, HBGA, Notch, UBB, ARID4A, NOTCH1 precursor, 4E-BP1, DYNLL, DKK3, Adult hemoglobin, MLX, PAF1
309	response to hypoxia	427	2.282E-03	2.362E-02	2.282E-03	2.362E-02	11	STAT5, RPS27A, Ubiquitin, Fatty acid-binding protein, ADSL, Ajuba, HMBS, UBC, Notch, UBB, NOTCH1 precursor
310	regulation of photoreceptor cell differentiation	8	2.328E-03	2.363E-02	2.328E-03	2.363E-02	2	Notch, NOTCH1 precursor
311	taurine metabolic process	8	2.328E-03	2.363E-02	2.328E-03	2.363E-02	2	STAT5A, STAT5
31	positive regulation of histone phosphorylation	8	2.	2.	2.	2.	2	ERK1 (MAPK3), ERK1/2

2			328E-03	363E-02	328E-03	363E-02		
313	compartment pattern specification	8	2.328E-03	2.363E-02	2.328E-03	2.363E-02	2	Notch, NOTCH1 precursor
314	regulation of early endosome to late endosome transport	8	2.328E-03	2.363E-02	2.328E-03	2.363E-02	2	ERK1 (MAPK3), ERK1/2
315	cardiac vascular smooth muscle cell development	8	2.328E-03	2.363E-02	2.328E-03	2.363E-02	2	Notch, NOTCH1 precursor
316	apoptotic process	1380	2.334E-03	2.363E-02	2.334E-03	2.363E-02	24	MST3, Reticulon 4, DLC1 (Dynein LC8a), p38 MAPK, ERK1 (MAPK3), Bim, RPS27A, Ubiquitin, Phosphatase regulator (inhibitor), Fatty acid-binding protein, ERK1/2, NIP2, RIFK, UBC, CED-6, Notch, UBB, BMF, NOTCH1 precursor, DBC1, DYNLL, Occludin, WWOX, Galectin-13
317	establishment of protein localization	1461	2.34	2.37	2.34	2.37	25	PEX2, VAMP5, GGA1, NASP, ERK1 (MAPK3), RBP-J kappa (CBF1), Autophagin-1, RPS27A, SNX9, Kinesin light chain, Phosphatase regulator (inhibitor), ERK1/2, Syntaxin 16, Rab-9B, PEX19, BBS9, COG3, SNX1, NUP93, Notch,

			9E-03	0E-02	9E-03	0E-02		NOTCH1 precursor, GGA, RAB24, COG8, APOBEC-1
318	organonitrogen compound biosynthetic process	700	2.398E-03	2.412E-02	2.398E-03	2.412E-02	15	MIBP, CERT, UCK1, IMD2, ATP1A3, Fatty acid-binding protein, ADSL, Guanylate cyclase A (NPR1), MOCS2(small), DDC, RIFK, HMBS, Guanylate cyclase, CTP synthase II, E-FABP
319	regulation of protein oligomerization	29	2.431E-03	2.438E-02	2.431E-03	2.438E-02	3	Bim, SNX9, BMF
320	regulation of catalytic activity	2567	2.445E-03	2.438E-02	2.445E-03	2.438E-02	38	SAE1, Annexin VIII, DLC1 (Dynein LC8a), CRK, PPP1R12B, TIRAP (Mal), p38 MAPK, ERK1 (MAPK3), Bim, RPS27A, Ubiquitin, SNX9, Phosphatase regulator (inhibitor), Fatty acid-binding protein, ERK1/2, NIP2, ARHGEF10, CrkL, Ajuba, RIFK, Rap1GDS1, UBC, PCP2, ANXA8L2, Notch, UBB, PAP41, NOTCH1 precursor, GCK(MAP4K2), DBC1, IL1RN, FAM116A, DYNLL, Cystatin B, PYDC1 (POP1), CAMK2N1, Rich1, RIP
321	protein localization	1878	2.447E-03	2.438E-02	2.447E-03	2.438E-02	30	PEX2, VAMP5, GGA1, NASP, ERK1 (MAPK3), RBP-J kappa (CBF1), Twinfilin, Autophagin-1, RPS27A, CDC4L, EYA1, SNX9, Kinesin light chain, Phosphatase regulator (inhibitor), ERK1/2, Syntaxin 16, Ajuba, Rab-9B, PTP-2, PEX19, BBS9, COG3, SNX1, NUP93, Notch, NOTCH1 precursor, GGA, RAB24, COG8, APOBEC-1
322	epidermal cell differentiation	196	2.461E	2.445E	2.461E	2.445E	7	SPRR1A, RBP-J kappa (CBF1), SPRR4, Notch, NOTCH1 precursor, Sprr2f, SPRR3

			-03	-02	-03	-02		
323	regulation of body fluid levels	847	2.480E-03	2.456E-02	2.480E-03	2.456E-02	17	Annexin VIII, KLC2, CRK, HBG, STAT5A, p38 MAPK, ERK1 (MAPK3), STAT5, Kinesin light chain, ERK1/2, Guanylate cyclase A (NPR1), GRB2, HBGA, ANXA8L2, Dysbindin, Guanylate cyclase, Adult hemoglobin
324	response to decreased oxygen levels	433	2.541E-03	2.508E-02	2.541E-03	2.508E-02	11	STAT5, RPS27A, Ubiquitin, Fatty acid-binding protein, ADSL, Ajuba, HMBS, UBC, Notch, UBB, NOTCH1 precursor
325	small GTPase mediated signal transduction	500	2.618E-03	2.577E-02	2.618E-03	2.577E-02	12	p38 MAPK, ERK1 (MAPK3), p38gamma (MAPK12), ERK1/2, CrkL, GRB2, Rab-9B, CNK1, RRAD, RAB24, Rich1, DOK3
326	organic cyclic compound biosynthetic process	3482	2.665E-03	2.615E-02	2.665E-03	2.615E-02	48	PEX2, LAGY, MIBP, DLC1 (Dynein LC8a), LCORL, GDP-mannose 4,6 dehydratase, SCMH1, STAT5A, p38 MAPK, ERK1 (MAPK3), STAT5, RBP-J kappa (CBF1), FERD3L, BMAL2, UCK1, RPS27A, p38gamma (MAPK12), ZNF397, Ubiquitin, IMD2, EYA1, Phosphatase regulator (inhibitor), ATP1A3, ERK1/2, ADSL, PQBP-1, Guanylate cyclase A (NPR1), MOCS2(small), Ajuba, DDC, RIFK, ZNF655, HMBS, UBC, HKR1, Notch, UBB, PAP41, Guanylate cyclase, ARID4A, NOTCH1 precursor, SCML1, CTP synthase II, ZNF397OS, DYNLL, GOCAP1, MLX, PAF1
327	response to transition metal nanoparticle	149	2.757E-	2.698E-	2.757E-	2.698E-	6	Metallothionein-I, Metallothionein-IG, Metallothionein-II, HMBS, Metallothionein-1M, APOBEC-1

			03	02	03	02		
328	single-organism metabolic process	5494	2.837E-03	2.743E-02	2.837E-03	2.743E-02	69	MST3, PEX2, MUC15, LAGY, MIBP, GDP-mannose 4,6 dehydratase, CRK, STAT5A, TIRAP (Mal), RAD1, p38 MAPK, ERK1 (MAPK3), Carbonic anhydrase XIII, CERT, STAT5, RBP1, Autophagin-1, UCK1, RPS27A, p38gamma (MAPK12), Ubiquitin, IMD2, EYA1, NANP, XPF, Phosphatase regulator (inhibitor), PDXP, ATP1A3, Fatty acid-binding protein, ERK1/2, ADSL, CRABP2, Guanylate cyclase A (NPR1), MOCS2(small), CrkL, Ajuba, GRB2, HXK4, DDC, RIFK, COG3, HMBS, UBC, GBP5, NUP93, ACSBG1, RAD23A, UBB, PAP41, Guanylate cyclase, IGHG1, ARID4A, GCK(MAP4K2), RRAD, DHRS10, IL1RN, CTP synthase II, RPA4, BMP1, DSS1, Occludin, WWOX, E-FABP, Adult hemoglobin, GOCAP1, MLX, APOBEC-1, PAF1, Galectin-13
329	positive regulation of metabolic process	3038	2.870E-03	2.743E-02	2.870E-03	2.743E-02	43	CRK, STAT5A, TIRAP (Mal), GGA1, p38 MAPK, ERK1 (MAPK3), STAT5, RBP-J kappa (CBF1), SMIF, BMAL2, Autophagin-1, RPS27A, Ubiquitin, EYA1, SCOCO, Fatty acid-binding protein, ERK1/2, NIP2, ARHGEF10, LCKBP1, Guanylate cyclase A (NPR1), CrkL, Ajuba, GRB2, HXK4, UBC, SNX1, Dysbindin, Notch, UBB, Guanylate cyclase, NOTCH1 precursor, GCK(MAP4K2), USP5, GGA, IL1RN, WWOX, Adult hemoglobin, PAIP1, MLX, APOBEC-1, MLCK, PAF1
330	regulation of myeloid cell differentiation	257	2.892E-03	2.743E-02	2.892E-03	2.743E-02	8	STAT5A, p38 MAPK, STAT5, RBP1, LCKBP1, Notch, Adult hemoglobin, PAF1
331	nucleoside monophosphate biosynthetic process	104	2.906E-03	2.743E-02	2.906E-03	2.743E-02	5	UCK1, IMD2, ATP1A3, ADSL, RIFK
332	epidermis development	377	2.90	2.74	2.90	2.74	10	SPRR1A, RBP-J kappa (CBF1), Fatty acid-binding protein, CRABP2, SPRR4, Notch, NOTCH1 precursor, Sprr2f, E-FABP, SPRR3

			6E-03	3E-02	6E-03	3E-02		
333	programmed cell death	1406	2.967E-03	2.743E-02	2.967E-03	2.743E-02	24	MST3, Reticulon 4, DLC1 (Dynein LC8a), p38 MAPK, ERK1 (MAPK3), Bim, RPS27A, Ubiquitin, Phosphatase regulator (inhibitor), Fatty acid-binding protein, ERK1/2, NIP2, RIFK, UBC, CED-6, Notch, UBB, BMF, NOTCH1 precursor, DBC1, DYNLL, Occludin, WWOX, Galectin-13
334	regulation of serine-type endopeptidase activity	9	2.975E-03	2.743E-02	2.975E-03	2.743E-02	2	Annexin VIII, ANXA8L2
335	negative regulation of erythrocyte differentiation	9	2.975E-03	2.743E-02	2.975E-03	2.743E-02	2	STAT5A, STAT5
336	negative regulation of cell migration involved in sprouting angiogenesis	9	2.975E-03	2.743E-02	2.975E-03	2.743E-02	2	Notch, NOTCH1 precursor
337	citrate metabolic process	9	2.975E	2.743E	2.975E	2.743E	2	STAT5A, STAT5

			-03	-02	-03	-02		
338	monocyte activation	9	2.975E-03	2.743E-02	2.975E-03	2.743E-02	2	Metallothionein-I, Metallothionein-IG
339	hypothalamus cell differentiation	9	2.975E-03	2.743E-02	2.975E-03	2.743E-02	2	Ubiquitin, UBB
340	peptidyl-tyrosine autophosphorylation	9	2.975E-03	2.743E-02	2.975E-03	2.743E-02	2	ERK1 (MAPK3), ERK1/2
341	coronary artery morphogenesis	9	2.975E-03	2.743E-02	2.975E-03	2.743E-02	2	Notch, NOTCH1 precursor
342	negative regulation of phospholipase activity	9	2.975E-0	2.743E-0	2.975E-0	2.743E-0	2	Annexin VIII, ANXA8L2

			3	2	3	2		
343	valine metabolic process	9	2.975E-03	2.743E-02	2.975E-03	2.743E-02	2	STAT5A, STAT5
344	female meiosis I	9	2.975E-03	2.743E-02	2.975E-03	2.743E-02	2	Ubiquitin, UBB
345	negative regulation of serine-type endopeptidase activity	9	2.975E-03	2.743E-02	2.975E-03	2.743E-02	2	Annexin VIII, ANXA8L2
346	positive regulation of gamma-delta T cell differentiation	9	2.975E-03	2.743E-02	2.975E-03	2.743E-02	2	STAT5A, STAT5
347	positive regulation of protein homooligomerization	9	2.975E-03	2.743E-02	2.975E-03	2.743E-02	2	Bim, BMF

348	fibroblast growth factor receptor signaling pathway	203	2.991E-03	2.747E-02	2.991E-03	2.747E-02	7	ERK1 (MAPK3), RPS27A, Ubiquitin, ERK1/2, GRB2, UBC, UBB
349	epithelial to mesenchymal transition	64	2.997E-03	2.747E-02	2.997E-03	2.747E-02	4	Reticulon 4, RBP-J kappa (CBF1), Notch, NOTCH1 precursor
350	mRNA metabolic process	718	3.046E-03	2.784E-02	3.046E-03	2.784E-02	15	p38 MAPK, SMIF, Tex1, RPS27A, Ubiquitin, Phosphatase regulator (inhibitor), Sen15, RRP42, UBC, UBB, Cyclophilin E, DBC1, PAIP1, APOBEC-1, PAF1
351	regulation of response to stimulus	3878	3.066E-03	2.790E-02	3.066E-03	2.790E-02	52	MST3, LAGY, Reticulon 4, DLC1 (Dynein LC8a), CRK, STAT5A, TIRAP (Mal), p38 MAPK, IL-1F5, ERK1 (MAPK3), Bim, LASP1, STAT5, RBP-J kappa (CBF1), RPS27A, Ubiquitin, EYA1, PhLP, SCOCO, Phosphatase regulator (inhibitor), Fatty acid-binding protein, ERK1/2, NIP2, ARHGEF10, MYLK1, LCKBP1, CrkL, Ajuba, GRB2, Rap1GDS1, UBC, Telokin, Dysbindin, Notch, UBB, BMF, Guanylate cyclase, IGHG1, NOTCH1 precursor, GCK(MAP4K2), DBC1, IL1RN, FAM116A, DYNLL, WWOX, DKK3, ACP33, Adult hemoglobin, Rich1, SAP, RIP, MLCK
352	cellular metabolic process	9941	3.070E-03	2.790E-02	3.070E-03	2.790E-02	112	MST3, SAE1, PEX2, MUC15, LAGY, MIBP, DLC1 (Dynein LC8a), LCORL, GDP-mannose 4,6 dehydratase, TIPIN, SCMH1, CRK, RNASE6, Calpain 1(mu), STAT5A, SPRR1A, TIRAP (Mal), RAD1, p38 MAPK, NASP, ERK1 (MAPK3), Carbonic anhydrase XIII, CERT, STAT5, RBP-J kappa (CBF1), FERD3L, RBP1, SMIF, Twinfilin, BMAL2, Autophagin-1, Tex1, UCK1, RPS27A, UIP5, p38gamma (MAPK12), ZNF397, Ubiquitin, IMD2, EYA1, PhLP, NANP, DUPD1, XPF, Phosphatase regulator (inhibitor), PDXP, LYPLAL1, ATP1A3, Fatty acid-binding protein, ERK1/2, ADSL, MYLK1, CRABP2, PQBP-1, SPRR4, Guanylate cyclase

								A (NPR1), MOCS2(small), GA17, CrkL, Ajuba, GRB2, PTP-2, CNK1, PEX19, HXK4, DDC, RIFK, ZNF655, Sen15, COG3, HMBS, RRP42, UBC, Telokin, ST13 (Hip), C16orf75, GBP5, HKR1, Notch, ACSBG1, RAD23A, UBB, PAP41, Guanylate cyclase, ARID4A, Cyclophilin E, NOTCH1 precursor, GCK(MAP4K2), 4E-BP1, RRAD, SCML1, DBC1, GINS2, USP5, AO7, RAB24, IL1RN, CTP synthase II, RPA4, ZNF397OS, DYNLL, DSS1, Occludin, E-FABP, Adult hemoglobin, PAIP1, MLX, APOBEC-1, SPRR3, MLCK, PAF1, Galectin-13
353	heterocycle biosynthetic process	3324	3.101E-03	2.804E-02	3.101E-03	2.804E-02	46	LAGY, MIBP, DLC1 (Dynein LC8a), LCORL, GDP-mannose 4,6 dehydratase, SCMH1, STAT5A, p38 MAPK, ERK1 (MAPK3), STAT5, RBP-J kappa (CBF1), FERD3L, BMAL2, UCK1, RPS27A, p38gamma (MAPK12), ZNF397, Ubiquitin, IMD2, EYA1, Phosphatase regulator (inhibitor), ATP1A3, ERK1/2, ADSL, PQBP-1, Guanylate cyclase A (NPR1), MOCS2(small), Ajuba, DDC, RIFK, ZNF655, HMBS, UBC, HKR1, Notch, UBB, PAP41, Guanylate cyclase, ARID4A, NOTCH1 precursor, SCML1, CTP synthase II, ZNF397OS, DYNLL, MLX, PAF1
354	cell differentiation	3974	3.103E-03	2.804E-02	3.103E-03	2.804E-02	53	MST3, Neurochondrin, PEX2, LAGY, Reticulon 4, VAMP5, STAT5A, SPRR1A, TIRAP (Mal), TCL1A, p38 MAPK, ERK1 (MAPK3), Bim, Metallothionein-I, STAT5, EFHD1, RBP-J kappa (CBF1), FERD3L, NHS, p38gamma (MAPK12), Ubiquitin, EYA1, Phosphatase regulator (inhibitor), Fatty acid-binding protein, ERK1/2, NIP2, ARHGEF10, Metallothionein-IG, PQBP-1, LCKBP1, SPRR4, NEPH2, GRB2, PTP-2, BBS9, Metallothionein-II, Tropomodulin, HMBS, Dysbindin, Notch, UBB, IRX4, ARID4A, NOTCH1 precursor, Sprr2f, BMP1, DYNLL, WWOX, Adult hemoglobin, RIP, SPRR3, MLCK, PAF1
355	negative regulation of cellular metabolic process	2082	3.176E-03	2.862E-02	3.176E-03	2.862E-02	32	PEX2, LAGY, DLC1 (Dynein LC8a), TIPIN, SCMH1, HBG, RBP-J kappa (CBF1), FERD3L, RPS27A, Ubiquitin, PhLP, XPF, Fatty acid-binding protein, LCKBP1, Ajuba, HXK4, UBC, HBGA, ST13 (Hip), Notch, UBB, ARID4A, NOTCH1 precursor, 4E-BP1, DYNLL, Cystatin B, DKK3, PYDC1 (POP1), Adult hemoglobin, MLX, CAMK2N1, PAF1
356	positive regulation of natural killer cell mediated cytotoxicity	32	3.233E-0	2.889E-0	3.233E-0	2.889E-0	3	STAT5A, STAT5, SAP

			3	2	3	2		
357	endocardial cushion development	32	3.233E-03	2.889E-02	3.233E-03	2.889E-02	3	RBP-J kappa (CBF1), Notch, NOTCH1 precursor
358	heart trabecula morphogenesis	32	3.233E-03	2.889E-02	3.233E-03	2.889E-02	3	RBP-J kappa (CBF1), Notch, NOTCH1 precursor
359	regulation of protein metabolic process	2522	3.274E-03	2.917E-02	3.274E-03	2.917E-02	37	Annexin VIII, CRK, STAT5A, TIRAP (Mal), GGA1, p38 MAPK, ERK1 (MAPK3), Bim, STAT5, Twinfilin, Autophagin-1, RPS27A, Ubiquitin, PhLP, Phosphatase regulator (inhibitor), Fatty acid-binding protein, ERK1/2, LCKBP1, GA17, CrkL, Ajuba, UBC, SNX1, ST13 (Hip), ANXA8L2, RAD23A, UBB, GCK(MAP4K2), 4E-BP1, USP5, GGA, IL1RN, Cystatin B, PYDC1 (POP1), Adult hemoglobin, PAIP1, CAMK2N1
360	regulation of catabolic process	1101	3.339E-03	2.967E-02	3.339E-03	2.967E-02	20	CRK, GGA1, Autophagin-1, SNX9, SCOCO, Phosphatase regulator (inhibitor), Fatty acid-binding protein, NIP2, ARHGEF10, Ajuba, HXK4, Rap1GDS1, SNX1, PCP2, RAD23A, USP5, GGA, FAM116A, Rich1, RIP
361	carbohydrate biosynthetic process	155	3.352E-03	2.970E-02	3.352E-03	2.970E-02	6	RPS27A, Ubiquitin, Phosphatase regulator (inhibitor), HXK4, UBC, UBB

362	posttranscriptional regulation of gene expression	585	3.405E-03	3.009E-02	3.405E-03	3.009E-02	13	PEX2, TIRAP (Mal), p38 MAPK, ERK1 (MAPK3), Phosphatase regulator (inhibitor), ERK1/2, GA17, Ajuba, PEX19, COG3, 4E-BP1, PAIP1, APOBEC-1
363	Fc receptor signaling pathway	324	3.448E-03	3.038E-02	3.448E-03	3.038E-02	9	CRK, ERK1 (MAPK3), RPS27A, Ubiquitin, ERK1/2, GRB2, UBC, UBB, IGHG1
364	regulation of growth	802	3.506E-03	3.081E-02	3.506E-03	3.081E-02	16	LAGY, Reticulon 4, STAT5A, TIRAP (Mal), Bim, Metallothionein-I, STAT5, RBP-J kappa (CBF1), Metallothionein-IG, Guanylate cyclase A (NPR1), Metallothionein-II, Notch, Guanylate cyclase, NOTCH1 precursor, RRAD, Metallothionein-1M
365	positive regulation of natural killer cell mediated immunity	33	3.532E-03	3.096E-02	3.532E-03	3.096E-02	3	STAT5A, STAT5, SAP
366	activation of immune response	520	3.589E-03	3.137E-02	3.589E-03	3.137E-02	12	CRK, TIRAP (Mal), p38 MAPK, ERK1 (MAPK3), RPS27A, Ubiquitin, ERK1/2, GRB2, UBC, UBB, IGHG1, ACP33
36	negative regulation of cellular	13	3.	3.	3.	3.	23	PEX2, LAGY, TIPIN, SCMH1, HBG, RBP-J kappa (CBF1), FERD3L, RPS27A, Ubiquitin, Fatty acid-binding protein,

7	macromolecule biosynthetic process	49	6.79E-03	1.37E-02	6.79E-03	1.37E-02		LCKBP1, Ajuba, UBC, HBGA, Notch, UBB, ARID4A, NOTCH1 precursor, 4E-BP1, DKK3, Adult hemoglobin, MLX, PAF1
368	negative regulation of serine-type peptidase activity	10	3.696E-03	3.137E-02	3.696E-03	3.137E-02	2	Annexin VIII, ANXA8L2
369	retinoic acid biosynthetic process	10	3.696E-03	3.137E-02	3.696E-03	3.137E-02	2	RBP1, CRABP2
370	regulation of serine-type peptidase activity	10	3.696E-03	3.137E-02	3.696E-03	3.137E-02	2	Annexin VIII, ANXA8L2
371	glomerular mesangial cell development	10	3.696E-03	3.137E-02	3.696E-03	3.137E-02	2	Notch, NOTCH1 precursor
372	energy homeostasis	10	3.69	3.13	3.69	3.13	2	Ubiquitin, UBB

			6E-03	7E-02	6E-03	7E-02		
373	negative regulation of glial cell proliferation	10	3.696E-03	3.137E-02	3.696E-03	3.137E-02	2	Notch, NOTCH1 precursor
374	mitral valve morphogenesis	10	3.696E-03	3.137E-02	3.696E-03	3.137E-02	2	Notch, NOTCH1 precursor
375	venous blood vessel morphogenesis	10	3.696E-03	3.137E-02	3.696E-03	3.137E-02	2	Notch, NOTCH1 precursor
376	pulmonary valve morphogenesis	10	3.696E-03	3.137E-02	3.696E-03	3.137E-02	2	Notch, NOTCH1 precursor
377	pulmonary valve development	10	3.696E	3.137E	3.696E	3.137E	2	Notch, NOTCH1 precursor

			-03	-02	-03	-02		
378	keratinization	68	3.732E-03	3.150E-02	3.732E-03	3.150E-02	4	SPRR1A, SPRR4, Sprr2f, SPRR3
379	JAK-STAT cascade	68	3.732E-03	3.150E-02	3.732E-03	3.150E-02	4	STAT5A, ERK1 (MAPK3), STAT5, ERK1/2
380	coagulation	663	3.787E-03	3.179E-02	3.787E-03	3.179E-02	14	Annexin VIII, KLC2, CRK, HBG, p38 MAPK, ERK1 (MAPK3), Kinesin light chain, ERK1/2, GRB2, HBGA, ANXA8L2, Dysbindin, Guanylate cyclase, Adult hemoglobin
381	blood coagulation	663	3.787E-03	3.179E-02	3.787E-03	3.179E-02	14	Annexin VIII, KLC2, CRK, HBG, p38 MAPK, ERK1 (MAPK3), Kinesin light chain, ERK1/2, GRB2, HBGA, ANXA8L2, Dysbindin, Guanylate cyclase, Adult hemoglobin
382	cellular response to metal ion	159	3.798E-0	3.181E-0	3.798E-0	3.181E-0	6	Metallothionein-I, Fatty acid-binding protein, Metallothionein-IG, Metallothionein-II, HMBS, Metallothionein-1M

			3	2	3	2		
383	positive regulation of BMP signaling pathway	34	3.848E-03	3.214E-02	3.848E-03	3.214E-02	3	RBP-J kappa (CBF1), Notch, NOTCH1 precursor
384	response to stimulus	9680	3.937E-03	3.280E-02	3.937E-03	3.280E-02	109	MST3, Annexin VIII, KLC2, Reticulon 4, Siglec-8, DLC1 (Dynein LC8a), GDP-mannose 4,6 dehydratase, TIPIN, CRK, RNASE6, HBG, Beta crystallin B2, PPP1R12B, STAT5A, TIRAP (Mal), RAD1, p38 MAPK, ERK1 (MAPK3), Bim, Metallothionein-I, CERT, STAT5, RBP-J kappa (CBF1), RBP1, SMIF, BMAL2, Autophagin-1, RPS27A, p38gamma (MAPK12), Ccdc109a, Ubiquitin, IMD2, CDC4L, Sti1, EYA1, PhLP, KAAG1, Kinesin light chain, XPF, Phosphatase regulator (inhibitor), PDXP, ATP1A3, Fatty acid-binding protein, ERK1/2, ADSL, MYLK1, CRABP2, Metallothionein-IG, LCKBP1, Guanylate cyclase A (NPR1), NEPH2, CrkL, Ajuba, GRB2, Rab-9B, PTP-2, CNK1, HXK4, DDC, BBS9, Metallothionein-II, Tropomodulin, HMBS, UBC, HBGA, Telokin, SAAL1, PCP2, CED-6, GBP5, NUP93, ANXA8L2, Siglec-E, Dysbindin, Notch, ACSBG1, RAD23A, UBB, BMF, Guanylate cyclase, IGHG1, CALCOCO2, NOTCH1 precursor, GCK(MAP4K2), 4E-BP1, RRAD, Sprr2f, DBC1, Metallothionein-1M, RAB24, IL1RN, RPA4, DYNLL, Cystatin B, DSS1, WWOX, E-FABP, DKK3, ACP33, PYDC1 (POP1), Adult hemoglobin, Rich1, Melusin, SAP, APOBEC-1, SPRR3, MLCK, PAF1, DOK3
385	negative regulation of transcription, DNA-templated	1201	4.119E-03	3.414E-02	4.119E-03	3.414E-02	21	PEX2, LAGY, SCMH1, HBG, RBP-J kappa (CBF1), FERD3L, RPS27A, Ubiquitin, Fatty acid-binding protein, LCKBP1, Ajuba, UBC, HBGA, Notch, UBB, ARID4A, NOTCH1 precursor, DKK3, Adult hemoglobin, MLX, PAF1
386	single-organism process	15116	4.119E	3.414E	4.119E	3.414E	157	MST3, Neurochondrin, Annexin VIII, PEX2, KLC2, MUC15, LAGY, Reticulon 4, ZNF261, MIBP, Siglec-8, DLC1 (Dynein LC8a), GDP-mannose 4,6 dehydratase, TIPIN, SCMH1, CRK, HBG, Beta crystallin B2, DCTN2, PPP1R12B, VAMP5, Calpain 1(mu), STAT5A, SPRR1A, TIRAP (Mal), RAD1, TCL1A, p38 MAPK, NASP, ERK1 (MAPK3), Carbonic

			-03	-02	-03	-02		anhydrase XIII, Bim, LASP1, Metallothionein-I, CERT, STAT5, EFHD1, RBP-J kappa (CBF1), FERD3L, RBP1, SMIF, Twinfilin, MCT4, BMAL2, Autophagin-1, MTCP1(p13), NHS, UCK1, KLC3, RPS27A, p38gamma (MAPK12), Ccdc109a, Ubiquitin, IMD2, CDC4L, EYA1, PhLP, SNX9, Kinesin light chain, NANP, XPF, Phosphatase regulator (inhibitor), PDXP, ATP1A3, Fatty acid-binding protein, ERK1/2, NIP2, ADSL, ARHGEF10, MYLK1, CRABP2, MTCP1(p8), Metallothionein-IG, PQBP-1, Kizuna (C20orf19), LCKBP1, Syntaxin 16, SPRR4, Guanylate cyclase A (NPR1), NEPH2, MOCS2(small), CrkL, Ajuba, GRB2, Rab-9B, PTP-2, CNK1, PEX19, HXK4, DDC, RIFK, Rap1GDS1, BBS9, Metallothionein-II, ZNF655, Tropomodulin, COG3, HMBS, UBC, HBGA, Telokin, SNX1, PCP2, Troponin C, skeletal muscle, CED-6, GBP5, NUP93, HKR1, ANXA8L2, Siglec-E, Dysbindin, Notch, ACSBG1, RAD23A, UBB, BMF, PAP41, IRX4, Guanylate cyclase, IGHG1, ARID4A, NOTCH1 precursor, GCK(MAP4K2), 4E-BP1, RRAD, DHRS10, Sprr2f, DBC1, GINS2, RAB24, IL1RN, CTP synthase II, RPA4, BMP1, DYNLL, Cystatin B, DSS1, Occludin, WWOX, E-FABP, DKK3, ACP33, PYDC1 (POP1), Adult hemoglobin, GOCAP1, MLX, CAMK2N1, Rich1, Melusin, SAP, APOBEC-1, RIP, SPRR3, MLCK, PAF1, Galectin-13, DOK3
387	hemostasis	670	4.152E-03	3.432E-02	4.152E-03	3.432E-02	14	Annexin VIII, KLC2, CRK, HBG, p38 MAPK, ERK1 (MAPK3), Kinesin light chain, ERK1/2, GRB2, HBGA, ANXA8L2, Dysbindin, Guanylate cyclase, Adult hemoglobin
388	cellular process	16599	4.167E-03	3.435E-02	4.167E-03	3.435E-02	169	MST3, Neurochondrin, SAE1, Annexin VIII, PEX2, KLC2, MUC15, LAGY, Reticulon 4, ZNF261, MIBP, Siglec-8, DLC1 (Dynein LC8a), LCORL, GDP-mannose 4,6 dehydratase, TIPIN, SCMH1, CRK, RNASE6, DCTN2, PPP1R12B, VAMP5, Calpain 1(mu), STAT5A, SPRR1A, TIRAP (Mal), RAD1, TCL1A, p38 MAPK, NASP, ERK1 (MAPK3), Carbonic anhydrase XIII, Bim, LASP1, Metallothionein-I, CERT, STAT5, EFHD1, RBP-J kappa (CBF1), FERD3L, RBP1, SMIF, Twinfilin, MCT4, BMAL2, Autophagin-1, NHS, Tex1, UCK1, KLC3, RPS27A, UIP5, p38gamma (MAPK12), ZNF397, Ccdc109a, Ubiquitin, IMD2, CDC4L, EYA1, PhLP, SNX9, Kinesin light chain, NANP, DUPD1, XPF, Phosphatase regulator (inhibitor), PDXP, LYPLAL1, ATP1A3, Fatty acid-binding protein, ERK1/2, NIP2, ADSL, ARHGEF10, MYLK1, CRABP2, Metallothionein-IG, PQBP-1, Kizuna (C20orf19),

								LCKBP1, SPRR4, Guanylate cyclase A (NPR1), NEPH2, MOCS2(small), GA17, CrkL, Ajuba, GRB2, Rab-9B, PTP-2, CNK1, PEX19, HXK4, DDC, RIFK, Rap1GDS1, BBS9, Metallothionein-II, ZNF655, Sen15, Tropomodulin, COG3, HMBS, RRP42, UBC, Telokin, PCP2, ST13 (Hip), Troponin C, skeletal muscle, CED-6, C16orf75, GBP5, NUP93, HKR1, ANXA8L2, Siglec-E, Dysbindin, Notch, ACSBG1, RAD23A, UBB, BMF, PAP41, IRX4, Guanylate cyclase, IGHG1, ARID4A, Cyclophilin E, CALCOCO2, NOTCH1 precursor, GCK(MAP4K2), 4E-BP1, RRAD, Sprr2f, SCML1, DBC1, Metallothionein-1M, GINS2, USP5, AO7, RAB24, IL1RN, CTP synthase II, RPA4, ZNF397OS, BMP1, DYNLL, DSS1, Occludin, WWOX, E-FABP, DKK3, ACP33, PYDC1 (POP1), Adult hemoglobin, PAIP1, MLX, CAMK2N1, Rich1, Melusin, SAP, p47, APOBEC-1, RIP, SPRR3, MLCK, PAF1, Galectin-13, DOK3
389	smooth muscle tissue development	35	4.180E-03	3.438E-02	4.180E-03	3.438E-02	3	MYLK1, Telokin, MLCK
390	connective tissue development	274	4.257E-03	3.487E-02	4.257E-03	3.487E-02	8	p38 MAPK, ERK1 (MAPK3), Ubiquitin, ERK1/2, Notch, UBB, NOTCH1 precursor, BMP1
391	cellular developmental process	4128	4.262E-03	3.487E-02	4.262E-03	3.487E-02	54	MST3, Neurochondrin, PEX2, LAGY, Reticulon 4, VAMP5, STAT5A, SPRR1A, TIRAP (Mal), TCL1A, p38 MAPK, ERK1 (MAPK3), Bim, Metallothionein-I, CERT, STAT5, EFHD1, RBP-J kappa (CBF1), FERD3L, NHS, p38gamma (MAPK12), Ubiquitin, EYA1, Phosphatase regulator (inhibitor), Fatty acid-binding protein, ERK1/2, NIP2, ARHGEF10, Metallothionein-IG, PQBP-1, LCKBP1, SPRR4, NEPH2, GRB2, PTP-2, BBS9, Metallothionein-II, Tropomodulin, HMBS, Dysbindin, Notch, UBB, IRX4, ARID4A, NOTCH1 precursor, Sprr2f, BMP1, DYNLL, WWOX, Adult hemoglobin, RIP, SPRR3, MLCK, PAF1
392	regulation of cellular component	820	4.3	3.5	4.3	3.5	16	MST3, Reticulon 4, CRK, TIRAP (Mal), p38 MAPK, STAT5, RBP-J kappa (CBF1), ERK1/2, MYLK1, Ajuba, PTP-2, Telokin, Notch, NOTCH1 precursor, IL1RN, MLCK

	movement		37E-03	36E-02	37E-03	36E-02		
393	cell cycle checkpoint	275	4.351E-03	3.536E-02	4.351E-03	3.536E-02	8	TIPIN, RAD1, p38 MAPK, RPS27A, Ubiquitin, UBC, UBB, RPA4
394	metabolic process	11562	4.417E-03	3.536E-02	4.417E-03	3.536E-02	126	MST3, SAE1, PEX2, KLC2, MUC15, LAGY, MIBP, DLC1 (Dynein LC8a), LCORL, GDP-mannose 4,6 dehydratase, TIPIN, SCMH1, CRK, RNASE6, DCTN2, Calpain 1(mu), STAT5A, SPRR1A, TIRAP (Mal), RAD1, p38 MAPK, NASP, ERK1 (MAPK3), Carbonic anhydrase XIII, Bim, CERT, STAT5, RBP-J kappa (CBF1), FERD3L, RBP1, SMIF, Twinfilin, BMAL2, Autophagin-1, Tex1, UCK1, KLC3, RPS27A, UIP5, p38gamma (MAPK12), ZNF397, Ubiquitin, IMD2, EYA1, PhLP, Kinesin light chain, NANP, DUPD1, XPF, Phosphatase regulator (inhibitor), PDXP, LYPLAL1, ATP1A3, Fatty acid-binding protein, ERK1/2, ADSL, MYLK1, CRABP2, PQBP-1, SPRR4, Guanylate cyclase A (NPR1), MOCS2(small), GA17, CrkL, Ajuba, GRB2, PTP-2, CNK1, PEX19, HXK4, Grancalcin, DDC, RIFK, ZNF655, Sen15, COG3, HMBS, RRP42, UBC, Telokin, ST13 (Hip), C16orf75, GBP5, NUP93, HKR1, Notch, ACSBG1, RAD23A, UBB, PAP41, Guanylate cyclase, IGHG1, ARID4A, Cyclophilin E, NOTCH1 precursor, GCK(MAP4K2), 4E-BP1, RRAD, DHRS10, SCML1, DBC1, GINS2, N6amt2, USP5, AO7, RAB24, IL1RN, CTP synthase II, RPA4, ZNF397OS, BMP1, DYNLL, DSS1, Occludin, WWOX, E-FABP, Adult hemoglobin, GOCAP1, PAIP1, MLX, APOBEC-1, RIP, SPRR3, MLCK, PAF1, Galectin-13
395	positive regulation of protein kinase activity	604	4.448E-03	3.536E-02	4.448E-03	3.536E-02	13	CRK, TIRAP (Mal), p38 MAPK, ERK1 (MAPK3), RPS27A, Ubiquitin, ERK1/2, CrkL, Ajuba, UBC, UBB, GCK(MAP4K2), IL1RN

396	myosin filament assembly	11	4.490E-03	3.536E-02	4.490E-03	3.536E-02	2	Rap1GDS1, Tropomodulin
397	regulation of histone phosphorylation	11	4.490E-03	3.536E-02	4.490E-03	3.536E-02	2	ERK1 (MAPK3), ERK1/2
398	cell migration involved in heart development	11	4.490E-03	3.536E-02	4.490E-03	3.536E-02	2	Notch, NOTCH1 precursor
399	mitral valve development	11	4.490E-03	3.536E-02	4.490E-03	3.536E-02	2	Notch, NOTCH1 precursor
400	cardiac endothelial cell differentiation	11	4.490E-03	3.536E-02	4.490E-03	3.536E-02	2	Notch, NOTCH1 precursor
40	myosin filament organization	11	4.	3.	4.	3.	2	Rap1GDS1, Tropomodulin

1			490E-03	536E-02	490E-03	536E-02		
402	diterpenoid biosynthetic process	11	4.490E-03	3.536E-02	4.490E-03	3.536E-02	2	RBP1, CRABP2
403	negative regulation of stem cell differentiation	11	4.490E-03	3.536E-02	4.490E-03	3.536E-02	2	Notch, NOTCH1 precursor
404	mesangial cell development	11	4.490E-03	3.536E-02	4.490E-03	3.536E-02	2	Notch, NOTCH1 precursor
405	glomerular mesangial cell differentiation	11	4.490E-03	3.536E-02	4.490E-03	3.536E-02	2	Notch, NOTCH1 precursor
406	positive regulation of endothelial cell differentiation	11	4.49	3.53	4.49	3.53	2	Notch, NOTCH1 precursor

			0E-03	6E-02	0E-03	6E-02		
407	negative regulation of catalytic activity	975	4.499E-03	3.536E-02	4.499E-03	3.536E-02	18	Annexin VIII, DLC1 (Dynein LC8a), RPS27A, Ubiquitin, Phosphatase regulator (inhibitor), Fatty acid-binding protein, Ajuba, UBC, ANXA8L2, Notch, UBB, PAP41, NOTCH1 precursor, DBC1, DYNLL, Cystatin B, PYDC1 (POP1), CAMK2N1
408	developmental programmed cell death	36	4.529E-03	3.551E-02	4.529E-03	3.551E-02	3	Bim, Notch, NOTCH1 precursor
409	antigen processing and presentation	338	4.540E-03	3.551E-02	4.540E-03	3.551E-02	9	KLC2, DLC1 (Dynein LC8a), DCTN2, RPS27A, Ubiquitin, Kinesin light chain, UBC, UBB, DYNLL
410	regulation of phosphorylation	1539	4.618E-03	3.603E-02	4.618E-03	3.603E-02	25	DLC1 (Dynein LC8a), CRK, TIRAP (Mal), p38 MAPK, ERK1 (MAPK3), Twinfilin, RPS27A, Ubiquitin, Fatty acid-binding protein, ERK1/2, NIP2, LCKBP1, CrkL, Ajuba, GRB2, HXK4, UBC, UBB, GCK(MAP4K2), IL1RN, DYNLL, PYDC1 (POP1), Adult hemoglobin, CAMK2N1, MLCK
411	response to osmotic stress	116	4.635E	3.608E	4.635E	3.608E	5	p38 MAPK, MYLK1, Telokin, APOBEC-1, MLCK

			-03	-02	-03	-02		
412	developmental process	6399	4.695E-03	3.646E-02	4.695E-03	3.646E-02	77	MST3, Neurochondrin, PEX2, LAGY, Reticulon 4, ZNF261, DLC1 (Dynein LC8a), SCMH1, Beta crystallin B2, VAMP5, STAT5A, SPRR1A, TIRAP (Mal), RAD1, TCL1A, p38 MAPK, NASP, ERK1 (MAPK3), Bim, Metallothionein-I, CERT, STAT5, EFHD1, RBP-J kappa (CBF1), FERD3L, NHS, p38gamma (MAPK12), Ubiquitin, IMD2, EYA1, Phosphatase regulator (inhibitor), Fatty acid-binding protein, ERK1/2, NIP2, ARHGEF10, MYLK1, CRABP2, Metallothionein-IG, PQBP-1, LCKBP1, SPRR4, NEPH2, CrkL, Ajuba, GRB2, PTP-2, HXK4, DDC, BBS9, Metallothionein-II, Tropomodulin, HMBS, Telokin, HKR1, Dysbindin, Notch, ACSBG1, UBB, PAP41, IRX4, Guanylate cyclase, ARID4A, NOTCH1 precursor, 4E-BP1, Sprr2f, SCML1, BMP1, DYNLL, WWOX, E-FABP, DKK3, Adult hemoglobin, Melusin, RIP, SPRR3, MLCK, PAF1
413	peptidyl-serine phosphorylation	117	4.806E-03	3.707E-02	4.806E-03	3.707E-02	5	p38 MAPK, ERK1 (MAPK3), p38gamma (MAPK12), ERK1/2, MLCK
414	positive regulation of phosphorus metabolic process	1138	4.814E-03	3.707E-02	4.814E-03	3.707E-02	20	CRK, TIRAP (Mal), p38 MAPK, ERK1 (MAPK3), RPS27A, Ubiquitin, Fatty acid-binding protein, ERK1/2, NIP2, ARHGEF10, LCKBP1, Guanylate cyclase A (NPR1), CrkL, Ajuba, HXK4, UBC, UBB, Guanylate cyclase, GCK(MAP4K2), IL1RN
415	positive regulation of phosphate metabolic process	1138	4.814E-03	3.707E-02	4.814E-03	3.707E-02	20	CRK, TIRAP (Mal), p38 MAPK, ERK1 (MAPK3), RPS27A, Ubiquitin, Fatty acid-binding protein, ERK1/2, NIP2, ARHGEF10, LCKBP1, Guanylate cyclase A (NPR1), CrkL, Ajuba, HXK4, UBC, UBB, Guanylate cyclase, GCK(MAP4K2), IL1RN
41	positive regulation of NF-kappaB transcription	16	4.	3.	4.	3.	6	TIRAP (Mal), RPS27A, Ubiquitin, UBC, UBB, AO7

6	factor activity	7	821E-03	707E-02	821E-03	707E-02		
417	regulation of biological quality	3588	4.852E-03	3.722E-02	4.852E-03	3.722E-02	48	Neurochondrin, Annexin VIII, PEX2, KLC2, Reticulon 4, DLC1 (Dynein LC8a), CRK, HBG, VAMP5, STAT5A, TIRAP (Mal), p38 MAPK, ERK1 (MAPK3), Bim, Metallothionein-I, CERT, STAT5, RBP1, Twinfilin, Ccdc109a, Ubiquitin, Kinesin light chain, XPF, PDXP, Fatty acid-binding protein, ERK1/2, CRABP2, LCKBP1, Guanylate cyclase A (NPR1), GRB2, PEX19, HXK4, Rap1GDS1, Metallothionein-II, COG3, UBC, HBGA, ANXA8L2, Dysbindin, UBB, Guanylate cyclase, ARID4A, IL1RN, DYNLL, Adult hemoglobin, PAIP1, APOBEC-1, MLCK
418	peptide cross-linking	37	4.895E-03	3.746E-02	4.895E-03	3.746E-02	3	SPRR1A, SPRR4, SPRR3
419	organic cyclic compound metabolic process	6014	5.031E-03	3.841E-02	5.031E-03	3.841E-02	73	PEX2, LAGY, MIBP, DLC1 (Dynein LC8a), LCORL, GDP-mannose 4,6 dehydratase, TIPIN, SCMH1, RNASE6, STAT5A, RAD1, p38 MAPK, NASP, ERK1 (MAPK3), STAT5, RBP-J kappa (CBF1), FERD3L, SMIF, BMAL2, Tex1, UCK1, RPS27A, p38gamma (MAPK12), ZNF397, Ubiquitin, IMD2, EYA1, XPF, Phosphatase regulator (inhibitor), PDXP, ATP1A3, ERK1/2, ADSL, PQBP-1, Guanylate cyclase A (NPR1), MOCS2(small), Ajuba, HXK4, DDC, RIFK, ZNF655, Sen15, HMBS, RRP42, UBC, C16orf75, GBP5, HKR1, Notch, RAD23A, UBB, PAP41, Guanylate cyclase, ARID4A, Cyclophilin E, NOTCH1 precursor, RRAD, DHRS10, SCML1, DBC1, GINS2, CTP synthase II, RPA4, ZNF397OS, DYNLL, DSS1, Occludin, WWOX, GOCAP1, PAIP1, MLX, APOBEC-1, PAF1
420	negative regulation of G1/S transition of mitotic cell cycle	119	5.162E-0	3.913E-0	5.162E-0	3.913E-0	5	RPS27A, Ubiquitin, ZNF655, UBC, UBB

			3	2	3	2		
421	mitotic DNA damage checkpoint	119	5.162E-03	3.913E-02	5.162E-03	3.913E-02	5	TIPIN, RPS27A, Ubiquitin, UBC, UBB
422	negative regulation of cell cycle G1/S phase transition	119	5.162E-03	3.913E-02	5.162E-03	3.913E-02	5	RPS27A, Ubiquitin, ZNF655, UBC, UBB
423	response to organic substance	3414	5.184E-03	3.920E-02	5.184E-03	3.920E-02	46	Reticulon 4, CRK, STAT5A, TIRAP (Mal), p38 MAPK, ERK1 (MAPK3), Bim, Metallothionein-I, STAT5, RBP1, SMIF, RPS27A, p38gamma (MAPK12), Ubiquitin, IMD2, Phosphatase regulator (inhibitor), PDXP, Fatty acid-binding protein, ERK1/2, Metallothionein-IG, LCKBP1, CrkL, GRB2, PTP-2, HXK4, DDC, Metallothionein-II, HMBS, UBC, GBP5, NUP93, Notch, ACSBG1, UBB, Guanylate cyclase, CALCOCO2, NOTCH1 precursor, 4E-BP1, Sprr2f, IL1RN, WWOX, PYDC1 (POP1), Adult hemoglobin, APOBEC-1, MLCK, PAF1
424	negative regulation of RNA biosynthetic process	1226	5.199E-03	3.923E-02	5.199E-03	3.923E-02	21	PEX2, LAGY, SCMH1, HBG, RBP-J kappa (CBF1), FERD3L, RPS27A, Ubiquitin, Fatty acid-binding protein, LCKBP1, Ajuba, UBC, HBGA, Notch, UBB, ARID4A, NOTCH1 precursor, DKK3, Adult hemoglobin, MLX, PAF1
425	cytokine secretion	38	5.279E-0	3.929E-0	5.279E-0	3.929E-0	3	RBP-J kappa (CBF1), Notch, NOTCH1 precursor

			3	2	3	2		
426	primary metabolic process	10185	5.282E-03	3.929E-02	5.282E-03	3.929E-02	113	MST3, SAE1, PEX2, MUC15, LAGY, MIBP, DLC1 (Dynein LC8a), LCORL, GDP-mannose 4,6 dehydratase, TIPIN, SCMH1, RNASE6, Calpain 1(mu), STAT5A, SPRR1A, RAD1, p38 MAPK, NASP, ERK1 (MAPK3), Bim, CERT, STAT5, RBP-J kappa (CBF1), FERD3L, RBP1, SMIF, Twinfilin, BMAL2, Autophagin-1, Tex1, UCK1, RPS27A, UIP5, p38gamma (MAPK12), ZNF397, Ubiquitin, IMD2, EYA1, PhLP, NANP, DUPD1, XPF, Phosphatase regulator (inhibitor), PDXP, LYPLAL1, ATP1A3, Fatty acid-binding protein, ERK1/2, ADSL, MYLK1, CRABP2, PQBP-1, SPRR4, Guanylate cyclase A (NPR1), MOCS2(small), GA17, Ajuba, PTP-2, PEX19, HXK4, Grancalcin, DDC, RIFK, ZNF655, Sen15, COG3, HMBS, RRP42, UBC, Telokin, ST13 (Hip), C16orf75, GBP5, NUP93, HKR1, Notch, ACSBG1, RAD23A, UBB, PAP41, Guanylate cyclase, IGHG1, ARID4A, Cyclophilin E, NOTCH1 precursor, GCK(MAP4K2), 4E-BP1, RRAD, DHRS10, SCML1, DBC1, GINS2, USP5, AO7, IL1RN, CTP synthase II, RPA4, ZNF397OS, BMP1, DYNLL, DSS1, Occludin, WWOX, E-FABP, Adult hemoglobin, GOCAP1, PAIP1, MLX, APOBEC-1, SPRR3, MLCK, PAF1, Galectin-13
427	regulation of biological process	12391	5.305E-03	3.929E-02	5.305E-03	3.929E-02	133	MST3, Neurochondrin, SAE1, Annexin VIII, PEX2, LAGY, Reticulon 4, ZNF261, MIBP, Siglec-8, DLC1 (Dynein LC8a), LCORL, GDP-mannose 4,6 dehydratase, TIPIN, SCMH1, CRK, HBG, PPP1R12B, Calpain 1(mu), STAT5A, TIRAP (Mal), TCL1A, GGA1, p38 MAPK, IL-1F5, ERK1 (MAPK3), Bim, LASP1, Metallothionein-I, CERT, STAT5, RBP-J kappa (CBF1), FERD3L, RBP1, SMIF, Twinfilin, BMAL2, Autophagin-1, RPS27A, p38gamma (MAPK12), ZNF397, Ccdc109a, Ubiquitin, CDC4L, EYA1, PhLP, SNX9, SCOCO, XPF, Phosphatase regulator (inhibitor), PDXP, LYPLAL1, ATP1A3, Fatty acid-binding protein, ERK1/2, NIP2, ARHGEF10, MYLK1, CRABP2, Metallothionein-IG, PQBP-1, LCKBP1, Guanylate cyclase A (NPR1), GA17, CrkL, Ajuba, GRB2, Rab-9B, PTP-2, CNK1, PEX19, HXK4, RIFK, Rap1GDS1, Metallothionein-II, ZNF655, COG3, UBC, HBGA, Telokin, SNX1, PCP2, ST13 (Hip), Troponin C, skeletal muscle, CED-6, NUP93, HKR1, ANXA8L2, Siglec-E, Dysbindin, Notch, RAD23A, UBB, BMF, PAP41, IRX4, Guanylate cyclase, IGHG1, ARID4A, Cyclophilin E, NOTCH1 precursor, GCK(MAP4K2), 4E-BP1, RRAD, SCML1, DBC1, Metallothionein-1M, USP5, AO7, GGA, RAB24, IL1RN, FAM116A, ZNF397OS, BMP1, DYNLL, Cystatin B, WWOX, DKK3, ACP33, PYDC1 (POP1), Adult hemoglobin, PAIP1, MLX, CAMK2N1, Rich1, Melusin, SAP, APOBEC-1, RIP, MLCK, PAF1, DOK3

428	mammary gland involution	12	5.355E-03	3.929E-02	5.355E-03	3.929E-02	2	STAT5A, STAT5
429	regulation of gamma-delta T cell differentiation	12	5.355E-03	3.929E-02	5.355E-03	3.929E-02	2	STAT5A, STAT5
430	atrioventricular valve morphogenesis	12	5.355E-03	3.929E-02	5.355E-03	3.929E-02	2	Notch, NOTCH1 precursor
431	positive regulation of natural killer cell differentiation	12	5.355E-03	3.929E-02	5.355E-03	3.929E-02	2	STAT5A, STAT5
432	oxaloacetate metabolic process	12	5.355E-03	3.929E-02	5.355E-03	3.929E-02	2	STAT5A, STAT5
43	regulation of somitogenesis	12	5.	3.	5.	3.	2	Notch, NOTCH1 precursor

3			355E-03	929E-02	355E-03	929E-02		
434	mesangial cell differentiation	12	5.355E-03	3.929E-02	5.355E-03	3.929E-02	2	Notch, NOTCH1 precursor
435	renal interstitial cell development	12	5.355E-03	3.929E-02	5.355E-03	3.929E-02	2	Notch, NOTCH1 precursor
436	creatine metabolic process	12	5.355E-03	3.929E-02	5.355E-03	3.929E-02	2	STAT5A, STAT5
437	cellular protein modification process	2690	5.420E-03	3.959E-02	5.420E-03	3.959E-02	38	MST3, SAE1, MUC15, LAGY, STAT5A, SPRR1A, p38 MAPK, ERK1 (MAPK3), CERT, STAT5, Twinfilin, RPS27A, UIP5, p38gamma (MAPK12), Ubiquitin, EYA1, DUPD1, PDXP, LYPLAL1, ERK1/2, MYLK1, SPRR4, Guanylate cyclase A (NPR1), PTP-2, COG3, HMBS, UBC, Telokin, UBB, Guanylate cyclase, ARID4A, Cyclophilin E, GCK(MAP4K2), USP5, AO7, SPRR3, MLCK, PAF1
438	protein modification process	2690	5.42	3.95	5.42	3.95	38	MST3, SAE1, MUC15, LAGY, STAT5A, SPRR1A, p38 MAPK, ERK1 (MAPK3), CERT, STAT5, Twinfilin, RPS27A, UIP5, p38gamma (MAPK12), Ubiquitin, EYA1, DUPD1, PDXP, LYPLAL1, ERK1/2, MYLK1, SPRR4, Guanylate cyclase A

			0E-03	9E-02	0E-03	9E-02		(NPR1), PTP-2, COG3, HMBS, UBC, Telokin, UBB, Guanylate cyclase, ARID4A, Cyclophilin E, GCK(MAP4K2), USP5, AO7, SPRR3, MLCK, PAF1
439	regulation of transforming growth factor beta receptor signaling pathway	121	5.536E-03	4.034E-02	5.536E-03	4.034E-02	5	RPS27A, Ubiquitin, Phosphatase regulator (inhibitor), UBC, UBB
440	aromatic compound biosynthetic process	3336	5.622E-03	4.087E-02	5.622E-03	4.087E-02	45	LAGY, MIBP, DLC1 (Dynein LC8a), LCORL, GDP-mannose 4,6 dehydratase, SCMH1, STAT5A, p38 MAPK, ERK1 (MAPK3), STAT5, RBP-J kappa (CBF1), FERD3L, BMAL2, UCK1, RPS27A, p38gamma (MAPK12), ZNF397, Ubiquitin, IMD2, EYA1, Phosphatase regulator (inhibitor), ATP1A3, ERK1/2, ADSL, PQBP-1, Guanylate cyclase A (NPR1), Ajuba, DDC, RIFK, ZNF655, HMBS, UBC, HKR1, Notch, UBB, PAP41, Guanylate cyclase, ARID4A, NOTCH1 precursor, SCML1, CTP synthase II, ZNF397OS, DYNLL, MLX, PAF1
441	negative regulation of nucleobase-containing compound metabolic process	1398	5.647E-03	4.096E-02	5.647E-03	4.096E-02	23	PEX2, LAGY, TIPIN, SCMH1, HBG, RBP-J kappa (CBF1), FERD3L, RPS27A, Ubiquitin, XPF, Fatty acid-binding protein, LCKBP1, Ajuba, UBC, HBGA, Notch, UBB, ARID4A, NOTCH1 precursor, DKK3, Adult hemoglobin, MLX, PAF1
442	cellular protein metabolic process	3617	5.670E-03	4.104E-02	5.670E-03	4.104E-02	48	MST3, SAE1, MUC15, LAGY, STAT5A, SPRR1A, p38 MAPK, ERK1 (MAPK3), CERT, STAT5, Twinfilin, RPS27A, UIP5, p38gamma (MAPK12), Ubiquitin, EYA1, PhLP, DUPD1, PDXP, LYPLAL1, ERK1/2, MYLK1, SPRR4, Guanylate cyclase A (NPR1), MOCS2(small), GA17, PTP-2, PEX19, COG3, HMBS, UBC, Telokin, ST13 (Hip), Notch, RAD23A, UBB, Guanylate cyclase, ARID4A, Cyclophilin E, NOTCH1 precursor, GCK(MAP4K2), 4E-BP1, USP5, AO7, PAIP1, SPRR3, MLCK, PAF1
443	negative regulation of cellular response to growth factor stimulus	122	5.730	4.138	5.730	4.138	5	RPS27A, Ubiquitin, Phosphatase regulator (inhibitor), UBC, UBB

			E-03	E-02	E-03	E-02		
444	regulation of protein serine/threonine kinase activity	553	5.812E-03	4.170E-02	5.812E-03	4.170E-02	12	CRK, TIRAP (Mal), p38 MAPK, ERK1 (MAPK3), RPS27A, Ubiquitin, ERK1/2, Ajuba, UBC, UBB, GCK(MAP4K2), IL1RN
445	single-organism developmental process	6348	5.813E-03	4.170E-02	5.813E-03	4.170E-02	76	MST3, Neurochondrin, PEX2, LAGY, Reticulon 4, ZNF261, DLC1 (Dynein LC8a), SCMH1, Beta crystallin B2, VAMP5, STAT5A, SPRR1A, TIRAP (Mal), RAD1, TCL1A, p38 MAPK, NASP, ERK1 (MAPK3), Bim, Metallothionein-I, CERT, STAT5, EFHD1, RBP-J kappa (CBF1), FERD3L, NHS, p38gamma (MAPK12), Ubiquitin, IMD2, EYA1, Phosphatase regulator (inhibitor), Fatty acid-binding protein, ERK1/2, NIP2, ARHGEF10, MYLK1, CRABP2, Metallothionein-IG, PQBP-1, LCKBP1, SPRR4, NEPH2, CrkL, Ajuba, GRB2, PTP-2, HXK4, DDC, BBS9, Metallothionein-II, Tropomodulin, HMBS, Telokin, HKR1, Dysbindin, Notch, ACSBG1, UBB, PAP41, IRX4, Guanylate cyclase, ARID4A, NOTCH1 precursor, 4E-BP1, Sprr2f, BMP1, DYNLL, WWOX, E-FABP, DKK3, Adult hemoglobin, Melusin, RIP, SPRR3, MLCK, PAF1
446	negative regulation of ubiquitin-protein ligase activity involved in mitotic cell cycle	77	5.813E-03	4.170E-02	5.813E-03	4.170E-02	4	RPS27A, Ubiquitin, UBC, UBB
447	regulation of cell migration	698	5.911E-03	4.230E-02	5.911E-03	4.230E-02	14	MST3, Reticulon 4, TIRAP (Mal), p38 MAPK, RBP-J kappa (CBF1), ERK1/2, MYLK1, Ajuba, PTP-2, Telokin, Notch, NOTCH1 precursor, IL1RN, MLCK

448	regulation of cellular macromolecule biosynthetic process	492	6.028E-03	4.305E-02	6.028E-03	4.305E-02	55	PEX2, LAGY, DLC1 (Dynein LC8a), LCORL, TIPIN, SCMH1, CRK, HBG, STAT5A, TIRAP (Mal), p38 MAPK, ERK1 (MAPK3), STAT5, RBP-J kappa (CBF1), FERD3L, SMIF, BMAL2, RPS27A, p38gamma (MAPK12), ZNF397, Ubiquitin, EYA1, Phosphatase regulator (inhibitor), Fatty acid-binding protein, ERK1/2, CRABP2, PQBP-1, LCKBP1, GA17, Ajuba, HXK4, ZNF655, UBC, HBGA, HKR1, Notch, UBB, IRX4, ARID4A, Cyclophilin E, NOTCH1 precursor, 4E-BP1, SCML1, DBC1, AO7, ZNF397OS, DYNLL, WWOX, DKK3, PYDC1 (POP1), Adult hemoglobin, PAIP1, MLX, APOBEC-1, PAF1
449	regulation of organ formation	40	6.099E-03	4.336E-02	6.099E-03	4.336E-02	3	EYA1, Notch, NOTCH1 precursor
450	response to copper ion	40	6.099E-03	4.336E-02	6.099E-03	4.336E-02	3	Metallothionein-I, Metallothionein-IG, Metallothionein-II
451	cellular response to fibroblast growth factor stimulus	232	6.172E-03	4.378E-02	6.172E-03	4.378E-02	7	ERK1 (MAPK3), RPS27A, Ubiquitin, ERK1/2, GRB2, UBC, UBB
452	positive regulation of catalytic activity	1493	6.247E-03	4.394E-02	6.247E-03	4.394E-02	24	SAE1, CRK, PPP1R12B, TIRAP (Mal), p38 MAPK, ERK1 (MAPK3), Bim, RPS27A, Ubiquitin, SNX9, Phosphatase regulator (inhibitor), Fatty acid-binding protein, ERK1/2, NIP2, CrkL, Ajuba, RIFK, Rap1GDS1, UBC, UBB, GCK(MAP4K2), IL1RN, Rich1, RIP

453	neuronal stem cell maintenance	13	6.291E-03	4.394E-02	6.291E-03	4.394E-02	2	Notch, NOTCH1 precursor
454	negative regulation of calcium ion-dependent exocytosis	13	6.291E-03	4.394E-02	6.291E-03	4.394E-02	2	Notch, NOTCH1 precursor
455	renal interstitial cell differentiation	13	6.291E-03	4.394E-02	6.291E-03	4.394E-02	2	Notch, NOTCH1 precursor
456	positive regulation of transcription from RNA polymerase II promoter in response to stress	13	6.291E-03	4.394E-02	6.291E-03	4.394E-02	2	Notch, NOTCH1 precursor
457	regulation of epithelial cell proliferation involved in prostate gland development	13	6.291E-03	4.394E-02	6.291E-03	4.394E-02	2	Notch, NOTCH1 precursor
45	positive regulation of gamma-delta T cell activation	13	6.	4.	6.	4.	2	STAT5A, STAT5

8			2.91E-03	3.94E-02	2.91E-03	3.94E-02		
459	DNA damage response, signal transduction by p53 class mediator resulting in cell cycle arrest	79	6.362E-03	4.405E-02	6.362E-03	4.405E-02	4	RPS27A, Ubiquitin, UBC, UBB
460	signal transduction involved in mitotic G1 DNA damage checkpoint	79	6.362E-03	4.405E-02	6.362E-03	4.405E-02	4	RPS27A, Ubiquitin, UBC, UBB
461	prostate gland development	79	6.362E-03	4.405E-02	6.362E-03	4.405E-02	4	STAT5A, STAT5, Notch, NOTCH1 precursor
462	intracellular signal transduction involved in G1 DNA damage checkpoint	79	6.362E-03	4.405E-02	6.362E-03	4.405E-02	4	RPS27A, Ubiquitin, UBC, UBB
463	positive regulation of kinase activity	632	6.44	4.45	6.44	4.45	13	CRK, TIRAP (Mal), p38 MAPK, ERK1 (MAPK3), RPS27A, Ubiquitin, ERK1/2, CrkL, Ajuba, UBC, UBB, GCK(MAP4K2), IL1RN

			1E-03	0E-02	1E-03	0E-02		
464	cellular response to transforming growth factor beta stimulus	234	6.459E-03	4.450E-02	6.459E-03	4.450E-02	7	SMIF, RPS27A, Ubiquitin, Phosphatase regulator (inhibitor), UBC, UBB, WWOX
465	regulation of nucleobase-containing compound metabolic process	4986	6.531E-03	4.450E-02	6.531E-03	4.450E-02	62	PEX2, LAGY, DLC1 (Dynein LC8a), LCORL, TIPIN, SCMH1, CRK, HBG, STAT5A, TIRAP (Mal), p38 MAPK, ERK1 (MAPK3), STAT5, RBP-J kappa (CBF1), FERD3L, SMIF, BMAL2, RPS27A, p38gamma (MAPK12), ZNF397, Ubiquitin, EYA1, SNX9, XPF, Phosphatase regulator (inhibitor), Fatty acid-binding protein, ERK1/2, NIP2, ARHGEF10, CRABP2, PQBP-1, LCKBP1, Guanylate cyclase A (NPR1), Ajuba, Rap1GDS1, ZNF655, UBC, HBGA, PCP2, HKR1, Notch, UBB, IRX4, Guanylate cyclase, ARID4A, Cyclophilin E, NOTCH1 precursor, SCML1, DBC1, AO7, FAM116A, ZNF397OS, DYNLL, WWOX, DKK3, PYDC1 (POP1), Adult hemoglobin, MLX, Rich1, APOBEC-1, RIP, PAF1
466	dopamine metabolic process	41	6.536E-03	4.450E-02	6.536E-03	4.450E-02	3	Guanylate cyclase A (NPR1), DDC, Guanylate cyclase
467	nitric oxide mediated signal transduction	41	6.536E-03	4.450E-02	6.536E-03	4.450E-02	3	Metallothionein-I, Metallothionein-II, Guanylate cyclase
46	lipopolysaccharide-mediated signaling pathway	41	6.	4.	6.	4.	3	p38 MAPK, ERK1 (MAPK3), ERK1/2

8			536E-03	450E-02	536E-03	450E-02		
469	antigen receptor-mediated signaling pathway	178	6.540E-03	4.450E-02	6.540E-03	4.450E-02	6	RPS27A, Ubiquitin, ERK1/2, UBC, UBB, ACP33
470	mitotic DNA integrity checkpoint	126	6.554E-03	4.450E-02	6.554E-03	4.450E-02	5	TIPIN, RPS27A, Ubiquitin, UBC, UBB
471	epidermal growth factor receptor signaling pathway	235	6.606E-03	4.450E-02	6.606E-03	4.450E-02	7	ERK1 (MAPK3), RPS27A, Ubiquitin, ERK1/2, GRB2, UBC, UBB
472	signal transduction involved in mitotic DNA integrity checkpoint	80	6.649E-03	4.450E-02	6.649E-03	4.450E-02	4	RPS27A, Ubiquitin, UBC, UBB
473	signal transduction involved in mitotic cell cycle checkpoint	80	6.64	4.45	6.64	4.45	4	RPS27A, Ubiquitin, UBC, UBB

			9E-03	0E-02	9E-03	0E-02		
474	signal transduction involved in DNA damage checkpoint	80	6.649E-03	4.450E-02	6.649E-03	4.450E-02	4	RPS27A, Ubiquitin, UBC, UBB
475	positive regulation of mitochondrion organization	80	6.649E-03	4.450E-02	6.649E-03	4.450E-02	4	DLC1 (Dynein LC8a), Bim, BMF, DYNLL
476	signal transduction involved in DNA integrity checkpoint	80	6.649E-03	4.450E-02	6.649E-03	4.450E-02	4	RPS27A, Ubiquitin, UBC, UBB
477	activation of MAPKK activity	80	6.649E-03	4.450E-02	6.649E-03	4.450E-02	4	CRK, ERK1 (MAPK3), ERK1/2, CrkL
478	signal transduction involved in mitotic DNA damage checkpoint	80	6.649E	4.450E	6.649E	4.450E	4	RPS27A, Ubiquitin, UBC, UBB

			-03	-02	-03	-02		
479	positive regulation of cellular component organization	936	6.694E-03	4.468E-02	6.694E-03	4.468E-02	17	MST3, DLC1 (Dynein LC8a), p38 MAPK, ERK1 (MAPK3), Bim, SNX9, PDXP, ERK1/2, ARHGEF10, LCKBP1, Ajuba, GRB2, Notch, BMF, NOTCH1 precursor, DYNLL, MLCK
480	regulation of protein complex assembly	296	6.705E-03	4.468E-02	6.705E-03	4.468E-02	8	Bim, Twinfilin, SNX9, LCKBP1, Ajuba, GRB2, BMF, 4E-BP1
481	response to fibroblast growth factor	236	6.756E-03	4.493E-02	6.756E-03	4.493E-02	7	ERK1 (MAPK3), RPS27A, Ubiquitin, ERK1/2, GRB2, UBC, UBB
482	single-organism cellular process	13479	6.825E-03	4.529E-02	6.825E-03	4.529E-02	142	MST3, Neurochondrin, PEX2, KLC2, MUC15, LAGY, Reticulon 4, MIBP, Siglec-8, DLC1 (Dynein LC8a), GDP-mannose 4,6 dehydratase, TIPIN, SCMH1, CRK, DCTN2, PPP1R12B, VAMP5, Calpain 1(mu), STAT5A, SPRR1A, TIRAP (Mal), RAD1, TCL1A, p38 MAPK, NASP, ERK1 (MAPK3), Carbonic anhydrase XIII, Bim, LASP1, Metallothionein-I, CERT, STAT5, EFHD1, RBP-J kappa (CBF1), FERD3L, RBP1, SMIF, Twinfilin, MCT4, BMAL2, Autophagin-1, NHS, UCK1, KLC3, RPS27A, p38gamma (MAPK12), Ccdc109a, Ubiquitin, IMD2, CDC4L, EYA1, PhLP, SNX9, Kinesin light chain, NANP, XPF, Phosphatase regulator (inhibitor), PDXP, ATP1A3, Fatty acid-binding protein, ERK1/2, NIP2, ADSL, ARHGEF10, MYLK1, CRABP2, Metallothionein-IG, PQBP-1, Kizuna (C20orf19), LCKBP1, SPRR4, Guanylate cyclase A (NPR1), NEPH2, CrkL, Ajuba, GRB2, Rab-9B, PTP-2, CNK1, PEX19, HXK4, DDC, RIFK, Rap1GDS1, BBS9, Metallothionein-II, ZNF655, Tropomodulin, COG3, HMBS, UBC, Telokin, PCP2,

								Troponin C, skeletal muscle, CED-6, GBP5, NUP93, Siglec-E, Dysbindin, Notch, ACSBG1, RAD23A, UBB, BMF, PAP41, IRX4, Guanylate cyclase, IGHG1, ARID4A, NOTCH1 precursor, GCK(MAP4K2), 4E-BP1, RRAD, Sprr2f, DBC1, GINS2, RAB24, IL1RN, CTP synthase II, RPA4, BMP1, DYNLL, DSS1, Occludin, WWOX, E-FABP, DKK3, ACP33, PYDC1 (POP1), Adult hemoglobin, MLX, CAMK2N1, Rich1, Melusin, SAP, APOBEC-1, RIP, SPRR3, MLCK, PAF1, Galectin-13, DOK3
483	diencephalon development	180	6.895E-03	4.555E-02	6.895E-03	4.555E-02	6	DLC1 (Dynein LC8a), RAD1, RBP-J kappa (CBF1), Ubiquitin, UBB, DYNLL
484	ERBB signaling pathway	237	6.908E-03	4.555E-02	6.908E-03	4.555E-02	7	ERK1 (MAPK3), RPS27A, Ubiquitin, ERK1/2, GRB2, UBC, UBB
485	nucleobase metabolic process	81	6.945E-03	4.555E-02	6.945E-03	4.555E-02	4	UCK1, IMD2, ERK1/2, ADSL
486	signal transduction involved in cell cycle checkpoint	81	6.945E-03	4.555E-02	6.945E-03	4.555E-02	4	RPS27A, Ubiquitin, UBC, UBB
48	pyrimidine ribonucleoside metabolic process	42	6.	4.	6.	4.	3	UCK1, CTP synthase II, APOBEC-1

7			991E-03	555E-02	991E-03	555E-02		
488	response to epidermal growth factor	42	6.991E-03	4.555E-02	6.991E-03	4.555E-02	3	ERK1 (MAPK3), STAT5, ERK1/2
489	auditory receptor cell differentiation	42	6.991E-03	4.555E-02	6.991E-03	4.555E-02	3	RBP-J kappa (CBF1), Notch, NOTCH1 precursor
490	cellular response to osmotic stress	42	6.991E-03	4.555E-02	6.991E-03	4.555E-02	3	MYLK1, Telokin, MLCK
491	regulation of cardiac muscle cell proliferation	42	6.991E-03	4.555E-02	6.991E-03	4.555E-02	3	RBP-J kappa (CBF1), Notch, NOTCH1 precursor
492	organic substance metabolic process	1047	7.01	4.55	7.01	4.55	115	MST3, SAE1, PEX2, MUC15, LAGY, MIBP, DLC1 (Dynein LC8a), LCORL, GDP-mannose 4,6 dehydratase, TIPIN, SCMH1, RNASE6, Calpain 1(mu), STAT5A, SPRR1A, RAD1, p38 MAPK, NASP, ERK1 (MAPK3), Bim, CERT, STAT5, RBP-

		6	2E-03	9E-02	2E-03	9E-02		J kappa (CBF1), FERD3L, RBP1, SMIF, Twinfilin, BMAL2, Autophagin-1, Tex1, UCK1, RPS27A, UIP5, p38gamma (MAPK12), ZNF397, Ubiquitin, IMD2, EYA1, PhLP, NANP, DUPD1, XPF, Phosphatase regulator (inhibitor), PDXP, LYPLAL1, ATP1A3, Fatty acid-binding protein, ERK1/2, ADSL, MYLK1, CRABP2, PQBP-1, SPRR4, Guanylate cyclase A (NPR1), MOCS2(small), GA17, Ajuba, GRB2, PTP-2, PEX19, HXK4, Grancalcin, DDC, RIFK, ZNF655, Sen15, COG3, HMBS, RRP42, UBC, Telokin, ST13 (Hip), C16orf75, GBP5, NUP93, HKR1, Notch, ACSBG1, RAD23A, UBB, PAP41, Guanylate cyclase, IGHG1, ARID4A, Cyclophilin E, NOTCH1 precursor, GCK(MAP4K2), 4E-BP1, RRAD, DHRS10, SCML1, DBC1, GINS2, USP5, AO7, IL1RN, CTP synthase II, RPA4, ZNF397OS, BMP1, DYNLL, DSS1, Occludin, WWOX, E-FABP, Adult hemoglobin, GOCAP1, PAIP1, MLX, APOBEC-1, RIP, SPRR3, MLCK, PAF1, Galectin-13
493	transforming growth factor beta receptor signaling pathway	181	7.078E-03	4.593E-02	7.078E-03	4.593E-02	6	SMIF, RPS27A, Ubiquitin, Phosphatase regulator (inhibitor), UBC, UBB
494	positive regulation of myeloid leukocyte differentiation	82	7.249E-03	4.630E-02	7.249E-03	4.630E-02	4	STAT5A, STAT5, LCKBP1, Notch
495	heart valve formation	14	7.294E-03	4.630E-02	7.294E-03	4.630E-02	2	Notch, NOTCH1 precursor
496	vascular smooth muscle cell development	14	7.29	4.63	7.29	4.63	2	Notch, NOTCH1 precursor

			4E-03	0E-02	4E-03	0E-02		
497	3'-UTR-mediated mRNA stabilization	14	7.294E-03	4.630E-02	7.294E-03	4.630E-02	2	TIRAP (Mal), p38 MAPK
498	glomerulus morphogenesis	14	7.294E-03	4.630E-02	7.294E-03	4.630E-02	2	NEPH2, Notch
499	cardiac ventricle formation	14	7.294E-03	4.630E-02	7.294E-03	4.630E-02	2	Notch, NOTCH1 precursor
500	terpenoid biosynthetic process	14	7.294E-03	4.630E-02	7.294E-03	4.630E-02	2	RBP1, CRABP2

**Table 9S: Enrichment analysis report.** Enrichment by GO Processes (healthy control vs Grade IV)								
Enrichment by GO Processes					GRADE IV_INPUT_genelist			
**#**	**Processes**	**Total**	**pValue**	**Min FDR**	**p-value**	**FDR**	**In Data**	**Network Objects from Active Data**
1	positive regulation of histone phosphorylation	8	7.346E-09	1.355E-05	7.346E-09	1.355E-05	4	Cyclin B1, ERK1 (MAPK3), Cyclin B, ERK1/2
2	negative regulation of inclusion body assembly	10	2.193E-08	2.023E-05	2.193E-08	2.023E-05	4	HSPA1B, HSP70, HSPA1A, HSPA2
3	regulation of histone phosphorylation	11	3.438E-08	2.114E-05	3.438E-08	2.114E-05	4	Cyclin B1, ERK1 (MAPK3), Cyclin B, ERK1/2
4	protein refolding	22	7.416E-07	3.421E-04	7.416E-07	3.421E-04	4	HSPA1B, HSP70, HSPA1A, HSPA2

5	regulation of protein serine/threonine kinase activity	553	1.711E-06	5.737E-04	1.711E-06	5.737E-04	11	Ajuba, Cyclin B1, HEXIM1, TIRAP (Mal), Calmyrin, HSP70, ERK1 (MAPK3), HSPA2, CKS2, Cyclin B, ERK1/2
6	regulation of protein phosphorylation	1267	2.476E-06	5.737E-04	2.476E-06	5.737E-04	16	Ajuba, Cyclin B1, HEXIM1, TIRAP (Mal), Calmyrin, HSP70, ERK1 (MAPK3), eEF2K, CD43, HSPA2, CKS2, Spinophilin, Cyclin B, Adult hemoglobin, CAMK2N1, ERK1/2
7	regulation of cyclin-dependent protein serine/threonine kinase activity	119	2.585E-06	5.737E-04	2.585E-06	5.737E-04	6	Cyclin B1, HEXIM1, HSP70, HSPA2, CKS2, Cyclin B
8	heat acclimation	9	2.798E-06	5.737E-04	2.798E-06	5.737E-04	3	HSPA1B, HSP70, HSPA1A
9	cellular heat acclimation	9	2.798E-06	5.737E-04	2.798E-06	5.737E-04	3	HSPA1B, HSP70, HSPA1A
10	regulation of inclusion body assembly	31	3.	5.	3.	5.	4	HSPA1B, HSP70, HSPA1A, HSPA2

			120E-06	756E-04	120E-06	756E-04		
11	response to ischemia	3	4.037E-06	6.771E-04	4.037E-06	6.771E-04	4	HSPA1B, Calmyrin, HSP70, HSPA1A
12	regulation of apolipoprotein binding	2	1.061E-05	1.223E-03	1.061E-05	1.223E-03	2	ERK1 (MAPK3), ERK1/2
13	response to DDT	2	1.061E-05	1.223E-03	1.061E-05	1.223E-03	2	Cyclin B1, Cyclin B
14	regulation of vesicle size	2	1.061E-05	1.223E-03	1.061E-05	1.223E-03	2	Rab-3B, Rab-3
15	synaptonemal complex disassembly	2	1.06	1.22	1.06	1.22	2	HSP70, HSPA2

			1E-05	3E-03	1E-05	3E-03		
16	negative regulation of apolipoprotein binding	2	1.061E-05	1.223E-03	1.061E-05	1.223E-03	2	ERK1 (MAPK3), ERK1/2
17	negative regulation of vasoconstriction	15	1.495E-05	1.493E-03	1.495E-05	1.493E-03	3	HSPA1B, HSP70, HSPA1A
18	negative regulation of extrinsic apoptotic signaling pathway in absence of ligand	46	1.559E-05	1.493E-03	1.559E-05	1.493E-03	4	HSPA1B, HSP70, HSPA1A, EYA1
19	negative regulation of signal transduction in absence of ligand	46	1.559E-05	1.493E-03	1.559E-05	1.493E-03	4	HSPA1B, HSP70, HSPA1A, EYA1
20	macromolecular complex subunit organization	1823	1.619E	1.493E	1.619E	1.493E	18	Cyclin B1, Clp1, ASAP, AL1A1, HBG, HBGA, Calmyrin, COLEC12, HSP70, ERK1 (MAPK3), Annexin VI, CKS2, Spinophilin, Cyclin B, IMD2, Adult hemoglobin, ERK1/2, PQBP-1

			-05	-03	-05	-03		
21	interleukin-1-mediated signaling pathway	17	2.223E-05	1.953E-03	2.223E-05	1.953E-03	3	TIRAP (Mal), ERK1 (MAPK3), ERK1/2
22	regulation of phosphorylation	1539	2.875E-05	2.411E-03	2.875E-05	2.411E-03	16	Ajuba, Cyclin B1, HEXIM1, TIRAP (Mal), Calmyrin, HSP70, ERK1 (MAPK3), eEF2K, CD43, HSPA2, CKS2, Spinophilin, Cyclin B, Adult hemoglobin, CAMK2N1, ERK1/2
23	regulation of protein kinase activity	896	3.091E-05	2.441E-03	3.091E-05	2.441E-03	12	Ajuba, Cyclin B1, HEXIM1, TIRAP (Mal), Calmyrin, HSP70, ERK1 (MAPK3), HSPA2, CKS2, Cyclin B, CAMK2N1, ERK1/2
24	mitotic spindle stabilization	3	3.176E-05	2.441E-03	3.176E-05	2.441E-03	2	Cyclin B1, Cyclin B
25	regulation of erythrocyte differentiation	57	3.673E-0	2.626E-0	3.673E-0	2.626E-0	4	HSPA1B, HSP70, HSPA1A, Adult hemoglobin

			5	3	5	3		
26	oxygen transport	20	3.701E-05	2.626E-03	3.701E-05	2.626E-03	3	HBG, HBGA, Adult hemoglobin
27	cell cycle	1580	3.961E-05	2.691E-03	3.961E-05	2.691E-03	16	Ajuba, Cyclin B1, TIPIN, ASAP, Calmyrin, HSP70, ERK1 (MAPK3), HSPA2, CKS2, Annexin XI, Spinophilin, Septin 3, Cyclin B, EYA1, ERK1/2, PRCC
28	regulation of protein modification process	1584	4.084E-05	2.691E-03	4.084E-05	2.691E-03	16	Ajuba, Cyclin B1, HEXIM1, TIRAP (Mal), Calmyrin, HSP70, ERK1 (MAPK3), eEF2K, CD43, HSPA2, CKS2, Spinophilin, Cyclin B, Adult hemoglobin, CAMK2N1, ERK1/2
29	macromolecular complex assembly	1415	4.310E-05	2.742E-03	4.310E-05	2.742E-03	15	Cyclin B1, Clp1, ASAP, AL1A1, HBG, HBGA, COLEC12, HSP70, ERK1 (MAPK3), Annexin VI, Cyclin B, IMD2, Adult hemoglobin, ERK1/2, PQBP-1
30	protein complex subunit organization	1596	4.474E-05	2.752E-03	4.474E-05	2.752E-03	16	Cyclin B1, ASAP, AL1A1, HBG, HBGA, Calmyrin, COLEC12, HSP70, ERK1 (MAPK3), Annexin VI, CKS2, Spinophilin, Cyclin B, IMD2, Adult hemoglobin, ERK1/2

31	negative regulation of myeloid cell apoptotic process	23	5.709E-05	3.377E-03	5.709E-05	3.377E-03	3	HSPA1B, HSP70, HSPA1A
32	cellular response to iron(III) ion	4	6.338E-05	3.377E-03	6.338E-05	3.377E-03	2	Cyclin B1, Cyclin B
33	regulation of cyclin-dependent protein serine/threonine kinase activity involved in G2/M transition of mitotic cell cycle	4	6.338E-05	3.377E-03	6.338E-05	3.377E-03	2	HSP70, HSPA2
34	positive regulation of cyclin-dependent protein serine/threonine kinase activity involved in G2/M transition of mitotic cell cycle	4	6.338E-05	3.377E-03	6.338E-05	3.377E-03	2	HSP70, HSPA2
35	regulation of kinase activity	966	6.407E-05	3.377E-03	6.407E-05	3.377E-03	12	Ajuba, Cyclin B1, HEXIM1, TIRAP (Mal), Calmyrin, HSP70, ERK1 (MAPK3), HSPA2, CKS2, Cyclin B, CAMK2N1, ERK1/2
36	gas transport	25	7.	3.	7.	3.	3	HBG, HBGA, Adult hemoglobin

			379E-05	782E-03	379E-05	782E-03		
37	positive regulation of histone modification	69	7.804E-05	3.892E-03	7.804E-05	3.892E-03	4	Cyclin B1, ERK1 (MAPK3), Cyclin B, ERK1/2
38	regulation of phosphate metabolic process	2257	8.155E-05	3.960E-03	8.155E-05	3.960E-03	19	Ajuba, Cyclin B1, AL1A1, HEXIM1, TIRAP (Mal), Calmyrin, EPS8L1, HSP70, ERK1 (MAPK3), eEF2K, CD43, HSPA2, CKS2, Spinophilin, Cyclin B, Rab-3, Adult hemoglobin, CAMK2N1, ERK1/2
39	regulation of phosphorus metabolic process	2275	9.078E-05	4.295E-03	9.078E-05	4.295E-03	19	Ajuba, Cyclin B1, AL1A1, HEXIM1, TIRAP (Mal), Calmyrin, EPS8L1, HSP70, ERK1 (MAPK3), eEF2K, CD43, HSPA2, CKS2, Spinophilin, Cyclin B, Rab-3, Adult hemoglobin, CAMK2N1, ERK1/2
40	response to mechanical stimulus	325	9.340E-05	4.308E-03	9.340E-05	4.308E-03	7	Cyclin B1, HSPA1B, HSP70, ERK1 (MAPK3), HSPA1A, Cyclin B, ERK1/2
41	spindle stabilization	5	1.05	4.63	1.05	4.63	2	Cyclin B1, Cyclin B

			4E-04	1E-03	4E-04	1E-03		
42	peptidyl-cysteine methylation	5	1.054E-04	4.631E-03	1.054E-04	4.631E-03	2	Rab-3B, Rab-3
43	negative regulation of microtubule depolymerization	29	1.161E-04	4.983E-03	1.161E-04	4.983E-03	3	Cyclin B1, Calmyrin, Cyclin B
44	regulation of extrinsic apoptotic signaling pathway in absence of ligand	77	1.198E-04	5.023E-03	1.198E-04	5.023E-03	4	HSPA1B, HSP70, HSPA1A, EYA1
45	protein complex assembly	1200	1.227E-04	5.032E-03	1.227E-04	5.032E-03	13	Cyclin B1, ASAP, AL1A1, HBG, HBGA, COLEC12, HSP70, ERK1 (MAPK3), Annexin VI, Cyclin B, IMD2, Adult hemoglobin, ERK1/2
46	protein complex biogenesis	1203	1.258E	5.047E	1.258E	5.047E	13	Cyclin B1, ASAP, AL1A1, HBG, HBGA, COLEC12, HSP70, ERK1 (MAPK3), Annexin VI, Cyclin B, IMD2, Adult hemoglobin, ERK1/2

			-04	-03	-04	-03		
47	positive regulation of protein serine/threonine kinase activity	346	1.377E-04	5.089E-03	1.377E-04	5.089E-03	7	Ajuba, TIRAP (Mal), Calmyrin, HSP70, ERK1 (MAPK3), HSPA2, ERK1/2
48	telomere maintenance	80	1.390E-04	5.089E-03	1.390E-04	5.089E-03	4	HSPA1B, HSP70, HSPA1A, OBFC1
49	telomere organization	81	1.458E-04	5.089E-03	1.458E-04	5.089E-03	4	HSPA1B, HSP70, HSPA1A, OBFC1
50	response to stress	4156	1.538E-04	5.089E-03	1.538E-04	5.089E-03	27	IGHM, Ajuba, IGHG4, Cyclin B1, HSPA1B, TIPIN, AL1A1, HBG, HBGA, CDO1, TIRAP (Mal), Olfactory receptor, Calmyrin, COLEC12, IGHG1, HSP70, ERK1 (MAPK3), CALCOCO2, HSPA1A, CD43, HSPA2, Cyclin B, Adult hemoglobin, EYA1, RIG-G, ERK1/2, CapZIP
51	positive regulation of attachment of spindle microtubules to kinetochore	6	1.578E-0	5.089E-0	1.578E-0	5.089E-0	2	Cyclin B1, Cyclin B

			4	3	4	3		
52	MAPK import into nucleus	6	1.578E-04	5.089E-03	1.578E-04	5.089E-03	2	ERK1 (MAPK3), ERK1/2
53	positive regulation of dopamine uptake involved in synaptic transmission	6	1.578E-04	5.089E-03	1.578E-04	5.089E-03	2	Rab-3B, Rab-3
54	positive regulation of neurotransmitter uptake	6	1.578E-04	5.089E-03	1.578E-04	5.089E-03	2	Rab-3B, Rab-3
55	positive regulation of catecholamine uptake involved in synaptic transmission	6	1.578E-04	5.089E-03	1.578E-04	5.089E-03	2	Rab-3B, Rab-3
56	regulation of Golgi inheritance	6	1.578E-04	5.089E-03	1.578E-04	5.089E-03	2	ERK1 (MAPK3), ERK1/2

57	regulation of chromosome condensation	6	1.578E-04	5.089E-03	1.578E-04	5.089E-03	2	Cyclin B1, Cyclin B
58	positive regulation of chromosome organization	83	1.603E-04	5.089E-03	1.603E-04	5.089E-03	4	Cyclin B1, ERK1 (MAPK3), Cyclin B, ERK1/2
59	phagocytosis	248	1.627E-04	5.089E-03	1.627E-04	5.089E-03	6	IGHG4, COLEC12, IGHG1, ERK1 (MAPK3), Annexin XI, ERK1/2
60	regulation of microtubule depolymerization	34	1.880E-04	5.780E-03	1.880E-04	5.780E-03	3	Cyclin B1, Calmyrin, Cyclin B
61	cell division	772	1.997E-04	5.982E-03	1.997E-04	5.982E-03	10	Cyclin B1, TIPIN, ASAP, Calmyrin, HSP70, HSPA2, CKS2, Annexin XI, Septin 3, Cyclin B
62	regulation of primary metabolic process	65	2.	5.	2.	5.	36	Ajuba, Cyclin B1, HSPA1B, TIPIN, AL1A1, HBG, HBGA, SNX1, HEXIM1, TIRAP (Mal), Olfactory receptor,

		40	010E-04	982E-03	010E-04	982E-03		PTD015, Calmyrin, EPS8L1, HSP70, ERK1 (MAPK3), eEF2K, HSPA1A, CD43, HSPA2, AO7, CKS2, OBFC1, ZNF397OS, ICMT, ZNF397, Spinophilin, Cyclin B, Rab-3, Adult hemoglobin, EYA1, CAMK2N1, ZHX3, ERK1/2, DEAF, PQBP-1
63	regulation of transferase activity	1099	2.159E-04	6.147E-03	2.159E-04	6.147E-03	12	Ajuba, Cyclin B1, HEXIM1, TIRAP (Mal), Calmyrin, HSP70, ERK1 (MAPK3), HSPA2, CKS2, Cyclin B, CAMK2N1, ERK1/2
64	response to iron(III) ion	7	2.204E-04	6.147E-03	2.204E-04	6.147E-03	2	Cyclin B1, Cyclin B
65	caveolin-mediated endocytosis	7	2.204E-04	6.147E-03	2.204E-04	6.147E-03	2	ERK1 (MAPK3), ERK1/2
66	positive regulation of chromosome segregation	7	2.204E-04	6.147E-03	2.204E-04	6.147E-03	2	Cyclin B1, Cyclin B
67	positive regulation of erythrocyte differentiation	36	2.23	6.14	2.23	6.14	3	HSPA1B, HSP70, HSPA1A

			2E-04	7E-03	2E-04	7E-03		
68	cellular component assembly	2054	2.630E-04	7.136E-03	2.630E-04	7.136E-03	17	Ajuba, Cyclin B1, Clp1, ASAP, AL1A1, HBG, HBGA, COLEC12, HSP70, ERK1 (MAPK3), Annexin VI, Spinophilin, Cyclin B, IMD2, Adult hemoglobin, ERK1/2, PQBP-1
69	negative regulation of microtubule polymerization or depolymerization	39	2.837E-04	7.587E-03	2.837E-04	7.587E-03	3	Cyclin B1, Calmyrin, Cyclin B
70	regulation of early endosome to late endosome transport	8	2.933E-04	7.730E-03	2.933E-04	7.730E-03	2	ERK1 (MAPK3), ERK1/2
71	regulation of cell proliferation	1882	3.081E-04	8.007E-03	3.081E-04	8.007E-03	16	Cyclin B1, HSPA1B, TIPIN, TIRAP (Mal), Calmyrin, HSP70, SHCBP1, HSPA1A, CD43, ICMT, Spinophilin, Cyclin B, EYA1, RIG-G, ERK1/2, DEAF
72	regulation of T cell mediated immunity	99	3.156E	8.088E	3.156E	8.088E	4	HSPA1B, HSP70, HSPA1A, CD43

			-04	-03	-04	-03		
73	positive regulation of immune response	670	3.281E-04	8.213E-03	3.281E-04	8.213E-03	9	IGHG4, HSPA1B, TIRAP (Mal), COLEC12, IGHG1, HSP70, ERK1 (MAPK3), HSPA1A, ERK1/2
74	regulation of myeloid cell apoptotic process	41	3.294E-04	8.213E-03	3.294E-04	8.213E-03	3	HSPA1B, HSP70, HSPA1A
75	cellular response to interleukin-1	101	3.407E-04	8.381E-03	3.407E-04	8.381E-03	4	TIRAP (Mal), HSP70, ERK1 (MAPK3), ERK1/2
76	regulation of macromolecule metabolic process	6163	3.513E-04	8.411E-03	3.513E-04	8.411E-03	34	Ajuba, Cyclin B1, HSPA1B, TIPIN, Clp1, HBG, HBGA, SNX1, HEXIM1, TIRAP (Mal), Olfactory receptor, PTD015, Calmyrin, HSP70, ERK1 (MAPK3), eEF2K, HSPA1A, CD43, HSPA2, AO7, CKS2, OBFC1, ZNF397OS, ICMT, ZNF397, Spinophilin, Cyclin B, Adult hemoglobin, EYA1, CAMK2N1, ZHX3, ERK1/2, DEAF, PQBP-1
77	regulation of protein metabolic process	2522	3.520E-0	8.411E-0	3.520E-0	8.411E-0	19	Ajuba, Cyclin B1, HSPA1B, SNX1, HEXIM1, TIRAP (Mal), Calmyrin, HSP70, ERK1 (MAPK3), eEF2K, HSPA1A, CD43, HSPA2, CKS2, Spinophilin, Cyclin B, Adult hemoglobin, CAMK2N1, ERK1/2

			4	3	4	3		
78	regulation of cellular component organization	2107	3.556E-04	8.411E-03	3.556E-04	8.411E-03	17	Ajuba, ZNF261, Cyclin B1, HSPA1B, ASAP, Olfactory receptor, Calmyrin, EPS8L1, HSP70, ERK1 (MAPK3), HSPA1A, HSPA2, Spinophilin, Cyclin B, Rab-3, ERK1/2, PQBP-1
79	peptidyl-tyrosine autophosphorylation	9	3.763E-04	8.788E-03	3.763E-04	8.788E-03	2	ERK1 (MAPK3), ERK1/2
80	regulation of metabolic process	7302	3.915E-04	9.028E-03	3.915E-04	9.028E-03	38	Ajuba, Cyclin B1, HSPA1B, TIPIN, Clp1, AL1A1, HBG, HBGA, SNX1, HEXIM1, TIRAP (Mal), Olfactory receptor, PTD015, Calmyrin, PAP41, EPS8L1, HSP70, ERK1 (MAPK3), eEF2K, HSPA1A, CD43, HSPA2, AO7, CKS2, OBFC1, ZNF397OS, ICMT, ZNF397, Spinophilin, Cyclin B, Rab-3, Adult hemoglobin, EYA1, CAMK2N1, ZHX3, ERK1/2, DEAF, PQBP-1
81	germ cell development	294	4.054E-04	9.235E-03	4.054E-04	9.235E-03	6	Cyclin B1, Calmyrin, HSP70, HSPA2, Cyclin B, DEAF
82	regulation of catalytic activity	2567	4.410E-04	9.923E-03	4.410E-04	9.923E-03	19	Ajuba, Cyclin B1, HSPA1B, AL1A1, HEXIM1, TIRAP (Mal), Calmyrin, PAP41, EPS8L1, HSP70, ERK1 (MAPK3), HSPA1A, HSPA2, CKS2, Spinophilin, Cyclin B, Rab-3, CAMK2N1, ERK1/2

83	wound healing	857	4.583E-04	1.019E-02	4.583E-04	1.019E-02	10	Ajuba, Cyclin B1, HBG, HBGA, HSP70, ERK1 (MAPK3), CD43, Cyclin B, Adult hemoglobin, ERK1/2
84	cellular process involved in reproduction	703	4.655E-04	1.019E-02	4.655E-04	1.019E-02	9	Cyclin B1, HSPA1B, Calmyrin, HSP70, HSPA1A, HSPA2, CKS2, Cyclin B, DEAF
85	regulation of mitotic spindle organization	10	4.693E-04	1.019E-02	4.693E-04	1.019E-02	2	Cyclin B1, Cyclin B
86	G2/M transition of mitotic cell cycle	200	5.070E-04	1.065E-02	5.070E-04	1.065E-02	5	Ajuba, Cyclin B1, HSP70, HSPA2, Cyclin B
87	cell cycle G2/M phase transition	200	5.070E-04	1.065E-02	5.070E-04	1.065E-02	5	Ajuba, Cyclin B1, HSP70, HSPA2, Cyclin B
88	regulation of cellular protein metabolic process	19	5.	1.	5.	1.	16	Ajuba, Cyclin B1, HEXIM1, TIRAP (Mal), Calmyrin, HSP70, ERK1 (MAPK3), eEF2K, CD43, HSPA2, CKS2,

		68	081E-04	065E-02	081E-04	065E-02		Spinophilin, Cyclin B, Adult hemoglobin, CAMK2N1, ERK1/2
89	binding of sperm to zona pellucida	48	5.258E-04	1.090E-02	5.258E-04	1.090E-02	3	HSPA1B, HSP70, HSPA1A
90	positive regulation of protein phosphorylation	875	5.391E-04	1.105E-02	5.391E-04	1.105E-02	10	Ajuba, Cyclin B1, TIRAP (Mal), Calmyrin, HSP70, ERK1 (MAPK3), CD43, HSPA2, Cyclin B, ERK1/2
91	cellular response to heat	49	5.588E-04	1.133E-02	5.588E-04	1.133E-02	3	HSPA1B, HSP70, HSPA1A
92	cellular response to iron ion	11	5.724E-04	1.148E-02	5.724E-04	1.148E-02	2	Cyclin B1, Cyclin B
93	phosphate-containing compound metabolic process	2859	6.23	1.23	6.23	1.23	20	FLJ22494, Ajuba, Cyclin B1, HSPA1B, Clp1, TIRAP (Mal), PAP41, HSP70, ERK1 (MAPK3), eEF2K, COASY, HSPA1A, CKS2, Cyclin B, IMD2, Rab-3, EYA1, DUPD1, Fructosamine-3-kinase, ERK1/2

			2E-04	3E-02	2E-04	3E-02		
94	[cell cycle process]	1237	6.299E-04	1.233E-02	6.299E-04	1.233E-02	12	Ajuba, Cyclin B1, TIPIN, ASAP, HSP70, HSPA2, CKS2, Annexin XI, Spinophilin, Cyclin B, EYA1, PRCC
95	[regulation of cell cycle]	1062	6.351E-04	1.233E-02	6.351E-04	1.233E-02	11	Cyclin B1, TIPIN, ASAP, HEXIM1, Olfactory receptor, HSP70, HSPA2, CKS2, Spinophilin, Cyclin B, PRCC
96	[regulation of dopamine uptake involved in synaptic transmission]	12	6.855E-04	1.290E-02	6.855E-04	1.290E-02	2	Rab-3B, Rab-3
97	[regulation of catecholamine uptake involved in synaptic transmission]	12	6.855E-04	1.290E-02	6.855E-04	1.290E-02	2	Rab-3B, Rab-3
98	[DNA damage induced protein phosphorylation]	12	6.855E	1.290E	6.855E	1.290E	2	ERK1 (MAPK3), ERK1/2

			-04	-02	-04	-02		
99	cellular component biogenesis	2239	7.191E-04	1.340E-02	7.191E-04	1.340E-02	17	Ajuba, Cyclin B1, Clp1, ASAP, AL1A1, HBG, HBGA, COLEC12, HSP70, ERK1 (MAPK3), Annexin VI, Spinophilin, Cyclin B, IMD2, Adult hemoglobin, ERK1/2, PQBP-1
100	defense response	1833	7.375E-04	1.361E-02	7.375E-04	1.361E-02	15	IGHM, IGHG4, HSPA1B, CDO1, TIRAP (Mal), Olfactory receptor, COLEC12, IGHG1, HSP70, ERK1 (MAPK3), CALCOCO2, HSPA1A, CD43, RIG-G, ERK1/2
101	positive regulation of transferase activity	754	7.688E-04	1.404E-02	7.688E-04	1.404E-02	9	Ajuba, Cyclin B1, TIRAP (Mal), Calmyrin, HSP70, ERK1 (MAPK3), HSPA2, Cyclin B, ERK1/2
102	regulation of attachment of spindle microtubules to kinetochore	13	8.084E-04	1.462E-02	8.084E-04	1.462E-02	2	Cyclin B1, Cyclin B
103	phosphorus metabolic process	2922	8.255E-0	1.479E-0	8.255E-0	1.479E-0	20	FLJ22494, Ajuba, Cyclin B1, HSPA1B, Clp1, TIRAP (Mal), PAP41, HSP70, ERK1 (MAPK3), eEF2K, COASY, HSPA1A, CKS2, Cyclin B, IMD2, Rab-3, EYA1, DUPD1, Fructosamine-3-kinase, ERK1/2

			4	2	4	2		
104	endocytosis	610	8.409E-04	1.488E-02	8.409E-04	1.488E-02	8	IGHG4, SNX1, COLEC12, IGHG1, ERK1 (MAPK3), Annexin XI, Rab-3, ERK1/2
105	positive regulation of myeloid cell differentiation	129	8.576E-04	1.488E-02	8.576E-04	1.488E-02	4	HSPA1B, HSP70, HSPA1A, Adult hemoglobin
106	regulation of histone modification	129	8.576E-04	1.488E-02	8.576E-04	1.488E-02	4	Cyclin B1, ERK1 (MAPK3), Cyclin B, ERK1/2
107	mitotic cell cycle	930	8.628E-04	1.488E-02	8.628E-04	1.488E-02	10	Ajuba, Cyclin B1, TIPIN, ASAP, Calmyrin, HSP70, HSPA2, Cyclin B, EYA1, PRCC
108	negative regulation of extrinsic apoptotic signaling pathway	131	9.082E-04	1.552E-02	9.082E-04	1.552E-02	4	HSPA1B, HSP70, HSPA1A, EYA1

109	regulation of spindle organization	14	9.411E-04	1.593E-02	9.411E-04	1.593E-02	2	Cyclin B1, Cyclin B
110	immune response-regulating cell surface receptor signaling pathway involved in phagocytosis	134	9.880E-04	1.642E-02	9.880E-04	1.642E-02	4	IGHG4, IGHG1, ERK1 (MAPK3), ERK1/2
111	Fc-gamma receptor signaling pathway involved in phagocytosis	134	9.880E-04	1.642E-02	9.880E-04	1.642E-02	4	IGHG4, IGHG1, ERK1 (MAPK3), ERK1/2
112	cellular component organization	5419	1.001E-03	1.644E-02	1.001E-03	1.644E-02	30	Ajuba, ZNF261, Cyclin B1, HSPA1B, TIPIN, Clp1, ASAP, AL1A1, HBG, HBGA, Olfactory receptor, Rab-3B, Calmyrin, COLEC12, HSP70, ERK1 (MAPK3), Annexin VI, HSPA1A, HSPA2, CKS2, OBFC1, ICMT, Spinophilin, Cyclin B, IMD2, Rab-3, Adult hemoglobin, EYA1, ERK1/2, PQBP-1
113	positive regulation of T cell mediated cytotoxicity	60	1.011E-03	1.644E-02	1.011E-03	1.644E-02	3	HSPA1B, HSP70, HSPA1A
11	Fc-gamma receptor signaling pathway	13	1.	1.	1.	1.	4	IGHG4, IGHG1, ERK1 (MAPK3), ERK1/2

4		5	016E-03	644E-02	016E-03	644E-02		
115	Fc receptor mediated stimulatory signaling pathway	136	1.044E-03	1.675E-02	1.044E-03	1.675E-02	4	IGHG4, IGHG1, ERK1 (MAPK3), ERK1/2
116	sperm-egg recognition	61	1.061E-03	1.686E-02	1.061E-03	1.686E-02	3	HSPA1B, HSP70, HSPA1A
117	negative regulation of protein phosphorylation	354	1.069E-03	1.686E-02	1.069E-03	1.686E-02	6	Cyclin B1, HEXIM1, Calmyrin, HSP70, Cyclin B, CAMK2N1
118	regulation of chromatin organization	140	1.162E-03	1.817E-02	1.162E-03	1.817E-02	4	Cyclin B1, ERK1 (MAPK3), Cyclin B, ERK1/2
119	positive regulation of immune system process	969	1.17	1.82	1.17	1.82	10	IGHG4, HSPA1B, TIRAP (Mal), COLEC12, IGHG1, HSP70, ERK1 (MAPK3), HSPA1A, CD43, ERK1/2

			8E-03	7E-02	8E-03	7E-02		
120	response to interleukin-1	142	1.225E-03	1.883E-02	1.225E-03	1.883E-02	4	TIRAP (Mal), HSP70, ERK1 (MAPK3), ERK1/2
121	regulation of microtubule polymerization or depolymerization	65	1.276E-03	1.945E-02	1.276E-03	1.945E-02	3	Cyclin B1, Calmyrin, Cyclin B
122	cellular process involved in reproduction in multicellular organism	367	1.286E-03	1.945E-02	1.286E-03	1.945E-02	6	Cyclin B1, Calmyrin, HSP70, HSPA2, Cyclin B, DEAF
123	positive regulation of biological process	5249	1.343E-03	2.014E-02	1.343E-03	2.014E-02	29	Ajuba, IGHG4, Cyclin B1, HSPA1B, TIPIN, AL1A1, SNX1, TIRAP (Mal), Olfactory receptor, PTD015, Rab-3B, Calmyrin, EPS8L1, COLEC12, IGHG1, HSP70, ERK1 (MAPK3), HSPA1A, CD43, HSPA2, OBFC1, ICMT, Cyclin B, Rab-3, Adult hemoglobin, EYA1, ZHX3, ERK1/2, DEAF
124	regulation of neurotransmitter uptake	17	1.398E	2.079E	1.398E	2.079E	2	Rab-3B, Rab-3

			-03	-02	-03	-02		
125	single fertilization	148	1.427E-03	2.106E-02	1.427E-03	2.106E-02	4	HSPA1B, Olfactory receptor, HSP70, HSPA1A
126	cell recognition	149	1.463E-03	2.142E-02	1.463E-03	2.142E-02	4	HSPA1B, COLEC12, HSP70, HSPA1A
127	cellular component organization or biogenesis	5557	1.547E-03	2.196E-02	1.547E-03	2.196E-02	30	Ajuba, ZNF261, Cyclin B1, HSPA1B, TIPIN, Clp1, ASAP, AL1A1, HBG, HBGA, Olfactory receptor, Rab-3B, Calmyrin, COLEC12, HSP70, ERK1 (MAPK3), Annexin VI, HSPA1A, HSPA2, CKS2, OBFC1, ICMT, Spinophilin, Cyclin B, IMD2, Rab-3, Adult hemoglobin, EYA1, ERK1/2, PQBP-1
128	regulation of myeloid cell differentiation	257	1.556E-03	2.196E-02	1.556E-03	2.196E-02	5	HSPA1B, Calmyrin, HSP70, HSPA1A, Adult hemoglobin
129	ventricular cardiac muscle cell development	18	1.569E-0	2.196E-0	1.569E-0	2.196E-0	2	Cyclin B1, Cyclin B

			3	2	3	2		
130	mitotic metaphase plate congression	18	1.569E-03	2.196E-02	1.569E-03	2.196E-02	2	Cyclin B1, Cyclin B
131	peptidyl-cysteine modification	18	1.569E-03	2.196E-02	1.569E-03	2.196E-02	2	Rab-3B, Rab-3
132	regulation of T cell mediated cytotoxicity	70	1.581E-03	2.196E-02	1.581E-03	2.196E-02	3	HSPA1B, HSP70, HSPA1A
133	response to ethanol	258	1.583E-03	2.196E-02	1.583E-03	2.196E-02	5	HSPA1B, AL1A1, CDO1, HSP70, HSPA1A
134	negative regulation of protein depolymerization	71	1.646E-03	2.261E-02	1.646E-03	2.261E-02	3	Cyclin B1, Calmyrin, Cyclin B

135	positive regulation of phosphorylation	1014	1.654E-03	2.261E-02	1.654E-03	2.261E-02	10	Ajuba, Cyclin B1, TIRAP (Mal), Calmyrin, HSP70, ERK1 (MAPK3), CD43, HSPA2, Cyclin B, ERK1/2
136	positive regulation of ERK1 and ERK2 cascade	156	1.731E-03	2.348E-02	1.731E-03	2.348E-02	4	TIRAP (Mal), Calmyrin, ERK1 (MAPK3), ERK1/2
137	mitotic cell cycle process	850	1.772E-03	2.384E-02	1.772E-03	2.384E-02	9	Ajuba, Cyclin B1, TIPIN, ASAP, HSP70, HSPA2, Cyclin B, EYA1, PRCC
138	cell-cell recognition	73	1.783E-03	2.384E-02	1.783E-03	2.384E-02	3	HSPA1B, HSP70, HSPA1A
139	negative regulation of protein complex disassembly	74	1.854E-03	2.461E-02	1.854E-03	2.461E-02	3	Cyclin B1, Calmyrin, Cyclin B
14	regulation of cellular metabolic process	64	1.	2.	1.	2.	33	Ajuba, Cyclin B1, TIPIN, AL1A1, HBG, HBGA, HEXIM1, TIRAP (Mal), Olfactory receptor, PTD015, Calmyrin,

0		46	953E-03	567E-02	953E-03	567E-02		EPS8L1, HSP70, ERK1 (MAPK3), eEF2K, CD43, HSPA2, AO7, CKS2, OBFC1, ZNF397OS, ICMT, ZNF397, Spinophilin, Cyclin B, Rab-3, Adult hemoglobin, EYA1, CAMK2N1, ZHX3, ERK1/2, DEAF, PQBP-1
141	protein oligomerization	542	1.967E-03	2.567E-02	1.967E-03	2.567E-02	7	AL1A1, HBG, HBGA, COLEC12, Annexin VI, IMD2, Adult hemoglobin
142	regulation of molecular function	3133	1.976E-03	2.567E-02	1.976E-03	2.567E-02	20	Ajuba, Cyclin B1, HSPA1B, AL1A1, HEXIM1, TIRAP (Mal), Calmyrin, PAP41, EPS8L1, HSP70, ERK1 (MAPK3), HSPA1A, HSPA2, AO7, CKS2, Spinophilin, Cyclin B, Rab-3, CAMK2N1, ERK1/2
143	positive regulation of T cell mediated immunity	77	2.078E-03	2.681E-02	2.078E-03	2.681E-02	3	HSPA1B, HSP70, HSPA1A
144	positive regulation of mRNA 3'-end processing	21	2.140E-03	2.742E-02	2.140E-03	2.742E-02	2	Cyclin B1, Cyclin B
145	response to toxic substance	277	2.15	2.74	2.15	2.74	5	Cyclin B1, HSP70, ERK1 (MAPK3), Cyclin B, ERK1/2

			9E-03	7E-02	9E-03	7E-02		
146	nuclear division	553	2.203E-03	2.783E-02	2.203E-03	2.783E-02	7	Cyclin B1, TIPIN, ASAP, HSP70, HSPA2, CKS2, Cyclin B
147	regulation of immune response	1058	2.261E-03	2.838E-02	2.261E-03	2.838E-02	10	IGHG4, HSPA1B, TIRAP (Mal), COLEC12, IGHG1, HSP70, ERK1 (MAPK3), HSPA1A, CD43, ERK1/2
148	response to unfolded protein	170	2.366E-03	2.950E-02	2.366E-03	2.950E-02	4	HSPA1B, HSP70, HSPA1A, HSPA2
149	cell cycle phase transition	416	2.420E-03	2.996E-02	2.420E-03	2.996E-02	6	Ajuba, Cyclin B1, TIPIN, HSP70, HSPA2, Cyclin B
150	response to wounding	1450	2.464E	3.028E	2.464E	3.028E	12	Ajuba, Cyclin B1, HBG, HBGA, CDO1, TIRAP (Mal), HSP70, ERK1 (MAPK3), CD43, Cyclin B, Adult hemoglobin, ERK1/2

			-03	-02	-03	-02		
151	negative regulation of phosphorylation	418	2.478E-03	3.028E-02	2.478E-03	3.028E-02	6	Cyclin B1, HEXIM1, Calmyrin, HSP70, Cyclin B, CAMK2N1
152	negative regulation of growth	289	2.594E-03	3.148E-02	2.594E-03	3.148E-02	5	HSPA1B, TIRAP (Mal), HSP70, HSPA1A, Spinophilin
153	regulation of organelle organization	901	2.630E-03	3.172E-02	2.630E-03	3.172E-02	9	Cyclin B1, ASAP, Olfactory receptor, Calmyrin, ERK1 (MAPK3), Spinophilin, Cyclin B, Rab-3, ERK1/2
154	regulation of protein binding	176	2.682E-03	3.186E-02	2.682E-03	3.186E-02	4	TIRAP (Mal), HSP70, ERK1 (MAPK3), ERK1/2
155	single-organism organelle organization	1878	2.758E-0	3.186E-0	2.758E-0	3.186E-0	14	Cyclin B1, HSPA1B, TIPIN, ASAP, Calmyrin, HSP70, HSPA1A, HSPA2, CKS2, OBFC1, Spinophilin, Cyclin B, Rab-3, EYA1

			3	2	3	2		
156	regulation of mRNA 3'-end processing	24	2.795E-03	3.186E-02	2.795E-03	3.186E-02	2	Cyclin B1, Cyclin B
157	positive regulation of cardiac muscle cell proliferation	24	2.795E-03	3.186E-02	2.795E-03	3.186E-02	2	Cyclin B1, Cyclin B
158	regulation of lymphocyte mediated immunity	179	2.850E-03	3.186E-02	2.850E-03	3.186E-02	4	HSPA1B, HSP70, HSPA1A, CD43
159	regulation of cytoskeleton organization	431	2.884E-03	3.186E-02	2.884E-03	3.186E-02	6	Cyclin B1, Olfactory receptor, Calmyrin, ERK1 (MAPK3), Cyclin B, ERK1/2
160	positive regulation of cell cycle	180	2.908E-03	3.186E-02	2.908E-03	3.186E-02	4	Cyclin B1, HSP70, HSPA2, Cyclin B

161	positive regulation of leukocyte mediated cytotoxicity	87	2.941E-03	3.186E-02	2.941E-03	3.186E-02	3	HSPA1B, HSP70, HSPA1A
162	organelle fission	583	2.957E-03	3.186E-02	2.957E-03	3.186E-02	7	Cyclin B1, TIPIN, ASAP, HSP70, HSPA2, CKS2, Cyclin B
163	cellular response to stress	1685	2.965E-03	3.186E-02	2.965E-03	3.186E-02	13	Cyclin B1, HSPA1B, TIPIN, TIRAP (Mal), Calmyrin, HSP70, ERK1 (MAPK3), HSPA1A, Cyclin B, Adult hemoglobin, EYA1, ERK1/2, CapZIP
164	regulation of chromosome organization	181	2.966E-03	3.186E-02	2.966E-03	3.186E-02	4	Cyclin B1, ERK1 (MAPK3), Cyclin B, ERK1/2
165	regulation of nucleobase-containing compound metabolic process	4986	2.992E-03	3.186E-02	2.992E-03	3.186E-02	27	Ajuba, Cyclin B1, TIPIN, AL1A1, HBG, HBGA, HEXIM1, TIRAP (Mal), Olfactory receptor, PTD015, Calmyrin, EPS8L1, HSP70, ERK1 (MAPK3), AO7, OBFC1, ZNF397OS, ICMT, ZNF397, Cyclin B, Rab-3, Adult hemoglobin, EYA1, ZHX3, ERK1/2, DEAF, PQBP-1
16	pattern recognition receptor signaling pathway	18	3.	3.	3.	3.	4	TIRAP (Mal), COLEC12, ERK1 (MAPK3), ERK1/2

6		2	026E-03	186E-02	026E-03	186E-02		
167	response to insecticide	25	3.031E-03	3.186E-02	3.031E-03	3.186E-02	2	Cyclin B1, Cyclin B
168	metaphase plate congression	25	3.031E-03	3.186E-02	3.031E-03	3.186E-02	2	Cyclin B1, Cyclin B
169	positive regulation of histone acetylation	25	3.031E-03	3.186E-02	3.031E-03	3.186E-02	2	ERK1 (MAPK3), ERK1/2
170	synaptonemal complex organization	25	3.031E-03	3.186E-02	3.031E-03	3.186E-02	2	HSP70, HSPA2
171	regulation of synaptic transmission, dopaminergic	25	3.03	3.18	3.03	3.18	2	Rab-3B, Rab-3

			1E-03	6E-02	1E-03	6E-02		
172	response to topologically incorrect protein	183	3.086E-03	3.186E-02	3.086E-03	3.186E-02	4	HSPA1B, HSP70, HSPA1A, HSPA2
173	regulation of protein depolymerization	89	3.137E-03	3.186E-02	3.137E-03	3.186E-02	3	Cyclin B1, Calmyrin, Cyclin B
174	positive regulation of protein modification process	1109	3.179E-03	3.186E-02	3.179E-03	3.186E-02	10	Ajuba, Cyclin B1, TIRAP (Mal), Calmyrin, HSP70, ERK1 (MAPK3), CD43, HSPA2, Cyclin B, ERK1/2
175	innate immune response-activating signal transduction	186	3.271E-03	3.186E-02	3.271E-03	3.186E-02	4	TIRAP (Mal), COLEC12, ERK1 (MAPK3), ERK1/2
176	male meiosis I	26	3.277E	3.186E	3.277E	3.186E	2	HSP70, HSPA2

			-03	-02	-03	-02		
177	deoxyribonucleoside diphosphate catabolic process	1	3.281E-03	3.186E-02	3.281E-03	3.186E-02	1	FLJ22494
178	GDP catabolic process	1	3.281E-03	3.186E-02	3.281E-03	3.186E-02	1	FLJ22494
179	TIRAP-dependent toll-like receptor signaling pathway	1	3.281E-03	3.186E-02	3.281E-03	3.186E-02	1	TIRAP (Mal)
180	siRNA loading onto RISC involved in RNA interference	1	3.281E-03	3.186E-02	3.281E-03	3.186E-02	1	Clp1
181	dADP catabolic process	1	3.281E-0	3.186E-0	3.281E-0	3.186E-0	1	FLJ22494

			3	2	3	2		
182	regulation of chemokine (C-X-C motif) ligand 1 production	1	3.281E-03	3.186E-02	3.281E-03	3.186E-02	1	TIRAP (Mal)
183	TIRAP-dependent toll-like receptor 4 signaling pathway	1	3.281E-03	3.186E-02	3.281E-03	3.186E-02	1	TIRAP (Mal)
184	regulation of interleukin-15 production	1	3.281E-03	3.186E-02	3.281E-03	3.186E-02	1	TIRAP (Mal)
185	positive regulation of chemokine (C-X-C motif) ligand 1 production	1	3.281E-03	3.186E-02	3.281E-03	3.186E-02	1	TIRAP (Mal)
186	fructoselysine metabolic process	1	3.281E-03	3.186E-02	3.281E-03	3.186E-02	1	Fructosamine-3-kinase

187	positive regulation of interleukin-15 production	1	3.281E-03	3.186E-02	3.281E-03	3.186E-02	1	TIRAP (Mal)
188	purine deoxyribonucleoside diphosphate catabolic process	1	3.281E-03	3.186E-02	3.281E-03	3.186E-02	1	FLJ22494
189	dGDP catabolic process	1	3.281E-03	3.186E-02	3.281E-03	3.186E-02	1	FLJ22494
190	thymocyte aggregation	1	3.281E-03	3.186E-02	3.281E-03	3.186E-02	1	CD43
191	immune response-activating signal transduction	445	3.375E-03	3.260E-02	3.375E-03	3.260E-02	6	IGHG4, TIRAP (Mal), COLEC12, IGHG1, ERK1 (MAPK3), ERK1/2
19	regulation of adaptive immune response based on somatic	19	3.	3.	3.	3.	4	HSPA1B, HSP70, HSPA1A, CD43

2	recombination of immune receptors built from immunoglobulin superfamily domains	0	529E-03	358E-02	529E-03	358E-02		
193	positive regulation of peptidyl-lysine acetylation	27	3.531E-03	3.358E-02	3.531E-03	3.358E-02	2	ERK1 (MAPK3), ERK1/2
194	positive regulation of neurotransmitter transport	27	3.531E-03	3.358E-02	3.531E-03	3.358E-02	2	Rab-3B, Rab-3
195	positive regulation of cell killing	93	3.551E-03	3.360E-02	3.551E-03	3.360E-02	3	HSPA1B, HSP70, HSPA1A
196	positive regulation of protein kinase activity	604	3.594E-03	3.383E-02	3.594E-03	3.383E-02	7	Ajuba, TIRAP (Mal), Calmyrin, HSP70, ERK1 (MAPK3), HSPA2, ERK1/2
197	toll-like receptor 5 signaling pathway	94	3.66	3.42	3.66	3.42	3	TIRAP (Mal), ERK1 (MAPK3), ERK1/2

			0E-03	7E-02	0E-03	7E-02		
198	response to stilbenoid	28	3.795E-03	3.490E-02	3.795E-03	3.490E-02	2	Adult hemoglobin, RIG-G
199	positive regulation of protein acetylation	28	3.795E-03	3.490E-02	3.795E-03	3.490E-02	2	ERK1 (MAPK3), ERK1/2
200	protein import	194	3.801E-03	3.490E-02	3.801E-03	3.490E-02	4	Olfactory receptor, HSP70, ERK1 (MAPK3), ERK1/2
201	positive regulation of phosphorus metabolic process	1138	3.821E-03	3.490E-02	3.821E-03	3.490E-02	10	Ajuba, Cyclin B1, TIRAP (Mal), Calmyrin, HSP70, ERK1 (MAPK3), CD43, HSPA2, Cyclin B, ERK1/2
202	positive regulation of phosphate metabolic process	1138	3.821E	3.490E	3.821E	3.490E	10	Ajuba, Cyclin B1, TIRAP (Mal), Calmyrin, HSP70, ERK1 (MAPK3), CD43, HSPA2, Cyclin B, ERK1/2

			-03	-02	-03	-02		
203	negative regulation of cellular component organization	615	3.967E-03	3.605E-02	3.967E-03	3.605E-02	7	Cyclin B1, HSPA1B, Calmyrin, HSP70, HSPA1A, HSPA2, Cyclin B
204	regulation of cell cycle process	616	4.002E-03	3.613E-02	4.002E-03	3.613E-02	7	Cyclin B1, TIPIN, ASAP, Olfactory receptor, Spinophilin, Cyclin B, PRCC
205	activation of innate immune response	197	4.014E-03	3.613E-02	4.014E-03	3.613E-02	4	TIRAP (Mal), COLEC12, ERK1 (MAPK3), ERK1/2
206	oocyte maturation	29	4.068E-03	3.643E-02	4.068E-03	3.643E-02	2	Cyclin B1, Cyclin B
207	toll-like receptor TLR6:TLR2 signaling pathway	99	4.233E-0	3.701E-0	4.233E-0	3.701E-0	3	TIRAP (Mal), ERK1 (MAPK3), ERK1/2

			3	2	3	2		
208	toll-like receptor TLR1:TLR2 signaling pathway	99	4.233E-03	3.701E-02	4.233E-03	3.701E-02	3	TIRAP (Mal), ERK1 (MAPK3), ERK1/2
209	response to abiotic stimulus	1551	4.268E-03	3.701E-02	4.268E-03	3.701E-02	12	Ajuba, Cyclin B1, HSPA1B, TIPIN, HSP70, ERK1 (MAPK3), HSPA1A, Cyclin B, Rab-3, EYA1, ERK1/2, CapZIP
210	Ras protein signal transduction	201	4.311E-03	3.701E-02	4.311E-03	3.701E-02	4	EPS8L1, ERK1 (MAPK3), Rab-3, ERK1/2
211	regulation of nitrogen compound metabolic process	5112	4.336E-03	3.701E-02	4.336E-03	3.701E-02	27	Ajuba, Cyclin B1, TIPIN, AL1A1, HBG, HBGA, HEXIM1, TIRAP (Mal), Olfactory receptor, PTD015, Calmyrin, EPS8L1, HSP70, ERK1 (MAPK3), AO7, OBFC1, ZNF397OS, ICMT, ZNF397, Cyclin B, Rab-3, Adult hemoglobin, EYA1, ZHX3, ERK1/2, DEAF, PQBP-1
212	positive regulation of cyclin-dependent protein kinase activity	30	4.349E-03	3.701E-02	4.349E-03	3.701E-02	2	HSP70, HSPA2

213	establishment of chromosome localization	30	4.349E-03	3.701E-02	4.349E-03	3.701E-02	2	Cyclin B1, Cyclin B
214	histone phosphorylation	30	4.349E-03	3.701E-02	4.349E-03	3.701E-02	2	Cyclin B1, Cyclin B
215	positive regulation of mRNA processing	30	4.349E-03	3.701E-02	4.349E-03	3.701E-02	2	Cyclin B1, Cyclin B
216	JAK-STAT cascade involved in growth hormone signaling pathway	30	4.349E-03	3.701E-02	4.349E-03	3.701E-02	2	ERK1 (MAPK3), ERK1/2
217	regulation of vasoconstriction	100	4.353E-03	3.701E-02	4.353E-03	3.701E-02	3	HSPA1B, HSP70, HSPA1A
21	toll-like receptor 2 signaling pathway	10	4.	3.	4.	3.	3	TIRAP (Mal), ERK1 (MAPK3), ERK1/2

8		1	476E-03	771E-02	476E-03	771E-02		
219	regulation of protein complex disassembly	101	4.476E-03	3.771E-02	4.476E-03	3.771E-02	3	Cyclin B1, Calmyrin, Cyclin B
220	positive regulation of kinase activity	632	4.600E-03	3.841E-02	4.600E-03	3.841E-02	7	Ajuba, TIRAP (Mal), Calmyrin, HSP70, ERK1 (MAPK3), HSPA2, ERK1/2
221	regulation of growth	802	4.621E-03	3.841E-02	4.621E-03	3.841E-02	8	Cyclin B1, HSPA1B, TIRAP (Mal), Calmyrin, HSP70, HSPA1A, Spinophilin, Cyclin B
222	mitotic cell cycle checkpoint	205	4.622E-03	3.841E-02	4.622E-03	3.841E-02	4	Cyclin B1, TIPIN, Cyclin B, PRCC
223	regulation of ERK1 and ERK2 cascade	206	4.70	3.89	4.70	3.89	4	TIRAP (Mal), Calmyrin, ERK1 (MAPK3), ERK1/2

			2E-03	0E-02	2E-03	0E-02		
224	regulation of adaptive immune response	207	4.783E-03	3.939E-02	4.783E-03	3.939E-02	4	HSPA1B, HSP70, HSPA1A, CD43
225	negative regulation of cysteine-type endopeptidase activity involved in apoptotic process	104	4.857E-03	3.957E-02	4.857E-03	3.957E-02	3	HSPA1B, HSP70, HSPA1A
226	regulation of leukocyte mediated cytotoxicity	104	4.857E-03	3.957E-02	4.857E-03	3.957E-02	3	HSPA1B, HSP70, HSPA1A
227	positive regulation of protein metabolic process	1374	4.868E-03	3.957E-02	4.868E-03	3.957E-02	11	Ajuba, Cyclin B1, SNX1, TIRAP (Mal), Calmyrin, HSP70, ERK1 (MAPK3), CD43, HSPA2, Cyclin B, ERK1/2
228	cellular response to DNA damage stimulus	810	4.902E	3.967E	4.902E	3.967E	8	HSPA1B, TIPIN, Calmyrin, HSP70, ERK1 (MAPK3), HSPA1A, EYA1, ERK1/2

			-03	-02	-03	-02		
229	negative regulation of cell growth	210	5.031E-03	4.053E-02	5.031E-03	4.053E-02	4	HSPA1B, HSP70, HSPA1A, Spinophilin
230	regulation of extrinsic apoptotic signaling pathway	211	5.116E-03	4.092E-02	5.116E-03	4.092E-02	4	HSPA1B, HSP70, HSPA1A, EYA1
231	regulation of cellular response to stress	485	5.124E-03	4.092E-02	5.124E-03	4.092E-02	6	Ajuba, TIRAP (Mal), ERK1 (MAPK3), Adult hemoglobin, EYA1, ERK1/2
232	negative regulation of phosphorus metabolic process	487	5.226E-03	4.136E-02	5.226E-03	4.136E-02	6	Cyclin B1, HEXIM1, Calmyrin, HSP70, Cyclin B, CAMK2N1
233	negative regulation of phosphate metabolic process	487	5.226E-0	4.136E-0	5.226E-0	4.136E-0	6	Cyclin B1, HEXIM1, Calmyrin, HSP70, Cyclin B, CAMK2N1

			3	2	3	2		
234	chromosome localization	33	5.245E-03	4.136E-02	5.245E-03	4.136E-02	2	Cyclin B1, Cyclin B
235	negative regulation of cysteine-type endopeptidase activity	108	5.395E-03	4.236E-02	5.395E-03	4.236E-02	3	HSPA1B, HSP70, HSPA1A
236	negative regulation of biological process	4668	5.427E-03	4.243E-02	5.427E-03	4.243E-02	25	Ajuba, Cyclin B1, HSPA1B, TIPIN, Clp1, HBG, HBGA, HEXIM1, TIRAP (Mal), PTD015, Thioredoxin-like 2, Calmyrin, HSP70, HSPA1A, CD43, HSPA2, Spinophilin, Cyclin B, Adult hemoglobin, EYA1, RIG-G, CAMK2N1, ZHX3, ERK1/2, PRCC
237	response to organic substance	3414	5.456E-03	4.247E-02	5.456E-03	4.247E-02	20	Cyclin B1, HSPA1B, AL1A1, CDO1, TIRAP (Mal), Calmyrin, COLEC12, HSP70, ERK1 (MAPK3), CALCOCO2, eEF2K, SHCBP1, HSPA1A, HSPA2, Spinophilin, Cyclin B, IMD2, Adult hemoglobin, RIG-G, ERK1/2
238	regulation of immune system process	1602	5.521E-03	4.280E-02	5.521E-03	4.280E-02	12	IGHG4, HSPA1B, TIRAP (Mal), Calmyrin, COLEC12, IGHG1, HSP70, ERK1 (MAPK3), HSPA1A, CD43, Adult hemoglobin, ERK1/2

239	regulation of cell killing	110	5.677E-03	4.382E-02	5.677E-03	4.382E-02	3	HSPA1B, HSP70, HSPA1A
240	fertilization	218	5.736E-03	4.409E-02	5.736E-03	4.409E-02	4	HSPA1B, Olfactory receptor, HSP70, HSPA1A
241	regulation of leukocyte mediated immunity	219	5.828E-03	4.433E-02	5.828E-03	4.433E-02	4	HSPA1B, HSP70, HSPA1A, CD43
242	growth hormone receptor signaling pathway	35	5.886E-03	4.433E-02	5.886E-03	4.433E-02	2	ERK1 (MAPK3), ERK1/2
243	sexual reproduction	1019	5.891E-03	4.433E-02	5.891E-03	4.433E-02	9	Cyclin B1, HSPA1B, Olfactory receptor, Calmyrin, HSP70, HSPA1A, HSPA2, Cyclin B, DEAF
24	small GTPase mediated signal transduction	50	5.	4.	5.	4.	6	ARL5B, Rab-3B, EPS8L1, ERK1 (MAPK3), Rab-3, ERK1/2

4		0	927E-03	433E-02	927E-03	433E-02		
245	blood coagulation	663	5.949E-03	4.433E-02	5.949E-03	4.433E-02	7	HBG, HBGA, HSP70, ERK1 (MAPK3), CD43, Adult hemoglobin, ERK1/2
246	coagulation	663	5.949E-03	4.433E-02	5.949E-03	4.433E-02	7	HBG, HBGA, HSP70, ERK1 (MAPK3), CD43, Adult hemoglobin, ERK1/2
247	meiosis I	112	5.967E-03	4.433E-02	5.967E-03	4.433E-02	3	HSP70, HSPA2, CKS2
248	MyD88-dependent toll-like receptor signaling pathway	112	5.967E-03	4.433E-02	5.967E-03	4.433E-02	3	TIRAP (Mal), ERK1 (MAPK3), ERK1/2
249	regulation of mitotic cell cycle	501	5.98	4.43	5.98	4.43	6	Cyclin B1, TIPIN, ASAP, Spinophilin, Cyclin B, PRCC

			3E-03	3E-02	3E-03	3E-02		
250	meiotic nuclear division	222	6.112E-03	4.503E-02	6.112E-03	4.503E-02	4	HSP70, HSPA2, CKS2, Cyclin B
251	negative regulation of molecular function	1218	6.139E-03	4.503E-02	6.139E-03	4.503E-02	10	Ajuba, HSPA1B, HEXIM1, PAP41, HSP70, ERK1 (MAPK3), HSPA1A, Spinophilin, CAMK2N1, ERK1/2
252	single organism reproductive process	1418	6.150E-03	4.503E-02	6.150E-03	4.503E-02	11	Cyclin B1, HSPA1B, AL1A1, CDO1, Olfactory receptor, Calmyrin, HSP70, HSPA1A, HSPA2, Cyclin B, DEAF
253	regulation of biological process	12391	6.250E-03	4.527E-02	6.250E-03	4.527E-02	51	Ajuba, IGHG4, ZNF261, Cyclin B1, HSPA1B, TIPIN, Clp1, ASAP, AL1A1, ARL5B, HBG, HBGA, SNX1, HEXIM1, TIRAP (Mal), Olfactory receptor, PTD015, Thioredoxin-like 2, Rab-3B, Calmyrin, PAP41, EPS8L1, COLEC12, IGHG1, HSP70, ERK1 (MAPK3), Annexin VI, eEF2K, SHCBP1, HSPA1A, CD43, HSPA2, AO7, CKS2, OR10G3, OBFC1, ZNF397OS, ICMT, ZNF397, Spinophilin, Cyclin B, Rab-3, Adult hemoglobin, EYA1, RIG-G, CAMK2N1, ZHX3, ERK1/2, DEAF, PRCC, PQBP-1
254	hemostasis	670	6.291E	4.527E	6.291E	4.527E	7	HBG, HBGA, HSP70, ERK1 (MAPK3), CD43, Adult hemoglobin, ERK1/2

			-03	-02	-03	-02		
255	regulation of body fluid levels	847	6.379E-03	4.527E-02	6.379E-03	4.527E-02	8	HBG, HBGA, CDO1, HSP70, ERK1 (MAPK3), CD43, Adult hemoglobin, ERK1/2
256	negative regulation of fibril organization	2	6.552E-03	4.527E-02	6.552E-03	4.527E-02	1	HSP70
257	regulation of fibril organization	2	6.552E-03	4.527E-02	6.552E-03	4.527E-02	1	HSP70
258	GDP metabolic process	2	6.552E-03	4.527E-02	6.552E-03	4.527E-02	1	FLJ22494
259	taurine biosynthetic process	2	6.552E-0	4.527E-0	6.552E-0	4.527E-0	1	CDO1

			3	2	3	2			
260	positive regulation of male germ cell proliferation	2	6.552E-03	4.527E-02	6.552E-03	4.527E-02	1	Calmyrin	
261	fructosamine metabolic process	2	6.552E-03	4.527E-02	6.552E-03	4.527E-02	1	Fructosamine-3-kinase	
262	L-cysteine catabolic process to taurine	2	6.552E-03	4.527E-02	6.552E-03	4.527E-02	1	CDO1	
263	thrombopoietin-mediated signaling pathway	2	6.552E-03	4.527E-02	6.552E-03	4.527E-02	1	Calmyrin	
264	regulation of protein folding in endoplasmic reticulum	2	6.552E-03	4.527E-02	6.552E-03	4.527E-02	1	HSP70	

265	maintenance of presynaptic active zone structure	2	6.552E-03	4.527E-02	6.552E-03	4.527E-02	1	Rab-3
266	small RNA loading onto RISC	2	6.552E-03	4.527E-02	6.552E-03	4.527E-02	1	Clp1
267	T cell aggregation	2	6.552E-03	4.527E-02	6.552E-03	4.527E-02	1	CD43
268	transcription initiation from RNA polymerase I promoter	38	6.911E-03	4.757E-02	6.911E-03	4.757E-02	2	ERK1 (MAPK3), ERK1/2
269	protein methylation	119	7.054E-03	4.814E-02	7.054E-03	4.814E-02	3	Rab-3B, ICMT, Rab-3
27	protein alkylation	11	7.	4.	7.	4.	3	Rab-3B, ICMT, Rab-3

0		9	054E-03	814E-02	054E-03	814E-02		
271	negative regulation of protein modification process	519	7.071E-03	4.814E-02	7.071E-03	4.814E-02	6	Cyclin B1, HEXIM1, Calmyrin, HSP70, Cyclin B, CAMK2N1
272	activation of immune response	520	7.136E-03	4.840E-02	7.136E-03	4.840E-02	6	IGHG4, TIRAP (Mal), COLEC12, IGHG1, ERK1 (MAPK3), ERK1/2
273	innate immune response	1051	7.170E-03	4.846E-02	7.170E-03	4.846E-02	9	IGHM, IGHG4, TIRAP (Mal), COLEC12, IGHG1, ERK1 (MAPK3), CALCOCO2, RIG-G, ERK1/2
274	regulation of DNA metabolic process	369	7.259E-03	4.873E-02	7.259E-03	4.873E-02	5	Cyclin B1, TIPIN, OBFC1, Cyclin B, EYA1
275	regulation of chromosome segregation	39	7.26	4.87	7.26	4.87	2	Cyclin B1, Cyclin B

			9E-03	3E-02	9E-03	3E-02		
276	positive regulation of cellular protein metabolic process	1249	7.290E-03	4.873E-02	7.290E-03	4.873E-02	10	Ajuba, Cyclin B1, TIRAP (Mal), Calmyrin, HSP70, ERK1 (MAPK3), CD43, HSPA2, Cyclin B, ERK1/2
277	positive regulation of adaptive immune response based on somatic recombination of immune receptors built from immunoglobulin superfamily domains	121	7.384E-03	4.901E-02	7.384E-03	4.901E-02	3	HSPA1B, HSP70, HSPA1A
278	positive regulation of lymphocyte mediated immunity	121	7.384E-03	4.901E-02	7.384E-03	4.901E-02	3	HSPA1B, HSP70, HSPA1A
279	meiotic cell cycle	235	7.446E-03	4.924E-02	7.446E-03	4.924E-02	4	HSP70, HSPA2, CKS2, Cyclin B

Table 9S: Enrichment analysis report. Enrichment by GO Processes (healthy control vs SVZp)								
Enrichment by GO Processes					SVZp Limma Input_genelist			
**#**	**Processes**	**Total**	**pValue**	**Min FDR**	**p-value**	**FDR**	**In Data**	**Network Objects from Active Data**
1	positive regulation of histone phosphorylation	8	6.915E-09	1.536E-05	6.915E-09	1.536E-05	4	Cyclin B1, ERK1 (MAPK3), Cyclin B, ERK1/2
2	regulation of histone phosphorylation	11	3.236E-08	3.594E-05	3.236E-08	3.594E-05	4	Cyclin B1, ERK1 (MAPK3), Cyclin B, ERK1/2
3	wound healing	857	4.889E-07	3.619E-04	4.889E-07	3.619E-04	14	Ajuba, Tissue kallikreins, Cyclin B1, HBG, HBGA, Notch, ERK1 (MAPK3), NOTCH1 precursor, CD43, 14-3-3 zeta/delta, Cyclin B, Adult hemoglobin, ERK1/2, 14-3-3
4	cardiac left ventricle morphogenesis	24	1.010E-06	4.485E-04	1.010E-06	4.485E-04	4	Tissue kallikreins, Notch, NOTCH1 precursor, Kallikrein 3 (PSA)

5	positive regulation of cardiac muscle cell proliferation	24	1.010E-06	4.485E-04	1.010E-06	4.485E-04	4	Cyclin B1, Notch, NOTCH1 precursor, Cyclin B
6	response to wounding	1450	2.469E-06	9.140E-04	2.469E-06	9.140E-04	17	Ajuba, Tissue kallikreins, Cyclin B1, HBG, IRT-1, HBGA, TIRAP (Mal), Notch, ERK1 (MAPK3), NOTCH1 precursor, CD43, 14-3-3 zeta/delta, Cyclin B, Kallikrein 3 (PSA), Adult hemoglobin, ERK1/2, 14-3-3
7	small GTPase mediated signal transduction	500	4.417E-06	1.270E-03	4.417E-06	1.270E-03	10	Tissue kallikreins, IRT-1, Rab-11A, Rab-3B, ERK1 (MAPK3), PLEKHG2, Rab-3, Kallikrein 3 (PSA), ERK1/2, 14-3-3
8	tissue regeneration	77	5.008E-06	1.270E-03	5.008E-06	1.270E-03	5	Tissue kallikreins, Cyclin B1, Notch, NOTCH1 precursor, Cyclin B
9	neuron projection development	900	5.156E-06	1.270E-03	5.156E-06	1.270E-03	13	Tissue kallikreins, LST1, LHX1, Rab-11A, Olfactory receptor, Notch, ERK1 (MAPK3), NOTCH1 precursor, Spinophilin, Rab-3, Protrudin, ERK1/2, 14-3-3
10	positive regulation of biological process	52	6.	1.	6.	1.	34	Ajuba, Tissue kallikreins, IGHG4, Kallikrein 1, Cyclin B1, TIPIN, AL1A1, LHX1, IRT-1, Gpbp1, Rab-11A, SNX1, TEF-5,

		49	860E-06	270E-03	860E-06	270E-03		TIRAP (Mal), Olfactory receptor, PTD015, Notch, Rab-3B, IGHG1, ERK1 (MAPK3), SRB7, NOTCH1 precursor, CD43, 14-3-3 zeta/delta, OBFC1, PLEKHG2, Cyclin B, Rab-3, Kallikrein 3 (PSA), Adult hemoglobin, ZHX3, ERK1/2, DEAF, 14-3-3
11	cell projection organization	1231	6.901E-06	1.270E-03	6.901E-06	1.270E-03	15	Ajuba, Tissue kallikreins, LST1, LHX1, IRT-1, Rab-11A, Olfactory receptor, Notch, ERK1 (MAPK3), NOTCH1 precursor, Spinophilin, Rab-3, Protrudin, ERK1/2, 14-3-3
12	regulation of cardiac muscle cell proliferation	42	1.018E-05	1.270E-03	1.018E-05	1.270E-03	4	Cyclin B1, Notch, NOTCH1 precursor, Cyclin B
13	Notch signaling pathway involved in regulation of secondary heart field cardioblast proliferation	2	1.030E-05	1.270E-03	1.030E-05	1.270E-03	2	Notch, NOTCH1 precursor
14	regulation of apolipoprotein binding	2	1.030E-05	1.270E-03	1.030E-05	1.270E-03	2	ERK1 (MAPK3), ERK1/2
15	response to DDT	2	1.03	1.27	1.03	1.27	2	Cyclin B1, Cyclin B

			0E-05	0E-03	0E-05	0E-03		
16	venous endothelial cell differentiation	2	1.030E-05	1.270E-03	1.030E-05	1.270E-03	2	Notch, NOTCH1 precursor
17	regulation of vesicle size	2	1.030E-05	1.270E-03	1.030E-05	1.270E-03	2	Rab-3B, Rab-3
18	negative regulation of apolipoprotein binding	2	1.030E-05	1.270E-03	1.030E-05	1.270E-03	2	ERK1 (MAPK3), ERK1/2
19	interleukin-1-mediated signaling pathway	17	2.126E-05	2.485E-03	2.126E-05	2.485E-03	3	TIRAP (Mal), ERK1 (MAPK3), ERK1/2
20	positive regulation of epithelial cell differentiation	52	2.403E	2.536E	2.403E	2.536E	4	LHX1, Notch, NOTCH1 precursor, 14-3-3

			-05	-03	-05	-03		
21	regulation of cell adhesion involved in heart morphogenesis	3	3.083E-05	2.536E-03	3.083E-05	2.536E-03	2	Notch, NOTCH1 precursor
22	positive regulation of ephrin receptor signaling pathway	3	3.083E-05	2.536E-03	3.083E-05	2.536E-03	2	Notch, NOTCH1 precursor
23	bradykinin biosynthetic process	3	3.083E-05	2.536E-03	3.083E-05	2.536E-03	2	Tissue kallikreins, Kallikrein 3 (PSA)
24	mitotic spindle stabilization	3	3.083E-05	2.536E-03	3.083E-05	2.536E-03	2	Cyclin B1, Cyclin B
25	coronary vein morphogenesis	3	3.083E-0	2.536E-0	3.083E-0	2.536E-0	2	Notch, NOTCH1 precursor

			5	3	5	3		
26	tissue kallikrein-kinin cascade	3	3.083E-05	2.536E-03	3.083E-05	2.536E-03	2	Tissue kallikreins, Kallikrein 3 (PSA)
27	regulation of extracellular matrix assembly	3	3.083E-05	2.536E-03	3.083E-05	2.536E-03	2	Notch, NOTCH1 precursor
28	regulation of cardiac muscle tissue growth	56	3.229E-05	2.561E-03	3.229E-05	2.561E-03	4	Cyclin B1, Notch, NOTCH1 precursor, Cyclin B
29	oxygen transport	20	3.540E-05	2.711E-03	3.540E-05	2.711E-03	3	HBG, HBGA, Adult hemoglobin
30	positive regulation of epidermal cell differentiation	22	4.760E-05	3.333E-03	4.760E-05	3.333E-03	3	Notch, NOTCH1 precursor, 14-3-3

31	neuron development	115	4.963E-05	3.333E-03	4.963E-05	3.333E-03	13	Tissue kallikreins, LST1, LHX1, Rab-11A, Olfactory receptor, Notch, ERK1 (MAPK3), NOTCH1 precursor, Spinophilin, Rab-3, Protrudin, ERK1/2, 14-3-3
32	cell development	2031	5.530E-05	3.333E-03	5.530E-05	3.333E-03	18	Tissue kallikreins, LST1, Cyclin B1, LHX1, Rab-11A, Olfactory receptor, Notch, ERK1 (MAPK3), SRB7, NOTCH1 precursor, Spinophilin, Cyclin B, Rab-3, Adult hemoglobin, Protrudin, ERK1/2, DEAF, 14-3-3
33	cellular response to iron(III) ion	4	6.152E-05	3.333E-03	6.152E-05	3.333E-03	2	Cyclin B1, Cyclin B
34	atrioventricular node development	4	6.152E-05	3.333E-03	6.152E-05	3.333E-03	2	Notch, NOTCH1 precursor
35	negative regulation of pro-B cell differentiation	4	6.152E-05	3.333E-03	6.152E-05	3.333E-03	2	Notch, NOTCH1 precursor
36	apoptotic process involved in embryonic digit	4	6.	3.	6.	3.	2	Notch, NOTCH1 precursor

	morphogenesis		152E-05	33E-03	152E-05	33E-03		
37	cardiac right atrium morphogenesis	4	6.152E-05	3.333E-03	6.152E-05	3.333E-03	2	Notch, NOTCH1 precursor
38	cell migration involved in endocardial cushion formation	4	6.152E-05	3.333E-03	6.152E-05	3.333E-03	2	Notch, NOTCH1 precursor
39	left ventricular cardiac muscle tissue morphogenesis	4	6.152E-05	3.333E-03	6.152E-05	3.333E-03	2	Tissue kallikreins, Kallikrein 3 (PSA)
40	regulation of ephrin receptor signaling pathway	4	6.152E-05	3.333E-03	6.152E-05	3.333E-03	2	Notch, NOTCH1 precursor
41	negative regulation of endothelial cell chemotaxis	4	6.15	3.33	6.15	3.33	2	Notch, NOTCH1 precursor

			2E-05	3E-03	2E-05	3E-03		
42	regulation of heart growth	67	6.561E-05	3.431E-03	6.561E-05	3.431E-03	4	Cyclin B1, Notch, NOTCH1 precursor, Cyclin B
43	positive regulation of cellular biosynthetic process	1865	6.643E-05	3.431E-03	6.643E-05	3.431E-03	17	Ajuba, LHX1, IRT-1, Gpbp1, TEF-5, TIRAP (Mal), PTD015, Notch, ERK1 (MAPK3), SRB7, NOTCH1 precursor, CD43, OBFC1, Adult hemoglobin, ERK1/2, DEAF, 14-3-3
44	gas transport	25	7.059E-05	3.563E-03	7.059E-05	3.563E-03	3	HBG, HBGA, Adult hemoglobin
45	positive regulation of histone modification	69	7.364E-05	3.592E-03	7.364E-05	3.592E-03	4	Cyclin B1, ERK1 (MAPK3), Cyclin B, ERK1/2
46	regulation of cell proliferation	1882	7.440E	3.592E	7.440E	3.592E	17	Tissue kallikreins, Kallikrein 1, LST1, Cyclin B1, TIPIN, LHX1, IRT-1, TIRAP (Mal), Notch, NOTCH1 precursor, CD43, Spinophilin, Cyclin B, RIG-G, ERK1/2, DEAF, 14-3-3

			-05	-03	-05	-03		
47	regulation of cardiac muscle tissue development	70	7.791E-05	3.682E-03	7.791E-05	3.682E-03	4	Cyclin B1, Notch, NOTCH1 precursor, Cyclin B
48	positive regulation of biosynthetic process	1902	8.486E-05	3.926E-03	8.486E-05	3.926E-03	17	Ajuba, LHX1, IRT-1, Gpbp1, TEF-5, TIRAP (Mal), PTD015, Notch, ERK1 (MAPK3), SRB7, NOTCH1 precursor, CD43, OBFC1, Adult hemoglobin, ERK1/2, DEAF, 14-3-3
49	intracellular signal transduction	1918	9.414E-05	4.208E-03	9.414E-05	4.208E-03	17	Tissue kallikreins, Kallikrein 1, IRT-1, Rab-11A, TEF-5, TIRAP (Mal), Rab-3B, ERK1 (MAPK3), 14-3-3 zeta/delta, NFKBIL1, CKS2, PLEKHG2, Spinophilin, Rab-3, Kallikrein 3 (PSA), ERK1/2, 14-3-3
50	ventricular cardiac muscle tissue morphogenesis	74	9.683E-05	4.208E-03	9.683E-05	4.208E-03	4	Tissue kallikreins, Notch, NOTCH1 precursor, Kallikrein 3 (PSA)
51	spindle stabilization	5	1.023E-0	4.208E-0	1.023E-0	4.208E-0	2	Cyclin B1, Cyclin B

			4	3	4	3		
52	negative regulation of photoreceptor cell differentiation	5	1.023E-04	4.208E-03	1.023E-04	4.208E-03	2	Notch, NOTCH1 precursor
53	mitral valve formation	5	1.023E-04	4.208E-03	1.023E-04	4.208E-03	2	Notch, NOTCH1 precursor
54	peptidyl-cysteine methylation	5	1.023E-04	4.208E-03	1.023E-04	4.208E-03	2	Rab-3B, Rab-3
55	negative regulation of gene expression	1390	1.185E-04	4.361E-03	1.185E-04	4.361E-03	14	Ajuba, Cyclin B1, LHX1, HBG, IRT-1, HBGA, Notch, DR1, NOTCH1 precursor, Cyclin B, Adult hemoglobin, ZHX3, 14-3-3, RBBP7 (RbAp46)
56	negative regulation of chemotaxis	30	1.232E-04	4.361E-03	1.232E-04	4.361E-03	3	IRT-1, Notch, NOTCH1 precursor

57	positive regulation of nitrogen compound metabolic process	1779	1.343E-04	4.361E-03	1.343E-04	4.361E-03	16	Cyclin B1, LHX1, IRT-1, Gpbp1, TEF-5, PTD015, Notch, ERK1 (MAPK3), SRB7, NOTCH1 precursor, OBFC1, Cyclin B, Adult hemoglobin, ERK1/2, DEAF, 14-3-3
58	immune response	1590	1.346E-04	4.361E-03	1.346E-04	4.361E-03	15	IGHG4, LST1, IRT-1, TIRAP (Mal), Notch, IGHG1, ERK1 (MAPK3), CALCOCO2, NOTCH1 precursor, CD43, 14-3-3 zeta/delta, G18, RIG-G, ERK1/2, 14-3-3
59	regulation of primary metabolic process	6540	1.355E-04	4.361E-03	1.355E-04	4.361E-03	36	Ajuba, Tissue kallikreins, Cyclin B1, TIPIN, AL1A1, LHX1, HBG, IRT-1, HBGA, Gpbp1, SNX1, TEF-5, TIRAP (Mal), Olfactory receptor, PTD015, Notch, ERK1 (MAPK3), DR1, SRB7, NOTCH1 precursor, CD43, NFKBIL1, AO7, CKS2, OBFC1, PLEKHG2, Spinophilin, Cyclin B, Rab-3, Kallikrein 3 (PSA), Adult hemoglobin, ZHX3, ERK1/2, DEAF, 14-3-3, RBBP7 (RbAp46)
60	cardiac muscle tissue development	244	1.372E-04	4.361E-03	1.372E-04	4.361E-03	6	Tissue kallikreins, Cyclin B1, Notch, NOTCH1 precursor, Cyclin B, Kallikrein 3 (PSA)
61	ventricular cardiac muscle tissue development	81	1.377E-04	4.361E-03	1.377E-04	4.361E-03	4	Tissue kallikreins, Notch, NOTCH1 precursor, Kallikrein 3 (PSA)
62	positive regulation of metabolic process	30	1.	4.	1.	4.	22	Ajuba, Tissue kallikreins, Cyclin B1, LHX1, IRT-1, Gpbp1, SNX1, TEF-5, TIRAP (Mal), PTD015, Notch, ERK1 (MAPK3),

		38	411E-04	361E-03	411E-04	361E-03		SRB7, NOTCH1 precursor, CD43, OBFC1, Cyclin B, Kallikrein 3 (PSA), Adult hemoglobin, ERK1/2, DEAF, 14-3-3
63	phagocytosis	248	1.499E-04	4.361E-03	1.499E-04	4.361E-03	6	IGHG4, IRT-1, IGHG1, ERK1 (MAPK3), Annexin XI, ERK1/2
64	positive regulation of chromosome organization	83	1.513E-04	4.361E-03	1.513E-04	4.361E-03	4	Cyclin B1, ERK1 (MAPK3), Cyclin B, ERK1/2
65	kinin cascade	6	1.532E-04	4.361E-03	1.532E-04	4.361E-03	2	Tissue kallikreins, Kallikrein 3 (PSA)
66	positive regulation of dopamine uptake involved in synaptic transmission	6	1.532E-04	4.361E-03	1.532E-04	4.361E-03	2	Rab-3B, Rab-3
67	osteoblast fate commitment	6	1.53	4.36	1.53	4.36	2	Notch, NOTCH1 precursor

			2E-04	1E-03	2E-04	1E-03		
68	positive regulation of catecholamine uptake involved in synaptic transmission	6	1.532E-04	4.361E-03	1.532E-04	4.361E-03	2	Rab-3B, Rab-3
69	regulation of Golgi inheritance	6	1.532E-04	4.361E-03	1.532E-04	4.361E-03	2	ERK1 (MAPK3), ERK1/2
70	positive regulation of transcription of Notch receptor target	6	1.532E-04	4.361E-03	1.532E-04	4.361E-03	2	Notch, NOTCH1 precursor
71	arterial endothelial cell differentiation	6	1.532E-04	4.361E-03	1.532E-04	4.361E-03	2	Notch, NOTCH1 precursor
72	positive regulation of attachment of spindle microtubules to kinetochore	6	1.532E	4.361E	1.532E	4.361E	2	Cyclin B1, Cyclin B

			-04	-03	-04	-03		
73	MAPK import into nucleus	6	1.532E-04	4.361E-03	1.532E-04	4.361E-03	2	ERK1 (MAPK3), ERK1/2
74	positive regulation of transcription from RNA polymerase II promoter in response to hypoxia	6	1.532E-04	4.361E-03	1.532E-04	4.361E-03	2	Notch, NOTCH1 precursor
75	regulation of pro-B cell differentiation	6	1.532E-04	4.361E-03	1.532E-04	4.361E-03	2	Notch, NOTCH1 precursor
76	positive regulation of neurotransmitter uptake	6	1.532E-04	4.361E-03	1.532E-04	4.361E-03	2	Rab-3B, Rab-3
77	interleukin-4 secretion	6	1.532E-0	4.361E-0	1.532E-0	4.361E-0	2	Notch, NOTCH1 precursor

			4	3	4	3		
78	regulation of chromosome condensation	6	1.532E-04	4.361E-03	1.532E-04	4.361E-03	2	Cyclin B1, Cyclin B
79	cardiac cell development	84	1.585E-04	4.456E-03	1.585E-04	4.456E-03	4	Cyclin B1, Notch, NOTCH1 precursor, Cyclin B
80	inflammatory response	621	1.668E-04	4.631E-03	1.668E-04	4.631E-03	9	Tissue kallikreins, IRT-1, TIRAP (Mal), Notch, NOTCH1 precursor, CD43, 14-3-3 zeta/delta, Kallikrein 3 (PSA), 14-3-3
81	defense response	1833	1.899E-04	5.139E-03	1.899E-04	5.139E-03	16	Tissue kallikreins, IGHG4, IRT-1, TIRAP (Mal), Olfactory receptor, Notch, IGHG1, ERK1 (MAPK3), CALCOCO2, NOTCH1 precursor, CD43, 14-3-3 zeta/delta, Kallikrein 3 (PSA), RIG-G, ERK1/2, 14-3-3
82	negative regulation of response to external stimulus	262	2.018E-04	5.139E-03	2.018E-04	5.139E-03	6	Tissue kallikreins, IRT-1, Notch, NOTCH1 precursor, CD43, NFKBIL1

83	positive regulation of epidermis development	36	2.136E-04	5.139E-03	2.136E-04	5.139E-03	3	Notch, NOTCH1 precursor, 14-3-3
84	growth involved in heart morphogenesis	7	2.140E-04	5.139E-03	2.140E-04	5.139E-03	2	Notch, NOTCH1 precursor
85	response to iron(III) ion	7	2.140E-04	5.139E-03	2.140E-04	5.139E-03	2	Cyclin B1, Cyclin B
86	pericardium morphogenesis	7	2.140E-04	5.139E-03	2.140E-04	5.139E-03	2	Notch, NOTCH1 precursor
87	endocardial cell differentiation	7	2.140E-04	5.139E-03	2.140E-04	5.139E-03	2	Notch, NOTCH1 precursor
88	positive regulation of chromosome segregation	7	2.	5.	2.	5.	2	Cyclin B1, Cyclin B

			140E-04	139E-03	140E-04	139E-03		
89	cardiac right ventricle formation	7	2.140E-04	5.139E-03	2.140E-04	5.139E-03	2	Notch, NOTCH1 precursor
90	aortic valve morphogenesis	7	2.140E-04	5.139E-03	2.140E-04	5.139E-03	2	Notch, NOTCH1 precursor
91	caveolin-mediated endocytosis	7	2.140E-04	5.139E-03	2.140E-04	5.139E-03	2	ERK1 (MAPK3), ERK1/2
92	aortic valve development	7	2.140E-04	5.139E-03	2.140E-04	5.139E-03	2	Notch, NOTCH1 precursor
93	positive regulation of cell proliferation	116	2.15	5.13	2.15	5.13	12	Tissue kallikreins, Kallikrein 1, Cyclin B1, TIPIN, LHX1, IRT-1, TIRAP (Mal), Notch, NOTCH1 precursor, CD43, Cyclin B, ERK1/2

			2E-04	9E-03	2E-04	9E-03		
94	regulation of cellular metabolic process	6446	2.522E-04	5.910E-03	2.522E-04	5.910E-03	35	Ajuba, Tissue kallikreins, Cyclin B1, TIPIN, AL1A1, LHX1, HBG, IRT-1, HBGA, Gpbp1, TEF-5, TIRAP (Mal), Olfactory receptor, PTD015, Notch, ERK1 (MAPK3), DR1, SRB7, NOTCH1 precursor, CD43, NFKBIL1, AO7, CKS2, OBFC1, PLEKHG2, Spinophilin, Cyclin B, Rab-3, Kallikrein 3 (PSA), Adult hemoglobin, ZHX3, ERK1/2, DEAF, 14-3-3, RBBP7 (RbAp46)
95	cardiac muscle tissue morphogenesis	95	2.546E-04	5.910E-03	2.546E-04	5.910E-03	4	Tissue kallikreins, Notch, NOTCH1 precursor, Kallikrein 3 (PSA)
96	regulation of nitrogen compound metabolic process	5112	2.557E-04	5.910E-03	2.557E-04	5.910E-03	30	Ajuba, Cyclin B1, TIPIN, AL1A1, LHX1, HBG, IRT-1, HBGA, Gpbp1, TEF-5, TIRAP (Mal), Olfactory receptor, PTD015, Notch, ERK1 (MAPK3), DR1, SRB7, NOTCH1 precursor, NFKBIL1, AO7, OBFC1, PLEKHG2, Cyclin B, Rab-3, Adult hemoglobin, ZHX3, ERK1/2, DEAF, 14-3-3, RBBP7 (RbAp46)
97	regulation of metabolic process	7302	2.607E-04	5.910E-03	2.607E-04	5.910E-03	38	Ajuba, Tissue kallikreins, Cyclin B1, TIPIN, AL1A1, LHX1, HBG, IRT-1, HBGA, Gpbp1, SNX1, TEF-5, TIRAP (Mal), Olfactory receptor, PTD015, Notch, PAP41, ERK1 (MAPK3), DR1, SRB7, NOTCH1 precursor, CD43, NFKBIL1, AO7, CKS2, OBFC1, PLEKHG2, G18, Spinophilin, Cyclin B, Rab-3, Kallikrein 3 (PSA), Adult hemoglobin, ZHX3, ERK1/2, DEAF, 14-3-3, RBBP7 (RbAp46)
98	negative regulation of angiogenesis	96	2.650E	5.910E	2.650E	5.910E	4	Tissue kallikreins, Notch, NOTCH1 precursor, Kallikrein 3 (PSA)

			-04	-03	-04	-03		
99	interleukin-4 production	8	2.847E-04	5.910E-03	2.847E-04	5.910E-03	2	Notch, NOTCH1 precursor
100	regulation of transcription from RNA polymerase II promoter involved in myocardial precursor cell differentiation	8	2.847E-04	5.910E-03	2.847E-04	5.910E-03	2	Notch, NOTCH1 precursor
101	regulation of photoreceptor cell differentiation	8	2.847E-04	5.910E-03	2.847E-04	5.910E-03	2	Notch, NOTCH1 precursor
102	endocardium morphogenesis	8	2.847E-04	5.910E-03	2.847E-04	5.910E-03	2	Notch, NOTCH1 precursor
103	auditory receptor cell fate commitment	8	2.847E-0	5.910E-0	2.847E-0	5.910E-0	2	Notch, NOTCH1 precursor

			4	3	4	3		
104	compartment pattern specification	8	2.847E-04	5.910E-03	2.847E-04	5.910E-03	2	Notch, NOTCH1 precursor
105	inner ear receptor cell fate commitment	8	2.847E-04	5.910E-03	2.847E-04	5.910E-03	2	Notch, NOTCH1 precursor
106	regulation of early endosome to late endosome transport	8	2.847E-04	5.910E-03	2.847E-04	5.910E-03	2	ERK1 (MAPK3), ERK1/2
107	cardiac vascular smooth muscle cell development	8	2.847E-04	5.910E-03	2.847E-04	5.910E-03	2	Notch, NOTCH1 precursor
108	regulation of cellular component organization	2107	2.942E-04	6.050E-03	2.942E-04	6.050E-03	17	Ajuba, Tissue kallikreins, ZNF261, LST1, Cyclin B1, Rab-11A, Olfactory receptor, Notch, ERK1 (MAPK3), NOTCH1 precursor, 14-3-3 zeta/delta, Spinophilin, Cyclin B, Rab-3, ERK1/2, 14-3-3, RBBP7 (RbAp46)

109	digestive tract development	181	2.997E-04	6.106E-03	2.997E-04	6.106E-03	5	Cyclin B1, AL1A1, Notch, NOTCH1 precursor, Cyclin B
110	response to stress	4156	3.115E-04	6.290E-03	3.115E-04	6.290E-03	26	Ajuba, Tissue kallikreins, IGHG4, Cyclin B1, TIPIN, AL1A1, HBG, IRT-1, HBGA, TIRAP (Mal), Olfactory receptor, Notch, IGHG1, ERK1 (MAPK3), CALCOCO2, NOTCH1 precursor, CD43, 14-3-3 zeta/delta, Cyclin B, Kallikrein 3 (PSA), Adult hemoglobin, RIG-G, ERK1/2, 14-3-3, CapZIP, RBBP7 (RbAp46)
111	neuron differentiation	1346	3.251E-04	6.504E-03	3.251E-04	6.504E-03	13	Tissue kallikreins, LST1, LHX1, Rab-11A, Olfactory receptor, Notch, ERK1 (MAPK3), NOTCH1 precursor, Spinophilin, Rab-3, Protrudin, ERK1/2, 14-3-3
112	negative regulation of biological process	4668	3.285E-04	6.514E-03	3.285E-04	6.514E-03	28	Ajuba, Tissue kallikreins, LST1, Cyclin B1, TIPIN, LHX1, HBG, IRT-1, HBGA, TIRAP (Mal), PTD015, Notch, Thioredoxin-like 2, DR1, SRB7, NOTCH1 precursor, CD43, 14-3-3 zeta/delta, NFKBIL1, Spinophilin, Cyclin B, Kallikrein 3 (PSA), Adult hemoglobin, RIG-G, ZHX3, ERK1/2, 14-3-3, RBBP7 (RbAp46)
113	positive regulation of cellular process	4675	3.372E-04	6.628E-03	3.372E-04	6.628E-03	28	Ajuba, Tissue kallikreins, Kallikrein 1, Cyclin B1, TIPIN, AL1A1, LHX1, IRT-1, Gpbp1, Rab-11A, TEF-5, TIRAP (Mal), Olfactory receptor, PTD015, Notch, ERK1 (MAPK3), SRB7, NOTCH1 precursor, CD43, 14-3-3 zeta/delta, OBFC1, PLEKHG2, Cyclin B, Adult hemoglobin, ZHX3, ERK1/2, DEAF, 14-3-3
11	regulation of phosphorylation	15	3.	6.	3.	6.	14	Ajuba, Tissue kallikreins, Cyclin B1, IRT-1, TIRAP (Mal), ERK1 (MAPK3), CD43, CKS2, Spinophilin, Cyclin B,

4		39	416E-04	650E-03	416E-04	650E-03		Kallikrein 3 (PSA), Adult hemoglobin, ERK1/2, 14-3-3
115	muscle tissue morphogenesis	104	3.598E-04	6.650E-03	3.598E-04	6.650E-03	4	Tissue kallikreins, Notch, NOTCH1 precursor, Kallikrein 3 (PSA)
116	blood vessel endothelial cell differentiation	9	3.653E-04	6.650E-03	3.653E-04	6.650E-03	2	Notch, NOTCH1 precursor
117	negative regulation of cell migration involved in sprouting angiogenesis	9	3.653E-04	6.650E-03	3.653E-04	6.650E-03	2	Notch, NOTCH1 precursor
118	histamine production involved in inflammatory response	9	3.653E-04	6.650E-03	3.653E-04	6.650E-03	2	14-3-3 zeta/delta, 14-3-3
119	histamine secretion by mast cell	9	3.65	6.65	3.65	6.65	2	14-3-3 zeta/delta, 14-3-3

			3E-04	0E-03	3E-04	0E-03		
120	peptidyl-tyrosine autophosphorylation	9	3.653E-04	6.650E-03	3.653E-04	6.650E-03	2	ERK1 (MAPK3), ERK1/2
121	coronary artery morphogenesis	9	3.653E-04	6.650E-03	3.653E-04	6.650E-03	2	Notch, NOTCH1 precursor
122	histamine secretion involved in inflammatory response	9	3.653E-04	6.650E-03	3.653E-04	6.650E-03	2	14-3-3 zeta/delta, 14-3-3
123	regulation of protein serine/threonine kinase activity	553	3.996E-04	7.206E-03	3.996E-04	7.206E-03	8	Ajuba, Cyclin B1, TIRAP (Mal), ERK1 (MAPK3), CKS2, Cyclin B, ERK1/2, 14-3-3
124	digestive system development	193	4.023E	7.206E	4.023E	7.206E	5	Cyclin B1, AL1A1, Notch, NOTCH1 precursor, Cyclin B

			-04	-03	-04	-03		
125	regulation of nucleobase-containing compound metabolic process	4986	4.128E-04	7.335E-03	4.128E-04	7.335E-03	29	Ajuba, Cyclin B1, TIPIN, AL1A1, LHX1, HBG, HBGA, Gpbp1, TEF-5, TIRAP (Mal), Olfactory receptor, PTD015, Notch, ERK1 (MAPK3), DR1, SRB7, NOTCH1 precursor, NFKBIL1, AO7, OBFC1, PLEKHG2, Cyclin B, Rab-3, Adult hemoglobin, ZHX3, ERK1/2, DEAF, 14-3-3, RBBP7 (RbAp46)
126	neuron projection morphogenesis	703	4.162E-04	7.336E-03	4.162E-04	7.336E-03	9	Tissue kallikreins, LHX1, Olfactory receptor, Notch, ERK1 (MAPK3), NOTCH1 precursor, Rab-3, ERK1/2, 14-3-3
127	regulation of mitotic spindle organization	10	4.557E-04	7.496E-03	4.557E-04	7.496E-03	2	Cyclin B1, Cyclin B
128	regulation of secondary heart field cardioblast proliferation	10	4.557E-04	7.496E-03	4.557E-04	7.496E-03	2	Notch, NOTCH1 precursor
129	regulation of cardioblast proliferation	10	4.557E-0	7.496E-0	4.557E-0	7.496E-0	2	Notch, NOTCH1 precursor

			4	3	4	3		
130	glomerular mesangial cell development	10	4.557E-04	7.496E-03	4.557E-04	7.496E-03	2	Notch, NOTCH1 precursor
131	negative regulation of glial cell proliferation	10	4.557E-04	7.496E-03	4.557E-04	7.496E-03	2	Notch, NOTCH1 precursor
132	mitral valve morphogenesis	10	4.557E-04	7.496E-03	4.557E-04	7.496E-03	2	Notch, NOTCH1 precursor
133	venous blood vessel morphogenesis	10	4.557E-04	7.496E-03	4.557E-04	7.496E-03	2	Notch, NOTCH1 precursor
134	pulmonary valve morphogenesis	10	4.557E-04	7.496E-03	4.557E-04	7.496E-03	2	Notch, NOTCH1 precursor

135	pulmonary valve development	10	4.557E-04	7.496E-03	4.557E-04	7.496E-03	2	Notch, NOTCH1 precursor
136	regulation of developmental growth	199	4.627E-04	7.556E-03	4.627E-04	7.556E-03	5	Cyclin B1, Rab-11A, Notch, NOTCH1 precursor, Cyclin B
137	cardiac ventricle morphogenesis	112	4.767E-04	7.672E-03	4.767E-04	7.672E-03	4	Tissue kallikreins, Notch, NOTCH1 precursor, Kallikrein 3 (PSA)
138	muscle organ morphogenesis	112	4.767E-04	7.672E-03	4.767E-04	7.672E-03	4	Tissue kallikreins, Notch, NOTCH1 precursor, Kallikrein 3 (PSA)
139	Ras protein signal transduction	201	4.843E-04	7.738E-03	4.843E-04	7.738E-03	5	IRT-1, ERK1 (MAPK3), Rab-3, ERK1/2, 14-3-3
14	regulation of organ growth	11	5.	8.	5.	8.	4	Cyclin B1, Notch, NOTCH1 precursor, Cyclin B

0		5	268E-04	174E-03	268E-04	174E-03		
141	cardiac muscle cell myoblast differentiation	11	5.558E-04	8.174E-03	5.558E-04	8.174E-03	2	Notch, NOTCH1 precursor
142	cell migration involved in heart development	11	5.558E-04	8.174E-03	5.558E-04	8.174E-03	2	Notch, NOTCH1 precursor
143	Notch signaling involved in heart development	11	5.558E-04	8.174E-03	5.558E-04	8.174E-03	2	Notch, NOTCH1 precursor
144	mitral valve development	11	5.558E-04	8.174E-03	5.558E-04	8.174E-03	2	Notch, NOTCH1 precursor
145	cardiac endothelial cell differentiation	11	5.55	8.17	5.55	8.17	2	Notch, NOTCH1 precursor

			8E-04	4E-03	8E-04	4E-03		
146	cellular response to iron ion	11	5.558E-04	8.174E-03	5.558E-04	8.174E-03	2	Cyclin B1, Cyclin B
147	epithelial to mesenchymal transition involved in endocardial cushion formation	11	5.558E-04	8.174E-03	5.558E-04	8.174E-03	2	Notch, NOTCH1 precursor
148	negative regulation of stem cell differentiation	11	5.558E-04	8.174E-03	5.558E-04	8.174E-03	2	Notch, NOTCH1 precursor
149	mesangial cell development	11	5.558E-04	8.174E-03	5.558E-04	8.174E-03	2	Notch, NOTCH1 precursor
150	glomerular mesangial cell differentiation	11	5.558E	8.174E	5.558E	8.174E	2	Notch, NOTCH1 precursor

			-04	-03	-04	-03		
151	positive regulation of endothelial cell differentiation	11	5.558E-04	8.174E-03	5.558E-04	8.174E-03	2	Notch, NOTCH1 precursor
152	regulation of cyclin-dependent protein serine/threonine kinase activity	119	5.993E-04	8.738E-03	5.993E-04	8.738E-03	4	Cyclin B1, CKS2, Cyclin B, 14-3-3
153	morphogenesis of an epithelial sheet	51	6.019E-04	8.738E-03	6.019E-04	8.738E-03	3	Ajuba, Notch, NOTCH1 precursor
154	regulation of epidermal cell differentiation	52	6.373E-04	9.068E-03	6.373E-04	9.068E-03	3	Notch, NOTCH1 precursor, 14-3-3
155	regulation of phosphate metabolic process	2257	6.565E-0	9.068E-0	6.565E-0	9.068E-0	17	Ajuba, Tissue kallikreins, Cyclin B1, AL1A1, IRT-1, TIRAP (Mal), ERK1 (MAPK3), CD43, CKS2, PLEKHG2, Spinophilin, Cyclin B, Rab-3, Kallikrein 3 (PSA), Adult hemoglobin, ERK1/2, 14-3-3

			4	3	4	3		
156	atrioventricular valve morphogenesis	12	6.655E-04	9.068E-03	6.655E-04	9.068E-03	2	Notch, NOTCH1 precursor
157	DNA damage induced protein phosphorylation	12	6.655E-04	9.068E-03	6.655E-04	9.068E-03	2	ERK1 (MAPK3), ERK1/2
158	regulation of dopamine uptake involved in synaptic transmission	12	6.655E-04	9.068E-03	6.655E-04	9.068E-03	2	Rab-3B, Rab-3
159	cellular response to morphine	12	6.655E-04	9.068E-03	6.655E-04	9.068E-03	2	IRT-1, Spinophilin
160	regulation of somitogenesis	12	6.655E-04	9.068E-03	6.655E-04	9.068E-03	2	Notch, NOTCH1 precursor

161	mesangial cell differentiation	12	6.655E-04	9.068E-03	6.655E-04	9.068E-03	2	Notch, NOTCH1 precursor
162	renal interstitial cell development	12	6.655E-04	9.068E-03	6.655E-04	9.068E-03	2	Notch, NOTCH1 precursor
163	regulation of catecholamine uptake involved in synaptic transmission	12	6.655E-04	9.068E-03	6.655E-04	9.068E-03	2	Rab-3B, Rab-3
164	immune system process	2697	6.700E-04	9.071E-03	6.700E-04	9.071E-03	19	IGHG4, LST1, IRT-1, TIRAP (Mal), Notch, Rab-3B, IGHG1, ERK1 (MAPK3), CALCOCO2, NOTCH1 precursor, CD43, 14-3-3 zeta/delta, G18, Cyclin B, Rab-3, Adult hemoglobin, RIG-G, ERK1/2, 14-3-3
165	epidermis morphogenesis	53	6.740E-04	9.071E-03	6.740E-04	9.071E-03	3	Tissue kallikreins, Notch, NOTCH1 precursor
166	regulation of protein phosphorylation	12	6.	9.	6.	9.	12	Ajuba, Cyclin B1, IRT-1, TIRAP (Mal), ERK1 (MAPK3), CD43, CKS2, Spinophilin, Cyclin B, Adult hemoglobin,

6		67	780E-04	071E-03	780E-04	071E-03		ERK1/2, 14-3-3
167	positive regulation of transferase activity	754	6.889E-04	9.162E-03	6.889E-04	9.162E-03	9	Ajuba, Tissue kallikreins, Cyclin B1, TIRAP (Mal), ERK1 (MAPK3), Cyclin B, Kallikrein 3 (PSA), ERK1/2, 14-3-3
168	regulation of phosphorus metabolic process	2275	7.189E-04	9.504E-03	7.189E-04	9.504E-03	17	Ajuba, Tissue kallikreins, Cyclin B1, AL1A1, IRT-1, TIRAP (Mal), ERK1 (MAPK3), CD43, CKS2, PLEKHG2, Spinophilin, Cyclin B, Rab-3, Kallikrein 3 (PSA), Adult hemoglobin, ERK1/2, 14-3-3
169	endocytosis	610	7.612E-04	9.849E-03	7.612E-04	9.849E-03	8	IGHG4, IRT-1, SNX1, IGHG1, ERK1 (MAPK3), Annexin XI, Rab-3, ERK1/2
170	neuronal stem cell maintenance	13	7.849E-04	9.849E-03	7.849E-04	9.849E-03	2	Notch, NOTCH1 precursor
171	negative regulation of calcium ion-dependent exocytosis	13	7.84	9.84	7.84	9.84	2	Notch, NOTCH1 precursor

			9E-04	9E-03	9E-04	9E-03		
172	renal interstitial cell differentiation	13	7.849E-04	9.849E-03	7.849E-04	9.849E-03	2	Notch, NOTCH1 precursor
173	regulation of attachment of spindle microtubules to kinetochore	13	7.849E-04	9.849E-03	7.849E-04	9.849E-03	2	Cyclin B1, Cyclin B
174	positive regulation of transcription from RNA polymerase II promoter in response to stress	13	7.849E-04	9.849E-03	7.849E-04	9.849E-03	2	Notch, NOTCH1 precursor
175	regulation of epithelial cell proliferation involved in prostate gland development	13	7.849E-04	9.849E-03	7.849E-04	9.849E-03	2	Notch, NOTCH1 precursor
176	ventricular trabecula myocardium morphogenesis	13	7.849E	9.849E	7.849E	9.849E	2	Notch, NOTCH1 precursor

			-04	-03	-04	-03		
177	cellular response to isoquinoline alkaloid	13	7.849E-04	9.849E-03	7.849E-04	9.849E-03	2	IRT-1, Spinophilin
178	nephron epithelium development	56	7.920E-04	9.883E-03	7.920E-04	9.883E-03	3	LHX1, Notch, NOTCH1 precursor
179	positive regulation of cellular component organization	936	8.049E-04	9.987E-03	8.049E-04	9.987E-03	10	Ajuba, Cyclin B1, Rab-11A, Notch, ERK1 (MAPK3), NOTCH1 precursor, 14-3-3 zeta/delta, Cyclin B, ERK1/2, 14-3-3
180	regulation of histone modification	129	8.110E-04	1.001E-02	8.110E-04	1.001E-02	4	Cyclin B1, ERK1 (MAPK3), Cyclin B, ERK1/2
181	positive regulation of macromolecule metabolic process	2743	8.263E-0	1.014E-0	8.263E-0	1.014E-0	19	Ajuba, Cyclin B1, LHX1, IRT-1, Gpbp1, SNX1, TEF-5, TIRAP (Mal), PTD015, Notch, ERK1 (MAPK3), SRB7, NOTCH1 precursor, CD43, OBFC1, Cyclin B, ERK1/2, DEAF, 14-3-3

			4	2	4	2		
182	positive regulation of neural precursor cell proliferation	58	8.776E-04	1.035E-02	8.776E-04	1.035E-02	3	LHX1, Notch, NOTCH1 precursor
183	vesicle-mediated transport	1305	8.791E-04	1.035E-02	8.791E-04	1.035E-02	12	IGHG4, IRT-1, Rab-11A, SNX1, TIP47, IGHG1, ERK1 (MAPK3), 14-3-3 zeta/delta, Annexin XI, Rab-3, ERK1/2, 14-3-3
184	protein activation cascade	132	8.837E-04	1.035E-02	8.837E-04	1.035E-02	4	Tissue kallikreins, IGHG4, IGHG1, Kallikrein 3 (PSA)
185	regulation of transcription from RNA polymerase II promoter	1897	8.928E-04	1.035E-02	8.928E-04	1.035E-02	15	Ajuba, HBG, HBGA, TEF-5, PTD015, Notch, ERK1 (MAPK3), DR1, SRB7, NOTCH1 precursor, Adult hemoglobin, ZHX3, ERK1/2, DEAF, RBBP7 (RbAp46)
186	heart valve formation	14	9.138E-04	1.035E-02	9.138E-04	1.035E-02	2	Notch, NOTCH1 precursor

187	endocardial cushion formation	14	9.138E-04	1.035E-02	9.138E-04	1.035E-02	2	Notch, NOTCH1 precursor
188	vascular smooth muscle cell development	14	9.138E-04	1.035E-02	9.138E-04	1.035E-02	2	Notch, NOTCH1 precursor
189	regulation of spindle organization	14	9.138E-04	1.035E-02	9.138E-04	1.035E-02	2	Cyclin B1, Cyclin B
190	endocardium development	14	9.138E-04	1.035E-02	9.138E-04	1.035E-02	2	Notch, NOTCH1 precursor
191	cardiac ventricle formation	14	9.138E-04	1.035E-02	9.138E-04	1.035E-02	2	Notch, NOTCH1 precursor
19	glomerular mesangium development	14	9.	1.	9.	1.	2	Notch, NOTCH1 precursor

2			138E-04	035E-02	138E-04	035E-02		
193	brain renin-angiotensin system	14	9.138E-04	1.035E-02	9.138E-04	1.035E-02	2	Tissue kallikreins, Kallikrein 3 (PSA)
194	negative regulation of hematopoietic progenitor cell differentiation	14	9.138E-04	1.035E-02	9.138E-04	1.035E-02	2	Notch, NOTCH1 precursor
195	atrioventricular valve development	14	9.138E-04	1.035E-02	9.138E-04	1.035E-02	2	Notch, NOTCH1 precursor
196	regulation of endothelial cell chemotaxis	14	9.138E-04	1.035E-02	9.138E-04	1.035E-02	2	Notch, NOTCH1 precursor
197	negative regulation of behavior	59	9.22	1.04	9.22	1.04	3	IRT-1, Notch, NOTCH1 precursor

			5E-04	0E-02	5E-04	0E-02		
198	immune response-regulating cell surface receptor signaling pathway involved in phagocytosis	134	9.346E-04	1.043E-02	9.346E-04	1.043E-02	4	IGHG4, IGHG1, ERK1 (MAPK3), ERK1/2
199	Fc-gamma receptor signaling pathway involved in phagocytosis	134	9.346E-04	1.043E-02	9.346E-04	1.043E-02	4	IGHG4, IGHG1, ERK1 (MAPK3), ERK1/2
200	axonogenesis	632	9.572E-04	1.062E-02	9.572E-04	1.062E-02	8	LHX1, Olfactory receptor, Notch, ERK1 (MAPK3), NOTCH1 precursor, Rab-3, ERK1/2, 14-3-3
201	Fc-gamma receptor signaling pathway	135	9.608E-04	1.062E-02	9.608E-04	1.062E-02	4	IGHG4, IGHG1, ERK1 (MAPK3), ERK1/2
202	positive regulation of macromolecule biosynthetic process	1708	9.659E	1.062E	9.659E	1.062E	14	LHX1, Gpbp1, TEF-5, TIRAP (Mal), PTD015, Notch, ERK1 (MAPK3), SRB7, NOTCH1 precursor, CD43, OBFC1, ERK1/2, DEAF, 14-3-3

			-04	-02	-04	-02		
203	Fc receptor mediated stimulatory signaling pathway	136	9.875E-04	1.080E-02	9.875E-04	1.080E-02	4	IGHG4, IGHG1, ERK1 (MAPK3), ERK1/2
204	regulation of kinase activity	966	1.024E-03	1.108E-02	1.024E-03	1.108E-02	10	Ajuba, Tissue kallikreins, Cyclin B1, TIRAP (Mal), ERK1 (MAPK3), CKS2, Cyclin B, Kallikrein 3 (PSA), ERK1/2, 14-3-3
205	regulation of catalytic activity	2567	1.024E-03	1.108E-02	1.024E-03	1.108E-02	18	Ajuba, Tissue kallikreins, Cyclin B1, AL1A1, TIRAP (Mal), Notch, PAP41, ERK1 (MAPK3), NOTCH1 precursor, CKS2, PLEKHG2, G18, Spinophilin, Cyclin B, Rab-3, Kallikrein 3 (PSA), ERK1/2, 14-3-3
206	positive regulation of RNA metabolic process	1522	1.037E-03	1.108E-02	1.037E-03	1.108E-02	13	Cyclin B1, LHX1, Gpbp1, TEF-5, PTD015, Notch, ERK1 (MAPK3), SRB7, NOTCH1 precursor, Cyclin B, ERK1/2, DEAF, 14-3-3
207	regulation of transcription from RNA polymerase II promoter involved in heart development	15	1.052E-0	1.108E-0	1.052E-0	1.108E-0	2	Notch, NOTCH1 precursor

			3	2	3	2		
208	negative regulation of oligodendrocyte differentiation	15	1.052E-03	1.108E-02	1.052E-03	1.108E-02	2	Notch, NOTCH1 precursor
209	cardiac chamber formation	15	1.052E-03	1.108E-02	1.052E-03	1.108E-02	2	Notch, NOTCH1 precursor
210	cardiac conduction system development	15	1.052E-03	1.108E-02	1.052E-03	1.108E-02	2	Notch, NOTCH1 precursor
211	foregut morphogenesis	15	1.052E-03	1.108E-02	1.052E-03	1.108E-02	2	Notch, NOTCH1 precursor
212	regulation of growth	802	1.065E-03	1.116E-02	1.065E-03	1.116E-02	9	Cyclin B1, Rab-11A, TIRAP (Mal), Notch, NOTCH1 precursor, Spinophilin, Cyclin B, 14-3-3, RBBP7 (RbAp46)

213	developmental growth	360	1.078E-03	1.123E-02	1.078E-03	1.123E-02	6	Tissue kallikreins, Cyclin B1, LHX1, Notch, NOTCH1 precursor, Cyclin B
214	positive regulation of nucleobase-containing compound metabolic process	1728	1.082E-03	1.123E-02	1.082E-03	1.123E-02	14	Cyclin B1, LHX1, Gpbp1, TEF-5, PTD015, Notch, ERK1 (MAPK3), SRB7, NOTCH1 precursor, OBFC1, Cyclin B, ERK1/2, DEAF, 14-3-3
215	regulation of chromatin organization	140	1.100E-03	1.131E-02	1.100E-03	1.131E-02	4	Cyclin B1, ERK1 (MAPK3), Cyclin B, ERK1/2
216	regulation of epithelial cell differentiation	140	1.100E-03	1.131E-02	1.100E-03	1.131E-02	4	LHX1, Notch, NOTCH1 precursor, 14-3-3
217	negative regulation of transcription from RNA polymerase II promoter	807	1.113E-03	1.139E-02	1.113E-03	1.139E-02	9	Ajuba, HBG, HBGA, Notch, DR1, NOTCH1 precursor, Adult hemoglobin, ZHX3, RBBP7 (RbAp46)
21	myeloid leukocyte activation	14	1.	1.	1.	1.	4	IRT-1, CD43, 14-3-3 zeta/delta, 14-3-3

8		1	1.29E-03	1.50E-02	1.29E-03	1.50E-02		
219	negative regulation of cellular process	4254	1.138E-03	1.154E-02	1.138E-03	1.154E-02	25	Ajuba, Tissue kallikreins, LST1, Cyclin B1, TIPIN, LHX1, HBG, IRT-1, HBGA, PTD015, Notch, DR1, SRB7, NOTCH1 precursor, CD43, 14-3-3 zeta/delta, NFKBIL1, Spinophilin, Cyclin B, Adult hemoglobin, RIG-G, ZHX3, ERK1/2, 14-3-3, RBBP7 (RbAp46)
220	negative regulation of cellular macromolecule biosynthetic process	1349	1.172E-03	1.184E-02	1.172E-03	1.184E-02	12	Ajuba, TIPIN, LHX1, HBG, HBGA, Notch, DR1, NOTCH1 precursor, Adult hemoglobin, ZHX3, 14-3-3, RBBP7 (RbAp46)
221	histamine secretion	16	1.200E-03	1.195E-02	1.200E-03	1.195E-02	2	14-3-3 zeta/delta, 14-3-3
222	positive regulation of keratinocyte differentiation	16	1.200E-03	1.195E-02	1.200E-03	1.195E-02	2	Notch, NOTCH1 precursor
223	cardioblast differentiation	16	1.20	1.19	1.20	1.19	2	Notch, NOTCH1 precursor

			0E-03	5E-02	0E-03	5E-02		
224	coagulation	663	1.300E-03	1.284E-02	1.300E-03	1.284E-02	8	HBG, HBGA, ERK1 (MAPK3), CD43, 14-3-3 zeta/delta, Adult hemoglobin, ERK1/2, 14-3-3
225	blood coagulation	663	1.300E-03	1.284E-02	1.300E-03	1.284E-02	8	HBG, HBGA, ERK1 (MAPK3), CD43, 14-3-3 zeta/delta, Adult hemoglobin, ERK1/2, 14-3-3
226	positive regulation of cellular metabolic process	2852	1.328E-03	1.299E-02	1.328E-03	1.299E-02	19	Ajuba, Cyclin B1, LHX1, IRT-1, Gpbp1, TEF-5, TIRAP (Mal), PTD015, Notch, ERK1 (MAPK3), SRB7, NOTCH1 precursor, CD43, OBFC1, Cyclin B, Adult hemoglobin, ERK1/2, DEAF, 14-3-3
227	skin morphogenesis	67	1.335E-03	1.299E-02	1.335E-03	1.299E-02	3	Tissue kallikreins, Notch, NOTCH1 precursor
228	regulation of developmental process	2404	1.337E	1.299E	1.337E	1.299E	17	Tissue kallikreins, ZNF261, LST1, Cyclin B1, LHX1, Rab-11A, PTD015, Notch, SRB7, NOTCH1 precursor, Cyclin B, Kallikrein 3 (PSA), Adult hemoglobin, ZHX3, ERK1/2, DEAF, 14-3-3

			-03	-02	-03	-02		
229	response to stimulus	9680	1.341E-03	1.299E-02	1.341E-03	1.299E-02	44	Ajuba, Tissue kallikreins, IGHG4, Kallikrein 1, LST1, Cyclin B1, TIPIN, AL1A1, LHX1, HBG, IRT-1, HBGA, Rab-11A, TEF-5, TIRAP (Mal), Olfactory receptor, Notch, Rab-3B, IGHG1, ERK1 (MAPK3), CALCOCO2, NOTCH1 precursor, SULT1E1, CD43, 14-3-3 zeta/delta, NFKBIL1, CKS2, MTL5, OR10G3, PLEKHG2, Annexin XI, G18, Spinophilin, Cyclin B, Rab-3, Kallikrein 3 (PSA), Adult hemoglobin, Protrudin, RIG-G, ERK1/2, UGDH, 14-3-3, CapZIP, RBBP7 (RbAp46)
230	distal tubule development	17	1.357E-03	1.299E-02	1.357E-03	1.299E-02	2	Notch, NOTCH1 precursor
231	regulation of neurotransmitter uptake	17	1.357E-03	1.299E-02	1.357E-03	1.299E-02	2	Rab-3B, Rab-3
232	collecting duct development	17	1.357E-03	1.299E-02	1.357E-03	1.299E-02	2	Notch, NOTCH1 precursor
233	hemostasis	670	1.390E-0	1.325E-0	1.390E-0	1.325E-0	8	HBG, HBGA, ERK1 (MAPK3), CD43, 14-3-3 zeta/delta, Adult hemoglobin, ERK1/2, 14-3-3

			3	2	3	2		
234	glomerulus development	69	1.453E-03	1.349E-02	1.453E-03	1.349E-02	3	LHX1, Notch, NOTCH1 precursor
235	cell cycle	1580	1.458E-03	1.349E-02	1.458E-03	1.349E-02	13	Ajuba, Tissue kallikreins, Cyclin B1, TIPIN, Rab-11A, Notch, ERK1 (MAPK3), CKS2, Annexin XI, Spinophilin, Cyclin B, ERK1/2, 14-3-3
236	regulation of RNA metabolic process	4075	1.462E-03	1.349E-02	1.462E-03	1.349E-02	24	Ajuba, Cyclin B1, LHX1, HBG, HBGA, Gpbp1, TEF-5, TIRAP (Mal), Olfactory receptor, PTD015, Notch, ERK1 (MAPK3), DR1, SRB7, NOTCH1 precursor, NFKBIL1, AO7, Cyclin B, Adult hemoglobin, ZHX3, ERK1/2, DEAF, 14-3-3, RBBP7 (RbAp46)
237	regulation of biological quality	3588	1.487E-03	1.349E-02	1.487E-03	1.349E-02	22	Tissue kallikreins, LST1, AL1A1, HBG, HBGA, Rab-11A, TIRAP (Mal), Thioredoxin-like 2, Rab-3B, ERK1 (MAPK3), SULT1E1, CD43, 14-3-3 zeta/delta, NFKBIL1, MTL5, OBFC1, Cyclin B, Rab-3, Kallikrein 3 (PSA), Adult hemoglobin, ERK1/2, 14-3-3
238	positive regulation of multicellular organismal process	843	1.506E-03	1.349E-02	1.506E-03	1.349E-02	9	Tissue kallikreins, Kallikrein 1, Cyclin B1, LHX1, IRT-1, TIRAP (Mal), Notch, NOTCH1 precursor, Cyclin B

239	protein complex assembly	1200	1.514E-03	1.349E-02	1.514E-03	1.349E-02	11	Cyclin B1, AL1A1, HBG, IRT-1, HBGA, ERK1 (MAPK3), Cyclin B, Adult hemoglobin, ERK1/2, 14-3-3, RBBP7 (RbAp46)
240	regulation of cell proliferation involved in heart morphogenesis	18	1.524E-03	1.349E-02	1.524E-03	1.349E-02	2	Notch, NOTCH1 precursor
241	positive regulation of astrocyte differentiation	18	1.524E-03	1.349E-02	1.524E-03	1.349E-02	2	Notch, NOTCH1 precursor
242	negative regulation of myotube differentiation	18	1.524E-03	1.349E-02	1.524E-03	1.349E-02	2	Notch, NOTCH1 precursor
243	mitotic metaphase plate congression	18	1.524E-03	1.349E-02	1.524E-03	1.349E-02	2	Cyclin B1, Cyclin B
24	negative regulation of myoblast	18	1.	1.	1.	1.	2	Notch, NOTCH1 precursor

4	differentiation		524E-03	349E-02	524E-03	349E-02		
245	peptidyl-cysteine modification	18	1.524E-03	1.349E-02	1.524E-03	1.349E-02	2	Rab-3B, Rab-3
246	histamine transport	18	1.524E-03	1.349E-02	1.524E-03	1.349E-02	2	14-3-3 zeta/delta, 14-3-3
247	venous blood vessel development	18	1.524E-03	1.349E-02	1.524E-03	1.349E-02	2	Notch, NOTCH1 precursor
248	left/right axis specification	18	1.524E-03	1.349E-02	1.524E-03	1.349E-02	2	Notch, NOTCH1 precursor
249	anagen	18	1.52	1.34	1.52	1.34	2	Notch, NOTCH1 precursor

			4E-03	9E-02	4E-03	9E-02		
250	ventricular cardiac muscle cell development	18	1.524E-03	1.349E-02	1.524E-03	1.349E-02	2	Cyclin B1, Cyclin B
251	negative regulation of transcription, DNA-templated	1201	1.524E-03	1.349E-02	1.524E-03	1.349E-02	11	Ajuba, LHX1, HBG, HBGA, Notch, DR1, NOTCH1 precursor, Adult hemoglobin, ZHX3, 14-3-3, RBBP7 (RbAp46)
252	protein complex biogenesis	1203	1.545E-03	1.361E-02	1.545E-03	1.361E-02	11	Cyclin B1, AL1A1, HBG, IRT-1, HBGA, ERK1 (MAPK3), Cyclin B, Adult hemoglobin, ERK1/2, 14-3-3, RBBP7 (RbAp46)
253	negative regulation of nucleobase-containing compound metabolic process	1398	1.591E-03	1.392E-02	1.591E-03	1.392E-02	12	Ajuba, TIPIN, LHX1, HBG, HBGA, Notch, DR1, NOTCH1 precursor, Adult hemoglobin, ZHX3, 14-3-3, RBBP7 (RbAp46)
254	protein complex subunit organization	1596	1.597E	1.392E	1.597E	1.392E	13	Cyclin B1, AL1A1, HBG, IRT-1, HBGA, ERK1 (MAPK3), CKS2, Spinophilin, Cyclin B, Adult hemoglobin, ERK1/2, 14-3-3, RBBP7 (RbAp46)

			-03	-02	-03	-02		
255	cell morphogenesis involved in neuron differentiation	685	1.599E-03	1.392E-02	1.599E-03	1.392E-02	8	LHX1, Olfactory receptor, Notch, ERK1 (MAPK3), NOTCH1 precursor, Rab-3, ERK1/2, 14-3-3
256	regulation of molecular function	3133	1.621E-03	1.407E-02	1.621E-03	1.407E-02	20	Ajuba, Tissue kallikreins, Cyclin B1, AL1A1, TIRAP (Mal), Notch, PAP41, ERK1 (MAPK3), NOTCH1 precursor, NFKBIL1, AO7, CKS2, PLEKHG2, G18, Spinophilin, Cyclin B, Rab-3, Kallikrein 3 (PSA), ERK1/2, 14-3-3
257	regulation of immune system process	1602	1.652E-03	1.428E-02	1.652E-03	1.428E-02	13	IGHG4, LST1, IRT-1, TIRAP (Mal), Notch, IGHG1, ERK1 (MAPK3), NOTCH1 precursor, CD43, NFKBIL1, Adult hemoglobin, ERK1/2, 14-3-3
258	regulation of striated muscle tissue development	157	1.677E-03	1.440E-02	1.677E-03	1.440E-02	4	Cyclin B1, Notch, NOTCH1 precursor, Cyclin B
259	vasculogenesis involved in coronary vascular morphogenesis	19	1.699E-0	1.440E-0	1.699E-0	1.440E-0	2	Notch, NOTCH1 precursor

			3	2	3	2		
260	keratinocyte proliferation	19	1.699E-03	1.440E-02	1.699E-03	1.440E-02	2	Tissue kallikreins, 14-3-3
261	regulation of endothelial cell differentiation	19	1.699E-03	1.440E-02	1.699E-03	1.440E-02	2	Notch, NOTCH1 precursor
262	endocardial cushion morphogenesis	19	1.699E-03	1.440E-02	1.699E-03	1.440E-02	2	Notch, NOTCH1 precursor
263	positive regulation of organelle organization	394	1.708E-03	1.442E-02	1.708E-03	1.442E-02	6	Cyclin B1, ERK1 (MAPK3), 14-3-3 zeta/delta, Cyclin B, ERK1/2, 14-3-3
264	tissue morphogenesis	693	1.720E-03	1.447E-02	1.720E-03	1.447E-02	8	Ajuba, Tissue kallikreins, AL1A1, LHX1, Notch, NOTCH1 precursor, Kallikrein 3 (PSA), DEAF

265	cellular component organization	5419	1.730E-03	1.450E-02	1.730E-03	1.450E-02	29	Ajuba, Tissue kallikreins, ZNF261, LST1, Cyclin B1, TIPIN, AL1A1, LHX1, HBG, IRT-1, HBGA, Rab-11A, Olfactory receptor, Notch, Rab-3B, ERK1 (MAPK3), DR1, NOTCH1 precursor, 14-3-3 zeta/delta, CKS2, OBFC1, Spinophilin, Cyclin B, Rab-3, Adult hemoglobin, Protrudin, ERK1/2, 14-3-3, RBBP7 (RbAp46)
266	regulation of muscle organ development	159	1.757E-03	1.461E-02	1.757E-03	1.461E-02	4	Cyclin B1, Notch, NOTCH1 precursor, Cyclin B
267	regulation of muscle tissue development	159	1.757E-03	1.461E-02	1.757E-03	1.461E-02	4	Cyclin B1, Notch, NOTCH1 precursor, Cyclin B
268	negative regulation of RNA biosynthetic process	1226	1.796E-03	1.484E-02	1.796E-03	1.484E-02	11	Ajuba, LHX1, HBG, HBGA, Notch, DR1, NOTCH1 precursor, Adult hemoglobin, ZHX3, 14-3-3, RBBP7 (RbAp46)
269	cardiac ventricle development	160	1.797E-03	1.484E-02	1.797E-03	1.484E-02	4	Tissue kallikreins, Notch, NOTCH1 precursor, Kallikrein 3 (PSA)
27	cardiac muscle cell differentiation	16	1.	1.	1.	1.	4	Cyclin B1, Notch, NOTCH1 precursor, Cyclin B

0		1	839E-03	493E-02	839E-03	493E-02		
271	regulation of exocytosis	161	1.839E-03	1.493E-02	1.839E-03	1.493E-02	4	Notch, Rab-3B, NOTCH1 precursor, Rab-3
272	chordate embryonic development	868	1.841E-03	1.493E-02	1.841E-03	1.493E-02	9	Cyclin B1, LHX1, Notch, SRB7, NOTCH1 precursor, Cyclin B, Adult hemoglobin, ERK1/2, DEAF
273	regulation of epidermis development	75	1.847E-03	1.493E-02	1.847E-03	1.493E-02	3	Notch, NOTCH1 precursor, 14-3-3
274	cell differentiation in spinal cord	75	1.847E-03	1.493E-02	1.847E-03	1.493E-02	3	LHX1, Notch, NOTCH1 precursor
275	axon development	701	1.84	1.49	1.84	1.49	8	LHX1, Olfactory receptor, Notch, ERK1 (MAPK3), NOTCH1 precursor, Rab-3, ERK1/2, 14-3-3

			8E-03	3E-02	8E-03	3E-02		
276	cardiac vascular smooth muscle cell differentiation	20	1.884E-03	1.505E-02	1.884E-03	1.505E-02	2	Notch, NOTCH1 precursor
277	regulation of cell migration involved in sprouting angiogenesis	20	1.884E-03	1.505E-02	1.884E-03	1.505E-02	2	Notch, NOTCH1 precursor
278	response to muramyl dipeptide	20	1.884E-03	1.505E-02	1.884E-03	1.505E-02	2	Notch, NOTCH1 precursor
279	regulation of cellular localization	1234	1.891E-03	1.505E-02	1.891E-03	1.505E-02	11	Cyclin B1, Notch, Rab-3B, ERK1 (MAPK3), NOTCH1 precursor, 14-3-3 zeta/delta, NFKBIL1, Cyclin B, Rab-3, ERK1/2, 14-3-3
280	regulation of cellular biosynthetic process	4661	1.903E	1.510E	1.903E	1.510E	26	Ajuba, TIPIN, LHX1, HBG, IRT-1, HBGA, Gpbp1, TEF-5, TIRAP (Mal), Olfactory receptor, PTD015, Notch, ERK1 (MAPK3), DR1, SRB7, NOTCH1 precursor, CD43, NFKBIL1, AO7, OBFC1, Adult hemoglobin, ZHX3, ERK1/2, DEAF, 14-3-3, RBBP7 (RbAp46)

			-03	-02	-03	-02		
281	striated muscle tissue development	403	1.913E-03	1.512E-02	1.913E-03	1.512E-02	6	Tissue kallikreins, Cyclin B1, Notch, NOTCH1 precursor, Cyclin B, Kallikrein 3 (PSA)
282	negative regulation of macromolecule biosynthetic process	1430	1.926E-03	1.512E-02	1.926E-03	1.512E-02	12	Ajuba, TIPIN, LHX1, HBG, HBGA, Notch, DR1, NOTCH1 precursor, Adult hemoglobin, ZHX3, 14-3-3, RBBP7 (RbAp46)
283	regeneration	274	1.928E-03	1.512E-02	1.928E-03	1.512E-02	5	Tissue kallikreins, Cyclin B1, Notch, NOTCH1 precursor, Cyclin B
284	regulation of anatomical structure size	404	1.937E-03	1.512E-02	1.937E-03	1.512E-02	6	Tissue kallikreins, Rab-11A, Rab-3B, Rab-3, Kallikrein 3 (PSA), Adult hemoglobin
285	positive regulation of protein phosphorylation	875	1.945E-0	1.512E-0	1.945E-0	1.512E-0	9	Ajuba, Cyclin B1, IRT-1, TIRAP (Mal), ERK1 (MAPK3), CD43, Cyclin B, ERK1/2, 14-3-3

			3	2	3	2		
286	cellular response to chemical stimulus	2945	1.947E-03	1.512E-02	1.947E-03	1.512E-02	19	Cyclin B1, AL1A1, LHX1, IRT-1, TIRAP (Mal), Notch, ERK1 (MAPK3), NOTCH1 precursor, SULT1E1, NFKBIL1, PLEKHG2, Spinophilin, Cyclin B, Adult hemoglobin, Protrudin, RIG-G, ERK1/2, UGDH, 14-3-3
287	regulation of chemotaxis	164	1.967E-03	1.522E-02	1.967E-03	1.522E-02	4	IRT-1, TIRAP (Mal), Notch, NOTCH1 precursor
288	embryo development ending in birth or egg hatching	877	1.975E-03	1.523E-02	1.975E-03	1.523E-02	9	Cyclin B1, LHX1, Notch, SRB7, NOTCH1 precursor, Cyclin B, Adult hemoglobin, ERK1/2, DEAF
289	cell projection morphogenesis	879	2.006E-03	1.540E-02	2.006E-03	1.540E-02	9	Tissue kallikreins, LHX1, Olfactory receptor, Notch, ERK1 (MAPK3), NOTCH1 precursor, Rab-3, ERK1/2, 14-3-3
290	cellular response to hypoxia	165	2.011E-03	1.540E-02	2.011E-03	1.540E-02	4	Cyclin B1, Notch, NOTCH1 precursor, Cyclin B

291	protein heterooligomerization	166	2.055E-03	1.569E-02	2.055E-03	1.569E-02	4	HBG, HBGA, Adult hemoglobin, 14-3-3
292	regulation of glial cell proliferation	21	2.078E-03	1.570E-02	2.078E-03	1.570E-02	2	Notch, NOTCH1 precursor
293	secretory columnal luminar epithelial cell differentiation involved in prostate glandular acinus development	21	2.078E-03	1.570E-02	2.078E-03	1.570E-02	2	Notch, NOTCH1 precursor
294	positive regulation of mRNA 3'-end processing	21	2.078E-03	1.570E-02	2.078E-03	1.570E-02	2	Cyclin B1, Cyclin B
295	cellular response to decreased oxygen levels	167	2.101E-03	1.582E-02	2.101E-03	1.582E-02	4	Cyclin B1, Notch, NOTCH1 precursor, Cyclin B
29	negative regulation of nitrogen	14	2.	1.	2.	1.	12	Ajuba, TIPIN, LHX1, HBG, HBGA, Notch, DR1, NOTCH1 precursor, Adult hemoglobin, ZHX3, 14-3-3, RBBP7

6	compound metabolic process	46	1.15E-03	5.87E-02	1.15E-03	5.87E-02		(RbAp46)
297	activation of MAPKK activity	80	2.221E-03	1.661E-02	2.221E-03	1.661E-02	3	ERK1 (MAPK3), ERK1/2, 14-3-3
298	cardiac chamber morphogenesis	170	2.241E-03	1.668E-02	2.241E-03	1.668E-02	4	Tissue kallikreins, Notch, NOTCH1 precursor, Kallikrein 3 (PSA)
299	regulation of macromolecule biosynthetic process	4455	2.246E-03	1.668E-02	2.246E-03	1.668E-02	25	Ajuba, TIPIN, LHX1, HBG, HBGA, Gpbp1, TEF-5, TIRAP (Mal), Olfactory receptor, PTD015, Notch, ERK1 (MAPK3), DR1, SRB7, NOTCH1 precursor, CD43, NFKBIL1, AO7, OBFC1, Adult hemoglobin, ZHX3, ERK1/2, DEAF, 14-3-3, RBBP7 (RbAp46)
300	developmental maturation	285	2.288E-03	1.694E-02	2.288E-03	1.694E-02	5	Cyclin B1, Notch, NOTCH1 precursor, Cyclin B, Rab-3
301	negative regulation of RNA metabolic process	1267	2.32	1.71	2.32	1.71	11	Ajuba, LHX1, HBG, HBGA, Notch, DR1, NOTCH1 precursor, Adult hemoglobin, ZHX3, 14-3-3, RBBP7 (RbAp46)

			6E-03	7E-02	6E-03	7E-02		
302	regulation of organelle organization	901	2.372E-03	1.744E-02	2.372E-03	1.744E-02	9	Cyclin B1, Olfactory receptor, ERK1 (MAPK3), 14-3-3 zeta/delta, Spinophilin, Cyclin B, Rab-3, ERK1/2, 14-3-3
303	protein localization	1878	2.385E-03	1.748E-02	2.385E-03	1.748E-02	14	Ajuba, Rab-11A, SNX1, Olfactory receptor, Notch, Rab-3B, ERK1 (MAPK3), NOTCH1 precursor, 14-3-3 zeta/delta, NFKBIL1, Rab-3, Protrudin, ERK1/2, 14-3-3
304	regulation of biosynthetic process	4739	2.438E-03	1.782E-02	2.438E-03	1.782E-02	26	Ajuba, TIPIN, LHX1, HBG, IRT-1, HBGA, Gpbp1, TEF-5, TIRAP (Mal), Olfactory receptor, PTD015, Notch, ERK1 (MAPK3), DR1, SRB7, NOTCH1 precursor, CD43, NFKBIL1, AO7, OBFC1, Adult hemoglobin, ZHX3, ERK1/2, DEAF, 14-3-3, RBBP7 (RbAp46)
305	azole transport	23	2.493E-03	1.802E-02	2.493E-03	1.802E-02	2	14-3-3 zeta/delta, 14-3-3
306	mesenchyme morphogenesis	23	2.493E	1.802E	2.493E	1.802E	2	Notch, NOTCH1 precursor

			-03	-02	-03	-02		
307	muscle tissue development	425	2.496E-03	1.802E-02	2.496E-03	1.802E-02	6	Tissue kallikreins, Cyclin B1, Notch, NOTCH1 precursor, Cyclin B, Kallikrein 3 (PSA)
308	cell part morphogenesis	908	2.498E-03	1.802E-02	2.498E-03	1.802E-02	9	Tissue kallikreins, LHX1, Olfactory receptor, Notch, ERK1 (MAPK3), NOTCH1 precursor, Rab-3, ERK1/2, 14-3-3
309	negative regulation of macromolecule metabolic process	2103	2.510E-03	1.804E-02	2.510E-03	1.804E-02	15	Ajuba, Cyclin B1, TIPIN, LHX1, HBG, IRT-1, HBGA, Notch, DR1, NOTCH1 precursor, Cyclin B, Adult hemoglobin, ZHX3, 14-3-3, RBBP7 (RbAp46)
310	cellular response to stress	1685	2.587E-03	1.853E-02	2.587E-03	1.853E-02	13	Cyclin B1, TIPIN, IRT-1, TIRAP (Mal), Notch, ERK1 (MAPK3), NOTCH1 precursor, Cyclin B, Adult hemoglobin, ERK1/2, 14-3-3, CapZIP, RBBP7 (RbAp46)
311	cellular component organization or biogenesis	5557	2.611E-0	1.862E-0	2.611E-0	1.862E-0	29	Ajuba, Tissue kallikreins, ZNF261, LST1, Cyclin B1, TIPIN, AL1A1, LHX1, HBG, IRT-1, HBGA, Rab-11A, Olfactory receptor, Notch, Rab-3B, ERK1 (MAPK3), DR1, NOTCH1 precursor, 14-3-3 zeta/delta, CKS2, OBFC1, Spinophilin, Cyclin B, Rab-3, Adult hemoglobin, Protrudin, ERK1/2, 14-3-3, RBBP7 (RbAp46)

			3	2	3	2		
312	in utero embryonic development	579	2.615E-03	1.862E-02	2.615E-03	1.862E-02	7	Cyclin B1, Notch, SRB7, NOTCH1 precursor, Cyclin B, Adult hemoglobin, ERK1/2
313	response to external stimulus	2559	2.635E-03	1.865E-02	2.635E-03	1.865E-02	17	Tissue kallikreins, Kallikrein 1, Cyclin B1, LHX1, IRT-1, TIRAP (Mal), Olfactory receptor, Notch, ERK1 (MAPK3), NOTCH1 precursor, CD43, NFKBIL1, Spinophilin, Cyclin B, RIG-G, ERK1/2, 14-3-3
314	kidney epithelium development	85	2.640E-03	1.865E-02	2.640E-03	1.865E-02	3	LHX1, Notch, NOTCH1 precursor
315	response to chemical	5293	2.645E-03	1.865E-02	2.645E-03	1.865E-02	28	Tissue kallikreins, Kallikrein 1, Cyclin B1, AL1A1, LHX1, IRT-1, TIRAP (Mal), Olfactory receptor, Notch, ERK1 (MAPK3), CALCOCO2, NOTCH1 precursor, SULT1E1, CD43, 14-3-3 zeta/delta, NFKBIL1, MTL5, OR10G3, PLEKHG2, Annexin XI, Spinophilin, Cyclin B, Adult hemoglobin, Protrudin, RIG-G, ERK1/2, UGDH, 14-3-3
316	regulation of transferase activity	1099	2.665E-03	1.873E-02	2.665E-03	1.873E-02	10	Ajuba, Tissue kallikreins, Cyclin B1, TIRAP (Mal), ERK1 (MAPK3), CKS2, Cyclin B, Kallikrein 3 (PSA), ERK1/2, 14-3-3

317	cellular response to follicle-stimulating hormone stimulus	24	2.714E-03	1.878E-02	2.714E-03	1.878E-02	2	Notch, NOTCH1 precursor
318	apoptotic process involved in morphogenesis	24	2.714E-03	1.878E-02	2.714E-03	1.878E-02	2	Notch, NOTCH1 precursor
319	cardiac right ventricle morphogenesis	24	2.714E-03	1.878E-02	2.714E-03	1.878E-02	2	Notch, NOTCH1 precursor
320	cellular response to ammonium ion	24	2.714E-03	1.878E-02	2.714E-03	1.878E-02	2	IRT-1, Spinophilin
321	regulation of mRNA 3'-end processing	24	2.714E-03	1.878E-02	2.714E-03	1.878E-02	2	Cyclin B1, Cyclin B
322	endoderm development	86	2.	1.	2.	1.	3	LHX1, Notch, NOTCH1 precursor

2			729E-03	880E-02	729E-03	880E-02		
323	DNA-templated transcription, initiation	297	2.734E-03	1.880E-02	2.734E-03	1.880E-02	5	TEF-5, Notch, ERK1 (MAPK3), NOTCH1 precursor, ERK1/2
324	negative regulation of cellular biosynthetic process	1494	2.774E-03	1.901E-02	2.774E-03	1.901E-02	12	Ajuba, TIPIN, LHX1, HBG, HBGA, Notch, DR1, NOTCH1 precursor, Adult hemoglobin, ZHX3, 14-3-3, RBBP7 (RbAp46)
325	regulation of chromosome organization	181	2.811E-03	1.921E-02	2.811E-03	1.921E-02	4	Cyclin B1, ERK1 (MAPK3), Cyclin B, ERK1/2
326	cellular response to oxygen levels	182	2.867E-03	1.952E-02	2.867E-03	1.952E-02	4	Cyclin B1, Notch, NOTCH1 precursor, Cyclin B
327	cellular response to growth factor stimulus	753	2.87	1.95	2.87	1.95	8	LHX1, Notch, ERK1 (MAPK3), NOTCH1 precursor, PLEKHG2, Protrudin, ERK1/2, 14-3-3

			8E-03	2E-02	8E-03	2E-02		
328	regulation of vesicle-mediated transport	439	2.930E-03	1.952E-02	2.930E-03	1.952E-02	6	Notch, Rab-3B, ERK1 (MAPK3), NOTCH1 precursor, Rab-3, ERK1/2
329	metaphase plate congression	25	2.944E-03	1.952E-02	2.944E-03	1.952E-02	2	Cyclin B1, Cyclin B
330	regulation of blood volume by renin-angiotensin	25	2.944E-03	1.952E-02	2.944E-03	1.952E-02	2	Tissue kallikreins, Kallikrein 3 (PSA)
331	regulation of synaptic transmission, dopaminergic	25	2.944E-03	1.952E-02	2.944E-03	1.952E-02	2	Rab-3B, Rab-3
332	cell surface receptor signaling pathway involved in heart development	25	2.944E	1.952E	2.944E	1.952E	2	Notch, NOTCH1 precursor

			-03	-02	-03	-02		
333	response to insecticide	25	2.944E-03	1.952E-02	2.944E-03	1.952E-02	2	Cyclin B1, Cyclin B
334	positive regulation of histone acetylation	25	2.944E-03	1.952E-02	2.944E-03	1.952E-02	2	ERK1 (MAPK3), ERK1/2
335	cardiac epithelial to mesenchymal transition	25	2.944E-03	1.952E-02	2.944E-03	1.952E-02	2	Notch, NOTCH1 precursor
336	cell morphogenesis	1115	2.957E-03	1.955E-02	2.957E-03	1.955E-02	10	Tissue kallikreins, LST1, LHX1, Olfactory receptor, Notch, ERK1 (MAPK3), NOTCH1 precursor, Rab-3, ERK1/2, 14-3-3
337	regulation of cellular macromolecule biosynthetic process	4292	3.028E-0	1.995E-0	3.028E-0	1.995E-0	24	Ajuba, TIPIN, LHX1, HBG, HBGA, Gpbp1, TEF-5, TIRAP (Mal), Olfactory receptor, PTD015, Notch, ERK1 (MAPK3), DR1, SRB7, NOTCH1 precursor, NFKBIL1, AO7, OBFC1, Adult hemoglobin, ZHX3, ERK1/2, DEAF, 14-3-3, RBBP7 (RbAp46)

			3	2	3	2		
338	regulation of macromolecule metabolic process	6163	3.084E-03	2.026E-02	3.084E-03	2.026E-02	31	Ajuba, Cyclin B1, TIPIN, LHX1, HBG, IRT-1, HBGA, Gpbp1, SNX1, TEF-5, TIRAP (Mal), Olfactory receptor, PTD015, Notch, ERK1 (MAPK3), DR1, SRB7, NOTCH1 precursor, CD43, NFKBIL1, AO7, CKS2, OBFC1, Spinophilin, Cyclin B, Adult hemoglobin, ZHX3, ERK1/2, DEAF, 14-3-3, RBBP7 (RbAp46)
339	regulation of multicellular organismal process	2833	3.138E-03	2.034E-02	3.138E-03	2.034E-02	18	Tissue kallikreins, Kallikrein 1, Cyclin B1, LHX1, IRT-1, Rab-11A, TIRAP (Mal), Notch, Thioredoxin-like 2, NOTCH1 precursor, CD43, NFKBIL1, Cyclin B, Kallikrein 3 (PSA), Adult hemoglobin, ZHX3, DEAF, 14-3-3
340	regulation of heart morphogenesis	26	3.183E-03	2.034E-02	3.183E-03	2.034E-02	2	Notch, NOTCH1 precursor
341	glomerulus vasculature development	26	3.183E-03	2.034E-02	3.183E-03	2.034E-02	2	Notch, NOTCH1 precursor
342	TIRAP-dependent toll-like receptor signaling pathway	1	3.233E-03	2.034E-02	3.233E-03	2.034E-02	1	TIRAP (Mal)

343	oviduct epithelium development	1	3.233E-03	2.034E-02	3.233E-03	2.034E-02	1	LHX1
344	TIRAP-dependent toll-like receptor 4 signaling pathway	1	3.233E-03	2.034E-02	3.233E-03	2.034E-02	1	TIRAP (Mal)
345	regulation of interleukin-15 production	1	3.233E-03	2.034E-02	3.233E-03	2.034E-02	1	TIRAP (Mal)
346	positive regulation of chemokine (C-X-C motif) ligand 1 production	1	3.233E-03	2.034E-02	3.233E-03	2.034E-02	1	TIRAP (Mal)
347	uterine epithelium development	1	3.233E-03	2.034E-02	3.233E-03	2.034E-02	1	LHX1
34	positive regulation of muscle hyperplasia	1	3.	2.	3.	2.	1	IRT-1

8			233E-03	034E-02	233E-03	034E-02		
349	regulation of chemokine (C-X-C motif) ligand 1 production	1	3.233E-03	2.034E-02	3.233E-03	2.034E-02	1	TIRAP (Mal)
350	horizontal cell localization	1	3.233E-03	2.034E-02	3.233E-03	2.034E-02	1	LHX1
351	nephric duct elongation	1	3.233E-03	2.034E-02	3.233E-03	2.034E-02	1	LHX1
352	positive regulation of interleukin-15 production	1	3.233E-03	2.034E-02	3.233E-03	2.034E-02	1	TIRAP (Mal)
353	thymocyte aggregation	1	3.23	2.03	3.23	2.03	1	CD43

			3E-03	4E-02	3E-03	4E-02		
354	cell division	772	3.350E-03	2.093E-02	3.350E-03	2.093E-02	8	Cyclin B1, TIPIN, Rab-11A, Notch, NOTCH1 precursor, CKS2, Annexin XI, Cyclin B
355	regulation of neural precursor cell proliferation	93	3.405E-03	2.093E-02	3.405E-03	2.093E-02	3	LHX1, Notch, NOTCH1 precursor
356	cardiocyte differentiation	191	3.409E-03	2.093E-02	3.409E-03	2.093E-02	4	Cyclin B1, Notch, NOTCH1 precursor, Cyclin B
357	mast cell activation involved in immune response	27	3.430E-03	2.093E-02	3.430E-03	2.093E-02	2	14-3-3 zeta/delta, 14-3-3
358	kidney vasculature development	27	3.430E	2.093E	3.430E	2.093E	2	Notch, NOTCH1 precursor

			-03	-02	-03	-02		
359	positive regulation of peptidyl-lysine acetylation	27	3.430E-03	2.093E-02	3.430E-03	2.093E-02	2	ERK1 (MAPK3), ERK1/2
360	mast cell degranulation	27	3.430E-03	2.093E-02	3.430E-03	2.093E-02	2	14-3-3 zeta/delta, 14-3-3
361	epithelial cell fate commitment	27	3.430E-03	2.093E-02	3.430E-03	2.093E-02	2	Notch, NOTCH1 precursor
362	renal system vasculature development	27	3.430E-03	2.093E-02	3.430E-03	2.093E-02	2	Notch, NOTCH1 precursor
363	positive regulation of neurotransmitter transport	27	3.430E-0	2.093E-0	3.430E-0	2.093E-0	2	Rab-3B, Rab-3

			3	2	3	2		
364	[hair follicle maturation]	27	3.430E-03	2.093E-02	3.430E-03	2.093E-02	2	Notch, NOTCH1 precursor
365	[cellular response to stimulus]	7628	3.446E-03	2.096E-02	3.446E-03	2.096E-02	36	Tissue kallikreins, IGHG4, Kallikrein 1, Cyclin B1, TIPIN, AL1A1, LHX1, IRT-1, Rab-11A, TEF-5, TIRAP (Mal), Olfactory receptor, Notch, Rab-3B, IGHG1, ERK1 (MAPK3), NOTCH1 precursor, SULT1E1, CD43, 14-3-3 zeta/delta, NFKBIL1, CKS2, OR10G3, PLEKHG2, Spinophilin, Cyclin B, Rab-3, Kallikrein 3 (PSA), Adult hemoglobin, Protrudin, RIG-G, ERK1/2, UGDH, 14-3-3, CapZIP, RBBP7 (RbAp46)
366	[reproduction]	1742	3.454E-03	2.096E-02	3.454E-03	2.096E-02	13	Tissue kallikreins, Cyclin B1, AL1A1, LHX1, TEF-5, Olfactory receptor, Notch, NOTCH1 precursor, SULT1E1, CKS2, MTL5, Cyclin B, DEAF
367	[localization]	5388	3.487E-03	2.111E-02	3.487E-03	2.111E-02	28	Ajuba, IGHG4, Cyclin B1, LHX1, HBG, IRT-1, HBGA, Rab-11A, SNX1, TIP47, Olfactory receptor, Notch, Rab-3B, IGHG1, ERK1 (MAPK3), NOTCH1 precursor, CD43, 14-3-3 zeta/delta, NFKBIL1, OATP-C, Annexin XI, Spinophilin, Cyclin B, Rab-3, Adult hemoglobin, Protrudin, ERK1/2, 14-3-3
368	[toll-like receptor 5 signaling pathway]	94	3.510E-03	2.117E-02	3.510E-03	2.117E-02	3	TIRAP (Mal), ERK1 (MAPK3), ERK1/2

369	negative regulation of biosynthetic process	1538	3.517E-03	2.117E-02	3.517E-03	2.117E-02	12	Ajuba, TIPIN, LHX1, HBG, HBGA, Notch, DR1, NOTCH1 precursor, Adult hemoglobin, ZHX3, 14-3-3, RBBP7 (RbAp46)
370	cardiac chamber development	194	3.603E-03	2.157E-02	3.603E-03	2.157E-02	4	Tissue kallikreins, Notch, NOTCH1 precursor, Kallikrein 3 (PSA)
371	muscle cell development	194	3.603E-03	2.157E-02	3.603E-03	2.157E-02	4	Cyclin B1, Notch, NOTCH1 precursor, Cyclin B
372	immune effector process	614	3.617E-03	2.160E-02	3.617E-03	2.160E-02	7	IGHG4, IGHG1, ERK1 (MAPK3), 14-3-3 zeta/delta, RIG-G, ERK1/2, 14-3-3
373	regulation of anatomical structure morphogenesis	962	3.670E-03	2.166E-02	3.670E-03	2.166E-02	9	Tissue kallikreins, ZNF261, LST1, LHX1, Rab-11A, Notch, NOTCH1 precursor, Kallikrein 3 (PSA), 14-3-3
37	regulation of cell cycle process	61	3.	2.	3.	2.	7	Cyclin B1, TIPIN, IRT-1, Olfactory receptor, Spinophilin, Cyclin B, 14-3-3

4		6	682E-03	166E-02	682E-03	166E-02		
375	production of molecular mediator involved in inflammatory response	28	3.686E-03	2.166E-02	3.686E-03	2.166E-02	2	14-3-3 zeta/delta, 14-3-3
376	apoptotic process involved in development	28	3.686E-03	2.166E-02	3.686E-03	2.166E-02	2	Notch, NOTCH1 precursor
377	response to stilbenoid	28	3.686E-03	2.166E-02	3.686E-03	2.166E-02	2	Adult hemoglobin, RIG-G
378	positive regulation of protein acetylation	28	3.686E-03	2.166E-02	3.686E-03	2.166E-02	2	ERK1 (MAPK3), ERK1/2
379	response to abiotic stimulus	1551	3.76	2.20	3.76	2.20	12	Ajuba, Cyclin B1, TIPIN, IRT-1, Notch, ERK1 (MAPK3), NOTCH1 precursor, Cyclin B, Rab-3, ERK1/2, CapZIP, RBBP7 (RbAp46)

			6E-03	7E-02	6E-03	7E-02		
380	Notch receptor processing	29	3.951E-03	2.274E-02	3.951E-03	2.274E-02	2	Notch, NOTCH1 precursor
381	hippo signaling	29	3.951E-03	2.274E-02	3.951E-03	2.274E-02	2	TEF-5, 14-3-3
382	positive regulation of neuroblast proliferation	29	3.951E-03	2.274E-02	3.951E-03	2.274E-02	2	Notch, NOTCH1 precursor
383	negative regulation of exocytosis	29	3.951E-03	2.274E-02	3.951E-03	2.274E-02	2	Notch, NOTCH1 precursor
384	negative regulation of microtubule depolymerization	29	3.951E	2.274E	3.951E	2.274E	2	Cyclin B1, Cyclin B

			-03	-02	-03	-02		
385	mast cell mediated immunity	29	3.951E-03	2.274E-02	3.951E-03	2.274E-02	2	14-3-3 zeta/delta, 14-3-3
386	oocyte maturation	29	3.951E-03	2.274E-02	3.951E-03	2.274E-02	2	Cyclin B1, Cyclin B
387	Fc receptor signaling pathway	324	3.963E-03	2.274E-02	3.963E-03	2.274E-02	5	IGHG4, IGHG1, ERK1 (MAPK3), ERK1/2, 14-3-3
388	positive regulation of developmental process	1163	3.995E-03	2.287E-02	3.995E-03	2.287E-02	10	Cyclin B1, LHX1, Rab-11A, PTD015, Notch, NOTCH1 precursor, Cyclin B, Adult hemoglobin, ZHX3, 14-3-3
389	G2/M transition of mitotic cell cycle	200	4.016E-0	2.287E-0	4.016E-0	2.287E-0	4	Ajuba, Cyclin B1, Cyclin B, 14-3-3

			3	2	3	2		
390	cell cycle G2/M phase transition	200	4.016E-03	2.287E-02	4.016E-03	2.287E-02	4	Ajuba, Cyclin B1, Cyclin B, 14-3-3
391	toll-like receptor TLR6:TLR2 signaling pathway	99	4.060E-03	2.295E-02	4.060E-03	2.295E-02	3	TIRAP (Mal), ERK1 (MAPK3), ERK1/2
392	toll-like receptor TLR1:TLR2 signaling pathway	99	4.060E-03	2.295E-02	4.060E-03	2.295E-02	3	TIRAP (Mal), ERK1 (MAPK3), ERK1/2
393	response to growth factor	797	4.062E-03	2.295E-02	4.062E-03	2.295E-02	8	LHX1, Notch, ERK1 (MAPK3), NOTCH1 precursor, PLEKHG2, Protrudin, ERK1/2, 14-3-3
394	response to organic cyclic compound	1367	4.171E-03	2.323E-02	4.171E-03	2.323E-02	11	Cyclin B1, AL1A1, IRT-1, Notch, ERK1 (MAPK3), NOTCH1 precursor, Spinophilin, Cyclin B, Adult hemoglobin, RIG-G, ERK1/2

395	embryo development	1369	4.217E-03	2.323E-02	4.217E-03	2.323E-02	11	Cyclin B1, AL1A1, LHX1, Notch, SRB7, NOTCH1 precursor, Cyclin B, Adult hemoglobin, ERK1/2, UGDH, DEAF
396	negative regulation of striated muscle cell differentiation	30	4.225E-03	2.323E-02	4.225E-03	2.323E-02	2	Notch, NOTCH1 precursor
397	positive regulation of mRNA processing	30	4.225E-03	2.323E-02	4.225E-03	2.323E-02	2	Cyclin B1, Cyclin B
398	JAK-STAT cascade involved in growth hormone signaling pathway	30	4.225E-03	2.323E-02	4.225E-03	2.323E-02	2	ERK1 (MAPK3), ERK1/2
399	heart valve morphogenesis	30	4.225E-03	2.323E-02	4.225E-03	2.323E-02	2	Notch, NOTCH1 precursor
40	response to follicle-stimulating hormone	30	4.	2.	4.	2.	2	Notch, NOTCH1 precursor

0			225E-03	323E-02	225E-03	323E-02		
401	vascular smooth muscle cell differentiation	30	4.225E-03	2.323E-02	4.225E-03	2.323E-02	2	Notch, NOTCH1 precursor
402	left/right pattern formation	30	4.225E-03	2.323E-02	4.225E-03	2.323E-02	2	Notch, NOTCH1 precursor
403	establishment of chromosome localization	30	4.225E-03	2.323E-02	4.225E-03	2.323E-02	2	Cyclin B1, Cyclin B
404	histone phosphorylation	30	4.225E-03	2.323E-02	4.225E-03	2.323E-02	2	Cyclin B1, Cyclin B
405	positive regulation of kinase activity	632	4.23	2.32	4.23	2.32	7	Ajuba, Tissue kallikreins, TIRAP (Mal), ERK1 (MAPK3), Kallikrein 3 (PSA), ERK1/2, 14-3-3

			5E-03	3E-02	5E-03	3E-02		
406	toll-like receptor 2 signaling pathway	101	4.294E-03	2.343E-02	4.294E-03	2.343E-02	3	TIRAP (Mal), ERK1 (MAPK3), ERK1/2
407	cellular response to interleukin-1	101	4.294E-03	2.343E-02	4.294E-03	2.343E-02	3	TIRAP (Mal), ERK1 (MAPK3), ERK1/2
408	positive regulation of cell death	638	4.458E-03	2.417E-02	4.458E-03	2.417E-02	7	Tissue kallikreins, Kallikrein 1, AL1A1, Notch, NOTCH1 precursor, PLEKHG2, Adult hemoglobin
409	regulation of protein modification process	1584	4.461E-03	2.417E-02	4.461E-03	2.417E-02	12	Ajuba, Cyclin B1, IRT-1, TIRAP (Mal), ERK1 (MAPK3), CD43, CKS2, Spinophilin, Cyclin B, Adult hemoglobin, ERK1/2, 14-3-3
410	generation of neurons	1795	4.463E	2.417E	4.463E	2.417E	13	Tissue kallikreins, LST1, LHX1, Rab-11A, Olfactory receptor, Notch, ERK1 (MAPK3), NOTCH1 precursor, Spinophilin, Rab-3, Protrudin, ERK1/2, 14-3-3

			-03	-02	-03	-02		
411	protein transport	1380	4.479E-03	2.420E-02	4.479E-03	2.420E-02	11	Rab-11A, SNX1, Olfactory receptor, Notch, Rab-3B, ERK1 (MAPK3), NOTCH1 precursor, 14-3-3 zeta/delta, Rab-3, ERK1/2, 14-3-3
412	negative regulation of anoikis	31	4.507E-03	2.429E-02	4.507E-03	2.429E-02	2	Notch, NOTCH1 precursor
413	regulation of hormone levels	335	4.562E-03	2.450E-02	4.562E-03	2.450E-02	5	Tissue kallikreins, AL1A1, SULT1E1, 14-3-3 zeta/delta, 14-3-3
414	regulation of cell morphogenesis	481	4.577E-03	2.450E-02	4.577E-03	2.450E-02	6	ZNF261, LST1, Rab-11A, Notch, NOTCH1 precursor, 14-3-3
415	response to lipopolysaccharide	481	4.577E-0	2.450E-0	4.577E-0	2.450E-0	6	TIRAP (Mal), Notch, ERK1 (MAPK3), NOTCH1 precursor, NFKBIL1, ERK1/2

			3	2	3	2		
416	regulation of response to external stimulus	816	4.677E-03	2.497E-02	4.677E-03	2.497E-02	8	Tissue kallikreins, Kallikrein 1, IRT-1, TIRAP (Mal), Notch, NOTCH1 precursor, CD43, NFKBIL1
417	regulation of cellular response to stress	485	4.764E-03	2.519E-02	4.764E-03	2.519E-02	6	Ajuba, Tissue kallikreins, TIRAP (Mal), ERK1 (MAPK3), Adult hemoglobin, ERK1/2
418	prostate glandular acinus development	32	4.797E-03	2.519E-02	4.797E-03	2.519E-02	2	Notch, NOTCH1 precursor
419	regulation of hematopoietic progenitor cell differentiation	32	4.797E-03	2.519E-02	4.797E-03	2.519E-02	2	Notch, NOTCH1 precursor
420	endocardial cushion development	32	4.797E-03	2.519E-02	4.797E-03	2.519E-02	2	Notch, NOTCH1 precursor

421	negative regulation of glial cell differentiation	32	4.797E-03	2.519E-02	4.797E-03	2.519E-02	2	Notch, NOTCH1 precursor
422	heart valve development	32	4.797E-03	2.519E-02	4.797E-03	2.519E-02	2	Notch, NOTCH1 precursor
423	heart trabecula morphogenesis	32	4.797E-03	2.519E-02	4.797E-03	2.519E-02	2	Notch, NOTCH1 precursor
424	neurotrophin TRK receptor signaling pathway	341	4.915E-03	2.575E-02	4.915E-03	2.575E-02	5	ERK1 (MAPK3), PLEKHG2, Protrudin, ERK1/2, 14-3-3
425	regulation of transcription, DNA-templated	3938	4.938E-03	2.580E-02	4.938E-03	2.580E-02	22	Ajuba, LHX1, HBG, HBGA, Gpbp1, TEF-5, TIRAP (Mal), Olfactory receptor, PTD015, Notch, ERK1 (MAPK3), DR1, SRB7, NOTCH1 precursor, NFKBIL1, AO7, Adult hemoglobin, ZHX3, ERK1/2, DEAF, 14-3-3, RBBP7 (RbAp46)
42	macromolecular complex subunit organization	18	5.	2.	5.	2.	13	Cyclin B1, AL1A1, HBG, IRT-1, HBGA, ERK1 (MAPK3), CKS2, Spinophilin, Cyclin B, Adult hemoglobin, ERK1/2,

6		23	085E-03	645E-02	085E-03	645E-02		14-3-3, RBBP7 (RbAp46)
427	cytoplasmic sequestering of protein	33	5.096E-03	2.645E-02	5.096E-03	2.645E-02	2	NFKBIL1, 14-3-3
428	chromosome localization	33	5.096E-03	2.645E-02	5.096E-03	2.645E-02	2	Cyclin B1, Cyclin B
429	multi-organism process	2964	5.115E-03	2.648E-02	5.115E-03	2.648E-02	18	Tissue kallikreins, Cyclin B1, TEF-5, TIRAP (Mal), Olfactory receptor, Notch, ERK1 (MAPK3), CALCOCO2, NOTCH1 precursor, SULT1E1, CD43, NFKBIL1, MTL5, Cyclin B, RIG-G, ERK1/2, DEAF, 14-3-3
430	positive regulation of phosphorylation	1014	5.172E-03	2.671E-02	5.172E-03	2.671E-02	9	Ajuba, Cyclin B1, IRT-1, TIRAP (Mal), ERK1 (MAPK3), CD43, Cyclin B, ERK1/2, 14-3-3
431	neurotrophin signaling pathway	346	5.22	2.69	5.22	2.69	5	ERK1 (MAPK3), PLEKHG2, Protrudin, ERK1/2, 14-3-3

			4E-03	2E-02	4E-03	2E-02		
432	reproductive process	1617	5.256E-03	2.702E-02	5.256E-03	2.702E-02	12	Tissue kallikreins, Cyclin B1, AL1A1, LHX1, TEF-5, Olfactory receptor, Notch, NOTCH1 precursor, SULT1E1, MTL5, Cyclin B, DEAF
433	cellular component morphogenesis	1211	5.304E-03	2.718E-02	5.304E-03	2.718E-02	10	Tissue kallikreins, LST1, LHX1, Olfactory receptor, Notch, ERK1 (MAPK3), NOTCH1 precursor, Rab-3, ERK1/2, 14-3-3
434	regulation of acute inflammatory response	109	5.311E-03	2.718E-02	5.311E-03	2.718E-02	3	Tissue kallikreins, Kallikrein 1, CD43
435	cell morphogenesis involved in differentiation	834	5.324E-03	2.718E-02	5.324E-03	2.718E-02	8	LHX1, Olfactory receptor, Notch, ERK1 (MAPK3), NOTCH1 precursor, Rab-3, ERK1/2, 14-3-3
436	macromolecular complex assembly	1415	5.401E	2.727E	5.401E	2.727E	11	Cyclin B1, AL1A1, HBG, IRT-1, HBGA, ERK1 (MAPK3), Cyclin B, Adult hemoglobin, ERK1/2, 14-3-3, RBBP7 (RbAp46)

			-03	-02	-03	-02		
437	regulation of microtubule depolymerization	34	5.403E-03	2.727E-02	5.403E-03	2.727E-02	2	Cyclin B1, Cyclin B
438	positive regulation of BMP signaling pathway	34	5.403E-03	2.727E-02	5.403E-03	2.727E-02	2	Notch, NOTCH1 precursor
439	regulation of keratinocyte differentiation	34	5.403E-03	2.727E-02	5.403E-03	2.727E-02	2	Notch, NOTCH1 precursor
440	neurological system process involved in regulation of systemic arterial blood pressure	34	5.403E-03	2.727E-02	5.403E-03	2.727E-02	2	Tissue kallikreins, Kallikrein 3 (PSA)
441	cellular protein localization	1217	5.489E-0	2.764E-0	5.489E-0	2.764E-0	10	Ajuba, Rab-11A, SNX1, Olfactory receptor, ERK1 (MAPK3), 14-3-3 zeta/delta, NFKBIL1, Protrudin, ERK1/2, 14-3-3

			3	2	3	2		
442	cell differentiation	3974	5.524E-03	2.776E-02	5.524E-03	2.776E-02	22	Tissue kallikreins, LST1, Cyclin B1, LHX1, Rab-11A, TIRAP (Mal), Olfactory receptor, Notch, ERK1 (MAPK3), SRB7, NOTCH1 precursor, CD43, MTL5, Spinophilin, Cyclin B, Rab-3, Adult hemoglobin, Protrudin, ZHX3, ERK1/2, DEAF, 14-3-3
443	regulation of mitotic cell cycle	501	5.566E-03	2.791E-02	5.566E-03	2.791E-02	6	Cyclin B1, TIPIN, IRT-1, Spinophilin, Cyclin B, 14-3-3
444	regulation of tumor necrosis factor production	111	5.586E-03	2.794E-02	5.586E-03	2.794E-02	3	TIRAP (Mal), CD43, NFKBIL1
445	regulation of RNA biosynthetic process	3980	5.628E-03	2.809E-02	5.628E-03	2.809E-02	22	Ajuba, LHX1, HBG, HBGA, Gpbp1, TEF-5, TIRAP (Mal), Olfactory receptor, PTD015, Notch, ERK1 (MAPK3), DR1, SRB7, NOTCH1 precursor, NFKBIL1, AO7, Adult hemoglobin, ZHX3, ERK1/2, DEAF, 14-3-3, RBBP7 (RbAp46)
446	cellular macromolecule localization	1223	5.679E-03	2.815E-02	5.679E-03	2.815E-02	10	Ajuba, Rab-11A, SNX1, Olfactory receptor, ERK1 (MAPK3), 14-3-3 zeta/delta, NFKBIL1, Protrudin, ERK1/2, 14-3-3

447	growth hormone receptor signaling pathway	35	5.719E-03	2.815E-02	5.719E-03	2.815E-02	2	ERK1 (MAPK3), ERK1/2
448	regulation of anoikis	35	5.719E-03	2.815E-02	5.719E-03	2.815E-02	2	Notch, NOTCH1 precursor
449	cardiac atrium morphogenesis	35	5.719E-03	2.815E-02	5.719E-03	2.815E-02	2	Notch, NOTCH1 precursor
450	epithelial cell differentiation involved in prostate gland development	35	5.719E-03	2.815E-02	5.719E-03	2.815E-02	2	Notch, NOTCH1 precursor
451	MyD88-dependent toll-like receptor signaling pathway	112	5.726E-03	2.815E-02	5.726E-03	2.815E-02	3	TIRAP (Mal), ERK1 (MAPK3), ERK1/2
45	negative regulation of metabolic process	22	5.	2.	5.	2.	15	Ajuba, Cyclin B1, TIPIN, LHX1, HBG, IRT-1, HBGA, Notch, DR1, NOTCH1 precursor, Cyclin B, Adult hemoglobin,

2		94	728E-03	815E-02	728E-03	815E-02		ZHX3, 14-3-3, RBBP7 (RbAp46)
453	regulation of body fluid levels	847	5.832E-03	2.859E-02	5.832E-03	2.859E-02	8	HBG, HBGA, ERK1 (MAPK3), CD43, 14-3-3 zeta/delta, Adult hemoglobin, ERK1/2, 14-3-3
454	response to molecule of bacterial origin	508	5.947E-03	2.909E-02	5.947E-03	2.909E-02	6	TIRAP (Mal), Notch, ERK1 (MAPK3), NOTCH1 precursor, NFKBIL1, ERK1/2
455	biological regulation	12947	6.019E-03	2.928E-02	6.019E-03	2.928E-02	52	Ajuba, Tissue kallikreins, IGHG4, ZNF261, Kallikrein 1, LST1, Cyclin B1, TIPIN, AL1A1, LHX1, HBG, IRT-1, HBGA, Gpbp1, Rab-11A, SNX1, TEF-5, TIRAP (Mal), Olfactory receptor, PTD015, Notch, Thioredoxin-like 2, Rab-3B, PAP41, IGHG1, ERK1 (MAPK3), DR1, SRB7, NOTCH1 precursor, SULT1E1, CD43, 14-3-3 zeta/delta, NFKBIL1, AO7, CKS2, MTL5, OR10G3, OBFC1, PLEKHG2, G18, Spinophilin, Cyclin B, Rab-3, Kallikrein 3 (PSA), Adult hemoglobin, Protrudin, RIG-G, ZHX3, ERK1/2, DEAF, 14-3-3, RBBP7 (RbAp46)
456	developmental programmed cell death	36	6.043E-03	2.928E-02	6.043E-03	2.928E-02	2	Notch, NOTCH1 precursor
457	positive regulation of epithelial to mesenchymal transition	36	6.0	2.9	6.0	2.9	2	Notch, NOTCH1 precursor

			43E-03	28E-02	43E-03	28E-02		
458	cellular response to vascular endothelial growth factor stimulus	36	6.043E-03	2.928E-02	6.043E-03	2.928E-02	2	Notch, NOTCH1 precursor
459	negative regulation of cellular metabolic process	2082	6.051E-03	2.928E-02	6.051E-03	2.928E-02	14	Ajuba, Cyclin B1, TIPIN, LHX1, HBG, HBGA, Notch, DR1, NOTCH1 precursor, Cyclin B, Adult hemoglobin, ZHX3, 14-3-3, RBBP7 (RbAp46)
460	cell cycle process	1237	6.142E-03	2.966E-02	6.142E-03	2.966E-02	10	Ajuba, Cyclin B1, TIPIN, Rab-11A, Notch, CKS2, Annexin XI, Spinophilin, Cyclin B, 14-3-3
461	positive regulation of defense response	362	6.304E-03	3.015E-02	6.304E-03	3.015E-02	5	Tissue kallikreins, Kallikrein 1, TIRAP (Mal), ERK1 (MAPK3), ERK1/2
462	nephron development	116	6.310	3.015	6.310	3.015	3	LHX1, Notch, NOTCH1 precursor

			E-03	E-02	E-03	E-02		
463	epithelial cell proliferation	116	6.310E-03	3.015E-02	6.310E-03	3.015E-02	3	Tissue kallikreins, ERK1/2, 14-3-3
464	peptide biosynthetic process	37	6.375E-03	3.015E-02	6.375E-03	3.015E-02	2	Tissue kallikreins, Kallikrein 3 (PSA)
465	epithelial cell differentiation involved in kidney development	37	6.375E-03	3.015E-02	6.375E-03	3.015E-02	2	Notch, NOTCH1 precursor
466	positive regulation of phosphatidylinositol 3-kinase activity	37	6.375E-03	3.015E-02	6.375E-03	3.015E-02	2	Tissue kallikreins, Kallikrein 3 (PSA)
467	positive regulation of transcription, DNA-templated	1448	6.402E-	3.015E-	6.402E-	3.015E-	11	LHX1, Gpbp1, TEF-5, PTD015, Notch, ERK1 (MAPK3), SRB7, NOTCH1 precursor, ERK1/2, DEAF, 14-3-3

			03	02	03	02		
468	negative regulation of peptidyl-serine dephosphorylation	2	6.456E-03	3.015E-02	6.456E-03	3.015E-02	1	14-3-3
469	regulation of anterior head development	2	6.456E-03	3.015E-02	6.456E-03	3.015E-02	1	LHX1
470	negative regulation of smooth muscle cell chemotaxis	2	6.456E-03	3.015E-02	6.456E-03	3.015E-02	1	IRT-1
471	T cell aggregation	2	6.456E-03	3.015E-02	6.456E-03	3.015E-02	1	CD43
472	plasma membrane to endosome transport	2	6.456E-03	3.015E-02	6.456E-03	3.015E-02	1	Rab-11A

473	seminal clot liquefaction	2	6.456E-03	3.015E-02	6.456E-03	3.015E-02	1	Tissue kallikreins
474	positive regulation of anterior head development	2	6.456E-03	3.015E-02	6.456E-03	3.015E-02	1	LHX1
475	maintenance of presynaptic active zone structure	2	6.456E-03	3.015E-02	6.456E-03	3.015E-02	1	Rab-3
476	axis specification	117	6.461E-03	3.015E-02	6.461E-03	3.015E-02	3	LHX1, Notch, NOTCH1 precursor
477	innate immune response	1051	6.506E-03	3.029E-02	6.506E-03	3.029E-02	9	IGHG4, IRT-1, TIRAP (Mal), IGHG1, ERK1 (MAPK3), CALCOCO2, RIG-G, ERK1/2, 14-3-3
47	tissue development	18	6.	3.	6.	3.	13	Ajuba, Tissue kallikreins, Cyclin B1, AL1A1, LHX1, Notch, ERK1 (MAPK3), NOTCH1 precursor, Cyclin B, Kallikrein 3

8		79	542E-03	040E-02	542E-03	040E-02		(PSA), ERK1/2, DEAF, 14-3-3
479	cytokine secretion	38	6.715E-03	3.088E-02	6.715E-03	3.088E-02	2	Notch, NOTCH1 precursor
480	positive regulation of lipid kinase activity	38	6.715E-03	3.088E-02	6.715E-03	3.088E-02	2	Tissue kallikreins, Kallikrein 3 (PSA)
481	transcription initiation from RNA polymerase I promoter	38	6.715E-03	3.088E-02	6.715E-03	3.088E-02	2	ERK1 (MAPK3), ERK1/2
482	regulation of astrocyte differentiation	38	6.715E-03	3.088E-02	6.715E-03	3.088E-02	2	Notch, NOTCH1 precursor
483	cardiac muscle cell proliferation	38	6.71	3.08	6.71	3.08	2	Notch, NOTCH1 precursor

			5E-03	8E-02	5E-03	8E-02		
484	cellular response to fibroblast growth factor stimulus	232	6.762E-03	3.103E-02	6.762E-03	3.103E-02	4	LHX1, ERK1 (MAPK3), ERK1/2, 14-3-3
485	establishment of protein localization	1461	6.835E-03	3.130E-02	6.835E-03	3.130E-02	11	Rab-11A, SNX1, Olfactory receptor, Notch, Rab-3B, ERK1 (MAPK3), NOTCH1 precursor, 14-3-3 zeta/delta, Rab-3, ERK1/2, 14-3-3
486	neurogenesis	1891	6.894E-03	3.151E-02	6.894E-03	3.151E-02	13	Tissue kallikreins, LST1, LHX1, Rab-11A, Olfactory receptor, Notch, ERK1 (MAPK3), NOTCH1 precursor, Spinophilin, Rab-3, Protrudin, ERK1/2, 14-3-3
487	regulation of cell cycle	1062	6.950E-03	3.170E-02	6.950E-03	3.170E-02	9	Cyclin B1, TIPIN, IRT-1, Olfactory receptor, Notch, CKS2, Spinophilin, Cyclin B, 14-3-3
488	prostate gland epithelium morphogenesis	39	7.063E	3.189E	7.063E	3.189E	2	Notch, NOTCH1 precursor

			-03	-02	-03	-02		
489	regulation of chromosome segregation	39	7.063E-03	3.189E-02	7.063E-03	3.189E-02	2	Cyclin B1, Cyclin B
490	striated muscle cell proliferation	39	7.063E-03	3.189E-02	7.063E-03	3.189E-02	2	Notch, NOTCH1 precursor
491	cardiac atrium development	39	7.063E-03	3.189E-02	7.063E-03	3.189E-02	2	Notch, NOTCH1 precursor
492	negative regulation of microtubule polymerization or depolymerization	39	7.063E-03	3.189E-02	7.063E-03	3.189E-02	2	Cyclin B1, Cyclin B
493	striated muscle contraction	121	7.088E-0	3.193E-0	7.088E-0	3.193E-0	3	Tissue kallikreins, Kallikrein 3 (PSA), CapZIP

			3	2	3	2		
494	response to fibroblast growth factor	236	7.175E-03	3.226E-02	7.175E-03	3.226E-02	4	LHX1, ERK1 (MAPK3), ERK1/2, 14-3-3
495	positive regulation of cell differentiation	878	7.195E-03	3.226E-02	7.195E-03	3.226E-02	8	LHX1, Rab-11A, PTD015, Notch, NOTCH1 precursor, Adult hemoglobin, ZHX3, 14-3-3
496	morphogenesis of an epithelium	529	7.203E-03	3.226E-02	7.203E-03	3.226E-02	6	Ajuba, AL1A1, LHX1, Notch, NOTCH1 precursor, DEAF
497	regulation of organ formation	40	7.420E-03	3.296E-02	7.420E-03	3.296E-02	2	Notch, NOTCH1 precursor
498	cellular response to growth hormone stimulus	40	7.420E-03	3.296E-02	7.420E-03	3.296E-02	2	ERK1 (MAPK3), ERK1/2

499	somatic stem cell division	40	7.420E-03	3.296E-02	7.420E-03	3.296E-02	2	Notch, NOTCH1 precursor
500	cellular response to gonadotropin stimulus	40	7.420E-03	3.296E-02	7.420E-03	3.296E-02	2	Notch, NOTCH1 precursor
501	regulation of cellular process	11707	7.687E-03	3.403E-02	7.687E-03	3.403E-02	48	Ajuba, Tissue kallikreins, IGHG4, ZNF261, Kallikrein 1, LST1, Cyclin B1, TIPIN, AL1A1, LHX1, HBG, IRT-1, HBGA, Gpbp1, Rab-11A, TEF-5, TIRAP (Mal), Olfactory receptor, PTD015, Notch, Thioredoxin-like 2, Rab-3B, IGHG1, ERK1 (MAPK3), DR1, SRB7, NOTCH1 precursor, CD43, 14-3-3 zeta/delta, NFKBIL1, AO7, CKS2, OR10G3, OBFC1, PLEKHG2, G18, Spinophilin, Cyclin B, Rab-3, Kallikrein 3 (PSA), Adult hemoglobin, Protrudin, RIG-G, ZHX3, ERK1/2, DEAF, 14-3-3, RBBP7 (RbAp46)
502	positive regulation of protein insertion into mitochondrial membrane involved in apoptotic signaling pathway	41	7.784E-03	3.403E-02	7.784E-03	3.403E-02	2	14-3-3 zeta/delta, 14-3-3
503	negative regulation of blood vessel endothelial cell migration	41	7.784E-03	3.403E-02	7.784E-03	3.403E-02	2	Notch, NOTCH1 precursor
50	response to phenylpropanoid	41	7.	3.	7.	3.	2	Adult hemoglobin, RIG-G

4			784E-03	403E-02	784E-03	403E-02		
505	negative regulation of gliogenesis	41	7.784E-03	3.403E-02	7.784E-03	3.403E-02	2	Notch, NOTCH1 precursor
506	regulation of protein insertion into mitochondrial membrane involved in apoptotic signaling pathway	41	7.784E-03	3.403E-02	7.784E-03	3.403E-02	2	14-3-3 zeta/delta, 14-3-3
507	lipopolysaccharide-mediated signaling pathway	41	7.784E-03	3.403E-02	7.784E-03	3.403E-02	2	ERK1 (MAPK3), ERK1/2
508	prostate gland morphogenesis	41	7.784E-03	3.403E-02	7.784E-03	3.403E-02	2	Notch, NOTCH1 precursor
509	cellular localization	2374	7.83	3.41	7.83	3.41	15	Ajuba, Cyclin B1, Rab-11A, SNX1, Olfactory receptor, Notch, ERK1 (MAPK3), NOTCH1 precursor, 14-3-3 zeta/delta, NFKBIL1, Cyclin B, Rab-3, Protrudin, ERK1/2, 14-3-3

			2E-03	7E-02	2E-03	7E-02		
510	cellular response to molecule of bacterial origin	243	7.936E-03	3.456E-02	7.936E-03	3.456E-02	4	TIRAP (Mal), ERK1 (MAPK3), NFKBIL1, ERK1/2
511	regulation of biological process	12391	7.964E-03	3.462E-02	7.964E-03	3.462E-02	50	Ajuba, Tissue kallikreins, IGHG4, ZNF261, Kallikrein 1, LST1, Cyclin B1, TIPIN, AL1A1, LHX1, HBG, IRT-1, HBGA, Gpbp1, Rab-11A, SNX1, TEF-5, TIRAP (Mal), Olfactory receptor, PTD015, Notch, Thioredoxin-like 2, Rab-3B, PAP41, IGHG1, ERK1 (MAPK3), DR1, SRB7, NOTCH1 precursor, CD43, 14-3-3 zeta/delta, NFKBIL1, AO7, CKS2, OR10G3, OBFC1, PLEKHG2, G18, Spinophilin, Cyclin B, Rab-3, Kallikrein 3 (PSA), Adult hemoglobin, Protrudin, RIG-G, ZHX3, ERK1/2, DEAF, 14-3-3, RBBP7 (RbAp46)
512	positive regulation of catalytic activity	1493	8.000E-03	3.470E-02	8.000E-03	3.470E-02	11	Ajuba, Tissue kallikreins, Cyclin B1, AL1A1, TIRAP (Mal), ERK1 (MAPK3), Cyclin B, Rab-3, Kallikrein 3 (PSA), ERK1/2, 14-3-3
513	regulation of protein kinase activity	896	8.090E-03	3.473E-02	8.090E-03	3.473E-02	8	Ajuba, Cyclin B1, TIRAP (Mal), ERK1 (MAPK3), CKS2, Cyclin B, ERK1/2, 14-3-3
514	peptide metabolic process	127	8.094E	3.473E	8.094E	3.473E	3	Tissue kallikreins, Kallikrein 3 (PSA), Adult hemoglobin

			-03	-02	-03	-02		
515	regulation of neuroblast proliferation	42	8.156E-03	3.473E-02	8.156E-03	3.473E-02	2	Notch, NOTCH1 precursor
516	mast cell activation	42	8.156E-03	3.473E-02	8.156E-03	3.473E-02	2	14-3-3 zeta/delta, 14-3-3
517	response to epidermal growth factor	42	8.156E-03	3.473E-02	8.156E-03	3.473E-02	2	ERK1 (MAPK3), ERK1/2
518	auditory receptor cell differentiation	42	8.156E-03	3.473E-02	8.156E-03	3.473E-02	2	Notch, NOTCH1 precursor
519	leukocyte degranulation	42	8.156E-0	3.473E-0	8.156E-0	3.473E-0	2	14-3-3 zeta/delta, 14-3-3

			3	2	3	2		
520	mitotic nuclear envelope disassembly	42	8.156E-03	3.473E-02	8.156E-03	3.473E-02	2	Cyclin B1, Cyclin B
521	gastrulation with mouth forming second	42	8.156E-03	3.473E-02	8.156E-03	3.473E-02	2	LHX1, UGDH
522	regulation of behavior	245	8.163E-03	3.473E-02	8.163E-03	3.473E-02	4	IRT-1, TIRAP (Mal), Notch, NOTCH1 precursor
523	positive regulation of RNA biosynthetic process	1498	8.196E-03	3.480E-02	8.196E-03	3.480E-02	11	LHX1, Gpbp1, TEF-5, PTD015, Notch, ERK1 (MAPK3), SRB7, NOTCH1 precursor, ERK1/2, DEAF, 14-3-3
524	negative regulation of neurogenesis	129	8.446E-03	3.577E-02	8.446E-03	3.577E-02	3	Notch, NOTCH1 precursor, 14-3-3

525	multi-organism reproductive process	1297	8.477E-03	3.577E-02	8.477E-03	3.577E-02	10	Tissue kallikreins, Cyclin B1, TEF-5, Olfactory receptor, Notch, NOTCH1 precursor, SULT1E1, MTL5, Cyclin B, DEAF
526	regulation of systemic arterial blood pressure by renin-angiotensin	43	8.536E-03	3.577E-02	8.536E-03	3.577E-02	2	Tissue kallikreins, Kallikrein 3 (PSA)
527	muscle cell proliferation	43	8.536E-03	3.577E-02	8.536E-03	3.577E-02	2	Notch, NOTCH1 precursor
528	myoblast differentiation	43	8.536E-03	3.577E-02	8.536E-03	3.577E-02	2	Notch, NOTCH1 precursor
529	ventricular septum morphogenesis	43	8.536E-03	3.577E-02	8.536E-03	3.577E-02	2	Notch, NOTCH1 precursor
53	regulation of myoblast differentiation	43	8.	3.	8.	3.	2	Notch, NOTCH1 precursor

0			536E-03	577E-02	536E-03	577E-02		
531	regulation of blood vessel size	130	8.626E-03	3.603E-02	8.626E-03	3.603E-02	3	Tissue kallikreins, Kallikrein 3 (PSA), Adult hemoglobin
532	humoral immune response	249	8.630E-03	3.603E-02	8.630E-03	3.603E-02	4	IGHG4, Notch, IGHG1, NOTCH1 precursor
533	cellular developmental process	4128	8.741E-03	3.642E-02	8.741E-03	3.642E-02	22	Tissue kallikreins, LST1, Cyclin B1, LHX1, Rab-11A, TIRAP (Mal), Olfactory receptor, Notch, ERK1 (MAPK3), SRB7, NOTCH1 precursor, CD43, MTL5, Spinophilin, Cyclin B, Rab-3, Adult hemoglobin, Protrudin, ZHX3, ERK1/2, DEAF, 14-3-3
534	cellular response to organic substance	2404	8.766E-03	3.646E-02	8.766E-03	3.646E-02	15	Cyclin B1, LHX1, IRT-1, TIRAP (Mal), Notch, ERK1 (MAPK3), NOTCH1 precursor, NFKBIL1, PLEKHG2, Spinophilin, Cyclin B, Protrudin, RIG-G, ERK1/2, 14-3-3
535	camera-type eye morphogenesis	131	8.80	3.65	8.80	3.65	3	AL1A1, LHX1, Notch

			8E-03	7E-02	8E-03	7E-02		
536	regulation of response to stimulus	3878	8.919E-03	3.671E-02	8.919E-03	3.671E-02	21	Ajuba, Tissue kallikreins, IGHG4, Kallikrein 1, AL1A1, IRT-1, TIRAP (Mal), Notch, Thioredoxin-like 2, IGHG1, ERK1 (MAPK3), NOTCH1 precursor, CD43, 14-3-3 zeta/delta, NFKBIL1, PLEKHG2, G18, Spinophilin, Adult hemoglobin, ERK1/2, 14-3-3
537	hair follicle morphogenesis	44	8.924E-03	3.671E-02	8.924E-03	3.671E-02	2	Notch, NOTCH1 precursor
538	response to iron ion	44	8.924E-03	3.671E-02	8.924E-03	3.671E-02	2	Cyclin B1, Cyclin B
539	nuclear envelope disassembly	44	8.924E-03	3.671E-02	8.924E-03	3.671E-02	2	Cyclin B1, Cyclin B
540	membrane disassembly	44	8.924E	3.671E	8.924E	3.671E	2	Cyclin B1, Cyclin B

			-03	-02	-03	-02		
541	response to lipid	1308	8.971E-03	3.678E-02	8.971E-03	3.678E-02	10	Cyclin B1, AL1A1, IRT-1, TIRAP (Mal), Notch, ERK1 (MAPK3), NOTCH1 precursor, NFKBIL1, Cyclin B, ERK1/2
542	regulation of tube size	132	8.993E-03	3.678E-02	8.993E-03	3.678E-02	3	Tissue kallikreins, Kallikrein 3 (PSA), Adult hemoglobin
543	toll-like receptor 4 signaling pathway	132	8.993E-03	3.678E-02	8.993E-03	3.678E-02	3	TIRAP (Mal), ERK1 (MAPK3), ERK1/2
544	positive regulation of protein modification process	1109	9.117E-03	3.722E-02	9.117E-03	3.722E-02	9	Ajuba, Cyclin B1, IRT-1, TIRAP (Mal), ERK1 (MAPK3), CD43, Cyclin B, ERK1/2, 14-3-3
545	pericardium development	45	9.320E-0	3.791E-0	9.320E-0	3.791E-0	2	Notch, NOTCH1 precursor

			3	2	3	2		
546	nephron tubule development	45	9.320E-03	3.791E-02	9.320E-03	3.791E-02	2	Notch, NOTCH1 precursor
547	stem cell maintenance	134	9.368E-03	3.797E-02	9.368E-03	3.797E-02	3	Notch, SRB7, NOTCH1 precursor
548	keratinocyte differentiation	134	9.368E-03	3.797E-02	9.368E-03	3.797E-02	3	Notch, NOTCH1 precursor, 14-3-3
549	cytokinesis, completion of separation	3	9.668E-03	3.802E-02	9.668E-03	3.802E-02	1	Annexin XI
550	positive regulation of epithelial cell differentiation involved in kidney development	3	9.668E-03	3.802E-02	9.668E-03	3.802E-02	1	LHX1

551	UDP-glucuronate biosynthetic process	3	9.668E-03	3.802E-02	9.668E-03	3.802E-02	1	UGDH
552	regulation of heart rate by hormone	3	9.668E-03	3.802E-02	9.668E-03	3.802E-02	1	14-3-3
553	positive regulation of toll-like receptor 2 signaling pathway	3	9.668E-03	3.802E-02	9.668E-03	3.802E-02	1	TIRAP (Mal)
554	glucocorticoid catabolic process	3	9.668E-03	3.802E-02	9.668E-03	3.802E-02	1	14-3-3
555	positive regulation of nephron tubule epithelial cell differentiation	3	9.668E-03	3.802E-02	9.668E-03	3.802E-02	1	LHX1
55	regulation of peptidyl-serine dephosphorylatio	3	9.	3.	9.	3.	1	14-3-3

6	n		668E-03	802E-02	668E-03	802E-02		
557	positive regulation of muscle adaptation	3	9.668E-03	3.802E-02	9.668E-03	3.802E-02	1	IRT-1
558	cervix development	3	9.668E-03	3.802E-02	9.668E-03	3.802E-02	1	LHX1
559	lateral motor column neuron migration	3	9.668E-03	3.802E-02	9.668E-03	3.802E-02	1	LHX1
560	cytosine metabolic process	3	9.668E-03	3.802E-02	9.668E-03	3.802E-02	1	ERK1/2
561	lymphocyte aggregation	3	9.66	3.80	9.66	3.80	1	CD43

			8E-03	2E-02	8E-03	2E-02		
562	regulation of muscle hyperplasia	3	9.668E-03	3.802E-02	9.668E-03	3.802E-02	1	IRT-1
563	negative regulation of hippo signaling	3	9.668E-03	3.802E-02	9.668E-03	3.802E-02	1	Ajuba
564	transcription from RNA polymerase I promoter	46	9.724E-03	3.802E-02	9.724E-03	3.802E-02	2	ERK1 (MAPK3), ERK1/2
565	ventricular cardiac muscle cell differentiation	46	9.724E-03	3.802E-02	9.724E-03	3.802E-02	2	Cyclin B1, Cyclin B
566	regulation of oligodendrocyte differentiation	46	9.724E	3.802E	9.724E	3.802E	2	Notch, NOTCH1 precursor

			-03	-02	-03	-02		
567	embryonic hindlimb morphogenesis	46	9.724E-03	3.802E-02	9.724E-03	3.802E-02	2	Notch, NOTCH1 precursor
568	regulation of toll-like receptor signaling pathway	46	9.724E-03	3.802E-02	9.724E-03	3.802E-02	2	TIRAP (Mal), NFKBIL1
569	spinal cord development	136	9.752E-03	3.807E-02	9.752E-03	3.807E-02	3	LHX1, Notch, NOTCH1 precursor
570	regulation of lymphocyte proliferation	259	9.873E-03	3.847E-02	9.873E-03	3.847E-02	4	LST1, IRT-1, TIRAP (Mal), CD43
571	macromolecule localization	2206	9.943E-0	3.867E-0	9.943E-0	3.867E-0	14	Ajuba, Rab-11A, SNX1, Olfactory receptor, Notch, Rab-3B, ERK1 (MAPK3), NOTCH1 precursor, 14-3-3 zeta/delta, NFKBIL1, Rab-3, Protrudin, ERK1/2, 14-3-3

			3	2	3	2		
572	regulation of mononuclear cell proliferation	260	1.000E-02	3.884E-02	1.000E-02	3.884E-02	4	LST1, IRT-1, TIRAP (Mal), CD43
573	leukocyte mediated immunity	262	1.027E-02	3.980E-02	1.027E-02	3.980E-02	4	IGHG4, IGHG1, 14-3-3 zeta/delta, 14-3-3
574	immune response-regulating signaling pathway	572	1.036E-02	4.009E-02	1.036E-02	4.009E-02	6	IGHG4, TIRAP (Mal), IGHG1, ERK1 (MAPK3), ERK1/2, 14-3-3
575	cellular response to biotic stimulus	263	1.040E-02	4.009E-02	1.040E-02	4.009E-02	4	TIRAP (Mal), ERK1 (MAPK3), NFKBIL1, ERK1/2
576	multicellular organismal reproductive process	1135	1.052E-02	4.009E-02	1.052E-02	4.009E-02	9	Tissue kallikreins, Cyclin B1, AL1A1, Olfactory receptor, Notch, NOTCH1 precursor, MTL5, Cyclin B, DEAF

577	protein import into nucleus	140	1.055E-02	4.009E-02	1.055E-02	4.009E-02	3	Olfactory receptor, ERK1 (MAPK3), ERK1/2
578	protein targeting to nucleus	140	1.055E-02	4.009E-02	1.055E-02	4.009E-02	3	Olfactory receptor, ERK1 (MAPK3), ERK1/2
579	single-organism nuclear import	140	1.055E-02	4.009E-02	1.055E-02	4.009E-02	3	Olfactory receptor, ERK1 (MAPK3), ERK1/2
580	mitotic spindle checkpoint	48	1.055E-02	4.009E-02	1.055E-02	4.009E-02	2	Cyclin B1, Cyclin B
581	trabecula morphogenesis	48	1.055E-02	4.009E-02	1.055E-02	4.009E-02	2	Notch, NOTCH1 precursor
58	cellular response to alkaloid	48	1.	4.	1.	4.	2	IRT-1, Spinophilin

2			055E-02	009E-02	055E-02	009E-02		
583	regulation of transcription from RNA polymerase II promoter in response to hypoxia	48	1.055E-02	4.009E-02	1.055E-02	4.009E-02	2	Notch, NOTCH1 precursor
584	coronary vasculature morphogenesis	48	1.055E-02	4.009E-02	1.055E-02	4.009E-02	2	Notch, NOTCH1 precursor
585	regulation of localization	2455	1.056E-02	4.009E-02	1.056E-02	4.009E-02	15	Ajuba, Cyclin B1, IRT-1, Rab-11A, TIRAP (Mal), Notch, Rab-3B, ERK1 (MAPK3), NOTCH1 precursor, 14-3-3 zeta/delta, NFKBIL1, Cyclin B, Rab-3, ERK1/2, 14-3-3
586	establishment of protein localization to organelle	411	1.058E-02	4.009E-02	1.058E-02	4.009E-02	5	Olfactory receptor, ERK1 (MAPK3), 14-3-3 zeta/delta, ERK1/2, 14-3-3
587	positive regulation of phosphorus metabolic process	1138	1.06	4.03	1.06	4.03	9	Ajuba, Cyclin B1, IRT-1, TIRAP (Mal), ERK1 (MAPK3), CD43, Cyclin B, ERK1/2, 14-3-3

			9E-02	8E-02	9E-02	8E-02		
588	positive regulation of phosphate metabolic process	1138	1.069E-02	4.038E-02	1.069E-02	4.038E-02	9	Ajuba, Cyclin B1, IRT-1, TIRAP (Mal), ERK1 (MAPK3), CD43, Cyclin B, ERK1/2, 14-3-3
589	positive regulation of apoptotic process	578	1.087E-02	4.099E-02	1.087E-02	4.099E-02	6	Tissue kallikreins, Kallikrein 1, AL1A1, Notch, NOTCH1 precursor, PLEKHG2
590	regulation of leukocyte proliferation	267	1.095E-02	4.105E-02	1.095E-02	4.105E-02	4	LST1, IRT-1, TIRAP (Mal), CD43
591	response to interleukin-1	142	1.096E-02	4.105E-02	1.096E-02	4.105E-02	3	TIRAP (Mal), ERK1 (MAPK3), ERK1/2
592	astrocyte differentiation	49	1.098E	4.105E	1.098E	4.105E	2	Notch, NOTCH1 precursor

			-02	-02	-02	-02		
593	regulation of histone acetylation	49	1.098E-02	4.105E-02	1.098E-02	4.105E-02	2	ERK1 (MAPK3), ERK1/2
594	negative regulation of BMP signaling pathway	49	1.098E-02	4.105E-02	1.098E-02	4.105E-02	2	Notch, NOTCH1 precursor
595	regulation of angiogenesis	268	1.109E-02	4.130E-02	1.109E-02	4.130E-02	4	Tissue kallikreins, Notch, NOTCH1 precursor, Kallikrein 3 (PSA)
596	positive regulation of response to external stimulus	268	1.109E-02	4.130E-02	1.109E-02	4.130E-02	4	Tissue kallikreins, Kallikrein 1, IRT-1, TIRAP (Mal)
597	cell cycle phase transition	416	1.110E-0	4.130E-0	1.110E-0	4.130E-0	5	Ajuba, Cyclin B1, TIPIN, Cyclin B, 14-3-3

			2	2	2	2		
598	positive regulation of MAP kinase activity	270	1.137E-02	4.209E-02	1.137E-02	4.209E-02	4	Ajuba, TIRAP (Mal), ERK1 (MAPK3), ERK1/2
599	regulation of embryonic development	144	1.138E-02	4.209E-02	1.138E-02	4.209E-02	3	LHX1, Notch, NOTCH1 precursor
600	nuclear import	144	1.138E-02	4.209E-02	1.138E-02	4.209E-02	3	Olfactory receptor, ERK1 (MAPK3), ERK1/2
601	negative regulation of cell-substrate adhesion	50	1.141E-02	4.209E-02	1.141E-02	4.209E-02	2	Notch, NOTCH1 precursor
602	negative regulation of ossification	50	1.141E-02	4.209E-02	1.141E-02	4.209E-02	2	Notch, NOTCH1 precursor

603	positive regulation of cell migration	419	1.143E-02	4.209E-02	1.143E-02	4.209E-02	5	IRT-1, TIRAP (Mal), Notch, NOTCH1 precursor, ERK1/2
604	positive regulation of programmed cell death	585	1.149E-02	4.224E-02	1.149E-02	4.224E-02	6	Tissue kallikreins, Kallikrein 1, AL1A1, Notch, NOTCH1 precursor, PLEKHG2
605	multicellular organism reproduction	1156	1.177E-02	4.319E-02	1.177E-02	4.319E-02	9	Tissue kallikreins, Cyclin B1, AL1A1, Olfactory receptor, Notch, NOTCH1 precursor, MTL5, Cyclin B, DEAF
606	placenta blood vessel development	51	1.185E-02	4.345E-02	1.185E-02	4.345E-02	2	Notch, ERK1/2
607	connective tissue development	274	1.195E-02	4.371E-02	1.195E-02	4.371E-02	4	Notch, ERK1 (MAPK3), NOTCH1 precursor, ERK1/2
60	inflammatory response to antigenic stimulus	52	1.	4.	1.	4.	2	Notch, NOTCH1 precursor

8			230E-02	479E-02	230E-02	479E-02		
609	cardiac muscle tissue growth	52	1.230E-02	4.479E-02	1.230E-02	4.479E-02	2	Notch, NOTCH1 precursor
610	positive regulation of vasoconstriction	52	1.230E-02	4.479E-02	1.230E-02	4.479E-02	2	Tissue kallikreins, Kallikrein 1
611	response to hypoxia	427	1.232E-02	4.479E-02	1.232E-02	4.479E-02	5	Ajuba, Cyclin B1, Notch, NOTCH1 precursor, Cyclin B
612	response to toxic substance	277	1.239E-02	4.494E-02	1.239E-02	4.494E-02	4	Cyclin B1, ERK1 (MAPK3), Cyclin B, ERK1/2
613	positive regulation of protein metabolic process	1374	1.24	4.49	1.24	4.49	10	Ajuba, Cyclin B1, IRT-1, SNX1, TIRAP (Mal), ERK1 (MAPK3), CD43, Cyclin B, ERK1/2, 14-3-3

			2E-02	4E-02	2E-02	4E-02		
614	positive regulation of cell motility	430	1.267E-02	4.494E-02	1.267E-02	4.494E-02	5	IRT-1, TIRAP (Mal), Notch, NOTCH1 precursor, ERK1/2
615	regulation of osteoblast differentiation	150	1.270E-02	4.494E-02	1.270E-02	4.494E-02	3	Notch, NOTCH1 precursor, ZHX3
616	oocyte development	53	1.276E-02	4.494E-02	1.276E-02	4.494E-02	2	Cyclin B1, Cyclin B
617	hair cell differentiation	53	1.276E-02	4.494E-02	1.276E-02	4.494E-02	2	Notch, NOTCH1 precursor
618	spindle checkpoint	53	1.276E	4.494E	1.276E	4.494E	2	Cyclin B1, Cyclin B

			-02	-02	-02	-02		
619	regulation of skeletal muscle fiber development	53	1.276E-02	4.494E-02	1.276E-02	4.494E-02	2	Notch, NOTCH1 precursor
620	regulation of cytoskeleton organization	431	1.279E-02	4.494E-02	1.279E-02	4.494E-02	5	Cyclin B1, Olfactory receptor, ERK1 (MAPK3), Cyclin B, ERK1/2
621	regulation of system process	599	1.279E-02	4.494E-02	1.279E-02	4.494E-02	6	Tissue kallikreins, Kallikrein 1, IRT-1, Thioredoxin-like 2, Adult hemoglobin, 14-3-3
622	positive regulation of posttranscriptional gene silencing	4	1.287E-02	4.494E-02	1.287E-02	4.494E-02	1	Ajuba
623	ectoderm formation	4	1.287E-0	4.494E-0	1.287E-0	4.494E-0	1	LHX1

			2	2	2	2		
624	positive regulation of toll-like receptor 3 signaling pathway	4	1.287E-02	4.494E-02	1.287E-02	4.494E-02	1	TIRAP (Mal)
625	motor neuron migration	4	1.287E-02	4.494E-02	1.287E-02	4.494E-02	1	LHX1
626	ciliary body morphogenesis	4	1.287E-02	4.494E-02	1.287E-02	4.494E-02	1	Notch
627	positive regulation of smooth muscle cell chemotaxis	4	1.287E-02	4.494E-02	1.287E-02	4.494E-02	1	IRT-1
628	metanephric part of ureteric bud development	4	1.287E-02	4.494E-02	1.287E-02	4.494E-02	1	LHX1

629	spinal cord motor neuron migration	4	1.287E-02	4.494E-02	1.287E-02	4.494E-02	1	LHX1
630	negative regulation of type IV hypersensitivity	4	1.287E-02	4.494E-02	1.287E-02	4.494E-02	1	CD43
631	regulation of cellular response to hypoxia	4	1.287E-02	4.494E-02	1.287E-02	4.494E-02	1	Ajuba
632	replication fork protection	4	1.287E-02	4.494E-02	1.287E-02	4.494E-02	1	TIPIN
633	oviduct development	4	1.287E-02	4.494E-02	1.287E-02	4.494E-02	1	LHX1
63	regulation of epidermal cell division	4	1.	4.	1.	4.	1	14-3-3

4			287E-02	494E-02	287E-02	494E-02		
635	UDP-glucuronate metabolic process	4	1.287E-02	4.494E-02	1.287E-02	4.494E-02	1	UGDH
636	positive regulation of gene silencing by miRNA	4	1.287E-02	4.494E-02	1.287E-02	4.494E-02	1	Ajuba
637	positive regulation of molecular function	1818	1.289E-02	4.494E-02	1.289E-02	4.494E-02	12	Ajuba, Tissue kallikreins, Cyclin B1, AL1A1, TIRAP (Mal), ERK1 (MAPK3), AO7, Cyclin B, Rab-3, Kallikrein 3 (PSA), ERK1/2, 14-3-3
638	response to decreased oxygen levels	433	1.302E-02	4.533E-02	1.302E-02	4.533E-02	5	Ajuba, Cyclin B1, Notch, NOTCH1 precursor, Cyclin B
639	regulation of systemic arterial blood pressure by hormone	54	1.32	4.56	1.32	4.56	2	Tissue kallikreins, Kallikrein 3 (PSA)

			2E-02	7E-02	2E-02	7E-02		
640	regulation of peptidyl-lysine acetylation	54	1.322E-02	4.567E-02	1.322E-02	4.567E-02	2	ERK1 (MAPK3), ERK1/2
641	glandular epithelial cell differentiation	54	1.322E-02	4.567E-02	1.322E-02	4.567E-02	2	Notch, NOTCH1 precursor
642	regulation of calcium ion-dependent exocytosis	54	1.322E-02	4.567E-02	1.322E-02	4.567E-02	2	Notch, NOTCH1 precursor
643	renal tubule development	54	1.322E-02	4.567E-02	1.322E-02	4.567E-02	2	Notch, NOTCH1 precursor
644	MAPK cascade	283	1.331E	4.592E	1.331E	4.592E	4	TIRAP (Mal), ERK1 (MAPK3), ERK1/2, 14-3-3

			-02	-02	-02	-02		
645	cellular component assembly	2054	1.337E-02	4.602E-02	1.337E-02	4.602E-02	13	Ajuba, Cyclin B1, AL1A1, HBG, IRT-1, HBGA, ERK1 (MAPK3), Spinophilin, Cyclin B, Adult hemoglobin, ERK1/2, 14-3-3, RBBP7 (RbAp46)
646	positive regulation of gene expression	1607	1.346E-02	4.629E-02	1.346E-02	4.629E-02	11	LHX1, Gpbp1, TEF-5, PTD015, Notch, ERK1 (MAPK3), SRB7, NOTCH1 precursor, ERK1/2, DEAF, 14-3-3
647	negative regulation of endothelial cell migration	55	1.369E-02	4.679E-02	1.369E-02	4.679E-02	2	Notch, NOTCH1 precursor
648	regulated secretory pathway	55	1.369E-02	4.679E-02	1.369E-02	4.679E-02	2	14-3-3 zeta/delta, 14-3-3
649	positive regulation of acute inflammatory response	55	1.369E-0	4.679E-0	1.369E-0	4.679E-0	2	Tissue kallikreins, Kallikrein 1

			2	2	2	2		
650	cell differentiation involved in kidney development	55	1.369E-02	4.679E-02	1.369E-02	4.679E-02	2	Notch, NOTCH1 precursor
651	growth	611	1.399E-02	4.755E-02	1.399E-02	4.755E-02	6	Tissue kallikreins, Cyclin B1, LHX1, Notch, NOTCH1 precursor, Cyclin B
652	response to bacterium	795	1.408E-02	4.755E-02	1.408E-02	4.755E-02	7	TIRAP (Mal), Notch, ERK1 (MAPK3), NOTCH1 precursor, CD43, NFKBIL1, ERK1/2
653	positive regulation of ERK1 and ERK2 cascade	156	1.410E-02	4.755E-02	1.410E-02	4.755E-02	3	TIRAP (Mal), ERK1 (MAPK3), ERK1/2
654	striated muscle cell differentiation	288	1.412E-02	4.755E-02	1.412E-02	4.755E-02	4	Cyclin B1, Notch, NOTCH1 precursor, Cyclin B

655	regulation of vasculature development	288	1.412E-02	4.755E-02	1.412E-02	4.755E-02	4	Tissue kallikreins, Notch, NOTCH1 precursor, Kallikrein 3 (PSA)
656	negative regulation of osteoblast differentiation	56	1.417E-02	4.755E-02	1.417E-02	4.755E-02	2	Notch, NOTCH1 precursor
657	oocyte differentiation	56	1.417E-02	4.755E-02	1.417E-02	4.755E-02	2	Cyclin B1, Cyclin B
658	stem cell division	56	1.417E-02	4.755E-02	1.417E-02	4.755E-02	2	Notch, NOTCH1 precursor
659	regulation of protein acetylation	56	1.417E-02	4.755E-02	1.417E-02	4.755E-02	2	ERK1 (MAPK3), ERK1/2
66	heart growth	56	1.	4.	1.	4.	2	Notch, NOTCH1 precursor

0			417E-02	755E-02	417E-02	755E-02		
661	positive regulation of glial cell differentiation	56	1.417E-02	4.755E-02	1.417E-02	4.755E-02	2	Notch, NOTCH1 precursor
662	negative regulation of muscle cell differentiation	56	1.417E-02	4.755E-02	1.417E-02	4.755E-02	2	Notch, NOTCH1 precursor
663	positive regulation of cellular component movement	443	1.425E-02	4.774E-02	1.425E-02	4.774E-02	5	IRT-1, TIRAP (Mal), Notch, NOTCH1 precursor, ERK1/2
664	muscle contraction	289	1.428E-02	4.777E-02	1.428E-02	4.777E-02	4	Tissue kallikreins, Leiomodin, Kallikrein 3 (PSA), CapZIP
665	platelet activation	290	1.44	4.82	1.44	4.82	4	ERK1 (MAPK3), 14-3-3 zeta/delta, ERK1/2, 14-3-3

			5E-02	5E-02	5E-02	5E-02		
666	immune response-activating signal transduction	445	1.451E-02	4.837E-02	1.451E-02	4.837E-02	5	IGHG4, TIRAP (Mal), IGHG1, ERK1 (MAPK3), ERK1/2
667	establishment of organelle localization	291	1.461E-02	4.866E-02	1.461E-02	4.866E-02	4	Cyclin B1, Rab-11A, Cyclin B, Rab-3
668	positive regulation of amine transport	57	1.466E-02	4.873E-02	1.466E-02	4.873E-02	2	Rab-3B, Rab-3
669	cytoplasmic transport	803	1.481E-02	4.916E-02	1.481E-02	4.916E-02	7	Rab-11A, SNX1, Olfactory receptor, ERK1 (MAPK3), 14-3-3 zeta/delta, ERK1/2, 14-3-3
670	positive regulation of locomotion	449	1.502E	4.981E	1.502E	4.981E	5	IRT-1, TIRAP (Mal), Notch, NOTCH1 precursor, ERK1/2

			-02	-02	-02	-02		
671	response to growth hormone	58	1.515E-02	4.984E-02	1.515E-02	4.984E-02	2	ERK1 (MAPK3), ERK1/2
672	positive regulation of mitotic cell cycle	58	1.515E-02	4.984E-02	1.515E-02	4.984E-02	2	Cyclin B1, Cyclin B
673	regulation of phosphatidylinositol 3-kinase activity	58	1.515E-02	4.984E-02	1.515E-02	4.984E-02	2	Tissue kallikreins, Kallikrein 3 (PSA)
674	hindlimb morphogenesis	58	1.515E-02	4.984E-02	1.515E-02	4.984E-02	2	Notch, NOTCH1 precursor
675	myeloid cell activation involved in immune response	58	1.515E-0	4.984E-0	1.515E-0	4.984E-0	2	14-3-3 zeta/delta, 14-3-3

			2	2	2	2		
676	regulation of gene expression	5135	1.522E-02	4.995E-02	1.522E-02	4.995E-02	25	Ajuba, Cyclin B1, LHX1, HBG, IRT-1, HBGA, Gpbp1, TEF-5, TIRAP (Mal), Olfactory receptor, PTD015, Notch, ERK1 (MAPK3), DR1, SRB7, NOTCH1 precursor, NFKBIL1, AO7, Cyclin B, Adult hemoglobin, ZHX3, ERK1/2, DEAF, 14-3-3, RBBP7 (RbAp46)
677	single organism reproductive process	1418	1.523E-02	4.995E-02	1.523E-02	4.995E-02	10	Tissue kallikreins, Cyclin B1, AL1A1, LHX1, Olfactory receptor, Notch, NOTCH1 precursor, MTL5, Cyclin B, DEAF

Table 9S: Enrichment analysis report. Enrichment by GO Processes (healthy control vs SVZn)

Enrichment by GO Processes					SVZn Limma Input_genelist			
**#**	**Processes**	**Total**	**pValue**	**Min FDR**	**p-value**	**FDR**	**In Data**	**Network Objects from Active Data**
1	single-organism organelle organization	1878	2.340E-10	5.662E-07	2.340E-10	5.662E-07	30	RBBP8 (CtIP), Cyclin B1, DLC1 (Dynein LC8a), HSPA1B, TIPIN, PPM1F, HDAC7, MENA, VAMP5, Histone deacetylase class II, Calmyrin, HSP70, ARID4A, SET, SMYD3, POLE3-POLE4 complex, HSPA2, HSPA1A, CKS2, OBFC1, POLE3 (YBL1), DYNLL, SET/NUP214 fusion protein, Cyclin B, EYA1, Pleckstrin, FOP, PDXP, PP2C, RUVBL1
2	negative regulation of phosphorus metabolic process	487	3.705E-09	2.167E-06	3.705E-09	2.167E-06	15	Cyclin B1, DLC1 (Dynein LC8a), PSMD10 (Gankyrin), PPM1F, HEXIM1, Calmyrin, HSP70, SMYD3, DYNLL, Cyclin B, Pleckstrin, PTPN2, FOP, CAMK2N1, PP2C
3	negative regulation of phosphate metabolic process	487	3.705E-09	2.167E-06	3.705E-09	2.167E-06	15	Cyclin B1, DLC1 (Dynein LC8a), PSMD10 (Gankyrin), PPM1F, HEXIM1, Calmyrin, HSP70, SMYD3, DYNLL, Cyclin B, Pleckstrin, PTPN2, FOP, CAMK2N1, PP2C
4	chromosome organization	941	4.258E-09	2.167E-06	4.258E-09	2.167E-06	20	Cyclin B1, HSPA1B, PPM1F, HDAC7, Histone deacetylase class II, HSP70, ARID4A, LOC646817, SET, SMYD3, POLE3-POLE4 complex, HSPA2, HSPA1A, OBFC1, POLE3 (YBL1), SET/NUP214 fusion protein, Cyclin B, EYA1, PP2C, RUVBL1

5	negative regulation of phosphorylation	418	4.478E-09	2.167E-06	4.478E-09	2.167E-06	14	Cyclin B1, DLC1 (Dynein LC8a), PSMD10 (Gankyrin), PPM1F, HEXIM1, Calmyrin, HSP70, SMYD3, DYNLL, Cyclin B, PTPN2, FOP, CAMK2N1, PP2C
6	protein refolding	22	3.832E-08	1.546E-05	3.832E-08	1.546E-05	5	HSPA1B, HSP70, HSPA2, HSPA1A, HSC70
7	macromolecular complex subunit organization	1823	5.499E-08	1.572E-05	5.499E-08	1.572E-05	26	Annexin V, eEF1A, Cyclin B1, PSMD10 (Gankyrin), Clp1, AL1A1, MENA, DARS, Histone deacetylase class II, Calmyrin, COLEC12, HSP70, Annexin VI, LOC646817, SET, TXNRD1, SMYD3, eEF1A1, CKS2, SET/NUP214 fusion protein, Cyclin B, IMD2, PDXP, HSC70, RUVBL1, PQBP-1
8	regulation of cyclin-dependent protein serine/threonine kinase activity	119	5.802E-08	1.572E-05	5.802E-08	1.572E-05	8	Cyclin T1, Cyclin B1, PSMD10 (Gankyrin), HEXIM1, HSP70, HSPA2, CKS2, Cyclin B
9	negative regulation of protein modification process	519	6.750E-08	1.572E-05	6.750E-08	1.572E-05	14	Cyclin B1, PSMD10 (Gankyrin), PPM1F, HEXIM1, Calmyrin, HSP70, SET, SMYD3, SET/NUP214 fusion protein, Cyclin B, PTPN2, FOP, CAMK2N1, PP2C
10	negative regulation of inclusion body assembly	10	7.	1.	7.	1.	4	HSPA1B, HSP70, HSPA2, HSPA1A

			482E-08	572E-05	482E-08	572E-05		
11	protein complex subunit organization	1596	7.717E-08	1.572E-05	7.717E-08	1.572E-05	24	Annexin V, eEF1A, Cyclin B1, PSMD10 (Gankyrin), AL1A1, MENA, DARS, Histone deacetylase class II, Calmyrin, COLEC12, HSP70, Annexin VI, LOC646817, SET, TXNRD1, SMYD3, eEF1A1, CKS2, SET/NUP214 fusion protein, Cyclin B, IMD2, PDXP, HSC70, RUVBL1
12	histone modification	368	8.326E-08	1.572E-05	8.326E-08	1.572E-05	12	Cyclin B1, PPM1F, HDAC7, Histone deacetylase class II, ARID4A, SMYD3, POLE3-POLE4 complex, POLE3 (YBL1), Cyclin B, EYA1, PP2C, RUVBL1
13	cellular component organization	5419	8.976E-08	1.572E-05	8.976E-08	1.572E-05	48	Annexin V, eEF1A, Reticulon 4, RBBP8 (CtIP), Cyclin B1, DLC1 (Dynein LC8a), HSPA1B, PSMD10 (Gankyrin), TIPIN, Clp1, AL1A1, PPM1F, HDAC7, MENA, DARS, VAMP5, Histone deacetylase class II, Olfactory receptor, Calmyrin, COLEC12, HSP70, ARID4A, Annexin VI, LOC646817, SET, TXNRD1, SMYD3, POLE3-POLE4 complex, Calmodulin, HSPA2, HSPA1A, eEF1A1, CKS2, OBFC1, POLE3 (YBL1), DYNLL, ICMT, SET/NUP214 fusion protein, Cyclin B, IMD2, EYA1, Pleckstrin, FOP, PDXP, PP2C, HSC70, RUVBL1, PQBP-1
14	covalent chromatin modification	371	9.093E-08	1.572E-05	9.093E-08	1.572E-05	12	Cyclin B1, PPM1F, HDAC7, Histone deacetylase class II, ARID4A, SMYD3, POLE3-POLE4 complex, POLE3 (YBL1), Cyclin B, EYA1, PP2C, RUVBL1
15	mitotic cell cycle	930	1.1	1.9	1.1	1.9	18	RBBP8 (CtIP), Cyclin B1, DLC1 (Dynein LC8a), PSMD10 (Gankyrin), TIPIN, Calmyrin, HSP70, SET, Calmodulin, HSPA2, DYNLL, SET/NUP214 fusion protein, Cyclin B,

			78E-07	00E-05	78E-07	00E-05		EYA1, FOP, PP2C, PRCC, RUVBL1
16	cellular component organization or biogenesis	5557	2.056E-07	3.109E-05	2.056E-07	3.109E-05	48	Annexin V, eEF1A, Reticulon 4, RBBP8 (CtIP), Cyclin B1, DLC1 (Dynein LC8a), HSPA1B, PSMD10 (Gankyrin), TIPIN, Clp1, AL1A1, PPM1F, HDAC7, MENA, DARS, VAMP5, Histone deacetylase class II, Olfactory receptor, Calmyrin, COLEC12, HSP70, ARID4A, Annexin VI, LOC646817, SET, TXNRD1, SMYD3, POLE3-POLE4 complex, Calmodulin, HSPA2, HSPA1A, eEF1A1, CKS2, OBFC1, POLE3 (YBL1), DYNLL, ICMT, SET/NUP214 fusion protein, Cyclin B, IMD2, EYA1, Pleckstrin, FOP, PDXP, PP2C, HSC70, RUVBL1, PQBP-1
17	organelle organization	2976	2.196E-07	3.127E-05	2.196E-07	3.127E-05	33	Reticulon 4, RBBP8 (CtIP), Cyclin B1, DLC1 (Dynein LC8a), HSPA1B, TIPIN, PPM1F, HDAC7, MENA, VAMP5, Histone deacetylase class II, Calmyrin, HSP70, ARID4A, LOC646817, SET, SMYD3, POLE3-POLE4 complex, HSPA2, HSPA1A, CKS2, OBFC1, POLE3 (YBL1), DYNLL, SET/NUP214 fusion protein, Cyclin B, EYA1, Pleckstrin, FOP, PDXP, PP2C, RUVBL1, PQBP-1
18	G2/M transition of mitotic cell cycle	200	2.640E-07	3.362E-05	2.640E-07	3.362E-05	9	Cyclin B1, DLC1 (Dynein LC8a), HSP70, Calmodulin, HSPA2, DYNLL, Cyclin B, FOP, PP2C
19	cell cycle G2/M phase transition	200	2.640E-07	3.362E-05	2.640E-07	3.362E-05	9	Cyclin B1, DLC1 (Dynein LC8a), HSP70, Calmodulin, HSPA2, DYNLL, Cyclin B, FOP, PP2C
20	negative regulation of cellular protein metabolic process	679	2.91	3.52	2.91	3.52	15	Cyclin B1, PSMD10 (Gankyrin), PPM1F, HEXIM1, Histone deacetylase class II, Calmyrin, HSP70, SET, SMYD3, SET/NUP214 fusion protein, Cyclin B, PTPN2, FOP, CAMK2N1, PP2C

			5E-07	7E-05	5E-07	7E-05		
21	cell cycle phase transition	416	3.115E-07	3.590E-05	3.115E-07	3.590E-05	12	RBBP8 (CtIP), Cyclin B1, DLC1 (Dynein LC8a), PSMD10 (Gankyrin), TIPIN, HSP70, Calmodulin, HSPA2, DYNLL, Cyclin B, FOP, PP2C
22	chromatin organization	704	4.613E-07	5.074E-05	4.613E-07	5.074E-05	15	Cyclin B1, PPM1F, HDAC7, Histone deacetylase class II, ARID4A, LOC646817, SET, SMYD3, POLE3-POLE4 complex, POLE3 (YBL1), SET/NUP214 fusion protein, Cyclin B, EYA1, PP2C, RUVBL1
23	negative regulation of protein phosphorylation	354	4.838E-07	5.090E-05	4.838E-07	5.090E-05	11	Cyclin B1, PPM1F, HEXIM1, Calmyrin, HSP70, SMYD3, Cyclin B, PTPN2, FOP, CAMK2N1, PP2C
24	negative regulation of cellular component organization	615	5.300E-07	5.344E-05	5.300E-07	5.344E-05	14	Reticulon 4, Cyclin B1, HSPA1B, PSMD10 (Gankyrin), Histone deacetylase class II, Calmyrin, HSP70, SET, HSPA2, HSPA1A, SET/NUP214 fusion protein, Cyclin B, PP2C, HSC70
25	histone dephosphorylation	5	8.412E	8.143E	8.412E	8.143E	3	PPM1F, EYA1, PP2C

			-07	-05	-07	-05		
26	mitotic cell cycle process	850	9.472E-07	8.816E-05	9.472E-07	8.816E-05	16	RBBP8 (CtIP), Cyclin B1, DLC1 (Dynein LC8a), PSMD10 (Gankyrin), TIPIN, HSP70, Calmodulin, HSPA2, DYNLL, SET/NUP214 fusion protein, Cyclin B, EYA1, FOP, PP2C, PRCC, RUVBL1
27	cell cycle	1580	1.131E-06	1.014E-04	1.131E-06	1.014E-04	22	RBBP8 (CtIP), Cyclin T1, Cyclin B1, DLC1 (Dynein LC8a), PSMD10 (Gankyrin), TIPIN, Calmyrin, HSP70, SET, Calmodulin, HSPA2, CKS2, Annexin XI, DYNLL, Septin 3, SET/NUP214 fusion protein, Cyclin B, EYA1, FOP, PP2C, PRCC, RUVBL1
28	negative regulation of protein metabolic process	780	1.661E-06	1.436E-04	1.661E-06	1.436E-04	15	Cyclin B1, PSMD10 (Gankyrin), PPM1F, HEXIM1, Histone deacetylase class II, Calmyrin, HSP70, SET, SMYD3, SET/NUP214 fusion protein, Cyclin B, PTPN2, FOP, CAMK2N1, PP2C
29	mitotic cell cycle phase transition	413	2.179E-06	1.819E-04	2.179E-06	1.819E-04	11	RBBP8 (CtIP), Cyclin B1, DLC1 (Dynein LC8a), PSMD10 (Gankyrin), HSP70, Calmodulin, HSPA2, DYNLL, Cyclin B, FOP, PP2C
30	negative regulation of cellular metabolic process	2082	2.677E-0	2.160E-0	2.677E-0	2.160E-0	25	RBBP8 (CtIP), Cyclin B1, DLC1 (Dynein LC8a), PSMD10 (Gankyrin), TIPIN, PPM1F, HDAC7, HEXIM1, Histone deacetylase class II, Calmyrin, HSP70, ARID4A, SET, Follistatin, SMYD3, DYNLL, SET/NUP214 fusion protein, Cyclin B, Pleckstrin, PTPN2, FOP, CAMK2N1, PP2C, HSC70, TARDBP (TDP43)

			6	4	6	4		
31	regulation of protein depolymerization	89	2.805E-06	2.189E-04	2.805E-06	2.189E-04	6	Cyclin B1, Histone deacetylase class II, Calmyrin, Cyclin B, Pleckstrin, PDXP
32	macromolecular complex assembly	1415	3.084E-06	2.332E-04	3.084E-06	2.332E-04	20	Annexin V, eEF1A, Cyclin B1, PSMD10 (Gankyrin), Clp1, AL1A1, DARS, COLEC12, HSP70, Annexin VI, LOC646817, SET, TXNRD1, SMYD3, eEF1A1, SET/NUP214 fusion protein, Cyclin B, IMD2, RUVBL1, PQBP-1
33	negative regulation of metabolic process	2294	4.630E-06	3.310E-04	4.630E-06	3.310E-04	26	RBBP8 (CtIP), Cyclin B1, DLC1 (Dynein LC8a), PSMD10 (Gankyrin), TIPIN, Clp1, PPM1F, HDAC7, HEXIM1, Histone deacetylase class II, Calmyrin, HSP70, ARID4A, SET, Follistatin, SMYD3, DYNLL, SET/NUP214 fusion protein, Cyclin B, Pleckstrin, PTPN2, FOP, CAMK2N1, PP2C, HSC70, TARDBP (TDP43)
34	protein complex assembly	1200	4.651E-06	3.310E-04	4.651E-06	3.310E-04	18	Annexin V, eEF1A, Cyclin B1, PSMD10 (Gankyrin), AL1A1, DARS, COLEC12, HSP70, Annexin VI, LOC646817, SET, TXNRD1, SMYD3, eEF1A1, SET/NUP214 fusion protein, Cyclin B, IMD2, RUVBL1
35	protein complex biogenesis	1203	4.815E-06	3.329E-04	4.815E-06	3.329E-04	18	Annexin V, eEF1A, Cyclin B1, PSMD10 (Gankyrin), AL1A1, DARS, COLEC12, HSP70, Annexin VI, LOC646817, SET, TXNRD1, SMYD3, eEF1A1, SET/NUP214 fusion protein, Cyclin B, IMD2, RUVBL1

36	negative regulation of catalytic activity	975	5.554E-06	3.733E-04	5.554E-06	3.733E-04	16	Annexin V, DLC1 (Dynein LC8a), HSPA1B, PSMD10 (Gankyrin), PPM1F, HEXIM1, Histone deacetylase class II, HSP70, SET, SMYD3, HSPA1A, DYNLL, SET/NUP214 fusion protein, FOP, CAMK2N1, PP2C
37	regulation of protein complex disassembly	101	5.862E-06	3.834E-04	5.862E-06	3.834E-04	6	Cyclin B1, Histone deacetylase class II, Calmyrin, Cyclin B, Pleckstrin, PDXP
38	heat acclimation	9	6.976E-06	4.177E-04	6.976E-06	4.177E-04	3	HSPA1B, HSP70, HSPA1A
39	cellular heat acclimation	9	6.976E-06	4.177E-04	6.976E-06	4.177E-04	3	HSPA1B, HSP70, HSPA1A
40	positive regulation of biological process	5249	7.042E-06	4.177E-04	7.042E-06	4.177E-04	43	Annexin V, eEF1A, Reticulon 4, IGHG4, Cyclin T1, Cyclin B1, DLC1 (Dynein LC8a), HSPA1B, PSMD10 (Gankyrin), TIPIN, ECE1, AL1A1, PPM1F, HDAC7, MENA, SNX1, Histone deacetylase class II, Olfactory receptor, Calmyrin, EPS8L1, COLEC12, HSP70, IGHG1, Cyclophilin E, LOC646817, Follistatin, TXNRD1, SMYD3, Calmodulin, HSPA2, HSPA1A, eEF1A1, OBFC1, DYNLL, ICMT, Cyclin B, EYA1, Pleckstrin, PTPN2, FOP, PDXP, PP2C, TARDBP (TDP43)
41	cell cycle process	12	7.	4.	7.	4.	18	RBBP8 (CtIP), Cyclin B1, DLC1 (Dynein LC8a), PSMD10 (Gankyrin), TIPIN, HSP70, Calmodulin, HSPA2, CKS2,

		37	076E-06	177E-04	076E-06	177E-04		Annexin XI, DYNLL, SET/NUP214 fusion protein, Cyclin B, EYA1, FOP, PP2C, PRCC, RUVBL1
42	negative regulation of microtubule depolymerization	29	7.934E-06	4.571E-04	7.934E-06	4.571E-04	4	Cyclin B1, Histone deacetylase class II, Calmyrin, Cyclin B
43	primary metabolic process	10185	8.599E-06	4.840E-04	8.599E-06	4.840E-04	66	FLJ22494, P5CR1, eEF1A, RBBP8 (CtIP), PGMU, IGHG4, Cyclin T1, RY1, Cyclin B1, DLC1 (Dynein LC8a), HSPA1B, PSMD10 (Gankyrin), TIPIN, Clp1, ECE1, AL1A1, Fbxo3, PPM1F, HDAC7, CDO1, HEXIM1, DARS, Histone deacetylase class II, Calmyrin, HSP70, IGHG1, Cyclophilin E, ARID4A, PGM2, eEF2K, LOC646817, SET, COASY, DPP3, TXNRD1, SMYD3, POLE3-POLE4 complex, Calmodulin, HSPA2, HSPA1A, RBP1, eEF1A1, SAE2, OBFC1, ZNF397OS, POLE3 (YBL1), DYNLL, ICMT, DOHH, ZNF397, SET/NUP214 fusion protein, Cyclin B, IMD2, EYA1, GMEB1, Pleckstrin, PTPN2, FOP, PDXP, Fructosamine-3-kinase, PP2C, HSC70, TARDBP (TDP43), TCOF1, RUVBL1, PQBP-1
44	single-organism metabolic process	5494	9.318E-06	5.125E-04	9.318E-06	5.125E-04	44	FLJ22494, P5CR1, eEF1A, RBBP8 (CtIP), PGMU, IGHG4, Cyclin B1, HSPA1B, PSMD10 (Gankyrin), AL1A1, PPM1F, HDAC7, CDO1, Cytoglobin, DARS, Histone deacetylase class II, Thioredoxin-like 2, Calmyrin, HSP70, IGHG1, ARID4A, PGM2, COASY, TXNRD1, SMYD3, POLE3-POLE4 complex, Calmodulin, HSPA1A, RBP1, eEF1A1, OBFC1, POLE3 (YBL1), ICMT, DOHH, SET/NUP214 fusion protein, Cyclin B, IMD2, EYA1, Pleckstrin, PDXP, Fructosamine-3-kinase, PP2C, HSC70, RUVBL1
45	chromatin modification	581	9.906E-06	5.327E-04	9.906E-06	5.327E-04	12	Cyclin B1, PPM1F, HDAC7, Histone deacetylase class II, ARID4A, SMYD3, POLE3-POLE4 complex, POLE3 (YBL1), Cyclin B, EYA1, PP2C, RUVBL1

46	regulation of inclusion body assembly	31	1.044E-05	5.492E-04	1.044E-05	5.492E-04	4	HSPA1B, HSP70, HSPA2, HSPA1A
47	regulation of catalytic activity	2567	1.157E-05	5.958E-04	1.157E-05	5.958E-04	27	Annexin V, Cyclin T1, Cyclin B1, DLC1 (Dynein LC8a), HSPA1B, PSMD10 (Gankyrin), AL1A1, PPM1F, HEXIM1, Histone deacetylase class II, Calmyrin, EPS8L1, HSP70, SET, SMYD3, Calmodulin, HSPA2, HSPA1A, SAE2, CKS2, DYNLL, SET/NUP214 fusion protein, Cyclin B, Pleckstrin, FOP, CAMK2N1, PP2C
48	regulation of primary metabolic process	6540	1.252E-05	6.310E-04	1.252E-05	6.310E-04	49	eEF1A, RBBP8 (CtIP), Cyclin T1, Cyclin B1, DLC1 (Dynein LC8a), HSPA1B, PSMD10 (Gankyrin), TIPIN, ECE1, AL1A1, PPM1F, HDAC7, SNX1, HEXIM1, Histone deacetylase class II, Olfactory receptor, Calmyrin, EPS8L1, HSP70, Cyclophilin E, ARID4A, eEF2K, LOC646817, SET, Follistatin, SMYD3, Calmodulin, HSPA2, HSPA1A, eEF1A1, CKS2, OBFC1, ZNF397OS, DYNLL, ICMT, ZNF397, SET/NUP214 fusion protein, Cyclin B, EYA1, GMEB1, Pleckstrin, PTPN2, FOP, CAMK2N1, PP2C, HSC70, TARDBP (TDP43), RUVBL1, PQBP-1
49	cellular nitrogen compound metabolic process	5949	1.294E-05	6.388E-04	1.294E-05	6.388E-04	46	FLJ22494, P5CR1, eEF1A, RBBP8 (CtIP), Cyclin T1, RY1, DLC1 (Dynein LC8a), HSPA1B, PSMD10 (Gankyrin), TIPIN, Clp1, ECE1, HDAC7, CDO1, HEXIM1, DARS, Histone deacetylase class II, Calmyrin, HSP70, Cyclophilin E, ARID4A, LOC646817, SET, COASY, TXNRD1, SMYD3, POLE3-POLE4 complex, HSPA1A, eEF1A1, OBFC1, ZNF397OS, POLE3 (YBL1), DYNLL, ICMT, ZNF397, SET/NUP214 fusion protein, IMD2, EYA1, GMEB1, PDXP, PP2C, HSC70, TARDBP (TDP43), TCOF1, RUVBL1, PQBP-1
50	response to ischemia	33	1.348E-05	6.460E-04	1.348E-05	6.460E-04	4	HSPA1B, Calmyrin, HSP70, HSPA1A

51	positive regulation of cell migration involved in sprouting angiogenesis	11	1.361E-05	6.460E-04	1.361E-05	6.460E-04	3	HDAC7, Histone deacetylase class II, Calmyrin
52	regulation of macromolecule metabolic process	6163	1.399E-05	6.508E-04	1.399E-05	6.508E-04	47	eEF1A, RBBP8 (CtIP), Cyclin T1, Cyclin B1, DLC1 (Dynein LC8a), HSPA1B, PSMD10 (Gankyrin), TIPIN, Clp1, ECE1, PPM1F, HDAC7, SNX1, HEXIM1, Histone deacetylase class II, Olfactory receptor, Calmyrin, HSP70, Cyclophilin E, ARID4A, eEF2K, LOC646817, SET, Follistatin, SMYD3, Calmodulin, HSPA2, HSPA1A, eEF1A1, CKS2, OBFC1, ZNF397OS, DYNLL, ICMT, ZNF397, SET/NUP214 fusion protein, Cyclin B, EYA1, GMEB1, PTPN2, FOP, CAMK2N1, PP2C, HSC70, TARDBP (TDP43), RUVBL1, PQBP-1
53	regulation of microtubule depolymerization	34	1.523E-05	6.954E-04	1.523E-05	6.954E-04	4	Cyclin B1, Histone deacetylase class II, Calmyrin, Cyclin B
54	regulation of cell cycle	1062	1.615E-05	7.237E-04	1.615E-05	7.237E-04	16	Cyclin T1, Cyclin B1, PSMD10 (Gankyrin), TIPIN, HEXIM1, Olfactory receptor, HSP70, Calmodulin, HSPA2, CKS2, SET/NUP214 fusion protein, Cyclin B, PDXP, PP2C, HSC70, PRCC
55	regulation of protein modification process	1584	1.653E-05	7.273E-04	1.653E-05	7.273E-04	20	Cyclin T1, Cyclin B1, PSMD10 (Gankyrin), PPM1F, HEXIM1, Histone deacetylase class II, Calmyrin, HSP70, eEF2K, SET, SMYD3, Calmodulin, HSPA2, CKS2, SET/NUP214 fusion protein, Cyclin B, PTPN2, FOP, CAMK2N1, PP2C
56	response to DDT	2	1.	7.	1.	7.	2	Cyclin B1, Cyclin B

			949E-05	996E-04	949E-05	996E-04		
57	synaptonemal complex disassembly	2	1.949E-05	7.996E-04	1.949E-05	7.996E-04	2	HSP70, HSPA2
58	negative regulation of fibril organization	2	1.949E-05	7.996E-04	1.949E-05	7.996E-04	2	HSP70, HSC70
59	regulation of fibril organization	2	1.949E-05	7.996E-04	1.949E-05	7.996E-04	2	HSP70, HSC70
60	nitrogen compound metabolic process	6452	2.106E-05	8.496E-04	2.106E-05	8.496E-04	48	FLJ22494, P5CR1, eEF1A, RBBP8 (CtIP), Cyclin T1, RY1, DLC1 (Dynein LC8a), HSPA1B, PSMD10 (Gankyrin), TIPIN, Clp1, ECE1, HDAC7, CDO1, HEXIM1, DARS, Histone deacetylase class II, Calmyrin, HSP70, Cyclophilin E, ARID4A, LOC646817, SET, COASY, TXNRD1, SMYD3, POLE3-POLE4 complex, Calmodulin, HSPA1A, eEF1A1, OBFC1, ZNF397OS, POLE3 (YBL1), DYNLL, ICMT, ZNF397, SET/NUP214 fusion protein, IMD2, EYA1, GMEB1, PDXP, Fructosamine-3-kinase, PP2C, HSC70, TARDBP (TDP43), TCOF1, RUVBL1, PQBP-1
61	cellular metabolic process	994	2.1	8.6	2.1	8.6	64	FLJ22494, P5CR1, eEF1A, RBBP8 (CtIP), PGMU, Cyclin T1, RY1, Cyclin B1, DLC1 (Dynein LC8a), HSPA1B, PSMD10 (Gankyrin), TIPIN, Clp1, ECE1, AL1A1, Fbxo3, PPM1F,

		1	80E-05	50E-04	80E-05	50E-04		HDAC7, CDO1, HEXIM1, DARS, Histone deacetylase class II, Calmyrin, HSP70, Cyclophilin E, ARID4A, PGM2, eEF2K, LOC646817, SET, COASY, TXNRD1, SMYD3, POLE3-POLE4 complex, Calmodulin, HSPA2, HSPA1A, RBP1, eEF1A1, SAE2, CKS2, OBFC1, ZNF397OS, POLE3 (YBL1), DYNLL, ICMT, DOHH, ZNF397, SET/NUP214 fusion protein, Cyclin B, IMD2, EYA1, GMEB1, Pleckstrin, PTPN2, FOP, PDXP, Fructosamine-3-kinase, PP2C, HSC70, TARDBP (TDP43), TCOF1, RUVBL1, PQBP-1
62	negative regulation of molecular function	1218	2.268E-05	8.851E-04	2.268E-05	8.851E-04	17	Annexin V, DLC1 (Dynein LC8a), HSPA1B, PSMD10 (Gankyrin), PPM1F, HEXIM1, Histone deacetylase class II, HSP70, SET, SMYD3, Calmodulin, HSPA1A, DYNLL, SET/NUP214 fusion protein, FOP, CAMK2N1, PP2C
63	cellular component assembly	2054	2.349E-05	8.996E-04	2.349E-05	8.996E-04	23	Annexin V, eEF1A, Cyclin B1, PSMD10 (Gankyrin), Clp1, AL1A1, HDAC7, DARS, Histone deacetylase class II, COLEC12, HSP70, Annexin VI, LOC646817, SET, TXNRD1, SMYD3, eEF1A1, SET/NUP214 fusion protein, Cyclin B, IMD2, PDXP, RUVBL1, PQBP-1
64	regulation of metabolic process	7302	2.379E-05	8.996E-04	2.379E-05	8.996E-04	52	Annexin V, eEF1A, RBBP8 (CtIP), Cyclin T1, Cyclin B1, DLC1 (Dynein LC8a), HSPA1B, PSMD10 (Gankyrin), TIPIN, Clp1, ECE1, AL1A1, PPM1F, HDAC7, SNX1, HEXIM1, Histone deacetylase class II, Olfactory receptor, Calmyrin, EPS8L1, HSP70, Cyclophilin E, ARID4A, eEF2K, LOC646817, SET, Follistatin, SMYD3, Calmodulin, HSPA2, HSPA1A, eEF1A1, SAE2, CKS2, OBFC1, ZNF397OS, DYNLL, ICMT, ZNF397, SET/NUP214 fusion protein, Cyclin B, EYA1, GMEB1, Pleckstrin, PTPN2, FOP, CAMK2N1, PP2C, HSC70, TARDBP (TDP43), RUVBL1, PQBP-1
65	heterocycle metabolic process	5709	2.646E-05	9.738E-04	2.646E-05	9.738E-04	44	FLJ22494, P5CR1, eEF1A, RBBP8 (CtIP), Cyclin T1, RY1, DLC1 (Dynein LC8a), HSPA1B, PSMD10 (Gankyrin), TIPIN, Clp1, HDAC7, HEXIM1, DARS, Histone deacetylase class II, Calmyrin, HSP70, Cyclophilin E, ARID4A, LOC646817, SET, COASY, TXNRD1, SMYD3, POLE3-POLE4 complex, HSPA1A, eEF1A1, OBFC1, ZNF397OS, POLE3 (YBL1), DYNLL, ICMT, ZNF397, SET/NUP214 fusion protein, IMD2, EYA1, GMEB1, PDXP, PP2C, HSC70, TARDBP (TDP43), TCOF1, RUVBL1, PQBP-1

66	negative regulation of microtubule polymerization or depolymerization	39	2.6566E-05	9.7388E-04	2.6566E-05	9.7388E-04	4	Cyclin B1, Histone deacetylase class II, Calmyrin, Cyclin B
67	organic substance metabolic process	10476	2.7666E-05	9.9916E-04	2.7666E-05	9.9916E-04	66	FLJ22494, P5CR1, eEF1A, RBBP8 (CtIP), PGMU, IGHG4, Cyclin T1, RY1, Cyclin B1, DLC1 (Dynein LC8a), HSPA1B, PSMD10 (Gankyrin), TIPIN, Clp1, ECE1, AL1A1, Fbxo3, PPM1F, HDAC7, CDO1, HEXIM1, DARS, Histone deacetylase class II, Calmyrin, HSP70, IGHG1, Cyclophilin E, ARID4A, PGM2, eEF2K, LOC646817, SET, COASY, DPP3, TXNRD1, SMYD3, POLE3-POLE4 complex, Calmodulin, HSPA2, HSPA1A, RBP1, eEF1A1, SAE2, OBFC1, ZNF397OS, POLE3 (YBL1), DYNLL, ICMT, DOHH, ZNF397, SET/NUP214 fusion protein, Cyclin B, IMD2, EYA1, GMEB1, Pleckstrin, PTPN2, FOP, PDXP, Fructosamine-3-kinase, PP2C, HSC70, TARDBP (TDP43), TCOF1, RUVBL1, PQBP-1
68	cellular macromolecule metabolic process	7347	2.8833E-05	1.0226E-03	2.8833E-05	1.0226E-03	52	eEF1A, RBBP8 (CtIP), PGMU, Cyclin T1, RY1, Cyclin B1, DLC1 (Dynein LC8a), HSPA1B, PSMD10 (Gankyrin), TIPIN, Clp1, Fbxo3, PPM1F, HDAC7, HEXIM1, DARS, Histone deacetylase class II, Calmyrin, HSP70, Cyclophilin E, ARID4A, PGM2, eEF2K, LOC646817, SET, SMYD3, POLE3-POLE4 complex, Calmodulin, HSPA2, HSPA1A, eEF1A1, SAE2, OBFC1, ZNF397OS, POLE3 (YBL1), DYNLL, ICMT, DOHH, ZNF397, SET/NUP214 fusion protein, Cyclin B, EYA1, GMEB1, PTPN2, FOP, PDXP, PP2C, HSC70, TARDBP (TDP43), TCOF1, RUVBL1, PQBP-1
69	macromolecule metabolic process	8229	3.2133E-05	1.1227E-03	3.2133E-05	1.1227E-03	56	eEF1A, RBBP8 (CtIP), PGMU, IGHG4, Cyclin T1, RY1, Cyclin B1, DLC1 (Dynein LC8a), HSPA1B, PSMD10 (Gankyrin), TIPIN, Clp1, ECE1, Fbxo3, PPM1F, HDAC7, HEXIM1, DARS, Histone deacetylase class II, Calmyrin, HSP70, IGHG1, Cyclophilin E, ARID4A, PGM2, eEF2K, LOC646817, SET, DPP3, SMYD3, POLE3-POLE4 complex, Calmodulin, HSPA2, HSPA1A, eEF1A1, SAE2, OBFC1, ZNF397OS, POLE3 (YBL1), DYNLL, ICMT, DOHH, ZNF397, SET/NUP214 fusion protein, Cyclin B, EYA1, GMEB1, PTPN2, FOP, PDXP, PP2C, HSC70, TARDBP (TDP43), TCOF1, RUVBL1, PQBP-1
70	nucleic acid metabolic process	4435	3.296	1.139	3.296	1.139	37	eEF1A, RBBP8 (CtIP), Cyclin T1, RY1, DLC1 (Dynein LC8a), HSPA1B, PSMD10 (Gankyrin), TIPIN, Clp1, HDAC7, HEXIM1, DARS, Histone deacetylase class II, Calmyrin, HSP70, Cyclophilin E, ARID4A, LOC646817, SET, SMYD3, POLE3-POLE4 complex, HSPA1A, eEF1A1, OBFC1,

			E-05	E-03	E-05	E-03		ZNF397OS, POLE3 (YBL1), DYNLL, ZNF397, SET/NUP214 fusion protein, EYA1, GMEB1, PP2C, HSC70, TARDBP (TDP43), TCOF1, RUVBL1, PQBP-1
71	negative regulation of macromolecule metabolic process	2103	3.417E-05	1.165E-03	3.417E-05	1.165E-03	23	RBBP8 (CtIP), Cyclin B1, PSMD10 (Gankyrin), TIPIN, Clp1, PPM1F, HDAC7, HEXIM1, Histone deacetylase class II, Calmyrin, HSP70, ARID4A, SET, Follistatin, SMYD3, SET/NUP214 fusion protein, Cyclin B, PTPN2, FOP, CAMK2N1, PP2C, HSC70, TARDBP (TDP43)
72	regulation of cellular component organization	2107	3.522E-05	1.184E-03	3.522E-05	1.184E-03	23	Reticulon 4, Cyclin B1, DLC1 (Dynein LC8a), HSPA1B, PSMD10 (Gankyrin), PPM1F, Histone deacetylase class II, Olfactory receptor, Calmyrin, EPS8L1, HSP70, SET, HSPA2, HSPA1A, DYNLL, SET/NUP214 fusion protein, Cyclin B, Pleckstrin, FOP, PDXP, PP2C, HSC70, PQBP-1
73	negative regulation of vasoconstriction	15	3.706E-05	1.229E-03	3.706E-05	1.229E-03	3	HSPA1B, HSP70, HSPA1A
74	DNA metabolic process	1014	3.796E-05	1.233E-03	3.796E-05	1.233E-03	15	RBBP8 (CtIP), HSPA1B, TIPIN, Calmyrin, HSP70, LOC646817, SET, SMYD3, POLE3-POLE4 complex, HSPA1A, OBFC1, POLE3 (YBL1), SET/NUP214 fusion protein, EYA1, RUVBL1
75	regulation of phosphorylation	1539	3.821E-	1.233E-	3.821E-	1.233E-	19	Cyclin T1, Cyclin B1, DLC1 (Dynein LC8a), PSMD10 (Gankyrin), ECE1, PPM1F, HEXIM1, Calmyrin, HSP70, eEF2K, SMYD3, HSPA2, CKS2, DYNLL, Cyclin B, PTPN2, FOP, CAMK2N1, PP2C

			05	03	05	03		
76	regulation of protein kinase activity	896	3.931E-05	1.252E-03	3.931E-05	1.252E-03	14	Cyclin T1, Cyclin B1, PSMD10 (Gankyrin), PPM1F, HEXIM1, Calmyrin, HSP70, SMYD3, HSPA2, CKS2, Cyclin B, FOP, CAMK2N1, PP2C
77	peptidyl-lysine modification	209	4.070E-05	1.279E-03	4.070E-05	1.279E-03	7	Histone deacetylase class II, ARID4A, SMYD3, POLE3-POLE4 complex, POLE3 (YBL1), DOHH, RUVBL1
78	regulation of organelle organization	901	4.176E-05	1.296E-03	4.176E-05	1.296E-03	14	Cyclin B1, DLC1 (Dynein LC8a), PSMD10 (Gankyrin), PPM1F, Histone deacetylase class II, Olfactory receptor, Calmyrin, SET, DYNLL, SET/NUP214 fusion protein, Cyclin B, Pleckstrin, PDXP, PP2C
79	negative regulation of extrinsic apoptotic signaling pathway in absence of ligand	46	5.145E-05	1.557E-03	5.145E-05	1.557E-03	4	HSPA1B, HSP70, HSPA1A, EYA1
80	negative regulation of signal transduction in absence of ligand	46	5.145E-05	1.557E-03	5.145E-05	1.557E-03	4	HSPA1B, HSP70, HSPA1A, EYA1

81	cell recognition	149	5.354E-05	1.599E-03	5.354E-05	1.599E-03	6	Reticulon 4, HSPA1B, COLEC12, HSP70, HSPA1A, DYNLL
82	nucleobase-containing compound metabolic process	5491	5.806E-05	1.700E-03	5.806E-05	1.700E-03	42	FLJ22494, eEF1A, RBBP8 (CtIP), Cyclin T1, RY1, DLC1 (Dynein LC8a), HSPA1B, PSMD10 (Gankyrin), TIPIN, Clp1, HDAC7, HEXIM1, DARS, Histone deacetylase class II, Calmyrin, HSP70, Cyclophilin E, ARID4A, LOC646817, SET, COASY, TXNRD1, SMYD3, POLE3-POLE4 complex, HSPA1A, eEF1A1, OBFC1, ZNF397OS, POLE3 (YBL1), DYNLL, ICMT, ZNF397, SET/NUP214 fusion protein, IMD2, EYA1, GMEB1, PP2C, HSC70, TARDBP (TDP43), TCOF1, RUVBL1, PQBP-1
83	mitotic spindle stabilization	3	5.831E-05	1.700E-03	5.831E-05	1.700E-03	2	Cyclin B1, Cyclin B
84	regulation of system process	599	6.926E-05	1.995E-03	6.926E-05	1.995E-03	11	Reticulon 4, HSPA1B, ECE1, Histone deacetylase class II, Thioredoxin-like 2, HSP70, Annexin VI, Follistatin, Calmodulin, HSPA1A, PP2C
85	cellular aromatic compound metabolic process	5743	7.503E-05	2.136E-03	7.503E-05	2.136E-03	43	FLJ22494, eEF1A, RBBP8 (CtIP), Cyclin T1, RY1, DLC1 (Dynein LC8a), HSPA1B, PSMD10 (Gankyrin), TIPIN, Clp1, HDAC7, HEXIM1, DARS, Histone deacetylase class II, Calmyrin, HSP70, Cyclophilin E, ARID4A, LOC646817, SET, COASY, TXNRD1, SMYD3, POLE3-POLE4 complex, HSPA1A, eEF1A1, OBFC1, ZNF397OS, POLE3 (YBL1), DYNLL, ICMT, ZNF397, SET/NUP214 fusion protein, IMD2, EYA1, GMEB1, PDXP, PP2C, HSC70, TARDBP (TDP43), TCOF1, RUVBL1, PQBP-1
86	regulation of nucleobase-containing	49	7.	2.	7.	2.	39	eEF1A, RBBP8 (CtIP), Cyclin T1, Cyclin B1, DLC1 (Dynein LC8a), PSMD10 (Gankyrin), TIPIN, ECE1, AL1A1, PPM1F,

	compound metabolic process	86	960E-05	240E-03	960E-05	240E-03		HDAC7, HEXIM1, Histone deacetylase class II, Olfactory receptor, Calmyrin, EPS8L1, HSP70, Cyclophilin E, ARID4A, LOC646817, SET, Follistatin, SMYD3, Calmodulin, eEF1A1, OBFC1, ZNF397OS, DYNLL, ICMT, ZNF397, SET/NUP214 fusion protein, Cyclin B, EYA1, GMEB1, PP2C, HSC70, TARDBP (TDP43), RUVBL1, PQBP-1
87	negative regulation of cellular biosynthetic process	1494	8.682E-05	2.415E-03	8.682E-05	2.415E-03	18	RBBP8 (CtIP), DLC1 (Dynein LC8a), PSMD10 (Gankyrin), TIPIN, PPM1F, HDAC7, HEXIM1, Histone deacetylase class II, HSP70, ARID4A, SET, Follistatin, DYNLL, SET/NUP214 fusion protein, Pleckstrin, PP2C, HSC70, TARDBP (TDP43)
88	regulation of kinase activity	966	8.821E-05	2.426E-03	8.821E-05	2.426E-03	14	Cyclin T1, Cyclin B1, PSMD10 (Gankyrin), PPM1F, HEXIM1, Calmyrin, HSP70, SMYD3, HSPA2, CKS2, Cyclin B, FOP, CAMK2N1, PP2C
89	cellular component biogenesis	2239	9.059E-05	2.430E-03	9.059E-05	2.430E-03	23	Annexin V, eEF1A, Cyclin B1, PSMD10 (Gankyrin), Clp1, AL1A1, HDAC7, DARS, Histone deacetylase class II, COLEC12, HSP70, Annexin VI, LOC646817, SET, TXNRD1, SMYD3, eEF1A1, SET/NUP214 fusion protein, Cyclin B, IMD2, PDXP, RUVBL1, PQBP-1
90	positive regulation of cytoskeleton organization	164	9.112E-05	2.430E-03	9.112E-05	2.430E-03	6	Cyclin B1, PPM1F, Cyclin B, Pleckstrin, PDXP, PP2C
91	regulation of cell migration involved in sprouting angiogenesis	20	9.13	2.43	9.13	2.43	3	HDAC7, Histone deacetylase class II, Calmyrin

			8E-05	0E-03	8E-05	0E-03		
92	regulation of cellular biosynthetic process	4661	1.007E-04	2.623E-03	1.007E-04	2.623E-03	37	eEF1A, RBBP8 (CtIP), Cyclin T1, DLC1 (Dynein LC8a), PSMD10 (Gankyrin), TIPIN, ECE1, PPM1F, HDAC7, HEXIM1, Histone deacetylase class II, Olfactory receptor, Calmyrin, HSP70, Cyclophilin E, ARID4A, LOC646817, SET, Follistatin, SMYD3, Calmodulin, eEF1A1, OBFC1, ZNF397OS, DYNLL, ICMT, ZNF397, SET/NUP214 fusion protein, EYA1, GMEB1, Pleckstrin, PTPN2, PP2C, HSC70, TARDBP (TDP43), RUVBL1, PQBP-1
93	regulation of phosphate metabolic process	2257	1.024E-04	2.623E-03	1.024E-04	2.623E-03	23	Cyclin T1, Cyclin B1, DLC1 (Dynein LC8a), PSMD10 (Gankyrin), ECE1, AL1A1, PPM1F, HEXIM1, Calmyrin, EPS8L1, HSP70, eEF2K, SMYD3, Calmodulin, HSPA2, CKS2, DYNLL, Cyclin B, Pleckstrin, PTPN2, FOP, CAMK2N1, PP2C
94	organic cyclic compound metabolic process	6014	1.031E-04	2.623E-03	1.031E-04	2.623E-03	44	FLJ22494, P5CR1, eEF1A, RBBP8 (CtIP), Cyclin T1, RY1, DLC1 (Dynein LC8a), HSPA1B, PSMD10 (Gankyrin), TIPIN, Clp1, HDAC7, HEXIM1, DARS, Histone deacetylase class II, Calmyrin, HSP70, Cyclophilin E, ARID4A, LOC646817, SET, COASY, TXNRD1, SMYD3, POLE3-POLE4 complex, HSPA1A, eEF1A1, OBFC1, ZNF397OS, POLE3 (YBL1), DYNLL, ICMT, ZNF397, SET/NUP214 fusion protein, IMD2, EYA1, GMEB1, PDXP, PP2C, HSC70, TARDBP (TDP43), TCOF1, RUVBL1, PQBP-1
95	metabolic process	11562	1.039E-04	2.623E-03	1.039E-04	2.623E-03	69	FLJ22494, P5CR1, eEF1A, RBBP8 (CtIP), PGMU, IGHG4, Cyclin T1, RY1, Cyclin B1, DLC1 (Dynein LC8a), HSPA1B, PSMD10 (Gankyrin), TIPIN, Clp1, ECE1, AL1A1, Fbxo3, PPM1F, HDAC7, CDO1, Cytoglobin, HEXIM1, DARS, Histone deacetylase class II, Thioredoxin-like 2, Calmyrin, HSP70, IGHG1, Cyclophilin E, ARID4A, PGM2, eEF2K, LOC646817, SET, COASY, DPP3, TXNRD1, SMYD3, POLE3-POLE4 complex, Calmodulin, HSPA2, HSPA1A, RBP1, eEF1A1, SAE2, CKS2, OBFC1, ZNF397OS, POLE3 (YBL1), DYNLL, ICMT, DOHH, ZNF397, SET/NUP214 fusion protein, Cyclin B, IMD2, EYA1, GMEB1, Pleckstrin, PTPN2, FOP, PDXP, Fructosamine-3-kinase, PP2C, HSC70, TARDBP (TDP43), TCOF1, RUVBL1, PQBP-1
96	negative regulation of biological process	46	1.	2.	1.	2.	37	Annexin V, Reticulon 4, RBBP8 (CtIP), Cyclin B1, DLC1 (Dynein LC8a), HSPA1B, PSMD10 (Gankyrin), TIPIN, Clp1,

		68	041E-04	623E-03	041E-04	623E-03		PPM1F, HDAC7, HEXIM1, Histone deacetylase class II, Thioredoxin-like 2, Calmyrin, HSP70, ARID4A, CLEC2D, SET, Follistatin, SMYD3, Calmodulin, HSPA2, HSPA1A, DYNLL, SET/NUP214 fusion protein, Cyclin B, EYA1, Pleckstrin, PTPN2, FOP, RIG-G, CAMK2N1, PP2C, HSC70, TARDBP (TDP43), PRCC
97	positive regulation of epithelial cell migration	105	1.052E-04	2.623E-03	1.052E-04	2.623E-03	5	PPM1F, HDAC7, Histone deacetylase class II, Calmyrin, PP2C
98	positive regulation of cellular process	4675	1.075E-04	2.656E-03	1.075E-04	2.656E-03	37	Annexin V, eEF1A, Reticulon 4, Cyclin T1, Cyclin B1, DLC1 (Dynein LC8a), HSPA1B, PSMD10 (Gankyrin), TIPIN, ECE1, AL1A1, PPM1F, HDAC7, Histone deacetylase class II, Olfactory receptor, Calmyrin, EPS8L1, HSP70, Cyclophilin E, LOC646817, TXNRD1, SMYD3, Calmodulin, HSPA2, HSPA1A, eEF1A1, OBFC1, DYNLL, ICMT, Cyclin B, EYA1, Pleckstrin, PTPN2, FOP, PDXP, PP2C, TARDBP (TDP43)
99	regulation of phosphorus metabolic process	2275	1.155E-04	2.725E-03	1.155E-04	2.725E-03	23	Cyclin T1, Cyclin B1, DLC1 (Dynein LC8a), PSMD10 (Gankyrin), ECE1, AL1A1, PPM1F, HEXIM1, Calmyrin, EPS8L1, HSP70, eEF2K, SMYD3, Calmodulin, HSPA2, CKS2, DYNLL, Cyclin B, Pleckstrin, PTPN2, FOP, CAMK2N1, PP2C
100	cellular response to iron(III) ion	4	1.163E-04	2.725E-03	1.163E-04	2.725E-03	2	Cyclin B1, Cyclin B
101	positive regulation of cyclin-dependent protein serine/threonine kinase activity	4	1.16	2.72	1.16	2.72	2	HSP70, HSPA2

	involved in G2/M transition of mitotic cell cycle		3E-04	5E-03	3E-04	5E-03		
102	clathrin coat disassembly	4	1.163E-04	2.725E-03	1.163E-04	2.725E-03	2	HSP70, HSC70
103	regulation of cyclin-dependent protein serine/threonine kinase activity involved in G2/M transition of mitotic cell cycle	4	1.163E-04	2.725E-03	1.163E-04	2.725E-03	2	HSP70, HSPA2
104	regulation of cellular metabolic process	6446	1.171E-04	2.725E-03	1.171E-04	2.725E-03	46	eEF1A, RBBP8 (CtIP), Cyclin T1, Cyclin B1, DLC1 (Dynein LC8a), PSMD10 (Gankyrin), TIPIN, ECE1, AL1A1, PPM1F, HDAC7, HEXIM1, Histone deacetylase class II, Olfactory receptor, Calmyrin, EPS8L1, HSP70, Cyclophilin E, ARID4A, eEF2K, LOC646817, SET, Follistatin, SMYD3, Calmodulin, HSPA2, eEF1A1, CKS2, OBFC1, ZNF397OS, DYNLL, ICMT, ZNF397, SET/NUP214 fusion protein, Cyclin B, EYA1, GMEB1, Pleckstrin, PTPN2, FOP, CAMK2N1, PP2C, HSC70, TARDBP (TDP43), RUVBL1, PQBP-1
105	cellular protein metabolic process	3617	1.233E-04	2.812E-03	1.233E-04	2.812E-03	31	eEF1A, Cyclin T1, Cyclin B1, HSPA1B, PSMD10 (Gankyrin), Fbxo3, PPM1F, HDAC7, DARS, Histone deacetylase class II, HSP70, Cyclophilin E, ARID4A, eEF2K, SMYD3, POLE3-POLE4 complex, HSPA2, HSPA1A, eEF1A1, SAE2, POLE3 (YBL1), ICMT, DOHH, Cyclin B, EYA1, PTPN2, FOP, PDXP, PP2C, HSC70, RUVBL1
106	single-organism process	15116	1.240	2.812	1.240	2.812	82	Annexin V, FLJ22494, P5CR1, eEF1A, Reticulon 4, RBBP8 (CtIP), PGMU, IGHG4, Cyclin T1, Cyclin B1, DLC1 (Dynein LC8a), HSPA1B, PSMD10 (Gankyrin), TIPIN, Clp1, ECE1, AL1A1, ARL5B, PPM1F, Beta crystallin B2, HDAC7, MENA, CDO1, Cytoglobin, SNX1, HEXIM1, DARS,

			E-04	E-03	E-04	E-03		VAMP5, Histone deacetylase class II, Olfactory receptor, Thioredoxin-like 2, Calmyrin, EPS8L1, COLEC12, HSP70, IGHG1, ARID4A, Annexin VI, PGM2, CLEC2D, eEF2K, LOC646817, SET, COASY, Follistatin, TXNRD1, SMYD3, POLE3-POLE4 complex, Calmodulin, HSPA2, HSPA1A, RBP1, eEF1A1, CKS2, OR10G3, OBFC1, Annexin XI, POLE3 (YBL1), DYNLL, ICMT, DOHH, Septin 3, SET/NUP214 fusion protein, Cyclin B, IMD2, EYA1, Pleckstrin, PTPN2, FOP, RIG-G, PDXP, CAMK2N1, Fructosamine-3-kinase, PP2C, HSC70, TARDBP (TDP43), PRCC, TCOF1, RUVBL1, CapZIP, PQBP-1, VILIP1
107	negative regulation of biosynthetic process	1538	1.255E-04	2.812E-03	1.255E-04	2.812E-03	18	RBBP8 (CtIP), DLC1 (Dynein LC8a), PSMD10 (Gankyrin), TIPIN, PPM1F, HDAC7, HEXIM1, Histone deacetylase class II, HSP70, ARID4A, SET, Follistatin, DYNLL, SET/NUP214 fusion protein, Pleckstrin, PP2C, HSC70, TARDBP (TDP43)
108	response to stress	4156	1.258E-04	2.812E-03	1.258E-04	2.812E-03	34	Annexin V, IGHM, P5CR1, RBBP8 (CtIP), IGHG4, Cyclin B1, HSPA1B, PSMD10 (Gankyrin), TIPIN, ECE1, AL1A1, HDAC7, CDO1, Cytoglobin, Histone deacetylase class II, Olfactory receptor, Calmyrin, COLEC12, HSP70, IGHG1, TXNRD1, Calmodulin, HSPA2, HSPA1A, Cyclin B, EYA1, Pleckstrin, PTPN2, RIG-G, PP2C, HSC70, TARDBP (TDP43), RUVBL1, CapZIP
109	regulation of cytoskeleton organization	431	1.266E-04	2.812E-03	1.266E-04	2.812E-03	9	Cyclin B1, PPM1F, Histone deacetylase class II, Olfactory receptor, Calmyrin, Cyclin B, Pleckstrin, PDXP, PP2C
110	regulation of protein phosphorylation	1267	1.329E-04	2.925E-03	1.329E-04	2.925E-03	16	Cyclin T1, Cyclin B1, PSMD10 (Gankyrin), PPM1F, HEXIM1, Calmyrin, HSP70, eEF2K, SMYD3, HSPA2, CKS2, Cyclin B, PTPN2, FOP, CAMK2N1, PP2C

111	negative regulation of myeloid cell apoptotic process	23	1.406E-04	3.049E-03	1.406E-04	3.049E-03	3	HSPA1B, HSP70, HSPA1A
112	regulation of nitrogen compound metabolic process	5112	1.411E-04	3.049E-03	1.411E-04	3.049E-03	39	eEF1A, RBBP8 (CtIP), Cyclin T1, Cyclin B1, DLC1 (Dynein LC8a), PSMD10 (Gankyrin), TIPIN, ECE1, AL1A1, PPM1F, HDAC7, HEXIM1, Histone deacetylase class II, Olfactory receptor, Calmyrin, EPS8L1, HSP70, Cyclophilin E, ARID4A, LOC646817, SET, Follistatin, SMYD3, Calmodulin, eEF1A1, OBFC1, ZNF397OS, DYNLL, ICMT, ZNF397, SET/NUP214 fusion protein, Cyclin B, EYA1, GMEB1, PP2C, HSC70, TARDBP (TDP43), RUVBL1, PQBP-1
113	regulation of biosynthetic process	4739	1.447E-04	3.099E-03	1.447E-04	3.099E-03	37	eEF1A, RBBP8 (CtIP), Cyclin T1, DLC1 (Dynein LC8a), PSMD10 (Gankyrin), TIPIN, ECE1, PPM1F, HDAC7, HEXIM1, Histone deacetylase class II, Olfactory receptor, Calmyrin, HSP70, Cyclophilin E, ARID4A, LOC646817, SET, Follistatin, SMYD3, Calmodulin, eEF1A1, OBFC1, ZNF397OS, DYNLL, ICMT, ZNF397, SET/NUP214 fusion protein, EYA1, GMEB1, Pleckstrin, PTPN2, PP2C, HSC70, TARDBP (TDP43), RUVBL1, PQBP-1
114	regulation of myeloid cell differentiation	257	1.485E-04	3.153E-03	1.485E-04	3.153E-03	7	HSPA1B, Calmyrin, HSP70, CLEC2D, HSPA1A, RBP1, PTPN2
115	cell division	772	1.547E-04	3.256E-03	1.547E-04	3.256E-03	12	RBBP8 (CtIP), Cyclin T1, Cyclin B1, TIPIN, Calmyrin, HSP70, HSPA2, CKS2, Annexin XI, Septin 3, Cyclin B, RUVBL1
11	response to topologically incorrect protein	18	1.	3.	1.	3.	6	HSPA1B, Histone deacetylase class II, HSP70, HSPA2, HSPA1A, HSC70

6		3	660E-04	464E-03	660E-04	464E-03		
117	regulation of protein serine/threonine kinase activity	553	1.711E-04	3.539E-03	1.711E-04	3.539E-03	10	Cyclin T1, Cyclin B1, PSMD10 (Gankyrin), HEXIM1, Calmyrin, HSP70, HSPA2, CKS2, Cyclin B, PP2C
118	glycogen catabolic process	25	1.814E-04	3.721E-03	1.814E-04	3.721E-03	3	PGMU, PGM2, Calmodulin
119	negative regulation of nitrogen compound metabolic process	1446	1.889E-04	3.842E-03	1.889E-04	3.842E-03	17	RBBP8 (CtIP), DLC1 (Dynein LC8a), PSMD10 (Gankyrin), TIPIN, PPM1F, HDAC7, HEXIM1, Histone deacetylase class II, HSP70, ARID4A, SET, Follistatin, DYNLL, SET/NUP214 fusion protein, PP2C, HSC70, TARDBP (TDP43)
120	cellular biosynthetic process	4991	1.920E-04	3.865E-03	1.920E-04	3.865E-03	38	P5CR1, eEF1A, RBBP8 (CtIP), PGMU, Cyclin T1, DLC1 (Dynein LC8a), TIPIN, Clp1, AL1A1, HDAC7, CDO1, HEXIM1, DARS, Histone deacetylase class II, HSP70, ARID4A, PGM2, eEF2K, LOC646817, SET, COASY, POLE3-POLE4 complex, RBP1, eEF1A1, ZNF397OS, POLE3 (YBL1), DYNLL, ZNF397, SET/NUP214 fusion protein, IMD2, EYA1, GMEB1, PP2C, HSC70, TARDBP (TDP43), TCOF1, RUVBL1, PQBP-1
121	spindle stabilization	5	1.93	3.86	1.93	3.86	2	Cyclin B1, Cyclin B

			3E-04	5E-03	3E-04	5E-03		
122	regulation of microtubule polymerization or depolymerization	65	2.002E-04	3.960E-03	2.002E-04	3.960E-03	4	Cyclin B1, Histone deacetylase class II, Calmyrin, Cyclin B
123	regulation of protein metabolic process	2522	2.016E-04	3.960E-03	2.016E-04	3.960E-03	24	Cyclin T1, Cyclin B1, HSPA1B, PSMD10 (Gankyrin), ECE1, PPM1F, SNX1, HEXIM1, Histone deacetylase class II, Calmyrin, HSP70, eEF2K, SET, SMYD3, Calmodulin, HSPA2, HSPA1A, CKS2, SET/NUP214 fusion protein, Cyclin B, PTPN2, FOP, CAMK2N1, PP2C
124	glucan catabolic process	26	2.044E-04	3.960E-03	2.044E-04	3.960E-03	3	PGMU, PGM2, Calmodulin
125	regulation of RNA metabolic process	4075	2.046E-04	3.960E-03	2.046E-04	3.960E-03	33	eEF1A, RBBP8 (CtIP), Cyclin T1, Cyclin B1, DLC1 (Dynein LC8a), PSMD10 (Gankyrin), PPM1F, HDAC7, HEXIM1, Histone deacetylase class II, Olfactory receptor, Calmyrin, HSP70, Cyclophilin E, ARID4A, LOC646817, SET, Follistatin, SMYD3, eEF1A1, ZNF397OS, DYNLL, ICMT, ZNF397, SET/NUP214 fusion protein, Cyclin B, EYA1, GMEB1, PP2C, HSC70, TARDBP (TDP43), RUVBL1, PQBP-1
126	regulation of cell death	1899	2.086E	4.006E	2.086E	4.006E	20	Annexin V, eEF1A, Reticulon 4, DLC1 (Dynein LC8a), HSPA1B, PSMD10 (Gankyrin), AL1A1, PPM1F, Histone deacetylase class II, Calmyrin, HSP70, SET, TXNRD1, HSPA1A, eEF1A1, DYNLL, SET/NUP214 fusion protein, EYA1, RIG-G, PP2C

			-04	-03	-04	-03		
127	phosphate-containing compound metabolic process	2859	2.108E-04	4.016E-03	2.108E-04	4.016E-03	26	FLJ22494, eEF1A, Cyclin T1, Cyclin B1, HSPA1B, Clp1, PPM1F, HSP70, PGM2, eEF2K, COASY, TXNRD1, Calmodulin, HSPA1A, eEF1A1, CKS2, Cyclin B, IMD2, EYA1, Pleckstrin, PTPN2, FOP, PDXP, Fructosamine-3-kinase, PP2C, HSC70
128	regulation of growth	802	2.200E-04	4.159E-03	2.200E-04	4.159E-03	12	Reticulon 4, Cyclin B1, HSPA1B, PSMD10 (Gankyrin), PPM1F, Calmyrin, HSP70, HSPA1A, Cyclin B, FOP, PP2C, RUVBL1
129	cell cycle checkpoint	275	2.249E-04	4.219E-03	2.249E-04	4.219E-03	7	RBBP8 (CtIP), Cyclin B1, PSMD10 (Gankyrin), TIPIN, Cyclin B, PP2C, PRCC
130	cellular polysaccharide catabolic process	27	2.292E-04	4.267E-03	2.292E-04	4.267E-03	3	PGMU, PGM2, Calmodulin
131	RNA metabolic process	3747	2.387E-0	4.409E-0	2.387E-0	4.409E-0	31	eEF1A, RBBP8 (CtIP), Cyclin T1, RY1, DLC1 (Dynein LC8a), HSPA1B, PSMD10 (Gankyrin), Clp1, HDAC7, HEXIM1, DARS, Histone deacetylase class II, HSP70, Cyclophilin E, ARID4A, LOC646817, SET, HSPA1A, eEF1A1, ZNF397OS, DYNLL, ZNF397, SET/NUP214 fusion protein, EYA1, GMEB1, PP2C, HSC70, TARDBP (TDP43), TCOF1, RUVBL1, PQBP-1

			4	3	4	3		
132	negative regulation of protein depolymerization	71	2.815E-04	5.091E-03	2.815E-04	5.091E-03	4	Cyclin B1, Histone deacetylase class II, Calmyrin, Cyclin B
133	regulation of apoptotic process	1794	2.889E-04	5.091E-03	2.889E-04	5.091E-03	19	Annexin V, eEF1A, Reticulon 4, DLC1 (Dynein LC8a), HSPA1B, PSMD10 (Gankyrin), AL1A1, PPM1F, Histone deacetylase class II, Calmyrin, HSP70, SET, HSPA1A, eEF1A1, DYNLL, SET/NUP214 fusion protein, EYA1, RIG-G, PP2C
134	positive regulation of attachment of spindle microtubules to kinetochore	6	2.890E-04	5.091E-03	2.890E-04	5.091E-03	2	Cyclin B1, Cyclin B
135	regulation of chromosome condensation	6	2.890E-04	5.091E-03	2.890E-04	5.091E-03	2	Cyclin B1, Cyclin B
136	vesicle uncoating	6	2.890E-04	5.091E-03	2.890E-04	5.091E-03	2	HSP70, HSC70

137	protein metabolic process	4519	2.902E-04	5.091E-03	2.902E-04	5.091E-03	35	eEF1A, IGHG4, Cyclin T1, Cyclin B1, HSPA1B, PSMD10 (Gankyrin), ECE1, Fbxo3, PPM1F, HDAC7, DARS, Histone deacetylase class II, HSP70, IGHG1, Cyclophilin E, ARID4A, eEF2K, DPP3, SMYD3, POLE3-POLE4 complex, HSPA2, HSPA1A, eEF1A1, SAE2, POLE3 (YBL1), ICMT, DOHH, Cyclin B, EYA1, PTPN2, FOP, PDXP, PP2C, HSC70, RUVBL1
138	organic substance biosynthetic process	5087	2.903E-04	5.091E-03	2.903E-04	5.091E-03	38	P5CR1, eEF1A, RBBP8 (CtIP), PGMU, Cyclin T1, DLC1 (Dynein LC8a), TIPIN, Clp1, AL1A1, HDAC7, CDO1, HEXIM1, DARS, Histone deacetylase class II, HSP70, ARID4A, PGM2, eEF2K, LOC646817, SET, COASY, POLE3-POLE4 complex, RBP1, eEF1A1, ZNF397OS, POLE3 (YBL1), DYNLL, ZNF397, SET/NUP214 fusion protein, IMD2, EYA1, GMEB1, PP2C, HSC70, TARDBP (TDP43), TCOF1, RUVBL1, PQBP-1
139	phosphorus metabolic process	2922	2.993E-04	5.205E-03	2.993E-04	5.205E-03	26	FLJ22494, eEF1A, Cyclin T1, Cyclin B1, HSPA1B, Clp1, PPM1F, HSP70, PGM2, eEF2K, COASY, TXNRD1, Calmodulin, HSPA1A, eEF1A1, CKS2, Cyclin B, IMD2, EYA1, Pleckstrin, PTPN2, FOP, PDXP, Fructosamine-3-kinase, PP2C, HSC70
140	cellular process	16599	3.011E-04	5.205E-03	3.011E-04	5.205E-03	86	Annexin V, FLJ22494, P5CR1, eEF1A, Reticulon 4, RBBP8 (CtIP), PGMU, IGHG4, Cyclin T1, RY1, Cyclin B1, DLC1 (Dynein LC8a), HSPA1B, PSMD10 (Gankyrin), TIPIN, Clp1, ECE1, AL1A1, ARL5B, Fbxo3, PPM1F, HDAC7, MENA, CDO1, HEXIM1, DARS, VAMP5, Histone deacetylase class II, Olfactory receptor, Thioredoxin-like 2, Calmyrin, EPS8L1, COLEC12, HSP70, IGHG1, Cyclophilin E, ARID4A, Annexin VI, PGM2, CLEC2D, eEF2K, LOC646817, SET, COASY, Follistatin, TXNRD1, SMYD3, POLE3-POLE4 complex, Calmodulin, HSPA2, HSPA1A, RBP1, eEF1A1, SAE2, CKS2, OR10G3, OBFC1, ZNF397OS, Annexin XI, POLE3 (YBL1), DYNLL, ICMT, DOHH, ZNF397, Septin 3, SET/NUP214 fusion protein, Cyclin B, IMD2, EYA1, GMEB1, Pleckstrin, PTPN2, FOP, RIG-G, PDXP, CAMK2N1, Fructosamine-3-kinase, PP2C, HSC70, TARDBP (TDP43), PRCC, TCOF1, RUVBL1, CapZIP, PQBP-1, VILIP1
141	polysaccharide catabolic process	30	3.15	5.37	3.15	5.37	3	PGMU, PGM2, Calmodulin

			1E-04	1E-03	1E-04	1E-03		
142	positive regulation of cyclin-dependent protein kinase activity	30	3.151E-04	5.371E-03	3.151E-04	5.371E-03	3	PSMD10 (Gankyrin), HSP70, HSPA2
143	regulation of programmed cell death	1811	3.255E-04	5.492E-03	3.255E-04	5.492E-03	19	Annexin V, eEF1A, Reticulon 4, DLC1 (Dynein LC8a), HSPA1B, PSMD10 (Gankyrin), AL1A1, PPM1F, Histone deacetylase class II, Calmyrin, HSP70, SET, HSPA1A, eEF1A1, DYNLL, SET/NUP214 fusion protein, EYA1, RIG-G, PP2C
144	substantia nigra development	74	3.300E-04	5.492E-03	3.300E-04	5.492E-03	4	DLC1 (Dynein LC8a), HSP70, Calmodulin, DYNLL
145	negative regulation of protein complex disassembly	74	3.300E-04	5.492E-03	3.300E-04	5.492E-03	4	Cyclin B1, Histone deacetylase class II, Calmyrin, Cyclin B
146	regulation of cellular protein metabolic process	1968	3.344E	5.492E	3.344E	5.492E	20	Cyclin T1, Cyclin B1, PSMD10 (Gankyrin), PPM1F, HEXIM1, Histone deacetylase class II, Calmyrin, HSP70, eEF2K, SET, SMYD3, Calmodulin, HSPA2, CKS2, SET/NUP214 fusion protein, Cyclin B, PTPN2, FOP, CAMK2N1, PP2C

			-04	-03	-04	-03		
147	mRNA metabolic process	718	3.354E-04	5.492E-03	3.354E-04	5.492E-03	11	RY1, HSPA1B, PSMD10 (Gankyrin), Clp1, HSP70, Cyclophilin E, SET, HSPA1A, SET/NUP214 fusion protein, HSC70, TARDBP (TDP43)
148	regulation of transferase activity	1099	3.359E-04	5.492E-03	3.359E-04	5.492E-03	14	Cyclin T1, Cyclin B1, PSMD10 (Gankyrin), PPM1F, HEXIM1, Calmyrin, HSP70, SMYD3, HSPA2, CKS2, Cyclin B, FOP, CAMK2N1, PP2C
149	cellular macromolecule biosynthetic process	4001	3.399E-04	5.521E-03	3.399E-04	5.521E-03	32	eEF1A, RBBP8 (CtIP), PGMU, Cyclin T1, DLC1 (Dynein LC8a), TIPIN, Clp1, HDAC7, HEXIM1, DARS, Histone deacetylase class II, HSP70, ARID4A, PGM2, eEF2K, LOC646817, SET, POLE3-POLE4 complex, eEF1A1, ZNF397OS, POLE3 (YBL1), DYNLL, ZNF397, SET/NUP214 fusion protein, EYA1, GMEB1, PP2C, HSC70, TARDBP (TDP43), TCOF1, RUVBL1, PQBP-1
150	negative regulation of organelle organization	297	3.583E-04	5.781E-03	3.583E-04	5.781E-03	7	Cyclin B1, PSMD10 (Gankyrin), Histone deacetylase class II, Calmyrin, SET, SET/NUP214 fusion protein, Cyclin B
151	positive regulation of organelle organization	394	3.681E-0	5.900E-0	3.681E-0	5.900E-0	8	Cyclin B1, DLC1 (Dynein LC8a), PPM1F, DYNLL, Cyclin B, Pleckstrin, PDXP, PP2C

			4	3	4	3		
152	regulation of molecular function	3133	3.710E-04	5.907E-03	3.710E-04	5.907E-03	27	Annexin V, Cyclin T1, Cyclin B1, DLC1 (Dynein LC8a), HSPA1B, PSMD10 (Gankyrin), AL1A1, PPM1F, HEXIM1, Histone deacetylase class II, Calmyrin, EPS8L1, HSP70, SET, SMYD3, Calmodulin, HSPA2, HSPA1A, SAE2, CKS2, DYNLL, SET/NUP214 fusion protein, Cyclin B, Pleckstrin, FOP, CAMK2N1, PP2C
153	negative regulation of gene expression	1390	3.793E-04	5.947E-03	3.793E-04	5.947E-03	16	RBBP8 (CtIP), Cyclin B1, PSMD10 (Gankyrin), Clp1, PPM1F, HDAC7, HEXIM1, Histone deacetylase class II, HSP70, ARID4A, SET, Follistatin, SET/NUP214 fusion protein, Cyclin B, PP2C, HSC70
154	gene expression	4210	3.828E-04	5.947E-03	3.828E-04	5.947E-03	33	eEF1A, RBBP8 (CtIP), Cyclin T1, RY1, DLC1 (Dynein LC8a), HSPA1B, PSMD10 (Gankyrin), Clp1, ECE1, HDAC7, HEXIM1, DARS, Histone deacetylase class II, HSP70, Cyclophilin E, ARID4A, eEF2K, LOC646817, SET, HSPA1A, eEF1A1, ZNF397OS, DYNLL, ZNF397, SET/NUP214 fusion protein, EYA1, GMEB1, PP2C, HSC70, TARDBP (TDP43), TCOF1, RUVBL1, PQBP-1
155	subthalamus development	77	3.842E-04	5.947E-03	3.842E-04	5.947E-03	4	DLC1 (Dynein LC8a), HSP70, Calmodulin, DYNLL
156	regulation of extrinsic apoptotic signaling pathway in absence of ligand	77	3.842E-04	5.947E-03	3.842E-04	5.947E-03	4	HSPA1B, HSP70, HSPA1A, EYA1

157	single-organism cellular process	13479	3.874E-04	5.947E-03	3.874E-04	5.947E-03	75	Annexin V, FLJ22494, P5CR1, eEF1A, Reticulon 4, RBBP8 (CtIP), PGMU, IGHG4, Cyclin T1, Cyclin B1, DLC1 (Dynein LC8a), HSPA1B, PSMD10 (Gankyrin), TIPIN, Clp1, ECE1, AL1A1, ARL5B, PPM1F, HDAC7, MENA, CDO1, DARS, VAMP5, Histone deacetylase class II, Olfactory receptor, Thioredoxin-like 2, Calmyrin, EPS8L1, COLEC12, HSP70, IGHG1, ARID4A, Annexin VI, PGM2, CLEC2D, eEF2K, LOC646817, SET, COASY, Follistatin, TXNRD1, SMYD3, POLE3-POLE4 complex, Calmodulin, HSPA2, HSPA1A, RBP1, eEF1A1, CKS2, OR10G3, OBFC1, Annexin XI, POLE3 (YBL1), DYNLL, ICMT, Septin 3, SET/NUP214 fusion protein, Cyclin B, IMD2, EYA1, Pleckstrin, PTPN2, FOP, RIG-G, PDXP, CAMK2N1, Fructosamine-3-kinase, PP2C, HSC70, TARDBP (TDP43), PRCC, RUVBL1, PQBP-1, VILIP1
158	protein homooligomerization	301	3.883E-04	5.947E-03	3.883E-04	5.947E-03	7	Annexin V, eEF1A, AL1A1, COLEC12, Annexin VI, eEF1A1, IMD2
159	response to iron(III) ion	7	4.035E-04	6.065E-03	4.035E-04	6.065E-03	2	Cyclin B1, Cyclin B
160	positive regulation of chromosome segregation	7	4.035E-04	6.065E-03	4.035E-04	6.065E-03	2	Cyclin B1, Cyclin B
161	negative regulation of protein kinase activity by regulation of protein phosphorylation	7	4.035E	6.065E	4.035E	6.065E	2	PPM1F, PP2C

			-04	-03	-04	-03		
162	biosynthetic process	5178	4.238E-04	6.331E-03	4.238E-04	6.331E-03	38	P5CR1, eEF1A, RBBP8 (CtIP), PGMU, Cyclin T1, DLC1 (Dynein LC8a), TIPIN, Clp1, AL1A1, HDAC7, CDO1, HEXIM1, DARS, Histone deacetylase class II, HSP70, ARID4A, PGM2, eEF2K, LOC646817, SET, COASY, POLE3-POLE4 complex, RBP1, eEF1A1, ZNF397OS, POLE3 (YBL1), DYNLL, ZNF397, SET/NUP214 fusion protein, IMD2, EYA1, GMEB1, PP2C, HSC70, TARDBP (TDP43), TCOF1, RUVBL1, PQBP-1
163	nucleosome assembly	142	4.290E-04	6.369E-03	4.290E-04	6.369E-03	5	LOC646817, SET, SMYD3, SET/NUP214 fusion protein, RUVBL1
164	telomere maintenance	80	4.445E-04	6.535E-03	4.445E-04	6.535E-03	4	HSPA1B, HSP70, HSPA1A, OBFC1
165	macromolecule biosynthetic process	4060	4.456E-04	6.535E-03	4.456E-04	6.535E-03	32	eEF1A, RBBP8 (CtIP), PGMU, Cyclin T1, DLC1 (Dynein LC8a), TIPIN, Clp1, HDAC7, HEXIM1, DARS, Histone deacetylase class II, HSP70, ARID4A, PGM2, eEF2K, LOC646817, SET, POLE3-POLE4 complex, eEF1A1, ZNF397OS, POLE3 (YBL1), DYNLL, ZNF397, SET/NUP214 fusion protein, EYA1, GMEB1, PP2C, HSC70, TARDBP (TDP43), TCOF1, RUVBL1, PQBP-1
166	negative regulation of cellular process	4254	4.657E-0	6.753E-0	4.657E-0	6.753E-0	33	Annexin V, Reticulon 4, RBBP8 (CtIP), Cyclin B1, DLC1 (Dynein LC8a), HSPA1B, PSMD10 (Gankyrin), TIPIN, PPM1F, HDAC7, HEXIM1, Histone deacetylase class II, Calmyrin, HSP70, ARID4A, CLEC2D, SET, Follistatin, SMYD3, HSPA1A, DYNLL, SET/NUP214 fusion protein, Cyclin B, EYA1, Pleckstrin, PTPN2, FOP, RIG-G, CAMK2N1, PP2C, HSC70, TARDBP (TDP43), PRCC

			4	3	4	3		
167	telomere organization	81	4.660E-04	6.753E-03	4.660E-04	6.753E-03	4	HSPA1B, HSP70, HSPA1A, OBFC1
168	positive regulation of blood vessel endothelial cell migration	35	4.999E-04	7.201E-03	4.999E-04	7.201E-03	3	HDAC7, Histone deacetylase class II, Calmyrin
169	galactose catabolic process	8	5.364E-04	7.636E-03	5.364E-04	7.636E-03	2	PGMU, PGM2
170	positive regulation of histone phosphorylation	8	5.364E-04	7.636E-03	5.364E-04	7.636E-03	2	Cyclin B1, Cyclin B
171	positive regulation of erythrocyte differentiation	36	5.436E-04	7.693E-03	5.436E-04	7.693E-03	3	HSPA1B, HSP70, HSPA1A

172	cellular carbohydrate catabolic process	37	5.897E-04	8.249E-03	5.897E-04	8.249E-03	3	PGMU, PGM2, Calmodulin
173	nitric oxide metabolic process	37	5.897E-04	8.249E-03	5.897E-04	8.249E-03	3	DLC1 (Dynein LC8a), Calmodulin, DYNLL
174	chromatin assembly	156	6.582E-04	9.154E-03	6.582E-04	9.154E-03	5	LOC646817, SET, SMYD3, SET/NUP214 fusion protein, RUVBL1
175	regulation of RNA biosynthetic process	3980	7.062E-04	9.765E-03	7.062E-04	9.765E-03	31	eEF1A, RBBP8 (CtIP), Cyclin T1, DLC1 (Dynein LC8a), PSMD10 (Gankyrin), PPM1F, HDAC7, HEXIM1, Histone deacetylase class II, Olfactory receptor, Calmyrin, HSP70, Cyclophilin E, ARID4A, LOC646817, SET, Follistatin, SMYD3, eEF1A1, ZNF397OS, DYNLL, ICMT, ZNF397, SET/NUP214 fusion protein, EYA1, GMEB1, PP2C, HSC70, TARDBP (TDP43), RUVBL1, PQBP-1
176	regulation of myeloid cell apoptotic process	41	7.987E-04	1.097E-02	7.987E-04	1.097E-02	3	HSPA1B, HSP70, HSPA1A
17	protein complex disassembly	16	8.	1.	8.	1.	5	Histone deacetylase class II, HSP70, SET, SET/NUP214 fusion protein, HSC70

7		3	023E-04	097E-02	023E-04	097E-02		
178	regulation of mitotic spindle organization	10	8.571E-04	1.140E-02	8.571E-04	1.140E-02	2	Cyclin B1, Cyclin B
179	retinoic acid biosynthetic process	10	8.571E-04	1.140E-02	8.571E-04	1.140E-02	2	AL1A1, RBP1
180	peptidyl-threonine dephosphorylation	10	8.571E-04	1.140E-02	8.571E-04	1.140E-02	2	PPM1F, PP2C
181	positive regulation of actin filament depolymerization	10	8.571E-04	1.140E-02	8.571E-04	1.140E-02	2	Pleckstrin, PDXP
182	mitotic nuclear envelope disassembly	42	8.57	1.14	8.57	1.14	3	Cyclin B1, SET/NUP214 fusion protein, Cyclin B

			4E-04	0E-02	4E-04	0E-02		
183	cellular response to stimulus	7628	9.223E-04	1.218E-02	9.223E-04	1.218E-02	49	Annexin V, P5CR1, eEF1A, Reticulon 4, RBBP8 (CtIP), IGHG4, Cyclin T1, Cyclin B1, DLC1 (Dynein LC8a), HSPA1B, PSMD10 (Gankyrin), TIPIN, AL1A1, ARL5B, PPM1F, HDAC7, MENA, Histone deacetylase class II, Olfactory receptor, Calmyrin, EPS8L1, COLEC12, HSP70, IGHG1, CLEC2D, eEF2K, Follistatin, TXNRD1, SMYD3, Calmodulin, HSPA1A, RBP1, eEF1A1, CKS2, OR10G3, DYNLL, SET/NUP214 fusion protein, Cyclin B, IMD2, EYA1, Pleckstrin, PTPN2, RIG-G, PDXP, PP2C, TARDBP (TDP43), RUVBL1, CapZIP, VILIP1
184	negative regulation of protein kinase activity	253	9.264E-04	1.218E-02	9.264E-04	1.218E-02	6	PPM1F, HEXIM1, SMYD3, FOP, CAMK2N1, PP2C
185	response to unfolded protein	170	9.687E-04	1.265E-02	9.687E-04	1.265E-02	5	HSPA1B, HSP70, HSPA2, HSPA1A, HSC70
186	nuclear envelope disassembly	44	9.828E-04	1.265E-02	9.828E-04	1.265E-02	3	Cyclin B1, SET/NUP214 fusion protein, Cyclin B
187	membrane disassembly	44	9.828	1.265	9.828	1.265	3	Cyclin B1, SET/NUP214 fusion protein, Cyclin B

			E-04	E-02	E-04	E-02		
188	positive regulation of cell-matrix adhesion	44	9.828E-04	1.265E-02	9.828E-04	1.265E-02	3	PPM1F, Calmyrin, PP2C
189	negative regulation of RNA biosynthetic process	1226	9.890E-04	1.266E-02	9.890E-04	1.266E-02	14	RBBP8 (CtIP), PSMD10 (Gankyrin), PPM1F, HDAC7, HEXIM1, Histone deacetylase class II, HSP70, ARID4A, SET, Follistatin, SET/NUP214 fusion protein, PP2C, HSC70, TARDBP (TDP43)
190	regulation of vasoconstriction	100	1.030E-03	1.307E-02	1.030E-03	1.307E-02	4	HSPA1B, ECE1, HSP70, HSPA1A
191	regulation of histone phosphorylation	11	1.045E-03	1.307E-02	1.045E-03	1.307E-02	2	Cyclin B1, Cyclin B
192	cellular response to iron ion	11	1.045E-	1.307E-	1.045E-	1.307E-	2	Cyclin B1, Cyclin B

			03	02	03	02		
193	diterpenoid biosynthetic process	11	1.045E-03	1.307E-02	1.045E-03	1.307E-02	2	AL1A1, RBP1
194	nucleosome organization	173	1.047E-03	1.307E-02	1.047E-03	1.307E-02	5	LOC646817, SET, SMYD3, SET/NUP214 fusion protein, RUVBL1
195	macromolecular complex disassembly	174	1.075E-03	1.334E-02	1.075E-03	1.334E-02	5	Histone deacetylase class II, HSP70, SET, SET/NUP214 fusion protein, HSC70
196	regulation of macromolecule biosynthetic process	4455	1.088E-03	1.342E-02	1.088E-03	1.342E-02	33	eEF1A, RBBP8 (CtIP), Cyclin T1, DLC1 (Dynein LC8a), PSMD10 (Gankyrin), TIPIN, PPM1F, HDAC7, HEXIM1, Histone deacetylase class II, Olfactory receptor, Calmyrin, HSP70, Cyclophilin E, ARID4A, LOC646817, SET, Follistatin, SMYD3, eEF1A1, OBFC1, ZNF397OS, DYNLL, ICMT, ZNF397, SET/NUP214 fusion protein, EYA1, GMEB1, PP2C, HSC70, TARDBP (TDP43), RUVBL1, PQBP-1
197	cellular response to stress	1685	1.092E-03	1.342E-02	1.092E-03	1.342E-02	17	P5CR1, RBBP8 (CtIP), Cyclin B1, HSPA1B, PSMD10 (Gankyrin), TIPIN, Histone deacetylase class II, Calmyrin, HSP70, TXNRD1, HSPA1A, Cyclin B, EYA1, PP2C, TARDBP (TDP43), RUVBL1, CapZIP

198	regulation of epithelial cell migration	176	1.131E-03	1.375E-02	1.131E-03	1.375E-02	5	PPM1F, HDAC7, Histone deacetylase class II, Calmyrin, PP2C
199	protein-DNA complex assembly	176	1.131E-03	1.375E-02	1.131E-03	1.375E-02	5	LOC646817, SET, SMYD3, SET/NUP214 fusion protein, RUVBL1
200	response to stimulus	9680	1.158E-03	1.399E-02	1.158E-03	1.399E-02	58	Annexin V, IGHM, P5CR1, eEF1A, Reticulon 4, RBBP8 (CtIP), PGMU, IGHG4, Cyclin T1, Cyclin B1, DLC1 (Dynein LC8a), HSPA1B, PSMD10 (Gankyrin), TIPIN, ECE1, AL1A1, ARL5B, PPM1F, Beta crystallin B2, HDAC7, MENA, CDO1, Cytoglobin, Histone deacetylase class II, Olfactory receptor, Calmyrin, EPS8L1, COLEC12, HSP70, IGHG1, CLEC2D, eEF2K, Follistatin, TXNRD1, SMYD3, Calmodulin, HSPA2, HSPA1A, RBP1, eEF1A1, CKS2, OR10G3, Annexin XI, DYNLL, SET/NUP214 fusion protein, Cyclin B, IMD2, EYA1, Pleckstrin, PTPN2, RIG-G, PDXP, PP2C, HSC70, TARDBP (TDP43), RUVBL1, CapZIP, VILIP1
201	cellular component disassembly	470	1.162E-03	1.399E-02	1.162E-03	1.399E-02	8	Cyclin B1, Histone deacetylase class II, HSP70, SET, HSPA2, SET/NUP214 fusion protein, Cyclin B, HSC70
202	protein dephosphorylation	178	1.189E-03	1.424E-02	1.189E-03	1.424E-02	5	PPM1F, EYA1, PTPN2, PDXP, PP2C

203	regulation of cellular macromolecule biosynthetic process	4292	1.205E-03	1.434E-02	1.205E-03	1.434E-02	32	eEF1A, RBBP8 (CtIP), Cyclin T1, DLC1 (Dynein LC8a), PSMD10 (Gankyrin), TIPIN, PPM1F, HDAC7, HEXIM1, Histone deacetylase class II, Olfactory receptor, Calmyrin, HSP70, Cyclophilin E, ARID4A, LOC646817, SET, Follistatin, SMYD3, eEF1A1, OBFC1, ZNF397OS, DYNLL, ZNF397, SET/NUP214 fusion protein, EYA1, GMEB1, PP2C, HSC70, TARDBP (TDP43), RUVBL1, PQBP-1
204	negative regulation of nucleobase-containing compound metabolic process	1398	1.209E-03	1.434E-02	1.209E-03	1.434E-02	15	RBBP8 (CtIP), PSMD10 (Gankyrin), TIPIN, PPM1F, HDAC7, HEXIM1, Histone deacetylase class II, HSP70, ARID4A, SET, Follistatin, SET/NUP214 fusion protein, PP2C, HSC70, TARDBP (TDP43)
205	regulation of cellular component biogenesis	591	1.246E-03	1.468E-02	1.246E-03	1.468E-02	9	HSPA1B, PPM1F, Histone deacetylase class II, EPS8L1, HSP70, HSPA2, HSPA1A, Pleckstrin, PP2C
206	positive regulation of cell cycle	180	1.249E-03	1.468E-02	1.249E-03	1.468E-02	5	Cyclin B1, PSMD10 (Gankyrin), HSP70, HSPA2, Cyclin B
207	binding of sperm to zona pellucida	48	1.267E-03	1.481E-02	1.267E-03	1.481E-02	3	HSPA1B, HSP70, HSPA1A
20	cellular protein modification process	26	1.	1.	1.	1.	23	Cyclin T1, Cyclin B1, PSMD10 (Gankyrin), Fbxo3, PPM1F, HDAC7, Histone deacetylase class II, Cyclophilin E,

8		90	285E-03	488E-02	285E-03	488E-02		ARID4A, eEF2K, SMYD3, POLE3-POLE4 complex, SAE2, POLE3 (YBL1), ICMT, DOHH, Cyclin B, EYA1, PTPN2, FOP, PDXP, PP2C, RUVBL1
209	protein modification process	2690	1.285E-03	1.488E-02	1.285E-03	1.488E-02	23	Cyclin T1, Cyclin B1, PSMD10 (Gankyrin), Fbxo3, PPM1F, HDAC7, Histone deacetylase class II, Cyclophilin E, ARID4A, eEF2K, SMYD3, POLE3-POLE4 complex, SAE2, POLE3 (YBL1), ICMT, DOHH, Cyclin B, EYA1, PTPN2, FOP, PDXP, PP2C, RUVBL1
210	regulation of transcription, DNA-templated	3938	1.299E-03	1.497E-02	1.299E-03	1.497E-02	30	eEF1A, RBBP8 (CtIP), Cyclin T1, DLC1 (Dynein LC8a), PSMD10 (Gankyrin), PPM1F, HDAC7, HEXIM1, Histone deacetylase class II, Olfactory receptor, Calmyrin, HSP70, Cyclophilin E, ARID4A, LOC646817, SET, Follistatin, SMYD3, eEF1A1, ZNF397OS, DYNLL, ZNF397, SET/NUP214 fusion protein, EYA1, GMEB1, PP2C, HSC70, TARDBP (TDP43), RUVBL1, PQBP-1
211	cellular response to heat	49	1.346E-03	1.543E-02	1.346E-03	1.543E-02	3	HSPA1B, HSP70, HSPA1A
212	negative regulation of RNA metabolic process	1267	1.354E-03	1.546E-02	1.354E-03	1.546E-02	14	RBBP8 (CtIP), PSMD10 (Gankyrin), PPM1F, HDAC7, HEXIM1, Histone deacetylase class II, HSP70, ARID4A, SET, Follistatin, SET/NUP214 fusion protein, PP2C, HSC70, TARDBP (TDP43)
213	chromatin assembly or disassembly	184	1.37	1.56	1.37	1.56	5	LOC646817, SET, SMYD3, SET/NUP214 fusion protein, RUVBL1

			7E-03	5E-02	7E-03	5E-02		
214	single organism reproductive process	1418	1.393E-03	1.575E-02	1.393E-03	1.575E-02	15	Cyclin B1, DLC1 (Dynein LC8a), HSPA1B, AL1A1, CDO1, Olfactory receptor, Calmyrin, HSP70, Follistatin, HSPA2, HSPA1A, DYNLL, Cyclin B, PP2C, RUVBL1
215	response to organic substance	3414	1.432E-03	1.611E-02	1.432E-03	1.611E-02	27	Annexin V, eEF1A, Reticulon 4, Cyclin T1, Cyclin B1, HSPA1B, AL1A1, CDO1, Histone deacetylase class II, Calmyrin, COLEC12, HSP70, eEF2K, SMYD3, Calmodulin, HSPA2, HSPA1A, RBP1, eEF1A1, SET/NUP214 fusion protein, Cyclin B, IMD2, PTPN2, RIG-G, PDXP, PP2C, HSC70
216	negative regulation of transferase activity	377	1.452E-03	1.627E-02	1.452E-03	1.627E-02	7	PSMD10 (Gankyrin), PPM1F, HEXIM1, SMYD3, FOP, CAMK2N1, PP2C
217	regulation of attachment of spindle microtubules to kinetochore	13	1.473E-03	1.635E-02	1.473E-03	1.635E-02	2	Cyclin B1, Cyclin B
218	regulation of neurotransmitter levels	187	1.479E	1.635E	1.479E	1.635E	5	DLC1 (Dynein LC8a), VAMP5, HSP70, DYNLL, HSC70

			-03	-02	-03	-02		
219	multicellular organismal reproductive process	1135	1.480E-03	1.635E-02	1.480E-03	1.635E-02	13	Cyclin B1, DLC1 (Dynein LC8a), AL1A1, CDO1, Olfactory receptor, Calmyrin, HSP70, Follistatin, HSPA2, DYNLL, Cyclin B, PP2C, RUVBL1
220	negative regulation of macromolecule biosynthetic process	1430	1.514E-03	1.666E-02	1.514E-03	1.666E-02	15	RBBP8 (CtIP), PSMD10 (Gankyrin), TIPIN, PPM1F, HDAC7, HEXIM1, Histone deacetylase class II, HSP70, ARID4A, SET, Follistatin, SET/NUP214 fusion protein, PP2C, HSC70, TARDBP (TDP43)
221	anatomical structure development	5718	1.559E-03	1.707E-02	1.559E-03	1.707E-02	39	Reticulon 4, RBBP8 (CtIP), Cyclin B1, DLC1 (Dynein LC8a), HSPA1B, Clp1, ECE1, AL1A1, Beta crystallin B2, HDAC7, MENA, CDO1, HEXIM1, VAMP5, Histone deacetylase class II, Olfactory receptor, Calmyrin, HSP70, ARID4A, LOC646817, Follistatin, TXNRD1, SMYD3, Calmodulin, HSPA2, HSPA1A, DYNLL, ICMT, Cyclin B, IMD2, EYA1, Pleckstrin, PTPN2, Fructosamine-3-kinase, PP2C, HSC70, TCOF1, PQBP-1, VILIP1
222	reproduction	1742	1.570E-03	1.711E-02	1.570E-03	1.711E-02	17	RBBP8 (CtIP), Cyclin B1, DLC1 (Dynein LC8a), HSPA1B, AL1A1, CDO1, Olfactory receptor, Calmyrin, HSP70, Follistatin, HSPA2, HSPA1A, CKS2, DYNLL, Cyclin B, PP2C, RUVBL1
223	negative regulation of kinase activity	282	1.614E-0	1.751E-0	1.614E-0	1.751E-0	6	PPM1F, HEXIM1, SMYD3, FOP, CAMK2N1, PP2C

			3	2	3	2		
224	[regulation of spindle organization]	14	1.713E-03	1.843E-02	1.713E-03	1.843E-02	2	Cyclin B1, Cyclin B
225	[terpenoid biosynthetic process]	14	1.713E-03	1.843E-02	1.713E-03	1.843E-02	2	AL1A1, RBP1
226	[multicellular organism reproduction]	1156	1.742E-03	1.866E-02	1.742E-03	1.866E-02	13	Cyclin B1, DLC1 (Dynein LC8a), AL1A1, CDO1, Olfactory receptor, Calmyrin, HSP70, Follistatin, HSPA2, DYNLL, Cyclin B, PP2C, RUVBL1
227	[sexual reproduction]	1019	1.804E-03	1.924E-02	1.804E-03	1.924E-02	12	Cyclin B1, DLC1 (Dynein LC8a), HSPA1B, Olfactory receptor, Calmyrin, HSP70, Follistatin, HSPA2, HSPA1A, DYNLL, Cyclin B, RUVBL1
228	[biological regulation]	12947	1.877E-03	1.986E-02	1.877E-03	1.986E-02	71	Annexin V, eEF1A, Reticulon 4, RBBP8 (CtIP), PGMU, IGHG4, Cyclin T1, Cyclin B1, DLC1 (Dynein LC8a), HSPA1B, PSMD10 (Gankyrin), TIPIN, Clp1, ECE1, AL1A1, ARL5B, PPM1F, HDAC7, MENA, CDO1, SNX1, HEXIM1, VAMP5, Histone deacetylase class II, Olfactory receptor, Thioredoxin-like 2, Calmyrin, EPS8L1, COLEC12, HSP70, IGHG1, Cyclophilin E, ARID4A, Annexin VI, CLEC2D, eEF2K, LOC646817, SET, Follistatin, TXNRD1, SMYD3, Calmodulin, HSPA2, HSPA1A, RBP1, eEF1A1, SAE2, CKS2,

								OR10G3, OBFC1, ZNF397OS, DYNLL, ICMT, ZNF397, SET/NUP214 fusion protein, Cyclin B, EYA1, GMEB1, Pleckstrin, PTPN2, FOP, RIG-G, PDXP, CAMK2N1, PP2C, HSC70, TARDBP (TDP43), PRCC, RUVBL1, PQBP-1, VILIP1
229	positive regulation of actin filament bundle assembly	55	1.880E-03	1.986E-02	1.880E-03	1.986E-02	3	PPM1F, Pleckstrin, PP2C
230	protein folding	292	1.924E-03	2.024E-02	1.924E-03	2.024E-02	6	HSPA1B, HSP70, Cyclophilin E, HSPA2, HSPA1A, HSC70
231	negative regulation of nitric oxide biosynthetic process	15	1.971E-03	2.056E-02	1.971E-03	2.056E-02	2	DLC1 (Dynein LC8a), DYNLL
232	negative regulation of interleukin-2 production	15	1.971E-03	2.056E-02	1.971E-03	2.056E-02	2	HDAC7, Histone deacetylase class II
233	regulation of erythrocyte differentiation	57	2.083E-[illegible]	2.163E-[illegible]	2.083E-[illegible]	2.163E-[illegible]	3	HSPA1B, HSP70, HSPA1A

			03	02	03	02		
234	heterocycle biosynthetic process	3324	2.122E-03	2.195E-02	2.122E-03	2.195E-02	26	P5CR1, eEF1A, RBBP8 (CtIP), Cyclin T1, DLC1 (Dynein LC8a), Clp1, HDAC7, HEXIM1, Histone deacetylase class II, HSP70, ARID4A, LOC646817, COASY, eEF1A1, ZNF397OS, DYNLL, ZNF397, IMD2, EYA1, GMEB1, PP2C, HSC70, TARDBP (TDP43), TCOF1, RUVBL1, PQBP-1
235	mitotic cell cycle checkpoint	205	2.211E-03	2.277E-02	2.211E-03	2.277E-02	5	Cyclin B1, PSMD10 (Gankyrin), TIPIN, Cyclin B, PRCC
236	cellular macromolecular complex assembly	644	2.240E-03	2.294E-02	2.240E-03	2.294E-02	9	PSMD10 (Gankyrin), Clp1, HSP70, LOC646817, SET, SMYD3, SET/NUP214 fusion protein, RUVBL1, PQBP-1
237	galactose metabolic process	16	2.246E-03	2.294E-02	2.246E-03	2.294E-02	2	PGMU, PGM2
238	protein-DNA complex subunit organization	207	2.306E-03	2.345E-02	2.306E-03	2.345E-02	5	LOC646817, SET, SMYD3, SET/NUP214 fusion protein, RUVBL1

239	negative regulation of cytoskeleton organization	125	2.341E-03	2.370E-02	2.341E-03	2.370E-02	4	Cyclin B1, Histone deacetylase class II, Calmyrin, Cyclin B
240	positive regulation of T cell mediated cytotoxicity	60	2.413E-03	2.423E-02	2.413E-03	2.423E-02	3	HSPA1B, HSP70, HSPA1A
241	regulation of cytokinesis	60	2.413E-03	2.423E-02	2.413E-03	2.423E-02	3	Olfactory receptor, Calmodulin, PDXP
242	negative regulation of cellular macromolecule biosynthetic process	1349	2.431E-03	2.427E-02	2.431E-03	2.427E-02	14	RBBP8 (CtIP), PSMD10 (Gankyrin), TIPIN, PPM1F, HDAC7, HEXIM1, Histone deacetylase class II, HSP70, ARID4A, SET, Follistatin, SET/NUP214 fusion protein, PP2C, HSC70
243	negative regulation of transcription, DNA-templated	1201	2.437E-03	2.427E-02	2.437E-03	2.427E-02	13	RBBP8 (CtIP), PSMD10 (Gankyrin), PPM1F, HDAC7, HEXIM1, Histone deacetylase class II, HSP70, ARID4A, SET, Follistatin, SET/NUP214 fusion protein, PP2C, HSC70
24	sperm-egg recognition	61	2.	2.	2.	2.	3	HSPA1B, HSP70, HSPA1A

4			530E-03	508E-02	530E-03	508E-02		
245	pharyngeal system development	17	2.539E-03	2.508E-02	2.539E-03	2.508E-02	2	ECE1, EYA1
246	regulation of multicellular organismal process	2833	2.557E-03	2.515E-02	2.557E-03	2.515E-02	23	Annexin V, Reticulon 4, Cyclin B1, HSPA1B, ECE1, PPM1F, HDAC7, Histone deacetylase class II, Thioredoxin-like 2, Calmyrin, HSP70, Annexin VI, CLEC2D, Follistatin, Calmodulin, HSPA1A, RBP1, Cyclin B, EYA1, Pleckstrin, PTPN2, PP2C, PQBP-1
247	regulation of histone modification	129	2.624E-03	2.567E-02	2.624E-03	2.567E-02	4	Cyclin B1, SET, SET/NUP214 fusion protein, Cyclin B
248	positive regulation of cell migration	419	2.634E-03	2.567E-02	2.634E-03	2.567E-02	7	PPM1F, HDAC7, Histone deacetylase class II, Calmyrin, HSP70, FOP, PP2C
249	response to radiation	660	2.64	2.56	2.64	2.56	9	PGMU, HSPA1B, TIPIN, HSP70, Calmodulin, HSPA1A, RBP1, EYA1, PP2C

			1E-03	7E-02	1E-03	7E-02		
250	DNA packaging	214	2.662E-03	2.577E-02	2.662E-03	2.577E-02	5	LOC646817, SET, SMYD3, SET/NUP214 fusion protein, RUVBL1
251	macromolecule modification	2847	2.725E-03	2.628E-02	2.725E-03	2.628E-02	23	Cyclin T1, Cyclin B1, PSMD10 (Gankyrin), Fbxo3, PPM1F, HDAC7, Histone deacetylase class II, Cyclophilin E, ARID4A, eEF2K, SMYD3, POLE3-POLE4 complex, SAE2, POLE3 (YBL1), ICMT, DOHH, Cyclin B, EYA1, PTPN2, FOP, PDXP, PP2C, RUVBL1
252	negative regulation of extrinsic apoptotic signaling pathway	131	2.774E-03	2.664E-02	2.774E-03	2.664E-02	4	HSPA1B, HSP70, HSPA1A, EYA1
253	protein oligomerization	542	2.829E-03	2.685E-02	2.829E-03	2.685E-02	8	Annexin V, eEF1A, AL1A1, COLEC12, Annexin VI, TXNRD1, eEF1A1, IMD2
254	mitotic metaphase plate congression	18	2.848E	2.685E	2.848E	2.685E	2	Cyclin B1, Cyclin B

			-03	-02	-03	-02		
255	ventricular cardiac muscle cell development	18	2.848E-03	2.685E-02	2.848E-03	2.685E-02	2	Cyclin B1, Cyclin B
256	regulation of microtubule cytoskeleton organization	132	2.851E-03	2.685E-02	2.851E-03	2.685E-02	4	Cyclin B1, Histone deacetylase class II, Calmyrin, Cyclin B
257	transcription, DNA-templated	2857	2.852E-03	2.685E-02	2.852E-03	2.685E-02	23	eEF1A, RBBP8 (CtIP), Cyclin T1, DLC1 (Dynein LC8a), Clp1, HDAC7, HEXIM1, Histone deacetylase class II, HSP70, ARID4A, LOC646817, eEF1A1, ZNF397OS, DYNLL, ZNF397, EYA1, GMEB1, PP2C, HSC70, TARDBP (TDP43), TCOF1, RUVBL1, PQBP-1
258	positive regulation of cellular component organization	936	2.863E-03	2.685E-02	2.863E-03	2.685E-02	11	Cyclin B1, DLC1 (Dynein LC8a), PPM1F, Histone deacetylase class II, Calmyrin, EPS8L1, DYNLL, Cyclin B, Pleckstrin, PDXP, PP2C
259	regulation of locomotion	804	2.986E-0	2.782E-0	2.986E-0	2.782E-0	10	Annexin V, Reticulon 4, PPM1F, HDAC7, Histone deacetylase class II, Calmyrin, HSP70, PTPN2, FOP, PP2C

			3	2	3	2		
260	regulation of biological quality	3588	2.989E-03	2.782E-02	2.989E-03	2.782E-02	27	Annexin V, Reticulon 4, PGMU, DLC1 (Dynein LC8a), HSPA1B, PSMD10 (Gankyrin), ECE1, AL1A1, CDO1, VAMP5, Thioredoxin-like 2, HSP70, ARID4A, TXNRD1, Calmodulin, HSPA1A, RBP1, OBFC1, DYNLL, Cyclin B, Pleckstrin, PTPN2, FOP, PDXP, PP2C, HSC70, TARDBP (TDP43)
261	negative regulation of multicellular organismal process	548	3.025E-03	2.805E-02	3.025E-03	2.805E-02	8	Annexin V, HSPA1B, HDAC7, Histone deacetylase class II, Thioredoxin-like 2, HSP70, HSPA1A, PP2C
262	positive regulation of cell motility	430	3.040E-03	2.808E-02	3.040E-03	2.808E-02	7	PPM1F, HDAC7, Histone deacetylase class II, Calmyrin, HSP70, FOP, PP2C
263	meiotic nuclear division	222	3.117E-03	2.868E-02	3.117E-03	2.868E-02	5	RBBP8 (CtIP), HSP70, HSPA2, CKS2, Cyclin B
264	cellular response to DNA damage stimulus	810	3.149E-03	2.886E-02	3.149E-03	2.886E-02	10	RBBP8 (CtIP), HSPA1B, PSMD10 (Gankyrin), TIPIN, Calmyrin, HSP70, HSPA1A, EYA1, PP2C, RUVBL1

265	positive regulation of protein depolymerization	19	3.174E-03	2.887E-02	3.174E-03	2.887E-02	2	Pleckstrin, PDXP
266	negative regulation of histone acetylation	19	3.174E-03	2.887E-02	3.174E-03	2.887E-02	2	SET, SET/NUP214 fusion protein
267	nuclear division	553	3.197E-03	2.897E-02	3.197E-03	2.897E-02	8	RBBP8 (CtIP), Cyclin B1, TIPIN, HSP70, HSPA2, CKS2, Cyclin B, RUVBL1
268	regulation of biological process	12391	3.230E-03	2.912E-02	3.230E-03	2.912E-02	68	Annexin V, eEF1A, Reticulon 4, RBBP8 (CtIP), IGHG4, Cyclin T1, Cyclin B1, DLC1 (Dynein LC8a), HSPA1B, PSMD10 (Gankyrin), TIPIN, Clp1, ECE1, AL1A1, ARL5B, PPM1F, HDAC7, MENA, SNX1, HEXIM1, Histone deacetylase class II, Olfactory receptor, Thioredoxin-like 2, Calmyrin, EPS8L1, COLEC12, HSP70, IGHG1, Cyclophilin E, ARID4A, Annexin VI, CLEC2D, eEF2K, LOC646817, SET, Follistatin, TXNRD1, SMYD3, Calmodulin, HSPA2, HSPA1A, RBP1, eEF1A1, SAE2, CKS2, OR10G3, OBFC1, ZNF397OS, DYNLL, ICMT, ZNF397, SET/NUP214 fusion protein, Cyclin B, EYA1, GMEB1, Pleckstrin, PTPN2, FOP, RIG-G, PDXP, CAMK2N1, PP2C, HSC70, TARDBP (TDP43), PRCC, RUVBL1, PQBP-1, VILIP1
269	regulation of gene expression	5135	3.237E-0	2.912E-0	3.237E-0	2.912E-0	35	eEF1A, RBBP8 (CtIP), Cyclin T1, Cyclin B1, DLC1 (Dynein LC8a), HSPA1B, PSMD10 (Gankyrin), Clp1, PPM1F, HDAC7, HEXIM1, Histone deacetylase class II, Olfactory receptor, Calmyrin, HSP70, Cyclophilin E, ARID4A, LOC646817, SET, Follistatin, SMYD3, HSPA1A, eEF1A1, ZNF397OS, DYNLL, ZNF397, SET/NUP214 fusion protein, Cyclin B, EYA1, GMEB1, PP2C, HSC70, TARDBP (TDP43), RUVBL1, PQBP-1

			3	2	3	2		
270	nucleobase-containing compound biosynthetic process	3245	3.267E-03	2.923E-02	3.267E-03	2.923E-02	25	eEF1A, RBBP8 (CtIP), Cyclin T1, DLC1 (Dynein LC8a), Clp1, HDAC7, HEXIM1, Histone deacetylase class II, HSP70, ARID4A, LOC646817, COASY, eEF1A1, ZNF397OS, DYNLL, ZNF397, IMD2, EYA1, GMEB1, PP2C, HSC70, TARDBP (TDP43), TCOF1, RUVBL1, PQBP-1
271	response to mechanical stimulus	325	3.274E-03	2.923E-02	3.274E-03	2.923E-02	6	Cyclin B1, HSPA1B, ECE1, HSP70, HSPA1A, Cyclin B
272	negative regulation of NF-kappaB import into nucleus	20	3.516E-03	3.087E-02	3.516E-03	3.087E-02	2	PSMD10 (Gankyrin), PP2C
273	positive regulation of cell junction assembly	20	3.516E-03	3.087E-02	3.516E-03	3.087E-02	2	PPM1F, PP2C
274	positive regulation of focal adhesion assembly	20	3.516E-03	3.087E-02	3.516E-03	3.087E-02	2	PPM1F, PP2C

275	nitric oxide biosynthetic process	20	3.516E-03	3.087E-02	3.516E-03	3.087E-02	2	DLC1 (Dynein LC8a), DYNLL
276	regulation of chromatin organization	140	3.521E-03	3.087E-02	3.521E-03	3.087E-02	4	Cyclin B1, SET, SET/NUP214 fusion protein, Cyclin B
277	positive regulation of cellular component movement	443	3.581E-03	3.125E-02	3.581E-03	3.125E-02	7	PPM1F, HDAC7, Histone deacetylase class II, Calmyrin, HSP70, FOP, PP2C
278	nuclear envelope organization	69	3.590E-03	3.125E-02	3.590E-03	3.125E-02	3	Cyclin B1, SET/NUP214 fusion protein, Cyclin B
279	regulation of T cell mediated cytotoxicity	70	3.738E-03	3.243E-02	3.738E-03	3.243E-02	3	HSPA1B, HSP70, HSPA1A
28	gamete generation	83	3.	3.	3.	3.	10	Cyclin B1, DLC1 (Dynein LC8a), Olfactory receptor, Calmyrin, HSP70, Follistatin, HSPA2, DYNLL, Cyclin B,

0		3	839E-03	285E-02	839E-03	285E-02		RUVBL1
281	positive regulation of locomotion	449	3.854E-03	3.285E-02	3.854E-03	3.285E-02	7	PPM1F, HDAC7, Histone deacetylase class II, Calmyrin, HSP70, FOP, PP2C
282	system development	4990	3.860E-03	3.285E-02	3.860E-03	3.285E-02	34	Reticulon 4, Cyclin B1, DLC1 (Dynein LC8a), HSPA1B, Clp1, ECE1, AL1A1, Beta crystallin B2, HDAC7, MENA, CDO1, HEXIM1, VAMP5, Histone deacetylase class II, Olfactory receptor, Calmyrin, HSP70, ARID4A, Follistatin, TXNRD1, Calmodulin, HSPA1A, DYNLL, ICMT, Cyclin B, IMD2, EYA1, Pleckstrin, PTPN2, PP2C, HSC70, TCOF1, PQBP-1, VILIP1
283	positive regulation of mRNA 3'-end processing	21	3.875E-03	3.285E-02	3.875E-03	3.285E-02	2	Cyclin B1, Cyclin B
284	deoxyribose phosphate catabolic process	21	3.875E-03	3.285E-02	3.875E-03	3.285E-02	2	FLJ22494, PGM2
285	regulation of muscle adaptation	71	3.89	3.28	3.89	3.28	3	ECE1, Histone deacetylase class II, Thioredoxin-like 2

			1E-03	5E-02	1E-03	5E-02		
286	meiotic cell cycle	235	3.971E-03	3.285E-02	3.971E-03	3.285E-02	5	RBBP8 (CtIP), HSP70, HSPA2, CKS2, Cyclin B
287	cellular process involved in reproduction	703	4.005E-03	3.285E-02	4.005E-03	3.285E-02	9	RBBP8 (CtIP), Cyclin B1, HSPA1B, Calmyrin, HSP70, HSPA2, HSPA1A, CKS2, Cyclin B
288	organic cyclic compound biosynthetic process	3482	4.079E-03	3.285E-02	4.079E-03	3.285E-02	26	P5CR1, eEF1A, RBBP8 (CtIP), Cyclin T1, DLC1 (Dynein LC8a), Clp1, HDAC7, HEXIM1, Histone deacetylase class II, HSP70, ARID4A, LOC646817, COASY, eEF1A1, ZNF397OS, DYNLL, ZNF397, IMD2, EYA1, GMEB1, PP2C, HSC70, TARDBP (TDP43), TCOF1, RUVBL1, PQBP-1
289	negative regulation of mitotic cell cycle phase transition	238	4.189E-03	3.285E-02	4.189E-03	3.285E-02	5	Cyclin B1, PSMD10 (Gankyrin), TIPIN, Cyclin B, PRCC
290	cellular response to chemical stimulus	2945	4.195E	3.285E	4.195E	3.285E	23	P5CR1, eEF1A, Reticulon 4, Cyclin T1, Cyclin B1, AL1A1, PPM1F, Histone deacetylase class II, Calmyrin, COLEC12, HSP70, eEF2K, TXNRD1, SMYD3, Calmodulin, eEF1A1, SET/NUP214 fusion protein, Cyclin B, IMD2, PTPN2, RIG-G, PDXP, PP2C

			-03	-02	-03	-02		
291	cell-cell recognition	73	4.207E-03	3.285E-02	4.207E-03	3.285E-02	3	HSPA1B, HSP70, HSPA1A
292	chaperone mediated protein folding requiring cofactor	22	4.250E-03	3.285E-02	4.250E-03	3.285E-02	2	HSP70, HSC70
293	negative regulation of peptidyl-lysine acetylation	22	4.250E-03	3.285E-02	4.250E-03	3.285E-02	2	SET, SET/NUP214 fusion protein
294	single fertilization	148	4.291E-03	3.285E-02	4.291E-03	3.285E-02	4	HSPA1B, Olfactory receptor, HSP70, HSPA1A
295	RNA biosynthetic process	2953	4.341E-0	3.285E-0	4.341E-0	3.285E-0	23	eEF1A, RBBP8 (CtIP), Cyclin T1, DLC1 (Dynein LC8a), Clp1, HDAC7, HEXIM1, Histone deacetylase class II, HSP70, ARID4A, LOC646817, eEF1A1, ZNF397OS, DYNLL, ZNF397, EYA1, GMEB1, PP2C, HSC70, TARDBP (TDP43), TCOF1, RUVBL1, PQBP-1

			3	2	3	2		
296	positive regulation of endothelial cell migration	74	4.371E-03	3.285E-02	4.371E-03	3.285E-02	3	HDAC7, Histone deacetylase class II, Calmyrin
297	organelle fission	583	4.392E-03	3.285E-02	4.392E-03	3.285E-02	8	RBBP8 (CtIP), Cyclin B1, TIPIN, HSP70, HSPA2, CKS2, Cyclin B, RUVBL1
298	endothelin maturation	1	4.439E-03	3.285E-02	4.439E-03	3.285E-02	1	ECE1
299	negative regulation of prolactin signaling pathway	1	4.439E-03	3.285E-02	4.439E-03	3.285E-02	1	PTPN2
300	dADP catabolic process	1	4.439E-03	3.285E-02	4.439E-03	3.285E-02	1	FLJ22494

301	regulation of cell diameter	1	4.439E-03	3.285E-02	4.439E-03	3.285E-02	1	Pleckstrin
302	negative regulation of macrophage colony-stimulating factor signaling pathway	1	4.439E-03	3.285E-02	4.439E-03	3.285E-02	1	PTPN2
303	positive regulation of inositol-polyphosphate 5-phosphatase activity	1	4.439E-03	3.285E-02	4.439E-03	3.285E-02	1	Pleckstrin
304	substance P catabolic process	1	4.439E-03	3.285E-02	4.439E-03	3.285E-02	1	ECE1
305	regulation of macrophage colony-stimulating factor signaling pathway	1	4.439E-03	3.285E-02	4.439E-03	3.285E-02	1	PTPN2
30	regulation of interleukin-4-mediated signaling	1	4.	3.	4.	3.	1	PTPN2

6	pathway		439E-03	285E-02	439E-03	285E-02		
307	organomercury metabolic process	1	4.439E-03	3.285E-02	4.439E-03	3.285E-02	1	TXNRD1
308	pyridoxal phosphate catabolic process	1	4.439E-03	3.285E-02	4.439E-03	3.285E-02	1	PDXP
309	negative regulation of interleukin-2-mediated signaling pathway	1	4.439E-03	3.285E-02	4.439E-03	3.285E-02	1	PTPN2
310	halogen metabolic process	1	4.439E-03	3.285E-02	4.439E-03	3.285E-02	1	TXNRD1
311	fructoselysine metabolic process	1	4.43	3.28	4.43	3.28	1	Fructosamine-3-kinase

			9E-03	5E-02	9E-03	5E-02		
312	dGDP catabolic process	1	4.439E-03	3.285E-02	4.439E-03	3.285E-02	1	FLJ22494
313	organometal metabolic process	1	4.439E-03	3.285E-02	4.439E-03	3.285E-02	1	TXNRD1
314	deoxyribonucleoside diphosphate catabolic process	1	4.439E-03	3.285E-02	4.439E-03	3.285E-02	1	FLJ22494
315	GDP catabolic process	1	4.439E-03	3.285E-02	4.439E-03	3.285E-02	1	FLJ22494
316	aspartyl-tRNA aminoacylation	1	4.439E	3.285E	4.439E	3.285E	1	DARS

			-03	-02	-03	-02		
317	regulation of positive thymic T cell selection	1	4.439E-03	3.285E-02	4.439E-03	3.285E-02	1	PTPN2
318	siRNA loading onto RISC involved in RNA interference	1	4.439E-03	3.285E-02	4.439E-03	3.285E-02	1	Clp1
319	negative regulation of interleukin-4-mediated signaling pathway	1	4.439E-03	3.285E-02	4.439E-03	3.285E-02	1	PTPN2
320	negative regulation of positive thymic T cell selection	1	4.439E-03	3.285E-02	4.439E-03	3.285E-02	1	PTPN2
321	regulation of interleukin-2-mediated signaling pathway	1	4.439E-0	3.285E-0	4.439E-0	3.285E-0	1	PTPN2

			3	2	3	2		
322	regulation of prolactin signaling pathway	1	4.439E-03	3.285E-02	4.439E-03	3.285E-02	1	PTPN2
323	methylmercury metabolic process	1	4.439E-03	3.285E-02	4.439E-03	3.285E-02	1	TXNRD1
324	calcitonin catabolic process	1	4.439E-03	3.285E-02	4.439E-03	3.285E-02	1	ECE1
325	phospholipase C-inhibiting G-protein coupled receptor signaling pathway	1	4.439E-03	3.285E-02	4.439E-03	3.285E-02	1	Pleckstrin
326	purine deoxyribonucleoside diphosphate catabolic process	1	4.439E-03	3.285E-02	4.439E-03	3.285E-02	1	FLJ22494

327	regulation of inositol-polyphosphate 5-phosphatase activity	1	4.439E-03	3.285E-02	4.439E-03	3.285E-02	1	Pleckstrin
328	negative regulation of cell cycle phase transition	243	4.573E-03	3.373E-02	4.573E-03	3.373E-02	5	Cyclin B1, PSMD10 (Gankyrin), TIPIN, Cyclin B, PRCC
329	response to metal ion	464	4.606E-03	3.373E-02	4.606E-03	3.373E-02	7	Annexin V, Cyclin B1, ECE1, TXNRD1, Calmodulin, Annexin XI, Cyclin B
330	platelet formation	23	4.642E-03	3.373E-02	4.642E-03	3.373E-02	2	Calmyrin, PP2C
331	negative regulation of peptidyl-serine phosphorylation	23	4.642E-03	3.373E-02	4.642E-03	3.373E-02	2	PPM1F, PP2C
33	platelet morphogenesis	23	4.	3.	4.	3.	2	Calmyrin, PP2C

2			642E-03	373E-02	642E-03	373E-02		
333	negative regulation of protein acetylation	23	4.642E-03	3.373E-02	4.642E-03	3.373E-02	2	SET, SET/NUP214 fusion protein
334	regulation of blood vessel endothelial cell migration	76	4.710E-03	3.407E-02	4.710E-03	3.407E-02	3	HDAC7, Histone deacetylase class II, Calmyrin
335	female gamete generation	152	4.716E-03	3.407E-02	4.716E-03	3.407E-02	4	Cyclin B1, DLC1 (Dynein LC8a), DYNLL, Cyclin B
336	aromatic compound biosynthetic process	3336	4.732E-03	3.408E-02	4.732E-03	3.408E-02	25	eEF1A, RBBP8 (CtIP), Cyclin T1, DLC1 (Dynein LC8a), Clp1, HDAC7, HEXIM1, Histone deacetylase class II, HSP70, ARID4A, LOC646817, COASY, eEF1A1, ZNF397OS, DYNLL, ZNF397, IMD2, EYA1, GMEB1, PP2C, HSC70, TARDBP (TDP43), TCOF1, RUVBL1, PQBP-1
337	positive regulation of T cell mediated immunity	77	4.88	3.50	4.88	3.50	3	HSPA1B, HSP70, HSPA1A

			6E-03	6E-02	6E-03	6E-02		
338	reproductive process	1617	4.897E-03	3.506E-02	4.897E-03	3.506E-02	15	Cyclin B1, DLC1 (Dynein LC8a), HSPA1B, AL1A1, CDO1, Olfactory receptor, Calmyrin, HSP70, Follistatin, HSPA2, HSPA1A, DYNLL, Cyclin B, PP2C, RUVBL1
339	regulation of cardiac muscle contraction by calcium ion signaling	24	5.049E-03	3.583E-02	5.049E-03	3.583E-02	2	Histone deacetylase class II, Calmodulin
340	regulation of mRNA 3'-end processing	24	5.049E-03	3.583E-02	5.049E-03	3.583E-02	2	Cyclin B1, Cyclin B
341	positive regulation of cardiac muscle cell proliferation	24	5.049E-03	3.583E-02	5.049E-03	3.583E-02	2	Cyclin B1, Cyclin B
342	regulation of microtubule-based process	156	5.168E	3.657E	5.168E	3.657E	4	Cyclin B1, Histone deacetylase class II, Calmyrin, Cyclin B

			-03	-02	-03	-02		
343	regulation of cell growth	476	5.283E-03	3.707E-02	5.283E-03	3.707E-02	7	Reticulon 4, HSPA1B, PSMD10 (Gankyrin), Calmyrin, HSP70, HSPA1A, FOP
344	positive regulation of angiogenesis	157	5.285E-03	3.707E-02	5.285E-03	3.707E-02	4	HDAC7, Histone deacetylase class II, Calmyrin, HSP70
345	regulation of striated muscle tissue development	157	5.285E-03	3.707E-02	5.285E-03	3.707E-02	4	Cyclin B1, HDAC7, Histone deacetylase class II, Cyclin B
346	regulation of muscle system process	253	5.414E-03	3.730E-02	5.414E-03	3.730E-02	5	ECE1, Histone deacetylase class II, Thioredoxin-like 2, Annexin VI, Calmodulin
347	regulation of cell motility	737	5.433E-0	3.730E-0	5.433E-0	3.730E-0	9	Annexin V, Reticulon 4, PPM1F, HDAC7, Histone deacetylase class II, Calmyrin, HSP70, FOP, PP2C

			3	2	3	2		
348	regulation of actin filament bundle assembly	80	5.435E-03	3.730E-02	5.435E-03	3.730E-02	3	PPM1F, Pleckstrin, PP2C
349	response to chemical	5293	5.453E-03	3.730E-02	5.453E-03	3.730E-02	35	Annexin V, P5CR1, eEF1A, Reticulon 4, Cyclin T1, Cyclin B1, HSPA1B, ECE1, AL1A1, PPM1F, MENA, CDO1, Histone deacetylase class II, Olfactory receptor, Calmyrin, COLEC12, HSP70, eEF2K, TXNRD1, SMYD3, Calmodulin, HSPA2, HSPA1A, RBP1, eEF1A1, OR10G3, Annexin XI, SET/NUP214 fusion protein, Cyclin B, IMD2, PTPN2, RIG-G, PDXP, PP2C, HSC70
350	regulation of apoptotic signaling pathway	479	5.464E-03	3.730E-02	5.464E-03	3.730E-02	7	DLC1 (Dynein LC8a), HSPA1B, PSMD10 (Gankyrin), HSP70, HSPA1A, DYNLL, EYA1
351	metaphase plate congression	25	5.472E-03	3.730E-02	5.472E-03	3.730E-02	2	Cyclin B1, Cyclin B
352	synaptonemal complex organization	25	5.472E-03	3.730E-02	5.472E-03	3.730E-02	2	HSP70, HSPA2

353	response to insecticide	25	5.472E-03	3.730E-02	5.472E-03	3.730E-02	2	Cyclin B1, Cyclin B
354	positive regulation of protein complex disassembly	25	5.472E-03	3.730E-02	5.472E-03	3.730E-02	2	Pleckstrin, PDXP
355	histone H4 deacetylation	25	5.472E-03	3.730E-02	5.472E-03	3.730E-02	2	HDAC7, Histone deacetylase class II
356	regulation of muscle organ development	159	5.526E-03	3.746E-02	5.526E-03	3.746E-02	4	Cyclin B1, HDAC7, Histone deacetylase class II, Cyclin B
357	regulation of muscle tissue development	159	5.526E-03	3.746E-02	5.526E-03	3.746E-02	4	Cyclin B1, HDAC7, Histone deacetylase class II, Cyclin B
35	macromolecule catabolic process	10	5.	3.	5.	3.	11	RBBP8 (CtIP), PGMU, Cyclin B1, HSPA1B, PSMD10 (Gankyrin), Histone deacetylase class II, HSP70, PGM2,

8		26	700E-03	853E-02	700E-03	853E-02		Calmodulin, HSPA1A, Cyclin B
359	RNA splicing	365	5.737E-03	3.867E-02	5.737E-03	3.867E-02	6	RY1, Clp1, HSP70, Cyclophilin E, HSC70, TARDBP (TDP43)
360	response to ethanol	258	5.873E-03	3.948E-02	5.873E-03	3.948E-02	5	HSPA1B, AL1A1, CDO1, HSP70, HSPA1A
361	male meiosis I	26	5.911E-03	3.963E-02	5.911E-03	3.963E-02	2	HSP70, HSPA2
362	regulation of cysteine-type endopeptidase activity involved in apoptotic process	259	5.968E-03	3.990E-02	5.968E-03	3.990E-02	5	HSPA1B, PPM1F, HSP70, HSPA1A, PP2C
363	cellular nitrogen compound biosynthetic process	3397	5.99	3.99	5.99	3.99	25	eEF1A, RBBP8 (CtIP), Cyclin T1, DLC1 (Dynein LC8a), Clp1, HDAC7, HEXIM1, Histone deacetylase class II, HSP70, ARID4A, LOC646817, COASY, eEF1A1, ZNF397OS, DYNLL, ZNF397, IMD2, EYA1, GMEB1, PP2C,

			8E-03	9E-02	8E-03	9E-02		HSC70, TARDBP (TDP43), TCOF1, RUVBL1, PQBP-1
364	regulation of cell cycle process	616	6.078E-03	4.041E-02	6.078E-03	4.041E-02	8	Cyclin B1, PSMD10 (Gankyrin), TIPIN, Olfactory receptor, Calmodulin, Cyclin B, PDXP, PRCC
365	glycogen metabolic process	84	6.224E-03	4.126E-02	6.224E-03	4.126E-02	3	PGMU, PGM2, Calmodulin
366	DNA replication	262	6.260E-03	4.139E-02	6.260E-03	4.139E-02	5	TIPIN, SET, POLE3-POLE4 complex, POLE3 (YBL1), SET/NUP214 fusion protein
367	response to inorganic substance	756	6.387E-03	4.212E-02	6.387E-03	4.212E-02	9	Annexin V, eEF1A, Cyclin B1, ECE1, Histone deacetylase class II, TXNRD1, Calmodulin, Annexin XI, Cyclin B
368	glucan metabolic process	85	6.431E	4.218E	6.431E	4.218E	3	PGMU, PGM2, Calmodulin

			-03	-02	-03	-02		
369	cellular glucan metabolic process	85	6.431E-03	4.218E-02	6.431E-03	4.218E-02	3	PGMU, PGM2, Calmodulin
370	DNA repair	499	6.789E-03	4.438E-02	6.789E-03	4.438E-02	7	RBBP8 (CtIP), HSPA1B, Calmyrin, HSP70, HSPA1A, EYA1, RUVBL1
371	protein depolymerization	28	6.836E-03	4.438E-02	6.836E-03	4.438E-02	2	HSP70, HSC70
372	retinoic acid metabolic process	28	6.836E-03	4.438E-02	6.836E-03	4.438E-02	2	AL1A1, RBP1
373	positive regulation of leukocyte mediated cytotoxicity	87	6.858E-0	4.438E-0	6.858E-0	4.438E-0	3	HSPA1B, HSP70, HSPA1A

			3	2	3	2		
374	regulation of cellular process	11707	6.859E-03	4.438E-02	6.859E-03	4.438E-02	64	Annexin V, eEF1A, Reticulon 4, RBBP8 (CtIP), IGHG4, Cyclin T1, Cyclin B1, DLC1 (Dynein LC8a), HSPA1B, PSMD10 (Gankyrin), TIPIN, ECE1, AL1A1, ARL5B, PPM1F, HDAC7, MENA, HEXIM1, Histone deacetylase class II, Olfactory receptor, Thioredoxin-like 2, Calmyrin, EPS8L1, COLEC12, HSP70, IGHG1, Cyclophilin E, ARID4A, CLEC2D, eEF2K, LOC646817, SET, Follistatin, TXNRD1, SMYD3, Calmodulin, HSPA2, HSPA1A, RBP1, eEF1A1, CKS2, OR10G3, OBFC1, ZNF397OS, DYNLL, ICMT, ZNF397, SET/NUP214 fusion protein, Cyclin B, EYA1, GMEB1, Pleckstrin, PTPN2, FOP, RIG-G, PDXP, CAMK2N1, PP2C, HSC70, TARDBP (TDP43), PRCC, RUVBL1, PQBP-1, VILIP1
375	positive regulation of ubiquitin-protein ligase activity involved in mitotic cell cycle	88	7.077E-03	4.561E-02	7.077E-03	4.561E-02	3	Cyclin B1, PSMD10 (Gankyrin), Cyclin B
376	positive regulation of growth	270	7.086E-03	4.561E-02	7.086E-03	4.561E-02	5	PSMD10 (Gankyrin), PPM1F, Calmyrin, FOP, PP2C
377	cellular amino acid metabolic process	506	7.305E-03	4.650E-02	7.305E-03	4.650E-02	7	P5CR1, CDO1, DARS, COASY, TXNRD1, ICMT, Fructosamine-3-kinase
378	protein-DNA complex disassembly	29	7.321	4.650	7.321	4.650	2	SET, SET/NUP214 fusion protein

			E-03	E-02	E-03	E-02		
379	chromatin disassembly	29	7.321E-03	4.650E-02	7.321E-03	4.650E-02	2	SET, SET/NUP214 fusion protein
380	nucleosome disassembly	29	7.321E-03	4.650E-02	7.321E-03	4.650E-02	2	SET, SET/NUP214 fusion protein
381	oocyte maturation	29	7.321E-03	4.650E-02	7.321E-03	4.650E-02	2	Cyclin B1, Cyclin B
382	regulation of cysteine-type endopeptidase activity	274	7.527E-03	4.757E-02	7.527E-03	4.757E-02	5	HSPA1B, PPM1F, HSP70, HSPA1A, PP2C
383	regulation of cell-matrix adhesion	90	7.529E-	4.757E-	7.529E-	4.757E-	3	PPM1F, Calmyrin, PP2C

			03	02	03	02		
384	histone H3 deacetylation	30	7.821E-03	4.866E-02	7.821E-03	4.866E-02	2	HDAC7, Histone deacetylase class II
385	negative regulation of striated muscle tissue development	30	7.821E-03	4.866E-02	7.821E-03	4.866E-02	2	HDAC7, Histone deacetylase class II
386	protein sumoylation	30	7.821E-03	4.866E-02	7.821E-03	4.866E-02	2	SAE2, EYA1
387	positive regulation of mRNA processing	30	7.821E-03	4.866E-02	7.821E-03	4.866E-02	2	Cyclin B1, Cyclin B
388	establishment of chromosome localization	30	7.821E-03	4.866E-02	7.821E-03	4.866E-02	2	Cyclin B1, Cyclin B

389	histone phosphorylation	30	7.821E-03	4.866E-02	7.821E-03	4.866E-02	2	Cyclin B1, Cyclin B
390	DNA damage checkpoint	176	7.866E-03	4.881E-02	7.866E-03	4.881E-02	4	RBBP8 (CtIP), PSMD10 (Gankyrin), TIPIN, PP2C
391	heart development	646	8.004E-03	4.954E-02	8.004E-03	4.954E-02	8	Reticulon 4, Cyclin B1, ECE1, HEXIM1, Histone deacetylase class II, Cyclin B, EYA1, PP2C

Table 9S: Enrichment analysis report. Enrichment by GO Processes (SVZp vs SVZn)								
Enrichment by GO Processes					SVZp VS SVZn Limma List_genelist			
#	Processes	Total	pValue	Min FDR	p-value	FDR	In Data	Network Objects from Active Data
1	hexose catabolic process	39	8.005E-06	3.021E-03	8.005E-06	3.021E-03	3	PGMU, PGM2, 3HIDH
2	monosaccharide catabolic process	45	1.238E-05	3.021E-03	1.238E-05	3.021E-03	3	PGMU, PGM2, 3HIDH
3	galactose catabolic process	8	2.728E-05	4.438E-03	2.728E-05	4.438E-03	2	PGMU, PGM2
4	positive regulation of guanylate cyclase activity	14	8.834E-05	9.464E-03	8.834E-05	9.464E-03	2	GCAP, GCAP1
5	regulation of guanylate cyclase activity	15	1.019E-04	9.464E-03	1.019E-04	9.464E-03	2	GCAP, GCAP1
6	galactose metabolic process	16	1.164E-04	9.464E-03	1.164E-04	9.464E-03	2	PGMU, PGM2
7	glycogen catabolic process	25	2.893E-04	1.660E-02	2.893E-04	1.660E-02	2	PGMU, PGM2
8	glucan catabolic process	26	3.132E-04	1.660E-02	3.132E-04	1.660E-02	2	PGMU, PGM2
9	cellular polysaccharide catabolic process	27	3.381E-04	1.660E-02	3.381E-04	1.660E-02	2	PGMU, PGM2
10	single-organism carbohydrate catabolic process	137	3.445E-04	1.660E-02	3.445E-04	1.660E-02	3	PGMU, PGM2, 3HIDH
11	polysaccharide catabolic process	30	4.182E-04	1.660E-02	4.182E-04	1.660E-02	2	PGMU, PGM2
12	carbohydrate catabolic process	147	4.234E-04	1.660E-02	4.234E-04	1.660E-02	3	PGMU, PGM2, 3HIDH
13	cellular response to epidermal growth factor stimulus	31	4.468E-04	1.660E-02	4.468E-04	1.660E-02	2	eEF1A1, eEF1A
14	positive regulation of cGMP biosynthetic	32	4.763E	1.660E	4.763E	1.660E	2	GCAP, GCAP1

	process		-04	-02	-04	-02		
15	glycogen biosynthetic process	35	5.703E-04	1.728E-02	5.703E-04	1.728E-02	2	PGMU, PGM2
16	glucan biosynthetic process	35	5.703E-04	1.728E-02	5.703E-04	1.728E-02	2	PGMU, PGM2
17	positive regulation of cGMP metabolic process	36	6.034E-04	1.728E-02	6.034E-04	1.728E-02	2	GCAP, GCAP1
18	cellular carbohydrate catabolic process	37	6.375E-04	1.728E-02	6.375E-04	1.728E-02	2	PGMU, PGM2
19	response to epidermal growth factor	42	8.217E-04	2.005E-02	8.217E-04	2.005E-02	2	eEF1A1, eEF1A
20	regulation of cGMP biosynthetic process	42	8.217E-04	2.005E-02	8.217E-04	2.005E-02	2	GCAP, GCAP1
21	glucose metabolic process	208	1.160E-03	2.581E-02	1.160E-03	2.581E-02	3	PGMU, PGM2, 3HIDH
22	regulation of cGMP metabolic process	50	1.163E-03	2.581E-02	1.163E-03	2.581E-02	2	GCAP, GCAP1
23	regulation of rhodopsin mediated signaling pathway	55	1.406E-03	2.983E-02	1.406E-03	2.983E-02	2	GCAP, GCAP1
24	cellular polysaccharide biosynthetic process	57	1.509E-03	3.049E-02	1.509E-03	3.049E-02	2	PGMU, PGM2
25	polysaccharide biosynthetic process	58	1.562E-03	3.049E-02	1.562E-03	3.049E-02	2	PGMU, PGM2
26	rhodopsin mediated signaling pathway	62	1.783E-03	3.346E-02	1.783E-03	3.346E-02	2	GCAP, GCAP1
27	hexose metabolic process	251	1.987E-03	3.530E-02	1.987E-03	3.530E-02	3	PGMU, PGM2, 3HIDH
28	trehalose biosynthetic process	2	2.026E-03	3.530E-02	2.026E-03	3.530E-02	1	PGMU
29	cellular carbohydrate biosynthetic process	70	2.266E-03	3.813E-02	2.266E-03	3.813E-02	2	PGMU, PGM2

30	positive regulation of cyclase activity	76	2.664E-03	4.334E-02	2.664E-03	4.334E-02	2	GCAP, GCAP1
31	positive regulation of lyase activity	78	2.804E-03	4.377E-02	2.804E-03	4.377E-02	2	GCAP, GCAP1
32	trehalose metabolic process	3	3.037E-03	4.377E-02	3.037E-03	4.377E-02	1	PGMU
33	cellular macromolecule biosynthetic process	4001	3.091E-03	4.377E-02	3.091E-03	4.377E-02	10	eEF1A1, eEF1A, MJD (ataxin-3), PGMU, KIAA1838, DR1, PGM2, GABP alpha, GMEB1, Gpbp1
34	glycogen metabolic process	84	3.243E-03	4.377E-02	3.243E-03	4.377E-02	2	PGMU, PGM2
35	monosaccharide metabolic process	299	3.262E-03	4.377E-02	3.262E-03	4.377E-02	3	PGMU, PGM2, 3HIDH
36	cellular glucan metabolic process	85	3.319E-03	4.377E-02	3.319E-03	4.377E-02	2	PGMU, PGM2
37	glucan metabolic process	85	3.319E-03	4.377E-02	3.319E-03	4.377E-02	2	PGMU, PGM2
38	macromolecule biosynthetic process	4060	3.460E-03	4.443E-02	3.460E-03	4.443E-02	10	eEF1A1, eEF1A, MJD (ataxin-3), PGMU, KIAA1838, DR1, PGM2, GABP alpha, GMEB1, Gpbp1

**Table 10S: Enrichment analysis report.** Enrichment by Diseases (by Biomarkers) (healthy control vs Grade II)								
Enrichment by Diseases (by Biomarkers)					GRADE II_INPUT_genelist			
**#**	**Diseases**	**Total**	**pValue**	**Min FDR**	**p-value**	**FDR**	**In Data**	**Network Objects from Active Data**
1	Papilloma, Intraductal	4	1.934E-13	5.590E-11	1.934E-13	5.590E-11	4	CD44 (EXT), CD44, CD44 soluble, CD44 (ICD)
2	Lymphangiomyoma	11	6.363E-11	6.130E-09	6.363E-11	6.130E-09	4	CD44 (EXT), CD44, CD44 soluble, CD44 (ICD)
3	Lymphangioleiomyomatosis	11	6.363E-11	6.130E-09	6.363E-11	6.130E-09	4	CD44 (EXT), CD44, CD44 soluble, CD44 (ICD)
4	Smooth Muscle Tumor	12	9.540E-11	6.893E-09	9.540E-11	6.893E-09	4	CD44 (EXT), CD44, CD44 soluble, CD44 (ICD)
5	Lymphatic Vessel Tumors	13	1.377E-10	7.962E-09	1.377E-10	7.962E-09	4	CD44 (EXT), CD44, CD44 soluble, CD44 (ICD)
6	Arthritis, Experimental	17	4.577E-10	2.205E-08	4.577E-10	2.205E-08	4	CD44 (EXT), CD44, CD44 soluble, CD44 (ICD)
7	Keratoacanthoma	19	7.447E-10	3.075E-08	7.447E-10	3.075E-08	4	CD44 (EXT), CD44, CD44 soluble, CD44 (ICD)
8	Lymphatic Metastasis	21	1.149E-09	4.151E-08	1.149E-09	4.151E-08	4	CD44 (EXT), CD44, CD44 soluble, CD44 (ICD)
9	Perivascular Epithelioid Cell Neoplasms	22	1.404E-09	4.507E-08	1.404E-09	4.507E-08	4	CD44 (EXT), CD44, CD44 soluble, CD44 (ICD)
10	Leukemia, Erythroblastic, Acute	27	3.360E-	9.711E-	3.360E-	9.711E-	4	CD44 (EXT), CD44, CD44 soluble, CD44 (ICD)

			09	08	09	08		
11	Leukemia, Monocytic, Acute	28	3.919E-09	1.030E-07	3.919E-09	1.030E-07	4	CD44 (EXT), CD44, CD44 soluble, CD44 (ICD)
12	Acute Lung Injury	6	6.478E-09	1.440E-07	6.478E-09	1.440E-07	3	MYLK1, MLCK, Telokin
13	Rhabdomyolysis	6	6.478E-09	1.440E-07	6.478E-09	1.440E-07	3	MYLK1, MLCK, Telokin
14	Lichen Planus, Oral	36	1.123E-08	2.182E-07	1.123E-08	2.182E-07	4	CD44 (EXT), CD44, CD44 soluble, CD44 (ICD)
15	Craniofacial Abnormalities	237	1.133E-08	2.182E-07	1.133E-08	2.182E-07	6	CD44 (EXT), CD44, CD44 soluble, CD44 (ICD), PQBP-1, EYA1
16	Leukemia, Plasma Cell	45	2.830E-08	5.112E-07	2.830E-08	5.112E-07	4	CD44 (EXT), CD44, CD44 soluble, CD44 (ICD)
17	Lichen Planus	51	4.734E-08	8.049E-07	4.734E-08	8.049E-07	4	CD44 (EXT), CD44, CD44 soluble, CD44 (ICD)
18	Lichenoid Eruptions	56	6.943E-08	1.115E-06	6.943E-08	1.115E-06	4	CD44 (EXT), CD44, CD44 soluble, CD44 (ICD)
19	Musculoskeletal Abnormalities	340	9.665E-08	1.470E-06	9.665E-08	1.470E-06	6	CD44 (EXT), CD44, CD44 soluble, CD44 (ICD), PQBP-1, EYA1
20	Papilloma	63	1.123E-07	1.622E-06	1.123E-07	1.622E-06	4	CD44 (EXT), CD44, CD44 soluble, CD44 (ICD)
21	Carcinoma, Transitional Cell	68	1.531E-07	2.107E-06	1.531E-07	2.107E-06	4	CD44 (EXT), CD44, CD44 soluble, CD44 (ICD)

22	Gastrointestinal Stromal Tumors	69	1.625E-07	2.134E-06	1.625E-07	2.134E-06	4	CD44 (EXT), CD44, CD44 soluble, CD44 (ICD)
23	Fractures, Bone	79	2.812E-07	3.533E-06	2.812E-07	3.533E-06	4	CD44 (EXT), CD44, CD44 soluble, CD44 (ICD)
24	Adenomatous Polyposis Coli	82	3.269E-07	3.936E-06	3.269E-07	3.936E-06	4	CD44 (EXT), CD44, CD44 soluble, CD44 (ICD)
25	Intestinal Polyposis	90	4.758E-07	5.501E-06	4.758E-07	5.501E-06	4	CD44 (EXT), CD44, CD44 soluble, CD44 (ICD)
26	Adenomatous Polyps	92	5.199E-07	5.565E-06	5.199E-07	5.565E-06	4	CD44 (EXT), CD44, CD44 soluble, CD44 (ICD)
27	Wilms Tumor	92	5.199E-07	5.565E-06	5.199E-07	5.565E-06	4	CD44 (EXT), CD44, CD44 soluble, CD44 (ICD)
28	Aortic Aneurysm, Abdominal	98	6.703E-07	6.919E-06	6.703E-07	6.919E-06	4	CD44 (EXT), CD44, CD44 soluble, CD44 (ICD)
29	Thyroid Carcinoma, Follicular	111	1.105E-06	1.101E-05	1.105E-06	1.101E-05	4	CD44 (EXT), CD44, CD44 soluble, CD44 (ICD)
30	Hodgkin Disease	113	1.187E-06	1.143E-05	1.187E-06	1.143E-05	4	CD44 (EXT), CD44, CD44 soluble, CD44 (ICD)
31	Epstein-Barr Virus Infections	119	1.460E-06	1.361E-05	1.460E-06	1.361E-05	4	CD44 (EXT), CD44, CD44 soluble, CD44 (ICD)
32	Inflammation	891	1.540E-06	1.391E-05	1.540E-06	1.391E-05	7	CD44 (EXT), CD44, CD44 soluble, MYLK1, MLCK, CD44 (ICD), Telokin
33	Musculoskeletal Diseases	251	1.675E-	1.467E-	1.675E-	1.467E-	10	CD44 (EXT), CD44, CD44 soluble, IGHG1, MYLK1, MLCK, CD44 (ICD), PQBP-1, EYA1, Telokin

		1	06	05	06	05		
34	Aortic Aneurysm	129	2.015E-06	1.713E-05	2.015E-06	1.713E-05	4	CD44 (EXT), CD44, CD44 soluble, CD44 (ICD)
35	Colorectal Neoplasms, Hereditary Nonpolyposis	137	2.562E-06	2.115E-05	2.562E-06	2.115E-05	4	CD44 (EXT), CD44, CD44 soluble, CD44 (ICD)
36	Leukemia, Promyelocytic, Acute	146	3.300E-06	2.577E-05	3.300E-06	2.577E-05	4	CD44 (EXT), CD44, CD44 soluble, CD44 (ICD)
37	Neoplasms, Experimental	146	3.300E-06	2.577E-05	3.300E-06	2.577E-05	4	CD44 (EXT), CD44, CD44 soluble, CD44 (ICD)
38	Hypersensitivity	1499	3.830E-06	2.913E-05	3.830E-06	2.913E-05	8	CD44 (EXT), CD44, CD44 soluble, IGHG1, MYLK1, MLCK, CD44 (ICD), Telokin
39	Leukemia-Lymphoma, Adult T-Cell	154	4.079E-06	3.023E-05	4.079E-06	3.023E-05	4	CD44 (EXT), CD44, CD44 soluble, CD44 (ICD)
40	Aortic Diseases	163	5.111E-06	3.692E-05	5.111E-06	3.692E-05	4	CD44 (EXT), CD44, CD44 soluble, CD44 (ICD)
41	Eosinophilia	49	5.853E-06	4.126E-05	5.853E-06	4.126E-05	3	MYLK1, MLCK, Telokin
42	Celiac Disease	177	7.083E-06	4.874E-05	7.083E-06	4.874E-05	4	CD44 (EXT), CD44, CD44 soluble, CD44 (ICD)
43	Asthma	1128	7.381E-06	4.961E-05	7.381E-06	4.961E-05	7	CD44 (EXT), CD44, CD44 soluble, MYLK1, MLCK, CD44 (ICD), Telokin
44	Respiratory Hypersensitivity	1143	8.052E-06	5.289E-05	8.052E-06	5.289E-05	7	CD44 (EXT), CD44, CD44 soluble, MYLK1, MLCK, CD44 (ICD), Telokin

45	Aneurysm	185	8.435E-06	5.417E-05	8.435E-06	5.417E-05	4	CD44 (EXT), CD44, CD44 soluble, CD44 (ICD)
46	Infection	1170	9.390E-06	5.814E-05	9.390E-06	5.814E-05	7	CD44 (EXT), CD44, CD44 soluble, MYLK1, MLCK, CD44 (ICD), Telokin
47	Biliary Tract Neoplasms	191	9.569E-06	5.814E-05	9.569E-06	5.814E-05	4	CD44 (EXT), CD44, CD44 soluble, CD44 (ICD)
48	Bronchial Diseases	1175	9.657E-06	5.814E-05	9.657E-06	5.814E-05	7	CD44 (EXT), CD44, CD44 soluble, MYLK1, MLCK, CD44 (ICD), Telokin
49	Leukemia, T-Cell	200	1.147E-05	6.768E-05	1.147E-05	6.768E-05	4	CD44 (EXT), CD44, CD44 soluble, CD44 (ICD)
50	Colonic Neoplasms	1232	1.318E-05	7.618E-05	1.318E-05	7.618E-05	7	CD44 (EXT), CD44, CD44 soluble, IGHG1, NOL3, CD44 (ICD), SNX1
51	Respiratory Distress Syndrome, Adult	65	1.378E-05	7.808E-05	1.378E-05	7.808E-05	3	MYLK1, MLCK, Telokin
52	Tumor Virus Infections	213	1.470E-05	8.172E-05	1.470E-05	8.172E-05	4	CD44 (EXT), CD44, CD44 soluble, CD44 (ICD)
53	Bacterial Infections and Mycoses	1294	1.817E-05	9.907E-05	1.817E-05	9.907E-05	7	CD44 (EXT), CD44, CD44 soluble, MYLK1, MLCK, CD44 (ICD), Telokin
54	Prostatic Intraepithelial Neoplasia	226	1.856E-05	9.933E-05	1.856E-05	9.933E-05	4	CD44 (EXT), CD44, CD44 soluble, CD44 (ICD)
55	Hypersensitivity, Immediate	1325	2.120E-05	1.114E-04	2.120E-05	1.114E-04	7	CD44 (EXT), CD44, CD44 soluble, MYLK1, MLCK, CD44 (ICD), Telokin
56	Malabsorption Syndromes	235	2.163E-	1.116E-	2.163E-	1.116E-	4	CD44 (EXT), CD44, CD44 soluble, CD44 (ICD)

			05	04	05	04		
57	Dermatitis, Allergic Contact	242	2.427E-05	1.229E-04	2.427E-05	1.229E-04	4	CD44 (EXT), CD44, CD44 soluble, CD44 (ICD)
58	Hypersensitivity, Delayed	243	2.467E-05	1.229E-04	2.467E-05	1.229E-04	4	CD44 (EXT), CD44, CD44 soluble, CD44 (ICD)
59	Carcinoma, Pancreatic Ductal	519	2.636E-05	1.291E-04	2.636E-05	1.291E-04	5	CD44 (EXT), CD44, CD44 soluble, CD44 (ICD), LHX1
60	Dermatitis, Contact	257	3.071E-05	1.479E-04	3.071E-05	1.479E-04	4	CD44 (EXT), CD44, CD44 soluble, CD44 (ICD)
61	Congenital Abnormalities	1536	5.517E-05	2.614E-04	5.517E-05	2.614E-04	7	CD44 (EXT), CD44, CD44 soluble, CD44 (ICD), PQBP-1, LHX1, EYA1
62	Lung Injury	104	5.645E-05	2.631E-04	5.645E-05	2.631E-04	3	MYLK1, MLCK, Telokin
63	Neoplasms, Muscle Tissue	304	5.910E-05	2.711E-04	5.910E-05	2.711E-04	4	CD44 (EXT), CD44, CD44 soluble, CD44 (ICD)
64	DNA Repair-Deficiency Disorders	308	6.218E-05	2.808E-04	6.218E-05	2.808E-04	4	CD44 (EXT), CD44, CD44 soluble, CD44 (ICD)
65	Oligodendroglioma	313	6.619E-05	2.943E-04	6.619E-05	2.943E-04	4	CD44 (EXT), CD44, CD44 soluble, CD44 (ICD)
66	Lung Diseases, Obstructive	1588	6.831E-05	2.991E-04	6.831E-05	2.991E-04	7	CD44 (EXT), CD44, CD44 soluble, MYLK1, MLCK, CD44 (ICD), Telokin
67	Stomach Neoplasms	3793	7.222E-05	3.115E-04	7.222E-05	3.115E-04	10	CD44 (EXT), CD44, CD44 soluble, VDAC 1, IGHG1, MYLK1, MLCK, CD44 (ICD), LHX1, Telokin

68	Myeloproliferative Disorders	322	7.389E-05	3.140E-04	7.389E-05	3.140E-04	4	CD44 (EXT), CD44, CD44 soluble, CD44 (ICD)
69	Stomach Diseases	3840	8.058E-05	3.375E-04	8.058E-05	3.375E-04	10	CD44 (EXT), CD44, CD44 soluble, VDAC 1, IGHG1, MYLK1, MLCK, CD44 (ICD), LHX1, Telokin
70	Endocrine Gland Neoplasms	4811	8.510E-05	3.513E-04	8.510E-05	3.513E-04	11	CD44 (EXT), CD44, CD44 soluble, IGHG1, MYLK1, NOL3, MLCK, CCDC32, CD44 (ICD), LHX1, Telokin
71	Neoplasms, Complex and Mixed	339	9.017E-05	3.670E-04	9.017E-05	3.670E-04	4	CD44 (EXT), CD44, CD44 soluble, CD44 (ICD)
72	Endometriosis	356	1.089E-04	4.373E-04	1.089E-04	4.373E-04	4	CD44 (EXT), CD44, CD44 soluble, CD44 (ICD)
73	Osteosarcoma	360	1.137E-04	4.503E-04	1.137E-04	4.503E-04	4	CD44 (EXT), CD44, CD44 soluble, CD44 (ICD)
74	Neoplasms, Bone Tissue	369	1.251E-04	4.871E-04	1.251E-04	4.871E-04	4	CD44 (EXT), CD44, CD44 soluble, CD44 (ICD)
75	Carcinoma in Situ	370	1.264E-04	4.871E-04	1.264E-04	4.871E-04	4	CD44 (EXT), CD44, CD44 soluble, CD44 (ICD)
76	Sclerosis	375	1.331E-04	5.062E-04	1.331E-04	5.062E-04	4	CD44 (EXT), CD44, CD44 soluble, CD44 (ICD)
77	Thyroid Carcinoma, Papillary	377	1.359E-04	5.099E-04	1.359E-04	5.099E-04	4	CD44 (EXT), CD44, CD44 soluble, CD44 (ICD)
78	Leukocyte Disorders	142	1.424E-04	5.275E-04	1.424E-04	5.275E-04	3	MYLK1, MLCK, Telokin
79	Herpesviridae Infections	391	1.563E-	5.718E-	1.563E-	5.718E-	4	CD44 (EXT), CD44, CD44 soluble, CD44 (ICD)

			04	04	04	04		
80	Peripheral Nervous System Neoplasms	406	1.806E-04	6.505E-04	1.806E-04	6.505E-04	4	CD44 (EXT), CD44, CD44 soluble, CD44 (ICD)
81	Biliary Tract Diseases	407	1.823E-04	6.505E-04	1.823E-04	6.505E-04	4	CD44 (EXT), CD44, CD44 soluble, CD44 (ICD)
82	Small Cell Lung Carcinoma	446	2.587E-04	9.119E-04	2.587E-04	9.119E-04	4	CD44 (EXT), CD44, CD44 soluble, CD44 (ICD)
83	Skin Diseases, Papulosquamous	453	2.746E-04	9.560E-04	2.746E-04	9.560E-04	4	CD44 (EXT), CD44, CD44 soluble, CD44 (ICD)
84	Arthritis, Rheumatoid	1367	2.787E-04	9.590E-04	2.787E-04	9.590E-04	6	CD44 (EXT), CD44, CD44 soluble, IGHG1, CD44 (ICD), EYA1
85	Carcinoma, Ductal	1414	3.350E-04	1.139E-03	3.350E-04	1.139E-03	6	CD44 (EXT), CD44, CD44 soluble, NOL3, CD44 (ICD), LHX1
86	Respiration Disorders	191	3.408E-04	1.145E-03	3.408E-04	1.145E-03	3	MYLK1, MLCK, Telokin
87	Neoplastic Syndromes, Hereditary	489	3.672E-04	1.220E-03	3.672E-04	1.220E-03	4	CD44 (EXT), CD44, CD44 soluble, CD44 (ICD)
88	Connective Tissue Diseases	2088	3.840E-04	1.249E-03	3.840E-04	1.249E-03	7	CD44 (EXT), CD44, CD44 soluble, VDAC 1, IGHG1, CD44 (ICD), EYA1
89	Esophageal Neoplasms	495	3.846E-04	1.249E-03	3.846E-04	1.249E-03	4	CD44 (EXT), CD44, CD44 soluble, CD44 (ICD)
90	Hematologic Diseases	2844	4.027E-04	1.293E-03	4.027E-04	1.293E-03	8	CD44 (EXT), CD44, CD44 soluble, IGHG1, MYLK1, MLCK, CD44 (ICD), Telokin

91	Neoplasms, Ductal, Lobular, and Medullary	1467	4.089E-04	1.299E-03	4.089E-04	1.299E-03	6	CD44 (EXT), CD44, CD44 soluble, NOL3, CD44 (ICD), LHX1
92	Crohn Disease	506	4.180E-04	1.313E-03	4.180E-04	1.313E-03	4	CD44 (EXT), CD44, CD44 soluble, CD44 (ICD)
93	Neuroblastoma	520	4.635E-04	1.440E-03	4.635E-04	1.440E-03	4	CD44 (EXT), CD44, CD44 soluble, CD44 (ICD)
94	Rheumatic Diseases	1509	4.762E-04	1.453E-03	4.762E-04	1.453E-03	6	CD44 (EXT), CD44, CD44 soluble, IGHG1, CD44 (ICD), EYA1
95	Endocrine System Diseases	5758	4.777E-04	1.453E-03	4.777E-04	1.453E-03	11	CD44 (EXT), CD44, CD44 soluble, IGHG1, MYLK1, NOL3, MLCK, CCDC32, CD44 (ICD), LHX1, Telokin
96	Skin Diseases, Eczematous	529	4.946E-04	1.489E-03	4.946E-04	1.489E-03	4	CD44 (EXT), CD44, CD44 soluble, CD44 (ICD)
97	Neuroectodermal Tumors, Primitive, Peripheral	531	5.017E-04	1.495E-03	5.017E-04	1.495E-03	4	CD44 (EXT), CD44, CD44 soluble, CD44 (ICD)
98	Nerve Sheath Neoplasms	533	5.088E-04	1.501E-03	5.088E-04	1.501E-03	4	CD44 (EXT), CD44, CD44 soluble, CD44 (ICD)
99	Wounds and Injuries	982	5.289E-04	1.544E-03	5.289E-04	1.544E-03	5	CD44 (EXT), CD44, CD44 soluble, MLCK, CD44 (ICD)
100	Ovarian Neoplasms	3027	6.207E-04	1.794E-03	6.207E-04	1.794E-03	8	CD44 (EXT), CD44, CD44 soluble, MYLK1, NOL3, MLCK, CD44 (ICD), Telokin
101	Dermatitis	566	6.380E-04	1.826E-03	6.380E-04	1.826E-03	4	CD44 (EXT), CD44, CD44 soluble, CD44 (ICD)
102	Arthritis	159	6.455E-	1.826E-	6.455E-	1.826E-	6	CD44 (EXT), CD44, CD44 soluble, IGHG1, CD44 (ICD), EYA1

		7	04	03	04	03		
103	Esophageal Diseases	569	6.508E-04	1.826E-03	6.508E-04	1.826E-03	4	CD44 (EXT), CD44, CD44 soluble, CD44 (ICD)
104	Laryngeal Neoplasms	2291	6.797E-04	1.889E-03	6.797E-04	1.889E-03	7	CD44 (EXT), CD44, CD44 soluble, MYLK1, MLCK, CD44 (ICD), Telokin
105	Laryngeal Diseases	2308	7.111E-04	1.926E-03	7.111E-04	1.926E-03	7	CD44 (EXT), CD44, CD44 soluble, MYLK1, MLCK, CD44 (ICD), Telokin
106	Adenoma	583	7.130E-04	1.926E-03	7.130E-04	1.926E-03	4	CD44 (EXT), CD44, CD44 soluble, CD44 (ICD)
107	Joint Diseases	1627	7.130E-04	1.926E-03	7.130E-04	1.926E-03	6	CD44 (EXT), CD44, CD44 soluble, IGHG1, CD44 (ICD), EYA1
108	Retrognathia	1	7.339E-04	1.946E-03	7.339E-04	1.946E-03	1	EYA1
109	Funnel Chest	1	7.339E-04	1.946E-03	7.339E-04	1.946E-03	1	EYA1
110	Carcinoma, Squamous Cell	595	7.696E-04	2.022E-03	7.696E-04	2.022E-03	4	CD44 (EXT), CD44, CD44 soluble, CD44 (ICD)
111	Ovarian Diseases	3139	7.969E-04	2.070E-03	7.969E-04	2.070E-03	8	CD44 (EXT), CD44, CD44 soluble, MYLK1, NOL3, MLCK, CD44 (ICD), Telokin
112	Adnexal Diseases	3142	8.022E-04	2.070E-03	8.022E-04	2.070E-03	8	CD44 (EXT), CD44, CD44 soluble, MYLK1, NOL3, MLCK, CD44 (ICD), Telokin
113	Sepsis	260	8.370E-04	2.141E-03	8.370E-04	2.141E-03	3	MYLK1, MLCK, Telokin

114	Systemic Inflammatory Response Syndrome	263	8.653E-04	2.194E-03	8.653E-04	2.194E-03	3	MYLK1, MLCK, Telokin
115	Carcinoma, Ductal, Breast	1108	9.170E-04	2.304E-03	9.170E-04	2.304E-03	5	CD44 (EXT), CD44, CD44 soluble, NOL3, CD44 (ICD)
116	Gonadal Disorders	3221	9.506E-04	2.368E-03	9.506E-04	2.368E-03	8	CD44 (EXT), CD44, CD44 soluble, MYLK1, NOL3, MLCK, CD44 (ICD), Telokin
117	Peripheral Nervous System Diseases	649	1.064E-03	2.629E-03	1.064E-03	2.629E-03	4	CD44 (EXT), CD44, CD44 soluble, CD44 (ICD)
118	Neoplasms, Squamous Cell	653	1.089E-03	2.665E-03	1.089E-03	2.665E-03	4	CD44 (EXT), CD44, CD44 soluble, CD44 (ICD)
119	Pancreatic Neoplasms	2479	1.097E-03	2.665E-03	1.097E-03	2.665E-03	7	CD44 (EXT), CD44, CD44 soluble, IGHG1, CCDC32, CD44 (ICD), LHX1
120	Lupus Erythematosus, Systemic	1161	1.132E-03	2.727E-03	1.132E-03	2.727E-03	5	CD44 (EXT), CD44, CD44 soluble, VDAC 1, CD44 (ICD)
121	Neuroectodermal Tumors, Primitive	3333	1.199E-03	2.864E-03	1.199E-03	2.864E-03	8	CD44 (EXT), CD44, CD44 soluble, MYLK1, MLCK, CD44 (ICD), LHX1, Telokin
122	Neoplasm Metastasis	676	1.238E-03	2.933E-03	1.238E-03	2.933E-03	4	CD44 (EXT), CD44, CD44 soluble, CD44 (ICD)
123	Neoplastic Processes	728	1.628E-03	3.826E-03	1.628E-03	3.826E-03	4	CD44 (EXT), CD44, CD44 soluble, CD44 (ICD)
124	DNA Virus Infections	731	1.653E-03	3.853E-03	1.653E-03	3.853E-03	4	CD44 (EXT), CD44, CD44 soluble, CD44 (ICD)
125	Pathologic Processes	267	1.742E-	4.028E-	1.742E-	4.028E-	7	CD44 (EXT), CD44, CD44 soluble, MYLK1, MLCK, CD44 (ICD), Telokin

		8	03	03	03	03		
126	Leukemia, Myeloid, Acute	1292	1.827E-03	4.191E-03	1.827E-03	4.191E-03	5	CD44 (EXT), CD44, CD44 soluble, MLCK, CD44 (ICD)
127	Melanoma, Cutaneous Malignant	763	1.935E-03	4.403E-03	1.935E-03	4.403E-03	4	CD44 (EXT), CD44, CD44 soluble, CD44 (ICD)
128	Autoimmune Diseases	2751	2.043E-03	4.613E-03	2.043E-03	4.613E-03	7	CD44 (EXT), CD44, CD44 soluble, VDAC 1, IGHG1, CD44 (ICD), EYA1
129	Bone Marrow Diseases	785	2.147E-03	4.811E-03	2.147E-03	4.811E-03	4	CD44 (EXT), CD44, CD44 soluble, CD44 (ICD)
130	Thyroid Neoplasms	787	2.167E-03	4.818E-03	2.167E-03	4.818E-03	4	CD44 (EXT), CD44, CD44 soluble, CD44 (ICD)
131	Branchio-Oto-Renal Syndrome	3	2.200E-03	4.854E-03	2.200E-03	4.854E-03	1	EYA1
132	Stomatognathic Diseases	5821	2.809E-03	6.151E-03	2.809E-03	6.151E-03	10	CD44 (EXT), CD44, CD44 soluble, IGHG1, MYLK1, MLCK, CD44 (ICD), EYA1, Telokin, SNX1
133	Mouth Diseases	4776	2.887E-03	6.273E-03	2.887E-03	6.273E-03	9	CD44 (EXT), CD44, CD44 soluble, IGHG1, MYLK1, MLCK, CD44 (ICD), Telokin, SNX1
134	Neoplasms, Connective Tissue	854	2.918E-03	6.294E-03	2.918E-03	6.294E-03	4	CD44 (EXT), CD44, CD44 soluble, CD44 (ICD)
135	Pulmonary Disease, Chronic Obstructive	864	3.044E-03	6.516E-03	3.044E-03	6.516E-03	4	CD44 (EXT), CD44, CD44 soluble, CD44 (ICD)
136	Pancreatic Diseases	2980	3.265E-03	6.938E-03	3.265E-03	6.938E-03	7	CD44 (EXT), CD44, CD44 soluble, IGHG1, CCDC32, CD44 (ICD), LHX1

137	Leukemia, Myeloid	1488	3.403E-03	7.178E-03	3.403E-03	7.178E-03	5	CD44 (EXT), CD44, CD44 soluble, MLCK, CD44 (ICD)
138	Virus Diseases	1506	3.586E-03	7.509E-03	3.586E-03	7.509E-03	5	CD44 (EXT), CD44, CD44 soluble, VDAC 1, CD44 (ICD)
139	Immune System Diseases	6037	3.771E-03	7.839E-03	3.771E-03	7.839E-03	10	CD44 (EXT), CD44, CD44 soluble, VDAC 1, IGHG1, MYLK1, MLCK, CD44 (ICD), EYA1, Telokin
140	Inflammatory Bowel Diseases	923	3.863E-03	7.974E-03	3.863E-03	7.974E-03	4	CD44 (EXT), CD44, CD44 soluble, CD44 (ICD)
141	Digestive System Neoplasms	11508	4.034E-03	8.269E-03	4.034E-03	8.269E-03	14	CD44 (EXT), CD44, CD44 soluble, VDAC 1, IGHG1, MYLK1, NOL3, MLCK, CCDC32, CD44 (ICD), LHX1, RRP42, Telokin, SNX1
142	Congenital, Hereditary, and Neonatal Diseases and Abnormalities	4013	4.106E-03	8.357E-03	4.106E-03	8.357E-03	8	CD44 (EXT), CD44, CD44 soluble, IGHG1, CD44 (ICD), PQBP-1, LHX1, EYA1
143	Thyroid Diseases	964	4.514E-03	9.122E-03	4.514E-03	9.122E-03	4	CD44 (EXT), CD44, CD44 soluble, CD44 (ICD)
144	Carcinoma, Renal Cell	1594	4.585E-03	9.201E-03	4.585E-03	9.201E-03	5	CD44 (EXT), CD44, CD44 soluble, CD44 (ICD), LHX1
145	Sarcoma	995	5.053E-03	1.007E-02	5.053E-03	1.007E-02	4	CD44 (EXT), CD44, CD44 soluble, CD44 (ICD)
146	Gastroenteritis	1014	5.405E-03	1.070E-02	5.405E-03	1.070E-02	4	CD44 (EXT), CD44, CD44 soluble, CD44 (ICD)
147	Multiple Sclerosis	1017	5.462E-03	1.074E-02	5.462E-03	1.074E-02	4	CD44 (EXT), CD44, CD44 soluble, CD44 (ICD)
14	Lymphoma, Non-Hodgkin	10	5.519	1.078	5.519	1.078	4	CD44 (EXT), CD44, CD44 soluble, CD44 (ICD)

8		20	E-03	E-02	E-03	E-02		
149	Gastrointestinal Neoplasms	10272	5.661E-03	1.093E-02	5.661E-03	1.093E-02	13	CD44 (EXT), CD44, CD44 soluble, VDAC 1, IGHG1, MYLK1, NOL3, MLCK, CD44 (ICD), LHX1, RRP42, Telokin, SNX1
150	Demyelinating Autoimmune Diseases, CNS	1028	5.674E-03	1.093E-02	5.674E-03	1.093E-02	4	CD44 (EXT), CD44, CD44 soluble, CD44 (ICD)
151	Mayer-Rokitansky-Kuster-Hauser Syndrome	8	5.857E-03	1.121E-02	5.857E-03	1.121E-02	1	LHX1
152	Mouth Neoplasms	4270	6.116E-03	1.159E-02	6.116E-03	1.159E-02	8	CD44 (EXT), CD44, CD44 soluble, MYLK1, MLCK, CD44 (ICD), Telokin, SNX1
153	Colonic Diseases	4272	6.135E-03	1.159E-02	6.135E-03	1.159E-02	8	CD44 (EXT), CD44, CD44 soluble, VDAC 1, IGHG1, NOL3, CD44 (ICD), SNX1
154	Digestive System Diseases	11984	6.562E-03	1.231E-02	6.562E-03	1.231E-02	14	CD44 (EXT), CD44, CD44 soluble, VDAC 1, IGHG1, MYLK1, NOL3, MLCK, CCDC32, CD44 (ICD), LHX1, RRP42, Telokin, SNX1
155	Pathological Conditions, Signs and Symptoms	4332	6.702E-03	1.250E-02	6.702E-03	1.250E-02	8	CD44 (EXT), CD44, CD44 soluble, MYLK1, MLCK, CD44 (ICD), PQBP-1, Telokin
156	Colorectal Neoplasms	9025	6.798E-03	1.258E-02	6.798E-03	1.258E-02	12	CD44 (EXT), CD44, CD44 soluble, VDAC 1, IGHG1, MYLK1, NOL3, MLCK, CD44 (ICD), RRP42, Telokin, SNX1
157	Autoimmune Diseases of the Nervous System	1084	6.843E-03	1.258E-02	6.843E-03	1.258E-02	4	CD44 (EXT), CD44, CD44 soluble, CD44 (ICD)
158	Intestinal Neoplasms	9036	6.878E-03	1.258E-02	6.878E-03	1.258E-02	12	CD44 (EXT), CD44, CD44 soluble, VDAC 1, IGHG1, MYLK1, NOL3, MLCK, CD44 (ICD), RRP42, Telokin, SNX1
159	Gastrointestinal Diseases	105	7.197E-	1.308E-	7.197E-	1.308E-	13	CD44 (EXT), CD44, CD44 soluble, VDAC 1, IGHG1, MYLK1, NOL3, MLCK, CD44 (ICD), LHX1, RRP42, Telokin, SNX1

		06	03	02	03	02		
160	Demyelinating Diseases	1105	7.321E-03	1.322E-02	7.321E-03	1.322E-02	4	CD44 (EXT), CD44, CD44 soluble, CD44 (ICD)
161	Genetic Diseases, Inborn	3437	7.373E-03	1.323E-02	7.373E-03	1.323E-02	7	CD44 (EXT), CD44, CD44 soluble, IGHG1, CD44 (ICD), PQBP-1, EYA1
162	Muscular Diseases	574	7.873E-03	1.405E-02	7.873E-03	1.405E-02	3	MYLK1, MLCK, Telokin
163	Hemic and Lymphatic Diseases	4547	9.087E-03	1.611E-02	9.087E-03	1.611E-02	8	CD44 (EXT), CD44, CD44 soluble, IGHG1, MYLK1, MLCK, CD44 (ICD), Telokin
164	Multiple Myeloma	1882	9.275E-03	1.634E-02	9.275E-03	1.634E-02	5	CD44 (EXT), CD44, CD44 soluble, IGHG1, CD44 (ICD)
165	Intestinal Diseases	9340	9.422E-03	1.637E-02	9.422E-03	1.637E-02	12	CD44 (EXT), CD44, CD44 soluble, VDAC 1, IGHG1, MYLK1, NOL3, MLCK, CD44 (ICD), RRP42, Telokin, SNX1
166	Neoplasms, Plasma Cell	1891	9.462E-03	1.637E-02	9.462E-03	1.637E-02	5	CD44 (EXT), CD44, CD44 soluble, IGHG1, CD44 (ICD)
167	Paraproteinemias	1891	9.462E-03	1.637E-02	9.462E-03	1.637E-02	5	CD44 (EXT), CD44, CD44 soluble, IGHG1, CD44 (ICD)
168	Neoplasms, Connective and Soft Tissue	1199	9.732E-03	1.674E-02	9.732E-03	1.674E-02	4	CD44 (EXT), CD44, CD44 soluble, CD44 (ICD)
169	Blood Protein Disorders	1922	1.012E-02	1.731E-02	1.012E-02	1.731E-02	5	CD44 (EXT), CD44, CD44 soluble, IGHG1, CD44 (ICD)
170	Hemostatic Disorders	1940	1.052E-02	1.789E-02	1.052E-02	1.789E-02	5	CD44 (EXT), CD44, CD44 soluble, IGHG1, CD44 (ICD)

171	Melanoma	3676	1.071E-02	1.809E-02	1.071E-02	1.809E-02	7	CD44 (EXT), CD44, CD44 soluble, MYLK1, MLCK, CD44 (ICD), Telokin
172	Nevi and Melanomas	3681	1.079E-02	1.812E-02	1.079E-02	1.812E-02	7	CD44 (EXT), CD44, CD44 soluble, MYLK1, MLCK, CD44 (ICD), Telokin
173	Lymphoma	1250	1.124E-02	1.877E-02	1.124E-02	1.877E-02	4	CD44 (EXT), CD44, CD44 soluble, CD44 (ICD)
174	Otorhinolaryngologic Neoplasms	3725	1.151E-02	1.902E-02	1.151E-02	1.902E-02	7	CD44 (EXT), CD44, CD44 soluble, MYLK1, MLCK, CD44 (ICD), Telokin
175	Neuromuscular Diseases	1259	1.152E-02	1.902E-02	1.152E-02	1.902E-02	4	CD44 (EXT), CD44, CD44 soluble, CD44 (ICD)
176	Hemorrhagic Disorders	2024	1.254E-02	2.058E-02	1.254E-02	2.058E-02	5	CD44 (EXT), CD44, CD44 soluble, IGHG1, CD44 (ICD)
177	Neuroendocrine Tumors	3813	1.307E-02	2.134E-02	1.307E-02	2.134E-02	7	CD44 (EXT), CD44, CD44 soluble, MYLK1, MLCK, CD44 (ICD), Telokin
178	Mandibular Diseases	20	1.458E-02	2.368E-02	1.458E-02	2.368E-02	1	EYA1
179	Skin and Connective Tissue Diseases	11328	1.581E-02	2.552E-02	1.581E-02	2.552E-02	13	CD44 (EXT), CD44, CD44 soluble, VDAC 1, IGHG1, MYLK1, NOL3, MLCK, CD44 (ICD), PQBP-1, EYA1, Telokin, SNX1
180	Glioma	5088	1.806E-02	2.900E-02	1.806E-02	2.900E-02	8	CD44 (EXT), CD44, CD44 soluble, MYLK1, MLCK, CD44 (ICD), LHX1, Telokin
181	Otorhinolaryngologic Diseases	4096	1.919E-02	3.064E-02	1.919E-02	3.064E-02	7	CD44 (EXT), CD44, CD44 soluble, MYLK1, MLCK, CD44 (ICD), Telokin
18	Microcephaly	27	1.964	3.119	1.964	3.119	1	PQBP-1

2			E-02	E-02	E-02	E-02		
183	Lip Neoplasms	809	1.987E-02	3.137E-02	1.987E-02	3.137E-02	3	MYLK1, MLCK, Telokin
184	Nervous System Neoplasms	1546	2.303E-02	3.617E-02	2.303E-02	3.617E-02	4	CD44 (EXT), CD44, CD44 soluble, CD44 (ICD)
185	Lip Diseases	867	2.383E-02	3.723E-02	2.383E-02	3.723E-02	3	MYLK1, MLCK, Telokin
186	Neoplasms, Neuroepithelial	5355	2.444E-02	3.797E-02	2.444E-02	3.797E-02	8	CD44 (EXT), CD44, CD44 soluble, MYLK1, MLCK, CD44 (ICD), LHX1, Telokin
187	Rectal Neoplasms	6533	2.544E-02	3.932E-02	2.544E-02	3.932E-02	9	CD44 (EXT), CD44, CD44 soluble, MYLK1, MLCK, CD44 (ICD), RRP42, Telokin, SNX1
188	Glioblastoma	2493	2.886E-02	4.437E-02	2.886E-02	4.437E-02	5	CD44 (EXT), CD44, CD44 soluble, MLCK, CD44 (ICD)
189	Cardiovascular Diseases	3460	3.035E-02	4.641E-02	3.035E-02	4.641E-02	6	CD44 (EXT), CD44, CD44 soluble, IGHG1, MLCK, CD44 (ICD)
190	Astrocytoma	2542	3.113E-02	4.734E-02	3.113E-02	4.734E-02	5	CD44 (EXT), CD44, CD44 soluble, MLCK, CD44 (ICD)

Table 10S: Enrichment analysis report. Enrichment by Diseases (by Biomarkers) (healthy control vs Grade III)

Enrichment by Diseases (by Biomarkers)					GRADE III_INPUT_4_genelist			
**#**	**Diseases**	**Total**	**pValue**	**Min FDR**	**p-value**	**FDR**	**In Data**	**Network Objects from Active Data**
1	Leukemia, Large Granular Lymphocytic	7	1.798E-09	1.638E-06	1.798E-09	1.638E-06	5	NOTCH1 (NICD), NOTCH1 receptor, Notch, NOTCH1 (NEXT), NOTCH1 precursor
2	Neoplasm, Residual	26	4.469E-09	2.036E-06	4.469E-09	2.036E-06	7	NOTCH1 (NICD), NOTCH1 receptor, DDC, Metallothionein-II, Notch, NOTCH1 (NEXT), NOTCH1 precursor
3	Tongue Neoplasms	65	1.420E-08	4.312E-06	1.420E-08	4.312E-06	9	NOTCH1 (NICD), Metallothionein-I, NOTCH1 receptor, Metallothionein-IG, Metallothionein-II, Notch, NOTCH1 (NEXT), NOTCH1 precursor, Metallothionein-1M
4	Tongue Diseases	69	2.434E-08	5.542E-06	2.434E-08	5.542E-06	9	NOTCH1 (NICD), Metallothionein-I, NOTCH1 receptor, Metallothionein-IG, Metallothionein-II, Notch, NOTCH1 (NEXT), NOTCH1 precursor, Metallothionein-1M
5	Aortic Aneurysm, Thoracic	32	6.107E-07	1.113E-04	6.107E-07	1.113E-04	6	Reticulon 4, NOTCH1 (NICD), NOTCH1 receptor, Notch, NOTCH1 (NEXT), NOTCH1 precursor
6	Histiocytosis, Langerhans-Cell	20	1.196E-06	1.816E-04	1.196E-06	1.816E-04	5	NOTCH1 (NICD), NOTCH1 receptor, Notch, NOTCH1 (NEXT), NOTCH1 precursor
7	Paget's Disease, Mammary	38	1.771E-06	2.124E-04	1.771E-06	2.124E-04	6	STAT5A, p38 MAPK, ERK1 (MAPK3), STAT5, KLC3, ERK1/2
8	Leukemia	1	1.	2.	1.	2.	1	Annexin VIII, NOTCH1 (NICD), TCL1A, STAT5, NOTCH1

	, Promyelocytic, Acute	46	866E-06	124E-04	866E-06	124E-04	0	receptor, ANXA8L2, Notch, NOTCH1 (NEXT), NOTCH1 precursor, 4E-BP1
9	Carcinoma, Squamous Cell	595	7.073E-06	7.159E-04	7.073E-06	7.159E-04	19	NOTCH1 (NICD), STAT5A, p38 MAPK, ERK1 (MAPK3), STAT5, NOTCH1 receptor, Fatty acid-binding protein, ERK1/2, MAGE-4 antigen, Notch, BMF, NOTCH1 (NEXT), NOTCH1 precursor, Cystatin B, WWOX, E-FABP, DKK3, MAGEA5, SPRR3
10	Paget Disease, Extramammary	31	1.200E-05	1.049E-03	1.200E-05	1.049E-03	5	STAT5A, p38 MAPK, ERK1 (MAPK3), STAT5, ERK1/2

No significant enrichment was found in healthy control vs Grade IV comparison by Diseases (by Biomarkers).

**Table 10S: Enrichment analysis report.** Enrichment by Diseases (by Biomarkers) (healthy control vs SVZp)								
Enrichment by Diseases (by Biomarkers)					SVZp Limma Input_genelist			
**#**	**Diseases**	**Total**	**pValue**	**Min FDR**	**p-value**	**FDR**	**In Data**	**Network Objects from Active Data**
1	Leukemia, Large Granular Lymphocytic	7	1.006E-11	5.824E-09	1.006E-11	5.824E-09	5	NOTCH1 (NICD), Notch, NOTCH1 (NEXT), NOTCH1 precursor, NOTCH1 receptor
2	Tongue Neoplasms	65	3.065E-09	8.873E-07	3.065E-09	8.873E-07	7	Cyclin B1, NOTCH1 (NICD), Notch, NOTCH1 (NEXT), NOTCH1 precursor, NOTCH1 receptor, Cyclin B
3	Tongue Diseases	69	4.697E-09	9.065E-07	4.697E-09	9.065E-07	7	Cyclin B1, NOTCH1 (NICD), Notch, NOTCH1 (NEXT), NOTCH1 precursor, NOTCH1 receptor, Cyclin B
4	Histiocytosis, Langerhans-Cell	20	7.165E-0	1.037E-0	7.165E-0	1.037E-0	5	NOTCH1 (NICD), Notch, NOTCH1 (NEXT), NOTCH1 precursor, NOTCH1 receptor

			9	6	9	6		
5	Neoplasm, Residual	26	2.990E-08	3.463E-06	2.990E-08	3.463E-06	5	NOTCH1 (NICD), Notch, NOTCH1 (NEXT), NOTCH1 precursor, NOTCH1 receptor
6	Dry Eye Syndromes	553	6.515E-08	5.389E-06	6.515E-08	5.389E-06	13	Tissue kallikreins, LST1, NOTCH1 (NICD), Notch, NOTCH1 (NEXT), IGHG1, NOTCH1 precursor, NFKBIL1, NOTCH1 receptor, Rab-3, Adult hemoglobin, RIG-G, ERK1/2
7	Lacrimal Apparatus Diseases	553	6.515E-08	5.389E-06	6.515E-08	5.389E-06	13	Tissue kallikreins, LST1, NOTCH1 (NICD), Notch, NOTCH1 (NEXT), IGHG1, NOTCH1 precursor, NFKBIL1, NOTCH1 receptor, Rab-3, Adult hemoglobin, RIG-G, ERK1/2
8	Aortic Aneurysm, Thoracic	32	9.004E-08	6.517E-06	9.004E-08	6.517E-06	5	NOTCH1 (NICD), Notch, NOTCH1 (NEXT), NOTCH1 precursor, NOTCH1 receptor
9	Carcinoma, Embryonal	34	1.237E-07	7.960E-06	1.237E-07	7.960E-06	5	NOTCH1 (NICD), Notch, NOTCH1 (NEXT), NOTCH1 precursor, NOTCH1 receptor

10	Pick Disease of the Brain	77	3.094E-07	1.740E-05	3.094E-07	1.740E-05	6	NOTCH1 (NICD), Notch, NOTCH1 (NEXT), NOTCH1 precursor, NOTCH1 receptor, ERK1/2
11	Frontotemporal Lobar Degeneration	79	3.607E-07	1.740E-05	3.607E-07	1.740E-05	6	NOTCH1 (NICD), Notch, NOTCH1 (NEXT), NOTCH1 precursor, NOTCH1 receptor, ERK1/2
12	Frontotemporal Dementia	79	3.607E-07	1.740E-05	3.607E-07	1.740E-05	6	NOTCH1 (NICD), Notch, NOTCH1 (NEXT), NOTCH1 precursor, NOTCH1 receptor, ERK1/2
13	Ameloblastoma	48	7.326E-07	3.263E-05	7.326E-07	3.263E-05	5	NOTCH1 (NICD), Notch, NOTCH1 (NEXT), NOTCH1 precursor, NOTCH1 receptor
14	Carcinoid Tumor	49	8.136E-07	3.365E-05	8.136E-07	3.365E-05	5	NOTCH1 (NICD), Notch, NOTCH1 (NEXT), NOTCH1 precursor, NOTCH1 receptor
15	Neoplasms, Squamou	65	2.	1.	2.	1.	12	Tissue kallikreins, Cyclin B1, NOTCH1 (NICD), Rab-11A, Notch, NOTCH1 (NEXT), ERK1 (MAPK3), NOTCH1 precursor, NOTCH1

	s Cell	3	924E-06	129E-04	924E-06	129E-04		receptor, Cyclin B, ERK1/2, 14-3-3
16	Odontogenic Tumors	65	3.373E-06	1.220E-04	3.373E-06	1.220E-04	5	NOTCH1 (NICD), Notch, NOTCH1 (NEXT), NOTCH1 precursor, NOTCH1 receptor
17	Neoplasm Metastasis	676	4.167E-06	1.419E-04	4.167E-06	1.419E-04	12	Tissue kallikreins, Cyclin B1, NOTCH1 (NICD), Notch, NOTCH1 (NEXT), ERK1 (MAPK3), NOTCH1 precursor, NOTCH1 receptor, Cyclin B, ERK1/2, UGDH, 14-3-3
18	Lymphoma, B-Cell	372	6.462E-06	1.962E-04	6.462E-06	1.962E-04	9	LST1, Cyclin B1, NOTCH1 (NICD), Notch, NOTCH1 (NEXT), NOTCH1 precursor, CD43, NOTCH1 receptor, Cyclin B
19	Sclerosis	375	6.894E-06	1.962E-04	6.894E-06	1.962E-04	9	NOTCH1 (NICD), IRT-1, Notch, NOTCH1 (NEXT), ERK1 (MAPK3), NOTCH1 precursor, NOTCH1 receptor, ERK1/2, 14-3-3
20	Thyroid Carcinoma, Papillary	377	7.19	1.96	7.19	1.96	9	NOTCH1 (NICD), Notch, NOTCH1 (NEXT), ERK1 (MAPK3), NOTCH1 precursor, CKS2, NOTCH1 receptor, ERK1/2, 14-3-3

			5E-06	2E-04	5E-06	2E-04		
21	Connective Tissue Diseases	2088	7.449E-06	1.962E-04	7.449E-06	1.962E-04	21	Tissue kallikreins, IGHG4, Kallikrein 1, LST1, NOTCH1 (NICD), IRT-1, TIRAP (Mal), Olfactory receptor, Notch, NOTCH1 (NEXT), IGHG1, ERK1 (MAPK3), NOTCH1 precursor, CD43, NFKBIL1, NOTCH1 receptor, Rab-3, Adult hemoglobin, RIG-G, ERK1/2, 14-3-3
22	Carcinoma, Squamous Cell	595	7.456E-06	1.962E-04	7.456E-06	1.962E-04	11	Cyclin B1, NOTCH1 (NICD), Rab-11A, Notch, NOTCH1 (NEXT), ERK1 (MAPK3), NOTCH1 precursor, NOTCH1 receptor, Cyclin B, ERK1/2, 14-3-3
23	Carcinoma, Adenoid Cystic	37	8.695E-06	2.130E-04	8.695E-06	2.130E-04	4	ERK1 (MAPK3), CD43, ERK1/2, 14-3-3
24	Neoplastic Processes	728	8.828E-06	2.130E-04	8.828E-06	2.130E-04	12	Tissue kallikreins, Cyclin B1, NOTCH1 (NICD), Notch, NOTCH1 (NEXT), ERK1 (MAPK3), NOTCH1 precursor, NOTCH1 receptor, Cyclin B, ERK1/2, UGDH, 14-3-3
25	Histiocytosis	84	1.196E	2.771E	1.196E	2.771E	5	NOTCH1 (NICD), Notch, NOTCH1 (NEXT), NOTCH1 precursor, NOTCH1 receptor

			-05	-04	-05	-04		
26	Hyperparathyroidism, Primary	14	1.499E-05	3.339E-04	1.499E-05	3.339E-04	3	HBG, HBGA, Adult hemoglobin
27	Arthritis, Rheumatoid	1367	1.876E-05	4.024E-04	1.876E-05	4.024E-04	16	Tissue kallikreins, IGHG4, Kallikrein 1, LST1, NOTCH1 (NICD), IRT-1, Notch, NOTCH1 (NEXT), IGHG1, NOTCH1 precursor, NFKBIL1, NOTCH1 receptor, Rab-3, Adult hemoglobin, RIG-G, ERK1/2
28	Colonic Neoplasms	1232	2.266E-05	4.686E-04	2.266E-05	4.686E-04	15	Tissue kallikreins, IGHG4, Cyclin B1, AL1A1, NOTCH1 (NICD), SNX1, Notch, NOTCH1 (NEXT), IGHG1, ERK1 (MAPK3), NOTCH1 precursor, SULT1E1, NOTCH1 receptor, Cyclin B, ERK1/2
29	Lymphoma	1250	2.683E-05	5.357E-04	2.683E-05	5.357E-04	15	Tissue kallikreins, LST1, Cyclin B1, NOTCH1 (NICD), Olfactory receptor, Notch, NOTCH1 (NEXT), ERK1 (MAPK3), NOTCH1 precursor, CD43, NOTCH1 receptor, NMRAL1, Cyclin B, Kallikrein 3 (PSA), ERK1/2
30	Precursor T-Cell Lymphoblastic Leukemia-Lymphoma	249	2.838E-0	5.478E-0	2.838E-0	5.478E-0	7	Tissue kallikreins, NOTCH1 (NICD), Olfactory receptor, Notch, NOTCH1 (NEXT), NOTCH1 precursor, NOTCH1 receptor

			5	4	5	4		
31	Carcinoma, Ductal, Breast	1108	2.954E-05	5.517E-04	2.954E-05	5.517E-04	14	Tissue kallikreins, Cyclin B1, NOTCH1 (NICD), Rab-11A, Notch, NOTCH1 (NEXT), ERK1 (MAPK3), NOTCH1 precursor, SULT1E1, NOTCH1 receptor, RNF11, Cyclin B, ERK1/2, 14-3-3
32	Esophageal Diseases	569	3.092E-05	5.595E-04	3.092E-05	5.595E-04	10	Cyclin B1, NOTCH1 (NICD), Notch, NOTCH1 (NEXT), ERK1 (MAPK3), NOTCH1 precursor, CKS2, NOTCH1 receptor, Cyclin B, ERK1/2
33	Wounds and Injuries	982	3.663E-05	6.427E-04	3.663E-05	6.427E-04	13	Tissue kallikreins, Kallikrein 1, NOTCH1 (NICD), IRT-1, Notch, NOTCH1 (NEXT), ERK1 (MAPK3), NOTCH1 precursor, NOTCH1 receptor, Kallikrein 3 (PSA), Adult hemoglobin, ERK1/2, 14-3-3
34	Immune System Diseases	6037	4.364E-05	7.431E-04	4.364E-05	7.431E-04	38	Tissue kallikreins, IGHG4, ZNF261, Kallikrein 1, LST1, Cyclin B1, AL1A1, HBG, Leiomodin, NOTCH1 (NICD), IRT-1, HBGA, TIP47, TIRAP (Mal), PROSC, Olfactory receptor, Notch, NOTCH1 (NEXT), IGHG1, ERK1 (MAPK3), NOTCH1 precursor, CD43, 14-3-3 zeta/delta, NFKBIL1, OATP-C, CKS2, Annexin XI, NOTCH1 receptor, Cyclin B, Rab-3, Kallikrein 3 (PSA), Adult hemoglobin, RIG-G, VILIP3, ERK1/2, 14-3-3, CapZIP, RBBP7 (RbAp46)
35	Stroke	371	4.935E-05	8.163E-04	4.935E-05	8.163E-04	8	NOTCH1 (NICD), Notch, NOTCH1 (NEXT), ERK1 (MAPK3), NOTCH1 precursor, NOTCH1 receptor, Adult hemoglobin, ERK1/2

36	Cerebrovascular Disorders	605	5.184E-05	8.337E-04	5.184E-05	8.337E-04	10	Tissue kallikreins, Kallikrein 1, NOTCH1 (NICD), Notch, NOTCH1 (NEXT), ERK1 (MAPK3), NOTCH1 precursor, NOTCH1 receptor, Adult hemoglobin, ERK1/2
37	Lymphoma, Non-Hodgkin	1020	5.411E-05	8.468E-04	5.411E-05	8.468E-04	13	Tissue kallikreins, LST1, Cyclin B1, NOTCH1 (NICD), Olfactory receptor, Notch, NOTCH1 (NEXT), NOTCH1 precursor, CD43, NOTCH1 receptor, NMRAL1, Cyclin B, Kallikrein 3 (PSA)
38	Rheumatic Diseases	1509	6.214E-05	9.468E-04	6.214E-05	9.468E-04	16	Tissue kallikreins, IGHG4, Kallikrein 1, LST1, NOTCH1 (NICD), IRT-1, Notch, NOTCH1 (NEXT), IGHG1, NOTCH1 precursor, NFKBIL1, NOTCH1 receptor, Rab-3, Adult hemoglobin, RIG-G, ERK1/2
39	Dementia	1515	6.514E-05	9.671E-04	6.514E-05	9.671E-04	16	Tissue kallikreins, Kallikrein 1, HBG, NOTCH1 (NICD), IRT-1, HBGA, Notch, NOTCH1 (NEXT), ERK1 (MAPK3), NOTCH1 precursor, 14-3-3 zeta/delta, NOTCH1 receptor, Adult hemoglobin, VILIP3, ERK1/2, 14-3-3
40	Hirsutism	4	7.354E-05	1.065E-03	7.354E-05	1.065E-03	2	Tissue kallikreins, Kallikrein 3 (PSA)
41	Aortic Aneurysm	12	9.	1.	9.	1.	5	NOTCH1 (NICD), Notch, NOTCH1 (NEXT), NOTCH1 precursor, NOTCH1 receptor

		9	427E-05	331E-03	427E-05	331E-03		
42	Lymphoma, Large B-Cell, Diffuse	132	1.051E-04	1.449E-03	1.051E-04	1.449E-03	5	NOTCH1 (NICD), Notch, NOTCH1 (NEXT), NOTCH1 precursor, NOTCH1 receptor
43	Carcinoma, Ductal	1414	1.091E-04	1.470E-03	1.091E-04	1.470E-03	15	Tissue kallikreins, Cyclin B1, LHX1, NOTCH1 (NICD), Rab-11A, Notch, NOTCH1 (NEXT), ERK1 (MAPK3), NOTCH1 precursor, SULT1E1, NOTCH1 receptor, RNF11, Cyclin B, ERK1/2, 14-3-3
44	Monoclonal Gammopathy of Undetermined Significance	136	1.209E-04	1.559E-03	1.209E-04	1.559E-03	5	NOTCH1 (NICD), Notch, NOTCH1 (NEXT), NOTCH1 precursor, NOTCH1 receptor
45	Arthritis	1597	1.211E-04	1.559E-03	1.211E-04	1.559E-03	16	Tissue kallikreins, IGHG4, Kallikrein 1, LST1, NOTCH1 (NICD), IRT-1, Notch, NOTCH1 (NEXT), IGHG1, NOTCH1 precursor, NFKBIL1, NOTCH1 receptor, Rab-3, Adult hemoglobin, RIG-G, ERK1/2
46	Hypergammaglobulinemia	140	1.38	1.74	1.38	1.74	5	NOTCH1 (NICD), Notch, NOTCH1 (NEXT), NOTCH1 precursor, NOTCH1 receptor

			6E-04	4E-03	6E-04	4E-03		
47	Stomach Neoplasms	3793	1.479E-04	1.776E-03	1.479E-04	1.776E-03	27	Ajuba, Tissue kallikreins, IGHG4, Kallikrein 1, Cyclin B1, FLJ11017, AL1A1, LHX1, Leiomodin, NOTCH1 (NICD), IRT-1, Rab-11A, Olfactory receptor, Notch, NOTCH1 (NEXT), IGHG1, ERK1 (MAPK3), NOTCH1 precursor, SULT1E1, CD43, 14-3-3 zeta/delta, CKS2, NOTCH1 receptor, Cyclin B, Adult hemoglobin, ERK1/2, 14-3-3
48	Autoimmune Diseases	2751	1.487E-04	1.776E-03	1.487E-04	1.776E-03	22	Tissue kallikreins, IGHG4, Kallikrein 1, LST1, Leiomodin, NOTCH1 (NICD), IRT-1, TIRAP (Mal), Olfactory receptor, Notch, NOTCH1 (NEXT), IGHG1, NOTCH1 precursor, CD43, NFKBIL1, Annexin XI, NOTCH1 receptor, Rab-3, Adult hemoglobin, RIG-G, ERK1/2, 14-3-3
49	Joint Diseases	1627	1.503E-04	1.776E-03	1.503E-04	1.776E-03	16	Tissue kallikreins, IGHG4, Kallikrein 1, LST1, NOTCH1 (NICD), IRT-1, Notch, NOTCH1 (NEXT), IGHG1, NOTCH1 precursor, NFKBIL1, NOTCH1 receptor, Rab-3, Adult hemoglobin, RIG-G, ERK1/2
50	Neoplasms, Ductal, Lobular, and Medullary	1467	1.640E-04	1.899E-03	1.640E-04	1.899E-03	15	Tissue kallikreins, Cyclin B1, LHX1, NOTCH1 (NICD), Rab-11A, Notch, NOTCH1 (NEXT), ERK1 (MAPK3), NOTCH1 precursor, SULT1E1, NOTCH1 receptor, RNF11, Cyclin B, ERK1/2, 14-3-3
51	Leukemia, Promyelocytic, Acute	146	1.686E	1.915E	1.686E	1.915E	5	NOTCH1 (NICD), Notch, NOTCH1 (NEXT), NOTCH1 precursor, NOTCH1 receptor

			-04	-03	-04	-03		
52	Small Cell Lung Carcinoma	446	1.765E-04	1.965E-03	1.765E-04	1.965E-03	8	Cyclin B1, NOTCH1 (NICD), Notch, NOTCH1 (NEXT), NOTCH1 precursor, NOTCH1 receptor, Cyclin B, 14-3-3
53	Stomach Diseases	3840	1.826E-04	1.995E-03	1.826E-04	1.995E-03	27	Ajuba, Tissue kallikreins, IGHG4, Kallikrein 1, Cyclin B1, FLJ11017, AL1A1, LHX1, Leiomodin, NOTCH1 (NICD), IRT-1, Rab-11A, Olfactory receptor, Notch, NOTCH1 (NEXT), IGHG1, ERK1 (MAPK3), NOTCH1 precursor, SULT1E1, CD43, 14-3-3 zeta/delta, CKS2, NOTCH1 receptor, Cyclin B, Adult hemoglobin, ERK1/2, 14-3-3
54	Adenoma	583	2.104E-04	2.256E-03	2.104E-04	2.256E-03	9	NOTCH1 (NICD), Notch, NOTCH1 (NEXT), ERK1 (MAPK3), NOTCH1 precursor, CD43, NOTCH1 receptor, Cyclin B, ERK1/2
55	Leukemia-Lymphoma, Adult T-Cell	154	2.162E-04	2.276E-03	2.162E-04	2.276E-03	5	NOTCH1 (NICD), Notch, NOTCH1 (NEXT), NOTCH1 precursor, NOTCH1 receptor
56	Eye Diseases	1692	2.355E-0	2.435E-0	2.355E-0	2.435E-0	16	Tissue kallikreins, LST1, Leiomodin, NOTCH1 (NICD), Notch, NOTCH1 (NEXT), IGHG1, ERK1 (MAPK3), NOTCH1 precursor, NFKBIL1, NOTCH1 receptor, Rab-3, Adult hemoglobin, RIG-G, ERK1/2, RBBP7 (RbAp46)

			4	3	4	3		
57	Salivary Gland Diseases	594	2.416E-04	2.455E-03	2.416E-04	2.455E-03	9	Tissue kallikreins, LST1, IGHG1, ERK1 (MAPK3), NFKBIL1, Rab-3, Adult hemoglobin, RIG-G, ERK1/2
58	Precursor Cell Lymphoblastic Leukemia - Lymphoma	605	2.766E-04	2.714E-03	2.766E-04	2.714E-03	9	Tissue kallikreins, NOTCH1 (NICD), Olfactory receptor, Notch, NOTCH1 (NEXT), NOTCH1 precursor, NOTCH1 receptor, ERK1/2, CapZIP
59	Hyperparathyroidism	36	2.781E-04	2.714E-03	2.781E-04	2.714E-03	3	HBG, HBGA, Adult hemoglobin
60	Aortic Diseases	163	2.812E-04	2.714E-03	2.812E-04	2.714E-03	5	NOTCH1 (NICD), Notch, NOTCH1 (NEXT), NOTCH1 precursor, NOTCH1 receptor
61	Virilism	8	3.401E-04	3.202E-03	3.401E-04	3.202E-03	2	Tissue kallikreins, Kallikrein 3 (PSA)

62	Melanoma, Cutaneous Malignant	763	3.429E-04	3.202E-03	3.429E-04	3.202E-03	10	Tissue kallikreins, Cyclin B1, NOTCH1 (NICD), Notch, NOTCH1 (NEXT), ERK1 (MAPK3), NOTCH1 precursor, NOTCH1 receptor, Cyclin B, ERK1/2
63	Esophageal Neoplasms	495	3.561E-04	3.252E-03	3.561E-04	3.252E-03	8	Cyclin B1, NOTCH1 (NICD), Notch, NOTCH1 (NEXT), NOTCH1 precursor, CKS2, NOTCH1 receptor, Cyclin B
64	Sjogren's Syndrome	497	3.658E-04	3.252E-03	3.658E-04	3.252E-03	8	Tissue kallikreins, LST1, IGHG1, NFKBIL1, Rab-3, Adult hemoglobin, RIG-G, ERK1/2
65	Xerostomia	497	3.658E-04	3.252E-03	3.658E-04	3.252E-03	8	Tissue kallikreins, LST1, IGHG1, NFKBIL1, Rab-3, Adult hemoglobin, RIG-G, ERK1/2
66	Anoxia	498	3.707E-04	3.252E-03	3.707E-04	3.252E-03	8	NOTCH1 (NICD), Notch, NOTCH1 (NEXT), ERK1 (MAPK3), NOTCH1 precursor, NOTCH1 receptor, ERK1/2, UGDH
67	Immunoproliferative Disorders	37	3.	3.	3.	3.	26	Tissue kallikreins, ZNF261, LST1, Cyclin B1, HBG, NOTCH1 (NICD), HBGA, PROSC, Olfactory receptor, Notch, NOTCH1 (NEXT),

		93	853E-04	330E-03	853E-04	330E-03		IGHG1, ERK1 (MAPK3), NOTCH1 precursor, CD43, 14-3-3 zeta/delta, OATP-C, CKS2, NOTCH1 receptor, Cyclin B, Kallikrein 3 (PSA), Adult hemoglobin, ERK1/2, 14-3-3, CapZIP, RBBP7 (RbAp46)
68	Carcinoma, Renal Cell	1594	4.018E-04	3.421E-03	4.018E-04	3.421E-03	15	Tissue kallikreins, Kallikrein 1, Cyclin B1, LHX1, HBG, NOTCH1 (NICD), HBGA, Notch, NOTCH1 (NEXT), NOTCH1 precursor, CKS2, NOTCH1 receptor, Cyclin B, Kallikrein 3 (PSA), Adult hemoglobin
69	beta-Thalassemia	41	4.100E-04	3.440E-03	4.100E-04	3.440E-03	3	HBG, HBGA, Adult hemoglobin
70	Thyroid Neoplasms	787	4.375E-04	3.619E-03	4.375E-04	3.619E-03	10	NOTCH1 (NICD), Notch, NOTCH1 (NEXT), ERK1 (MAPK3), NOTCH1 precursor, CKS2, NOTCH1 receptor, Cyclin B, ERK1/2, 14-3-3
71	Osteoarthritis	278	4.526E-04	3.691E-03	4.526E-04	3.691E-03	6	NOTCH1 (NICD), Notch, NOTCH1 (NEXT), NOTCH1 precursor, NOTCH1 receptor, ERK1/2
72	Lymphoproliferative Disorders	3847	4.82	3.88	4.82	3.88	26	Tissue kallikreins, ZNF261, LST1, Cyclin B1, HBG, NOTCH1 (NICD), HBGA, PROSC, Olfactory receptor, Notch, NOTCH1 (NEXT), IGHG1, ERK1 (MAPK3), NOTCH1 precursor, CD43, 14-3-3 zeta/delta, OATP-C, CKS2, NOTCH1 receptor, Cyclin B, Kallikrein

			9E-04	3E-03	9E-04	3E-03		3 (PSA), Adult hemoglobin, ERK1/2, 14-3-3, CapZIP, RBBP7 (RbAp46)
73	Neuroblastoma	520	4.936E-04	3.915E-03	4.936E-04	3.915E-03	8	NOTCH1 (NICD), Notch, NOTCH1 (NEXT), ERK1 (MAPK3), DR1, NOTCH1 precursor, NOTCH1 receptor, ERK1/2
74	Aneurysm	185	5.024E-04	3.931E-03	5.024E-04	3.931E-03	5	NOTCH1 (NICD), Notch, NOTCH1 (NEXT), NOTCH1 precursor, NOTCH1 receptor
75	Thyroid Diseases	964	5.510E-04	4.254E-03	5.510E-04	4.254E-03	11	Leiomodin, NOTCH1 (NICD), Notch, NOTCH1 (NEXT), ERK1 (MAPK3), NOTCH1 precursor, CKS2, NOTCH1 receptor, Cyclin B, ERK1/2, 14-3-3
76	Neuroectodermal Tumors, Primitive, Peripheral	531	5.664E-04	4.315E-03	5.664E-04	4.315E-03	8	NOTCH1 (NICD), Notch, NOTCH1 (NEXT), ERK1 (MAPK3), DR1, NOTCH1 precursor, NOTCH1 receptor, ERK1/2
77	Leukemia, T-Cell	200	7.151E	5.377E	7.151E	5.377E	5	NOTCH1 (NICD), Notch, NOTCH1 (NEXT), NOTCH1 precursor, NOTCH1 receptor

			-04	-03	-04	-03		
78	Lupus Erythematosus, Systemic	1161	7.306E-04	5.423E-03	7.306E-04	5.423E-03	12	NOTCH1 (NICD), TIRAP (Mal), Olfactory receptor, Notch, NOTCH1 (NEXT), NOTCH1 precursor, CD43, NFKBIL1, NOTCH1 receptor, RIG-G, ERK1/2, 14-3-3
79	Neoplasms, Neuroepithelial	5355	7.552E-04	5.535E-03	7.552E-04	5.535E-03	32	CDH26, Tissue kallikreins, ZNF261, LRFN1, Cyclin B1, TIPIN, LHX1, NOTCH1 (NICD), Gpbp1, PROSC, Olfactory receptor, Notch, C14orf119, NOTCH1 (NEXT), ERK1 (MAPK3), DR1, NOTCH1 precursor, SULT1E1, AO7, OATP-C, CKS2, OR10G3, PLEKHG2, NOTCH1 receptor, NMRAL1, DOHH, Cyclin B, Rab-3, RIG-G, ERK1/2, CapZIP, RBBP7 (RbAp46)
80	Hemangioma	51	7.809E-04	5.608E-03	7.809E-04	5.608E-03	3	IRT-1, ERK1 (MAPK3), ERK1/2
81	Pathologic Processes	2678	7.930E-04	5.608E-03	7.930E-04	5.608E-03	20	Tissue kallikreins, Kallikrein 1, LST1, Cyclin B1, NOTCH1 (NICD), IRT-1, Notch, NOTCH1 (NEXT), ERK1 (MAPK3), NOTCH1 precursor, 14-3-3 zeta/delta, NFKBIL1, OATP-C, NOTCH1 receptor, Cyclin B, Kallikrein 3 (PSA), Adult hemoglobin, ERK1/2, UGDH, 14-3-3
82	Bowen's Disease	12	7.942E-0	5.608E-0	7.942E-0	5.608E-0	2	ERK1 (MAPK3), ERK1/2

			4	3	4	3		
83	Lymphoma, T-Cell	431	8.221E-04	5.735E-03	8.221E-04	5.735E-03	7	NOTCH1 (NICD), Olfactory receptor, Notch, NOTCH1 (NEXT), NOTCH1 precursor, NOTCH1 receptor, NMRAL1
84	Glioblastoma	2493	8.685E-04	5.936E-03	8.685E-04	5.936E-03	19	CDH26, Tissue kallikreins, NOTCH1 (NICD), Gpbp1, PROSC, Olfactory receptor, Notch, NOTCH1 (NEXT), ERK1 (MAPK3), NOTCH1 precursor, AO7, OATP-C, CKS2, OR10G3, NOTCH1 receptor, Rab-3, RIG-G, ERK1/2, CapZIP
85	Carcinoma, Intraductal, Noninfiltrating	209	8.714E-04	5.936E-03	8.714E-04	5.936E-03	5	Cyclin B1, ERK1 (MAPK3), Cyclin B, ERK1/2, 14-3-3
86	Vascular Diseases	3123	8.933E-04	5.974E-03	8.933E-04	5.974E-03	22	Tissue kallikreins, Kallikrein 1, HBG, NOTCH1 (NICD), IRT-1, HBGA, Olfactory receptor, Notch, NOTCH1 (NEXT), IGHG1, ERK1 (MAPK3), NOTCH1 precursor, 14-3-3 zeta/delta, NFKBIL1, OATP-C, CKS2, NOTCH1 receptor, Adult hemoglobin, VILIP3, ERK1/2, 14-3-3, RBBP7 (RbAp46)
87	Phenomena and Processes	1189	8.997E-04	5.974E-03	8.997E-04	5.974E-03	12	Tissue kallikreins, NOTCH1 (NICD), Notch, NOTCH1 (NEXT), ERK1 (MAPK3), SRB7, NOTCH1 precursor, MTL5, NOTCH1 receptor, Kallikrein 3 (PSA), RIG-G, ERK1/2

88	Signs and Symptoms, Respiratory	571	9.080E-04	5.974E-03	9.080E-04	5.974E-03	8	NOTCH1 (NICD), Notch, NOTCH1 (NEXT), ERK1 (MAPK3), NOTCH1 precursor, NOTCH1 receptor, ERK1/2, UGDH
89	Musculoskeletal Diseases	2511	9.478E-04	6.166E-03	9.478E-04	6.166E-03	19	Tissue kallikreins, IGHG4, Kallikrein 1, LST1, NOTCH1 (NICD), IRT-1, Notch, NOTCH1 (NEXT), IGHG1, ERK1 (MAPK3), NOTCH1 precursor, NFKBIL1, OATP-C, NOTCH1 receptor, Rab-3, Kallikrein 3 (PSA), Adult hemoglobin, RIG-G, ERK1/2
90	Cholesteatoma	14	1.090E-03	6.918E-03	1.090E-03	6.918E-03	2	ERK1 (MAPK3), ERK1/2
91	Ganglioglioma	14	1.090E-03	6.918E-03	1.090E-03	6.918E-03	2	Cyclin B1, Cyclin B
92	Astrocytoma	2542	1.099E-03	6.918E-03	1.099E-03	6.918E-03	19	CDH26, Tissue kallikreins, NOTCH1 (NICD), Gpbp1, PROSC, Olfactory receptor, Notch, NOTCH1 (NEXT), ERK1 (MAPK3), NOTCH1 precursor, AO7, OATP-C, CKS2, OR10G3, NOTCH1 receptor, Rab-3, RIG-G, ERK1/2, CapZIP
93	Dementia, Vascular	60	1.	7.	1.	7.	3	Tissue kallikreins, Kallikrein 1, Notch

			254E-03	730E-03	254E-03	730E-03		
94	Pneumococcal Infections	15	1.255E-03	7.730E-03	1.255E-03	7.730E-03	2	ERK1 (MAPK3), ERK1/2
95	Endocrine System Diseases	5758	1.278E-03	7.792E-03	1.278E-03	7.792E-03	33	CDH26, Tissue kallikreins, Kallikrein 1, Cyclin B1, AL1A1, LHX1, HBG, Leiomodin, NOTCH1 (NICD), IRT-1, HBGA, TIP47, Olfactory receptor, Notch, NOTCH1 (NEXT), IGHG1, ERK1 (MAPK3), NOTCH1 precursor, Lemd1, Hn1, NFKBIL1, OATP-C, CKS2, NOTCH1 receptor, RNF11, Cyclin B, Kallikrein 3 (PSA), Adult hemoglobin, ZHX3, ERK1/2, 14-3-3, CCDC28A, RBBP7 (RbAp46)
96	Ovarian Neoplasms	3027	1.478E-03	8.911E-03	1.478E-03	8.911E-03	21	Tissue kallikreins, Kallikrein 1, Cyclin B1, AL1A1, NOTCH1 (NICD), IRT-1, TIP47, Olfactory receptor, Notch, NOTCH1 (NEXT), ERK1 (MAPK3), NOTCH1 precursor, Hn1, OATP-C, NOTCH1 receptor, Cyclin B, Kallikrein 3 (PSA), ZHX3, ERK1/2, 14-3-3, CCDC28A
97	Glioma	5088	1.549E-03	9.244E-03	1.549E-03	9.244E-03	30	CDH26, Tissue kallikreins, ZNF261, LRFN1, Cyclin B1, TIPIN, LHX1, NOTCH1 (NICD), Gpbp1, PROSC, Olfactory receptor, Notch, C14orf119, NOTCH1 (NEXT), ERK1 (MAPK3), NOTCH1 precursor, SULT1E1, AO7, OATP-C, CKS2, OR10G3, PLEKHG2, NOTCH1 receptor, NMRAL1, DOHH, Cyclin B, Rab-3, RIG-G, ERK1/2, CapZIP
98	Schizophrenia, Paranoid	17	1.61	9.55	1.61	9.55	2	14-3-3 zeta/delta, 14-3-3

			8E-03	9E-03	8E-03	9E-03		
99	Thalassemia	66	1.652E-03	9.662E-03	1.652E-03	9.662E-03	3	HBG, HBGA, Adult hemoglobin
100	Central Nervous System Infections	246	1.793E-03	1.038E-02	1.793E-03	1.038E-02	5	IRT-1, ERK1 (MAPK3), Adult hemoglobin, ERK1/2, 14-3-3
101	Carcinoma, Mucoepidermoid	18	1.816E-03	1.041E-02	1.816E-03	1.041E-02	2	ERK1 (MAPK3), ERK1/2
102	Leiomyoma	148	1.880E-03	1.067E-02	1.880E-03	1.067E-02	4	AL1A1, ERK1 (MAPK3), ERK1/2, 14-3-3
103	Neoplasms, Multiple Primary	71	2.038E	1.145E	2.038E	1.145E	3	RNF11, ERK1/2, 14-3-3

			-03	-02	-03	-02		
104	Neuroectodermal Tumors, Primitive	3333	2.115E-03	1.178E-02	2.115E-03	1.178E-02	22	Tissue kallikreins, ZNF261, LRFN1, Cyclin B1, TIPIN, LHX1, NOTCH1 (NICD), Olfactory receptor, Notch, C14orf119, NOTCH1 (NEXT), ERK1 (MAPK3), DR1, NOTCH1 precursor, SULT1E1, PLEKHG2, NOTCH1 receptor, NMRAL1, DOHH, Cyclin B, RIG-G, ERK1/2
105	Alzheimer Disease	1141	2.152E-03	1.182E-02	2.152E-03	1.182E-02	11	Tissue kallikreins, NOTCH1 (NICD), Notch, NOTCH1 (NEXT), ERK1 (MAPK3), NOTCH1 precursor, 14-3-3 zeta/delta, NOTCH1 receptor, VILIP3, ERK1/2, 14-3-3
106	Multiple Myeloma	1882	2.184E-03	1.182E-02	2.184E-03	1.182E-02	15	Tissue kallikreins, HBG, NOTCH1 (NICD), HBGA, Olfactory receptor, Notch, NOTCH1 (NEXT), IGHG1, NOTCH1 precursor, 14-3-3 zeta/delta, CKS2, NOTCH1 receptor, Adult hemoglobin, 14-3-3, RBBP7 (RbAp46)
107	Neurodegenerative Diseases	2079	2.184E-03	1.182E-02	2.184E-03	1.182E-02	16	Tissue kallikreins, HBG, NOTCH1 (NICD), HBGA, Notch, NOTCH1 (NEXT), ERK1 (MAPK3), NOTCH1 precursor, 14-3-3 zeta/delta, NOTCH1 receptor, RNF11, Adult hemoglobin, Protrudin, VILIP3, ERK1/2, 14-3-3
108	Paranasal Sinus Diseases	155	2.224E-0	1.192E-0	2.224E-0	1.192E-0	4	Tissue kallikreins, Olfactory receptor, Notch, Spinophilin

			3	2	3	2			
109	Neoplasms, Plasma Cell	1891	2.288E-03	1.196E-02	2.288E-03	1.196E-02	15	Tissue kallikreins, HBG, NOTCH1 (NICD), HBGA, Olfactory receptor, Notch, NOTCH1 (NEXT), IGHG1, NOTCH1 precursor, 14-3-3 zeta/delta, CKS2, NOTCH1 receptor, Adult hemoglobin, 14-3-3, RBBP7 (RbAp46)
110	Paraproteinemias	1891	2.288E-03	1.196E-02	2.288E-03	1.196E-02	15	Tissue kallikreins, HBG, NOTCH1 (NICD), HBGA, Olfactory receptor, Notch, NOTCH1 (NEXT), IGHG1, NOTCH1 precursor, 14-3-3 zeta/delta, CKS2, NOTCH1 receptor, Adult hemoglobin, 14-3-3, RBBP7 (RbAp46)
111	Parathyroid Diseases	74	2.293E-03	1.196E-02	2.293E-03	1.196E-02	3	HBG, HBGA, Adult hemoglobin
112	Ovarian Diseases	3139	2.333E-03	1.196E-02	2.333E-03	1.196E-02	21	Tissue kallikreins, Kallikrein 1, Cyclin B1, AL1A1, NOTCH1 (NICD), IRT-1, TIP47, Olfactory receptor, Notch, NOTCH1 (NEXT), ERK1 (MAPK3), NOTCH1 precursor, Hn1, OATP-C, NOTCH1 receptor, Cyclin B, Kallikrein 3 (PSA), ZHX3, ERK1/2, 14-3-3, CCDC28A
113	Tauopathies	1153	2.334E-03	1.196E-02	2.334E-03	1.196E-02	11	Tissue kallikreins, NOTCH1 (NICD), Notch, NOTCH1 (NEXT), ERK1 (MAPK3), NOTCH1 precursor, 14-3-3 zeta/delta, NOTCH1 receptor, VILIP3, ERK1/2, 14-3-3

114	Adnexal Diseases	3142	2.361E-03	1.199E-02	2.361E-03	1.199E-02	21	Tissue kallikreins, Kallikrein 1, Cyclin B1, AL1A1, NOTCH1 (NICD), IRT-1, TIP47, Olfactory receptor, Notch, NOTCH1 (NEXT), ERK1 (MAPK3), NOTCH1 precursor, Hn1, OATP-C, NOTCH1 receptor, Cyclin B, Kallikrein 3 (PSA), ZHX3, ERK1/2, 14-3-3, CCDC28A
115	Colonic Diseases	4272	2.383E-03	1.200E-02	2.383E-03	1.200E-02	26	Tissue kallikreins, IGHG4, Cyclin B1, AL1A1, NOTCH1 (NICD), SNX1, Olfactory receptor, Notch, Thioredoxin-like 2, NOTCH1 (NEXT), IGHG1, ERK1 (MAPK3), NOTCH1 precursor, SULT1E1, CD43, 14-3-3 zeta/delta, NFKBIL1, OATP-C, CKS2, Annexin XI, NOTCH1 receptor, Cyclin B, Kallikrein 3 (PSA), Adult hemoglobin, ERK1/2, 14-3-3
116	Encephalitis, Japanese	21	2.476E-03	1.236E-02	2.476E-03	1.236E-02	2	ERK1 (MAPK3), ERK1/2
117	Blood Protein Disorders	1922	2.678E-03	1.325E-02	2.678E-03	1.325E-02	15	Tissue kallikreins, HBG, NOTCH1 (NICD), HBGA, Olfactory receptor, Notch, NOTCH1 (NEXT), IGHG1, NOTCH1 precursor, 14-3-3 zeta/delta, CKS2, NOTCH1 receptor, Adult hemoglobin, 14-3-3, RBBP7 (RbAp46)
118	Hemostatic Disorders	1940	2.929E-03	1.437E-02	2.929E-03	1.437E-02	15	Tissue kallikreins, HBG, NOTCH1 (NICD), HBGA, Olfactory receptor, Notch, NOTCH1 (NEXT), IGHG1, NOTCH1 precursor, 14-3-3 zeta/delta, CKS2, NOTCH1 receptor, Adult hemoglobin, 14-3-3, RBBP7 (RbAp46)
11	Endocrine Gland Neoplasm	48	2.	1.	2.	1.	28	CDH26, Tissue kallikreins, Kallikrein 1, Cyclin B1, AL1A1, LHX1, NOTCH1 (NICD), IRT-1, TIP47, Olfactory receptor, Notch,

9	s	11	971E-03	446E-02	971E-03	446E-02		NOTCH1 (NEXT), IGHG1, ERK1 (MAPK3), NOTCH1 precursor, Lemd1, Hn1, OATP-C, CKS2, NOTCH1 receptor, RNF11, Cyclin B, Kallikrein 3 (PSA), ZHX3, ERK1/2, 14-3-3, CCDC28A, RBBP7 (RbAp46)
120	Lymphatic Diseases	2773	3.038E-03	1.466E-02	3.038E-03	1.466E-02	19	Tissue kallikreins, ZNF261, LST1, Cyclin B1, NOTCH1 (NICD), PROSC, Olfactory receptor, Notch, NOTCH1 (NEXT), IGHG1, ERK1 (MAPK3), NOTCH1 precursor, CD43, OATP-C, NOTCH1 receptor, Cyclin B, Kallikrein 3 (PSA), ERK1/2, CapZIP
121	Gonadal Disorders	3221	3.203E-03	1.533E-02	3.203E-03	1.533E-02	21	Tissue kallikreins, Kallikrein 1, Cyclin B1, AL1A1, NOTCH1 (NICD), IRT-1, TIP47, Olfactory receptor, Notch, NOTCH1 (NEXT), ERK1 (MAPK3), NOTCH1 precursor, Hn1, OATP-C, NOTCH1 receptor, Cyclin B, Kallikrein 3 (PSA), ZHX3, ERK1/2, 14-3-3, CCDC28A
122	Chondroma	24	3.231E-03	1.534E-02	3.231E-03	1.534E-02	2	ERK1 (MAPK3), ERK1/2
123	Cardiovascular Diseases	3460	3.404E-03	1.578E-02	3.404E-03	1.578E-02	22	Tissue kallikreins, Kallikrein 1, HBG, NOTCH1 (NICD), IRT-1, HBGA, Olfactory receptor, Notch, NOTCH1 (NEXT), IGHG1, ERK1 (MAPK3), NOTCH1 precursor, 14-3-3 zeta/delta, NFKBIL1, OATP-C, CKS2, NOTCH1 receptor, Adult hemoglobin, VILIP3, ERK1/2, 14-3-3, RBBP7 (RbAp46)
124	Pharmacological Phenomena	413	3.40	1.57	3.40	1.57	6	NOTCH1 (NICD), Notch, NOTCH1 (NEXT), NOTCH1 precursor, NOTCH1 receptor, ERK1/2

			7E-03	8E-02	7E-03	8E-02		
125	Drug Resistance	413	3.407E-03	1.578E-02	3.407E-03	1.578E-02	6	NOTCH1 (NICD), Notch, NOTCH1 (NEXT), NOTCH1 precursor, NOTCH1 receptor, ERK1/2
126	Encephalitis, Arbovirus	25	3.504E-03	1.610E-02	3.504E-03	1.610E-02	2	ERK1 (MAPK3), ERK1/2
127	Infertility	290	3.643E-03	1.661E-02	3.643E-03	1.661E-02	5	NOTCH1 (NICD), Notch, NOTCH1 (NEXT), NOTCH1 precursor, NOTCH1 receptor
128	Brain Diseases	2826	3.760E-03	1.701E-02	3.760E-03	1.701E-02	19	Tissue kallikreins, ZNF261, Kallikrein 1, HBG, NOTCH1 (NICD), IRT-1, HBGA, Olfactory receptor, Notch, NOTCH1 (NEXT), ERK1 (MAPK3), NOTCH1 precursor, 14-3-3 zeta/delta, NOTCH1 receptor, RNF11, Adult hemoglobin, VILIP3, ERK1/2, 14-3-3
129	Gastroesophageal Reflux	27	4.082E	1.832E	4.082E	1.832E	2	ERK1 (MAPK3), ERK1/2

			-03	-02	-03	-02		
130	Hemorrhagic Disorders	2024	4.374E-03	1.948E-02	4.374E-03	1.948E-02	15	Tissue kallikreins, HBG, NOTCH1 (NICD), HBGA, Olfactory receptor, Notch, NOTCH1 (NEXT), IGHG1, NOTCH1 precursor, 14-3-3 zeta/delta, CKS2, NOTCH1 receptor, Adult hemoglobin, 14-3-3, RBBP7 (RbAp46)
131	Creutzfeldt-Jakob Syndrome	29	4.700E-03	2.077E-02	4.700E-03	2.077E-02	2	IRT-1, 14-3-3
132	Uterine Cervical Neoplasms	3563	4.894E-03	2.145E-02	4.894E-03	2.145E-02	22	Tissue kallikreins, RY1, LRFN1, Cyclin B1, AL1A1, NOTCH1 (NICD), Gpbp1, TIP47, Olfactory receptor, Notch, NOTCH1 (NEXT), ERK1 (MAPK3), CALCOCO2, NOTCH1 precursor, MTL5, NOTCH1 receptor, Cyclin B, Rab-3, ERK1/2, UGDH, 14-3-3, CapZIP
133	Uterine Cervical Diseases	3565	4.927E-03	2.145E-02	4.927E-03	2.145E-02	22	Tissue kallikreins, RY1, LRFN1, Cyclin B1, AL1A1, NOTCH1 (NICD), Gpbp1, TIP47, Olfactory receptor, Notch, NOTCH1 (NEXT), ERK1 (MAPK3), CALCOCO2, NOTCH1 precursor, MTL5, NOTCH1 receptor, Cyclin B, Rab-3, ERK1/2, UGDH, 14-3-3, CapZIP
134	Oligodendroglioma	313	5.026E-0	2.172E-0	5.026E-0	2.172E-0	5	NOTCH1 (NICD), Notch, NOTCH1 (NEXT), NOTCH1 precursor, NOTCH1 receptor

			3	2	3	2		
135	Physiological Phenomena	1098	5.153E-03	2.210E-02	5.153E-03	2.210E-02	10	NOTCH1 (NICD), Notch, NOTCH1 (NEXT), ERK1 (MAPK3), SRB7, NOTCH1 precursor, MTL5, NOTCH1 receptor, RIG-G, ERK1/2
136	Paget Disease, Extramammary	31	5.358E-03	2.281E-02	5.358E-03	2.281E-02	2	ERK1 (MAPK3), ERK1/2
137	Gram-Positive Bacterial Infections	320	5.512E-03	2.329E-02	5.512E-03	2.329E-02	5	TIRAP (Mal), ERK1 (MAPK3), NFKBIL1, Adult hemoglobin, ERK1/2
138	Mouth Diseases	4776	5.565E-03	2.335E-02	5.565E-03	2.335E-02	27	Tissue kallikreins, RY1, LST1, Cyclin B1, NOTCH1 (NICD), SNX1, TIRAP (Mal), Olfactory receptor, Notch, PAP41, NOTCH1 (NEXT), IGHG1, ERK1 (MAPK3), NOTCH1 precursor, 14-3-3 zeta/delta, NFKBIL1, AO7, PLEKHG2, NOTCH1 receptor, Cyclin B, Rab-3, Adult hemoglobin, FAM63A, RIG-G, ERK1/2, DEAF, 14-3-3
139	Hemic and Lymphatic Diseases	4547	5.752E-03	2.396E-02	5.752E-03	2.396E-02	26	Tissue kallikreins, ZNF261, LST1, Cyclin B1, HBG, NOTCH1 (NICD), HBGA, PROSC, Olfactory receptor, Notch, NOTCH1 (NEXT), IGHG1, ERK1 (MAPK3), NOTCH1 precursor, CD43, 14-3-3 zeta/delta, OATP-C, CKS2, NOTCH1 receptor, Cyclin B, Kallikrein 3 (PSA), Adult hemoglobin, ERK1/2, 14-3-3, CapZIP, RBBP7 (RbAp46)

140	Paranasal Sinus Neoplasms	103	5.816E-03	2.405E-02	5.816E-03	2.405E-02	3	Tissue kallikreins, Olfactory receptor, Notch
141	Esophageal Motility Disorders	33	6.056E-03	2.487E-02	6.056E-03	2.487E-02	2	ERK1 (MAPK3), ERK1/2
142	Pathological Conditions, Signs and Symptoms	4332	6.169E-03	2.515E-02	6.169E-03	2.515E-02	25	Tissue kallikreins, Kallikrein 1, LST1, Cyclin B1, NOTCH1 (NICD), IRT-1, Notch, NOTCH1 (NEXT), ERK1 (MAPK3), SRB7, NOTCH1 precursor, 14-3-3 zeta/delta, NFKBIL1, OATP-C, MTL5, NOTCH1 receptor, Cyclin B, Rab-3, Kallikrein 3 (PSA), Adult hemoglobin, Protrudin, RIG-G, ERK1/2, UGDH, 14-3-3
143	Nose Neoplasms	107	6.462E-03	2.617E-02	6.462E-03	2.617E-02	3	Tissue kallikreins, Olfactory receptor, Notch
144	Stomatognathic Diseases	5821	6.583E-03	2.647E-02	6.583E-03	2.647E-02	31	Tissue kallikreins, RY1, LST1, Cyclin B1, AL1A1, NOTCH1 (NICD), SNX1, TIRAP (Mal), Olfactory receptor, Notch, PAP41, NOTCH1 (NEXT), IGHG1, ERK1 (MAPK3), NOTCH1 precursor, 14-3-3 zeta/delta, NFKBIL1, AO7, OATP-C, PLEKHG2, NOTCH1 receptor, Cyclin B, Rab-3, Adult hemoglobin, FAM63A, RIG-G, VILIP3, ZHX3, ERK1/2, DEAF, 14-3-3
14	Pancreatic Diseases	29	6.	2.	6.	2.	19	CDH26, Tissue kallikreins, IGHG4, Cyclin B1, AL1A1, LHX1, HBG, HBGA, Olfactory receptor, Notch, IGHG1, ERK1 (MAPK3), Lemd1,

5		80	720E-03	683E-02	720E-03	683E-02		NFKBIL1, Cyclin B, Adult hemoglobin, ERK1/2, 14-3-3, RBBP7 (RbAp46)
146	Bone Resorption	110	6.974E-03	2.766E-02	6.974E-03	2.766E-02	3	Tissue kallikreins, Notch, Kallikrein 3 (PSA)
147	Multiple Endocrine Neoplasia Type 2b	2	7.052E-03	2.777E-02	7.052E-03	2.777E-02	1	RNF11
148	Central Nervous System Diseases	3004	7.320E-03	2.864E-02	7.320E-03	2.864E-02	19	Tissue kallikreins, ZNF261, Kallikrein 1, HBG, NOTCH1 (NICD), IRT-1, HBGA, Olfactory receptor, Notch, NOTCH1 (NEXT), ERK1 (MAPK3), NOTCH1 precursor, 14-3-3 zeta/delta, NOTCH1 receptor, RNF11, Adult hemoglobin, VILIP3, ERK1/2, 14-3-3
149	Deglutition Disorders	37	7.570E-03	2.942E-02	7.570E-03	2.942E-02	2	ERK1 (MAPK3), ERK1/2
150	Paget's Disease, Mammary	38	7.97	3.07	7.97	3.07	2	ERK1 (MAPK3), ERK1/2

			2E-03	7E-02	2E-03	7E-02		
151	Aneuploidy	118	8.454E-03	3.242E-02	8.454E-03	3.242E-02	3	Tissue kallikreins, Kallikrein 3 (PSA), 14-3-3
152	Mental Disorders	1571	8.748E-03	3.332E-02	8.748E-03	3.332E-02	12	Tissue kallikreins, Kallikrein 1, AL1A1, NOTCH1 (NICD), Olfactory receptor, Notch, NOTCH1 (NEXT), NOTCH1 precursor, 14-3-3 zeta/delta, NOTCH1 receptor, Spinophilin, 14-3-3
153	Hair Diseases	120	8.850E-03	3.349E-02	8.850E-03	3.349E-02	3	Tissue kallikreins, Notch, Kallikrein 3 (PSA)
154	Lung Diseases, Interstitial	362	9.146E-03	3.423E-02	9.146E-03	3.423E-02	5	NOTCH1 (NICD), Notch, NOTCH1 (NEXT), NOTCH1 precursor, NOTCH1 receptor
155	Nutritional and Metabolic Diseases	3070	9.203E	3.423E	9.203E	3.423E	19	Tissue kallikreins, Kallikrein 1, Cyclin B1, NOTCH1 (NICD), Notch, NOTCH1 (NEXT), ERK1 (MAPK3), SRB7, NOTCH1 precursor, NFKBIL1, OATP-C, MTL5, NOTCH1 receptor, Cyclin B, Adult hemoglobin, RIG-G, ZHX3, ERK1/2, 14-3-3

			-03	-02	-03	-02		
156	Depressive Disorder, Major	509	9.224E-03	3.423E-02	9.224E-03	3.423E-02	6	NOTCH1 (NICD), Olfactory receptor, Notch, NOTCH1 (NEXT), NOTCH1 precursor, NOTCH1 receptor
157	Bacterial Infections	516	9.824E-03	3.623E-02	9.824E-03	3.623E-02	6	TIRAP (Mal), ERK1 (MAPK3), NFKBIL1, Adult hemoglobin, ERK1/2, 14-3-3
158	CADASIL	3	1.056E-02	3.782E-02	1.056E-02	3.782E-02	1	Notch
159	Mycobacterium Infections, Nontuberculous	3	1.056E-02	3.782E-02	1.056E-02	3.782E-02	1	Adult hemoglobin
160	Multiple Endocrine Neoplasia Type 2a	3	1.056E-0	3.782E-0	1.056E-0	3.782E-0	1	RNF11

			2	2	2	2		
161	Spastic Paraplegia, Autosomal Dominant	3	1.056E-02	3.782E-02	1.056E-02	3.782E-02	1	Protrudin
162	Tuberous Sclerosis	44	1.058E-02	3.782E-02	1.058E-02	3.782E-02	2	ERK1/2, 14-3-3
163	Depressive Disorder	542	1.230E-02	4.369E-02	1.230E-02	4.369E-02	6	NOTCH1 (NICD), Olfactory receptor, Notch, NOTCH1 (NEXT), NOTCH1 precursor, NOTCH1 receptor
164	Neoplasms, Cystic, Mucinous, and Serous	48	1.250E-02	4.415E-02	1.250E-02	4.415E-02	2	ERK1 (MAPK3), ERK1/2
165	Psychiatry and Psychology	1864	1.310E-02	4.597E-02	1.310E-02	4.597E-02	13	Tissue kallikreins, Kallikrein 1, AL1A1, NOTCH1 (NICD), Olfactory receptor, Notch, NOTCH1 (NEXT), NOTCH1 precursor, 14-3-3 zeta/delta, NOTCH1 receptor, Spinophilin, Rab-3, 14-3-3

166	Inflammation	891	1.321E-02	4.608E-02	1.321E-02	4.608E-02	8	Tissue kallikreins, Kallikrein 1, LST1, ERK1 (MAPK3), 14-3-3 zeta/delta, Adult hemoglobin, ERK1/2, 14-3-3
167	Hypertrophy	262	1.391E-02	4.816E-02	1.391E-02	4.816E-02	4	Tissue kallikreins, ERK1 (MAPK3), ERK1/2, 14-3-3
168	Autoimmune Diseases of the Nervous System	1084	1.397E-02	4.816E-02	1.397E-02	4.816E-02	9	Tissue kallikreins, NOTCH1 (NICD), IRT-1, Notch, NOTCH1 (NEXT), NOTCH1 precursor, NOTCH1 receptor, ERK1/2, 14-3-3
169	Anemia, Sickle Cell	144	1.446E-02	4.955E-02	1.446E-02	4.955E-02	3	HBG, HBGA, Adult hemoglobin

Table 10S: Enrichment analysis report. Enrichment by Diseases (by Biomarkers) (healthy control vs SVZn)

Enrichment by Diseases (by Biomarkers)					SVZn Limma Input_genelist			
**#**	**Diseases**	**Total**	**pValue**	**Min FDR**	**p-value**	**FDR**	**In Data**	**Network Objects from Active Data**
1	Leukemia, Myeloid, Chronic-Phase	19	1.928E-08	1.155E-05	1.928E-08	1.155E-05	5	HSPA1B, HSP70, HSPA2, HSPA1A, HSC70
2	Hearing Loss, Noise-Induced	13	2.772E-07	8.302E-05	2.772E-07	8.302E-05	4	HSPA1B, HSP70, HSPA2, HSPA1A
3	Leukemia, Biphenotypic, Acute	34	4.371E-07	8.728E-05	4.371E-07	8.728E-05	5	HSPA1B, HSP70, HSPA2, HSPA1A, HSC70
4	Abscess	6	1.799E-06	2.694E-04	1.799E-06	2.694E-04	3	HSPA1B, HSP70, HSPA1A
5	Leprosy, Tuberculoid	12	1.940E-05	2.324E-03	1.940E-05	2.324E-03	3	HSPA1B, HSP70, HSPA1A
6	Pulmonary Edema	13	2.5	2.5	2.5	2.5	3	HSPA1B, HSP70, HSPA1A

			13E-05	09E-03	13E-05	09E-03		
7	Leprosy, Paucibacillary	14	3.188E-05	2.728E-03	3.188E-05	2.728E-03	3	HSPA1B, HSP70, HSPA1A
8	Fistula	16	4.873E-05	3.260E-03	4.873E-05	3.260E-03	3	HSPA1B, HSP70, HSPA1A
9	Leukemia, Myelogenous, Chronic, BCR-ABL Positive	210	4.898E-05	3.260E-03	4.898E-05	3.260E-03	7	HSPA1B, HSP70, SET, HSPA2, HSPA1A, SET/NUP214 fusion protein, HSC70
10	Colonic Diseases	4272	7.243E-05	4.339E-03	7.243E-05	4.339E-03	36	Annexin V, eEF1A, RBBP8 (CtIP), IGHG4, Cyclin B1, HSPA1B, ECE1, AL1A1, HDAC7, MENA, SNX1, Histone deacetylase class II, Olfactory receptor, Thioredoxin-like 2, Calmyrin, EPS8L1, HSP70, IGHG1, eEF2K, TXNRD1, SMYD3, Calmodulin, HSPA2, HSPA1A, CKS2, Annexin XI, Cyclin B, GMEB1, PTPN2, Fructosamine-3-kinase, PP2C, HSC70, TARDBP (TDP43), PRCC, RUVBL1, VILIP1
11	Multiple Trauma	19	8.349E-05	4.546E-03	8.349E-05	4.546E-03	3	HSPA1B, HSP70, HSPA1A
12	Myeloproliferative Disorders	322	1.108E-04	5.532E-03	1.108E-04	5.532E-03	8	HSPA1B, HSP70, SET, HSPA2, HSPA1A, SET/NUP214 fusion protein, FOP, HSC70
1	Acute	5	1.	5.	1.	5.	4	HSPA1B, HSP70, HSPA1A, HSC70

3	Kidney Injury	6	2.26E-04	6.49E-03	2.26E-04	6.49E-03		
14	Urinary Tract Infections	23	1.506E-04	6.014E-03	1.506E-04	6.014E-03	3	HSPA1B, HSP70, HSPA1A
15	Heat Stress Disorders	23	1.506E-04	6.014E-03	1.506E-04	6.014E-03	3	HSPA1B, HSP70, HSPA1A
16	Suppuration	25	1.943E-04	7.274E-03	1.943E-04	7.274E-03	3	HSPA1B, HSP70, HSPA1A
17	Pancreatitis, Alcoholic	26	2.189E-04	7.713E-03	2.189E-04	7.713E-03	3	HSPA1B, HSP70, HSPA1A
18	Diabetic Ketoacidosis	27	2.455E-04	8.168E-03	2.455E-04	8.168E-03	3	HSPA1B, HSP70, HSPA1A
19	Ketosis	30	3.374E-04	1.036E-02	3.374E-04	1.036E-02	3	HSPA1B, HSP70, HSPA1A

20	Nasopharyngeal Diseases	135	3.793E-04	1.036E-02	3.793E-04	1.036E-02	5	Annexin V, HSPA1B, AL1A1, HSP70, HSPA1A
21	Nasopharyngeal Neoplasms	135	3.793E-04	1.036E-02	3.793E-04	1.036E-02	5	Annexin V, HSPA1B, AL1A1, HSP70, HSPA1A
22	Rotavirus Infections	7	4.224E-04	1.036E-02	4.224E-04	1.036E-02	2	HSP70, HSC70
23	Mycoplasma Infections	7	4.224E-04	1.036E-02	4.224E-04	1.036E-02	2	eEF1A, eEF1A1
24	Mycoplasmatales Infections	7	4.224E-04	1.036E-02	4.224E-04	1.036E-02	2	eEF1A, eEF1A1
25	Colonic Neoplasms	1232	4.322E-04	1.036E-02	4.322E-04	1.036E-02	15	Annexin V, IGHG4, Cyclin B1, HSPA1B, ECE1, AL1A1, SNX1, Histone deacetylase class II, HSP70, IGHG1, HSPA2, HSPA1A, Cyclin B, HSC70, RUVBL1
26	Duodenal Ulcer	34	4.907E-04	1.089E-02	4.907E-04	1.089E-02	3	HSPA1B, HSP70, HSPA1A

27	Altitude Sickness	34	4.907E-04	1.089E-02	4.907E-04	1.089E-02	3	HSPA1B, HSP70, HSPA1A
28	Acidosis	82	5.335E-04	1.139E-02	5.335E-04	1.139E-02	4	HSPA1B, HSP70, HSPA1A, PP2C
29	Reoviridae Infections	8	5.615E-04	1.139E-02	5.615E-04	1.139E-02	2	HSP70, HSC70
30	Bacterial Infections	516	5.706E-04	1.139E-02	5.706E-04	1.139E-02	9	eEF1A, PGMU, HSPA1B, HSP70, TXNRD1, HSPA2, HSPA1A, eEF1A1, PP2C
31	Acid-Base Imbalance	88	6.966E-04	1.346E-02	6.966E-04	1.346E-02	4	HSPA1B, HSP70, HSPA1A, PP2C
32	Craniofacial Abnormalities	237	7.479E-04	1.400E-02	7.479E-04	1.400E-02	6	Histone deacetylase class II, ICMT, EYA1, PP2C, TCOF1, PQBP-1
33	Agranulocytosis	41	8.543E-04	1.551E-02	8.543E-04	1.551E-02	3	HSPA1B, HSP70, HSPA1A

34	Hearing Loss, Sensorineural	95	9.283E-04	1.635E-02	9.283E-04	1.635E-02	4	HSPA1B, HSP70, HSPA2, HSPA1A
35	Varicocele	11	1.093E-03	1.871E-02	1.093E-03	1.871E-02	2	HSP70, HSPA2
36	Thyroiditis, Autoimmune	102	1.210E-03	2.013E-02	1.210E-03	2.013E-02	4	HSPA1B, ECE1, HSP70, HSPA1A
37	Craniofacial Dysostosis	12	1.308E-03	2.117E-02	1.308E-03	2.117E-02	2	ICMT, TCOF1
38	Neoplastic Processes	728	1.741E-03	2.745E-02	1.741E-03	2.745E-02	10	Cyclin B1, PSMD10 (Gankyrin), MENA, HSP70, SET, Follistatin, SMYD3, SET/NUP214 fusion protein, Cyclin B, VILIP1
39	Ganglioglioma	14	1.793E-03	2.753E-02	1.793E-03	2.753E-02	2	Cyclin B1, Cyclin B
40	Spondylitis, Ankylosing	118	2.067E-03	2.969E-02	2.067E-03	2.969E-02	4	HSPA1B, HSP70, HSPA2, HSPA1A

41	Leukemia, Myeloid, Acute	1292	2.069E-03	2.969E-02	2.069E-03	2.969E-02	14	HSPA1B, Fbxo3, Olfactory receptor, HSP70, SET, HSPA2, HSPA1A, CKS2, Septin 3, SET/NUP214 fusion protein, C10orf118, PP2C, HSC70, TCOF1
42	Bacterial Infections and Mycoses	1294	2.099E-03	2.969E-02	2.099E-03	2.969E-02	14	eEF1A, RBBP8 (CtIP), PGMU, Cyclin T1, HSPA1B, Histone deacetylase class II, HSP70, Follistatin, TXNRD1, HSPA2, HSPA1A, eEF1A1, PP2C, HSC70
43	Ankylosis	119	2.131E-03	2.969E-02	2.131E-03	2.969E-02	4	HSPA1B, HSP70, HSPA2, HSPA1A
44	Immune System Diseases	6037	2.204E-03	3.000E-02	2.204E-03	3.000E-02	41	IGHM, eEF1A, Reticulon 4, PGMU, IGHG4, Cyclin T1, Cyclin B1, HSPA1B, Copine-4, AL1A1, Beta crystallin B2, MENA, CDO1, HEXIM1, DARS, Histone deacetylase class II, PROSC, Olfactory receptor, HSP70, IGHG1, Annexin VI, CLEC2D, Follistatin, Calmodulin, HSPA2, HSPA1A, RBP1, eEF1A1, CKS2, Annexin XI, SET/NUP214 fusion protein, Cyclin B, EYA1, GMEB1, NTB-A, PTPN2, RIG-G, PP2C, HSC70, TARDBP (TDP43), CapZIP
45	Shock, Septic	62	2.829E-03	3.684E-02	2.829E-03	3.684E-02	3	HSPA1B, HSP70, HSPA1A
46	Leukopenia	62	2.829E-03	3.684E-02	2.829E-03	3.684E-02	3	HSPA1B, HSP70, HSPA1A
47	Thyroiditis	130	2.935E-03	3.695E-02	2.935E-03	3.695E-02	4	HSPA1B, ECE1, HSP70, HSPA1A

48	Edema	63	2.961E-03	3.695E-02	2.961E-03	3.695E-02	3	HSPA1B, HSP70, HSPA1A
49	Pneumonia, Bacterial	19	3.319E-03	4.058E-02	3.319E-03	4.058E-02	2	HSP70, HSPA2
50	Pathologic Processes	2678	3.898E-03	4.670E-02	3.898E-03	4.670E-02	22	Annexin V, eEF1A, Cyclin B1, HSPA1B, PSMD10 (Gankyrin), ECE1, MENA, HSP70, SET, Follistatin, SMYD3, Calmodulin, HSPA2, HSPA1A, eEF1A1, SET/NUP214 fusion protein, Cyclin B, FOP, PP2C, HSC70, TARDBP (TDP43), VILIP1
51	Seizures, Febrile	70	3.989E-03	4.685E-02	3.989E-03	4.685E-02	3	HSPA1B, HSP70, HSPA1A
52	Retrognathia	1	4.541E-03	5.081E-02	4.541E-03	5.081E-02	1	EYA1
53	Funnel Chest	1	4.541E-03	5.081E-02	4.541E-03	5.081E-02	1	EYA1
54	Musculoskeletal Abnormalities	340	4.580E-03	5.081E-02	4.580E-03	5.081E-02	6	Histone deacetylase class II, ICMT, EYA1, PP2C, TCOF1, PQBP-1

55	Blast Crisis	23	4.853E-03	5.286E-02	4.853E-03	5.286E-02	2	SET, SET/NUP214 fusion protein
56	Hearing Loss	252	5.877E-03	6.286E-02	5.877E-03	6.286E-02	5	HSPA1B, HSP70, HSPA2, HSPA1A, PP2C
57	Leprosy	81	5.999E-03	6.304E-02	5.999E-03	6.304E-02	3	HSPA1B, HSP70, HSPA1A
58	Shock	82	6.206E-03	6.368E-02	6.206E-03	6.368E-02	3	HSPA1B, HSP70, HSPA1A
59	Hearing Disorders	256	6.273E-03	6.368E-02	6.273E-03	6.368E-02	5	HSPA1B, HSP70, HSPA2, HSPA1A, PP2C
60	Sepsis	260	6.687E-03	6.583E-02	6.687E-03	6.583E-02	5	HSPA1B, HSP70, Follistatin, HSPA2, HSPA1A
61	Infection	1170	6.704E-03	6.583E-02	6.704E-03	6.583E-02	12	eEF1A, RBBP8 (CtIP), Cyclin T1, HSPA1B, Histone deacetylase class II, HSP70, Follistatin, HSPA2, HSPA1A, eEF1A1, PP2C, HSC70

62	Systemic Inflammatory Response Syndrome	263	7.011E-03	6.773E-02	7.011E-03	6.773E-02	5	HSPA1B, HSP70, Follistatin, HSPA2, HSPA1A
63	Leishmaniasis, Visceral	28	7.145E-03	6.780E-02	7.145E-03	6.780E-02	2	HSP70, HSPA2
64	Leukemia, Myeloid	1488	7.244E-03	6.780E-02	7.244E-03	6.780E-02	14	HSPA1B, Fbxo3, Olfactory receptor, HSP70, SET, HSPA2, HSPA1A, CKS2, Septin 3, SET/NUP214 fusion protein, C10orf118, PP2C, HSC70, TCOF1
65	Duodenal Diseases	89	7.779E-03	7.169E-02	7.779E-03	7.169E-02	3	HSPA1B, HSP70, HSPA1A
66	Alcohol-Induced Disorders	90	8.021E-03	7.280E-02	8.021E-03	7.280E-02	3	HSPA1B, HSP70, HSPA1A
67	Crohn Disease	506	8.284E-03	7.401E-02	8.284E-03	7.401E-02	7	HSPA1B, HSP70, CLEC2D, HSPA2, HSPA1A, PTPN2, FOP
68	Musculoskeletal Diseases	2511	8.476E-03	7.401E-02	8.476E-03	7.401E-02	20	IGHG4, HSPA1B, DARS, Histone deacetylase class II, HSP70, IGHG1, Annexin VI, Follistatin, Calmodulin, HSPA2, HSPA1A, ICMT, EYA1, NTB-A, PTPN2, RIG-G, PP2C, TARDBP (TDP43), TCOF1, PQBP-1

69	Delirium, Dementia, Amnestic, Cognitive Disorders	176	8.526E-03	7.401E-02	8.526E-03	7.401E-02	4	HSPA1B, HSP70, HSPA1A, TARDBP (TDP43)
70	Birth Weight	93	8.774E-03	7.500E-02	8.774E-03	7.500E-02	3	HSPA1B, HSP70, HSPA1A
71	Herpes Simplex	94	9.034E-03	7.500E-02	9.034E-03	7.500E-02	3	Histone deacetylase class II, HSP70, HSC70
72	Cataract, Lamellar	2	9.062E-03	7.500E-02	9.062E-03	7.500E-02	1	Beta crystallin B2
73	Carcinogenesis	32	9.266E-03	7.500E-02	9.266E-03	7.500E-02	2	SET, SET/NUP214 fusion protein
74	Cell Transformation, Neoplastic	32	9.266E-03	7.500E-02	9.266E-03	7.500E-02	2	SET, SET/NUP214 fusion protein
75	Bone Marrow Diseases	785	9.409E-03	7.515E-02	9.409E-03	7.515E-02	9	HSPA1B, HSP70, SET, HSPA2, HSPA1A, SET/NUP214 fusion protein, FOP, PP2C, HSC70

76	Actinomycetales Infections	285	9.723E-03	7.663E-02	9.723E-03	7.663E-02	5	PGMU, HSPA1B, HSP70, TXNRD1, HSPA1A
77	Neoplasm Metastasis	676	1.186E-02	9.227E-02	1.186E-02	9.227E-02	8	Cyclin B1, PSMD10 (Gankyrin), MENA, HSP70, Follistatin, SMYD3, Cyclin B, VILIP1
78	Sarcoidosis	195	1.208E-02	9.277E-02	1.208E-02	9.277E-02	4	HSPA1B, HSP70, IGHG1, HSPA1A
79	Arthritis	1597	1.301E-02	9.671E-02	1.301E-02	9.671E-02	14	IGHG4, HSPA1B, DARS, Histone deacetylase class II, HSP70, IGHG1, Annexin VI, Calmodulin, HSPA2, HSPA1A, EYA1, NTB-A, PTPN2, RIG-G
80	Peptic Ulcer	109	1.348E-02	9.671E-02	1.348E-02	9.671E-02	3	HSPA1B, HSP70, HSPA1A
81	Leishmaniasis	39	1.356E-02	9.671E-02	1.356E-02	9.671E-02	2	HSP70, HSPA2
82	Marinesco-Sjogren Syndrome	3	1.356E-02	9.671E-02	1.356E-02	9.671E-02	1	HSP70

83	Branchio-Oto-Renal Syndrome	3	1.356E-02	9.671E-02	1.356E-02	9.671E-02	1	EYA1
84	LEOPARD Syndrome	3	1.356E-02	9.671E-02	1.356E-02	9.671E-02	1	PP2C
85	Carcinoma, Non-Small-Cell Lung	2297	1.473E-02	1.027E-01	1.473E-02	1.027E-01	18	eEF1A, Cyclin B1, HSPA1B, ECE1, AL1A1, Cytoglobin, HSP70, TXNRD1, Calmodulin, HSPA2, HSPA1A, RBP1, eEF1A1, Cyclin B, PP2C, HSC70, RUVBL1, VILIP1
86	Spondylarthropathies	207	1.475E-02	1.027E-01	1.475E-02	1.027E-01	4	HSPA1B, HSP70, HSPA2, HSPA1A
87	Joint Diseases	1627	1.510E-02	1.040E-01	1.510E-02	1.040E-01	14	IGHG4, HSPA1B, DARS, Histone deacetylase class II, HSP70, IGHG1, Annexin VI, Calmodulin, HSPA2, HSPA1A, EYA1, NTB-A, PTPN2, RIG-G
88	Gram-Positive Bacterial Infections	320	1.540E-02	1.048E-01	1.540E-02	1.048E-01	5	PGMU, HSPA1B, HSP70, TXNRD1, HSPA1A
89	Spondylitis	212	1.597E-02	1.061E-01	1.597E-02	1.061E-01	4	HSPA1B, HSP70, HSPA2, HSPA1A

90	Spondylarthritis	212	1.597E-02	1.061E-01	1.597E-02	1.061E-01	4	HSPA1B, HSP70, HSPA2, HSPA1A
91	Small Cell Lung Carcinoma	446	1.611E-02	1.061E-01	1.611E-02	1.061E-01	6	Cyclin B1, HSPA1B, HSP70, HSPA1A, Cyclin B, PP2C
92	Leukemia	3224	1.673E-02	1.089E-01	1.673E-02	1.089E-01	23	IGHM, eEF1A, Reticulon 4, Cyclin B1, HSPA1B, Copine-4, Fbxo3, PROSC, Olfactory receptor, HSP70, SET, HSPA2, HSPA1A, eEF1A1, CKS2, Septin 3, SET/NUP214 fusion protein, Cyclin B, C10orf118, PP2C, HSC70, TCOF1, CapZIP
93	Sensation Disorders	328	1.696E-02	1.089E-01	1.696E-02	1.089E-01	5	HSPA1B, HSP70, HSPA2, HSPA1A, PP2C
94	Epilepsy	452	1.710E-02	1.089E-01	1.710E-02	1.089E-01	6	Reticulon 4, HSPA1B, HSPBAP1, HSP70, HSPA1A, HSC70
95	Pulmonary Valve Stenosis	4	1.804E-02	1.111E-01	1.804E-02	1.111E-01	1	PP2C
96	Mandibulofacial Dysostosis	4	1.804E-02	1.111E-01	1.804E-02	1.111E-01	1	TCOF1

97	Hallermann's Syndrome	4	1.804E-02	1.111E-01	1.804E-02	1.111E-01	1	ICMT
98	Skin Diseases, Viral	122	1.817E-02	1.111E-01	1.817E-02	1.111E-01	3	Histone deacetylase class II, HSP70, HSC70
99	Precursor Cell Lymphoblastic Leukemia-Lymphoma	605	2.034E-02	1.230E-01	2.034E-02	1.230E-01	7	HSPA1B, Olfactory receptor, HSP70, HSPA1A, SET/NUP214 fusion protein, HSC70, CapZIP
100	Alcohol-Related Disorders	347	2.106E-02	1.261E-01	2.106E-02	1.261E-01	5	HSPA1B, AL1A1, Olfactory receptor, HSP70, HSPA1A
101	Carcinoma, Bronchogenic	2400	2.210E-02	1.309E-01	2.210E-02	1.309E-01	18	eEF1A, Cyclin B1, HSPA1B, ECE1, AL1A1, Cytoglobin, HSP70, TXNRD1, Calmodulin, HSPA2, HSPA1A, RBP1, eEF1A1, Cyclin B, PP2C, HSC70, RUVBL1, VILIP1
102	Bronchial Neoplasms	2403	2.236E-02	1.309E-01	2.236E-02	1.309E-01	18	eEF1A, Cyclin B1, HSPA1B, ECE1, AL1A1, Cytoglobin, HSP70, TXNRD1, Calmodulin, HSPA2, HSPA1A, RBP1, eEF1A1, Cyclin B, PP2C, HSC70, RUVBL1, VILIP1
103	Uveitis, Intermediate	5	2.250E-02	1.309E-01	2.250E-02	1.309E-01	1	HSP70

104	Lymphoma, T-Cell, Cutaneous	134	2.322E-02	1.329E-01	2.322E-02	1.329E-01	3	CDO1, Histone deacetylase class II, PP2C
105	Lymphatic Diseases	2773	2.330E-02	1.329E-01	2.330E-02	1.329E-01	20	IGHM, eEF1A, Reticulon 4, Cyclin B1, HSPA1B, Copine-4, CDO1, Histone deacetylase class II, PROSC, Olfactory receptor, HSP70, IGHG1, HSPA2, HSPA1A, eEF1A1, SET/NUP214 fusion protein, Cyclin B, PP2C, HSC70, CapZIP
106	Esophageal Neoplasms	495	2.537E-02	1.429E-01	2.537E-02	1.429E-01	6	Cyclin B1, HSP70, DPP3, CKS2, Cyclin B, VILIP1
107	Graft vs Host Disease	139	2.552E-02	1.429E-01	2.552E-02	1.429E-01	3	HSPA1B, HSP70, HSPA1A
108	Cafe-au-Lait Spots	6	2.694E-02	1.481E-01	2.694E-02	1.481E-01	1	PP2C
109	Leukocyte Disorders	142	2.696E-02	1.481E-01	2.696E-02	1.481E-01	3	HSPA1B, HSP70, HSPA1A
110	Retinoblastoma	148	2.996E-02	1.632E-01	2.996E-02	1.632E-01	3	RBBP8 (CtIP), ARID4A, TARDBP (TDP43)

111	Retinal Neoplasms	149	3.048E-02	1.640E-01	3.048E-02	1.640E-01	3	RBBP8 (CtIP), ARID4A, TARDBP (TDP43)
112	Neoplasms, Neuroepithelial	5355	3.066E-02	1.640E-01	3.066E-02	1.640E-01	33	eEF1A, Reticulon 4, RBBP8 (CtIP), Cyclin B1, TIPIN, ECE1, Copine-4, Fbxo3, DARS, Histone deacetylase class II, PROSC, Olfactory receptor, EPS8L1, HSP70, Cyclophilin E, ARID4A, eEF2K, COASY, eEF1A1, CKS2, OR10G3, DOHH, SET/NUP214 fusion protein, Cyclin B, GMEB1, NTB-A, RIG-G, Fructosamine-3-kinase, PP2C, HSC70, TARDBP (TDP43), CapZIP, VILIP1
113	Leukemia, Lymphoid	1963	3.131E-02	1.650E-01	3.131E-02	1.650E-01	15	IGHM, eEF1A, Reticulon 4, HSPA1B, Copine-4, PROSC, Olfactory receptor, HSP70, HSPA2, HSPA1A, eEF1A1, SET/NUP214 fusion protein, PP2C, HSC70, CapZIP
114	Bone Diseases, Infectious	61	3.141E-02	1.650E-01	3.141E-02	1.650E-01	2	HSP70, HSPA2
115	Dysostoses	62	3.236E-02	1.682E-01	3.236E-02	1.682E-01	2	ICMT, TCOF1
116	Intestinal Diseases	9340	3.275E-02	1.682E-01	3.275E-02	1.682E-01	52	Annexin V, eEF1A, RBBP8 (CtIP), PGMU, IGHG4, RY1, Cyclin B1, HSPA1B, ECE1, Copine-4, AL1A1, HDAC7, MENA, SNX1, Histone deacetylase class II, Olfactory receptor, Thioredoxin-like 2, Calmyrin, EPS8L1, COLEC12, HSP70, IGHG1, ARID4A, CLEC2D, eEF2K, Follistatin, TXNRD1, SMYD3, Calmodulin, HSPA2, HSPA1A, LOH11CR2A, CKS2, Annexin XI, SET/NUP214 fusion protein, Cyclin B, IMD2, GMEB1, NTB-A, FAM63A, PTPN2, FOP, RIG-G, Fructosamine-3-kinase, PP2C, HSC70, TARDBP (TDP43), PRCC, TCOF1, RUVBL1, CapZIP, VILIP1
117	Death	154	3.314E-	1.682E-	3.314E-	1.682E-	3	Annexin V, ECE1, PP2C

			02	01	02	01		
118	Leukemia-Lymphoma, Adult T-Cell	154	3.314E-02	1.682E-01	3.314E-02	1.682E-01	3	Reticulon 4, SET/NUP214 fusion protein, PP2C
119	Alzheimer Disease	1141	3.435E-02	1.721E-01	3.435E-02	1.721E-01	10	Reticulon 4, HSPA1B, ECE1, HSP70, eEF2K, HSPA2, HSPA1A, Septin 3, TARDBP (TDP43), VILIP1
120	Ear Diseases	396	3.448E-02	1.721E-01	3.448E-02	1.721E-01	5	HSPA1B, HSP70, HSPA2, HSPA1A, PP2C
121	Tongue Neoplasms	65	3.528E-02	1.735E-01	3.528E-02	1.735E-01	2	Cyclin B1, Cyclin B
122	Mycobacterium Infections	272	3.553E-02	1.735E-01	3.553E-02	1.735E-01	4	PGMU, HSPA1B, HSP70, HSPA1A
123	Spinal Diseases	273	3.593E-02	1.735E-01	3.593E-02	1.735E-01	4	HSPA1B, HSP70, HSPA2, HSPA1A
124	Neoplasms	20696	3.618E	1.735E	3.618E	1.735E	98	Annexin V, IGHM, P5CR1, eEF1A, Reticulon 4, RBBP8 (CtIP), PGMU, IGHG4, Cyclin T1, RY1, Cyclin B1, DLC1 (Dynein LC8a), HSPA1B, PSMD10 (Gankyrin), TIPIN, Clp1, ECE1, Copine-4, AL1A1, Fbxo3, PPM1F, Beta crystallin B2, HDAC7, MENA, CDO1, Cytoglobin, SNX1, HEXIM1, DARS, VAMP5, Histone

			-02	-01	-02	-01		deacetylase class II, PROSC, Olfactory receptor, Thioredoxin-like 2, HSPBAP1, Calmyrin, EPS8L1, COLEC12, HSP70, IGHG1, Cyclophilin E, ARID4A, Annexin VI, PGM2, CLEC2D, eEF2K, ZFYVE19, SET, COASY, Follistatin, DPP3, TXNRD1, SMYD3, POLE3-POLE4 complex, Calmodulin, HSPA2, HSPA1A, RBP1, B230118H07Rik, eEF1A1, SAE2, LOH11CR2A, CKS2, OR10G3, OBFC1, ZNF397OS, Annexin XI, POLE3 (YBL1), DYNLL, ICMT, CBWD1, DOHH, ZNF397, Septin 3, SET/NUP214 fusion protein, Cyclin B, IMD2, C10orf118, GMEB1, Pleckstrin, NTB-A, FAM63A, PTPN2, FOP, RIG-G, PDXP, CAMK2N1, Fructosamine-3-kinase, PP2C, C6orf115, HSC70, TARDBP (TDP43), PRCC, TCOF1, RUVBL1, CapZIP, PQBP-1, VILIP1
125	Euglenozoa Infections	66	3.628E-02	1.735E-01	3.628E-02	1.735E-01	2	HSP70, HSPA2
126	Tauopathies	1153	3.650E-02	1.735E-01	3.650E-02	1.735E-01	10	Reticulon 4, HSPA1B, ECE1, HSP70, eEF2K, HSPA2, HSPA1A, Septin 3, TARDBP (TDP43), VILIP1
127	Gastrointestinal Diseases	10506	3.812E-02	1.789E-01	3.812E-02	1.789E-01	57	Annexin V, eEF1A, RBBP8 (CtIP), PGMU, IGHG4, RY1, Cyclin B1, HSPA1B, ECE1, Copine-4, AL1A1, Fbxo3, HDAC7, MENA, SNX1, Histone deacetylase class II, Olfactory receptor, Thioredoxin-like 2, Calmyrin, EPS8L1, COLEC12, HSP70, IGHG1, ARID4A, Annexin VI, PGM2, CLEC2D, eEF2K, Follistatin, DPP3, TXNRD1, SMYD3, Calmodulin, HSPA2, HSPA1A, RBP1, LOH11CR2A, CKS2, Annexin XI, SET/NUP214 fusion protein, Cyclin B, IMD2, GMEB1, NTB-A, FAM63A, PTPN2, FOP, RIG-G, Fructosamine-3-kinase, PP2C, HSC70, TARDBP (TDP43), PRCC, TCOF1, RUVBL1, CapZIP, VILIP1
128	Arrhythmias, Cardiac	163	3.822E-02	1.789E-01	3.822E-02	1.789E-01	3	ECE1, HSP70, HSC70
129	Tongue Diseases	69	3.934E-02	1.827E-01	3.934E-02	1.827E-01	2	Cyclin B1, Cyclin B
13	Autoimmune	27	3.9	1.8	3.9	1.8	19	Reticulon 4, PGMU, IGHG4, HSPA1B, DARS, Histone deacetylase class II, Olfactory receptor, HSP70, IGHG1, Annexin

0	Diseases	51	78E-02	33E-01	78E-02	33E-01		VI, CLEC2D, Calmodulin, HSPA1A, RBP1, Annexin XI, EYA1, NTB-A, PTPN2, RIG-G
131	Colorectal Neoplasms	9025	4.153E-02	1.899E-01	4.153E-02	1.899E-01	50	Annexin V, eEF1A, RBBP8 (CtIP), PGMU, IGHG4, RY1, Cyclin B1, HSPA1B, ECE1, Copine-4, AL1A1, HDAC7, MENA, SNX1, Histone deacetylase class II, Olfactory receptor, Thioredoxin-like 2, Calmyrin, EPS8L1, COLEC12, HSP70, IGHG1, ARID4A, eEF2K, Follistatin, TXNRD1, SMYD3, Calmodulin, HSPA2, HSPA1A, LOH11CR2A, CKS2, Annexin XI, SET/NUP214 fusion protein, Cyclin B, IMD2, GMEB1, NTB-A, FAM63A, FOP, RIG-G, Fructosamine-3-kinase, PP2C, HSC70, TARDBP (TDP43), PRCC, TCOF1, RUVBL1, CapZIP, VILIP1
132	Psychiatry and Psychology	1864	4.199E-02	1.906E-01	4.199E-02	1.906E-01	14	eEF1A, Reticulon 4, HSPA1B, AL1A1, Histone deacetylase class II, Olfactory receptor, HSP70, Calmodulin, HSPA1A, eEF1A1, HSC70, TARDBP (TDP43), PQBP-1, VILIP1
133	Intestinal Neoplasms	9036	4.246E-02	1.912E-01	4.246E-02	1.912E-01	50	Annexin V, eEF1A, RBBP8 (CtIP), PGMU, IGHG4, RY1, Cyclin B1, HSPA1B, ECE1, Copine-4, AL1A1, HDAC7, MENA, SNX1, Histone deacetylase class II, Olfactory receptor, Thioredoxin-like 2, Calmyrin, EPS8L1, COLEC12, HSP70, IGHG1, ARID4A, eEF2K, Follistatin, TXNRD1, SMYD3, Calmodulin, HSPA2, HSPA1A, LOH11CR2A, CKS2, Annexin XI, SET/NUP214 fusion protein, Cyclin B, IMD2, GMEB1, NTB-A, FAM63A, FOP, RIG-G, Fructosamine-3-kinase, PP2C, HSC70, TARDBP (TDP43), PRCC, TCOF1, RUVBL1, CapZIP, VILIP1
134	Esophageal Diseases	569	4.516E-02	2.019E-01	4.516E-02	2.019E-01	6	Cyclin B1, HSP70, DPP3, CKS2, Cyclin B, VILIP1
135	Renal Insufficiency	296	4.604E-02	2.043E-01	4.604E-02	2.043E-01	4	HSPA1B, HSP70, HSPA1A, HSC70
136	Connective Tissue Diseases	2088	4.960E-02	2.184E-01	4.960E-02	2.184E-01	15	PGMU, IGHG4, DARS, Histone deacetylase class II, Olfactory receptor, HSP70, IGHG1, Annexin VI, Calmodulin, RBP1, EYA1, NTB-A, PTPN2, RIG-G, PP2C

137	Dengue	80	5.132E-02	2.244E-01	5.132E-02	2.244E-01	2	HSP70, HSC70
138	Immunoproliferative Disorders	3793	5.202E-02	2.246E-01	5.202E-02	2.246E-01	24	IGHM, eEF1A, Reticulon 4, Cyclin B1, HSPA1B, Copine-4, Beta crystallin B2, MENA, CDO1, Histone deacetylase class II, PROSC, Olfactory receptor, HSP70, IGHG1, HSPA2, HSPA1A, eEF1A1, CKS2, SET/NUP214 fusion protein, Cyclin B, GMEB1, PP2C, HSC70, CapZIP
139	Jaw Abnormalities	81	5.247E-02	2.246E-01	5.247E-02	2.246E-01	2	EYA1, TCOF1
140	Smooth Muscle Tumor	12	5.317E-02	2.246E-01	5.317E-02	2.246E-01	1	RBP1
141	Heart Septal Defects, Atrial	12	5.317E-02	2.246E-01	5.317E-02	2.246E-01	1	PP2C
142	Skin Diseases, Infectious	187	5.358E-02	2.246E-01	5.358E-02	2.246E-01	3	Histone deacetylase class II, HSP70, HSC70
143	Atrial Fibrillation	82	5.363E-02	2.246E-01	5.363E-02	2.246E-01	2	HSP70, HSC70

144	Diabetic Angiopathies	189	5.498E-02	2.287E-01	5.498E-02	2.287E-01	3	HSPA1B, HSP70, HSPA1A
145	Respiration Disorders	191	5.639E-02	2.295E-01	5.639E-02	2.295E-01	3	HSPA1B, HSP70, HSPA1A
146	Pancreatitis	317	5.652E-02	2.295E-01	5.652E-02	2.295E-01	4	HSPA1B, Histone deacetylase class II, HSP70, HSPA1A
147	Gastrointestinal Neoplasms	1027 2	5.659E-02	2.295E-01	5.659E-02	2.295E-01	55	Annexin V, eEF1A, RBBP8 (CtIP), PGMU, IGHG4, RY1, Cyclin B1, HSPA1B, ECE1, Copine-4, AL1A1, Fbxo3, HDAC7, MENA, SNX1, Histone deacetylase class II, Olfactory receptor, Thioredoxin-like 2, Calmyrin, EPS8L1, COLEC12, HSP70, IGHG1, ARID4A, Annexin VI, PGM2, eEF2K, Follistatin, DPP3, TXNRD1, SMYD3, Calmodulin, HSPA2, HSPA1A, RBP1, LOH11CR2A, CKS2, Annexin XI, SET/NUP214 fusion protein, Cyclin B, IMD2, GMEB1, NTB-A, FAM63A, FOP, RIG-G, Fructosamine-3-kinase, PP2C, HSC70, TARDBP (TDP43), PRCC, TCOF1, RUVBL1, CapZIP, VILIP1
148	Maxillofacial Abnormalities	85	5.715E-02	2.295E-01	5.715E-02	2.295E-01	2	EYA1, TCOF1
149	Diphtheria	13	5.747E-02	2.295E-01	5.747E-02	2.295E-01	1	TXNRD1
150	Corynebacterium Infections	13	5.747E-	2.295E-	5.747E-	2.295E-	1	TXNRD1

			02	01	02	01		
151	Rectal Diseases	8766	5.789E-02	2.296E-01	5.789E-02	2.296E-01	48	Annexin V, eEF1A, RBBP8 (CtIP), PGMU, RY1, Cyclin B1, HSPA1B, ECE1, Copine-4, AL1A1, HDAC7, MENA, SNX1, Histone deacetylase class II, Olfactory receptor, Thioredoxin-like 2, Calmyrin, EPS8L1, COLEC12, HSP70, ARID4A, eEF2K, Follistatin, TXNRD1, SMYD3, Calmodulin, HSPA2, HSPA1A, LOH11CR2A, CKS2, Annexin XI, SET/NUP214 fusion protein, Cyclin B, IMD2, GMEB1, NTB-A, FAM63A, FOP, RIG-G, Fructosamine-3-kinase, PP2C, HSC70, TARDBP (TDP43), PRCC, TCOF1, RUVBL1, CapZIP, VILIP1
152	Melanoma, Cutaneous Malignant	763	5.880E-02	2.296E-01	5.880E-02	2.296E-01	7	Cyclin B1, HSPA1B, HSP70, HSPA2, HSPA1A, Cyclin B, HSC70
153	Inflammatory Bowel Diseases	923	5.880E-02	2.296E-01	5.880E-02	2.296E-01	8	HSPA1B, HSP70, CLEC2D, HSPA2, HSPA1A, PTPN2, FOP, PP2C
154	Hemorrhagic Fevers, Viral	87	5.954E-02	2.296E-01	5.954E-02	2.296E-01	2	HSP70, HSC70
155	Lymphoproliferative Disorders	3847	5.967E-02	2.296E-01	5.967E-02	2.296E-01	24	IGHM, eEF1A, Reticulon 4, Cyclin B1, HSPA1B, Copine-4, Beta crystallin B2, MENA, CDO1, Histone deacetylase class II, PROSC, Olfactory receptor, HSP70, IGHG1, HSPA2, HSPA1A, eEF1A1, CKS2, SET/NUP214 fusion protein, Cyclin B, GMEB1, PP2C, HSC70, CapZIP
156	Cystic Fibrosis	766	5.979E-02	2.296E-01	5.979E-02	2.296E-01	7	Annexin V, IGHM, IGHG4, HSPA1B, HSP70, IGHG1, HSPA1A
157	Infant, Newborn, Diseases	928	6.030	2.301	6.030	2.301	8	Annexin V, IGHM, IGHG4, HSPA1B, HSP70, IGHG1, Follistatin, HSPA1A

			E-02	E-01	E-02	E-01		
158	Pre-Eclampsia	325	6.082E-02	2.306E-01	6.082E-02	2.306E-01	4	Annexin V, HSPA1B, HSP70, HSPA1A
159	Noonan Syndrome	14	6.175E-02	2.312E-01	6.175E-02	2.312E-01	1	PP2C
160	Uterine Hemorrhage	14	6.175E-02	2.312E-01	6.175E-02	2.312E-01	1	Follistatin
161	Leukemia, T-Cell	200	6.296E-02	2.343E-01	6.296E-02	2.343E-01	3	Reticulon 4, SET/NUP214 fusion protein, PP2C
162	Cataract	92	6.567E-02	2.413E-01	6.567E-02	2.413E-01	2	Beta crystallin B2, EYA1
163	Wilms Tumor	92	6.567E-02	2.413E-01	6.567E-02	2.413E-01	2	SET, SET/NUP214 fusion protein
164	Brain Ischemia	205	6.67	2.42	6.67	2.42	3	HSPA1B, HSP70, HSPA1A

			6E-02	7E-01	6E-02	7E-01		
165	Glioma	5088	6.686E-02	2.427E-01	6.686E-02	2.427E-01	30	eEF1A, Reticulon 4, RBBP8 (CtIP), Cyclin B1, TIPIN, ECE1, Copine-4, Fbxo3, DARS, Histone deacetylase class II, PROSC, Olfactory receptor, EPS8L1, HSP70, Cyclophilin E, eEF2K, COASY, eEF1A1, CKS2, OR10G3, DOHH, SET/NUP214 fusion protein, Cyclin B, NTB-A, RIG-G, Fructosamine-3-kinase, PP2C, HSC70, CapZIP, VILIP1
166	Coronary Artery Disease	479	6.725E-02	2.427E-01	6.725E-02	2.427E-01	5	HSPA1B, ECE1, HSP70, Calmodulin, HSPA1A
167	Hypertension, Pregnancy-Induced	339	6.876E-02	2.456E-01	6.876E-02	2.456E-01	4	Annexin V, HSPA1B, HSP70, HSPA1A
168	Bone Diseases	793	6.924E-02	2.456E-01	6.924E-02	2.456E-01	7	HSPA1B, HSP70, HSPA2, HSPA1A, ICMT, EYA1, TCOF1
169	Medulloblastoma	2942	6.966E-02	2.456E-01	6.966E-02	2.456E-01	19	eEF1A, RBBP8 (CtIP), Cyclin B1, TIPIN, Fbxo3, DARS, Histone deacetylase class II, Olfactory receptor, EPS8L1, HSP70, eEF2K, COASY, DOHH, SET/NUP214 fusion protein, Cyclin B, NTB-A, RIG-G, Fructosamine-3-kinase, PP2C
170	Encephalomyelitis, Autoimmune, Experimental	16	7.026E-02	2.456E-01	7.026E-02	2.456E-01	1	NTB-A
17	Nervous System	16	7.0	2.4	7.0	2.4	1	NTB-A

1	Autoimmune Disease, Experimental		26E-02	56E-01	26E-02	56E-01		
172	Lens Diseases	96	7.072E-02	2.456E-01	7.072E-02	2.456E-01	2	Beta crystallin B2, EYA1
173	Neuroectodermal Tumors, Primitive	3333	7.095E-02	2.456E-01	7.095E-02	2.456E-01	21	eEF1A, RBBP8 (CtIP), Cyclin B1, TIPIN, Fbxo3, DARS, Histone deacetylase class II, Olfactory receptor, EPS8L1, HSP70, eEF2K, COASY, DOHH, SET/NUP214 fusion protein, Cyclin B, GMEB1, NTB-A, RIG-G, Fructosamine-3-kinase, PP2C, VILIP1
174	Neoplastic Syndromes, Hereditary	489	7.207E-02	2.467E-01	7.207E-02	2.467E-01	5	Cyclin B1, SET, SET/NUP214 fusion protein, Cyclin B, PP2C
175	Behavior and Behavior Mechanisms	489	7.207E-02	2.467E-01	7.207E-02	2.467E-01	5	eEF1A, Histone deacetylase class II, HSP70, eEF1A1, PQBP-1
176	Pathological Conditions, Signs and Symptoms	4332	7.397E-02	2.506E-01	7.397E-02	2.506E-01	26	Annexin V, eEF1A, Cyclin B1, HSPA1B, PSMD10 (Gankyrin), ECE1, MENA, Cytoglobin, Histone deacetylase class II, HSP70, SET, Follistatin, SMYD3, Calmodulin, HSPA2, HSPA1A, eEF1A1, SET/NUP214 fusion protein, Cyclin B, FOP, RIG-G, PP2C, HSC70, TARDBP (TDP43), PQBP-1, VILIP1
177	Arthritis, Experimental	17	7.448E-02	2.506E-01	7.448E-02	2.506E-01	1	HSP70
1	Multiple	1	7.	2.	7.	2.	1	PP2C

78	Endocrine Neoplasia	7	4.48E-02	5.06E-01	4.48E-02	5.06E-01		
179	Physiological Processes	217	7.629E-02	2.525E-01	7.629E-02	2.525E-01	3	HSPA1B, HSP70, HSPA1A
180	Growth	217	7.629E-02	2.525E-01	7.629E-02	2.525E-01	3	HSPA1B, HSP70, HSPA1A
181	Growth and Development	217	7.629E-02	2.525E-01	7.629E-02	2.525E-01	3	HSPA1B, HSP70, HSPA1A
182	Flavivirus Infections	102	7.853E-02	2.584E-01	7.853E-02	2.584E-01	2	HSP70, HSC70
183	Arbovirus Infections	103	7.985E-02	2.614E-01	7.985E-02	2.614E-01	2	HSP70, HSC70
184	Rheumatic Diseases	1509	8.041E-02	2.618E-01	8.041E-02	2.618E-01	11	IGHG4, DARS, Histone deacetylase class II, HSP70, IGHG1, Annexin VI, Calmodulin, EYA1, NTB-A, PTPN2, RIG-G

185	Dementia	1515	8.217E-02	2.627E-01	8.217E-02	2.627E-01	11	Reticulon 4, HSPA1B, ECE1, HSP70, eEF2K, TXNRD1, HSPA2, HSPA1A, Septin 3, TARDBP (TDP43), VILIP1
186	Orthomyxoviridae Infections	105	8.252E-02	2.627E-01	8.252E-02	2.627E-01	2	HSP70, HSC70
187	Influenza, Human	105	8.252E-02	2.627E-01	8.252E-02	2.627E-01	2	HSP70, HSC70
188	Lentigo	19	8.288E-02	2.627E-01	8.288E-02	2.627E-01	1	PP2C
189	Teratocarcinoma	19	8.288E-02	2.627E-01	8.288E-02	2.627E-01	1	Septin 3
190	Stomach Neoplasms	3793	8.393E-02	2.646E-01	8.393E-02	2.646E-01	23	Annexin V, PGMU, IGHG4, Cyclin B1, HSPA1B, AL1A1, Fbxo3, MENA, Olfactory receptor, HSP70, IGHG1, Annexin VI, PGM2, Calmodulin, HSPA2, HSPA1A, RBP1, CKS2, Cyclin B, IMD2, PP2C, HSC70, RUVBL1
191	Mandibular Diseases	20	8.704E-02	2.716E-01	8.704E-02	2.716E-01	1	EYA1

192	Melanosis	20	8.704E-02	2.716E-01	8.704E-02	2.716E-01	1	PP2C
193	Lymphoma, B-Cell	372	8.947E-02	2.777E-01	8.947E-02	2.777E-01	4	Cyclin B1, Histone deacetylase class II, Cyclin B, PP2C
194	Gastroenteritis	1014	8.999E-02	2.779E-01	8.999E-02	2.779E-01	8	HSPA1B, HSP70, CLEC2D, HSPA2, HSPA1A, PTPN2, FOP, PP2C
195	Thyroid Carcinoma, Follicular	111	9.070E-02	2.785E-01	9.070E-02	2.785E-01	2	HSP70, CKS2
196	Heart Septal Defects	21	9.119E-02	2.785E-01	9.119E-02	2.785E-01	1	PP2C
197	Arthritis, Rheumatoid	1367	9.159E-02	2.785E-01	9.159E-02	2.785E-01	10	IGHG4, DARS, Histone deacetylase class II, HSP70, IGHG1, Annexin VI, EYA1, NTB-A, PTPN2, RIG-G
198	Stomach Diseases	3840	9.337E-02	2.825E-01	9.337E-02	2.825E-01	23	Annexin V, PGMU, IGHG4, Cyclin B1, HSPA1B, AL1A1, Fbxo3, MENA, Olfactory receptor, HSP70, IGHG1, Annexin VI, PGM2, Calmodulin, HSPA2, HSPA1A, RBP1, CKS2, Cyclin B, IMD2, PP2C, HSC70, RUVBL1

199	Myocardial Ischemia	861	9.681E-02	2.914E-01	9.681E-02	2.914E-01	7	Annexin V, HSPA1B, ECE1, Olfactory receptor, HSP70, Calmodulin, HSPA1A
200	Bone Diseases, Developmental	243	9.885E-02	2.961E-01	9.885E-02	2.961E-01	3	ICMT, EYA1, TCOF1

**Table 10S: Enrichment analysis report.** Enrichment by Diseases (by Biomarkers) (SVZp vs SVZn)								
Enrichment by Diseases (by Biomarkers)					SVZp VS SVZn Limma List_genelist			
**#**	**Diseases**	**Total**	**pValue**	**Min FDR**	**p-value**	**FDR**	**In Data**	**Network Objects from Active Data**
1	Mycoplasmatales Infections	7	1.674 E-05	1.708 E-03	1.674 E-05	1.708 E-03	2	eEF1A1, eEF1A
2	Mycoplasma Infections	7	1.674 E-05	1.708 E-03	1.674 E-05	1.708 E-03	2	eEF1A1, eEF1A
3	Neuroblastoma	520	8.727 E-05	4.912 E-03	8.727 E-05	4.912 E-03	5	CAS-L, MJD (ataxin-3), DR1, GABP alpha, GMEB1
4	Neuroectodermal Tumors, Primitive, Peripheral	531	9.632 E-05	4.912 E-03	9.632 E-05	4.912 E-03	5	CAS-L, MJD (ataxin-3), DR1, GABP alpha, GMEB1

Supplementary Figures and Legends:

Autoantibody Profiling of Glioma Serum Samples to Identify Biomarkers Using Human Proteome Arrays

Parvez Syed[1], Shabarni Gupta[1], Saket Choudhary[2], Narendra Goud Pandala[1], Apurva Atak[1], Annie Richharia[1], Manubhai KP[1], Heng Zhu[3], Sridhar Epari[4], Santosh B. Noronha[2], Aliasgar Moiyadi[5], Sanjeeva Srivastava[1*]

[1]Department of Biosciences and Bioengineering, Indian Institute of Technology Bombay, Powai, Mumbai 400076, India

[2]Department of Chemical Engineering, Indian Institute of Technology Bombay, Powai, Mumbai 400076, India

[3]Department of Pharmacology and Molecular Sciences/High-Throughput Biology Center, Johns Hopkins University School of Medicine, Baltimore, MD, USA

[4]Department of Pathology, Tata Memorial Centre, Mumbai400 012, India

[5]Department of Neurosurgery, Tata Memorial Centre, Mumbai 400 012, India

[*]Correspondence: Dr. Sanjeeva Srivastava, Department of Biosciences and Bioengineering, IIT Bombay, Mumbai 400 076, India: E-mail: sanjeeva@iitb.ac.in
Phone: +91-22-2576-7779, Fax: +91-22-2572-3480

Supplementary Figure Legends:

Supplementary Figure 1: Enriched Networks emerging from TAAs in each grade.

Supplementary Fig. 1 represents the most enriched networks emerging from our analysis which associates one or more classifier proteins. The classifiers in these networks have been highlighted in red. The protein molecules outlined in pink are proteins attributed in Neurological disease, Immunological disease or Cancers. Panel A represents the network “Cell death and survival, inflammatory response, cell to cell signaling and interaction” while Panel B denotes network “Developmental disorder, hereditary disorder, neurological disease”. These two panels are the top two networks emerging from the Control Vs Grade II enrichment analysis, Panel C signifies the network “cell death and survival, cancer, cellular assembly and organization” and Panel D represents the network “cancer, gastrointestinal diseases, cellular growth and proliferation” Panel C and D are the top two networks implicated in the Control Vs Grade III analysis. Panel E denotes the network “Cancer, cellular movement, inflammatory response” while Panel F signifies network “cell morphology, cellular movement, cell to cell signalling and interactions”. Panel E and F are the top networks emerging from Control Vs Grade IV analysis. These images were generated from QIAGEN’s Ingenuity®Pathway Analysis (IPA®, QIAGEN Redwood City, www.qiagen.com/ingenuity).

Supplementary Figure 2: Signal intensities for significant proteins in each grade.

The signal intensities of protein spots in various chips have been generated for the visualization of the expression pattern for each classifier protein in case of Grade II, Grade III and Grade IV proteins as against healthy controls and IDH1p against WT. These images of the spots have also been documented as qualitative controls so as to assure that the intensity readings from each spot are not due to any artifacts.

Supplementary Figure 1: Enriched Networks emerging from TAAs in each grade.

a.

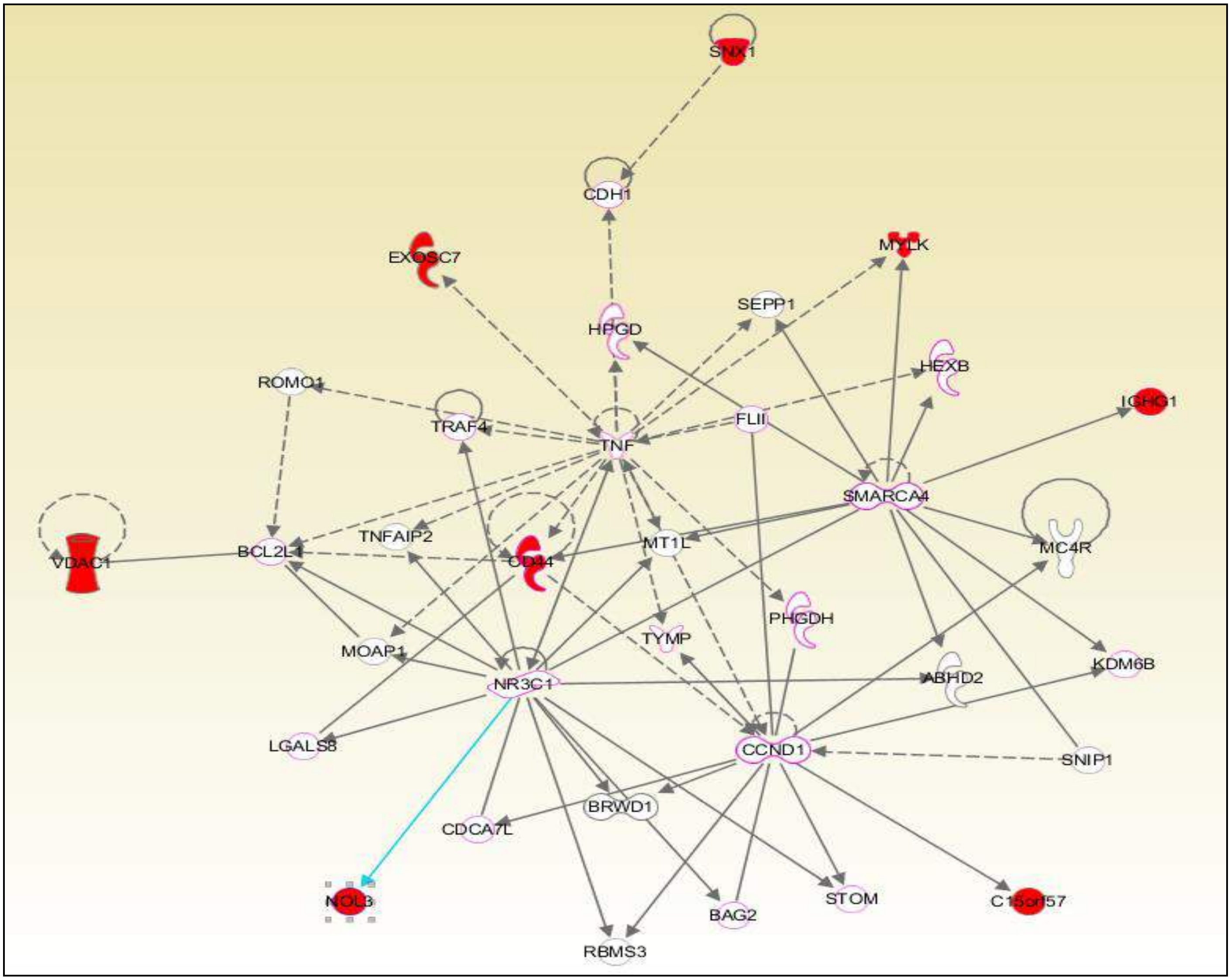

b.

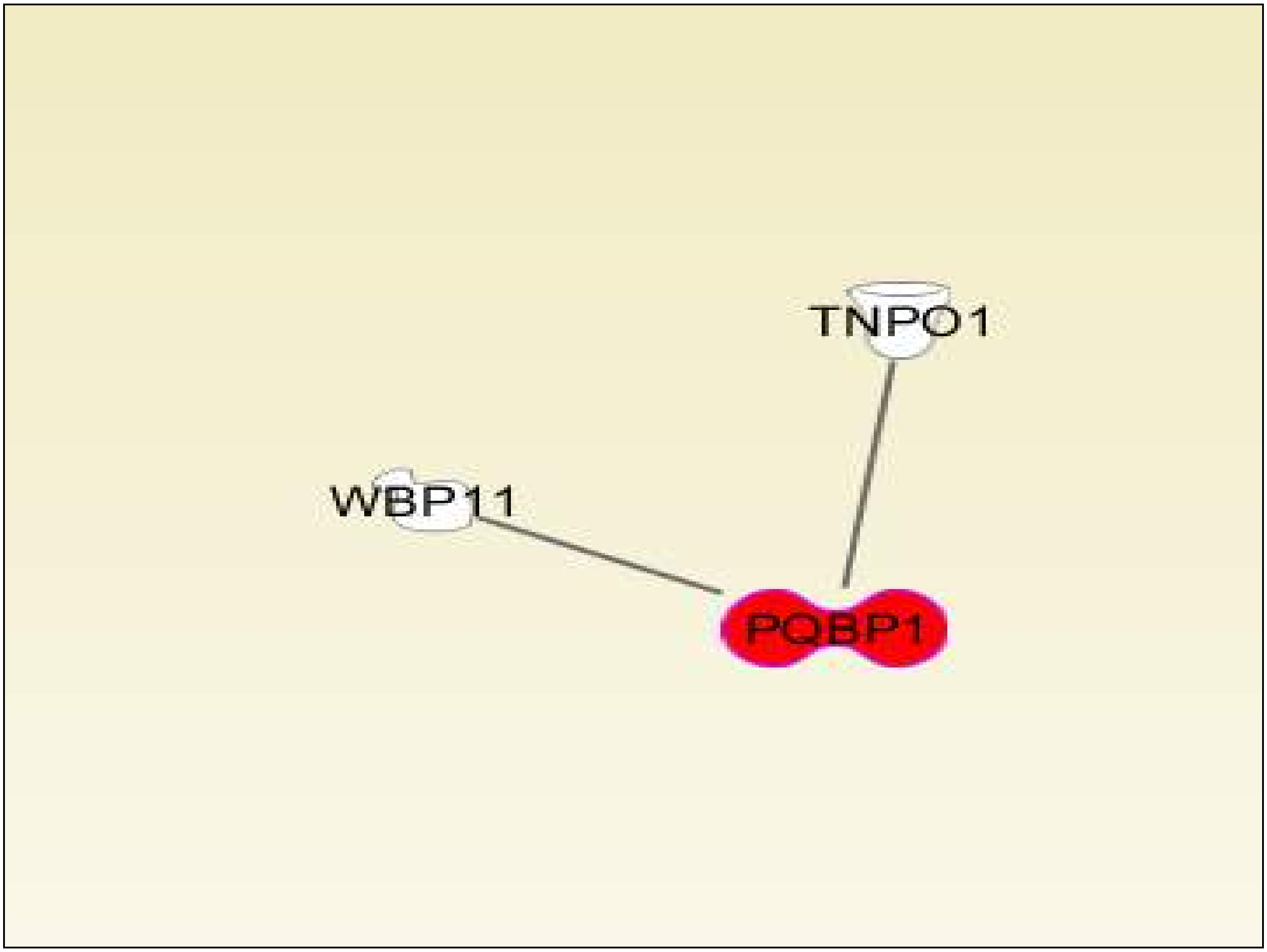

c.

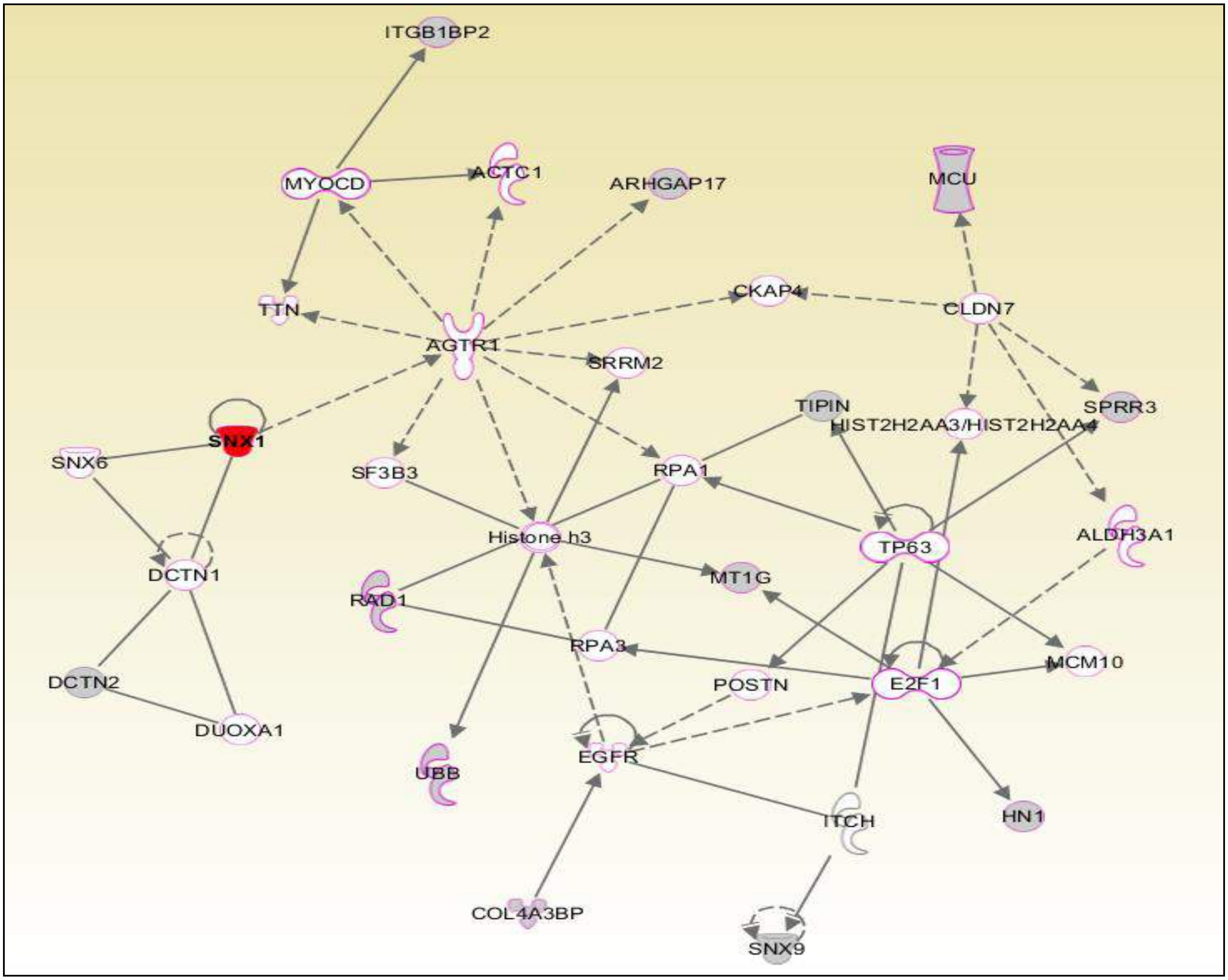
ITGB1BP2
MYOCD
ACTC1
ARHGAP17
MCU
CKAP4
CLDN7
TTN
AGTR1
SRRM2
TIPIN
SPRR3
HIST2H2AA3/HIST2H2AA4
SNX1
SNX6
SF3B3
RPA1
ALDH3A1
Histone h3
TP63
DCTN1
MT1G
RAD1
RPA3
MCM10
DCTN2
POSTN
E2F1
DUOXA1
EGFR
UBB
ITCH
HN1
COL4A3BP
SNX9

d.

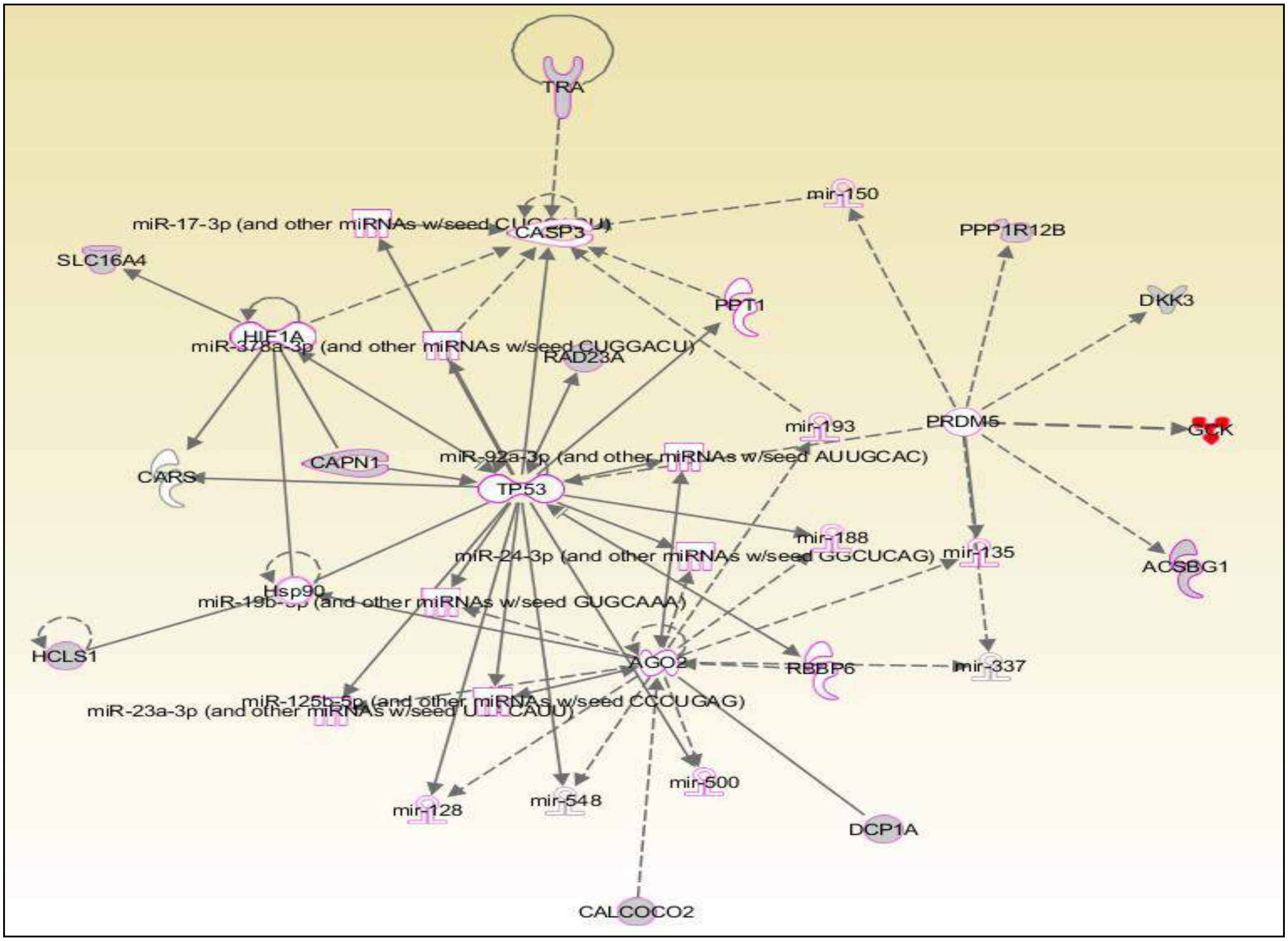
TRA
mir-150
PPP1R12B
CASP3
SLC16A4
DKK3
PPT1
HIF1A
miR-378a-3p (and other miRNAs w/seed CUGGACU)
RAD23A
mir-193
PRDM5
GCK
miR-92a-3p (and other miRNAs w/seed AUUGCAC)
CAPN1
CARS
TP53
mir-188
miR-24-3p (and other miRNAs w/seed GGCUCAG)
mir-135
ACSBG1
Hsp90
miR-19b-3p (and other miRNAs w/seed GUGCAAA)
HCLS1
AGO2
RBBP6
mir-337
miR-125b-5p (and other miRNAs w/seed CCCUGAG)
mir-500
mir-548
mir-128
DCP1A
CALCOCO2

e.

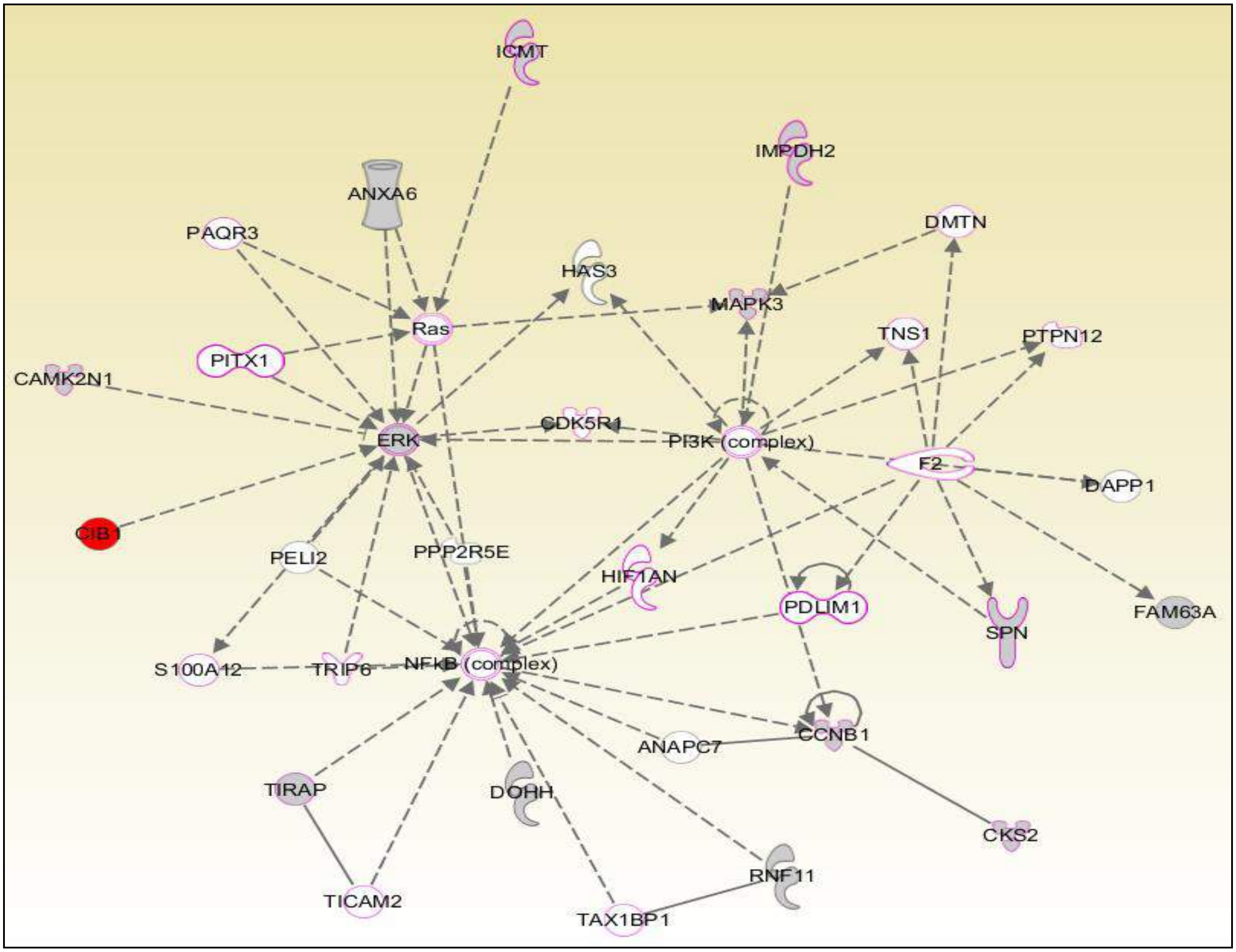

ICMT
IMPDH2
ANXA6
DMTN
PAQR3
HAS3
MAPK3
Ras
TNS1
PTPN12
PITX1
CAMK2N1
CDK5R1
ERK
PI3K (complex)
F2
DAPP1
CIB1
PELI2
PPP2R5E
HIF1AN
PDLIM1
SPN
FAM63A
S100A12
TRIP6
NFkB (complex)
ANAPC7
CCNB1
TIRAP
DOHH
CKS2
RNF11
TICAM2
TAX1BP1

f.

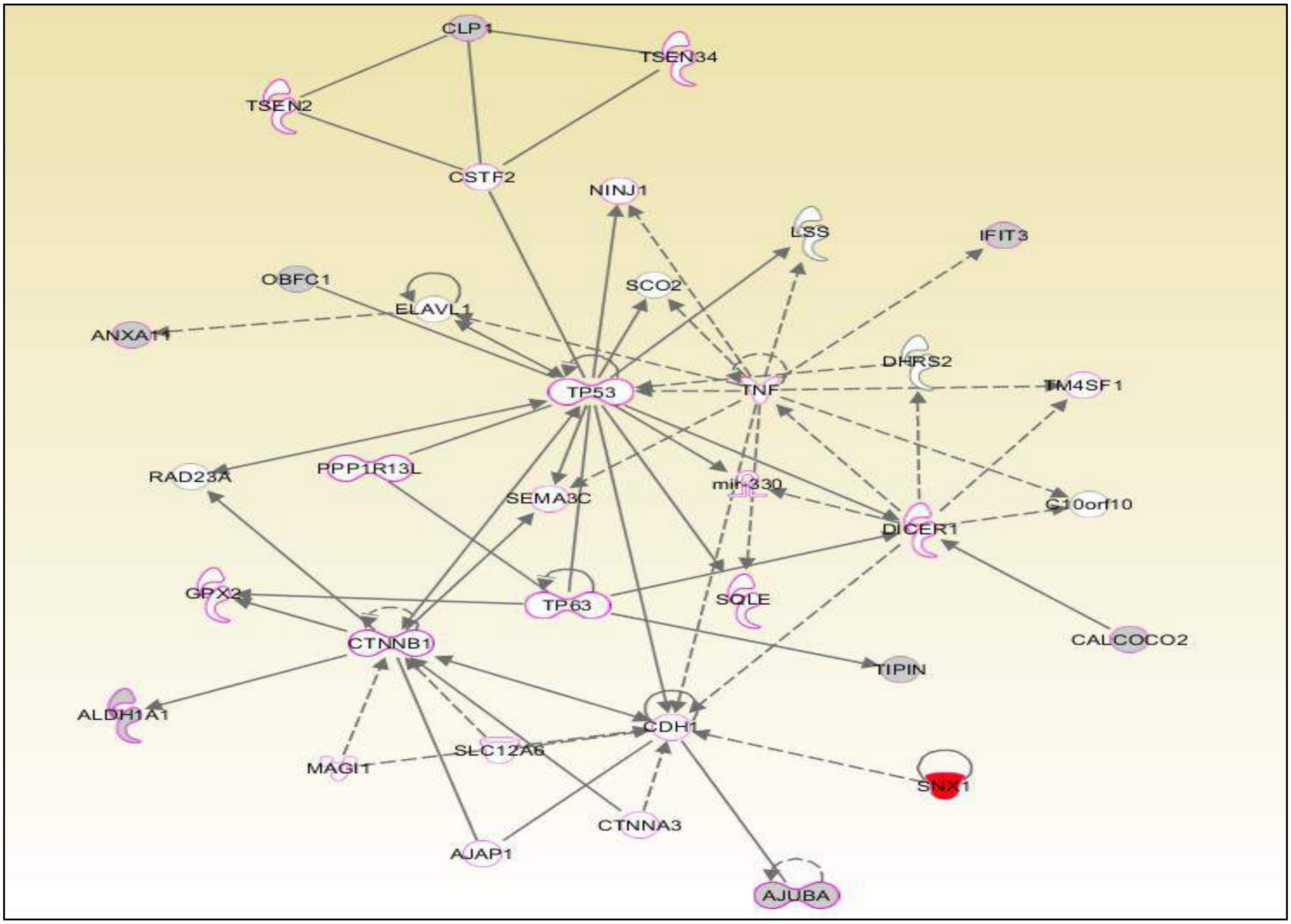

CLP1
TSEN34
TSEN2
CSTF2
NINJ1
LSS
IFIT3
OBFC1
SCO2
ELAVL1
ANXA11
DHRS2
TP53
TNF
TM4SF1
RAD23A
PPP1R13L
SEMA3C
mir-330
C10orf10
DICER1
GPX2
TP63
SQLE
CTNNB1
CALCOCO2
TIPIN
ALDH1A1
CDH1
SLC12A6
MAGI1
SNX1
CTNNA3
AJAP1
AJUBA

Supplementary Figure 2

List of classifier proteins

Control VS Grade II		Control VS Grade III		Control VS Grade IV		SVZp VS SVZn	
**Accession No.**	**Gene Symbol**	**Accession No.**	**Gene Symbol**	**Accession No.**	**Gene Symbol**	**Accession No.**	**Gene Symbol**
NM_003099.3	SNX1	NM_003099.3	SNX1	NM_003099.3	SNX1	NM_001938.2	DR1
BC018747.1	IGHG1	NM_020532.4	RTN4	BC018747.1	IGHG1	NM_018290.2	PGM2
NM_052849.2	CCDC32	NM_016246.2	HSD17B14	BC009561.1	C11orf74	NM_032448.1	FAM120B
ENST00000361290	MYLK	BC001890.1	GCK	BC068569.1	ZHX3	NM_006403.2	NEDD9
NM_003374.1	VDAC1	BC017723.1	MAGEA4	BC121798	EYA1	NM_024482.1	GMEB1
NM_144495.2	PQBP1	NM_173608.1	C14orf80	NM_144495.2	PQBP1	BC000267.1	GPBP1
BC004372.1	CD44	NM_198204.1	MLX	NM_021810.3	CDH26	NM_152740.2	HIBADH
BC121798	EYA1	NM_138794.1	LYPLAL1	BC013186.1	RCSD1	BCO17810.1	TMOD4
NM_015004.2	EXOSC7	NM_015926.3	TEX264	BC000846.2	CIB1	BC094687.1	EEF1A1
Nol3	NOL3	BC001241.1	ARHGAP17	BC037876.1	C17orf57	XM_379060.1	LOC339685

Control Vs Grade II Glioma

NM_003099.3 SNX1

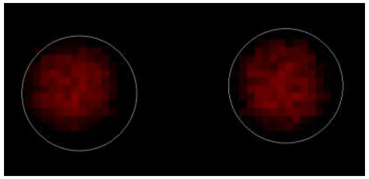
Control_H-02_2000153953

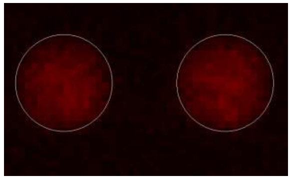
Control_H-03_2000153943

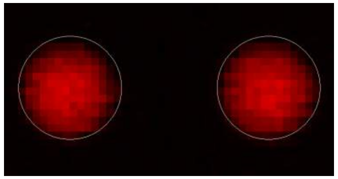
Control_H-19_2000154008

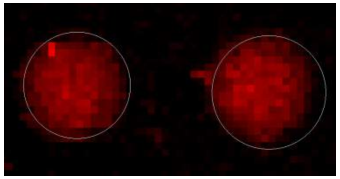
Control_H-23_2000154009

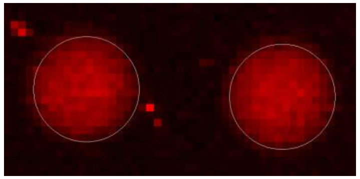
Control_H-25_2000153952

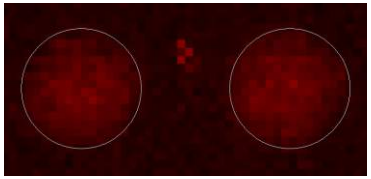
Control_H-35_2000153942

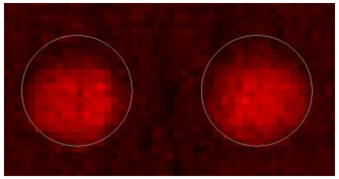
Control_H-41_2000154026

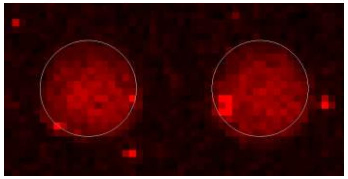
Control_H-58_2000153935

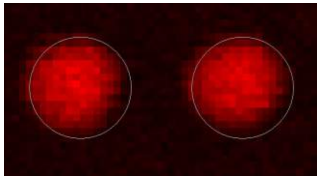
Control_H-59_2000154016

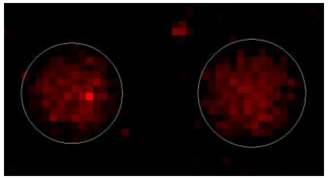
Control_HC 25_2000153932

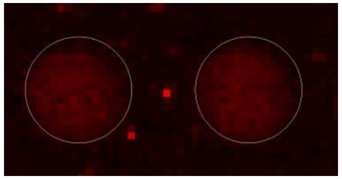
Control_HV-56_2000155735

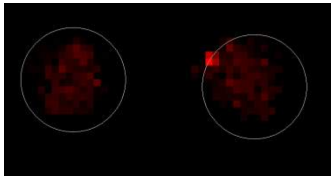
Control_HV-59_2000155740

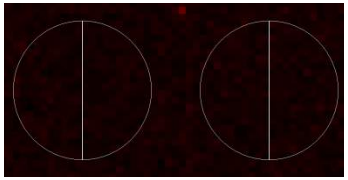
Control_HV-64_2000144456

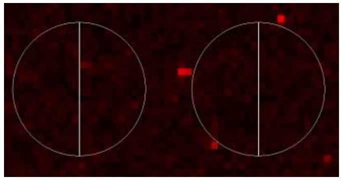
Control_HV-70_2000144457

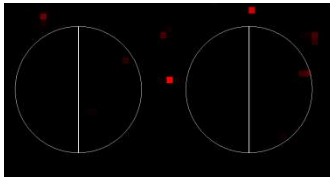
Control_HV-71_2000144458

Control

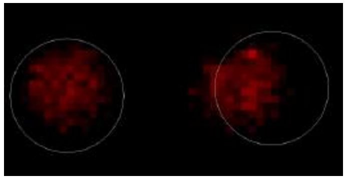

Grade II_CF 8551_2000153940

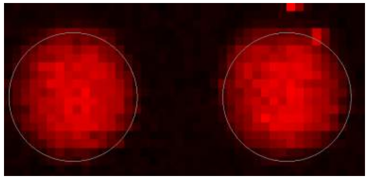

Grade II_CF 10729_2000154012

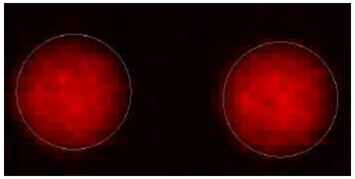

Grade II_CF 20468_2000153947

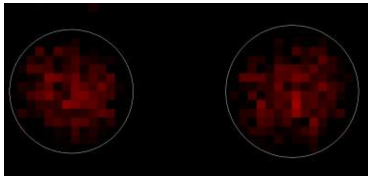

Grade II_CF 28254_2000153938

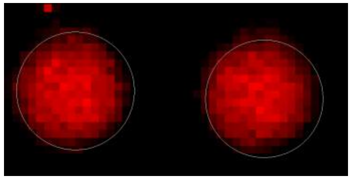

Grade II_CF 29538_2000154020

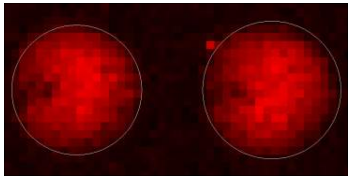

Grade II_CK 40_2000144445

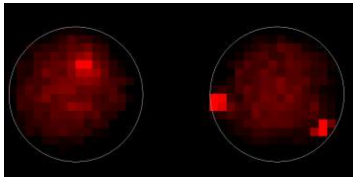

Grade II_CH 12323_2000144444

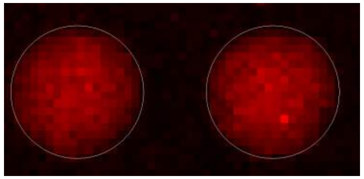

Grade II_CH 13717_2000144447

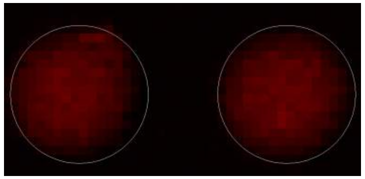

Grade II_CH 27095_2000154014

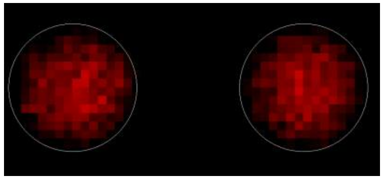

Grade II_CH 32225_2000154033

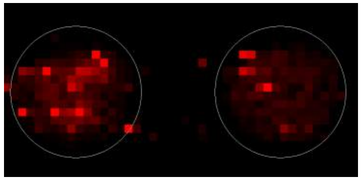

Grade II_CJ 863_20001543970

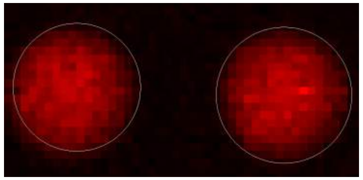

Grade II_CJ 4825_2000143981

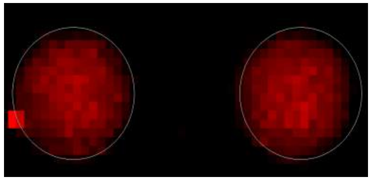

Grade II_CJ 7132_2000154028

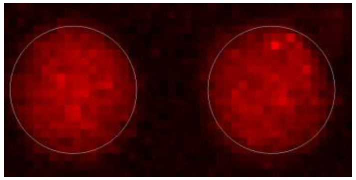

Grade II_CJ 10242_2000144449_2

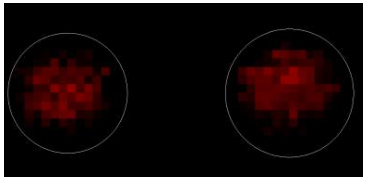

Grade II_CJ 16084_2000153931

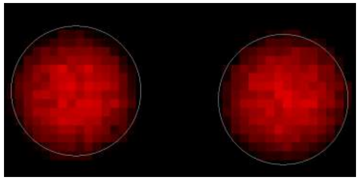

Grade II_CJ 17734_2000154015

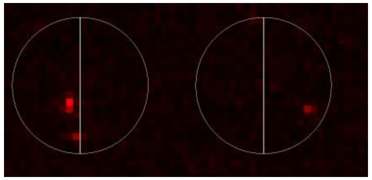

Grade II_CJ 33350_2000144452

Grade II

BC018747.1 IGHG1

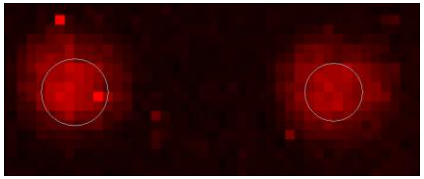

Control_H-02_2000153953

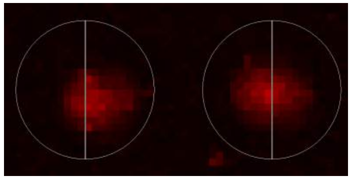

Control_H-03_2000153943

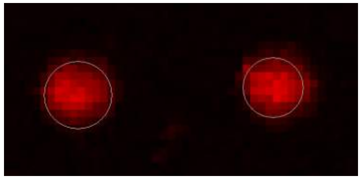

Control_H-19_2000154008

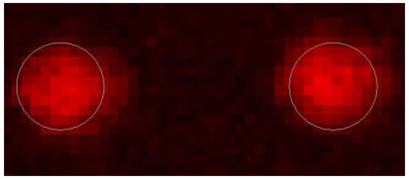

Control_H-23_2000154009

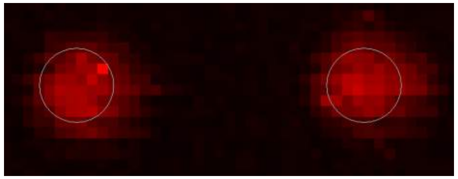

Control_H-25_2000153952

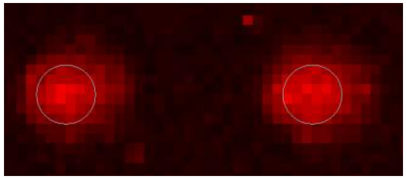

Control_H-35_2000153942

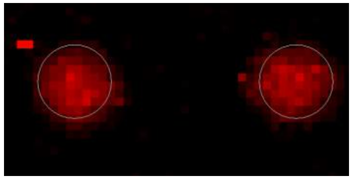

Control_H-41_2000154026

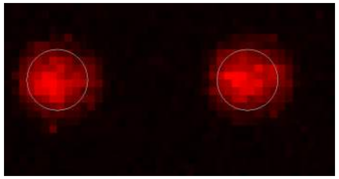

Control_H-58_2000153935

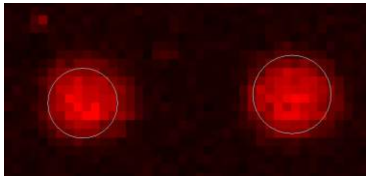

Control_H-59_2000154016

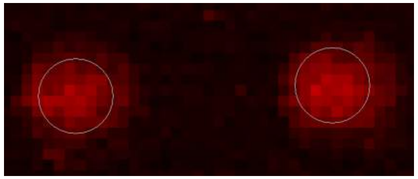

Control_HC 25_2000153932

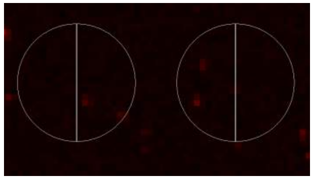

Control_HV-56_2000155735

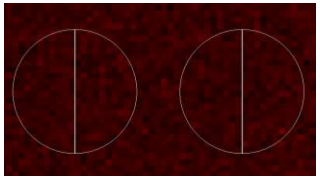

Control_HV-59_2000155740

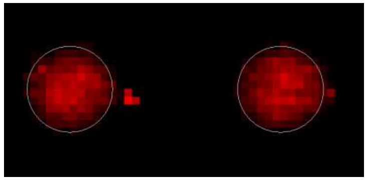

Control_HV-64_2000144456

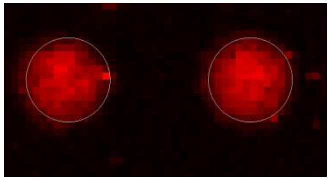

Control_HV-70_2000144457

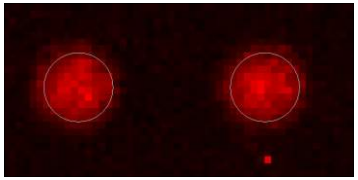

Control_HV-71_2000144458

Control

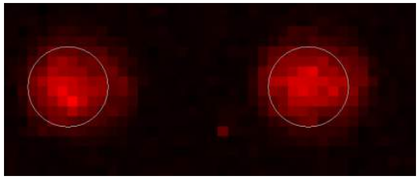

Grade II_CF 8551_2000153940

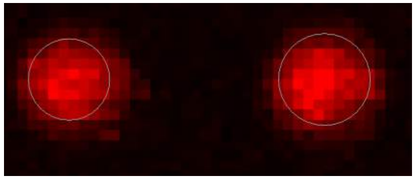

Grade II_CF 10729_2000154012

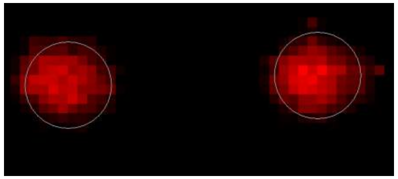

Grade II_CF 20468_2000153947

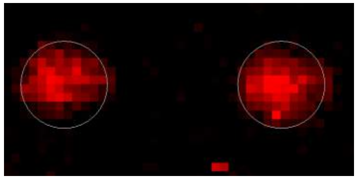

Grade II_CF 28254_2000153938

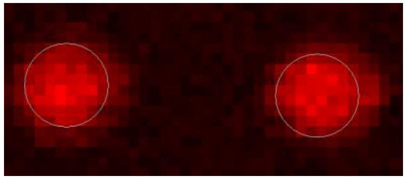

Grade II_CF 29538_2000154020

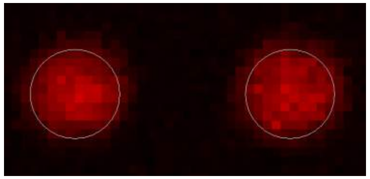

Grade II_CK 40_2000144445

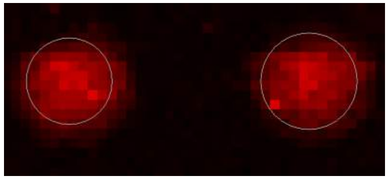

Grade II_CH 12323_2000144444

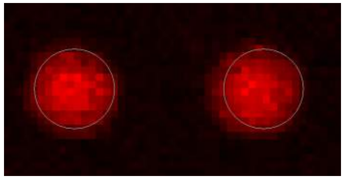

Grade II_CH 13717_2000144447

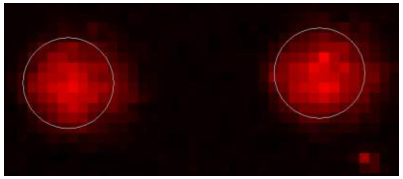

Grade II_CH 27095_2000154014

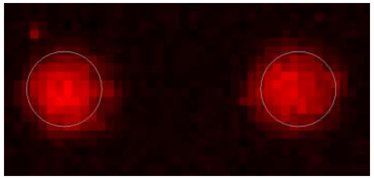

Grade II_CH 32225_2000154033

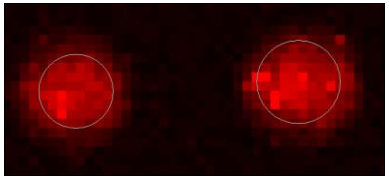

Grade II_CJ 863_20001543970

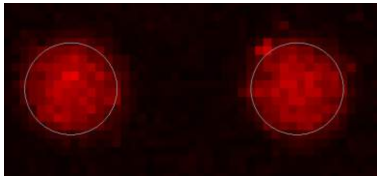

Grade II_CJ 4825_2000143981

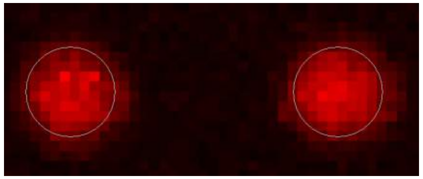

Grade II_CJ 7132_2000154028

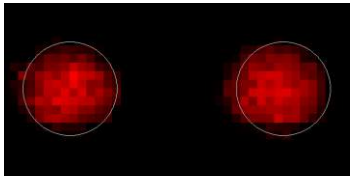

Grade II_CJ 10242_2000144449_2

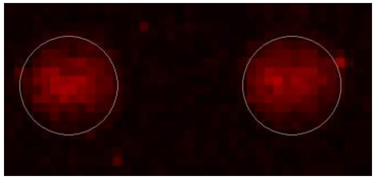

Grade II_CJ 16084_2000153931

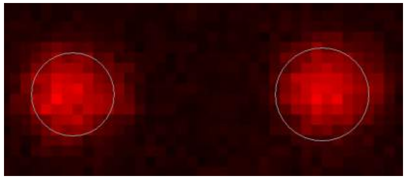

Grade II_CJ 17734_2000154015

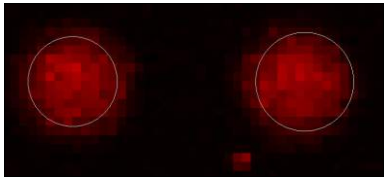

Grade II_CJ 33350_2000144452

Grade II

NM_052849.2 CCDC32

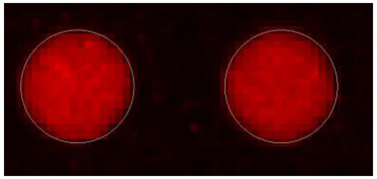
Control_H-02_2000153953

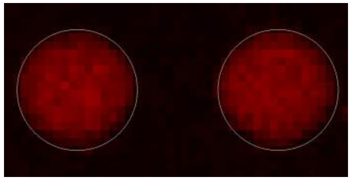
Control_H-03_2000153943

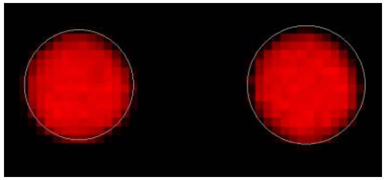
Control_H-19_2000154008

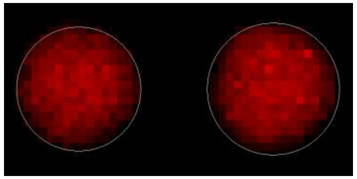
Control_HV-59_2000155740

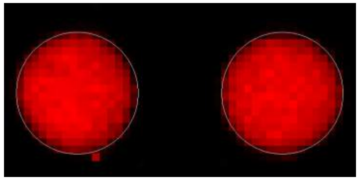
Control_H-23_2000154009

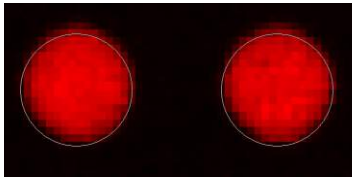
Control_H-25_2000153952

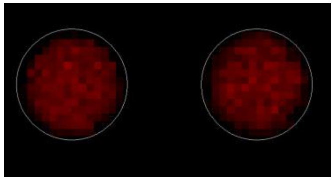
Control_H-35_2000153942

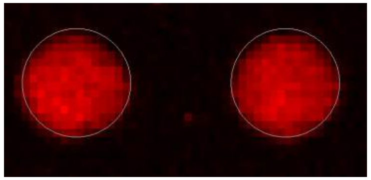
Control_HV-64_2000144456

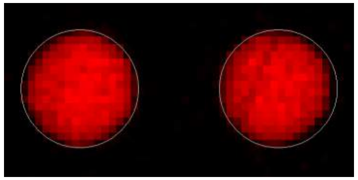
Control_H-41_2000154026

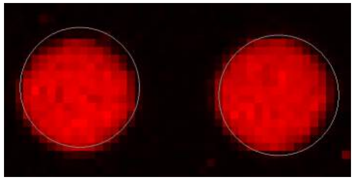
Control_H-58_2000153935

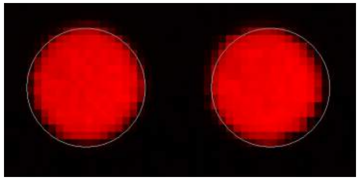
Control_H-59_2000154016

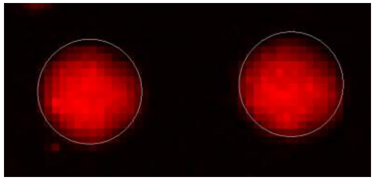
Control_HV-70_2000144457

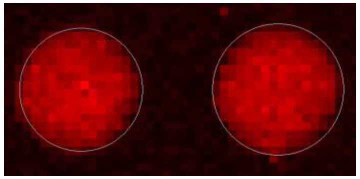
Control_HC 25_2000153932

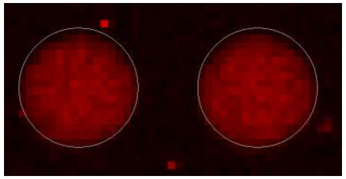
Control_HV-56_2000155735

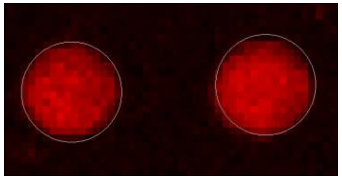
Control_HV-71_2000144458

Control

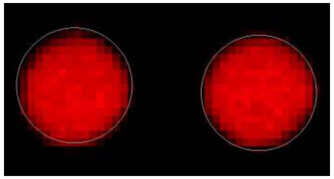
Grade II_CF 8551_2000153940

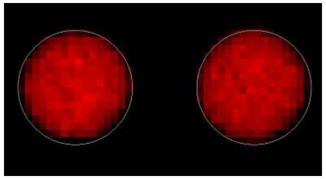
Grade II_CF 10729_2000154012

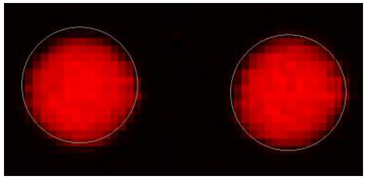
Grade II_CF 20468_2000153947

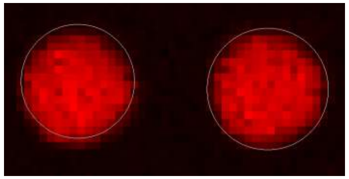
Grade II_CF 28254_2000153938

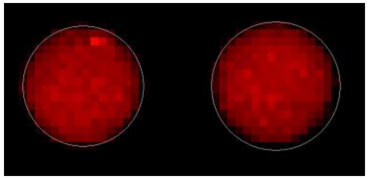
Grade II_CF 29538_2000154020

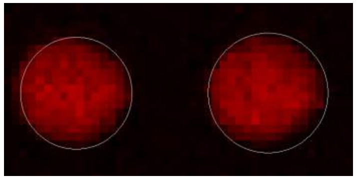
Grade II_CK 40_2000144445

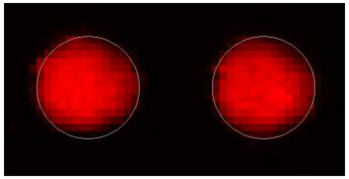
Grade II_CH 12323_2000144444

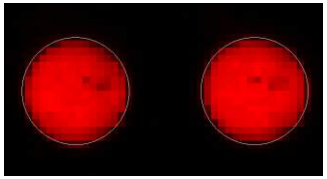
Grade II_CH 13717_2000144447

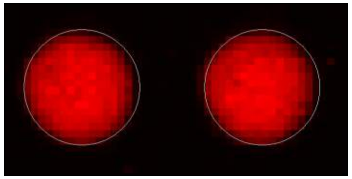
Grade II_CH 27095_2000154014

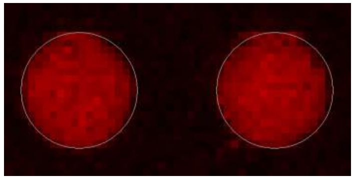
Grade II_CH 32225_2000154033

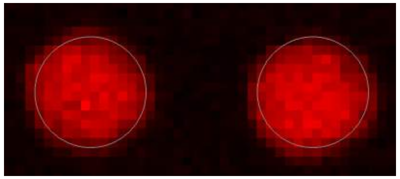
Grade II_CJ 863_20001543970

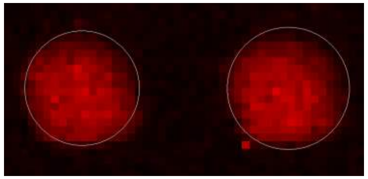
Grade II_CJ 4825_2000143981

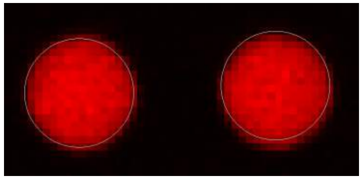
Grade II_CJ 7132_2000154028

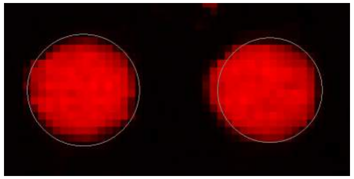
Grade II_CJ 10242_2000144449_2

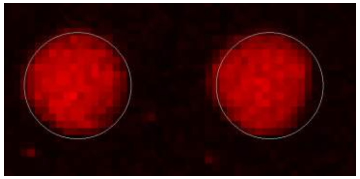
Grade II_CJ 16084_2000153931

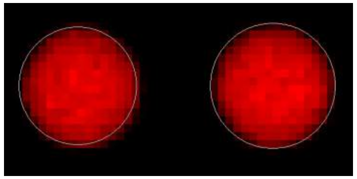
Grade II_CJ 17734_2000154015

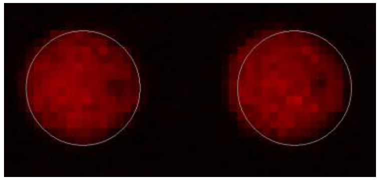
Grade II_CJ 33350_2000144452

Grade II

ENST00000361290 MYLK

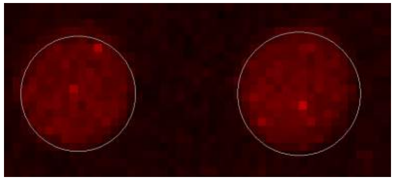

Control_H-02_2000153953

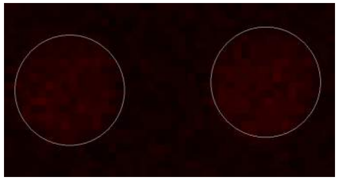

Control_H-03_2000153943

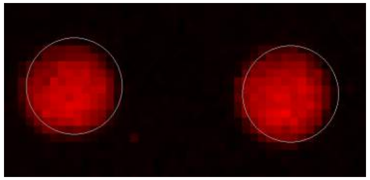

Control_H-19_2000154008

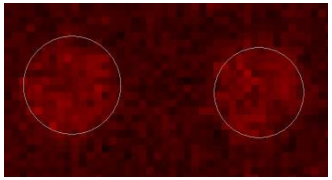

Control_HV-59_2000155740

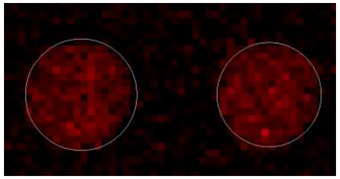

Control_H-23_2000154009

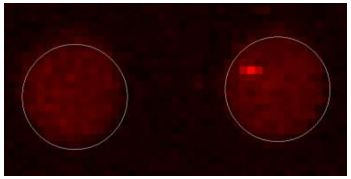

Control_H-25_2000153952

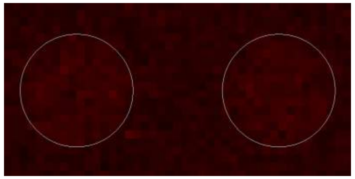

Control_H-35_2000153942

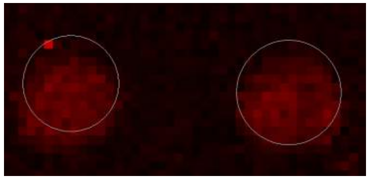

Control_HV-64_2000144456

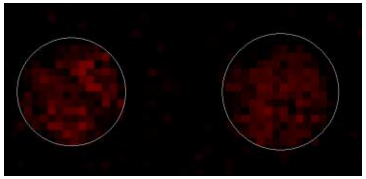

Control_H-41_2000154026

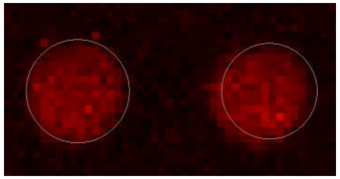

Control_H-58_2000153935

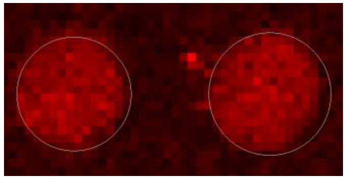

Control_H-59_2000154016

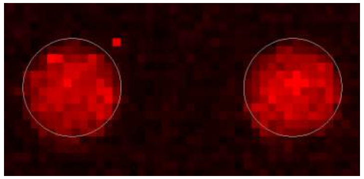

Control_HV-70_2000144457

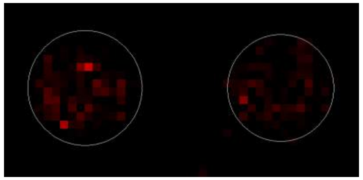

Control_HC 25_2000153932

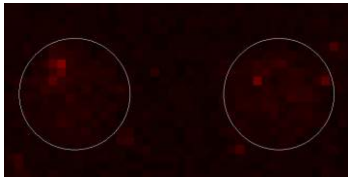

Control_HV-56_2000155735

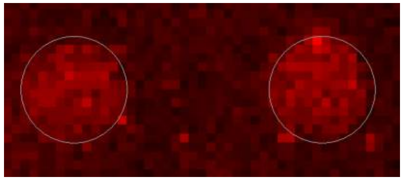

Control_HV-71_2000144458

Control

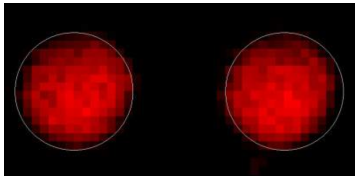
Grade II_CF 8551_2000153940

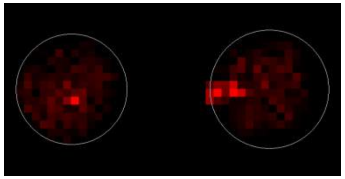
Grade II_CF 10729_2000154012

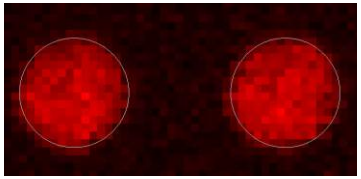
Grade II_CF 20468_2000153947

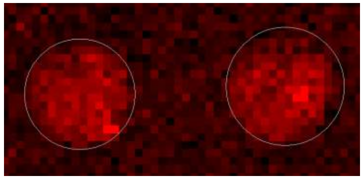
Grade II_CF 28254_2000153938

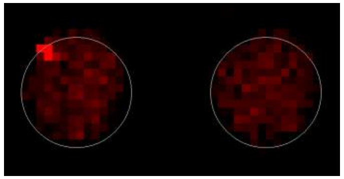
Grade II_CF 29538_2000154020

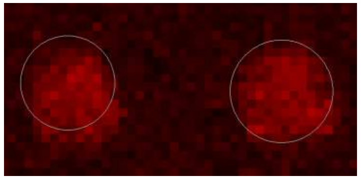
Grade II_CK 40_2000144445

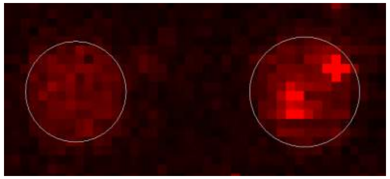
Grade II_CH 12323_2000144444

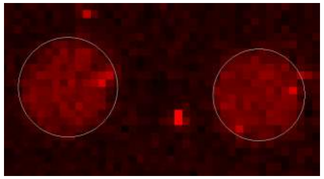
Grade II_CH 13717_2000144447

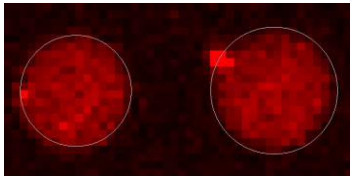
Grade II_CH 27095_2000154014

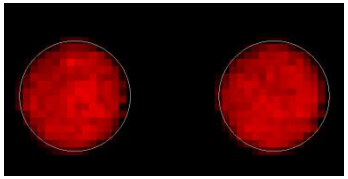
Grade II_CH 32225_2000154033

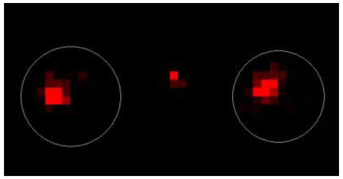
Grade II_CJ 863_20001543970

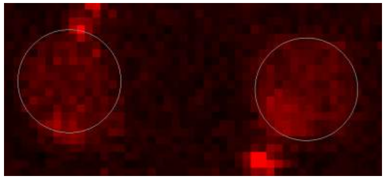
Grade II_CJ 4825_2000143981

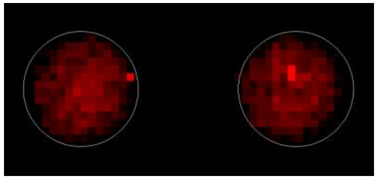
Grade II_CJ 7132_2000154028

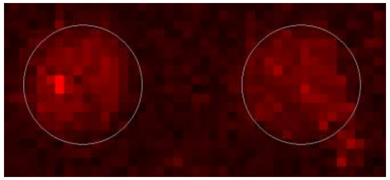
Grade II_CJ 10242_2000144449_2

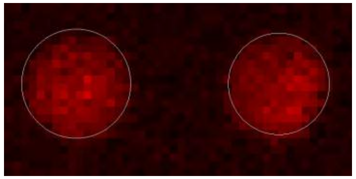
Grade II_CJ 16084_2000153931

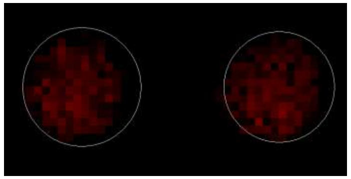
Grade II_CJ 17734_2000154015

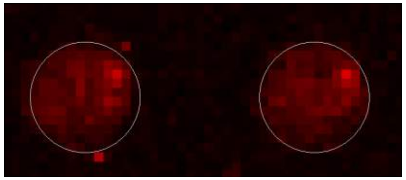
Grade II_CJ 33350_2000144452

Grade II

NM_003374.1 VDAC1

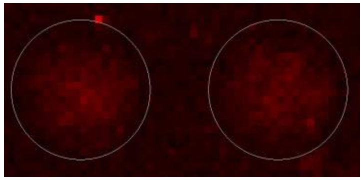
Control_H-02_2000153953

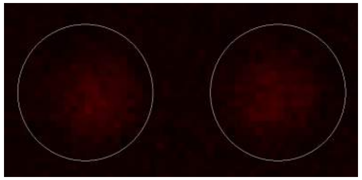
Control_H-03_2000153943

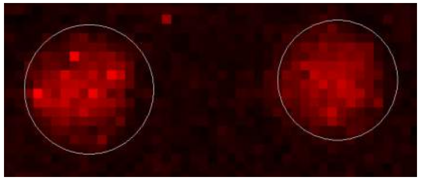
Control_H-19_2000154008

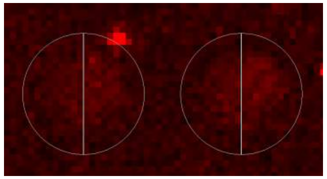
Control_HV-59_2000155740

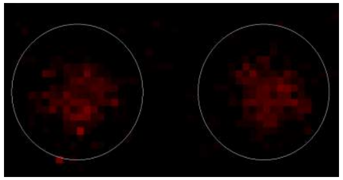
Control_H-23_2000154009

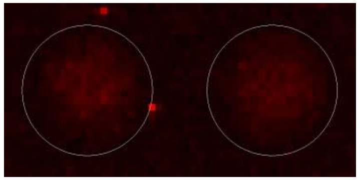
Control_H-25_2000153952

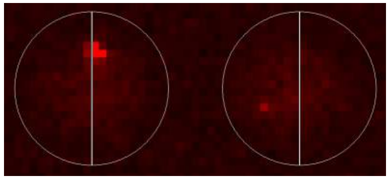
Control_H-35_2000153942

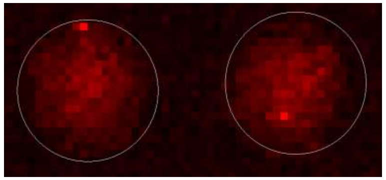
Control_HV-64_2000144456

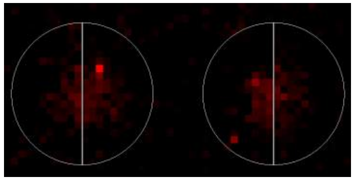
Control_H-41_2000154026

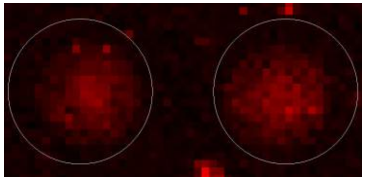
Control_H-58_2000153935

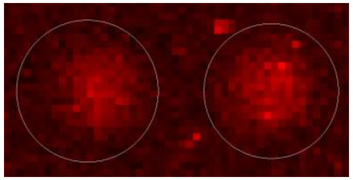
Control_H-59_2000154016

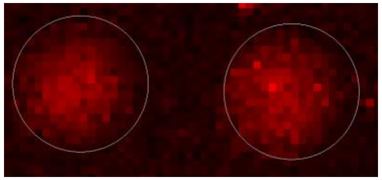
Control_HV-70_2000144457

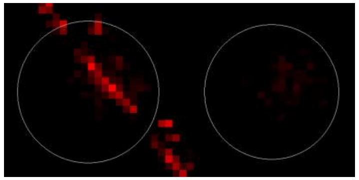
Control_HC 25_2000153932

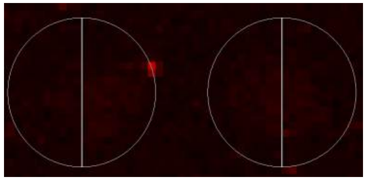
Control_HV-56_2000155735

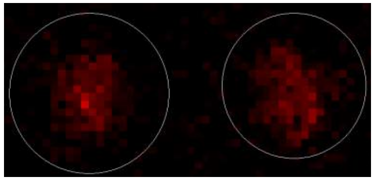
Control_HV-71_2000144458

Control

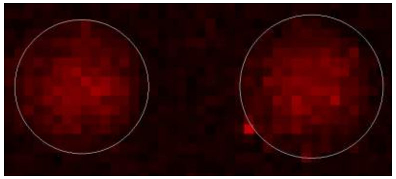

Grade II_CF 8551_2000153940

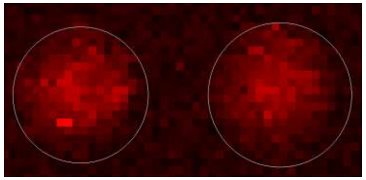

Grade II_CF 10729_2000154012

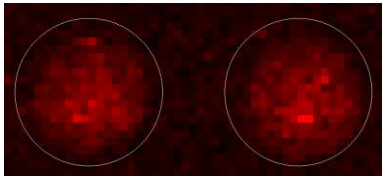

Grade II_CF 20468_2000153947

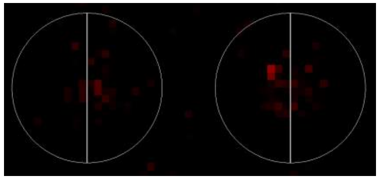

Grade II_CF 28254_2000153938

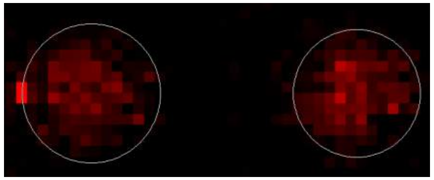

Grade II_CF 29538_2000154020

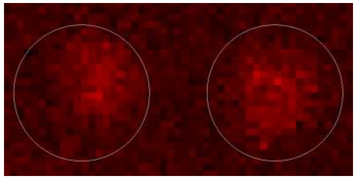

Grade II_CK 40_2000144445

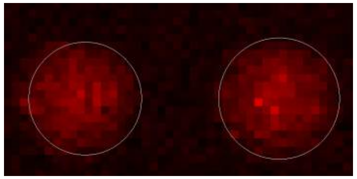

Grade II_CH 12323_2000144444

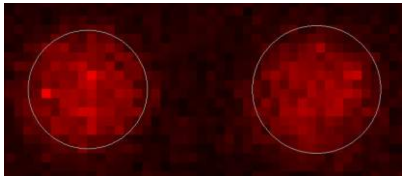

Grade II_CH 13717_2000144447

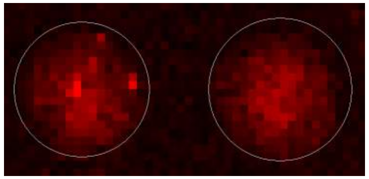

Grade II_CH 27095_2000154014

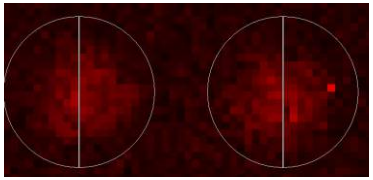

Grade II_CH 32225_2000154033

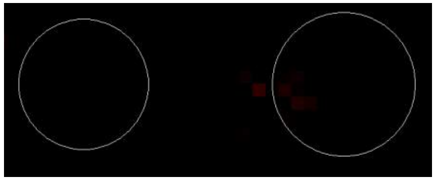

Grade II_CJ 863_20001543970

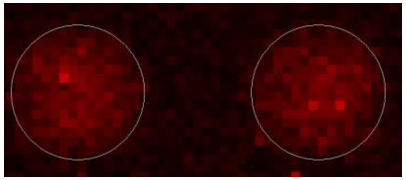

Grade II_CJ 4825_2000143981

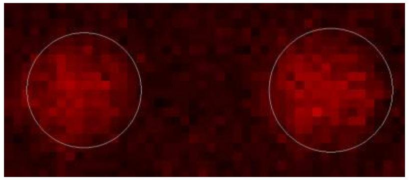

Grade II_CJ 7132_2000154028

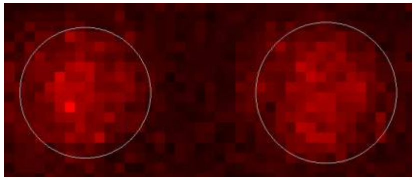

Grade II_CJ 10242_2000144449_2

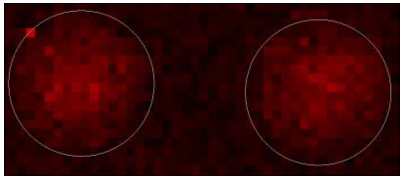

Grade II_CJ 16084_2000153931

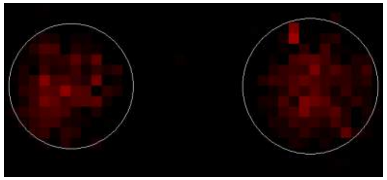

Grade II_CJ 17734_2000154015

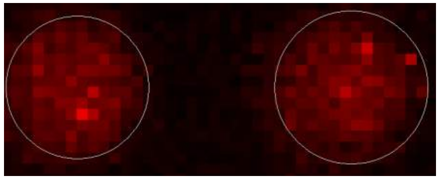

Grade II_CJ 33350_2000144452

Grade II

NM_144495.2 PQBP1

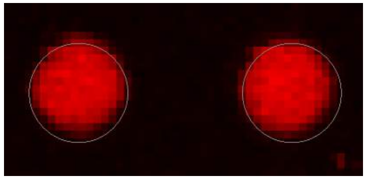
Control_H-02_2000153953

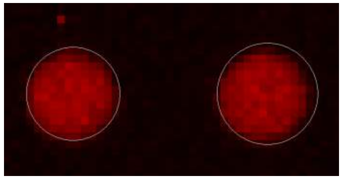
Control_H-03_2000153943

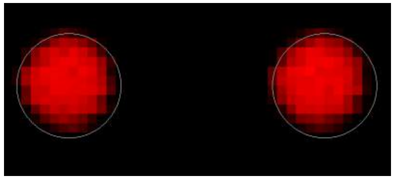
Control_H-19_2000154008

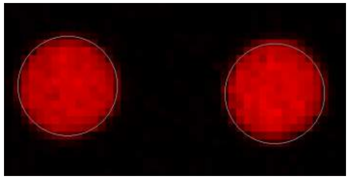
Control_H-23_2000154009

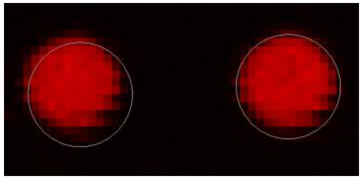
Control_H-25_2000153952

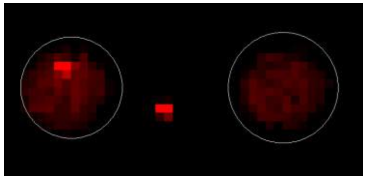
Control_H-35_2000153942

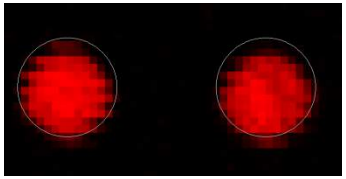
Control_H-41_2000154026

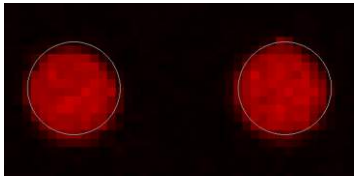
Control_H-58_2000153935

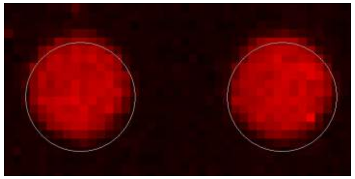
Control_H-59_2000154016

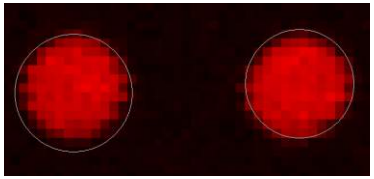
Control_HC 25_2000153932

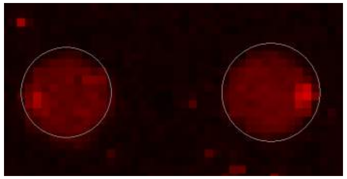
Control_HV-56_2000155735

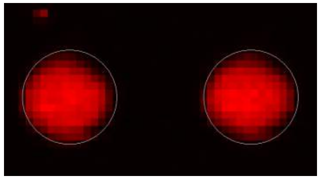
Control_HV-59_2000155740

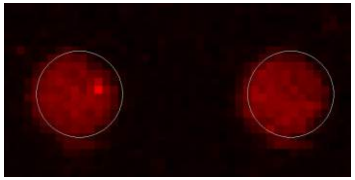
Control_HV-64_2000144456

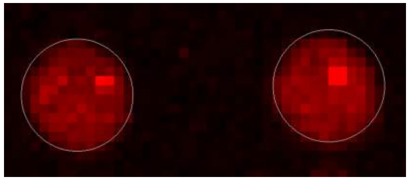
Control_HV-70_2000144457

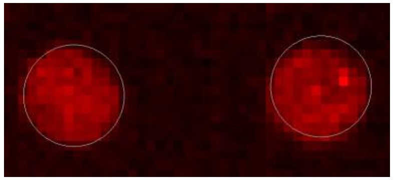
Control_HV-71_2000144458

Control

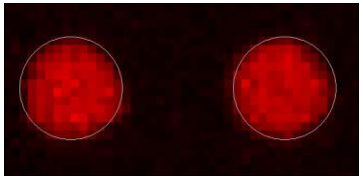

Grade II_CF 8551_2000153940

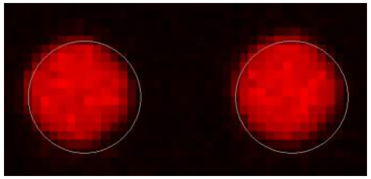

Grade II_CF 10729_2000154012

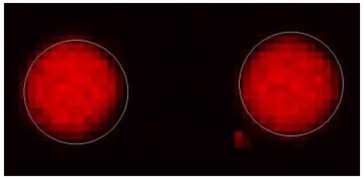

Grade II_CF 20468_2000153947

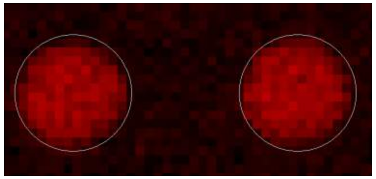

Grade II_CF 28254_2000153938

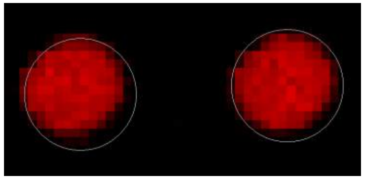

Grade II_CF 29538_2000154020

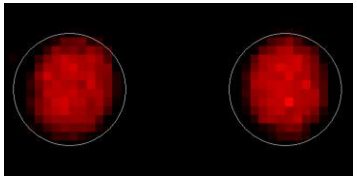

Grade II_CK 40_2000144445

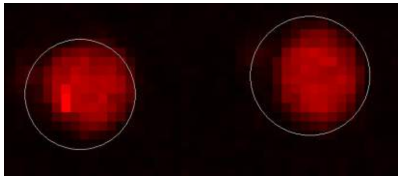

Grade II_CH 12323_2000144444

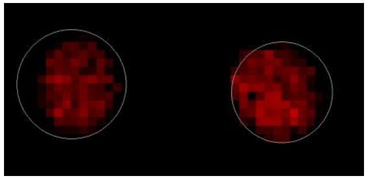

Grade II_CH 13717_2000144447

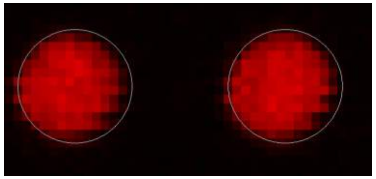

Grade II_CH 27095_2000154014

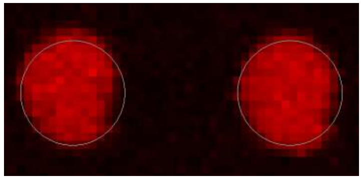

Grade II_CH 32225_2000154033

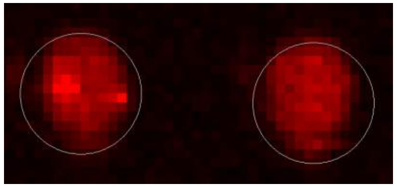

Grade II_CJ 863_20001543970

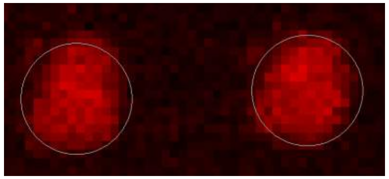

Grade II_CJ 4825_2000143981

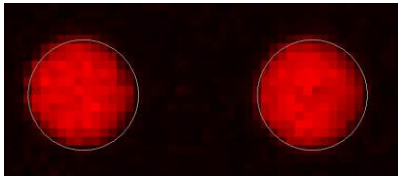

Grade II_CJ 7132_2000154028

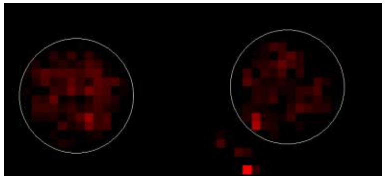

Grade II_CJ 10242_2000144449_2

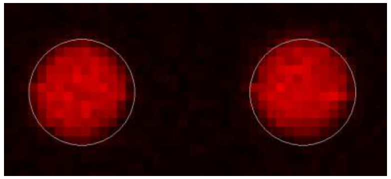

Grade II_CJ 16084_2000153931

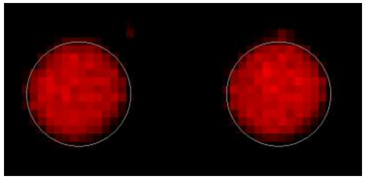

Grade II_CJ 17734_2000154015

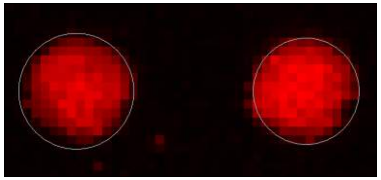

Grade II_CJ 33350_2000144452

Grade II

BC004372.1 CD44

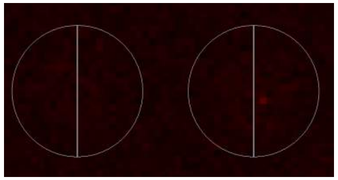
Control_H-02_2000153953

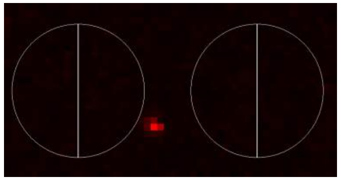
Control_H-03_2000153943

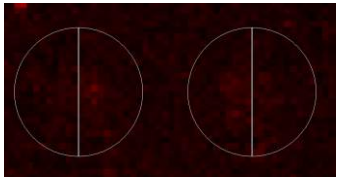
Control_H-19_2000154008

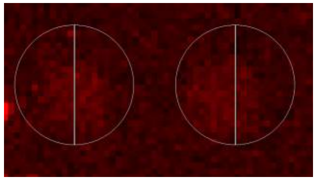
Control_HV-59_2000155740

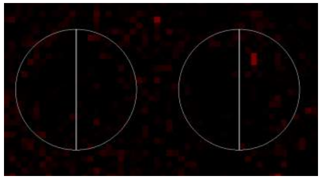
Control_H-23_2000154009

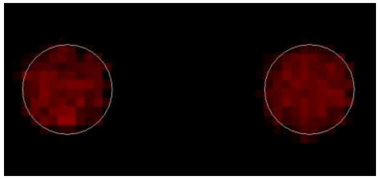
Control_H-25_2000153952

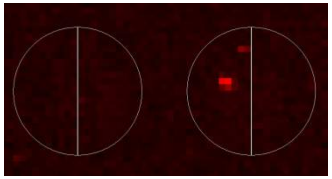
Control_H-35_2000153942

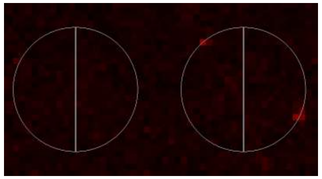
Control_HV-64_2000144456

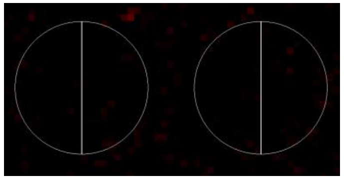
Control_H-41_2000154026

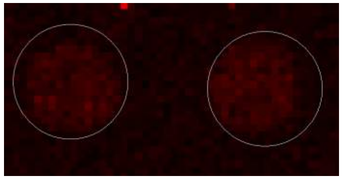
Control_H-58_2000153935

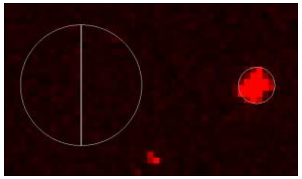
Control_H-59_2000154016

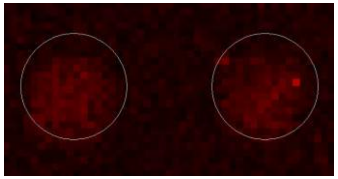
Control_HV-70_2000144457

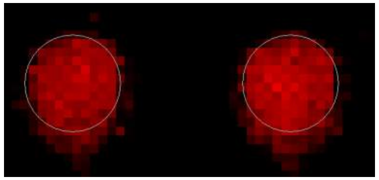
Control_HC 25_2000153932

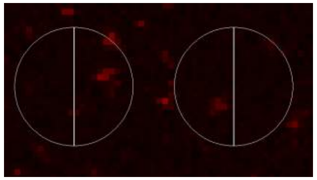
Control_HV-56_2000155735

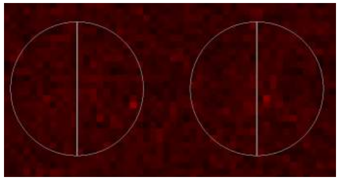
Control_HV-71_2000144458

Control

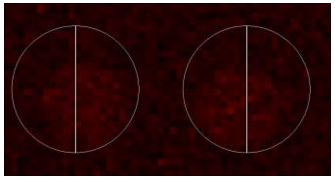

Grade II_CF 8551_2000153940

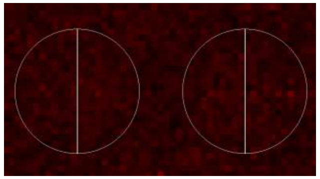

Grade II_CF 10729_2000154012

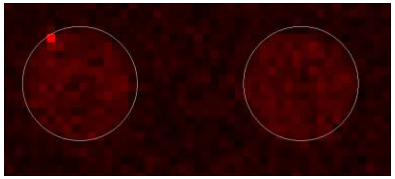

Grade II_CF 20468_2000153947

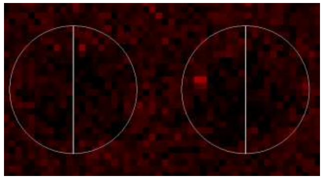

Grade II_CF 28254_2000153938

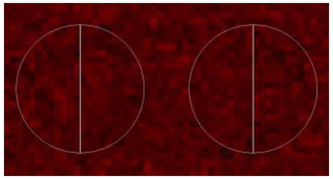

Grade II_CF 29538_2000154020

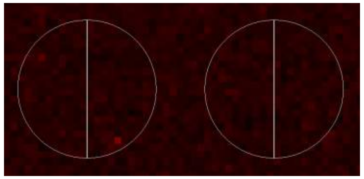

Grade II_CK 40_2000144445

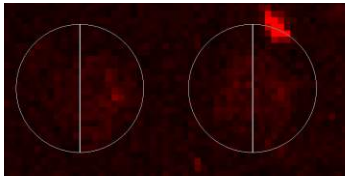

Grade II_CH 12323_2000144444

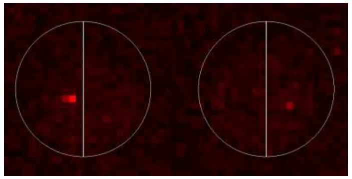

Grade II_CH 13717_2000144447

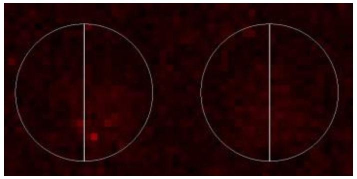

Grade II_CH 27095_2000154014

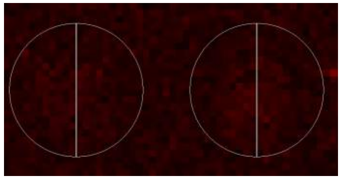

Grade II_CH 32225_2000154033

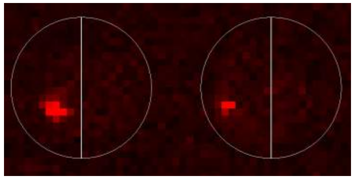

Grade II_CJ 863_20001543970

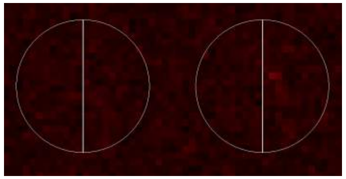

Grade II_CJ 4825_2000143981

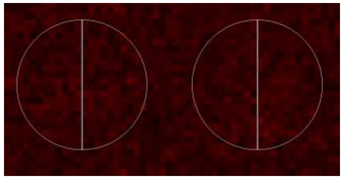

Grade II_CJ 7132_2000154028

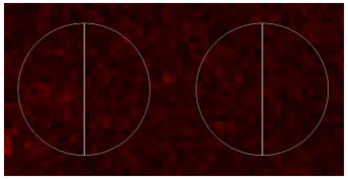

Grade II_CJ 10242_2000144449_2

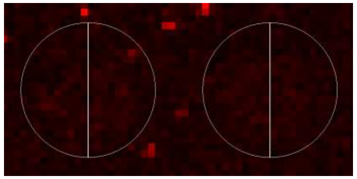

Grade II_CJ 16084_2000153931

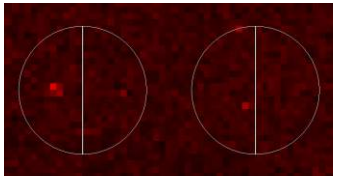

Grade II_CJ 17734_2000154015

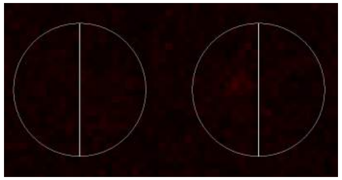

Grade II_CJ 33350_2000144452

Grade II

BC121798 EYA1

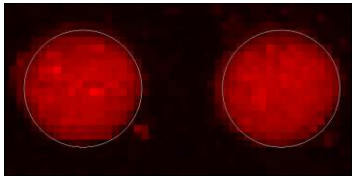
Control_H-02_2000153953

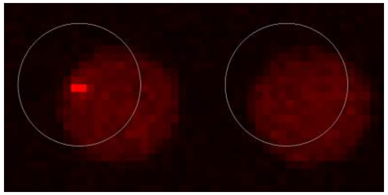
Control_H-03_2000153943

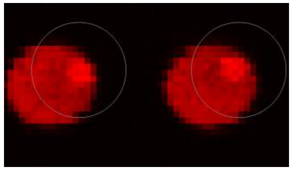
Control_H-19_2000154008

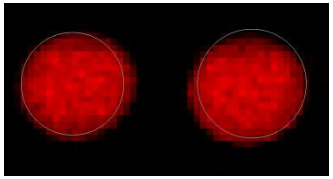
Control_H-23_2000154009

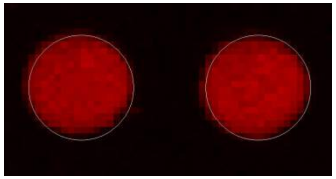
Control_H-25_2000153952

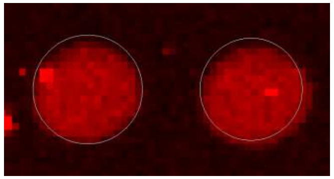
Control_H-35_2000153942

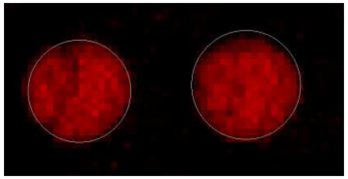
Control_H-41_2000154026

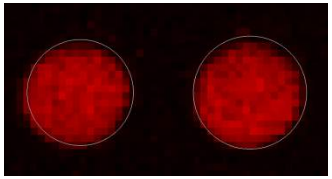
Control_H-58_2000153935

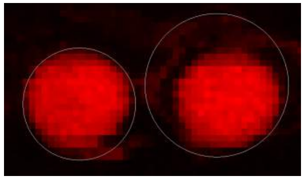
Control_H-59_2000154016

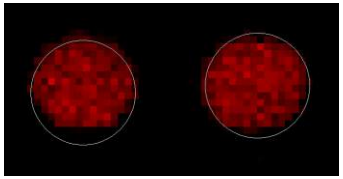
Control_HC 25_2000153932

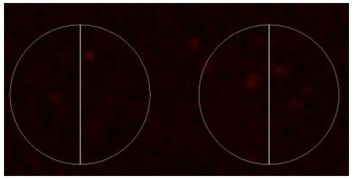
Control_HV-56_2000155735

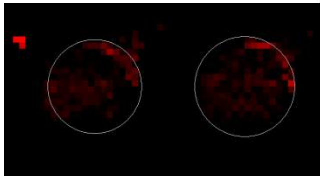
Control_HV-59_2000155740

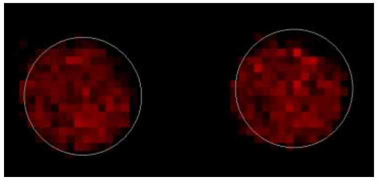
Control_HV-64_2000144456

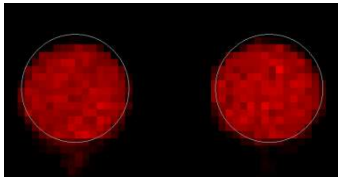
Control_HV-70_2000144457

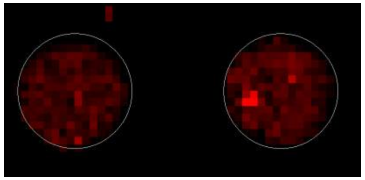
Control_HV-71_2000144458

Control

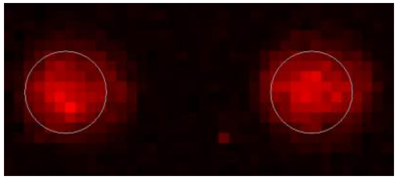
Grade II_CF 8551_2000153940

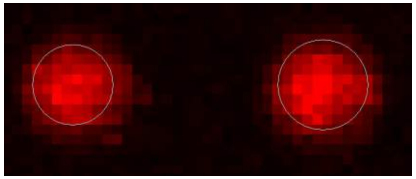
Grade II_CF 10729_2000154012

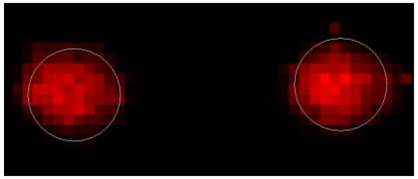
Grade II_CF 20468_2000153947

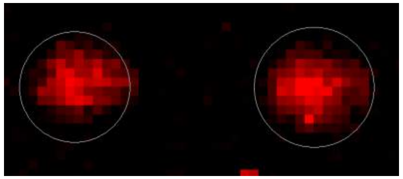
Grade II_CF 28254_2000153938

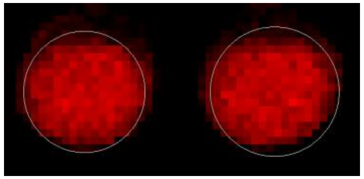
Grade II_CF 29538_2000154020

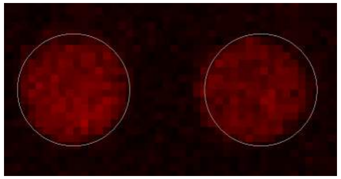
Grade II_CK 40_2000144445

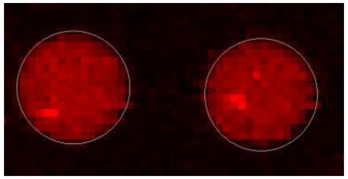
Grade II_CH 12323_2000144444

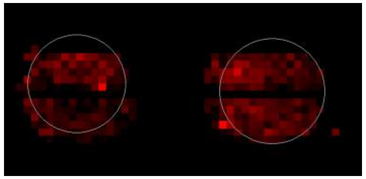
Grade II_CH 13717_2000144447

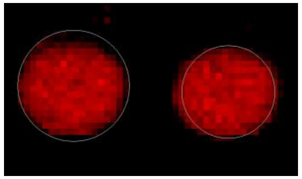
Grade II_CH 27095_2000154014

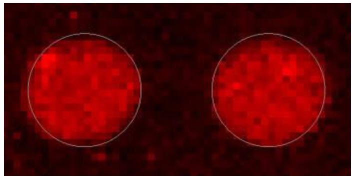
Grade II_CH 32225_2000154033

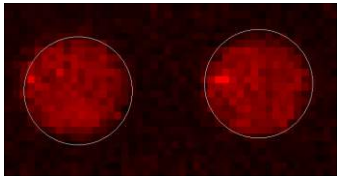
Grade II_CJ 863_20001543970

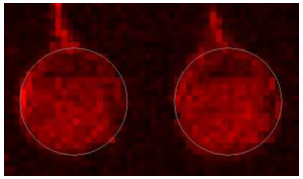
Grade II_CJ 4825_2000143981

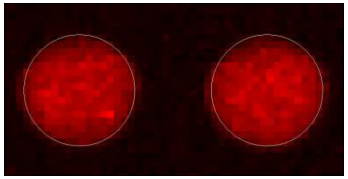
Grade II_CJ 7132_2000154028

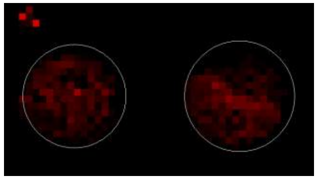
Grade II_CJ 10242_2000144449_2

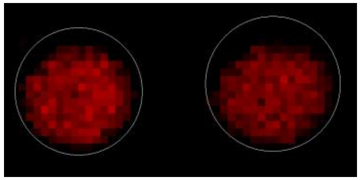
Grade II_CJ 16084_2000153931

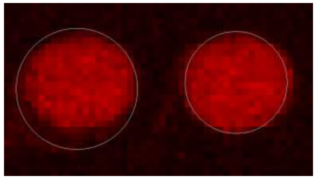
Grade II_CJ 17734_2000154015

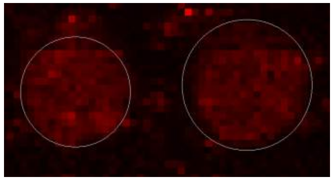
Grade II_CJ 33350_2000144452

Grade II

NM_015004.2 EXOSC7

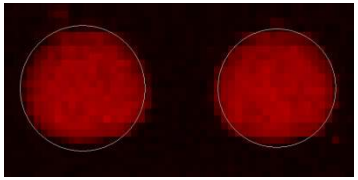
Control_H-02_2000153953

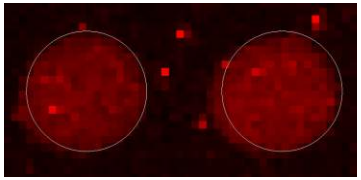
Control_H-03_2000153943

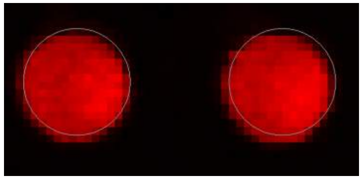
Control_H-19_2000154008

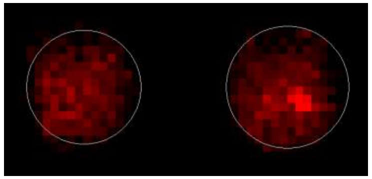
Control_HV-59_2000155740

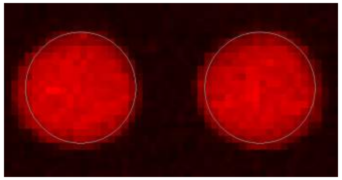
Control_H-23_2000154009

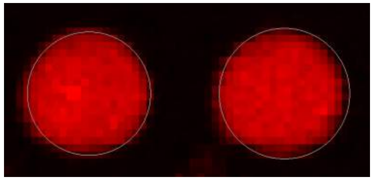
Control_H-25_2000153952

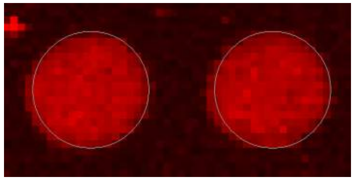
Control_H-35_2000153942

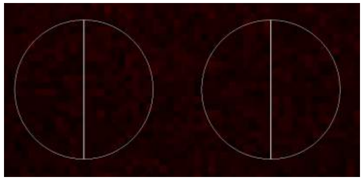
Control_HV-64_2000144456

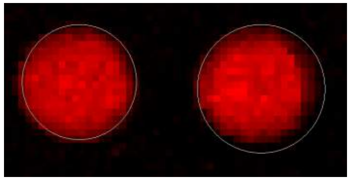
Control_H-41_2000154026

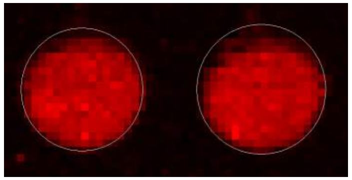
Control_H-58_2000153935

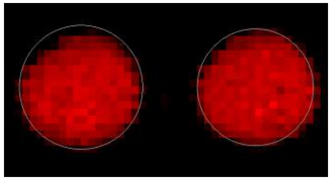
Control_H-59_2000154016

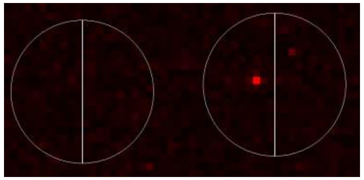
Control_HV-70_2000144457

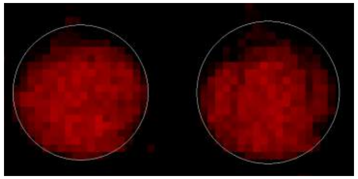
Control_HC 25_2000153932

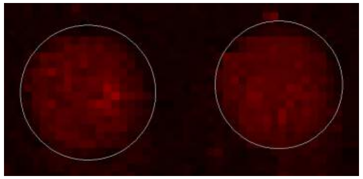
Control_HV-56_2000155735

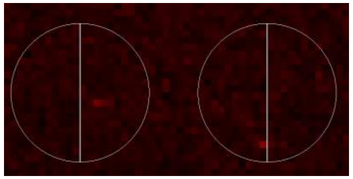
Control_HV-71_2000144458

Control

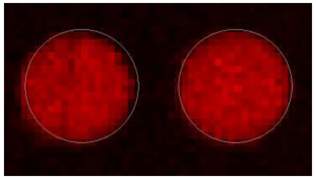

Grade II_CF 8551_2000153940

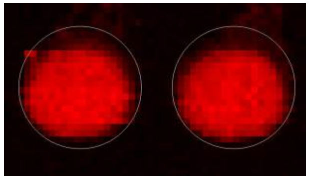

Grade II_CF 10729_2000154012

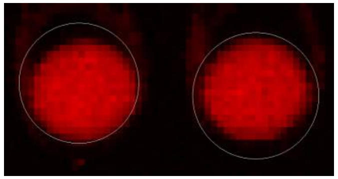

Grade II_CF 20468_2000153947

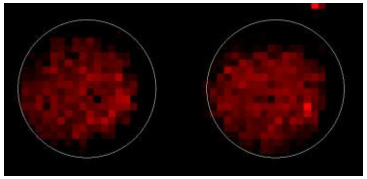

Grade II_CF 28254_2000153938

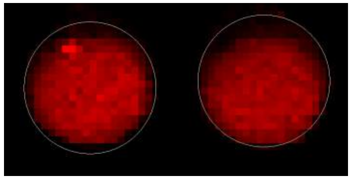

Grade II_CF 29538_2000154020

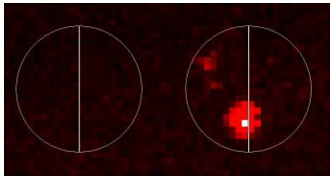

Grade II_CK 40_2000144445

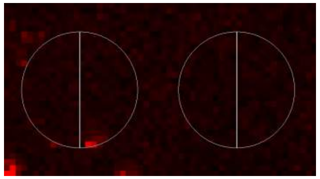

Grade II_CH 12323_2000144444

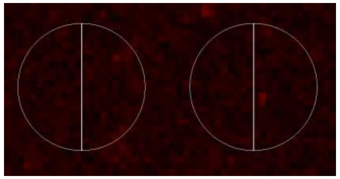

Grade II_CH 13717_2000144447

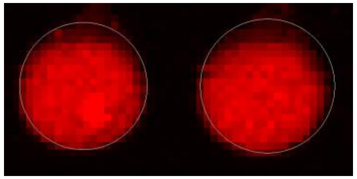

Grade II_CH 27095_2000154014

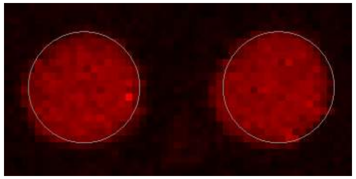

Grade II_CH 32225_2000154033

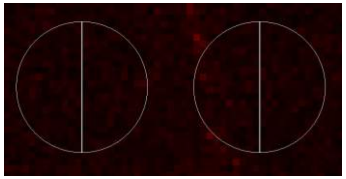

Grade II_CJ 863_20001543970

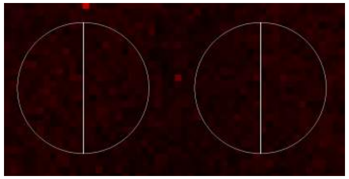

Grade II_CJ 4825_2000143981

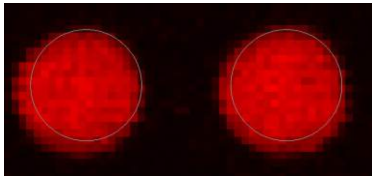

Grade II_CJ 7132_2000154028

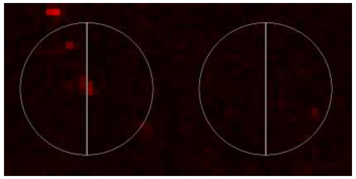

Grade II_CJ 10242_2000144449_2

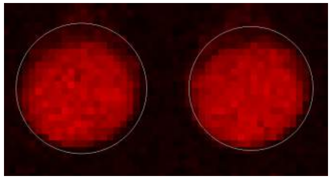

Grade II_CJ 16084_2000153931

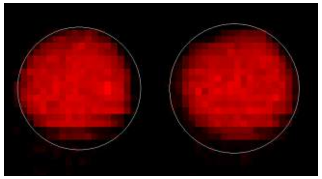

Grade II_CJ 17734_2000154015

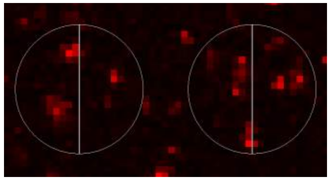

Grade II_CJ 33350_2000144452

Grade II

Nol3 NOL3

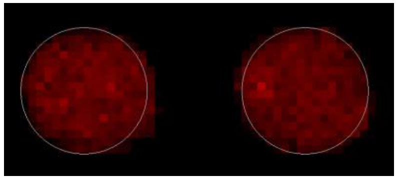
Control_H-02_2000153953

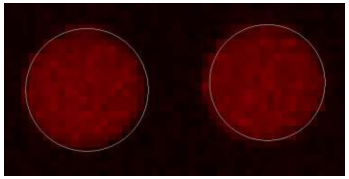
Control_H-03_2000153943

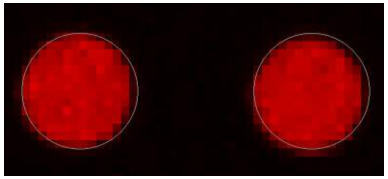
Control_H-19_2000154008

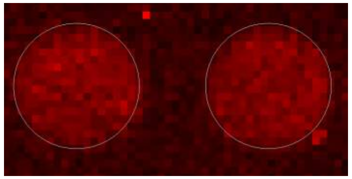
Control_HV-59_2000155740

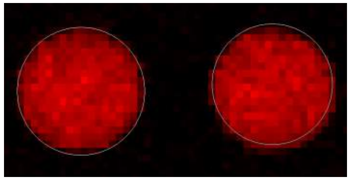
Control_H-23_2000154009

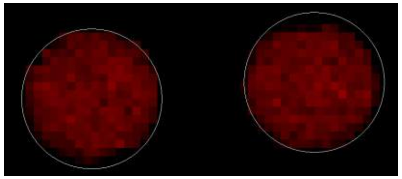
Control_H-25_2000153952

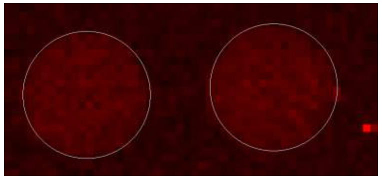
Control_H-35_2000153942

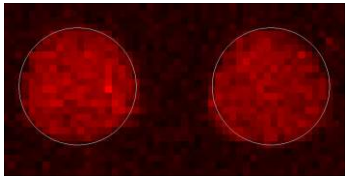
Control_HV-64_2000144456

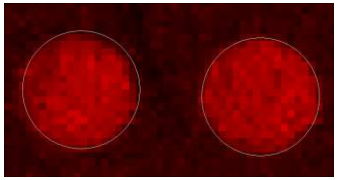
Control_H-41_2000154026

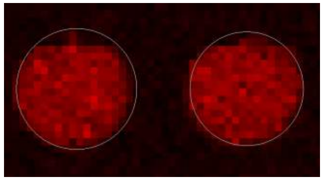
Control_H-58_2000153935

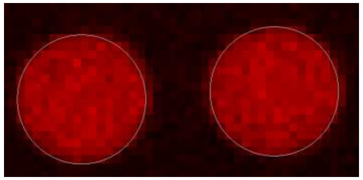
Control_H-59_2000154016

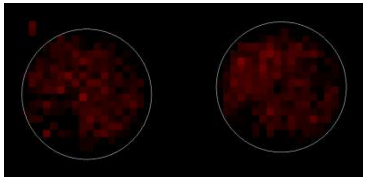
Control_HV-70_2000144457

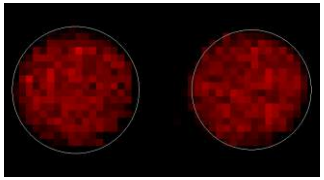
Control_HC 25_2000153932

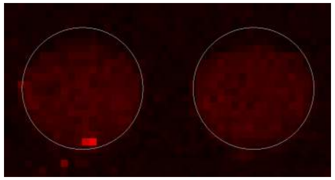
Control_HV-56_2000155735

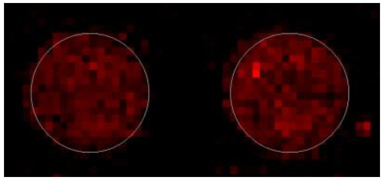
Control_HV-71_2000144458

Control

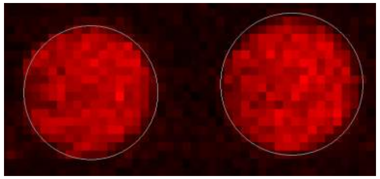

Grade II_CF 8551_2000153940

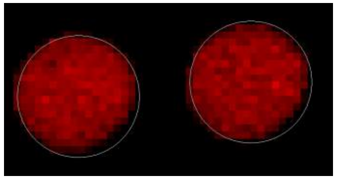

Grade II_CF 10729_2000154012

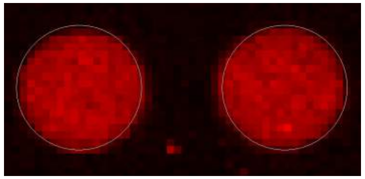

Grade II_CF 20468_2000153947

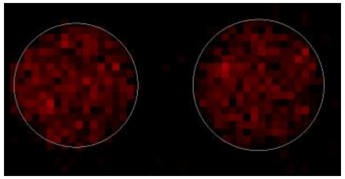

Grade II_CF 28254_2000153938

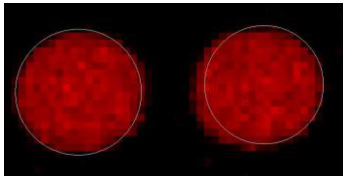

Grade II_CF 29538_2000154020

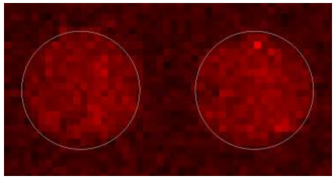

Grade II_CK 40_2000144445

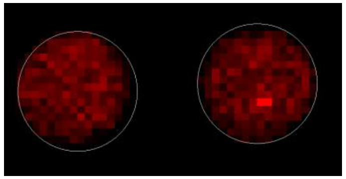

Grade II_CH 12323_2000144444

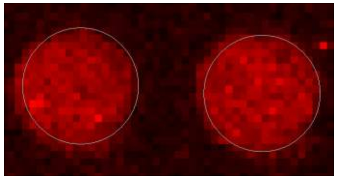

Grade II_CH 13717_2000144447

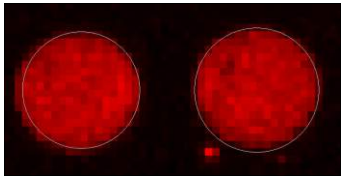

Grade II_CH 27095_2000154014

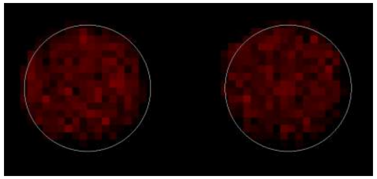

Grade II_CH 32225_2000154033

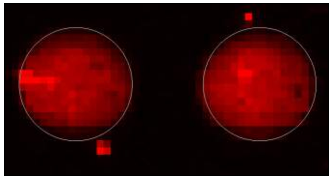

Grade II_CJ 863_20001543970

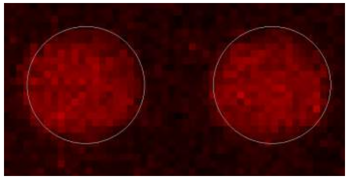

Grade II_CJ 4825_2000143981

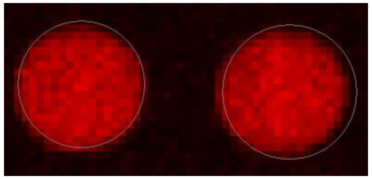

Grade II_CJ 7132_2000154028

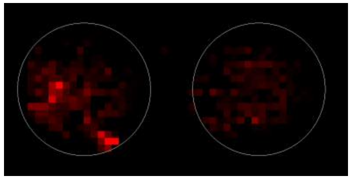

Grade II_CJ 10242_2000144449_2

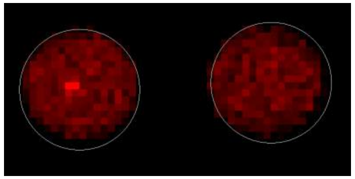

Grade II_CJ 16084_2000153931

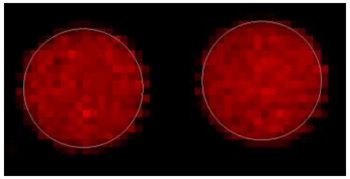

Grade II_CJ 17734_2000154015

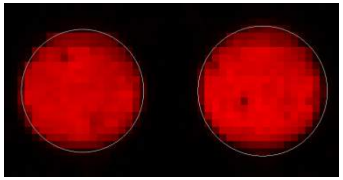

Grade II_CJ 33350_2000144452

Grade II

Control Vs Grade III

NM_003099.3 SNX1

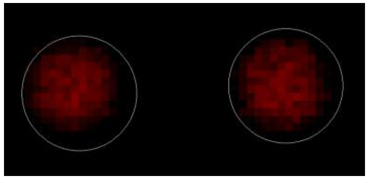

Control_H-02_2000153953

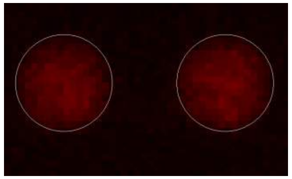

Control_H-03_2000153943

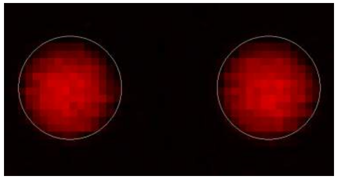

Control_H-19_2000154008

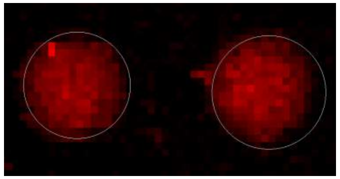

Control_H-23_2000154009

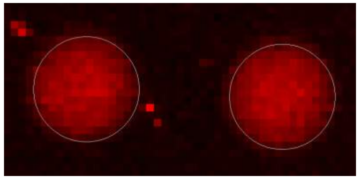

Control_H-25_2000153952

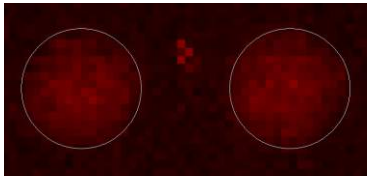

Control_H-35_2000153942

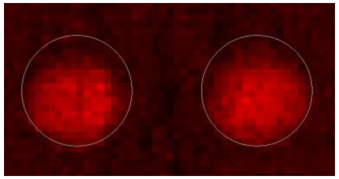

Control_H-41_2000154026

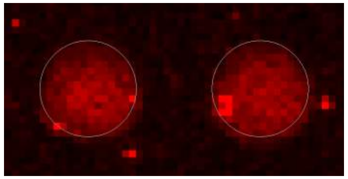

Control_H-58_2000153935

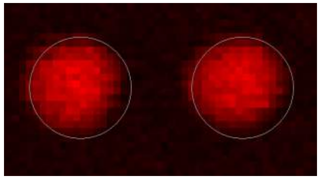

Control_H-59_2000154016

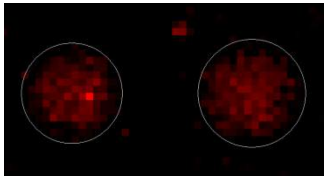

Control_HC 25_2000153932

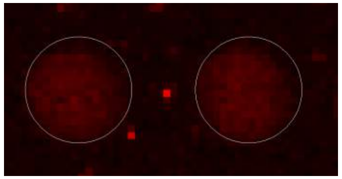

Control_HV-56_2000155735

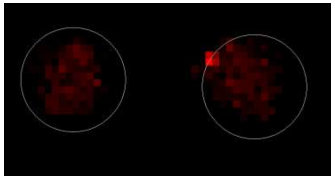

Control_HV-59_2000155740

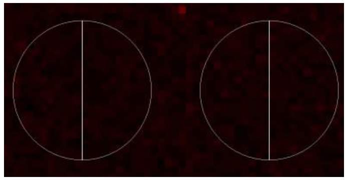

Control_HV-64_2000144456

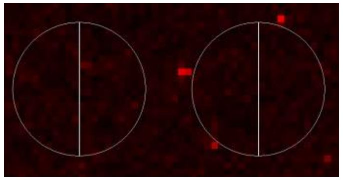

Control_HV-70_2000144457

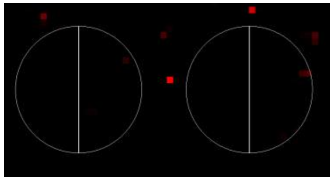

Control_HV-71_2000144458

Control

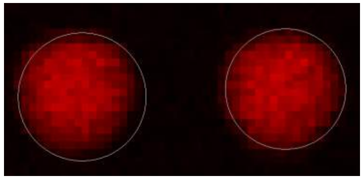

Grade III_CF 12839_2000143982

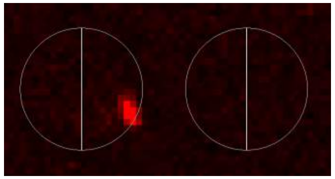

Grade III_CF 16216_2000144451

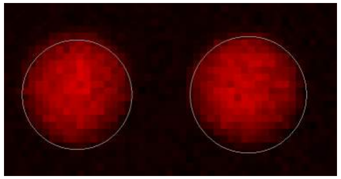

Grade III_CF 22483_2000144446

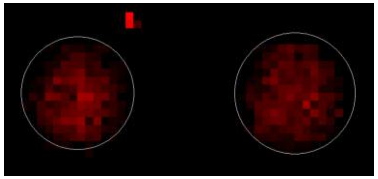

Grade III_CF 22807_2000154035

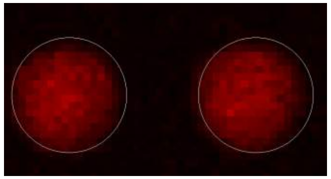

Grade III_CF 25539_2000143971

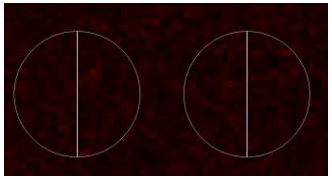

Grade III_CF 29446_2000144453

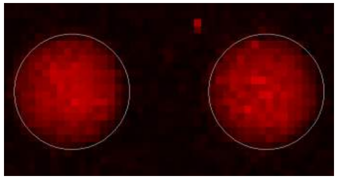

Grade III_CF 6980_2000154032

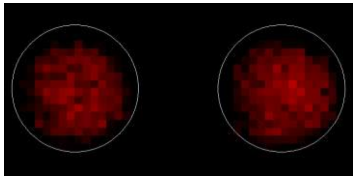

Grade III_CH 15130_2000143974

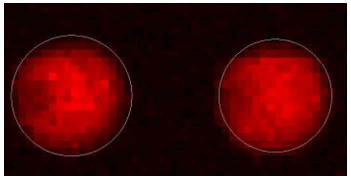

Grade III_CH 15138_2000143946

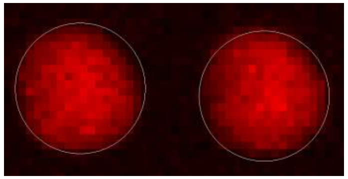

Grade III_CH 17244_2000144448

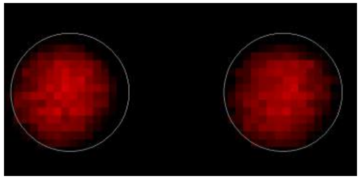

Grade III_CH 9010_2000143984

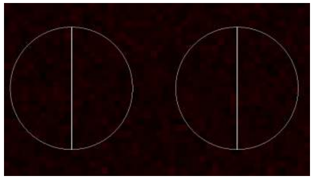

Grade III_CH 9043_2000144459

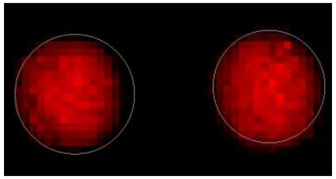

Grade III_CJ 1176_2000154027

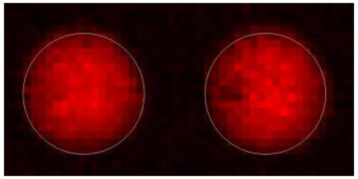

Grade III_CJ 14116_2000144450

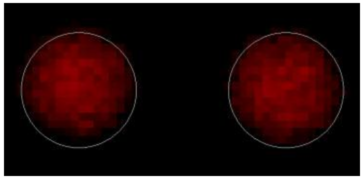

Grade III_CJ 16912_2000154006

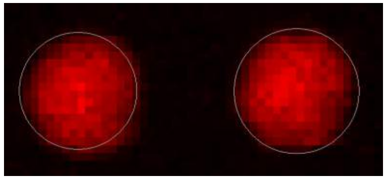

Grade III_CJ 20721_2000143947

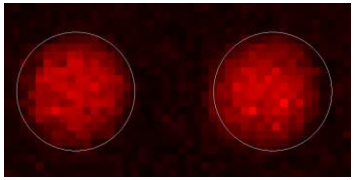

Grade III_CJ 7833_2000153939

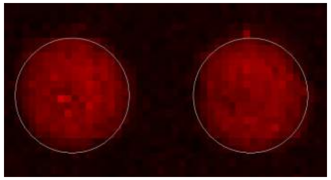

Grade III_CJ 9761_2000143973

Grade III

NM_020532.4 RTN4

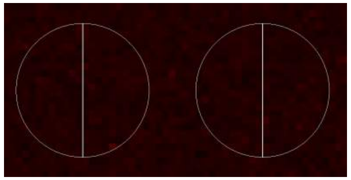
Control_H-02_2000153953

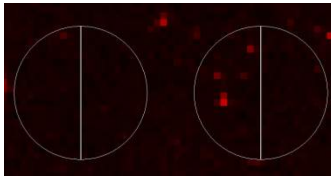
Control_H-03_2000153943

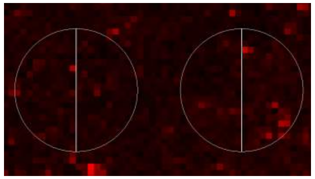
Control_H-19_2000154008

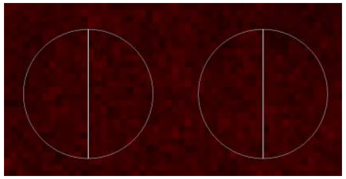
Control_H-23_2000154009

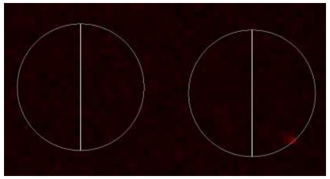
Control_H-25_2000153952

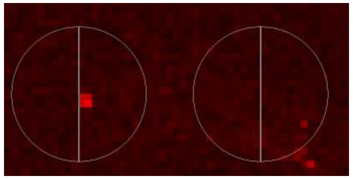
Control_H-35_2000153942

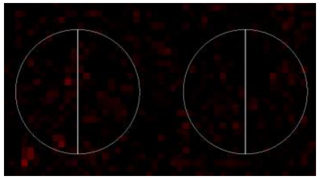
Control_H-41_2000154026

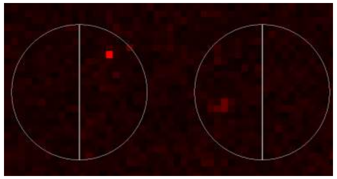
Control_H-58_2000153935

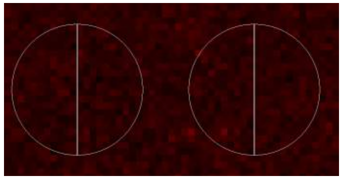
Control_H-59_2000154016

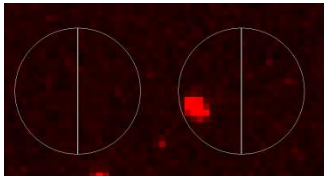
Control_HC 25_2000153932

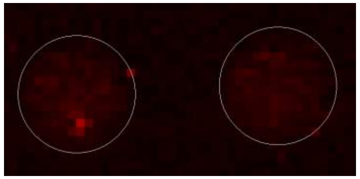
Control_HV-56_2000155735

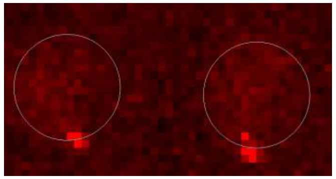
Control_HV-59_2000155740

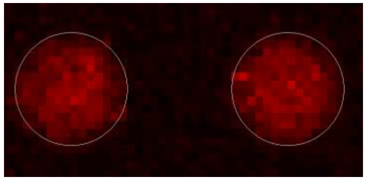
Control_HV-64_2000144456

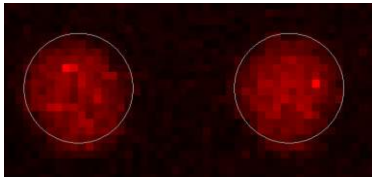
Control_HV-70_2000144457

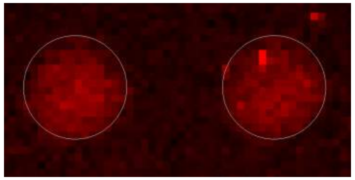
Control_HV-71_2000144458

Control

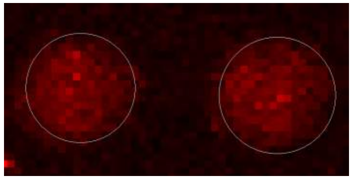
Grade III_CF 12839_2000143982

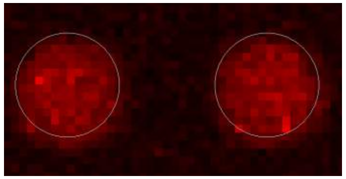
Grade III_CF 16216_2000144451

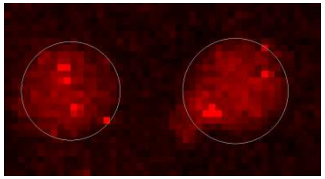
Grade III_CF 22483_2000144446

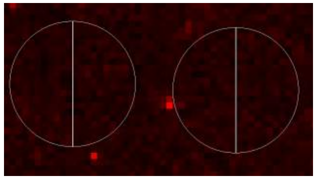
Grade III_CF 22807_2000154035

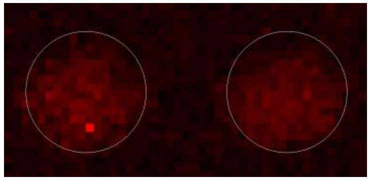
Grade III_CF 25539_2000143971

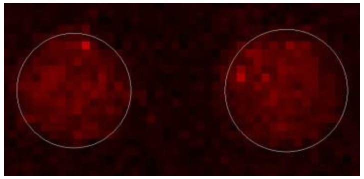
Grade III_CF 29446_2000144453

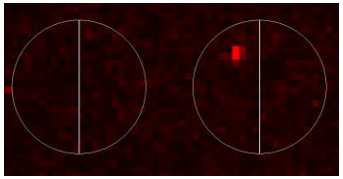
Grade III_CF 6980_2000154032

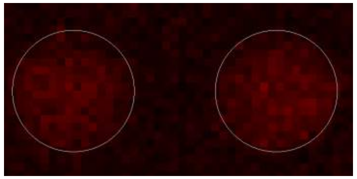
Grade III_CH 15130_2000143974

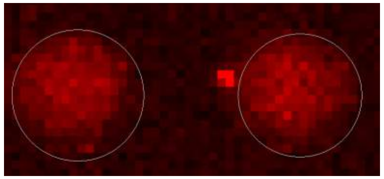
Grade III_CH 15138_2000143946

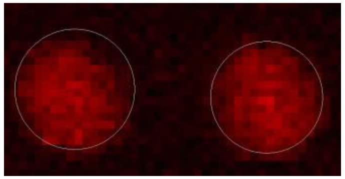
Grade III_CH 17244_2000144448

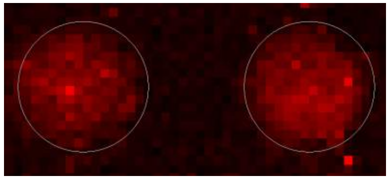
Grade III_CH 9010_2000143984

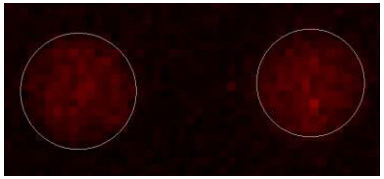
Grade III_CH 9043_2000144459

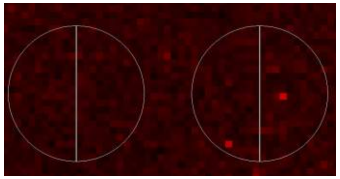
Grade III_CJ 1176_2000154027

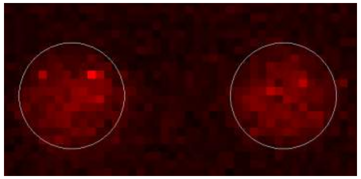
Grade III_CJ 14116_2000144450

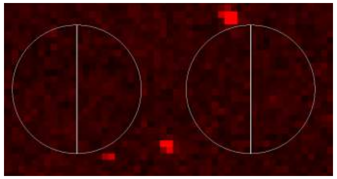
Grade III_CJ 16912_2000154006

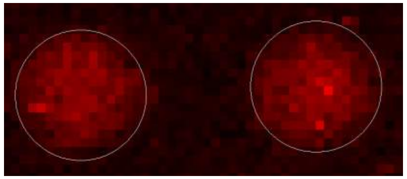
Grade III_CJ 20721_2000143947

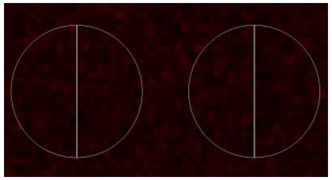
Grade III_CJ 7833_2000153939

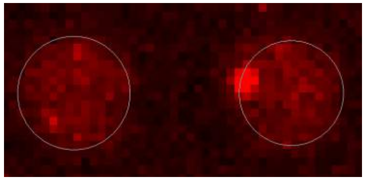
Grade III_CJ 9761_2000143973

Grade III

NM_016246.2 HSD17B14

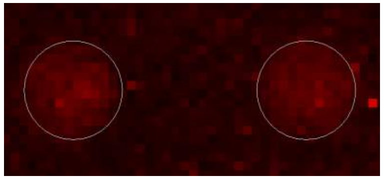
Control_H-02_2000153953

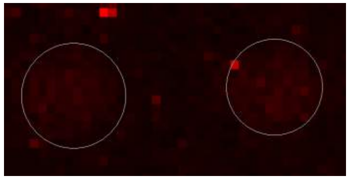
Control_H-03_2000153943

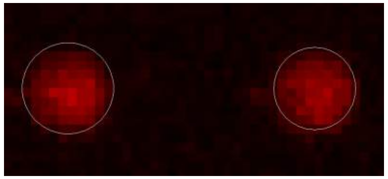
Control_H-19_2000154008

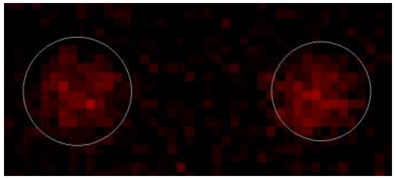
Control_H-23_2000154009

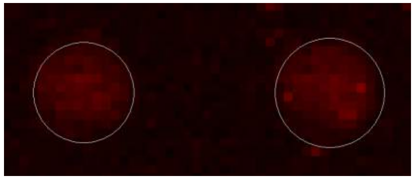
Control_H-25_2000153952

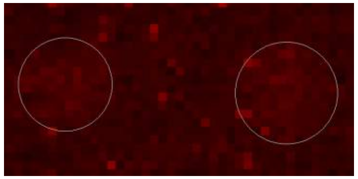
Control_H-35_2000153942

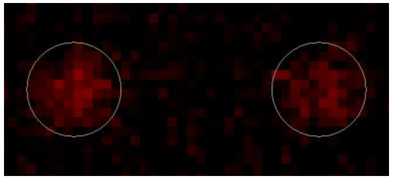
Control_H-41_2000154026

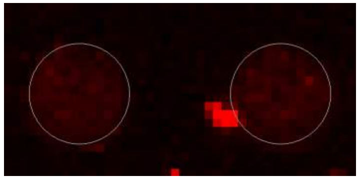
Control_H-58_2000153935

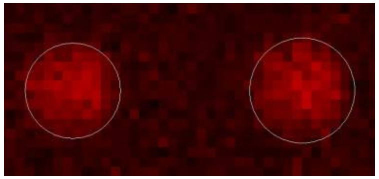
Control_H-59_2000154016

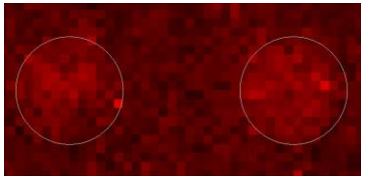
Control_HC 25_2000153932

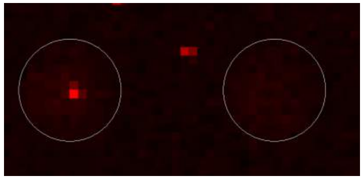
Control_HV-56_2000155735

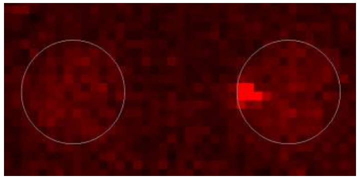
Control_HV-59_2000155740

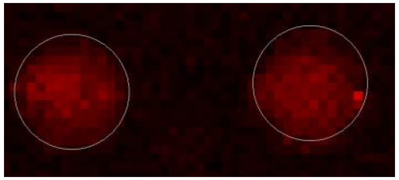
Control_HV-64_2000144456

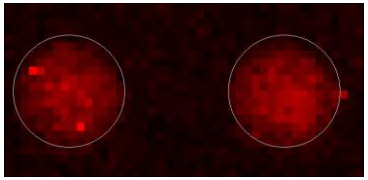
Control_HV-70_2000144457

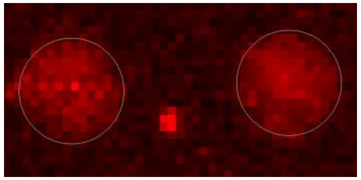
Control_HV-71_2000144458

Control

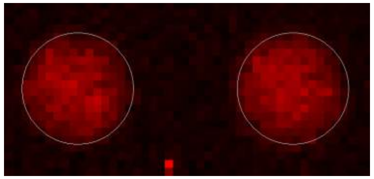
Grade III_CF 12839_2000143982

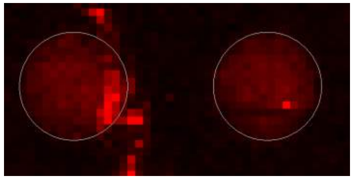
Grade III_CF 16216_2000144451

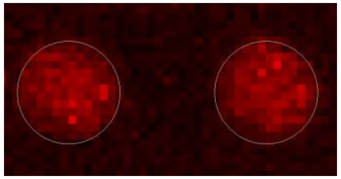
Grade III_CF 22483_2000144446

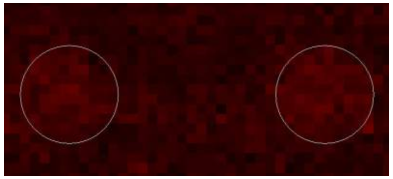
Grade III_CF 22807_2000154035

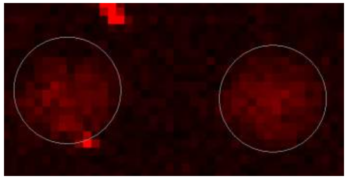
Grade III_CF 25539_2000143971

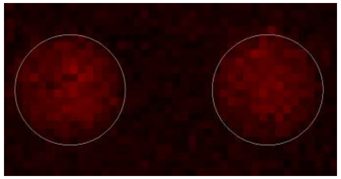
Grade III_CF 29446_2000144453

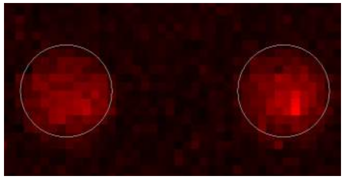
Grade III_CF 6980_2000154032

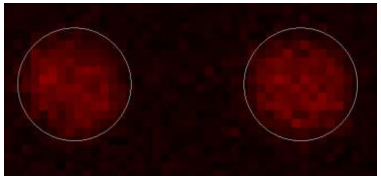
Grade III_CH 15130_2000143974

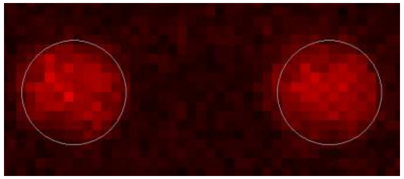
Grade III_CH 15138_2000143946

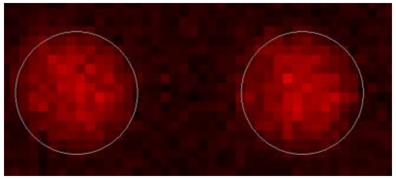
Grade III_CH 17244_2000144448

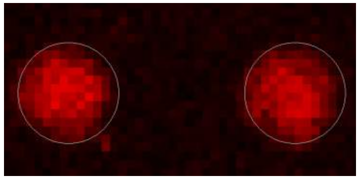
Grade III_CH 9010_2000143984

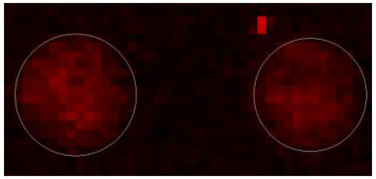
Grade III_CH 9043_2000144459

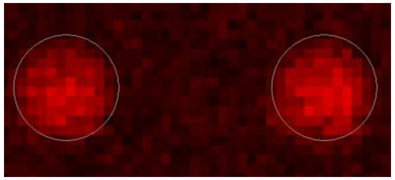
Grade III_CJ 1176_2000154027

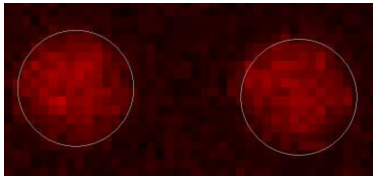
Grade III_CJ 14116_2000144450

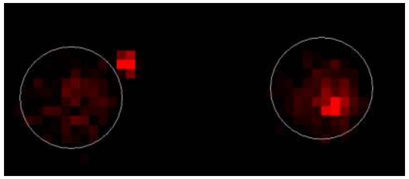
Grade III_CJ 16912_2000154006

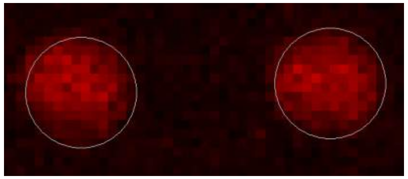
Grade III_CJ 20721_2000143947

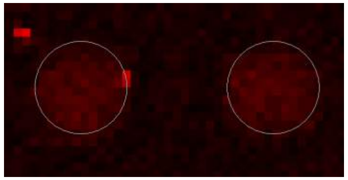
Grade III_CJ 7833_2000153939

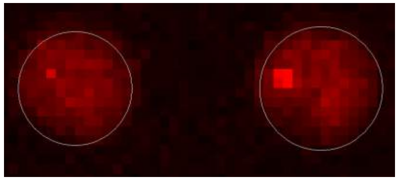
Grade III_CJ 9761_2000143973

Grade III

BC001890.1 GCK

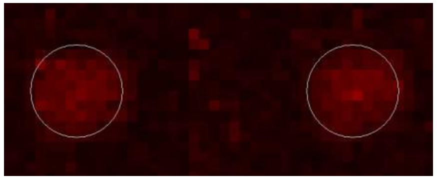
Control_H-02_2000153953

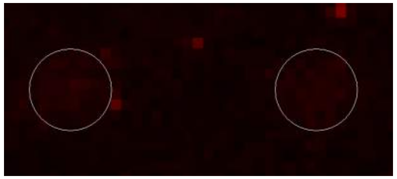
Control_H-03_2000153943

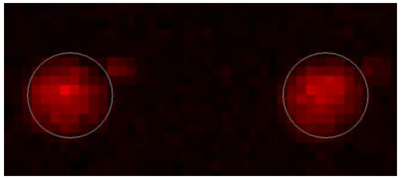
Control_H-19_2000154008

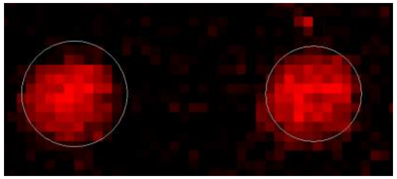
Control_H-23_2000154009

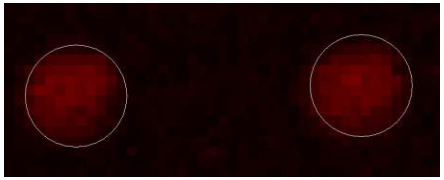
Control_H-25_2000153952

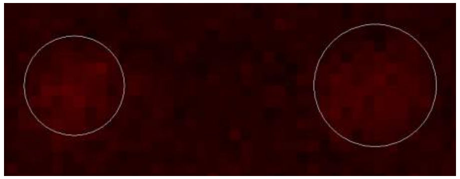
Control_H-35_2000153942

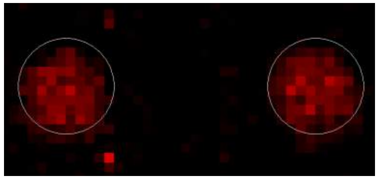
Control_H-41_2000154026

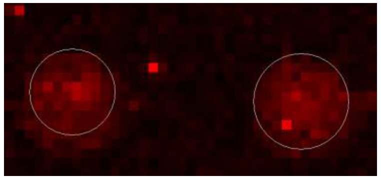
Control_H-58_2000153935

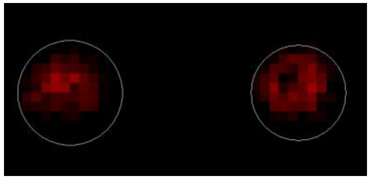
Control_H-59_2000154016

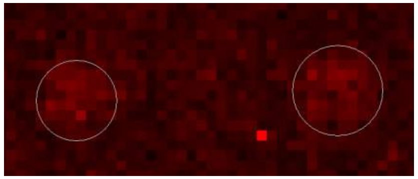
Control_HC 25_2000153932

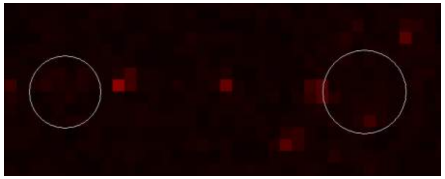
Control_HV-56_2000155735

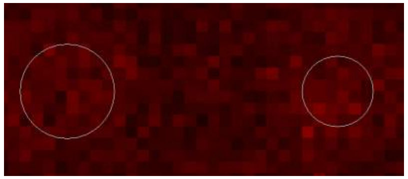
Control_HV-59_2000155740

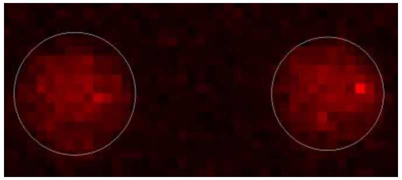
Control_HV-64_2000144456

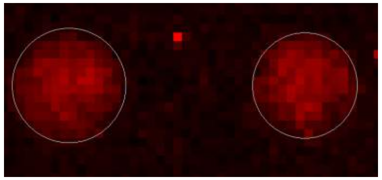
Control_HV-70_2000144457

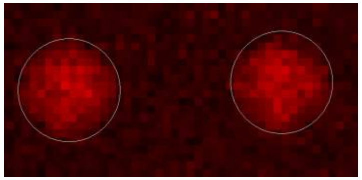
Control_HV-71_2000144458

Control

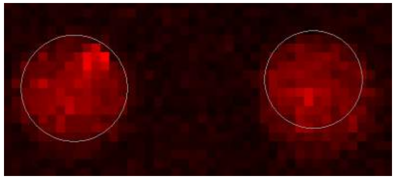

Grade III_CF 12839_2000143982

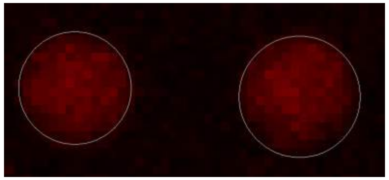

Grade III_CF 16216_2000144451

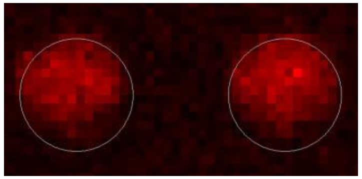

Grade III_CF 22483_2000144446

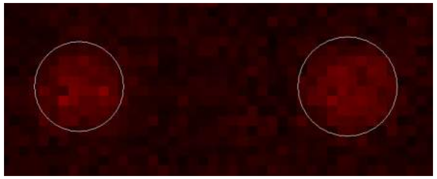

Grade III_CF 22807_2000154035

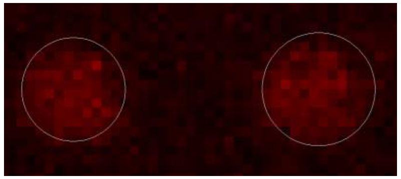

Grade III_CF 25539_2000143971

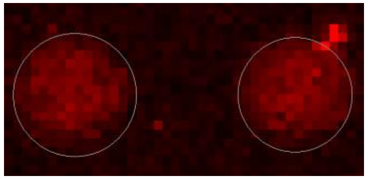

Grade III_CF 29446_2000144453

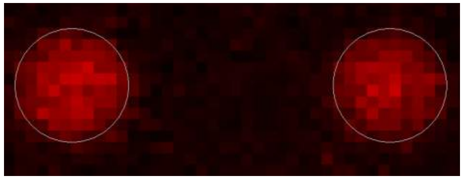

Grade III_CF 6980_2000154032

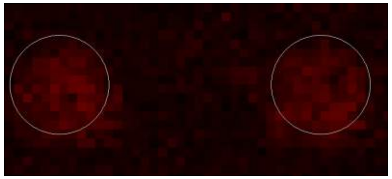

Grade III_CH 15130_2000143974

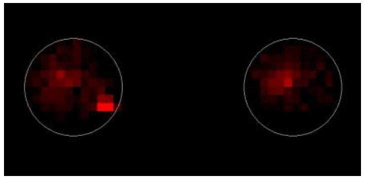

Grade III_CH 15138_2000143946

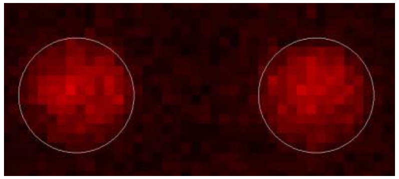

Grade III_CH 17244_2000144448

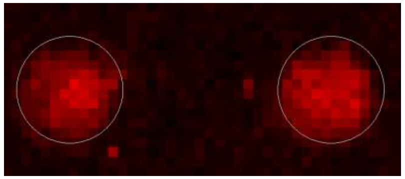

Grade III_CH 9010_2000143984

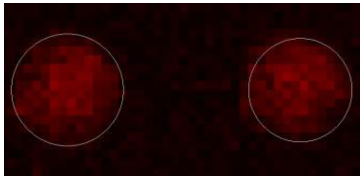

Grade III_CH 9043_2000144459

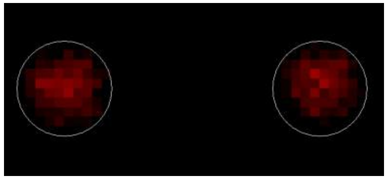

Grade III_CJ 1176_2000154027

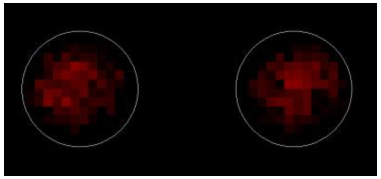

Grade III_CJ 14116_2000144450

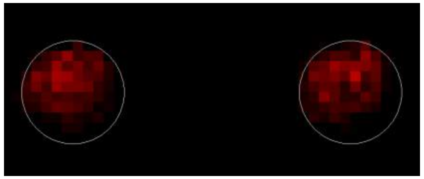

Grade III_CJ 16912_2000154006

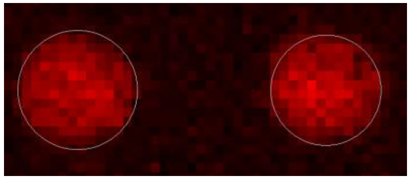

Grade III_CJ 20721_2000143947

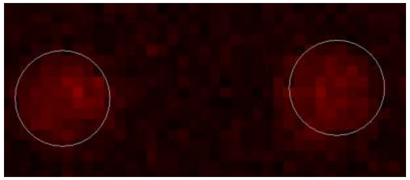

Grade III_CJ 7833_2000153939

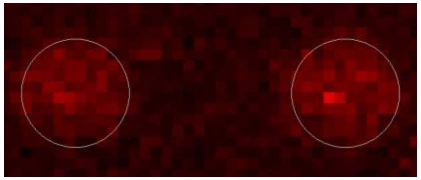

Grade III_CJ 9761_2000143973

Grade III

BC017723.1 MAGEA4

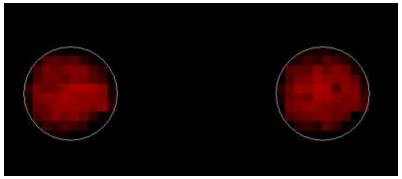
Control_H-02_2000153953

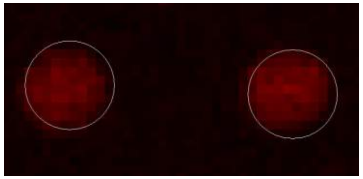
Control_H-03_2000153943

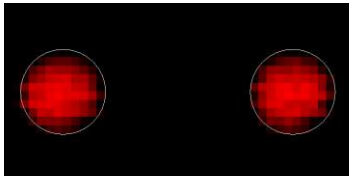
Control_H-19_2000154008

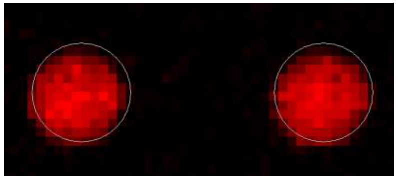
Control_H-23_2000154009

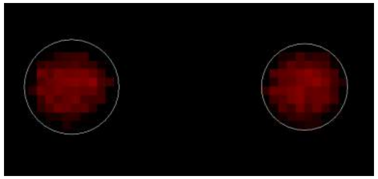
Control_H-25_2000153952

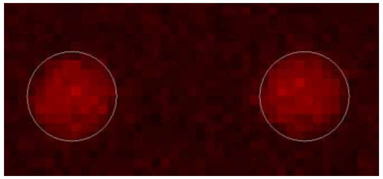
Control_H-35_2000153942

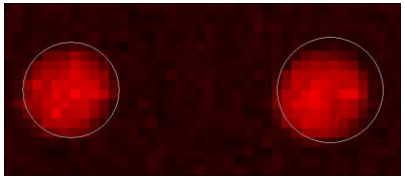
Control_H-41_2000154026

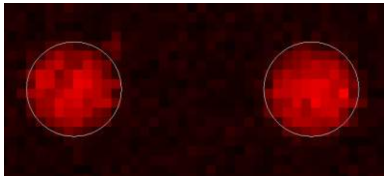
Control_H-58_2000153935

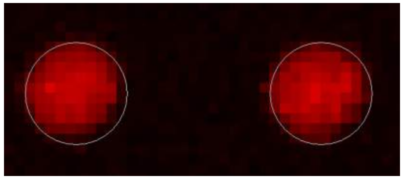
Control_H-59_2000154016

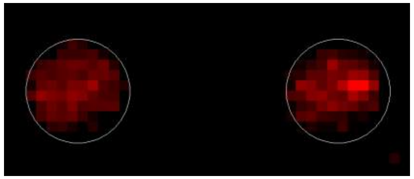
Control_HC 25_2000153932

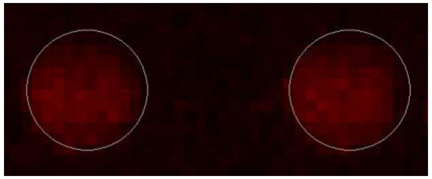
Control_HV-56_2000155735

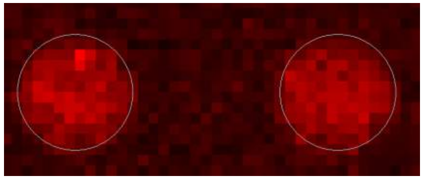
Control_HV-59_2000155740

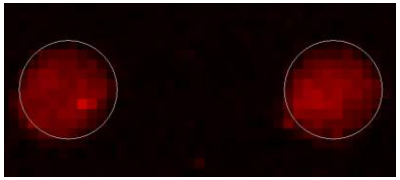
Control_HV-64_2000144456

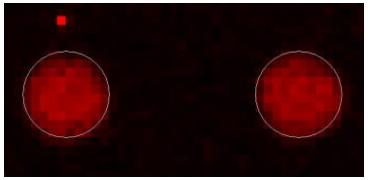
Control_HV-70_2000144457

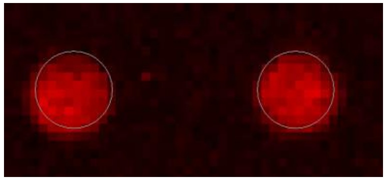
Control_HV-71_2000144458

Control

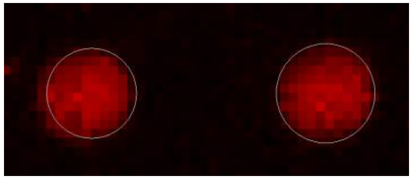
Grade III_CF 12839_2000143982

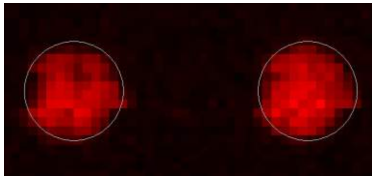
Grade III_CF 16216_2000144451

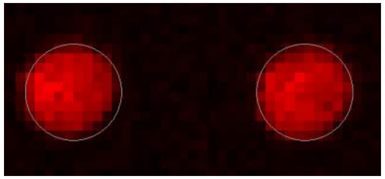
Grade III_CF 22483_2000144446

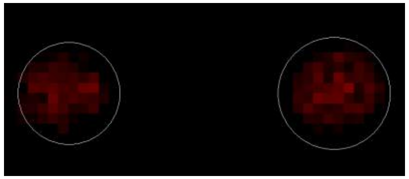
Grade III_CF 22807_2000154035

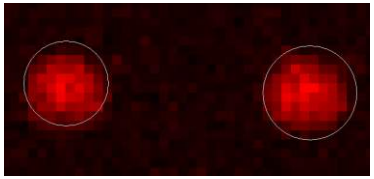
Grade III_CF 25539_2000143971

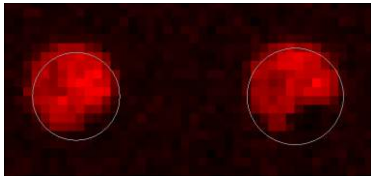
Grade III_CF 29446_2000144453

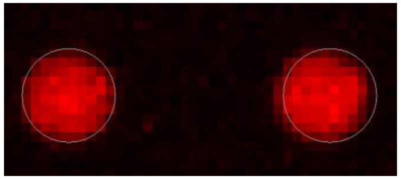
Grade III_CF 6980_2000154032

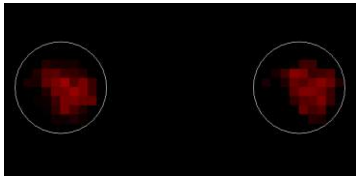
Grade III_CH 15130_2000143974

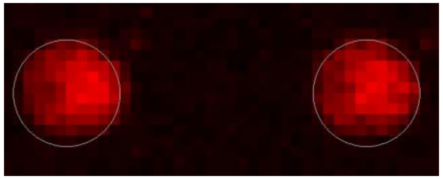
Grade III_CH 15138_2000143946

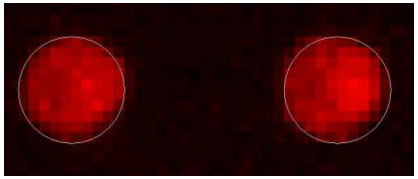
Grade III_CH 17244_2000144448

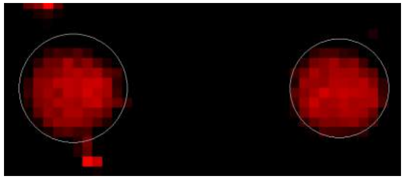
Grade III_CH 9010_2000143984

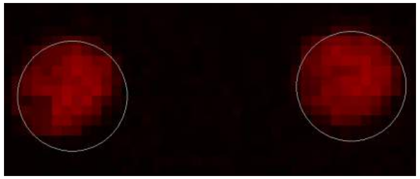
Grade III_CH 9043_2000144459

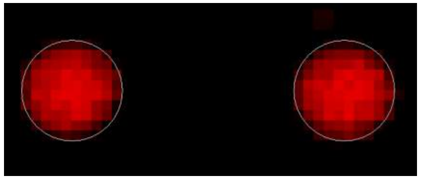
Grade III_CJ 1176_2000154027

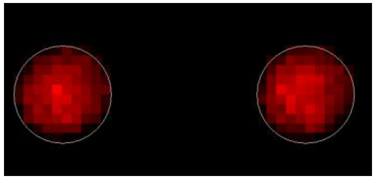
Grade III_CJ 14116_2000144450

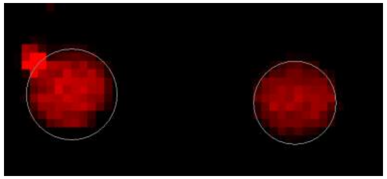
Grade III_CJ 16912_2000154006

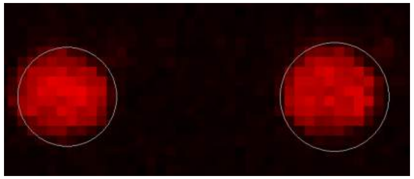
Grade III_CJ 20721_2000143947

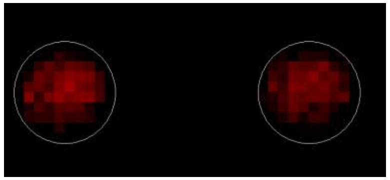
Grade III_CJ 7833_2000153939

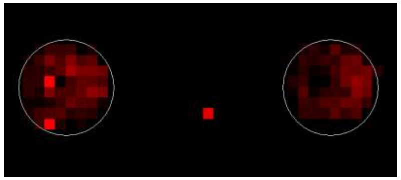
Grade III_CJ 9761_2000143973

Grade III

NM_173608.1 C14orf80

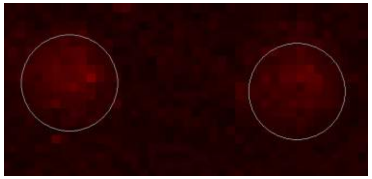
Control_H-02_2000153953

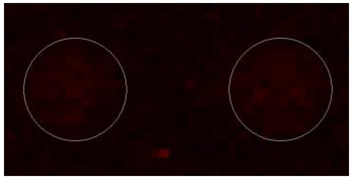
Control_H-03_2000153943

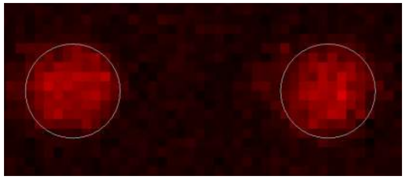
Control_H-19_2000154008

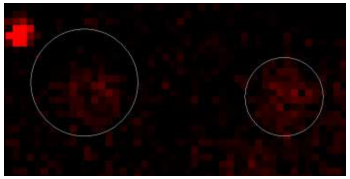
Control_H-23_2000154009

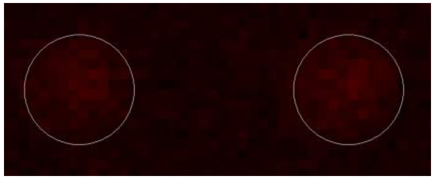
Control_H-25_2000153952

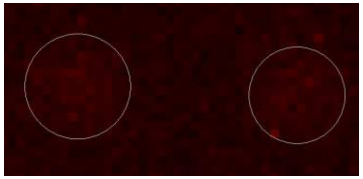
Control_H-35_2000153942

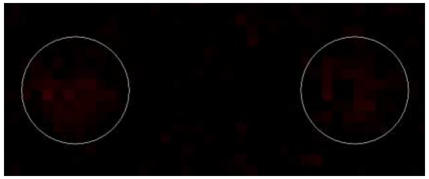
Control_H-41_2000154026

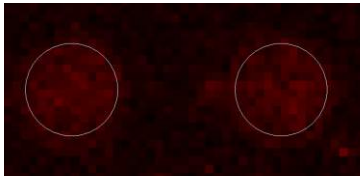
Control_H-58_2000153935

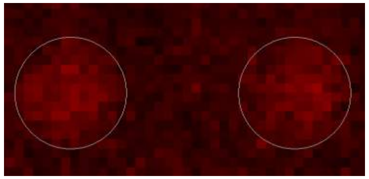
Control_H-59_2000154016

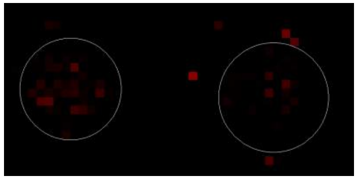
Control_HC 25_2000153932

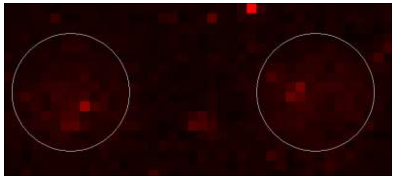
Control_HV-56_2000155735

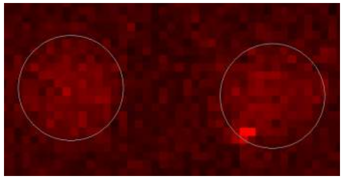
Control_HV-59_2000155740

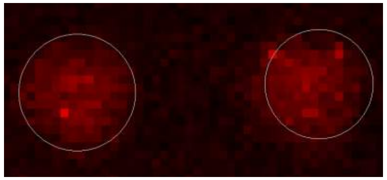
Control_HV-64_2000144456

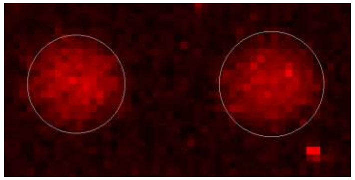
Control_HV-70_2000144457

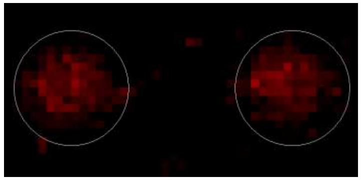
Control_HV-71_2000144458

Control

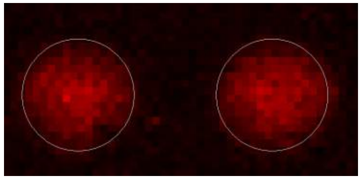
Grade III_CF 12839_2000143982

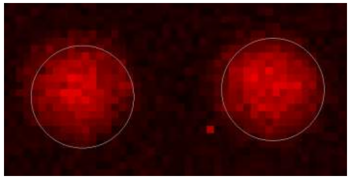
Grade III_CF 16216_2000144451

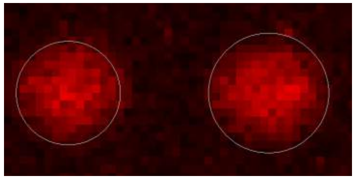
Grade III_CF 22483_2000144446

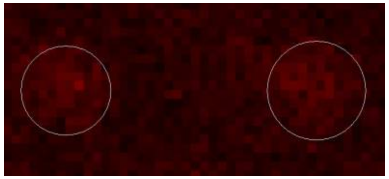
Grade III_CF 22807_2000154035

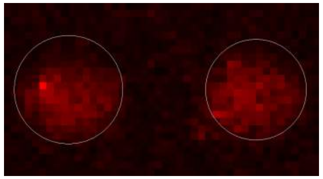
Grade III_CF 25539_2000143971

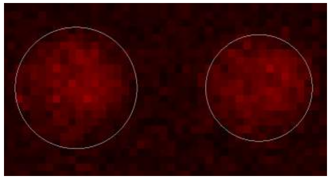
Grade III_CF 29446_2000144453

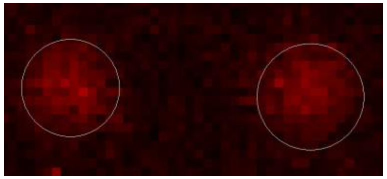
Grade III_CF 6980_2000154032

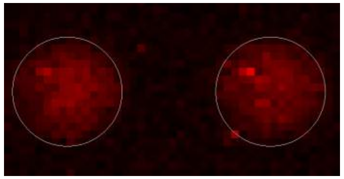
Grade III_CH 15130_2000143974

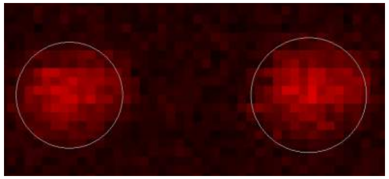
Grade III_CH 15138_2000143946

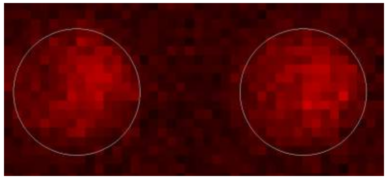
Grade III_CH 17244_2000144448

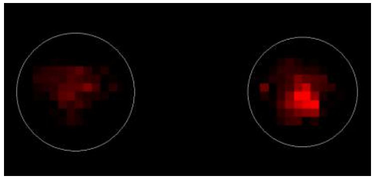
Grade III_CH 9010_2000143984

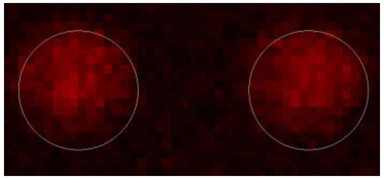
Grade III_CH 9043_2000144459

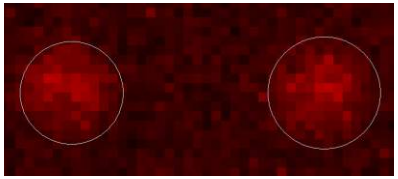
Grade III_CJ 1176_2000154027

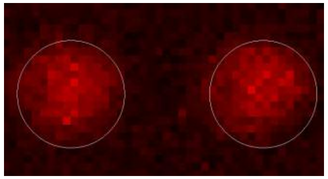
Grade III_CJ 14116_2000144450

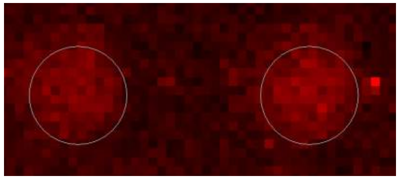
Grade III_CJ 16912_2000154006

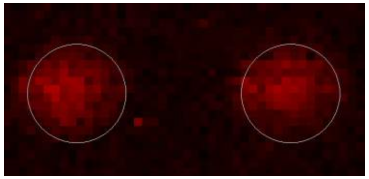
Grade III_CJ 20721_2000143947

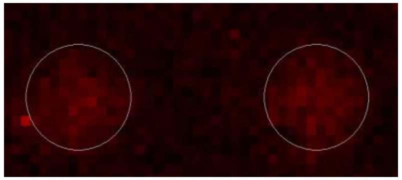
Grade III_CJ 7833_2000153939

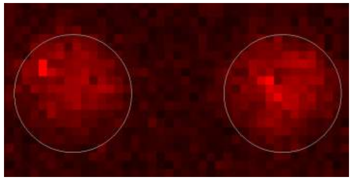
Grade III_CJ 9761_2000143973

Grade III

NM_198204.1 MLX

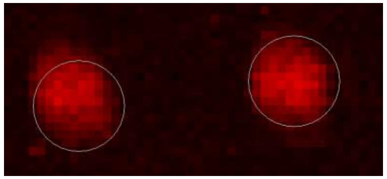

Control_H-02_2000153953

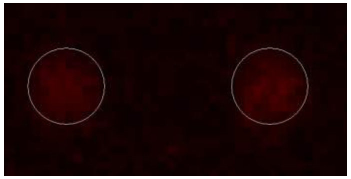

Control_H-03_2000153943

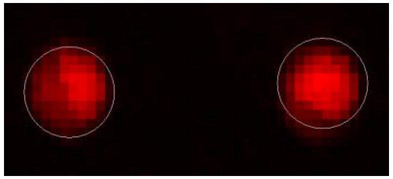

Control_H-19_2000154008

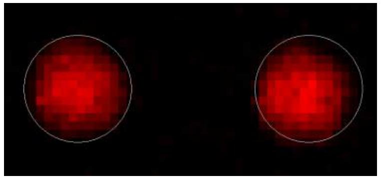

Control_H-23_2000154009

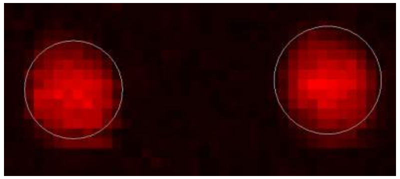

Control_H-25_2000153952

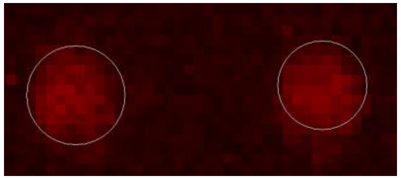

Control_H-35_2000153942

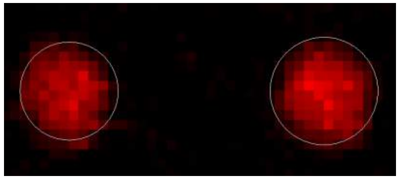

Control_H-41_2000154026

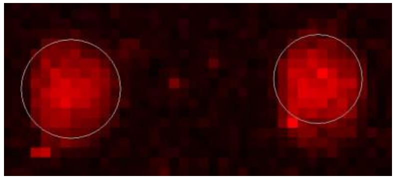

Control_H-58_2000153935

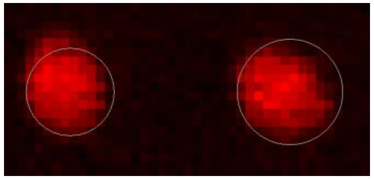

Control_H-59_2000154016

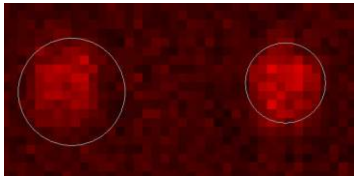

Control_HC 25_2000153932

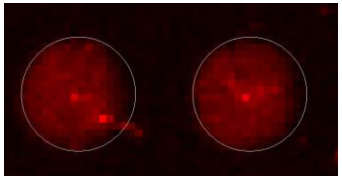

Control_HV-56_2000155735

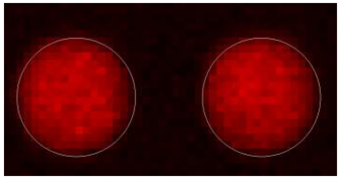

Control_HV-59_2000155740

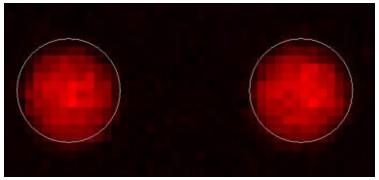

Control_HV-64_2000144456

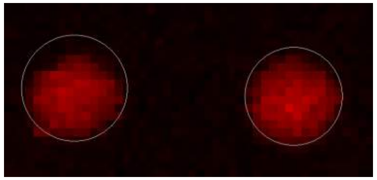

Control_HV-70_2000144457

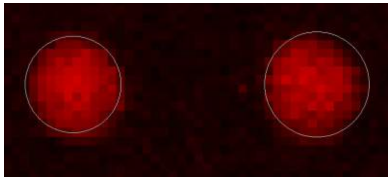

Control_HV-71_2000144458

Control

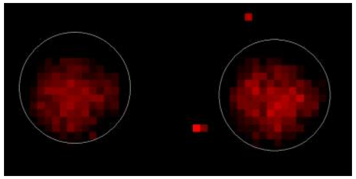

Grade III_CF 12839_2000143982

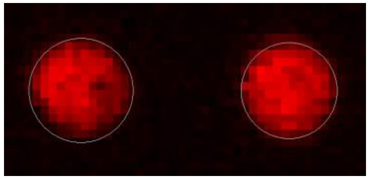

Grade III_CF 16216_2000144451

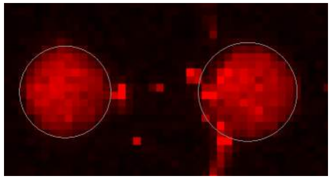

Grade III_CF 22483_2000144446

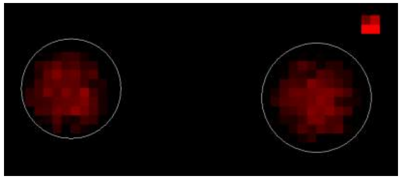

Grade III_CF 22807_2000154035

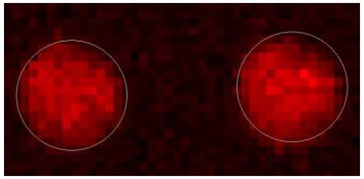

Grade III_CF 25539_2000143971

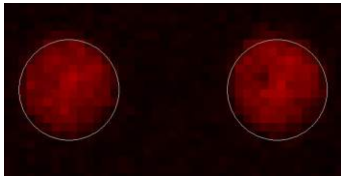

Grade III_CF 29446_2000144453

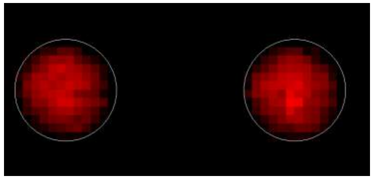

Grade III_CF 6980_2000154032

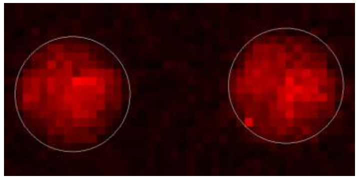

Grade III_CH 15130_2000143974

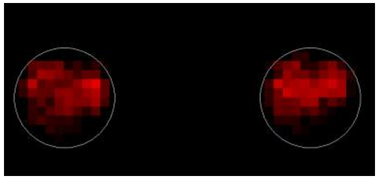

Grade III_CH 15138_2000143946

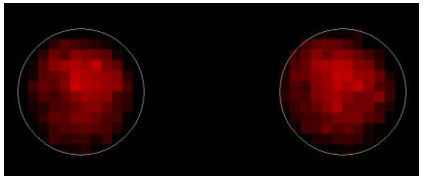

Grade III_CH 17244_2000144448

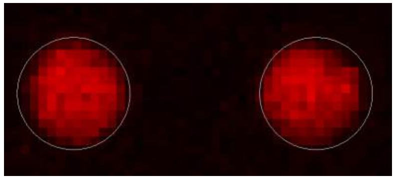

Grade III_CH 9010_2000143984

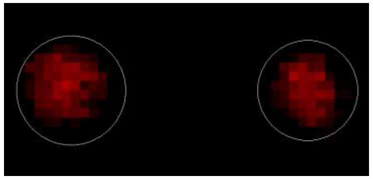

Grade III_CH 9043_2000144459

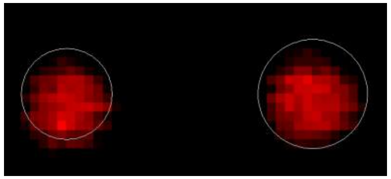

Grade III_CJ 1176_2000154027

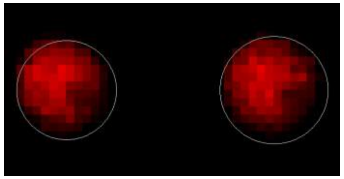

Grade III_CJ 14116_2000144450

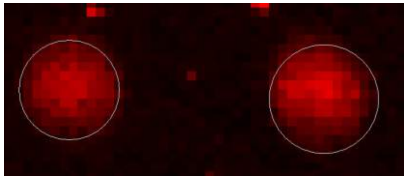

Grade III_CJ 16912_2000154006

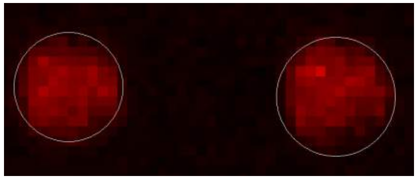

Grade III_CJ 20721_2000143947

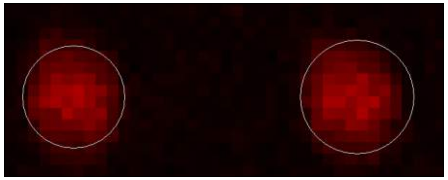

Grade III_CJ 7833_2000153939

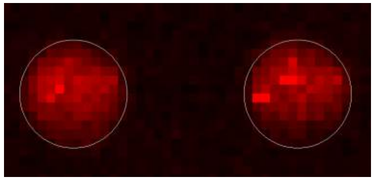

Grade III_CJ 9761_2000143973

Grade III

NM_138794.1 LYPLAL1

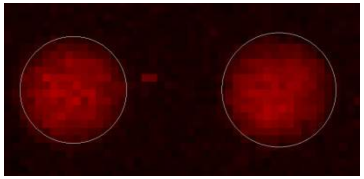
Control_H-02_2000153953

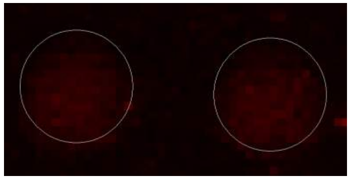
Control_H-03_2000153943

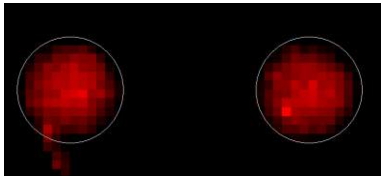
Control_H-19_2000154008

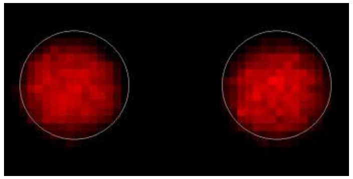
Control_H-23_2000154009

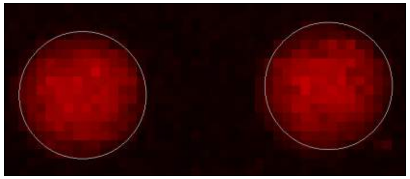
Control_H-25_2000153952

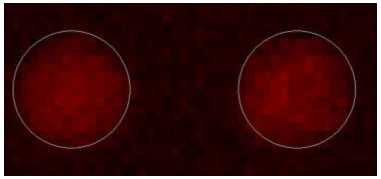
Control_H-35_2000153942

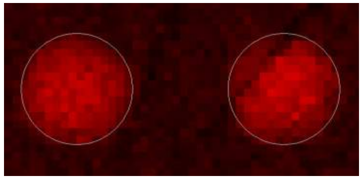
Control_H-41_2000154026

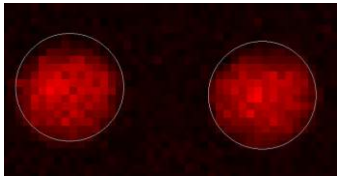
Control_H-58_2000153935

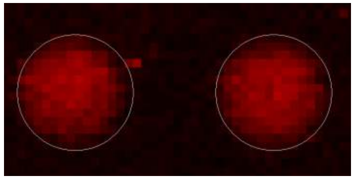
Control_H-59_2000154016

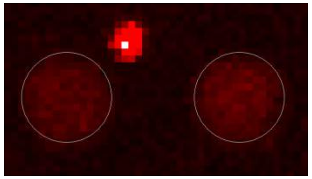
Control_HC 25_2000153932

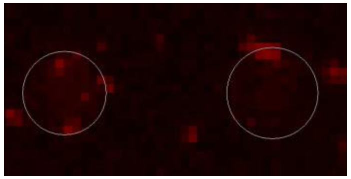
Control_HV-56_2000155735

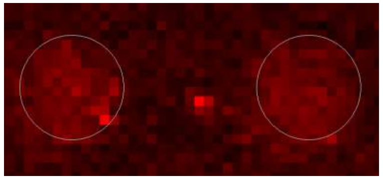
Control_HV-59_2000155740

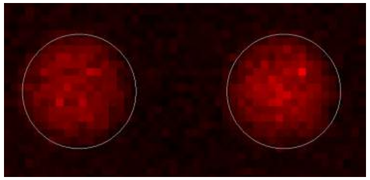
Control_HV-64_2000144456

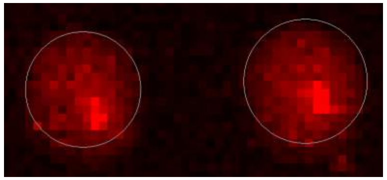
Control_HV-70_2000144457

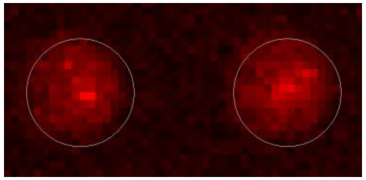
Control_HV-71_2000144458

Control

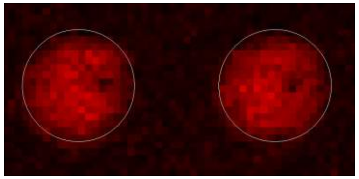
Grade III_CF 12839_2000143982

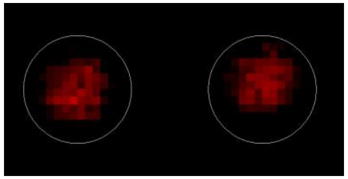
Grade III_CF 16216_2000144451

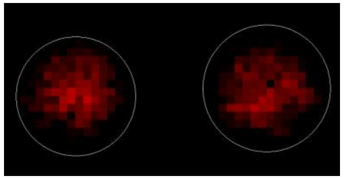
Grade III_CF 22483_2000144446

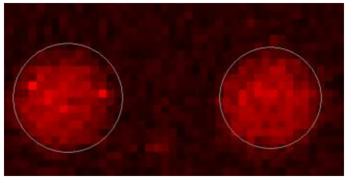
Grade III_CF 22807_2000154035

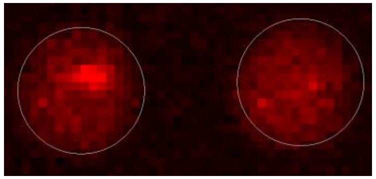
Grade III_CF 25539_2000143971

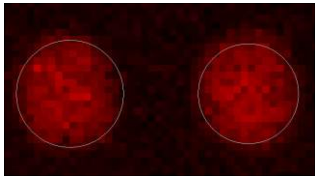
Grade III_CF 29446_2000144453

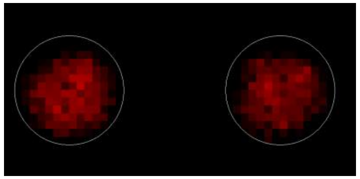
Grade III_CF 6980_2000154032

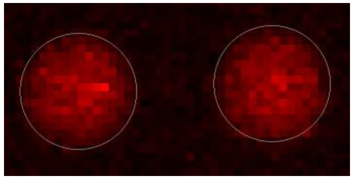
Grade III_CH 15130_2000143974

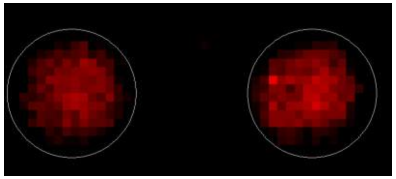
Grade III_CH 15138_2000143946

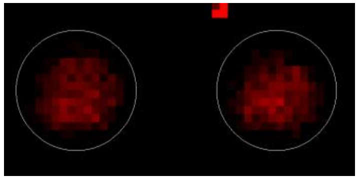
Grade III_CH 17244_2000144448

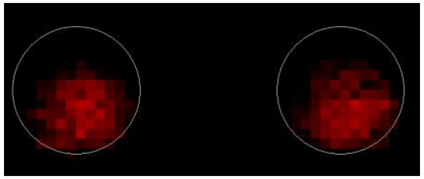
Grade III_CH 9010_2000143984

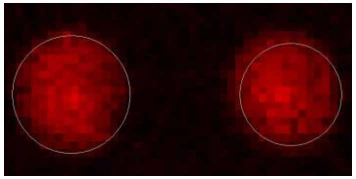
Grade III_CH 9043_2000144459

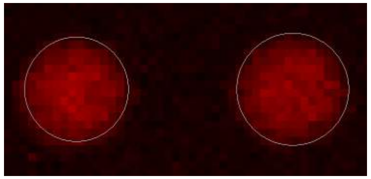
Grade III_CJ 1176_2000154027

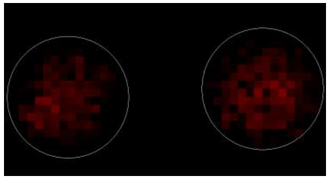
Grade III_CJ 14116_2000144450

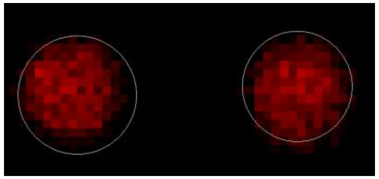
Grade III_CJ 16912_2000154006

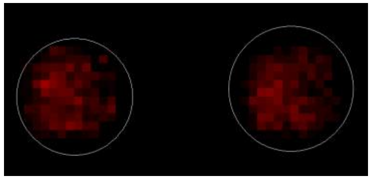
Grade III_CJ 20721_2000143947

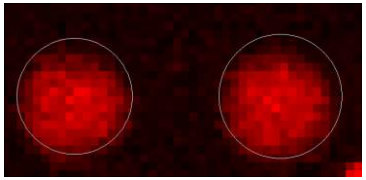
Grade III_CJ 7833_2000153939

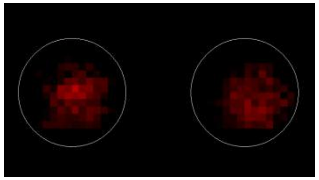
Grade III_CJ 9761_2000143973

Grade III

NM_015926.3 TEX264

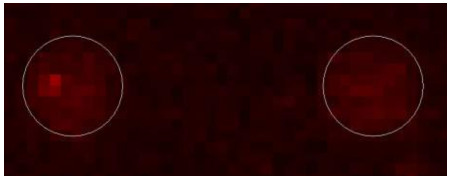
Control_H-02_2000153953

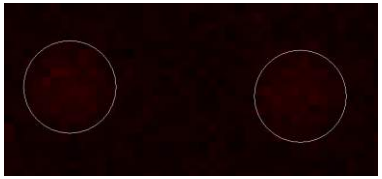
Control_H-03_2000153943

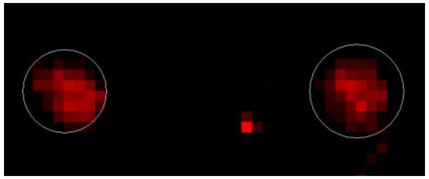
Control_H-19_2000154008

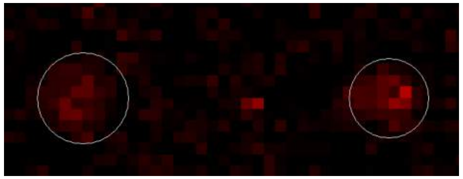
Control_H-23_2000154009

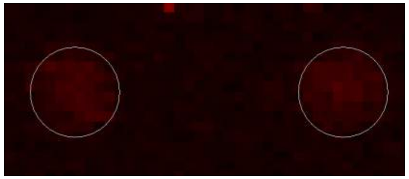
Control_H-25_2000153952

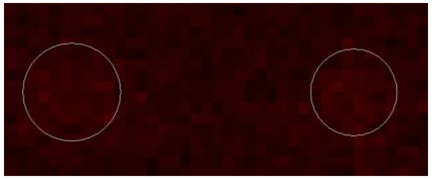
Control_H-35_2000153942

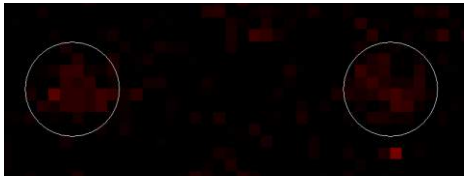
Control_H-41_2000154026

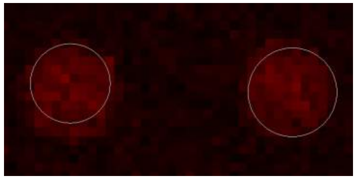
Control_H-58_2000153935

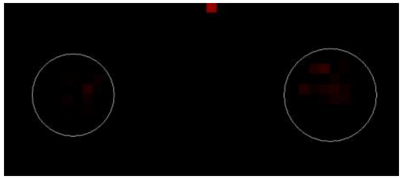
Control_H-59_2000154016

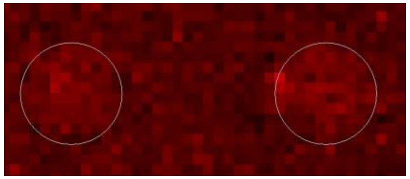
Control_HC 25_2000153932

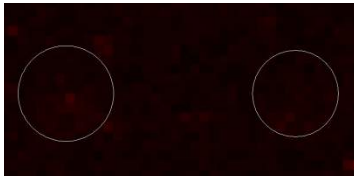
Control_HV-56_2000155735

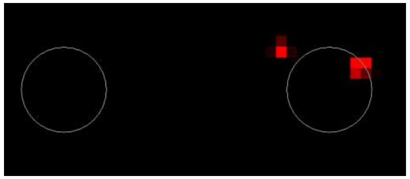
Control_HV-59_2000155740

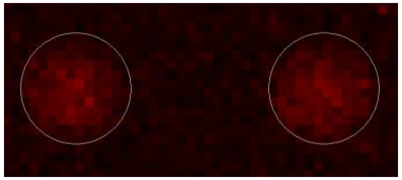
Control_HV-64_2000144456

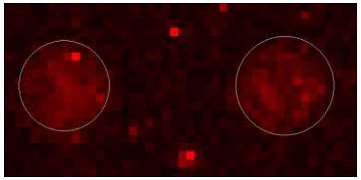
Control_HV-70_2000144457

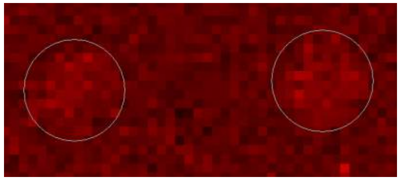
Control_HV-71_2000144458

Control

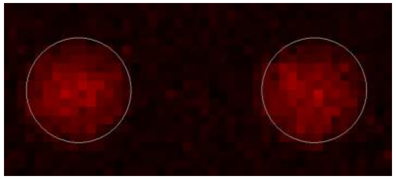

Grade III_CF 12839_2000143982

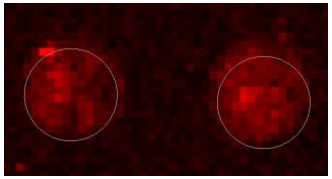

Grade III_CF 16216_2000144451

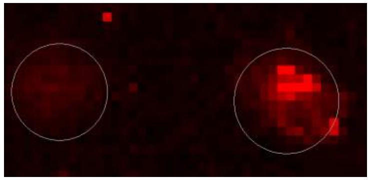

Grade III_CF 22483_2000144446

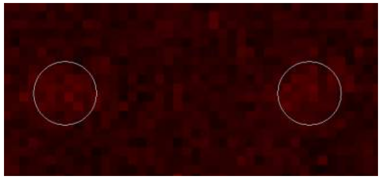

Grade III_CF 22807_2000154035

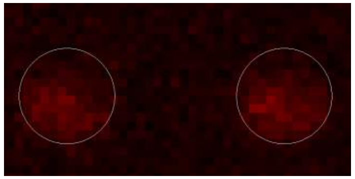

Grade III_CF 25539_2000143971

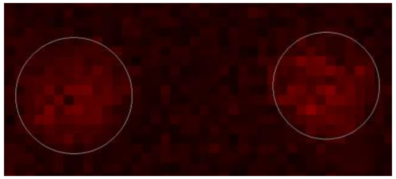

Grade III_CF 29446_2000144453

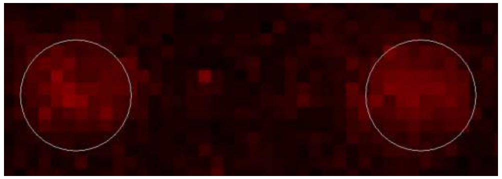

Grade III_CF 6980_2000154032

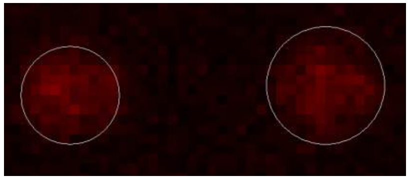

Grade III_CH 15130_2000143974

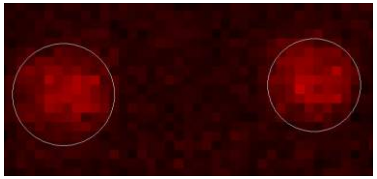

Grade III_CH 15138_2000143946

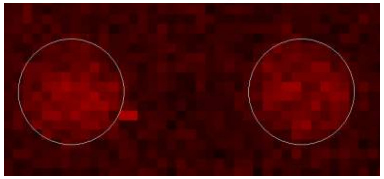

Grade III_CH 17244_2000144448

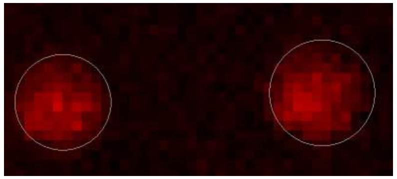

Grade III_CH 9010_2000143984

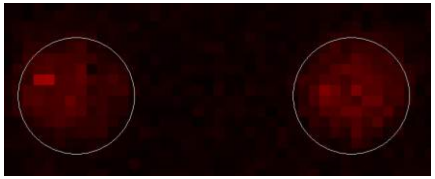

Grade III_CH 9043_2000144459

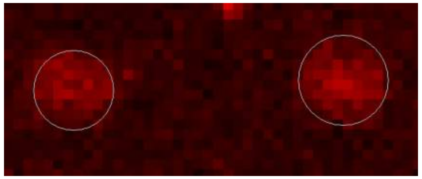

Grade III_CJ 1176_2000154027

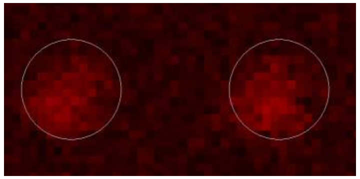

Grade III_CJ 14116_2000144450

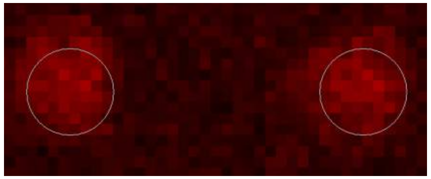

Grade III_CJ 16912_2000154006

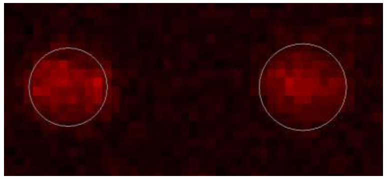

Grade III_CJ 20721_2000143947

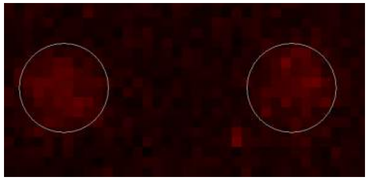

Grade III_CJ 7833_2000153939

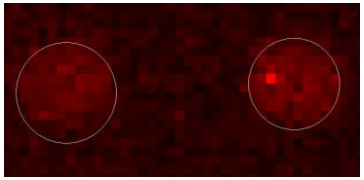

Grade III_CJ 9761_2000143973

Grade III

BC001241.1 ARHGAP17

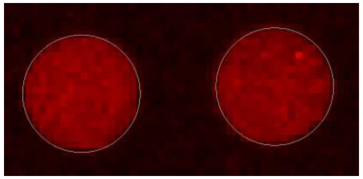
Control_H-02_2000153953

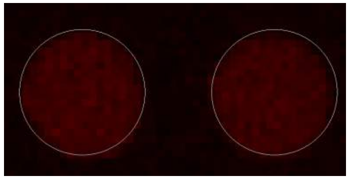
Control_H-03_2000153943

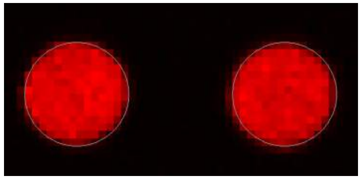
Control_H-19_2000154008

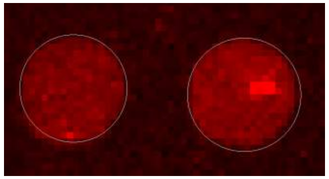
Control_H-23_2000154009

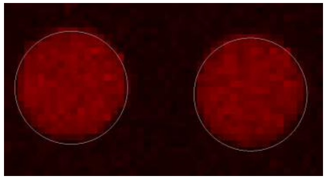
Control_H-25_2000153952

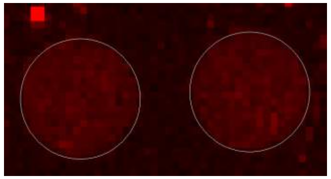
Control_H-35_2000153942

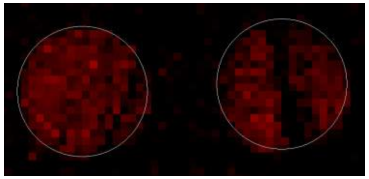
Control_H-41_2000154026

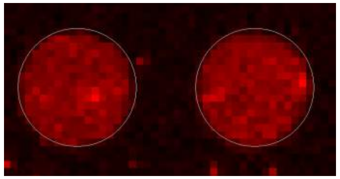
Control_H-58_2000153935

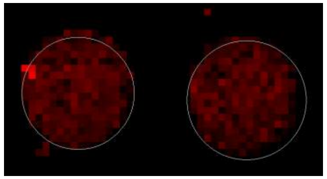
Control_H-59_2000154016

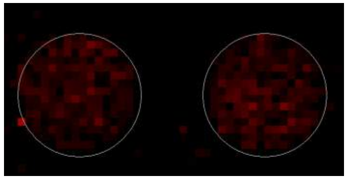
Control_HC 25_2000153932

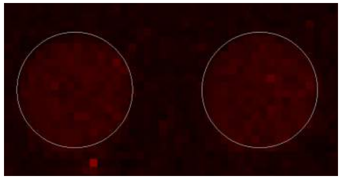
Control_HV-56_2000155735

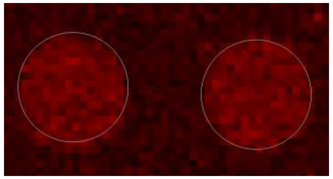
Control_HV-59_2000155740

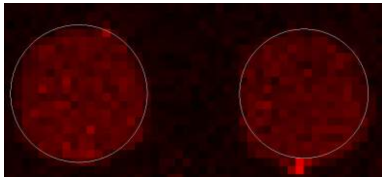
Control_HV-64_2000144456

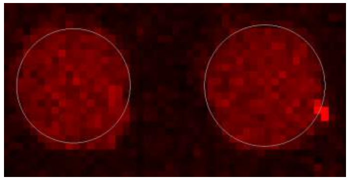
Control_HV-70_2000144457

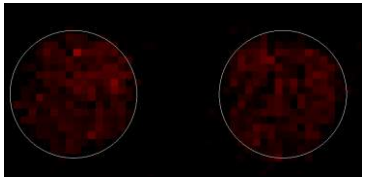
Control_HV-71_2000144458

Control

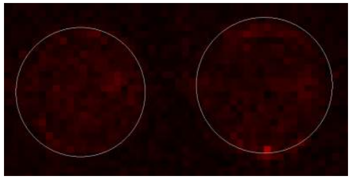
Grade III_CF 12839_2000143982

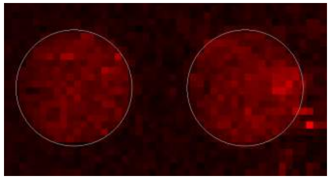
Grade III_CF 16216_2000144451

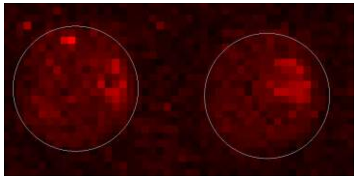
Grade III_CF 22483_2000144446

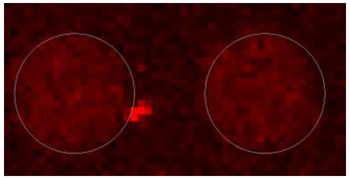
Grade III_CF 22807_2000154035

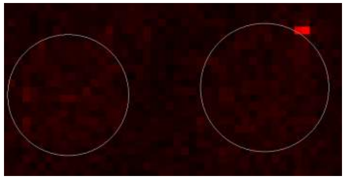
Grade III_CF 25539_2000143971

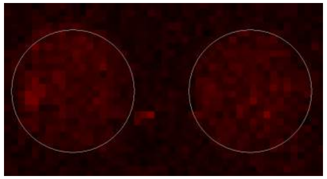
Grade III_CF 29446_2000144453

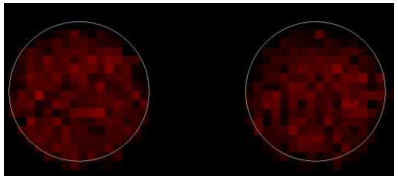
Grade III_CF 6980_2000154032

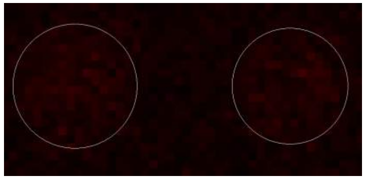
Grade III_CH 15130_2000143974

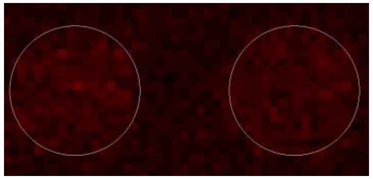
Grade III_CH 15138_2000143946

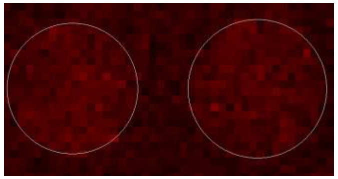
Grade III_CH 17244_2000144448

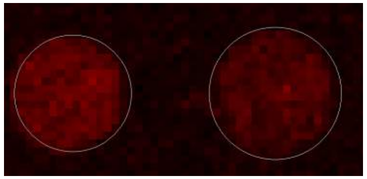
Grade III_CH 9010_2000143984

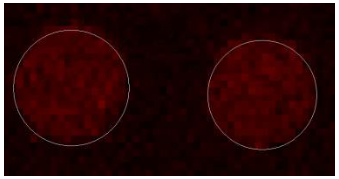
Grade III_CH 9043_2000144459

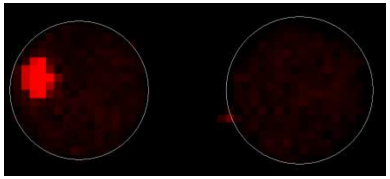
Grade III_CJ 1176_2000154027

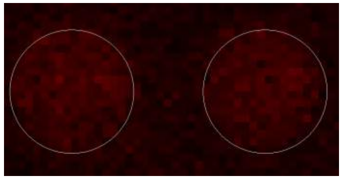
Grade III_CJ 14116_2000144450

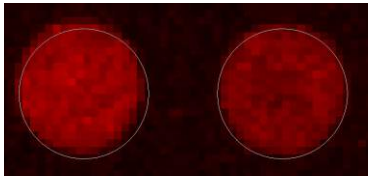
Grade III_CJ 16912_2000154006

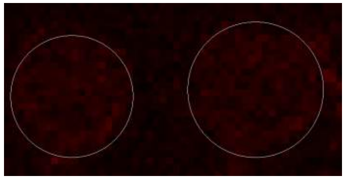
Grade III_CJ 20721_2000143947

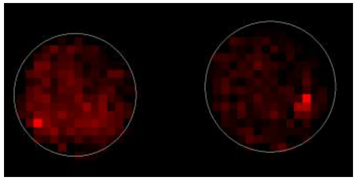
Grade III_CJ 7833_2000153939

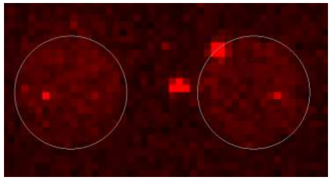
Grade III_CJ 9761_2000143973

Grade III

Control Vs Grade IV

NM_003099.3 SNX1

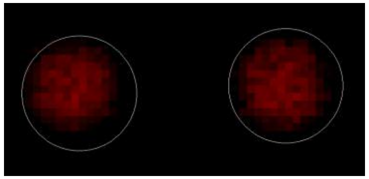

Control_H-02_2000153953

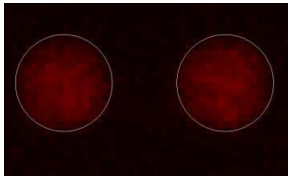

Control_H-03_2000153943

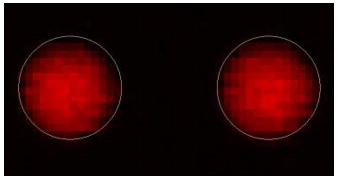

Control_H-19_2000154008

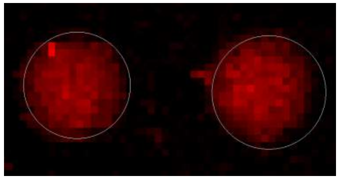

Control_H-23_2000154009

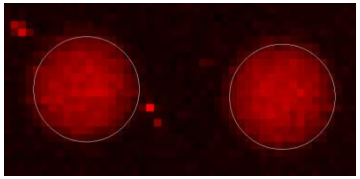

Control_H-25_2000153952

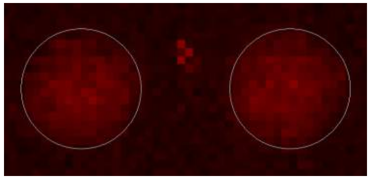

Control_H-35_2000153942

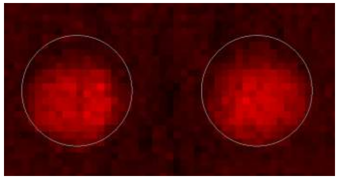

Control_H-41_2000154026

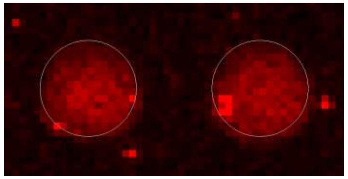

Control_H-58_2000153935

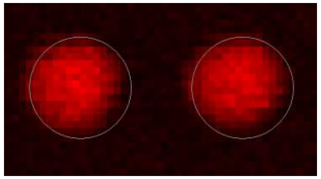

Control_H-59_2000154016

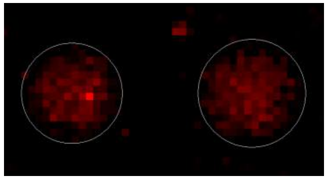

Control_HC 25_2000153932

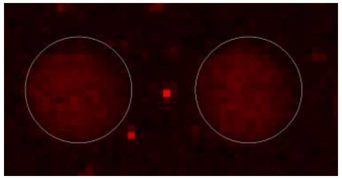

Control_HV-56_2000155735

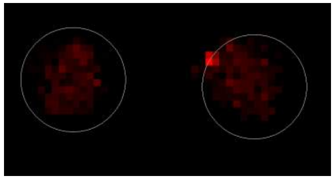

Control_HV-59_2000155740

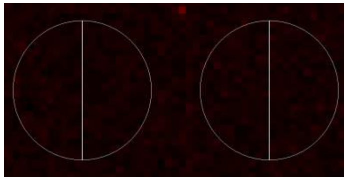

Control_HV-64_2000144456

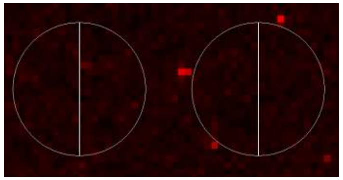

Control_HV-70_2000144457

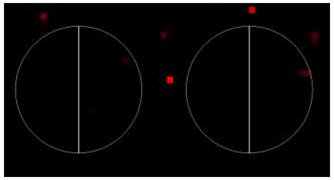

Control_HV-71_2000144458

Control

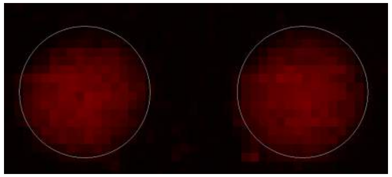
Grade IV_CF 7916_2000154021

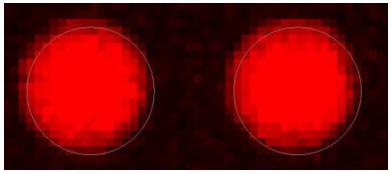
Grade IV_CJ 11291_2000154025

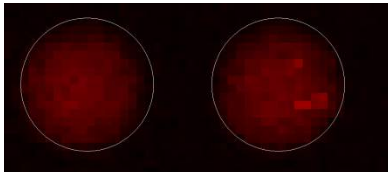
Grade IV_CJ 2041_2000154019

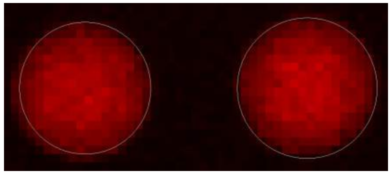
Grade IV_CJ 1519_2000153951

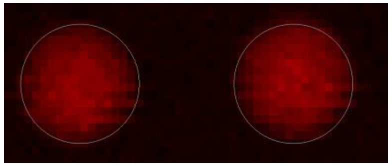
Grade IV_CF 7522_2000154010

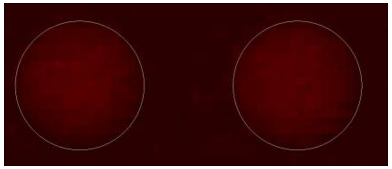
Grade IV_CH 9002_2000153954

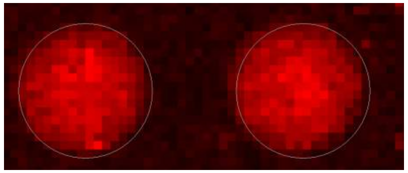
Grade IV_CF 11518_2000153942

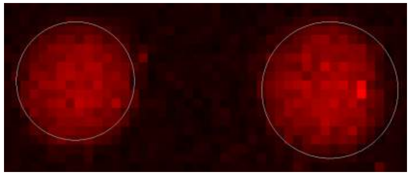
Grade IV_CH 3969_2000153950

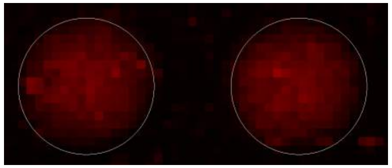
Grade IV_CH 4996_2000153933

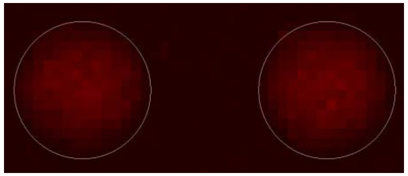
Grade IV_CF 12166_2000153934

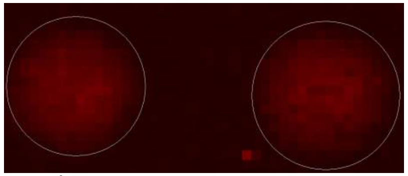
Grade IV_CH 14820_2000153796

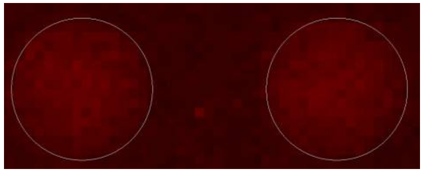
Grade IV_CJ 32705_2000153797

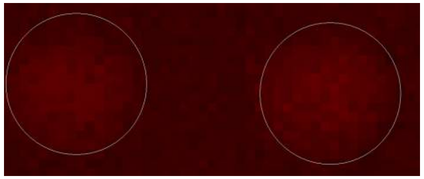
Grade IV_CH 29738_2000153798

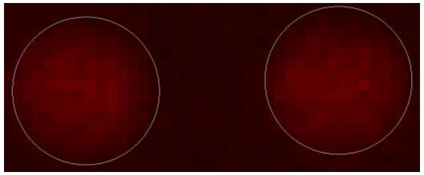
Grade IV_CJ 30014_2000153805

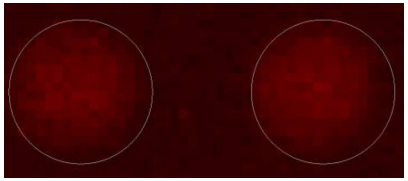
Grade IV_CH 18016_2000153791

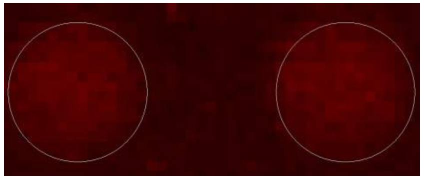
Grade IV_CH 30203_2000153793

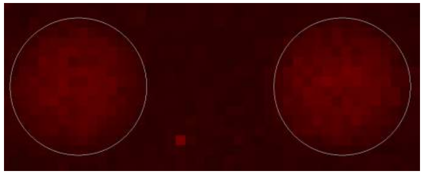
Grade IV_CH 28569_2000153799

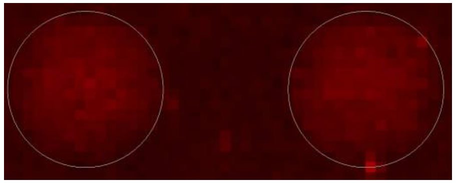
Grade IV_CJ 6254_2000153800

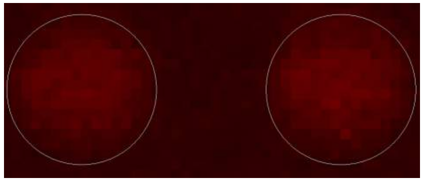
Grade IV_CH 22618_2000153782

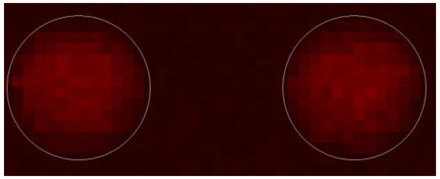
Grade IV_CJ 13655_2000153783

Grade IV

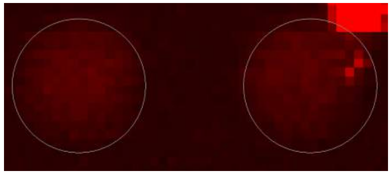
Grade IV_CJ 18704_2000153784

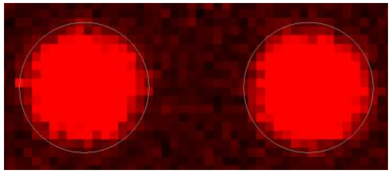
Grade IV_CH 21783_2000153785

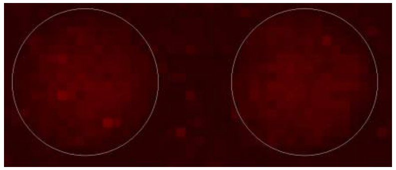
Grade IV_CF 27891_2000153776

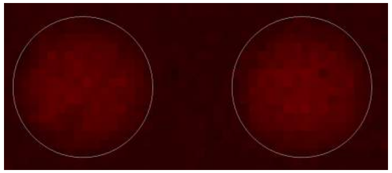
Grade IV_CH 8498_2000153777

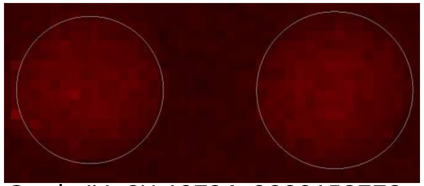
Grade IV_CH 12724_2000153778

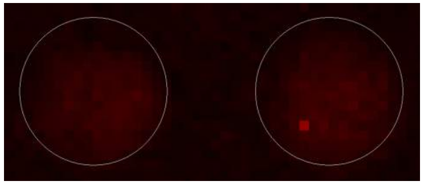
Grade IV_CF 11940_2000153779

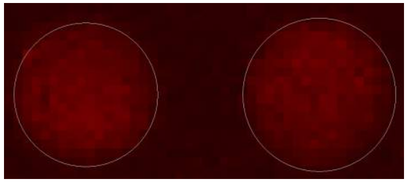
Grade IV_CJ 22776_2000153756

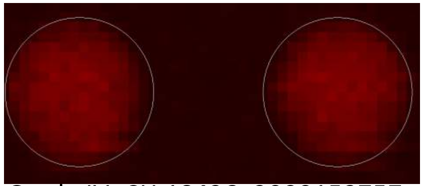
Grade IV_CH 18426_2000153757

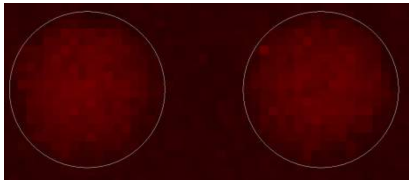
Grade IV_CH 6643_2000153758

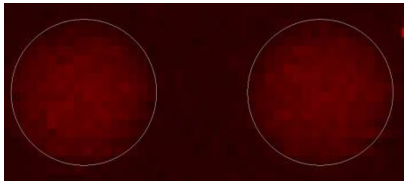
Grade IV_CH 29697_2000153759

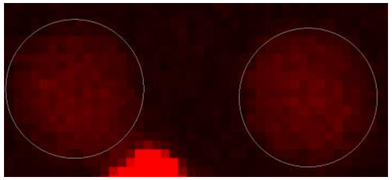
Grade IV_CJ 12441_2000153760

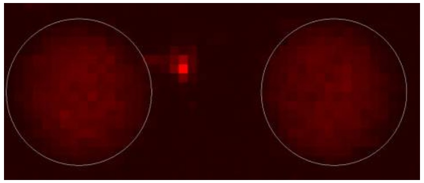
Grade IV_CH 5261_2000155722

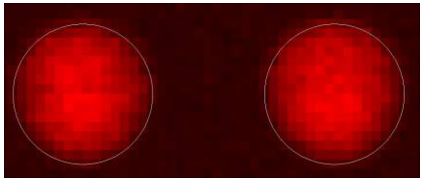
Grade IV_CF 19257 _2000155723

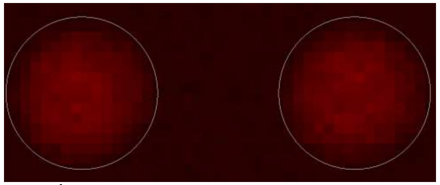
Grade IV_CJ 10048_2000155725

Grade IV

BC018747.1 IGHG1

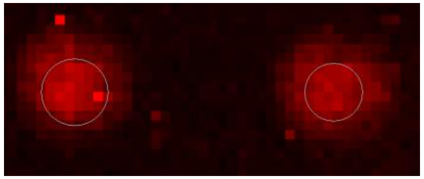
Control_H-02_2000153953

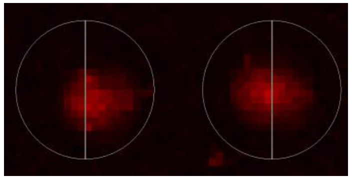
Control_H-03_2000153943

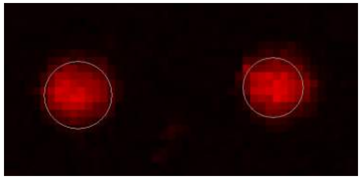
Control_H-19_2000154008

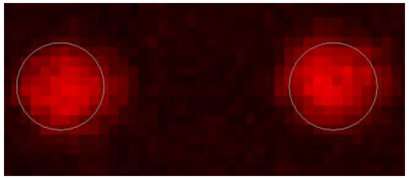
Control_H-23_2000154009

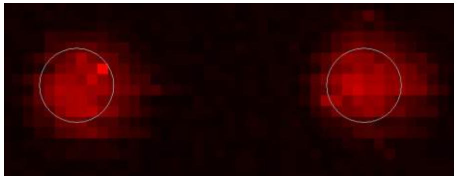
Control_H-25_2000153952

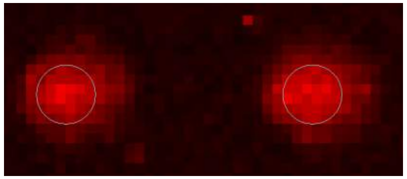
Control_H-35_2000153942

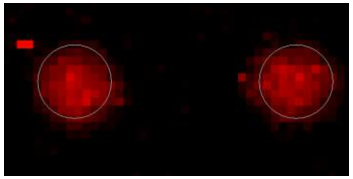
Control_H-41_2000154026

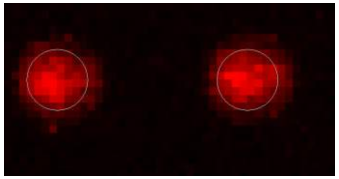
Control_H-58_2000153935

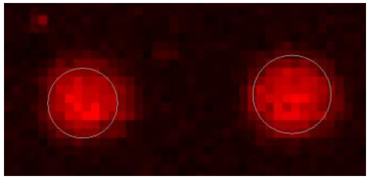
Control_H-59_2000154016

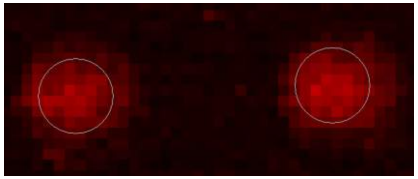
Control_HC 25_2000153932

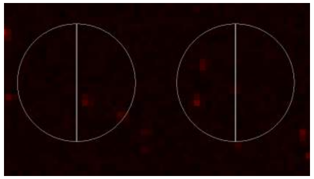
Control_HV-56_2000155735

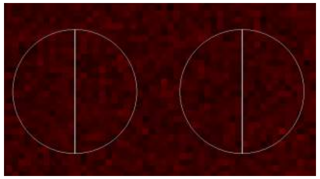
Control_HV-59_2000155740

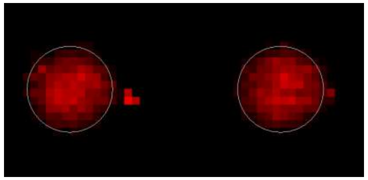
Control_HV-64_2000144456

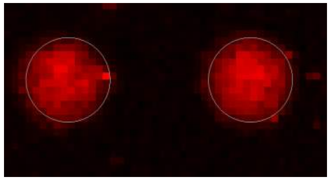
Control_HV-70_2000144457

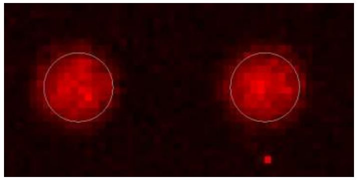
Control_HV-71_2000144458

Control

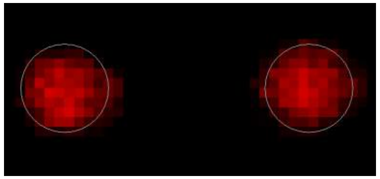
Grade IV_CF 7916_2000154021

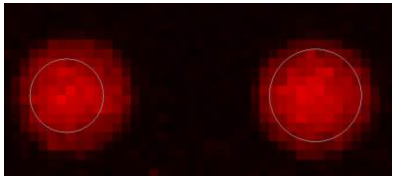
Grade IV_CJ 11291_2000154025

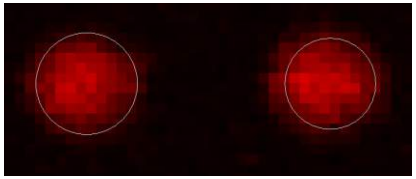
Grade IV_CJ 2041_2000154019

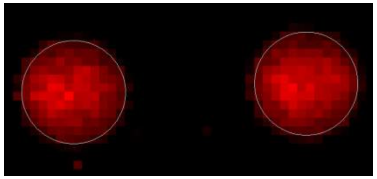
Grade IV_CJ 1519_2000153951

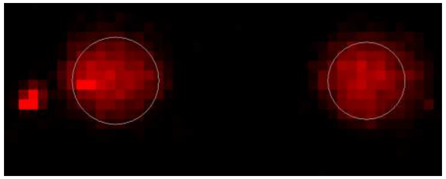
Grade IV_CF 7522_2000154010

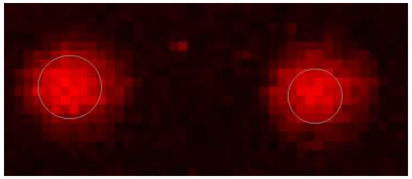
Grade IV_CH 9002_2000153954

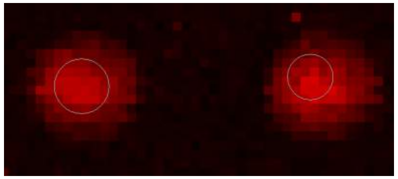
Grade IV_CF 11518_2000153942

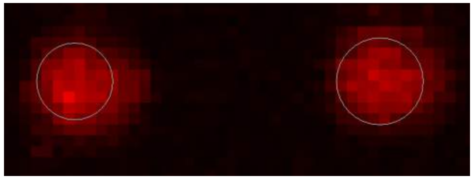
Grade IV_CH 3969_2000153950

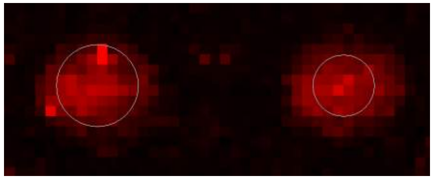
Grade IV_CH 4996_2000153933

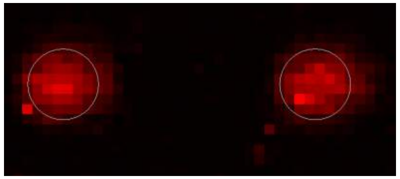
Grade IV_CF 12166_2000153934

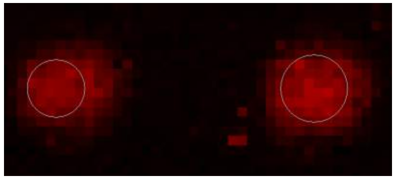
Grade IV_CH 14820_2000153796

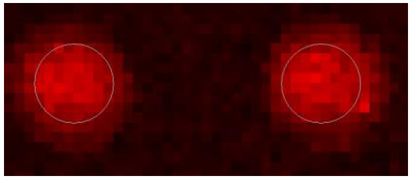
Grade IV_CJ 32705_2000153797

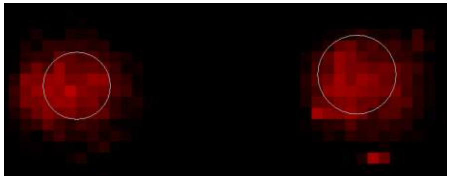
Grade IV_CH 29738_2000153798

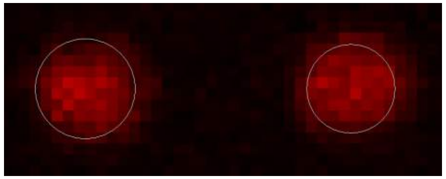
Grade IV_CJ 30014_2000153805

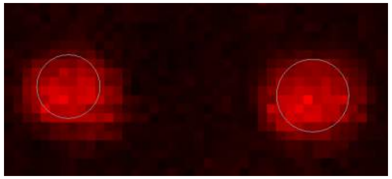
Grade IV_CH 18016_2000153791

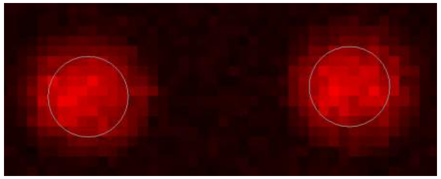
Grade IV_CH 30203_2000153793

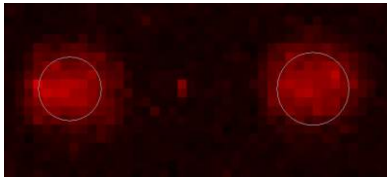
Grade IV_CH 28569_2000153799

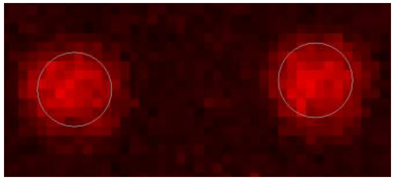
Grade IV_CJ 6254_2000153800

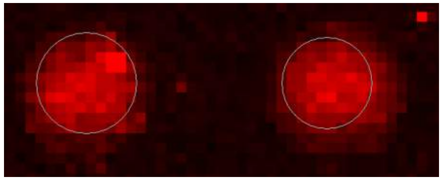
Grade IV_CH 22618_2000153782

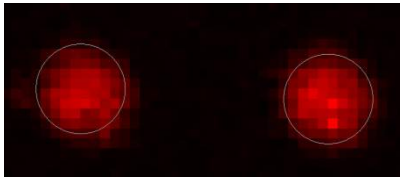
Grade IV_CJ 13655_2000153783

Grade IV

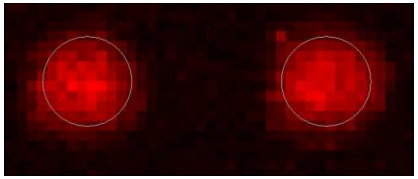
Grade IV_CJ 18704_2000153784

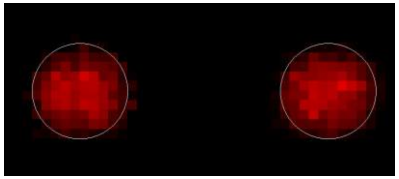
Grade IV_CH 21783_2000153785

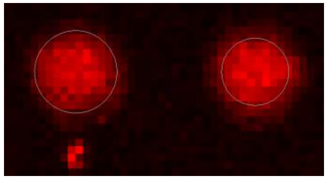
Grade IV_CF 27891_2000153776

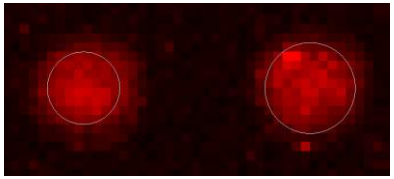
Grade IV_CH 8498_2000153777

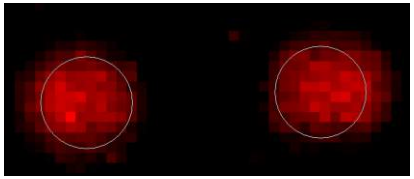
Grade IV_CH 12724_2000153778

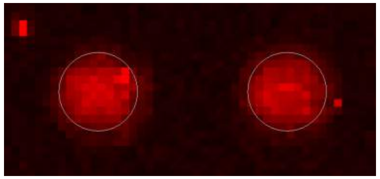
Grade IV_CF 11940_2000153779

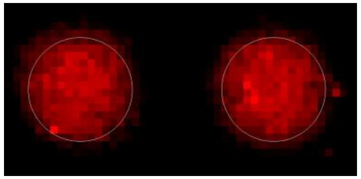
Grade IV_CJ 22776_2000153756

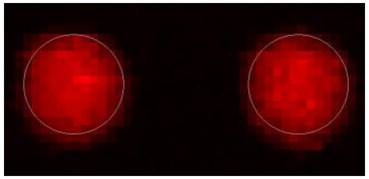
Grade IV_CH 18426_2000153757

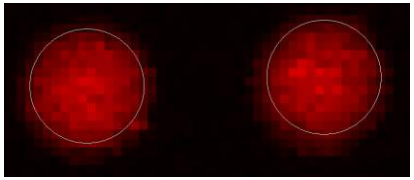
Grade IV_CH 6643_2000153758

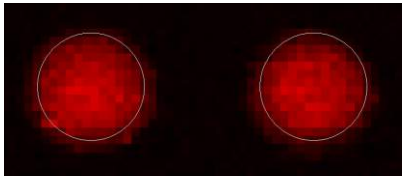
Grade IV_CH 29697_2000153759

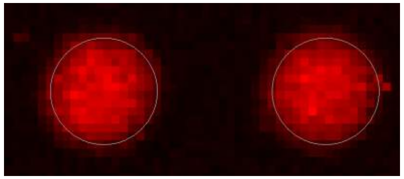
Grade IV_CJ 12441_2000153760

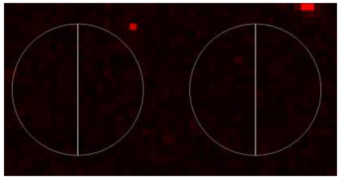
Grade IV_CH 5261_2000155722

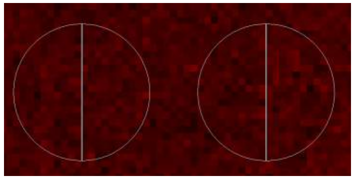
Grade IV_CF 19257 _2000155723

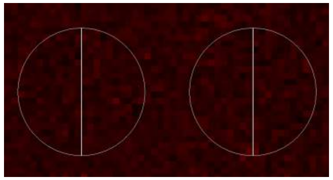
Grade IV_CJ 10048_2000155725

Grade IV

BC009561.1 C11orf74

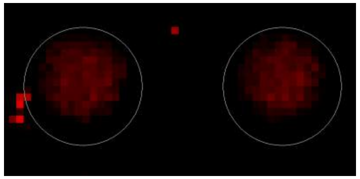
Control_H-02_2000153953

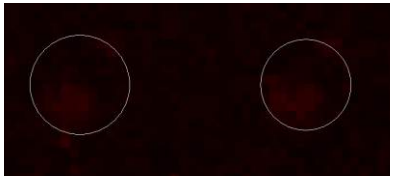
Control_H-03_2000153943

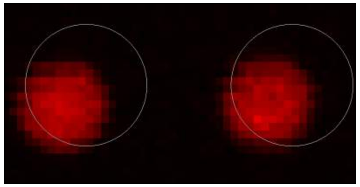
Control_H-19_2000154008

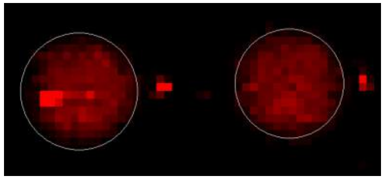
Control_H-23_2000154009

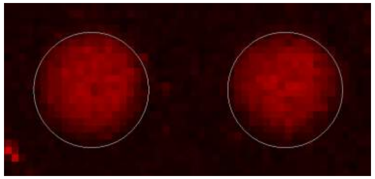
Control_H-25_2000153952

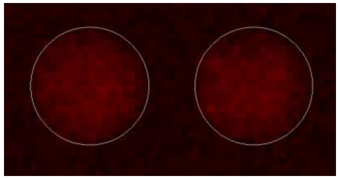
Control_H-35_2000153942

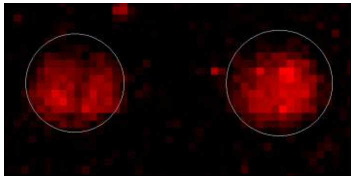
Control_H-41_2000154026

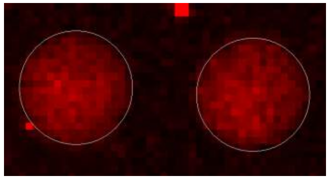
Control_H-58_2000153935

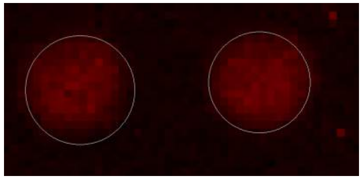
Control_H-59_2000154016

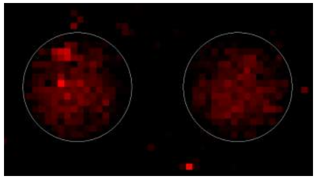
Control_HC 25_2000153932

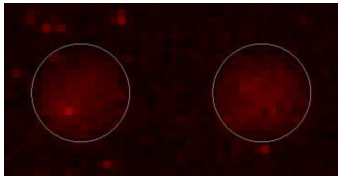
Control_HV-56_2000155735

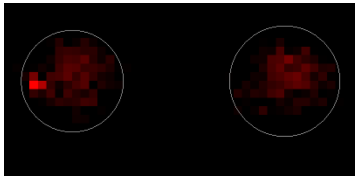
Control_HV-59_2000155740

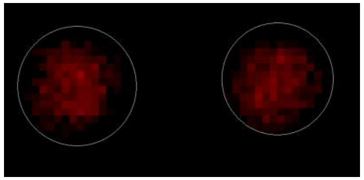
Control_HV-64_2000144456

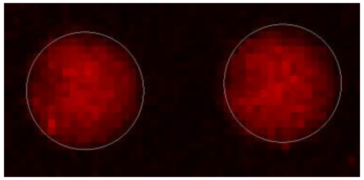
Control_HV-70_2000144457

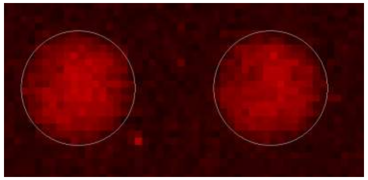
Control_HV-71_2000144458

Control

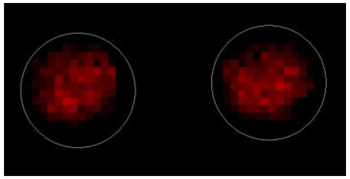

Grade IV_CF 7916_2000154021

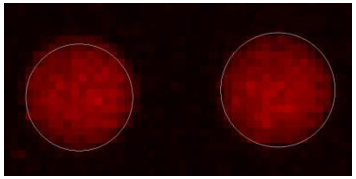

Grade IV_CJ 11291_2000154025

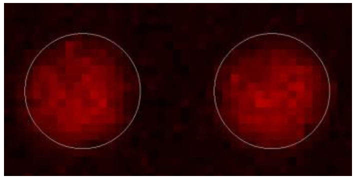

Grade IV_CJ 2041_2000154019

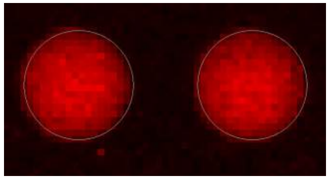

Grade IV_CJ 1519_2000153951

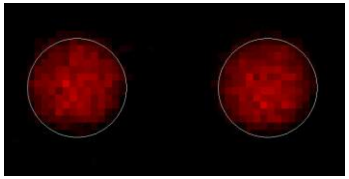

Grade IV_CF 7522_2000154010

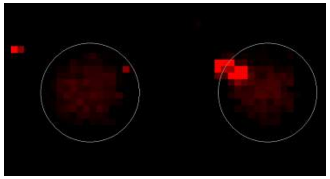

Grade IV_CH 9002_2000153954

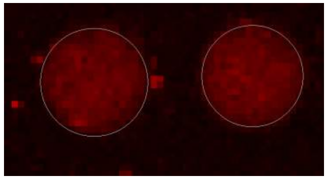

Grade IV_CF 11518_2000153942

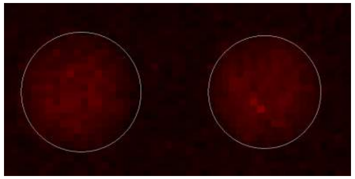

Grade IV_CH 3969_2000153950

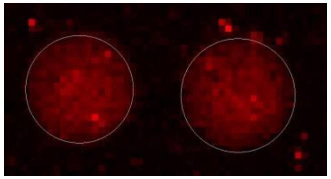

Grade IV_CH 4996_2000153933

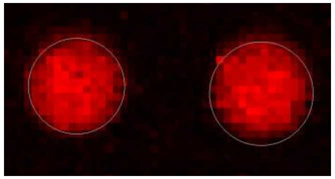

Grade IV_CF 12166_2000153934

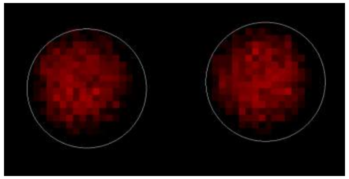

Grade IV_CH 14820_2000153796

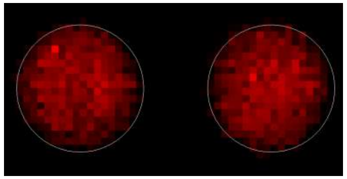

Grade IV_CJ 32705_2000153797

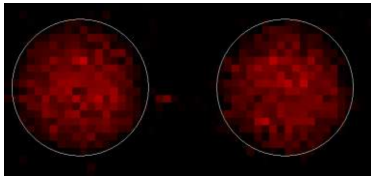

Grade IV_CH 29738_2000153798

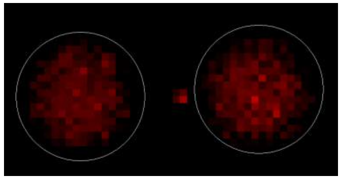

Grade IV_CJ 30014_2000153805

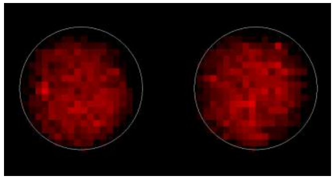

Grade IV_CH 18016_2000153791

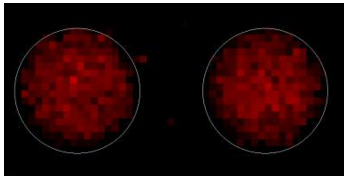

Grade IV_CH 30203_2000153793

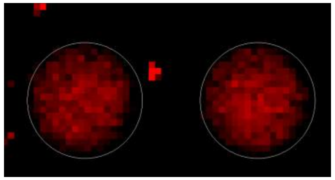

Grade IV_CH 28569_2000153799

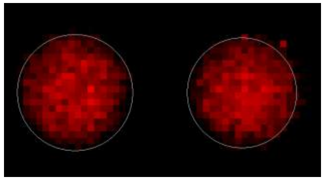

Grade IV_CJ 6254_2000153800

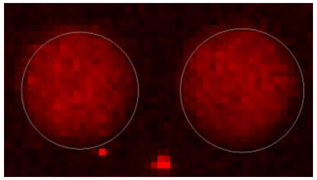

Grade IV_CH 22618_2000153782

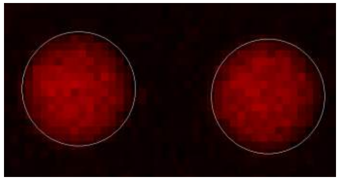

Grade IV_CJ 13655_2000153783

Grade IV

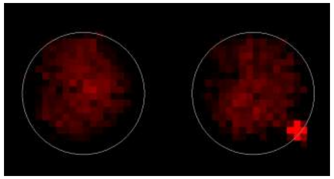

Grade IV_CJ 18704_2000153784

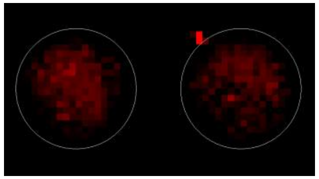

Grade IV_CH 21783_2000153785

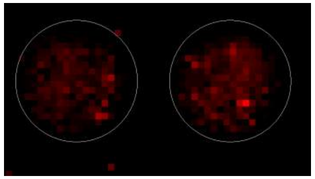

Grade IV_CF 27891_2000153776

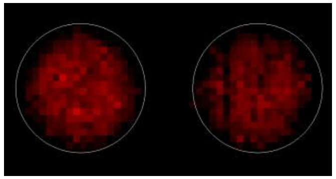

Grade IV_CH 8498_2000153777

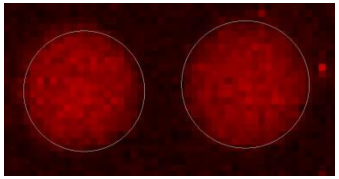

Grade IV_CH 12724_2000153778

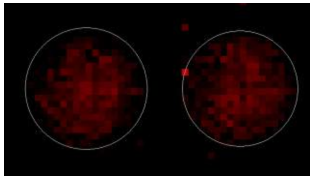

Grade IV_CF 11940_2000153779

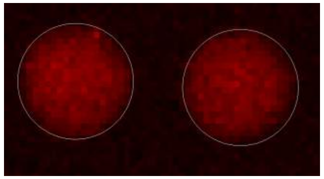

Grade IV_CJ 22776_2000153756

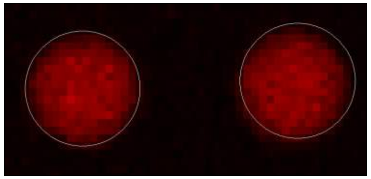

Grade IV_CH 18426_2000153757

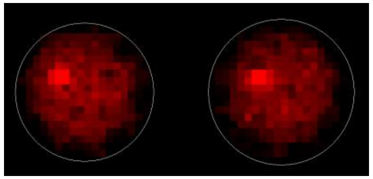

Grade IV_CH 6643_2000153758

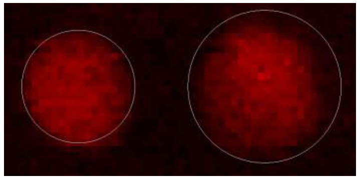

Grade IV_CH 29697_2000153759

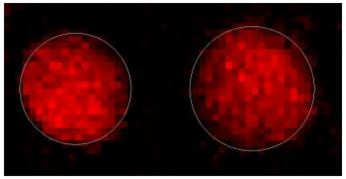

Grade IV_CJ 12441_2000153760

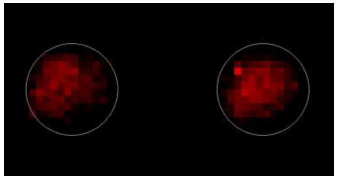

Grade IV_CH 5261_2000155722

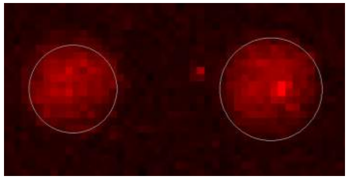

Grade IV_CF 19257 _2000155723

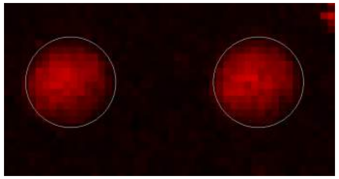

Grade IV_CJ 10048_2000155725

Grade IV

BC068569.1 ZHX3

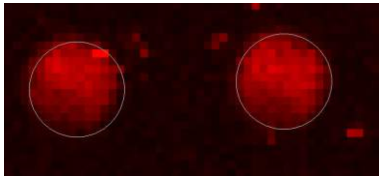
Control_H-02_2000153953

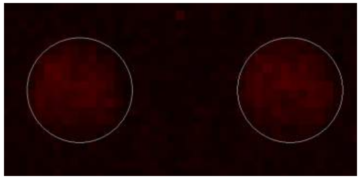
Control_H-03_2000153943

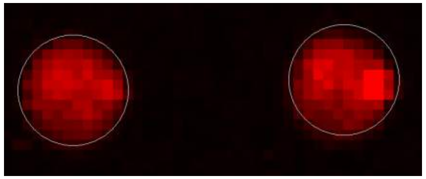
Control_H-19_2000154008

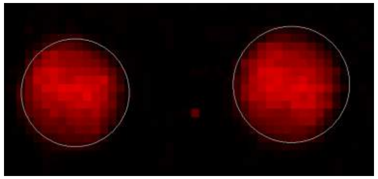
Control_H-23_2000154009

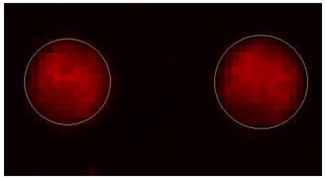
Control_H-25_2000153952

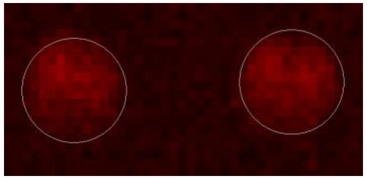
Control_H-35_2000153942

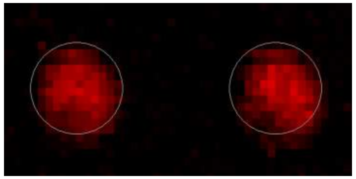
Control_H-41_2000154026

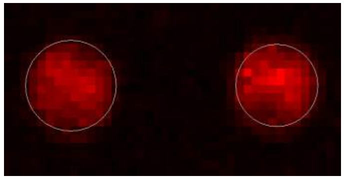
Control_H-58_2000153935

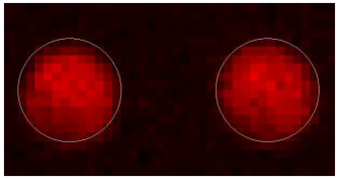
Control_H-59_2000154016

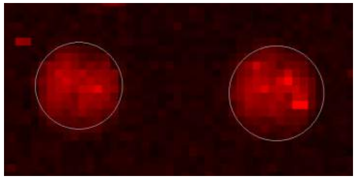
Control_HC 25_2000153932

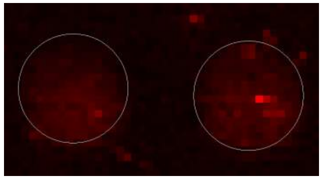
Control_HV-56_2000155735

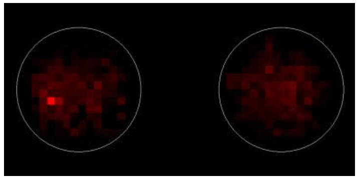
Control_HV-59_2000155740

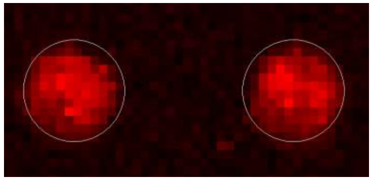
Control_HV-64_2000144456

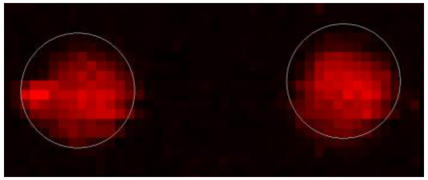
Control_HV-70_2000144457

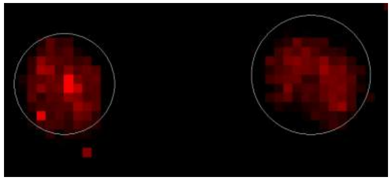
Control_HV-71_2000144458

Control

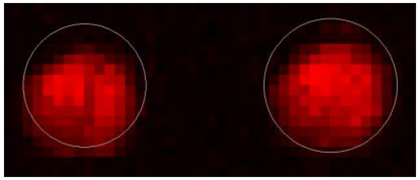
Grade IV_CF 7916_2000154021

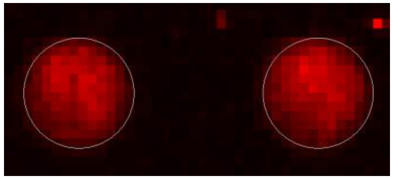
Grade IV_CJ 11291_2000154025

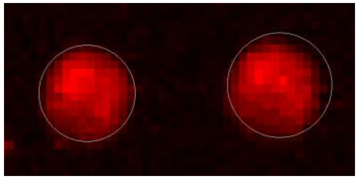
Grade IV_CJ 2041_2000154019

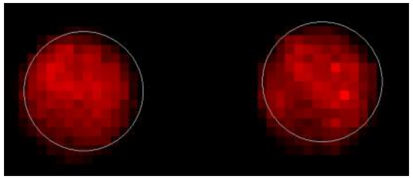
Grade IV_CJ 1519_2000153951

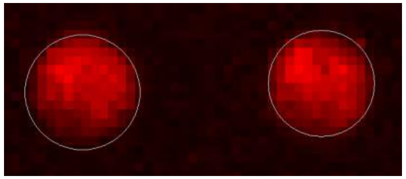
Grade IV_CF 7522_2000154010

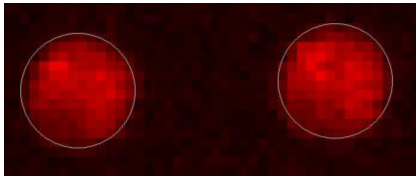
Grade IV_CH 9002_2000153954

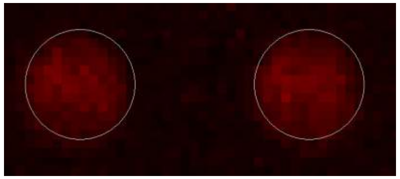
Grade IV_CF 11518_2000153942

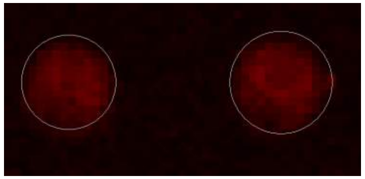
Grade IV_CH 3969_2000153950

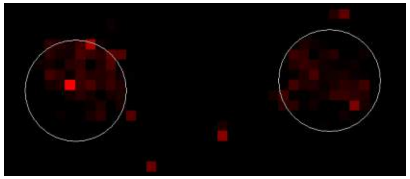
Grade IV_CH 4996_2000153933

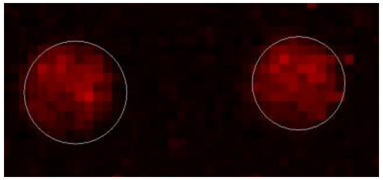
Grade IV_CF 12166_2000153934

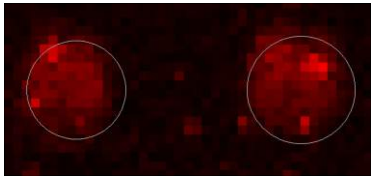
Grade IV_CH 14820_2000153796

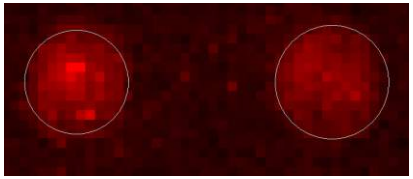
Grade IV_CJ 32705_2000153797

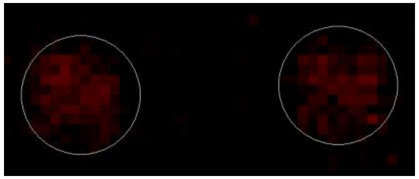
Grade IV_CH 29738_2000153798

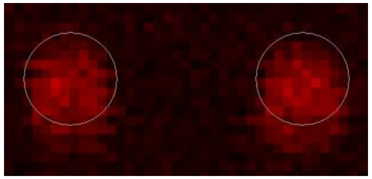
Grade IV_CJ 30014_2000153805

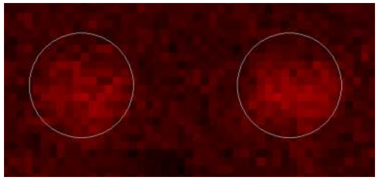
Grade IV_CH 18016_2000153791

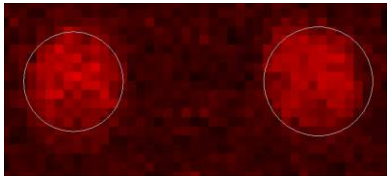
Grade IV_CH 30203_2000153793

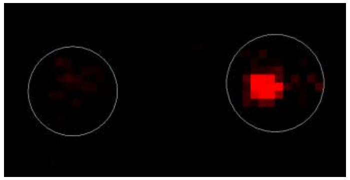
Grade IV_CH 28569_2000153799

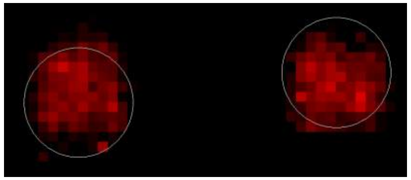
Grade IV_CJ 6254_2000153800

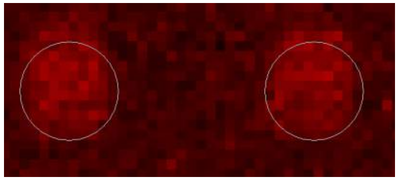
Grade IV_CH 22618_2000153782

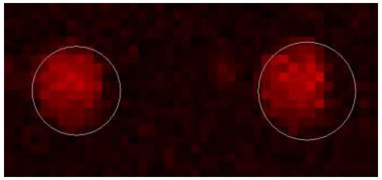
Grade IV_CJ 13655_2000153783

Grade IV

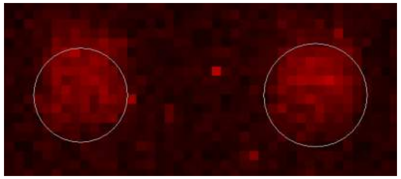

Grade IV_CJ 18704_2000153784

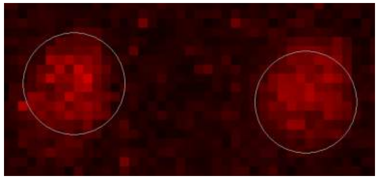

Grade IV_CH 21783_2000153785

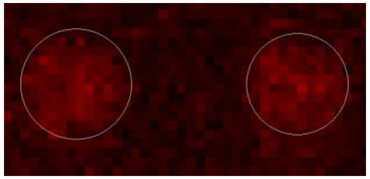

Grade IV_CF 27891_2000153776

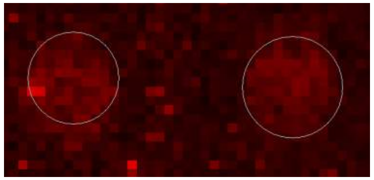

Grade IV_CH 8498_2000153777

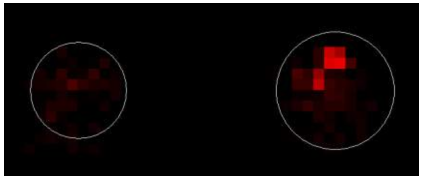

Grade IV_CH 12724_2000153778

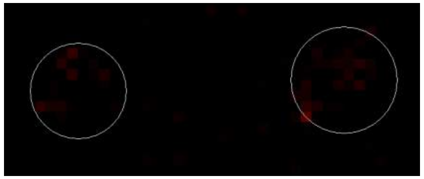

Grade IV_CF 11940_2000153779

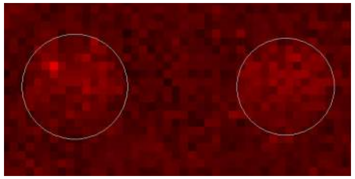

Grade IV_CJ 22776_2000153756

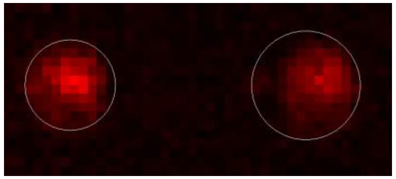

Grade IV_CH 18426_2000153757

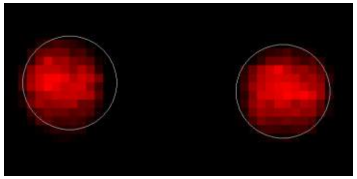

Grade IV_CH 6643_2000153758

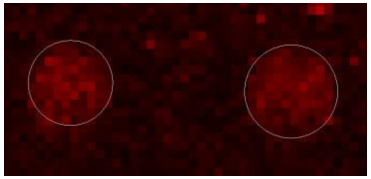

Grade IV_CH 29697_2000153759

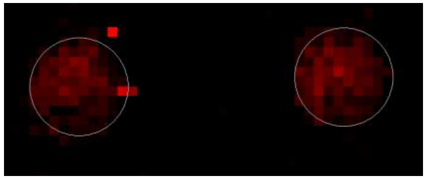

Grade IV_CJ 12441_2000153760

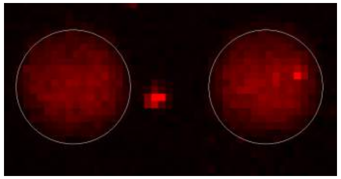

Grade IV_CH 5261_2000155722

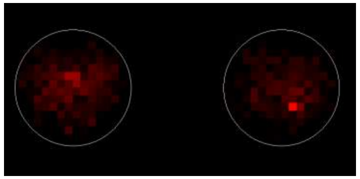

Grade IV_CF 19257 _2000155723

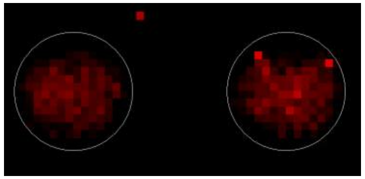

Grade IV_CJ 10048_2000155725

Grade IV

BC121798 EYA1

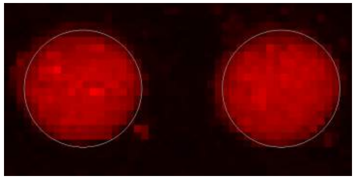

Control_H-02_2000153953

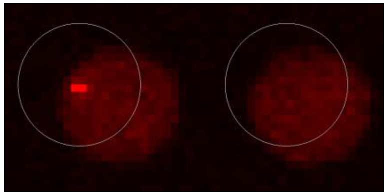

Control_H-03_2000153943

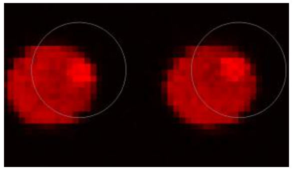

Control_H-19_2000154008

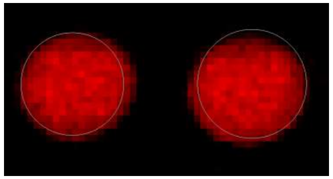

Control_H-23_2000154009

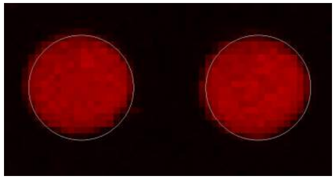

Control_H-25_2000153952

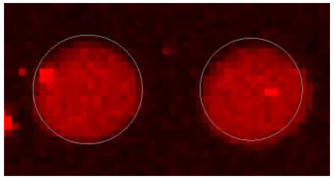

Control_H-35_2000153942

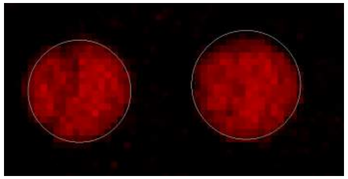

Control_H-41_2000154026

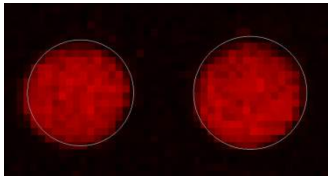

Control_H-58_2000153935

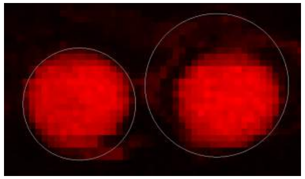

Control_H-59_2000154016

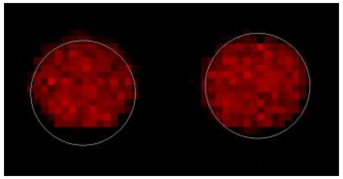

Control_HC 25_2000153932

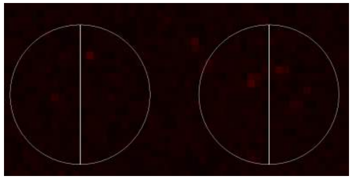

Control_HV-56_2000155735

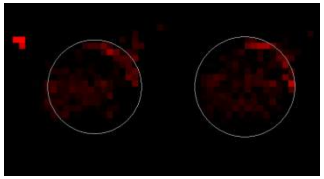

Control_HV-59_2000155740

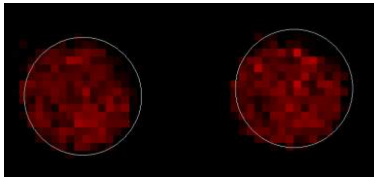

Control_HV-64_2000144456

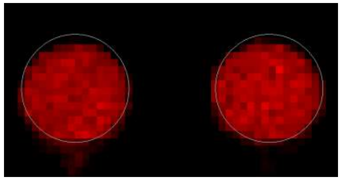

Control_HV-70_2000144457

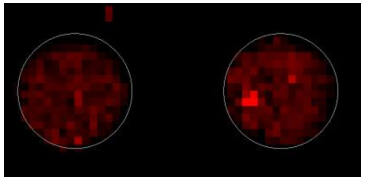

Control_HV-71_2000144458

Control

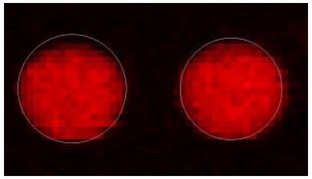

Grade IV_CF 7916_2000154021

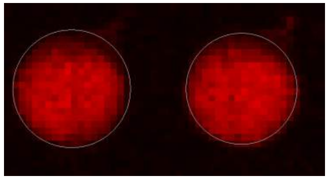

Grade IV_CJ 11291_2000154025

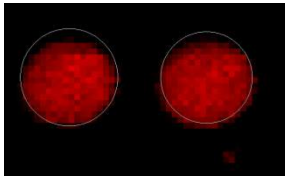

Grade IV_CJ 2041_2000154019

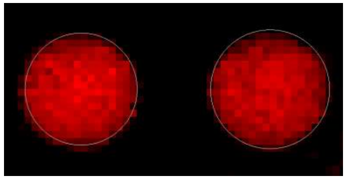

Grade IV_CJ 1519_2000153951

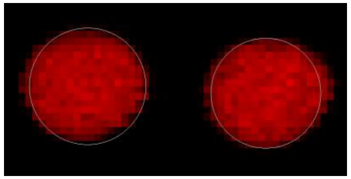

Grade IV_CF 7522_2000154010

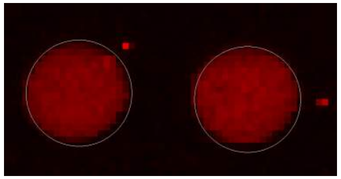

Grade IV_CH 9002_2000153954

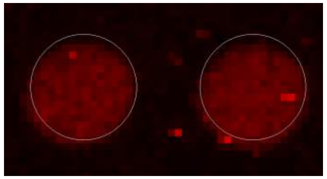

Grade IV_CF 11518_2000153942

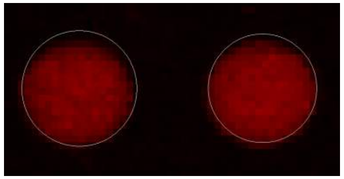

Grade IV_CH 3969_2000153950

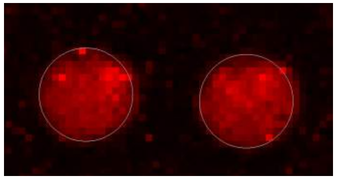

Grade IV_CH 4996_2000153933

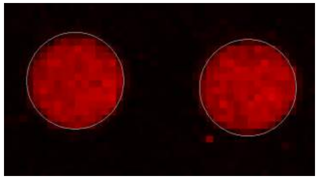

Grade IV_CF 12166_2000153934

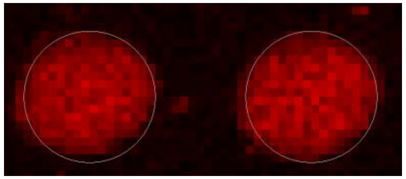

Grade IV_CH 14820_2000153796

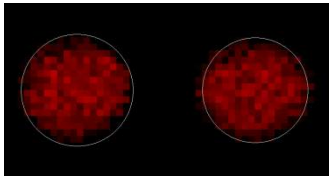

Grade IV_CJ 32705_2000153797

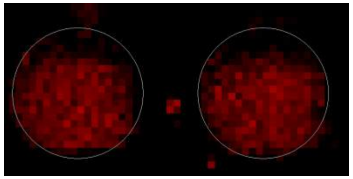

Grade IV_CH 29738_2000153798

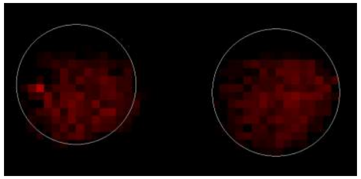

Grade IV_CJ 30014_2000153805

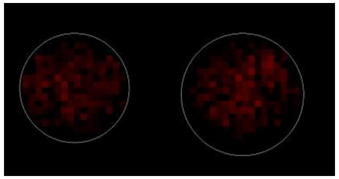

Grade IV_CH 18016_2000153791

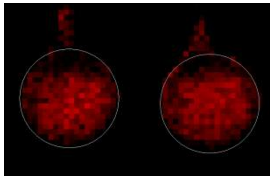

Grade IV_CH 30203_2000153793

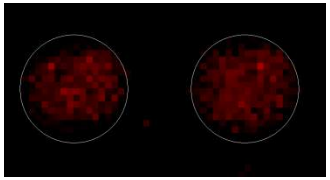

Grade IV_CH 28569_2000153799

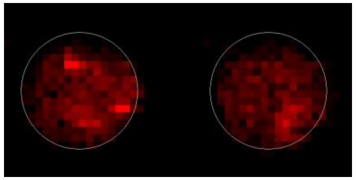

Grade IV_CJ 6254_2000153800

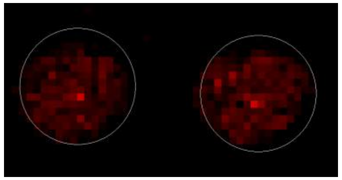

Grade IV_CH 22618_2000153782

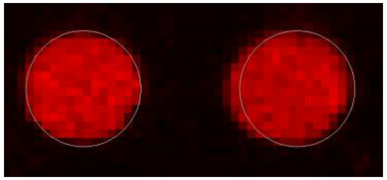

Grade IV_CJ 13655_2000153783

Grade IV

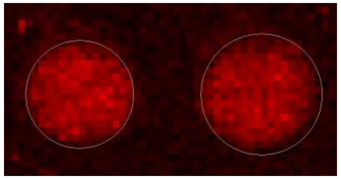
Grade IV_CJ 18704_2000153784

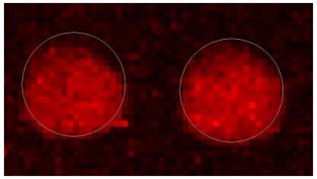
Grade IV_CH 21783_2000153785

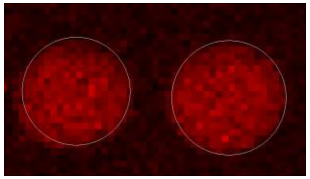
Grade IV_CF 27891_2000153776

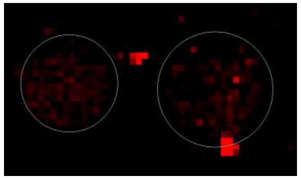
Grade IV_CH 8498_2000153777

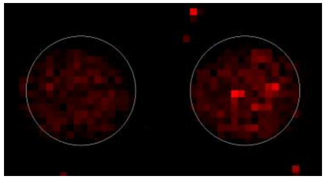
Grade IV_CH 12724_2000153778

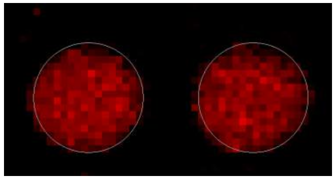
Grade IV_CF 11940_2000153779

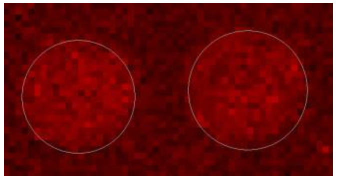
Grade IV_CJ 22776_2000153756

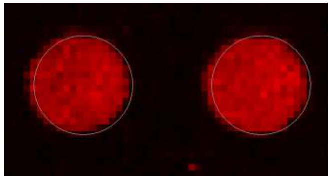
Grade IV_CH 18426_2000153757

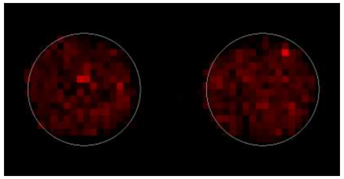
Grade IV_CH 6643_2000153758

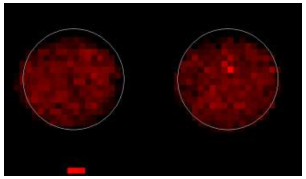
Grade IV_CH 29697_2000153759

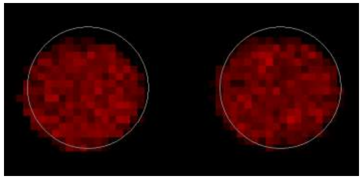
Grade IV_CJ 12441_2000153760

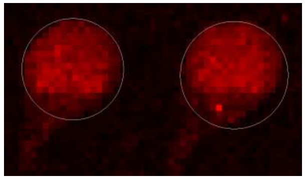
Grade IV_CH 5261_2000155722

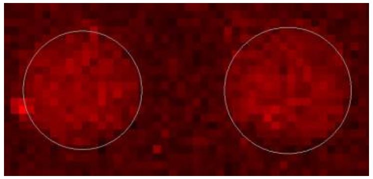
Grade IV_CF 19257 _2000155723

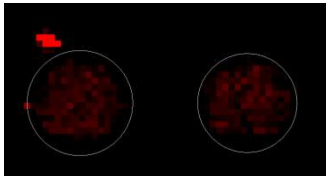
Grade IV_CJ 10048_2000155725

Grade IV

NM_144495.2 PQBP1

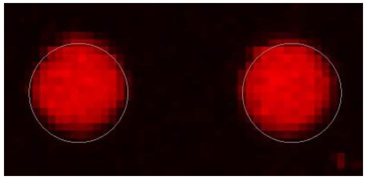
Control_H-02_2000153953

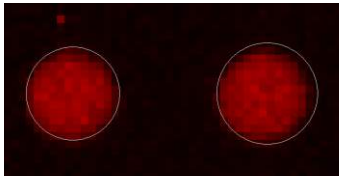
Control_H-03_2000153943

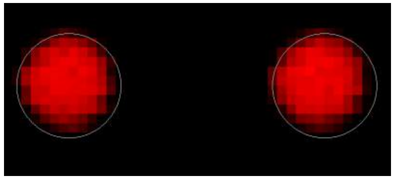
Control_H-19_2000154008

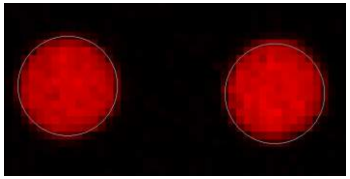
Control_H-23_2000154009

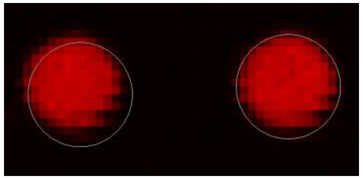
Control_H-25_2000153952

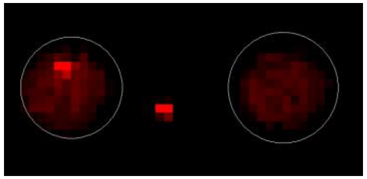
Control_H-35_2000153942

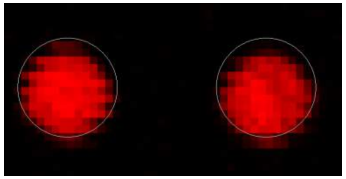
Control_H-41_2000154026

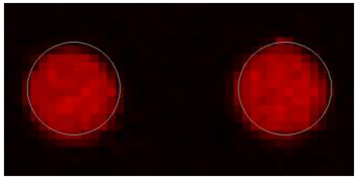
Control_H-58_2000153935

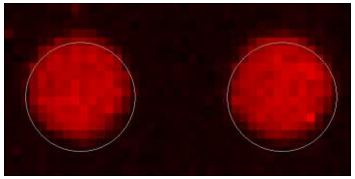
Control_H-59_2000154016

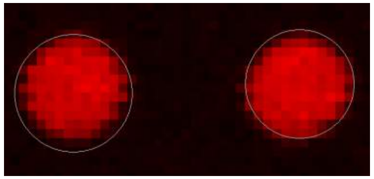
Control_HC 25_2000153932

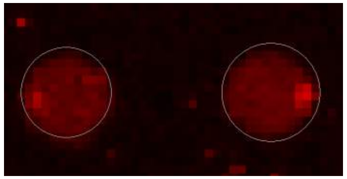
Control_HV-56_2000155735

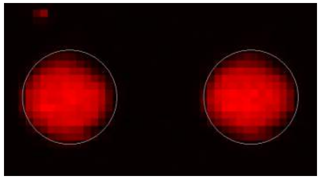
Control_HV-59_2000155740

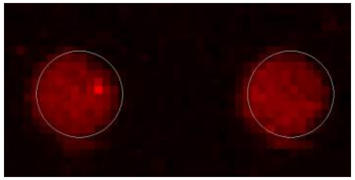
Control_HV-64_2000144456

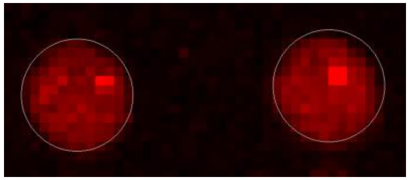
Control_HV-70_2000144457

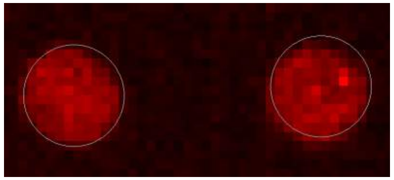
Control_HV-71_2000144458

Control

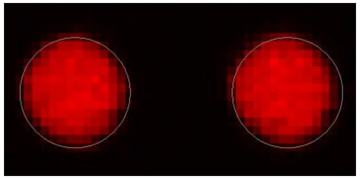

Grade IV_CF 7916_2000154021

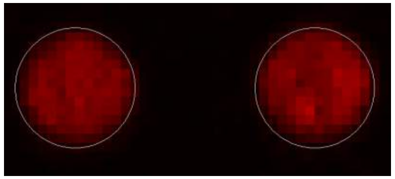

Grade IV_CJ 11291_2000154025

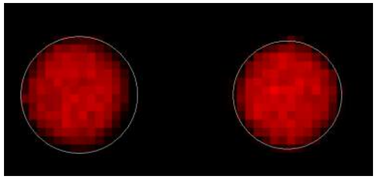

Grade IV_CJ 2041_2000154019

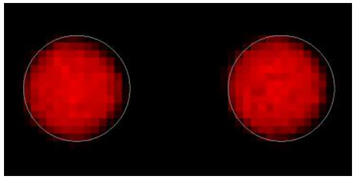

Grade IV_CJ 1519_2000153951

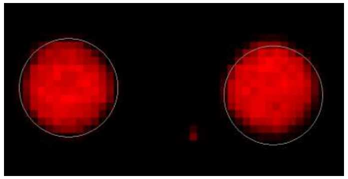

Grade IV_CF 7522_2000154010

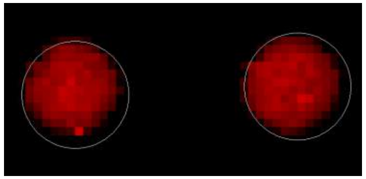

Grade IV_CH 9002_2000153954

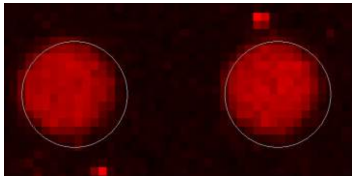

Grade IV_CF 11518_2000153942

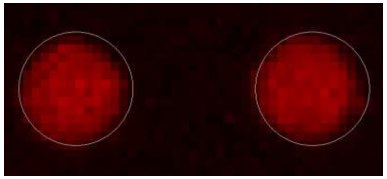

Grade IV_CH 3969_2000153950

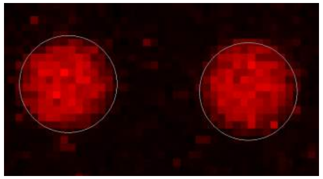

Grade IV_CH 4996_2000153933

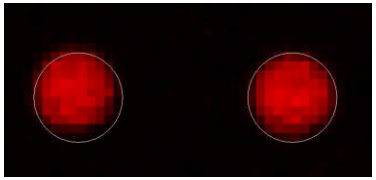

Grade IV_CF 12166_2000153934

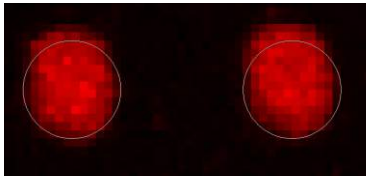

Grade IV_CH 14820_2000153796

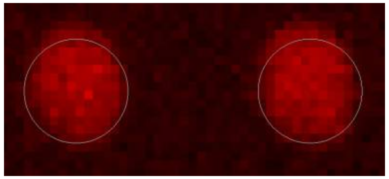

Grade IV_CJ 32705_2000153797

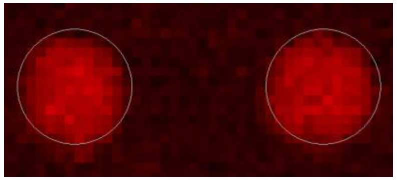

Grade IV_CH 29738_2000153798

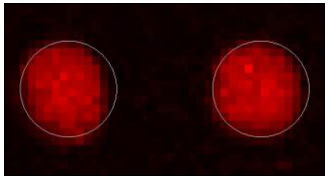

Grade IV_CJ 30014_2000153805

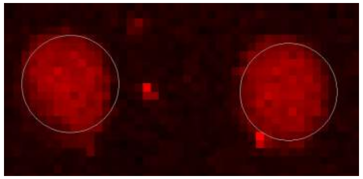

Grade IV_CH 18016_2000153791

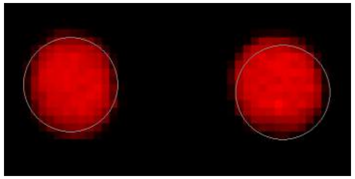

Grade IV_CH 30203_2000153793

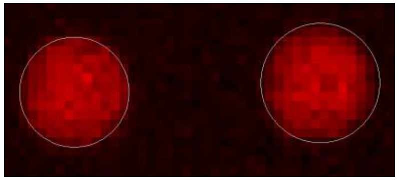

Grade IV_CH 28569_2000153799

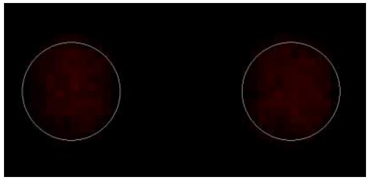

Grade IV_CJ 6254_2000153800

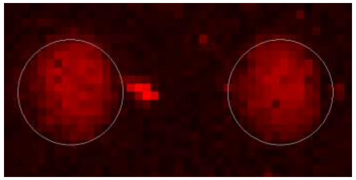

Grade IV_CH 22618_2000153782

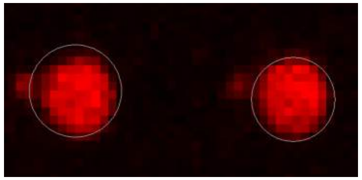

Grade IV_CJ 13655_2000153783

Grade IV

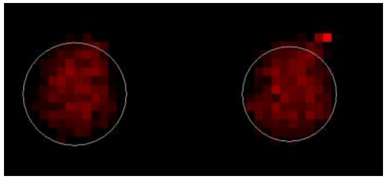
Grade IV_CJ 18704_2000153784

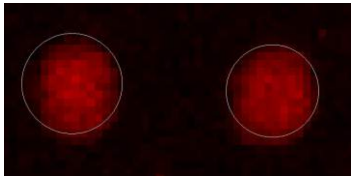
Grade IV_CH 21783_2000153785

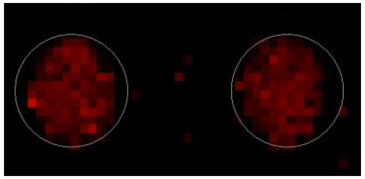
Grade IV_CF 27891_2000153776

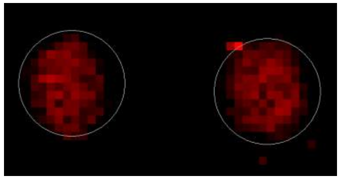
Grade IV_CH 8498_2000153777

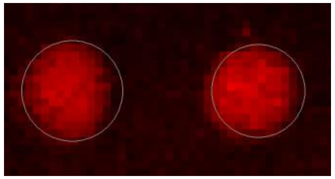
Grade IV_CH 12724_2000153778

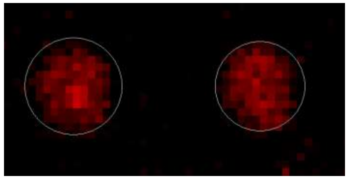
Grade IV_CF 11940_2000153779

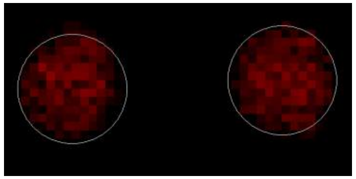
Grade IV_CJ 22776_2000153756

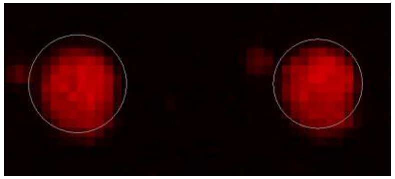
Grade IV_CH 18426_2000153757

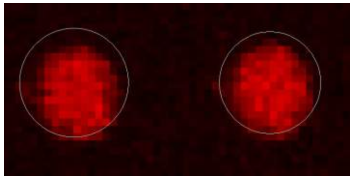
Grade IV_CH 6643_2000153758

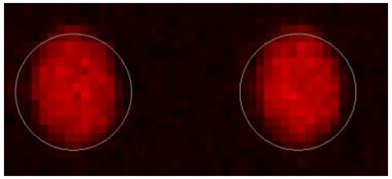
Grade IV_CH 29697_2000153759

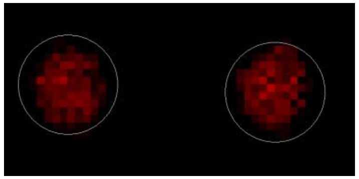
Grade IV_CJ 12441_2000153760

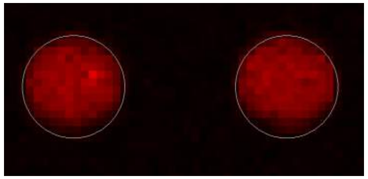
Grade IV_CH 5261_2000155722

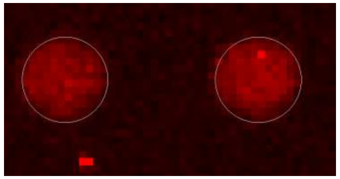
Grade IV_CF 19257 _2000155723

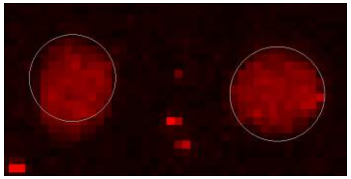
Grade IV_CJ 10048_2000155725

Grade IV

NM_021810.3 CDH26

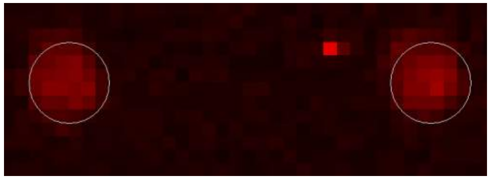
Control_H-02_2000153953

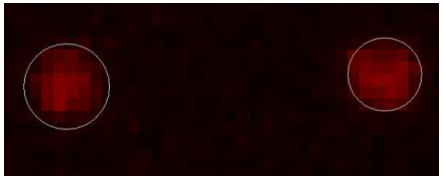
Control_H-03_2000153943

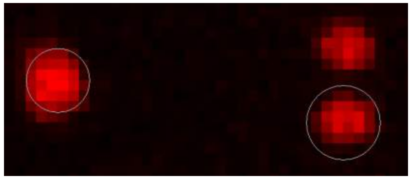
Control_H-19_2000154008

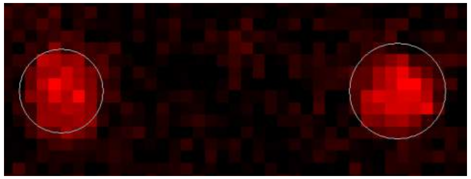
Control_H-23_2000154009

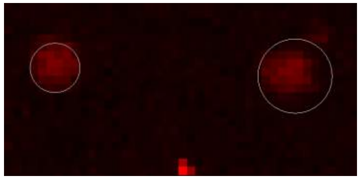
Control_H-25_2000153952

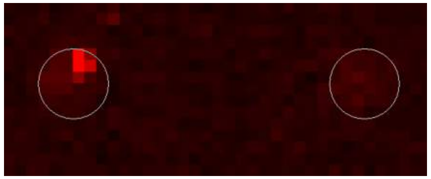
Control_H-35_2000153942

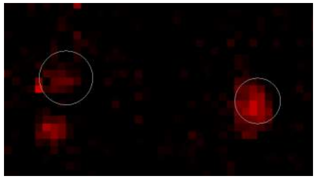
Control_H-41_2000154026

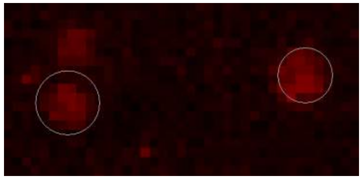
Control_H-58_2000153935

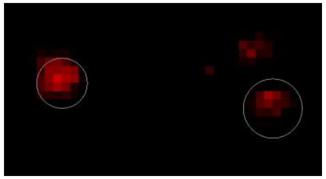
Control_H-59_2000154016

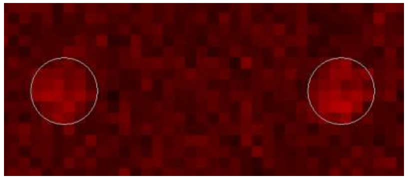
Control_HC 25_2000153932

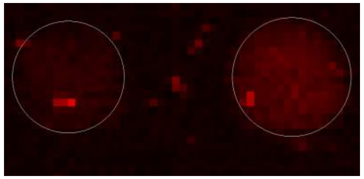
Control_HV-56_2000155735

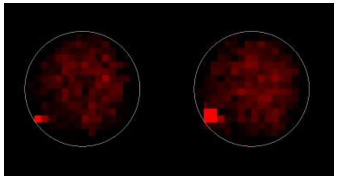
Control_HV-59_2000155740

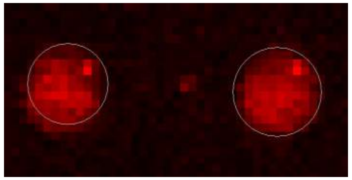
Control_HV-64_2000144456

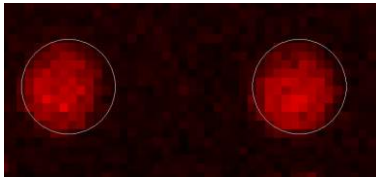
Control_HV-70_2000144457

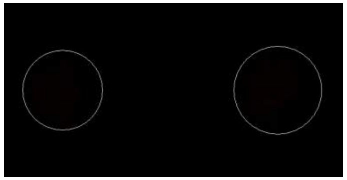
Control_HV-71_2000144458

Control

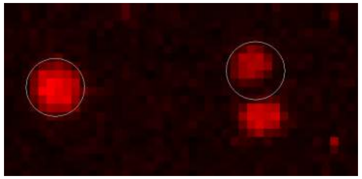
Grade IV_CF 7916_2000154021

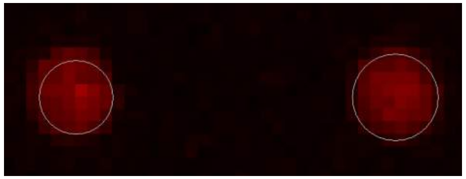
Grade IV_CJ 11291_2000154025

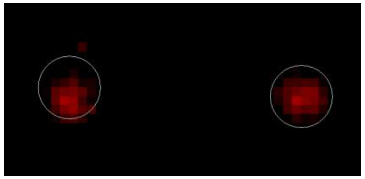
Grade IV_CJ 2041_2000154019

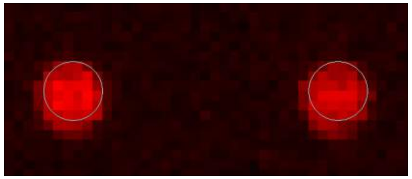
Grade IV_CJ 1519_2000153951

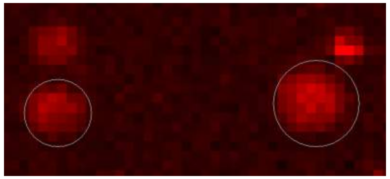
Grade IV_CF 7522_2000154010

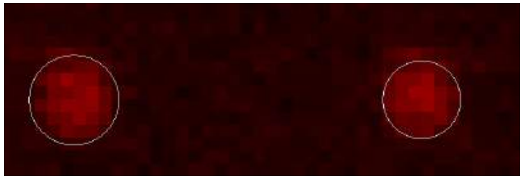
Grade IV_CH 9002_2000153954

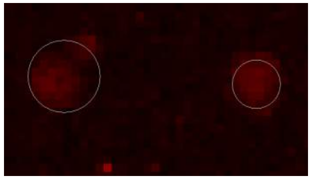
Grade IV_CF 11518_2000153942

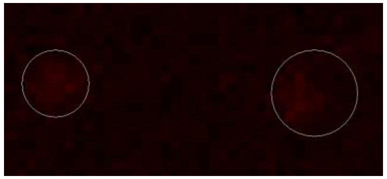
Grade IV_CH 3969_2000153950

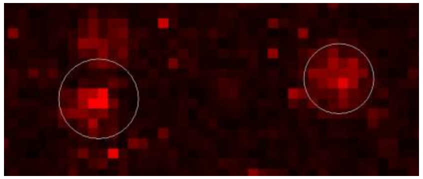
Grade IV_CH 4996_2000153933

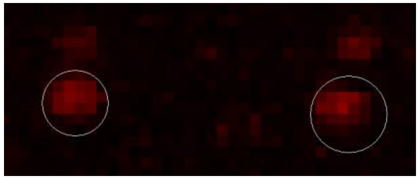
Grade IV_CF 12166_2000153934

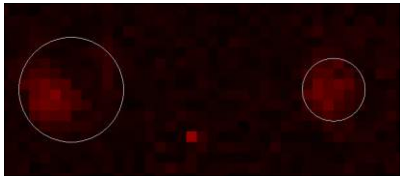
Grade IV_CH 14820_2000153796

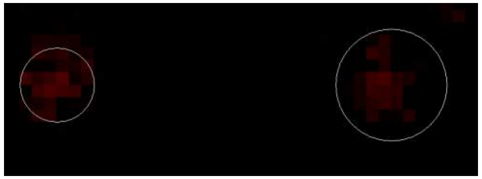
Grade IV_CJ 32705_2000153797

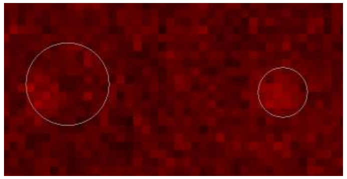
Grade IV_CH 29738_2000153798

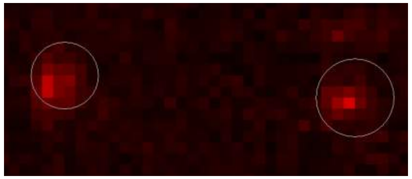
Grade IV_CJ 30014_2000153805

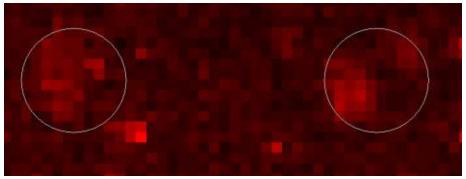
Grade IV_CH 18016_2000153791

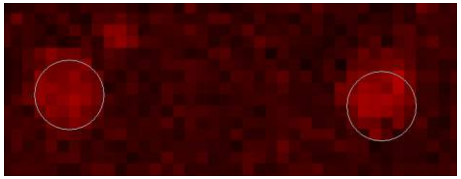
Grade IV_CH 30203_2000153793

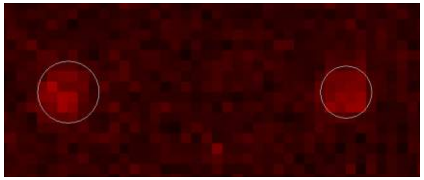
Grade IV_CH 28569_2000153799

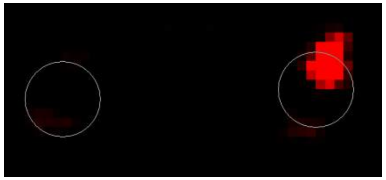
Grade IV_CJ 6254_2000153800

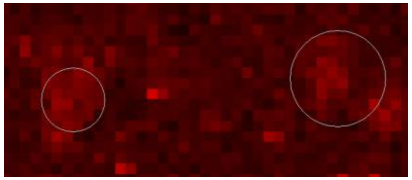
Grade IV_CH 22618_2000153782

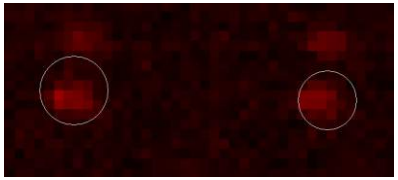
Grade IV_CJ 13655_2000153783

Grade IV

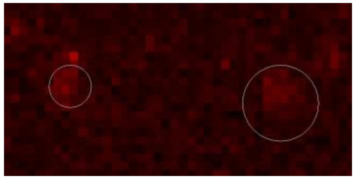
Grade IV_CJ 18704_2000153784

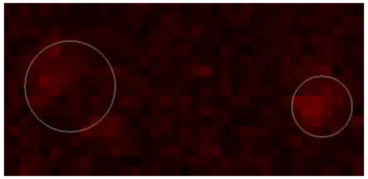
Grade IV_CH 21783_2000153785

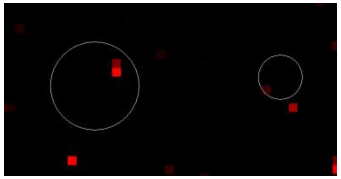
Grade IV_CF 27891_2000153776

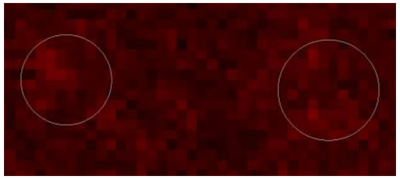
Grade IV_CH 8498_2000153777

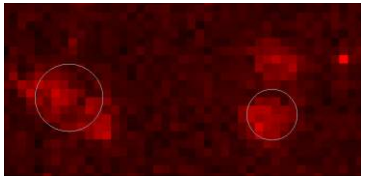
Grade IV_CH 12724_2000153778

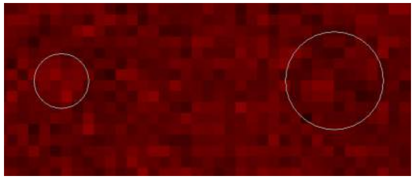
Grade IV_CF 11940_2000153779

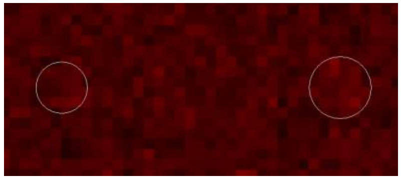
Grade IV_CJ 22776_2000153756

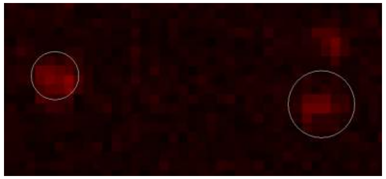
Grade IV_CH 18426_2000153757

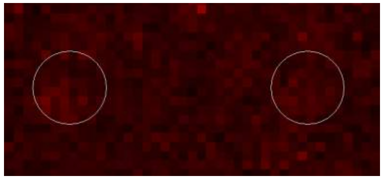
Grade IV_CH 6643_2000153758

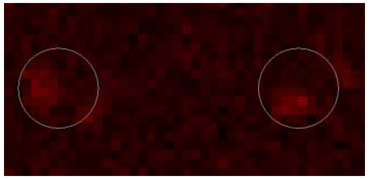
Grade IV_CH 29697_2000153759

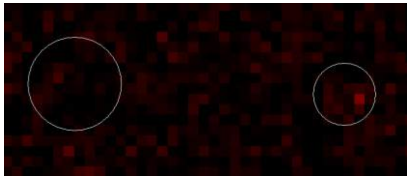
Grade IV_CJ 12441_2000153760

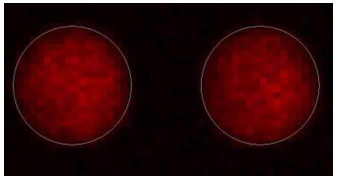
Grade IV_CH 5261_2000155722

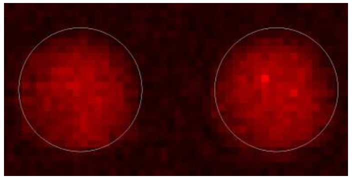
Grade IV_CF 19257 _2000155723

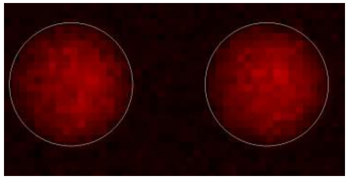
Grade IV_CJ 10048_2000155725

Grade IV

BC013186.1 RCSD1

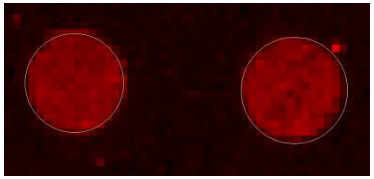
Control_H-02_2000153953

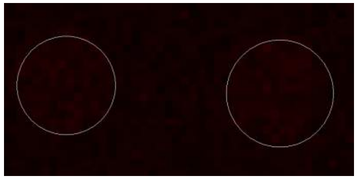
Control_H-03_2000153943

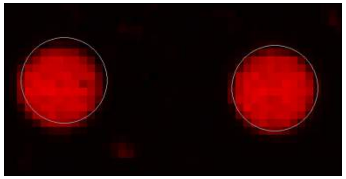
Control_H-19_2000154008

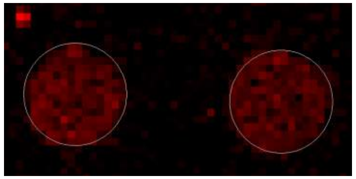
Control_H-23_2000154009

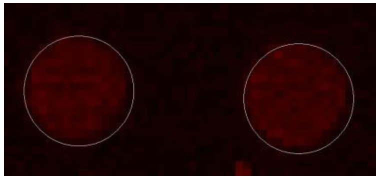
Control_H-25_2000153952

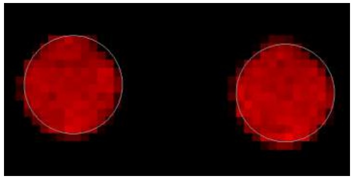
Control_H-35_2000153942

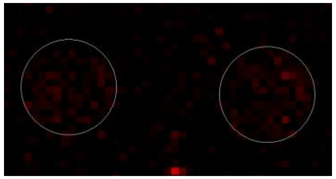
Control_H-41_2000154026

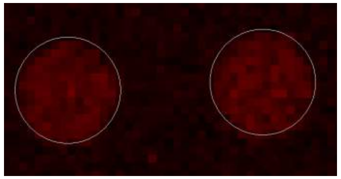
Control_H-58_2000153935

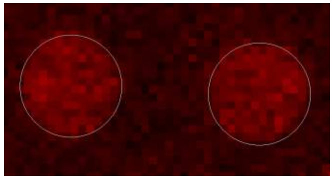
Control_H-59_2000154016

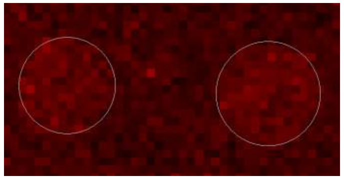
Control_HC 25_2000153932

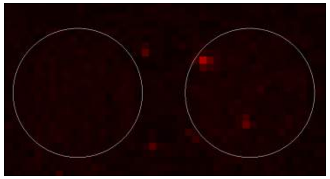
Control_HV-56_2000155735

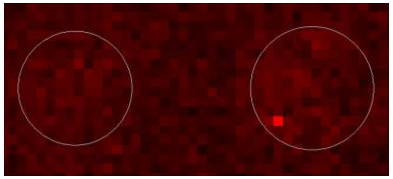
Control_HV-59_2000155740

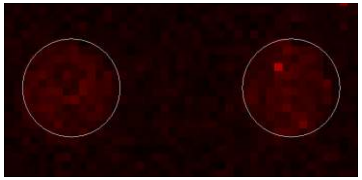
Control_HV-64_2000144456

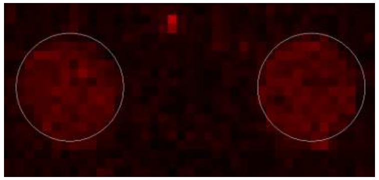
Control_HV-70_2000144457

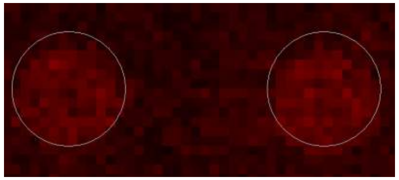
Control_HV-71_2000144458

Control

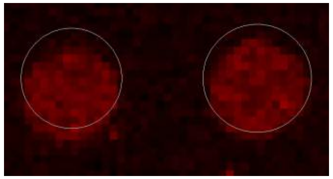
Grade IV_CF 7916_2000154021

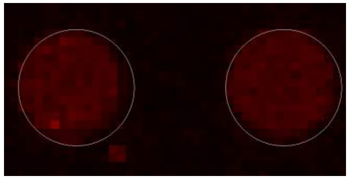
Grade IV_CJ 11291_2000154025

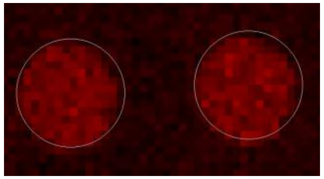
Grade IV_CJ 2041_2000154019

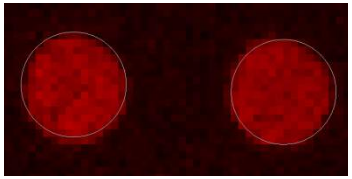
Grade IV_CJ 1519_2000153951

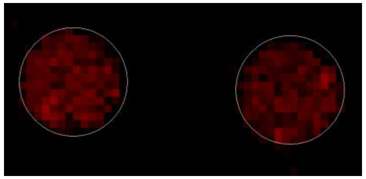
Grade IV_CF 7522_2000154010

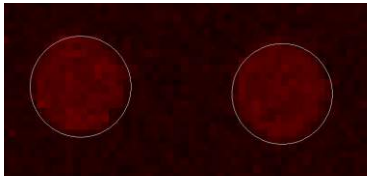
Grade IV_CH 9002_2000153954

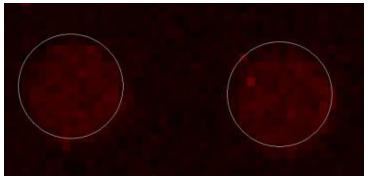
Grade IV_CF 11518_2000153942

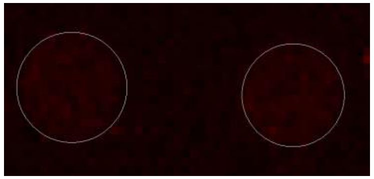
Grade IV_CH 3969_2000153950

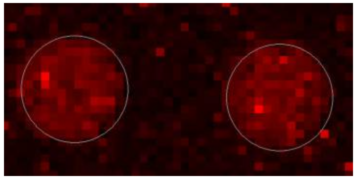
Grade IV_CH 4996_2000153933

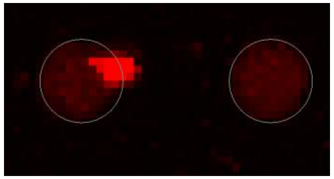
Grade IV_CF 12166_2000153934

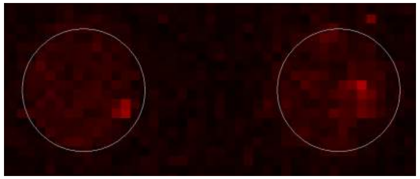
Grade IV_CH 14820_2000153796

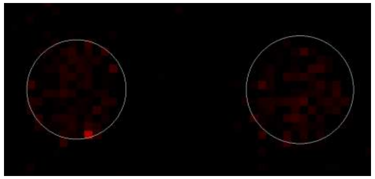
Grade IV_CJ 32705_2000153797

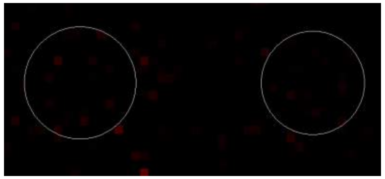
Grade IV_CH 29738_2000153798

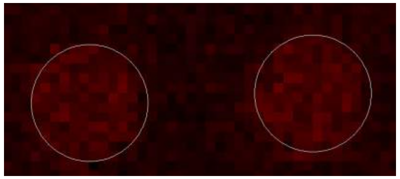
Grade IV_CJ 30014_2000153805

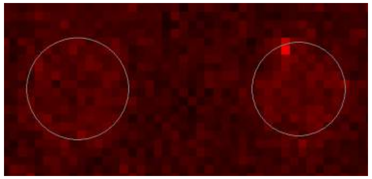
Grade IV_CH 18016_2000153791

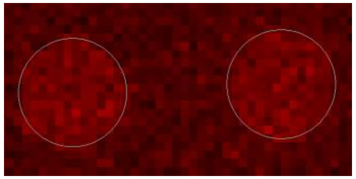
Grade IV_CH 30203_2000153793

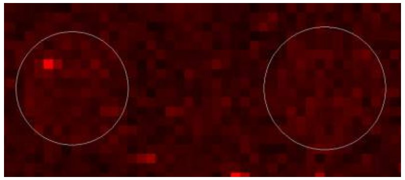
Grade IV_CH 28569_2000153799

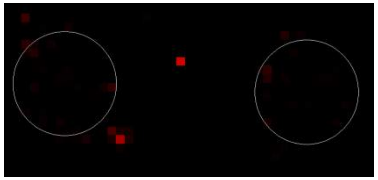
Grade IV_CJ 6254_2000153800

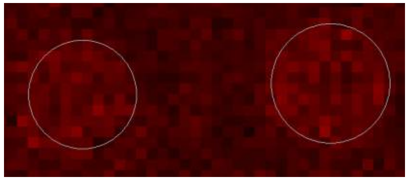
Grade IV_CH 22618_2000153782

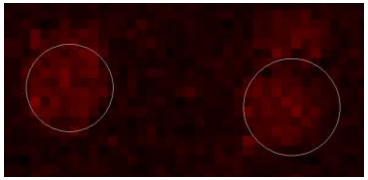
Grade IV_CJ 13655_2000153783

Grade IV

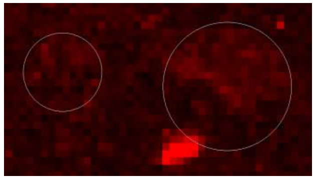

Grade IV_CJ 18704_2000153784

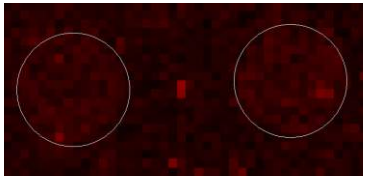

Grade IV_CH 21783_2000153785

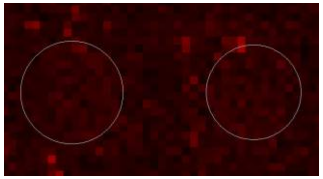

Grade IV_CF 27891_2000153776

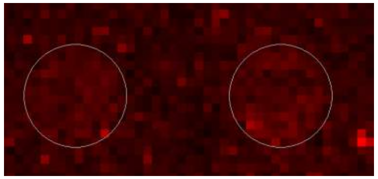

Grade IV_CH 8498_2000153777

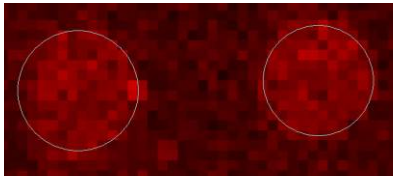

Grade IV_CH 12724_2000153778

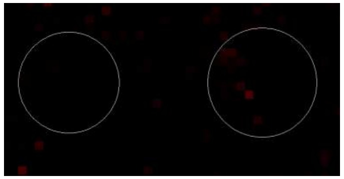

Grade IV_CF 11940_2000153779

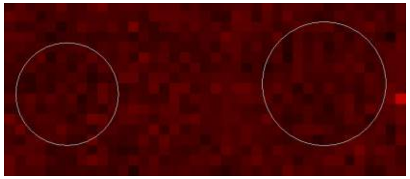

Grade IV_CJ 22776_2000153756

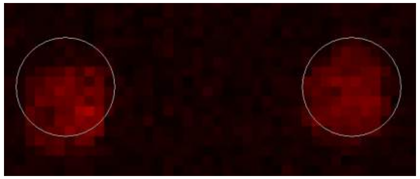

Grade IV_CH 18426_2000153757

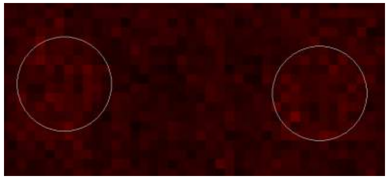

Grade IV_CH 6643_2000153758

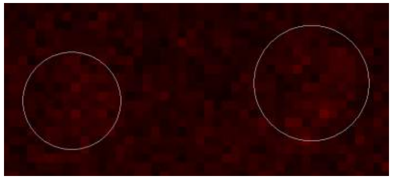

Grade IV_CH 29697_2000153759

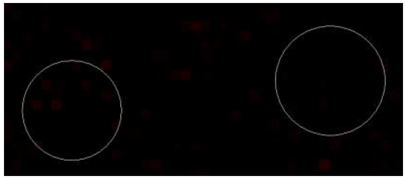

Grade IV_CJ 12441_2000153760

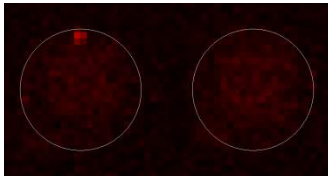

Grade IV_CH 5261_2000155722

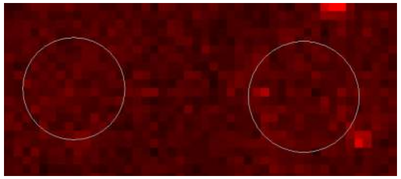

Grade IV_CF 19257 _2000155723

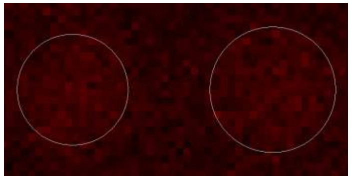

Grade IV_CJ 10048_2000155725

Grade IV

BC000846.2 CIB1

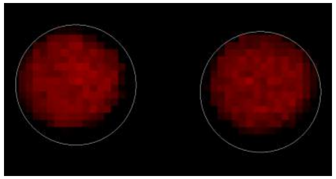

Control_H-02_2000153953

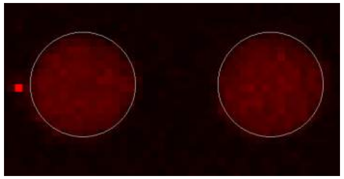

Control_H-03_2000153943

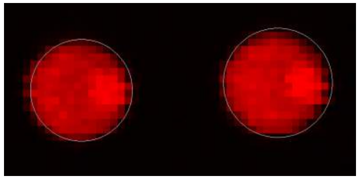

Control_H-19_2000154008

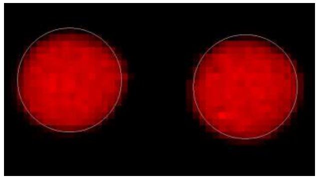

Control_H-23_2000154009

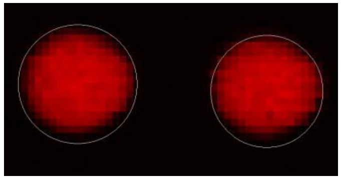

Control_H-25_2000153952

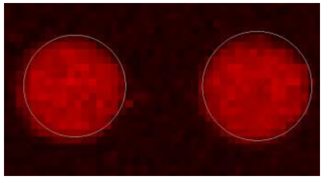

Control_H-35_2000153942

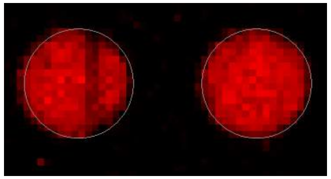

Control_H-41_2000154026

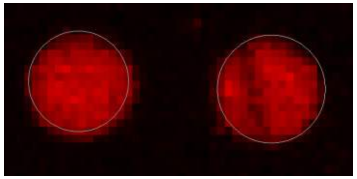

Control_H-58_2000153935

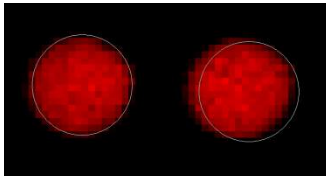

Control_H-59_2000154016

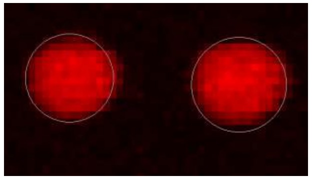

Control_HC 25_2000153932

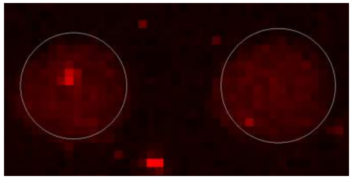

Control_HV-56_2000155735

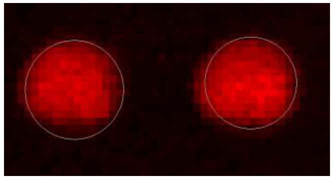

Control_HV-59_2000155740

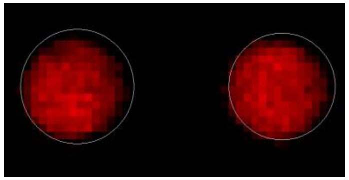

Control_HV-64_2000144456

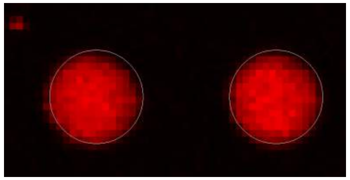

Control_HV-70_2000144457

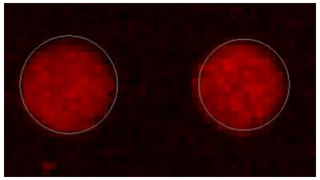

Control_HV-71_2000144458

Control

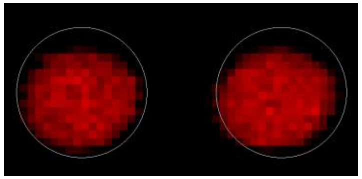

Grade IV_CF 7916_2000154021

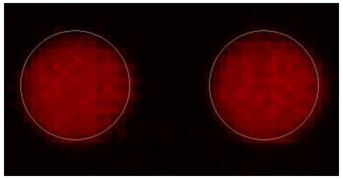

Grade IV_CJ 11291_2000154025

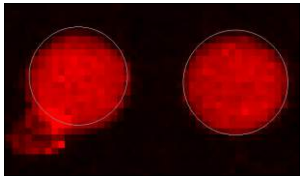

Grade IV_CJ 2041_2000154019

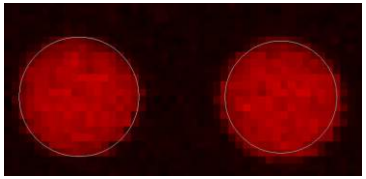

Grade IV_CJ 1519_2000153951

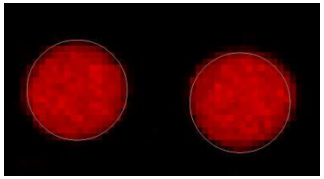

Grade IV_CF 7522_2000154010

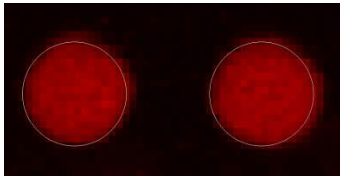

Grade IV_CH 9002_2000153954

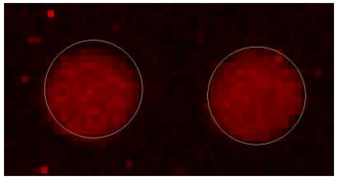

Grade IV_CF 11518_2000153942

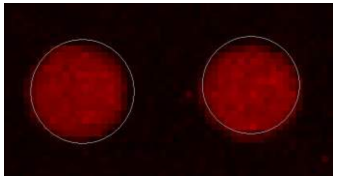

Grade IV_CH 3969_2000153950

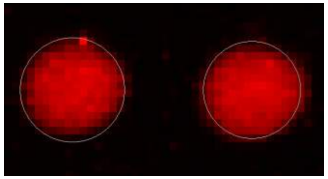

Grade IV_CH 4996_2000153933

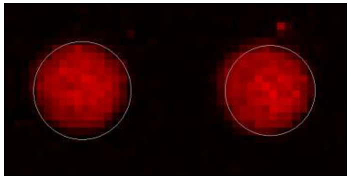

Grade IV_CF 12166_2000153934

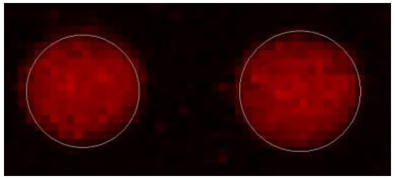

Grade IV_CH 14820_2000153796

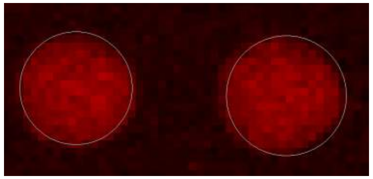

Grade IV_CJ 32705_2000153797

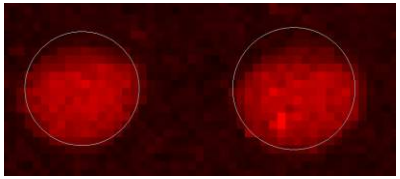

Grade IV_CH 29738_2000153798

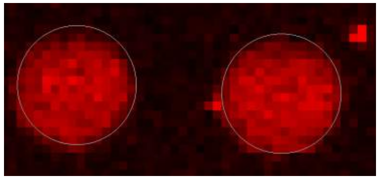

Grade IV_CJ 30014_2000153805

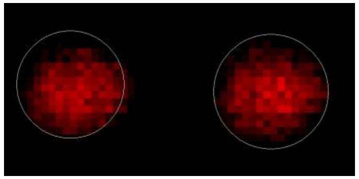

Grade IV_CH 18016_2000153791

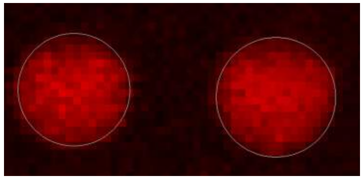

Grade IV_CH 30203_2000153793

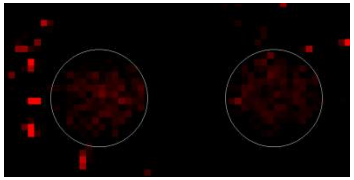

Grade IV_CH 28569_2000153799

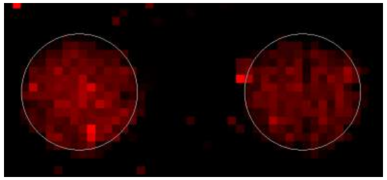

Grade IV_CJ 6254_2000153800

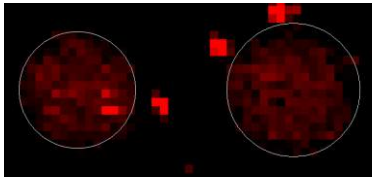

Grade IV_CH 22618_2000153782

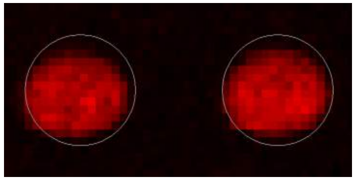

Grade IV_CJ 13655_2000153783

Grade IV

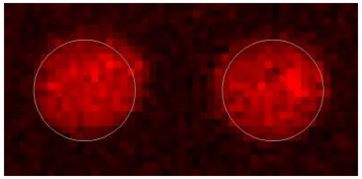

Grade IV_CJ 18704_2000153784

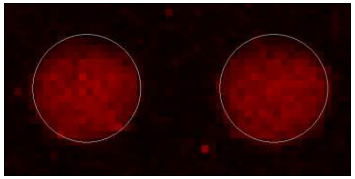

Grade IV_CH 21783_2000153785

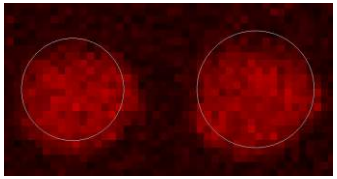

Grade IV_CF 27891_2000153776

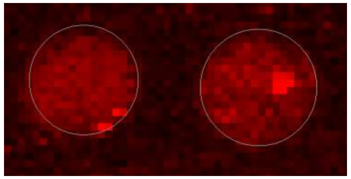

Grade IV_CH 8498_2000153777

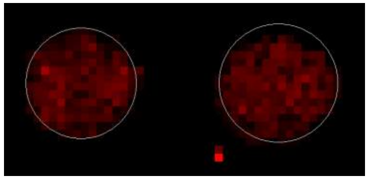

Grade IV_CH 12724_2000153778

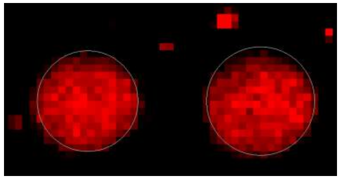

Grade IV_CF 11940_2000153779

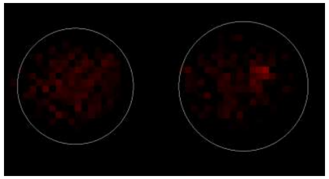

Grade IV_CJ 22776_2000153756

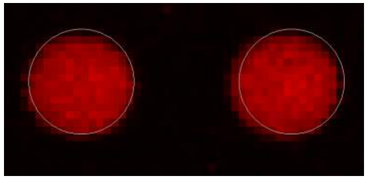

Grade IV_CH 18426_2000153757

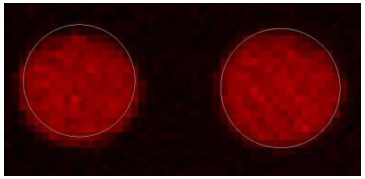

Grade IV_CH 6643_2000153758

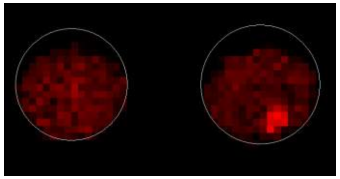

Grade IV_CH 29697_2000153759

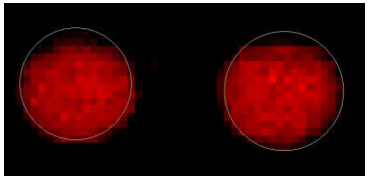

Grade IV_CJ 12441_2000153760

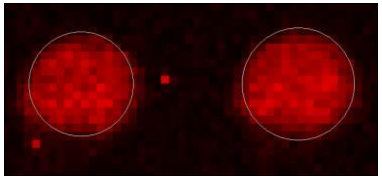

Grade IV_CH 5261_2000155722

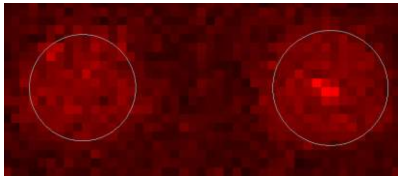

Grade IV_CF 19257 _2000155723

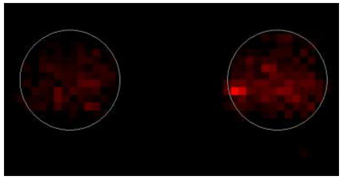

Grade IV_CJ 10048_2000155725

Grade IV

BC037876.1 C17orf57

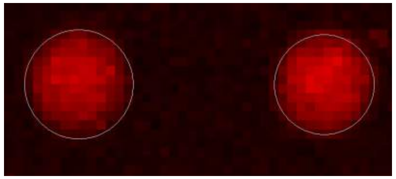
Control_H-02_2000153953

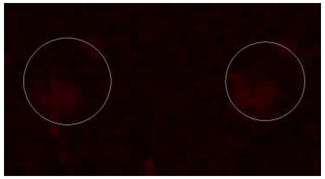
Control_H-03_2000153943

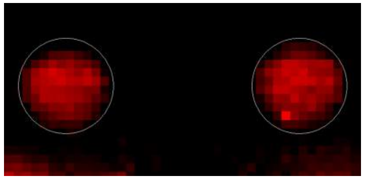
Control_H-19_2000154008

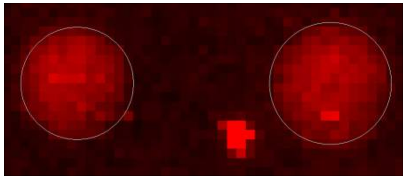
Control_H-23_2000154009

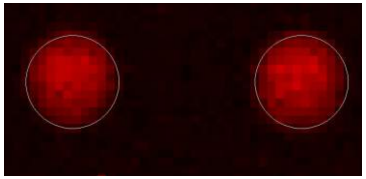
Control_H-25_2000153952

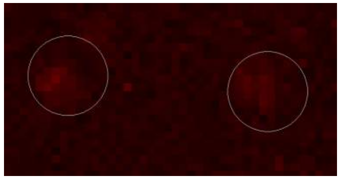
Control_H-35_2000153942

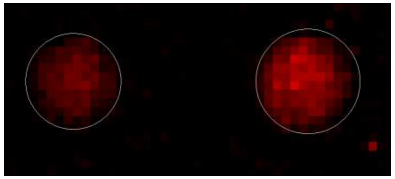
Control_H-41_2000154026

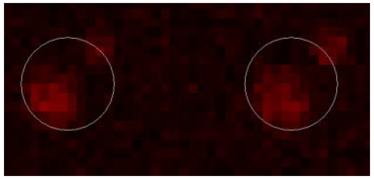
Control_H-58_2000153935

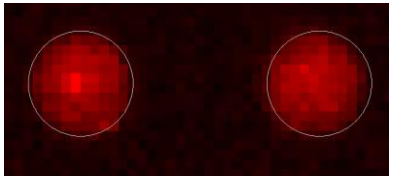
Control_H-59_2000154016

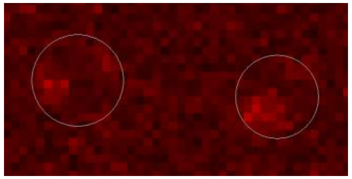
Control_HC 25_2000153932

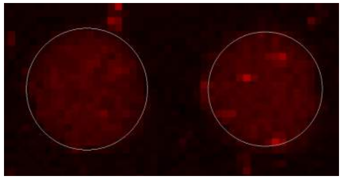
Control_HV-56_2000155735

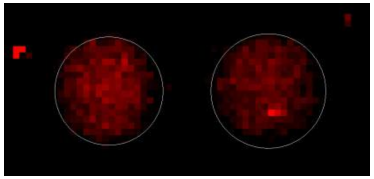
Control_HV-59_2000155740

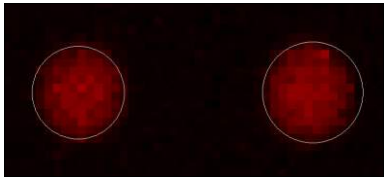
Control_HV-64_2000144456

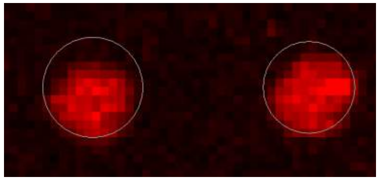
Control_HV-70_2000144457

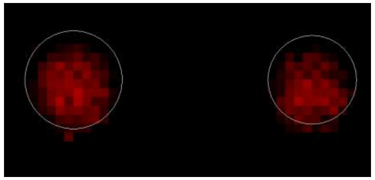
Control_HV-71_2000144458

Control

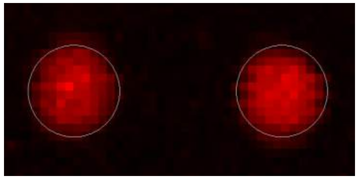
Grade IV_CF 7916_2000154021

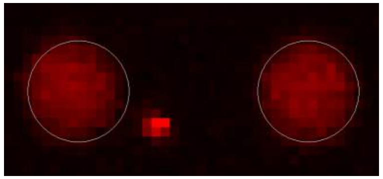
Grade IV_CJ 11291_2000154025

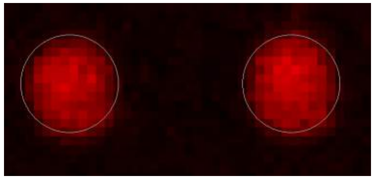
Grade IV_CJ 2041_2000154019

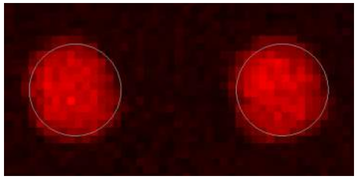
Grade IV_CJ 1519_2000153951

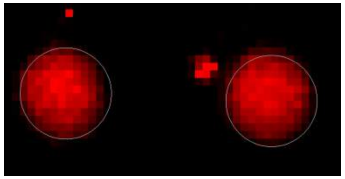
Grade IV_CF 7522_2000154010

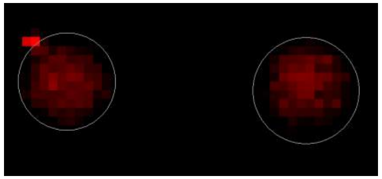
Grade IV_CH 9002_2000153954

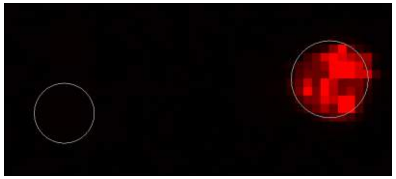
Grade IV_CF 11518_2000153942

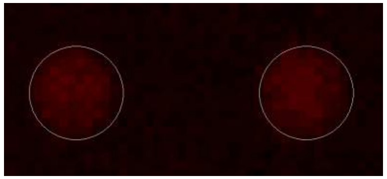
Grade IV_CH 3969_2000153950

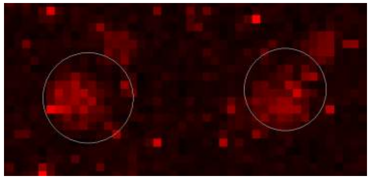
Grade IV_CH 4996_2000153933

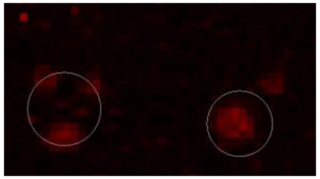
Grade IV_CF 12166_2000153934

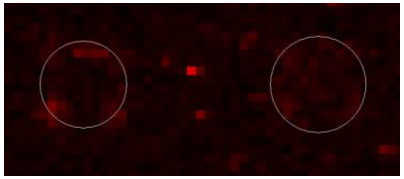
Grade IV_CH 14820_2000153796

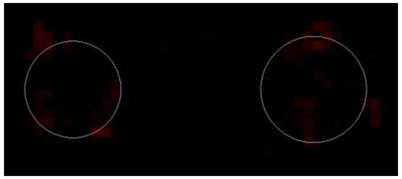
Grade IV_CJ 32705_2000153797

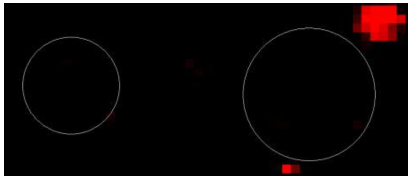
Grade IV_CH 29738_2000153798

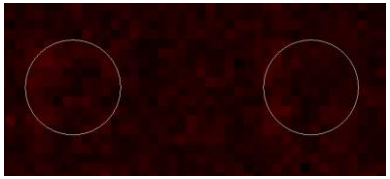
Grade IV_CJ 30014_2000153805

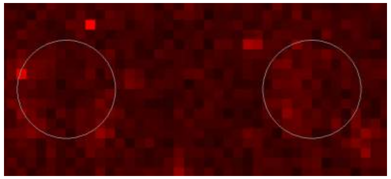
Grade IV_CH 18016_2000153791

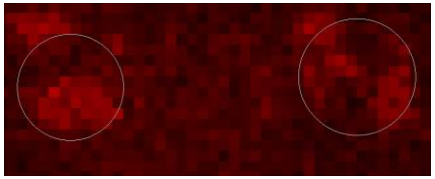
Grade IV_CH 30203_2000153793

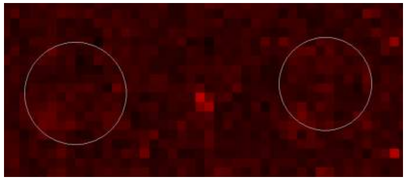
Grade IV_CH 28569_2000153799

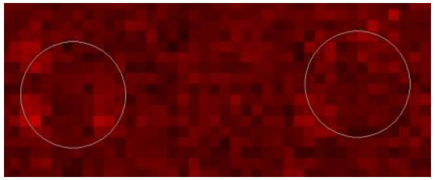
Grade IV_CJ 6254_2000153800

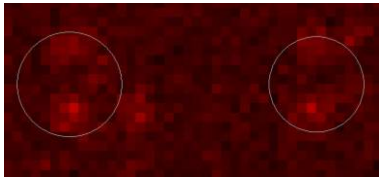
Grade IV_CH 22618_2000153782

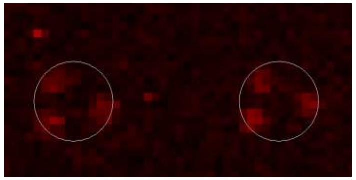
Grade IV_CJ 13655_2000153783

Grade IV

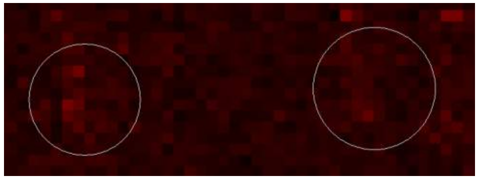
Grade IV_CJ 18704_2000153784

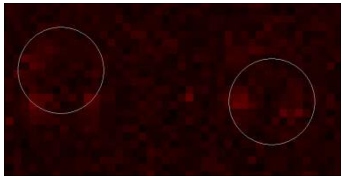
Grade IV_CH 21783_2000153785

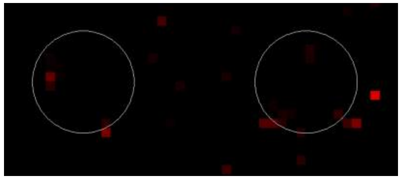
Grade IV_CF 27891_2000153776

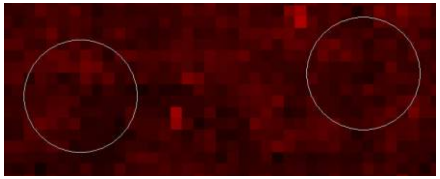
Grade IV_CH 8498_2000153777

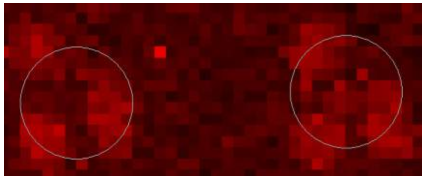
Grade IV_CH 12724_2000153778

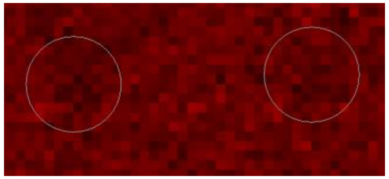
Grade IV_CF 11940_2000153779

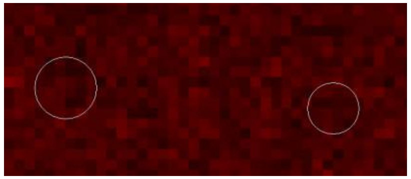
Grade IV_CJ 22776_2000153756

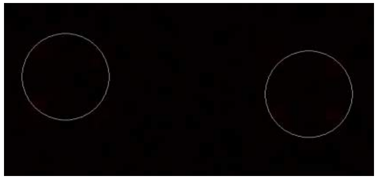
Grade IV_CH 18426_2000153757

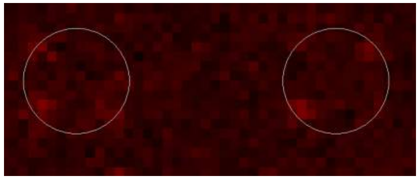
Grade IV_CH 6643_2000153758

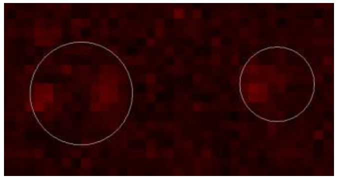
Grade IV_CH 29697_2000153759

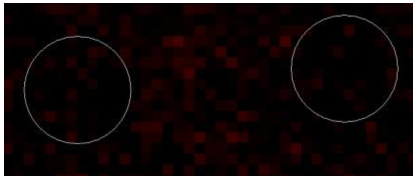
Grade IV_CJ 12441_2000153760

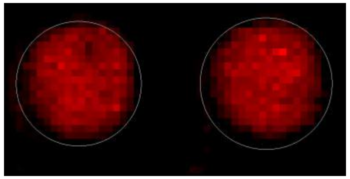
Grade IV_CH 5261_2000155722

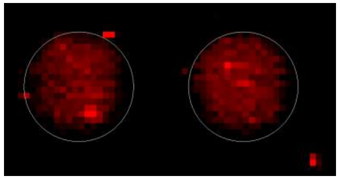
Grade IV_CF 19257 _2000155723

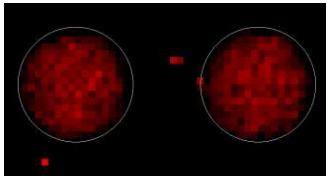
Grade IV_CJ 10048_2000155725

Grade IV

NM_001938.2 DR1

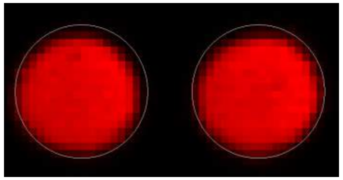
Grade IV_CF 7916_2000154021

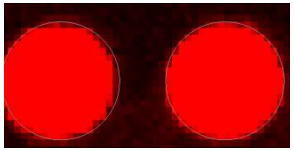
Grade IV_CJ 11291_2000154025

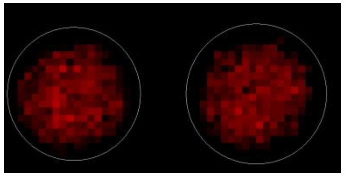
Grade IV_CJ 22776_2000153756

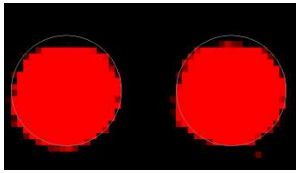
Grade IV_CJ 1519_2000153951

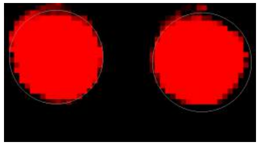
Grade IV_CH 28569_2000153799

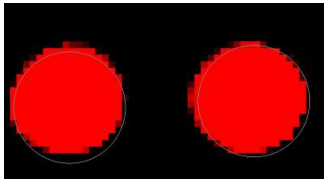
Grade IV_CF 7522_2000154010

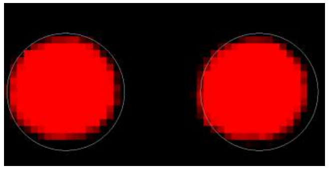
Grade IV_CH 9002_2000153954

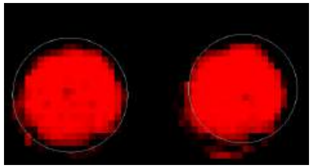
Grade IV_CH 21783_2000153785

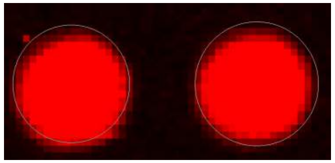
Grade IV_CH 3969_2000153950

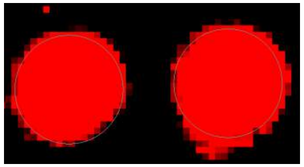
Grade IV_CH 4996_2000153933

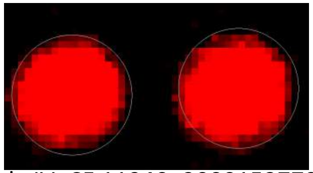
Grade IV_CF 11940_2000153779

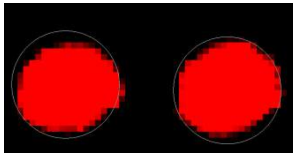
Grade IV_CH 14820_2000153796

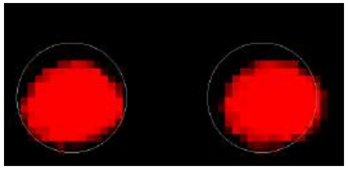
Grade IV_CH 18426_2000153757

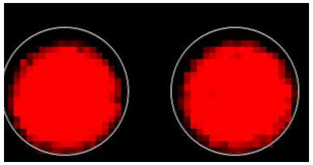
Grade IV_CH 6643_2000153758

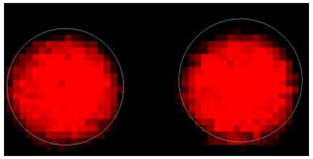
Grade IV_CH 29697_2000153759

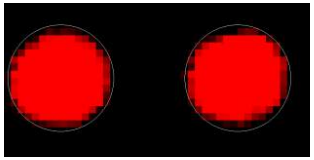
Grade IV_CF 12166_2000153934

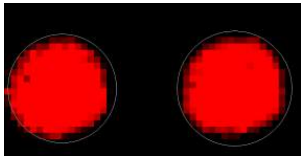
Grade IV_CH 30203_2000153793

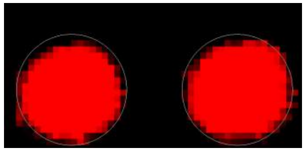
Grade IV_CH 29738_2000153798

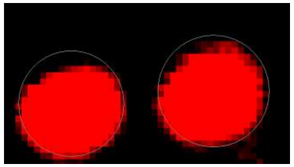
Grade IV_CJ 30014_2000153805

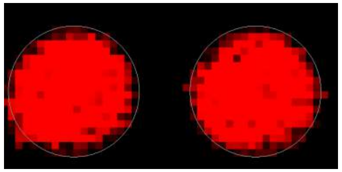
Grade IV_CH 22618_2000153782

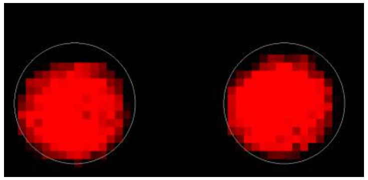
Grade IV_CJ 13655_2000153783

SVZp

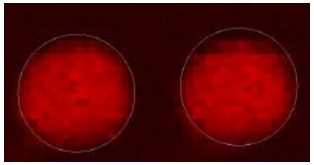

Grade IV_CJ 18704_2000153784

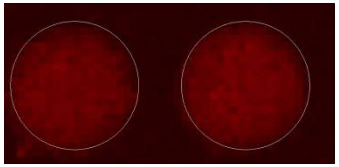

Grade IV_CF 27891_2000153776

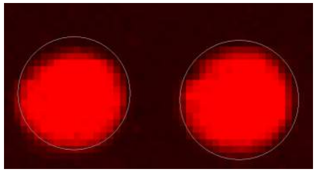

Grade IV_CJ 2041_2000154019

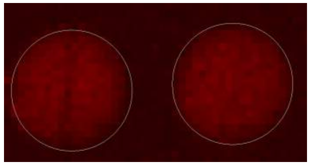

Grade IV_CH 8498_2000153777

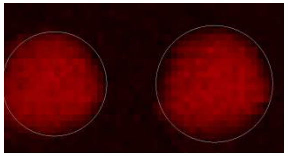

Grade IV_CJ 6254_2000153800

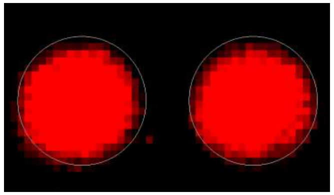

Grade IV_CH 12724_2000153778

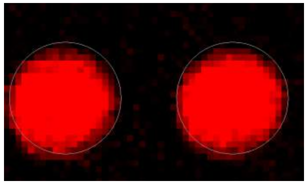

Grade IV_CJ 12441_2000153760

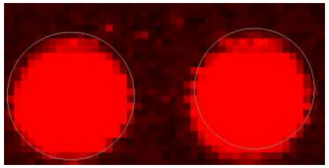

Grade IV_CF 11518_2000153942

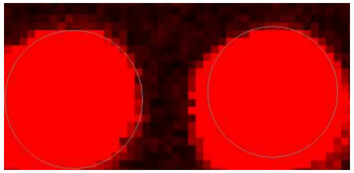

Grade IV_CH 5261_2000155722

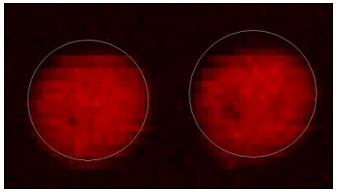

Grade IV_CF 19257_2000155723

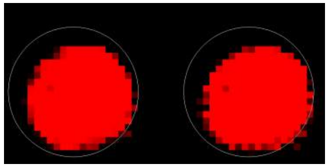

Grade IV_CH 18016_2000153791

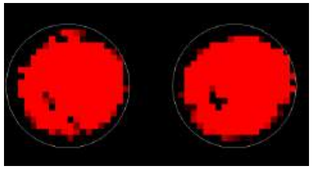

Grade IV_CJ 10048_2000155725

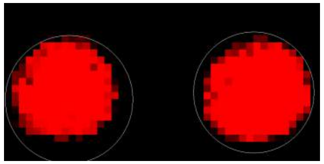

Grade IV_CJ 32705_2000153797

SVZn

NM_018290.2 PGM2

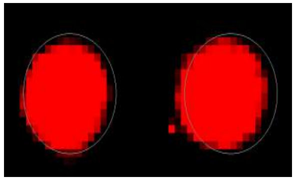
Grade IV_CF 7916_2000154021

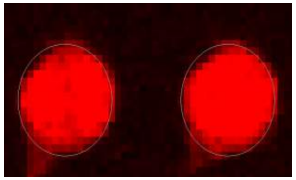
Grade IV_CJ 11291_2000154025

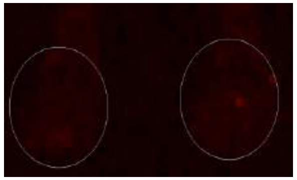
Grade IV_CJ 22776_2000153756

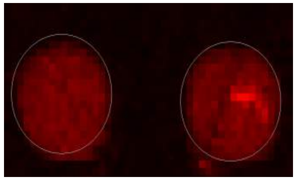
Grade IV_CJ 1519_2000153951

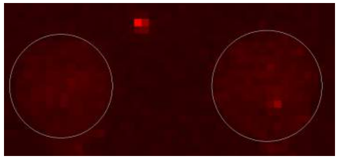
Grade IV_CH 28569_2000153799

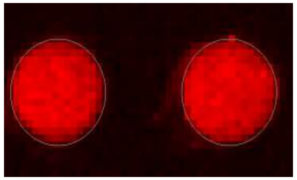
Grade IV_CF 7522_2000154010

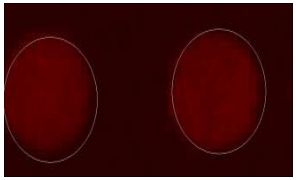
Grade IV_CH 9002_2000153954

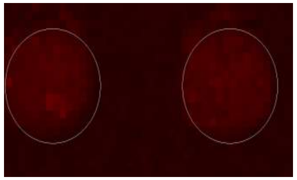
Grade IV_CH 21783_2000153785

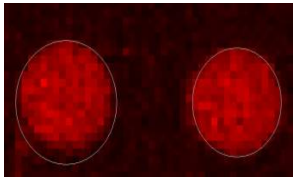
Grade IV_CH 3969_2000153950

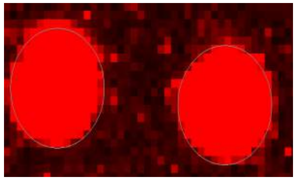
Grade IV_CH 4996_2000153933

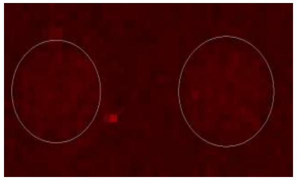
Grade IV_CF 11940_2000153779

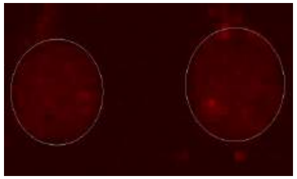
Grade IV_CH 14820_2000153796

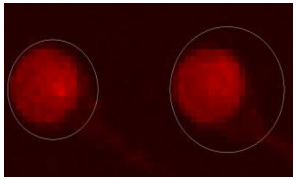
Grade IV_CH 18426_2000153757

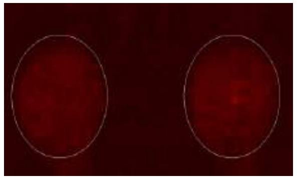
Grade IV_CH 6643_2000153758

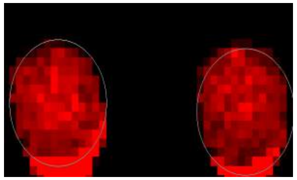
Grade IV_CH 29697_2000153759

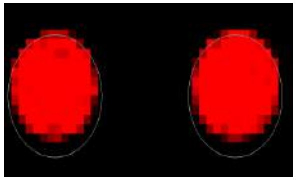
Grade IV_CF 12166_2000153934

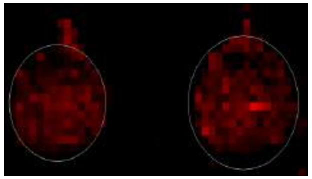
Grade IV_CH 30203_2000153793

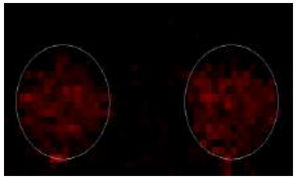
Grade IV_CH 29738_2000153798

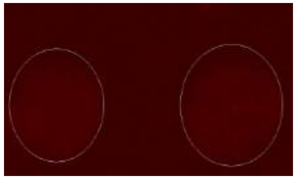
Grade IV_CJ 30014_2000153805

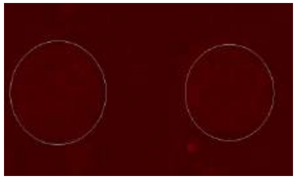
Grade IV_CH 22618_2000153782

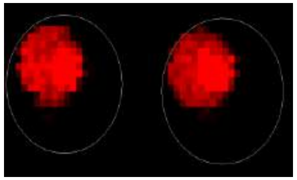
Grade IV_CJ 13655_2000153783

SVZp

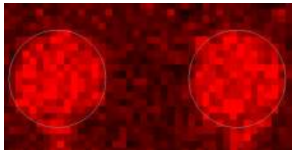
Grade IV_CJ 18704_2000153784

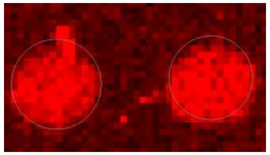
Grade IV_CF 27891_2000153776

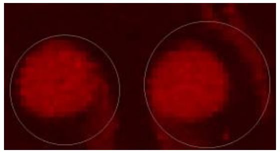
Grade IV_CJ 2041_2000154019

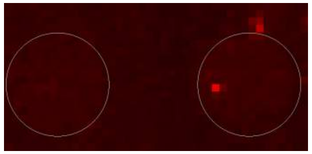
Grade IV_CH 8498_2000153777

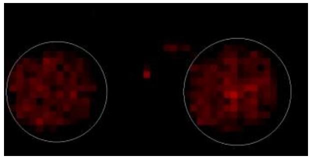
Grade IV_CJ 6254_2000153800

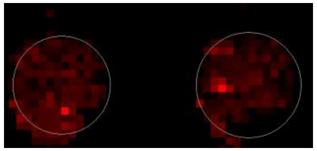
Grade IV_CH 12724_2000153778

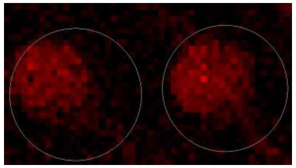
Grade IV_CJ 12441_2000153760

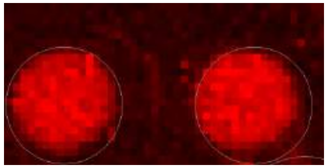
Grade IV_CF 11518_2000153942

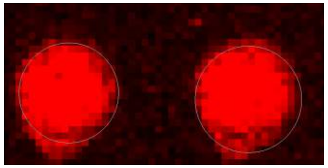
Grade IV_CH 5261_2000155722

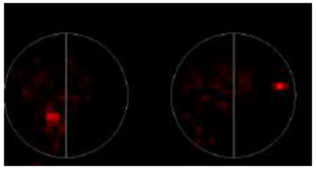
Grade IV_CF 19257 _2000155723

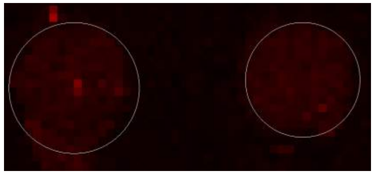
Grade IV_CH 18016_2000153791

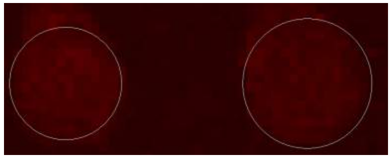
Grade IV_CJ 10048_2000155725

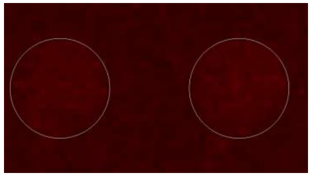
Grade IV_CJ 32705_2000153797

SVZn

NM_032448.1 FAM120B

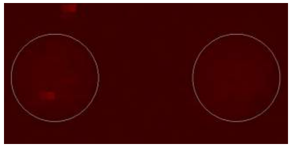
Grade IV_CF 7916_2000154021

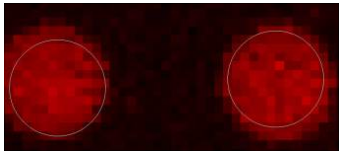
Grade IV_CJ 11291_2000154025

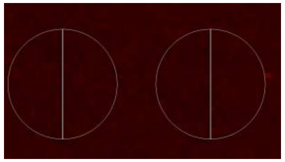
Grade IV_CJ 22776_2000153756

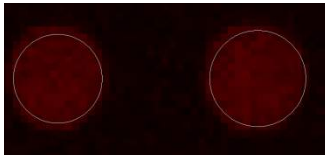
Grade IV_CJ 1519_2000153951

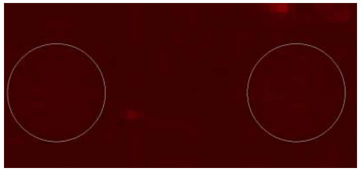
Grade IV_CH 28569_2000153799

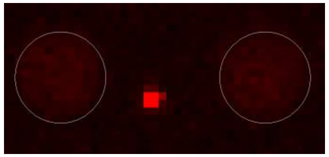
Grade IV_CF 7522_2000154010

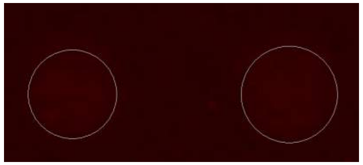
Grade IV_CH 9002_2000153954

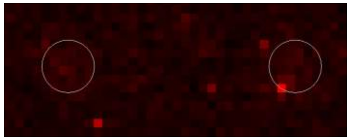
Grade IV_CH 21783_2000153785

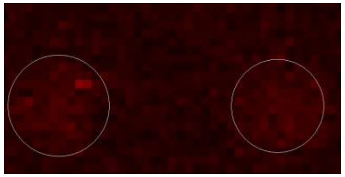
Grade IV_CH 3969_2000153950

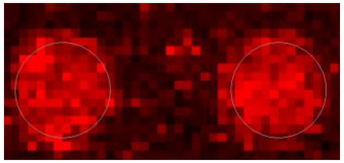
Grade IV_CH 4996_2000153933

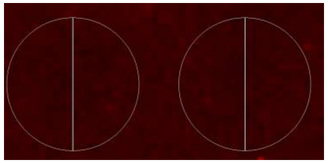
Grade IV_CF 11940_2000153779

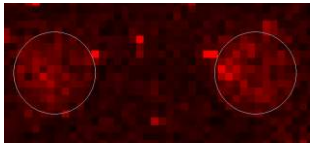
Grade IV_CH 14820_2000153796

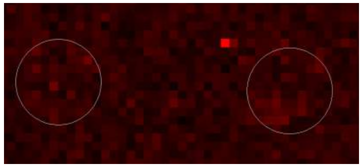
Grade IV_CH 18426_2000153757

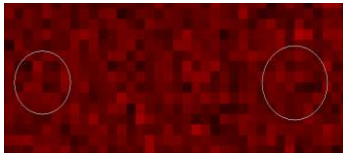
Grade IV_CH 6643_2000153758

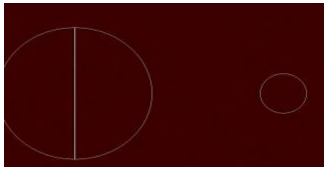
Grade IV_CH 29697_2000153759

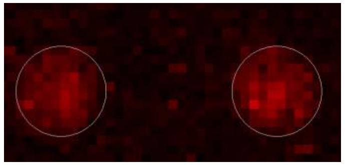
Grade IV_CF 12166_2000153934

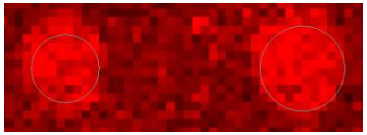
Grade IV_CH 30203_2000153793

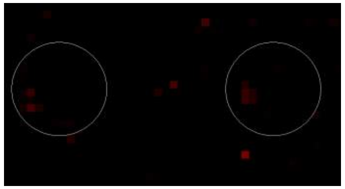
Grade IV_CH 29738_2000153798

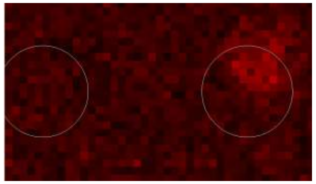
Grade IV_CJ 30014_2000153805

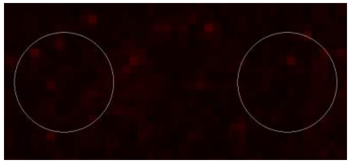
Grade IV_CH 22618_2000153782

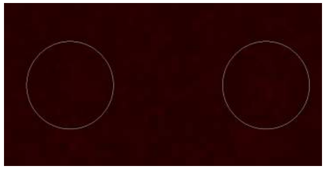
Grade IV_CJ 13655_2000153783

SVZp

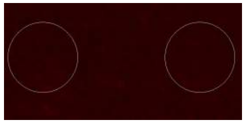

Grade IV_CJ 18704_2000153784

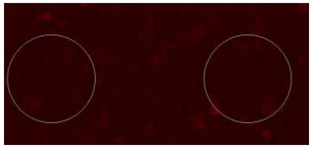

Grade IV_CF 27891_2000153776

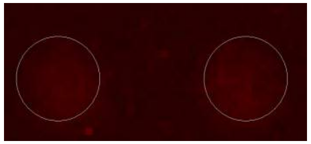

Grade IV_CJ 2041_2000154019

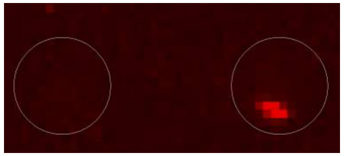

Grade IV_CH 8498_2000153777

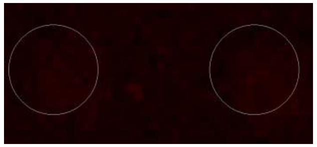

Grade IV_CJ 6254_2000153800

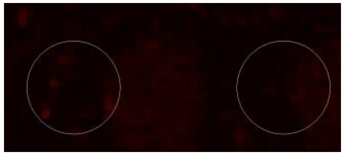

Grade IV_CH 12724_2000153778

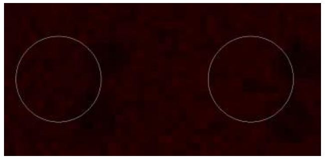

Grade IV_CJ 12441_2000153760

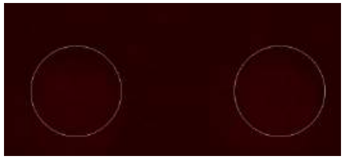

Grade IV_CF 11518_2000153942

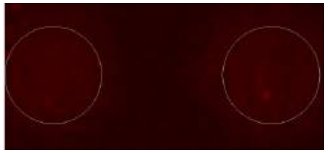

Grade IV_CH 5261_2000155722

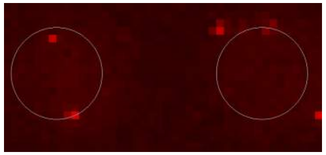

Grade IV_CF 19257 _2000155723

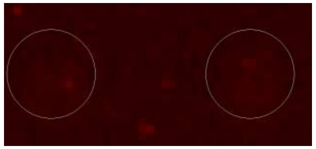

Grade IV_CH 18016_2000153791

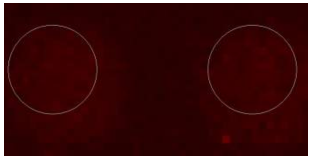

Grade IV_CJ 10048_2000155725

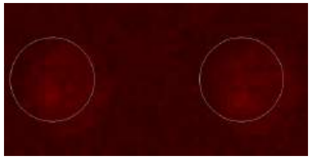

Grade IV_CJ 32705_2000153797

SVZn

NM_006403.2 NEDD9

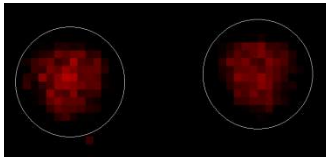
Grade IV_CF 7916_2000154021

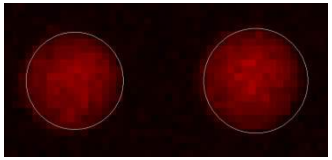
Grade IV_CJ 11291_2000154025

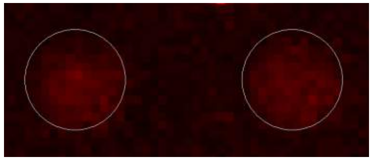
Grade IV_CJ 22776_2000153756

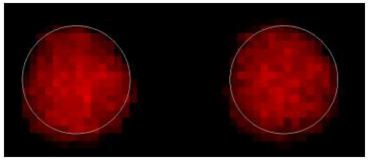
Grade IV_CJ 1519_2000153951

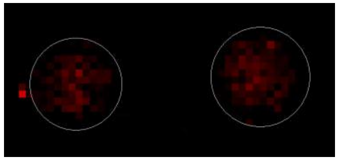
Grade IV_CH 28569_2000153799

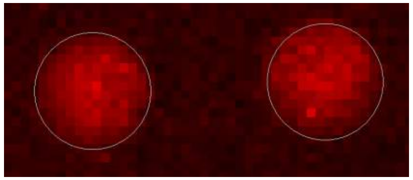
Grade IV_CF 7522_2000154010

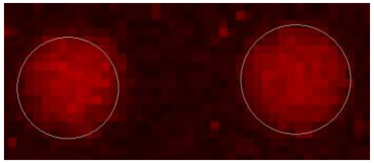
Grade IV_CH 9002_2000153954

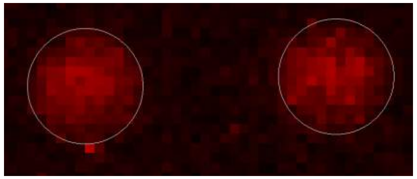
Grade IV_CH 21783_2000153785

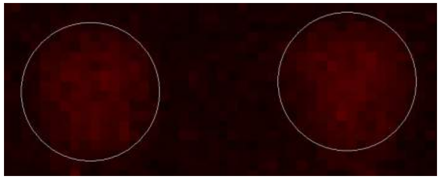
Grade IV_CH 3969_2000153950

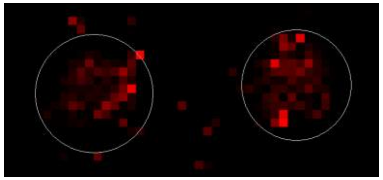
Grade IV_CH 4996_2000153933

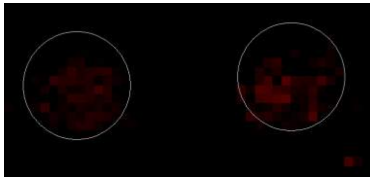
Grade IV_CF 11940_2000153779

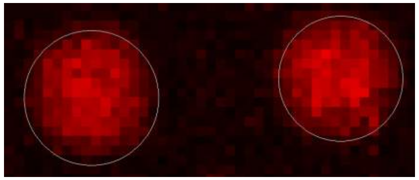
Grade IV_CH 14820_2000153796

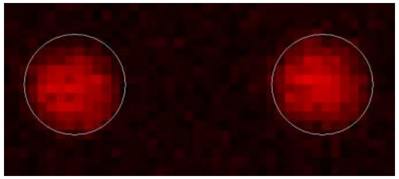
Grade IV_CH 18426_2000153757

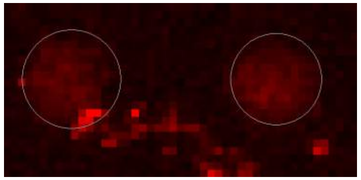
Grade IV_CH 6643_2000153758

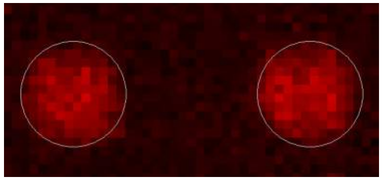
Grade IV_CH 29697_2000153759

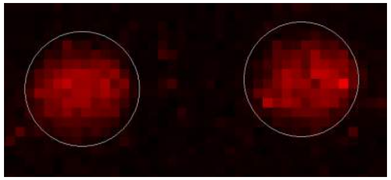
Grade IV_CF 12166_2000153934

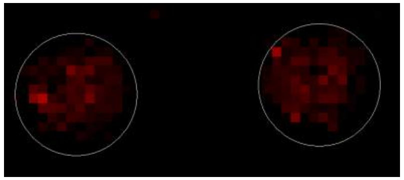
Grade IV_CH 30203_2000153793

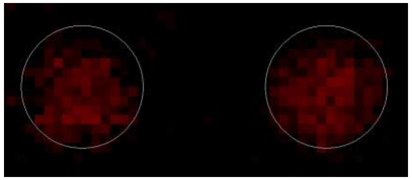
Grade IV_CH 29738_2000153798

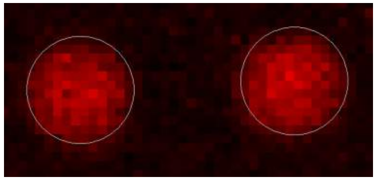
Grade IV_CJ 30014_2000153805

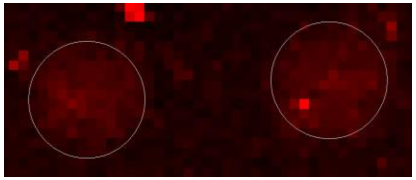
Grade IV_CH 22618_2000153782

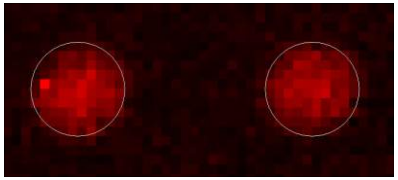
Grade IV_CJ 13655_2000153783

SVZp

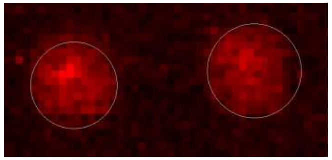
Grade IV_CJ 18704_2000153784

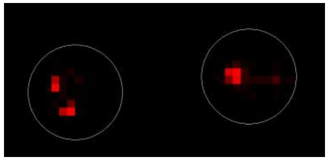
Grade IV_CF 27891_2000153776

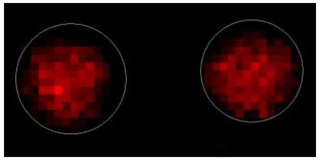
Grade IV_CJ 2041_2000154019

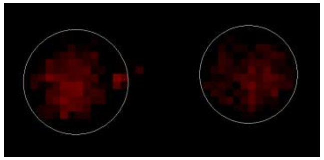
Grade IV_CH 8498_2000153777

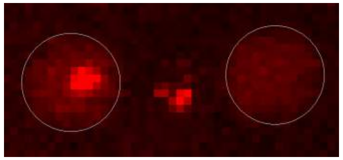
Grade IV_CJ 6254_2000153800

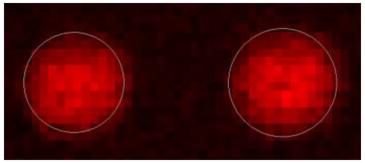
Grade IV_CH 12724_2000153778

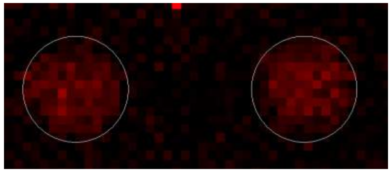
Grade IV_CJ 12441_2000153760

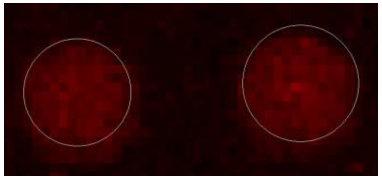
Grade IV_CF 11518_2000153942

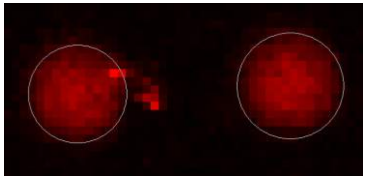
Grade IV_CH 5261_2000155722

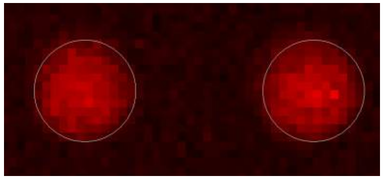
Grade IV_CF 19257 _2000155723

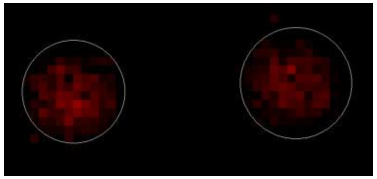
Grade IV_CH 18016_2000153791

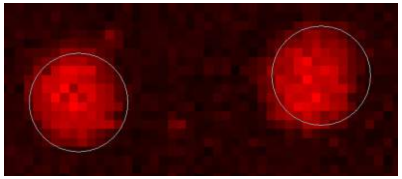
Grade IV_CJ 10048_2000155725

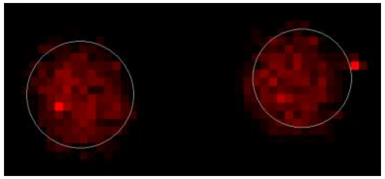
Grade IV_CJ 32705_2000153797

SVZn

NM_024482.1 GMEB1

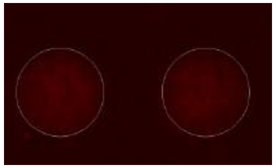
Grade IV_CF 7916_2000154021

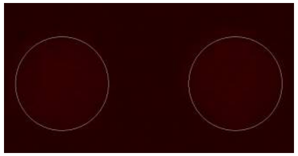
Grade IV_CJ 11291_2000154025

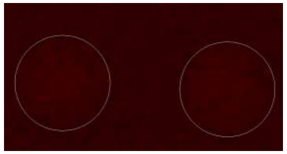
Grade IV_CJ 22776_2000153756

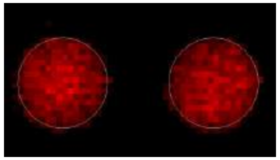
Grade IV_CJ 1519_2000153951

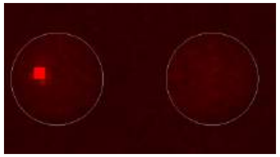
Grade IV_CH 28569_2000153799

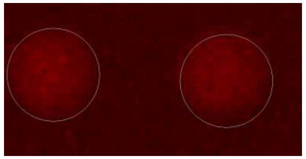
Grade IV_CF 7522_2000154010

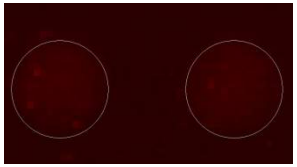
Grade IV_CH 9002_2000153954

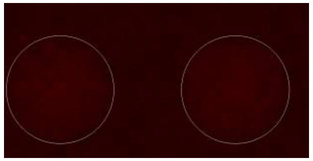
Grade IV_CH 21783_2000153785

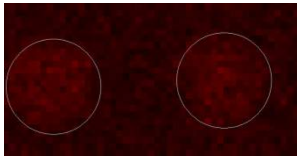
Grade IV_CH 3969_2000153950

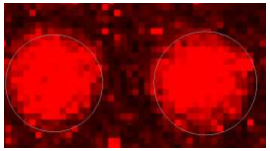
Grade IV_CH 4996_2000153933

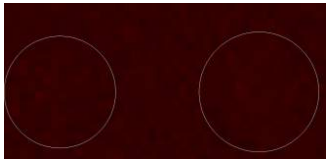
Grade IV_CF 11940_2000153779

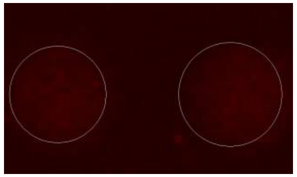
Grade IV_CH 14820_2000153796

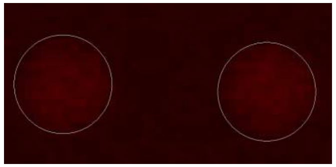
Grade IV_CH 18426_2000153757

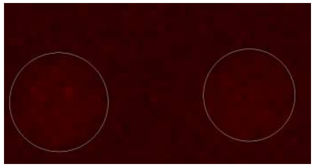
Grade IV_CH 6643_2000153758

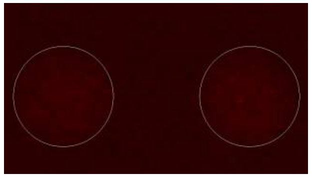
Grade IV_CH 29697_2000153759

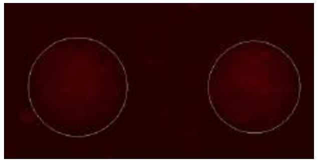
Grade IV_CF 12166_2000153934

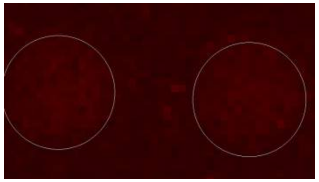
Grade IV_CH 30203_2000153793

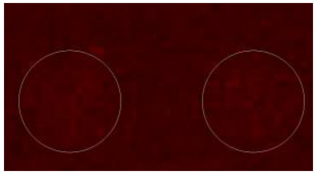
Grade IV_CH 29738_2000153798

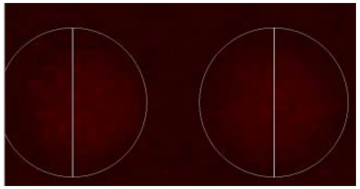
Grade IV_CJ 30014_2000153805

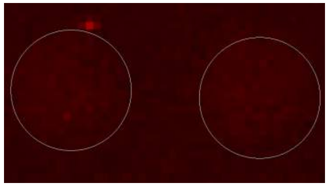
Grade IV_CH 22618_2000153782

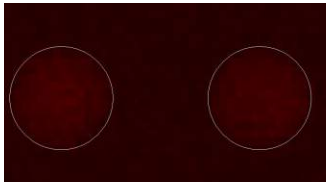
Grade IV_CJ 13655_2000153783

SVZp

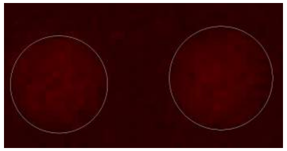
Grade IV_CJ 18704_2000153784

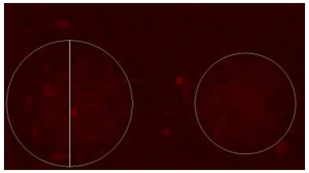
Grade IV_CF 27891_2000153776

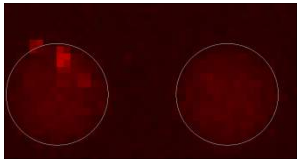
Grade IV_CJ 2041_2000154019

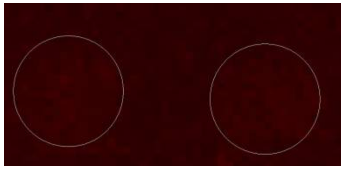
Grade IV_CH 8498_2000153777

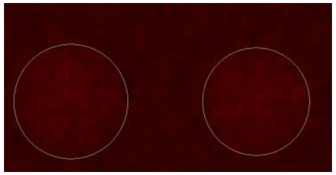
Grade IV_CJ 6254_2000153800

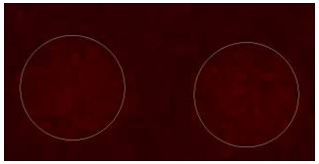
Grade IV_CH 12724_2000153778

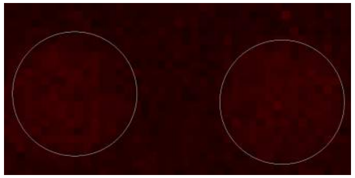
Grade IV_CJ 12441_2000153760

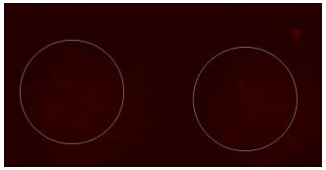
Grade IV_CF 11518_2000153942

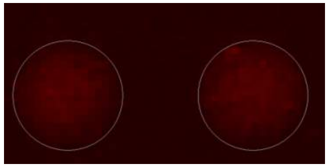
Grade IV_CH 5261_2000155722

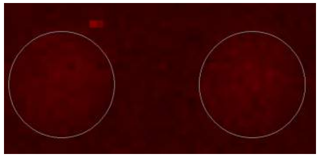
Grade IV_CF 19257 _2000155723

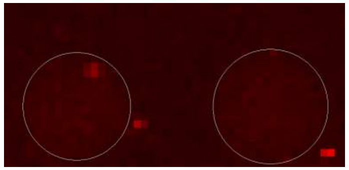
Grade IV_CH 18016_2000153791

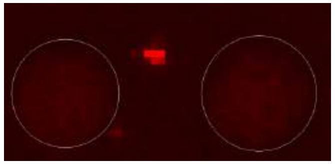
Grade IV_CJ 10048_2000155725

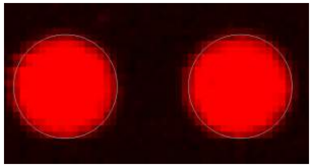
Grade IV_CJ 32705_2000153797

SVZn

BC000267.1 GPBP1

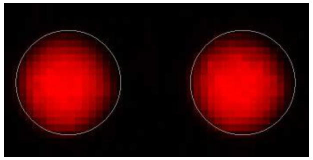
Grade IV_CF 7916_2000154021

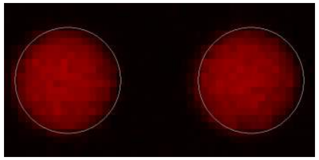
Grade IV_CJ 11291_2000154025

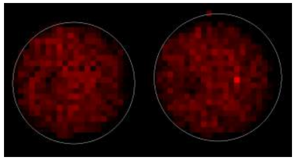
Grade IV_CJ 22776_2000153756

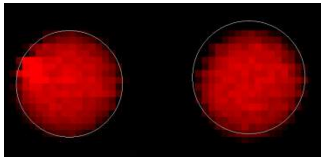
Grade IV_CJ 1519_2000153951

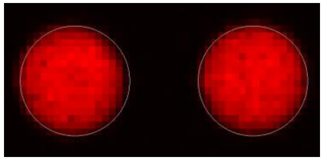
Grade IV_CH 28569_2000153799

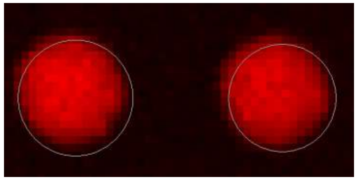
Grade IV_CF 7522_2000154010

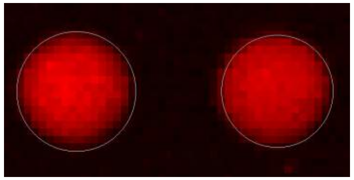
Grade IV_CH 9002_2000153954

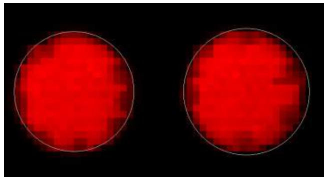
Grade IV_CH 21783_2000153785

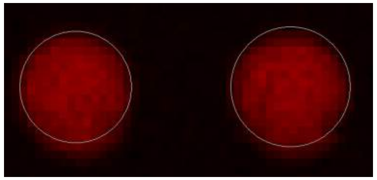
Grade IV_CH 3969_2000153950

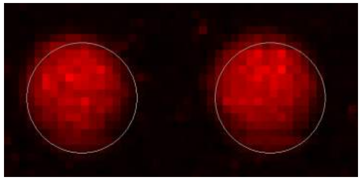
Grade IV_CH 4996_2000153933

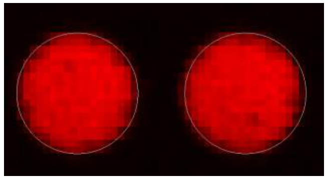
Grade IV_CF 11940_2000153779

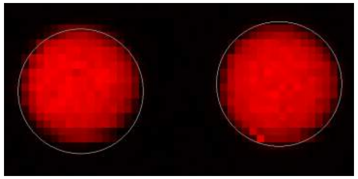
Grade IV_CH 14820_2000153796

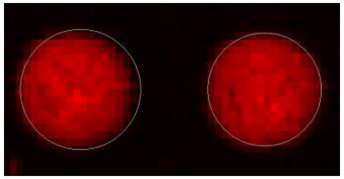
Grade IV_CH 18426_2000153757

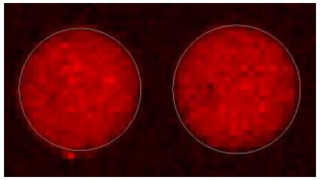
Grade IV_CH 6643_2000153758

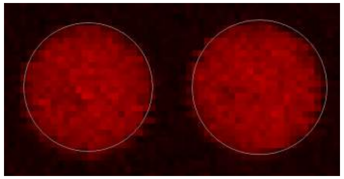
Grade IV_CH 29697_2000153759

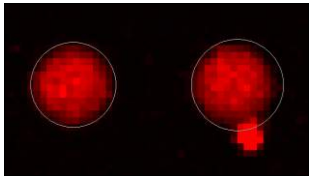
Grade IV_CF 12166_2000153934

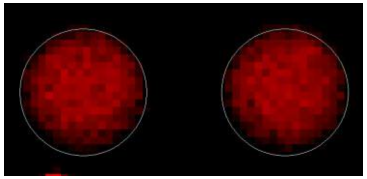
Grade IV_CH 30203_2000153793

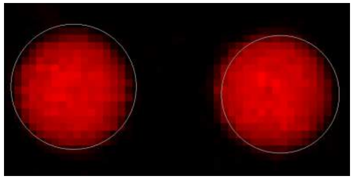
Grade IV_CH 29738_2000153798

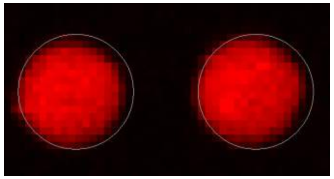
Grade IV_CJ 30014_2000153805

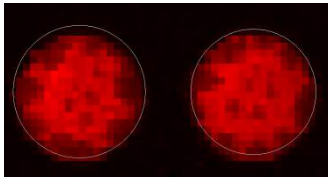
Grade IV_CH 22618_2000153782

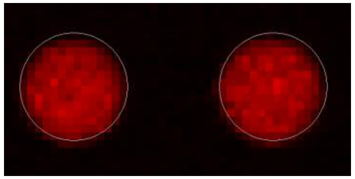
Grade IV_CJ 13655_2000153783

SVZp

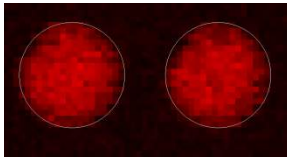
Grade IV_CJ 18704_2000153784

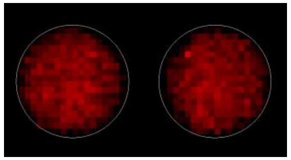
Grade IV_CF 27891_2000153776

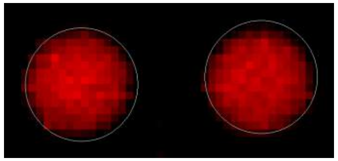
Grade IV_CJ 2041_2000154019

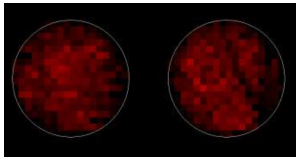
Grade IV_CH 8498_2000153777

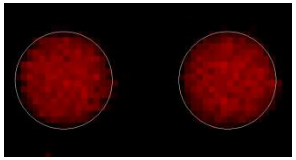
Grade IV_CJ 6254_2000153800

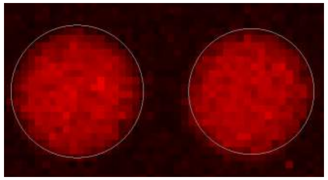
Grade IV_CH 12724_2000153778

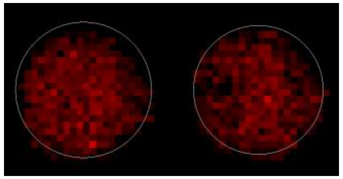
Grade IV_CJ 12441_2000153760

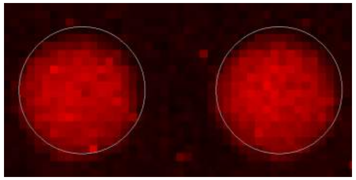
Grade IV_CF 11518_2000153942

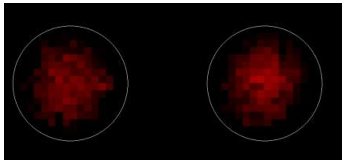
Grade IV_CH 5261_2000155722

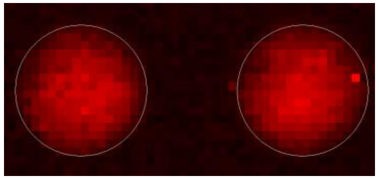
Grade IV_CF 19257 _2000155723

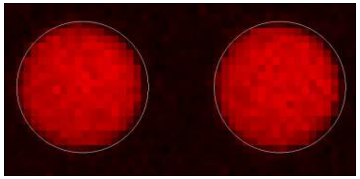
Grade IV_CH 18016_2000153791

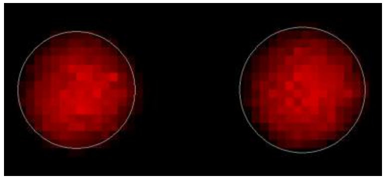
Grade IV_CJ 10048_2000155725

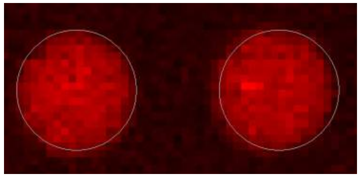
Grade IV_CJ 32705_2000153797

SVZn

NM_152740.2 HIBADH

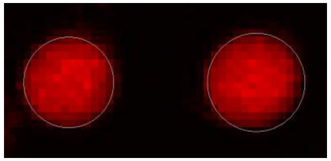
Grade IV_CF 7916_2000154021

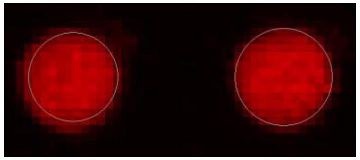
Grade IV_CJ 11291_2000154025

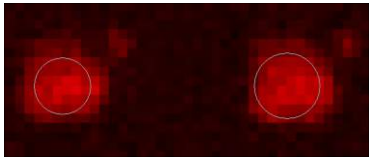
Grade IV_CJ 22776_2000153756

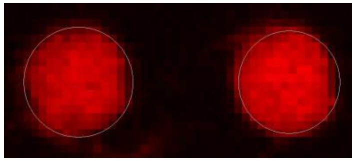
Grade IV_CJ 1519_2000153951

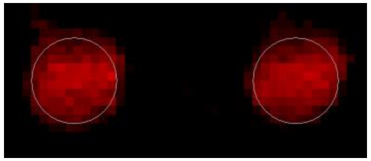
Grade IV_CH 28569_2000153799

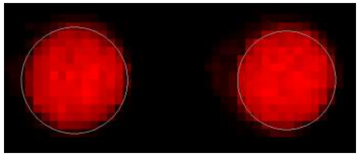
Grade IV_CF 7522_2000154010

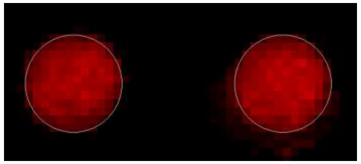
Grade IV_CH 9002_2000153954

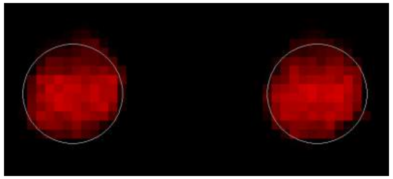
Grade IV_CH 21783_2000153785

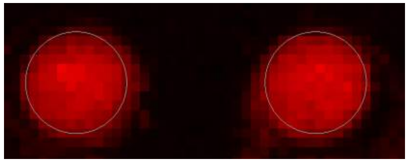
Grade IV_CH 3969_2000153950

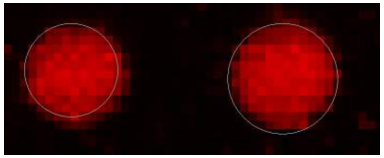
Grade IV_CH 4996_2000153933

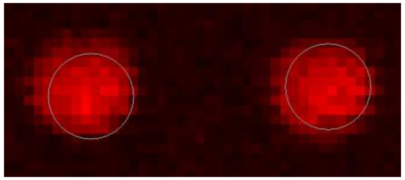
Grade IV_CF 11940_2000153779

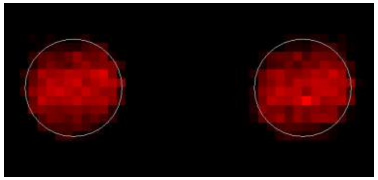
Grade IV_CH 14820_2000153796

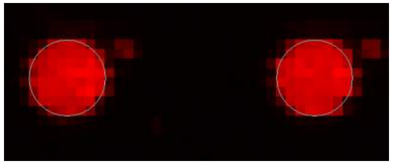
Grade IV_CH 18426_2000153757

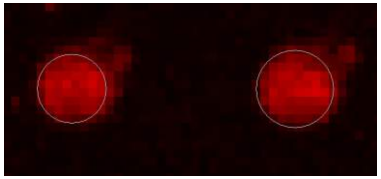
Grade IV_CH 6643_2000153758

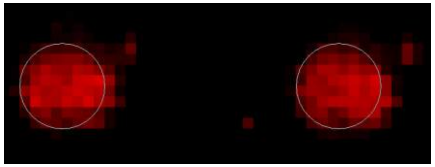
Grade IV_CH 29697_2000153759

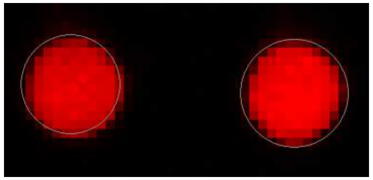
Grade IV_CF 12166_2000153934

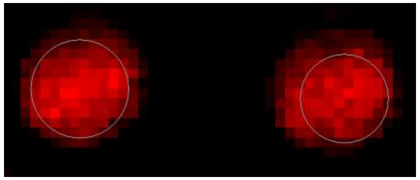
Grade IV_CH 30203_2000153793

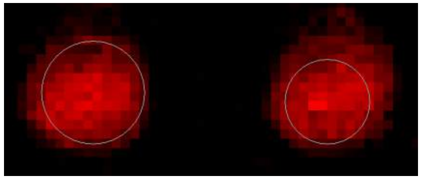
Grade IV_CH 29738_2000153798

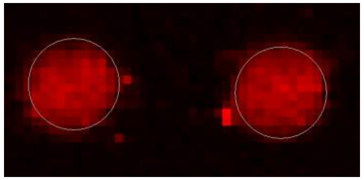
Grade IV_CJ 30014_2000153805

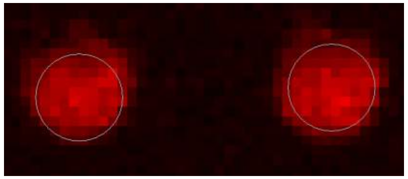
Grade IV_CH 22618_2000153782

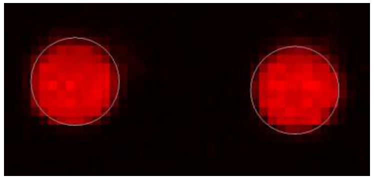
Grade IV_CJ 13655_2000153783

SVZp

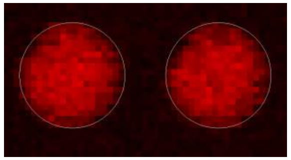

Grade IV_CJ 18704_2000153784

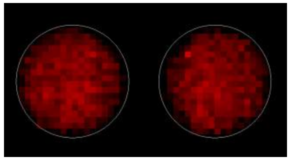

Grade IV_CF 27891_2000153776

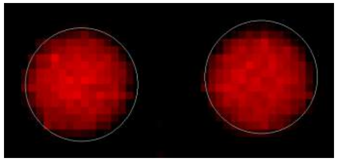

Grade IV_CJ 2041_2000154019

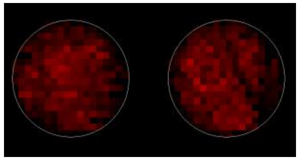

Grade IV_CH 8498_2000153777

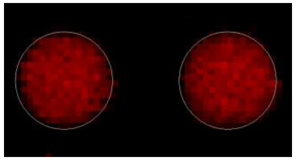

Grade IV_CJ 6254_2000153800

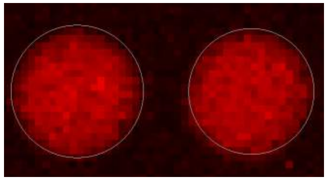

Grade IV_CH 12724_2000153778

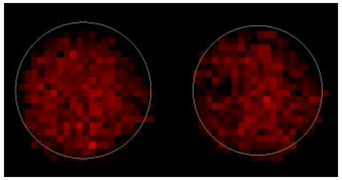

Grade IV_CJ 12441_2000153760

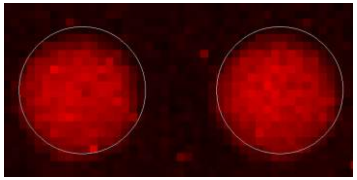

Grade IV_CF 11518_2000153942

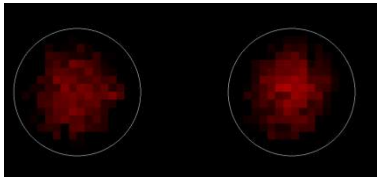

Grade IV_CH 5261_2000155722

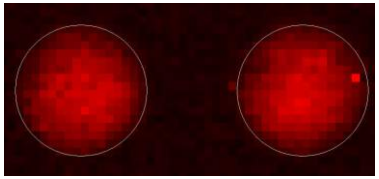

Grade IV_CF 19257 _2000155723

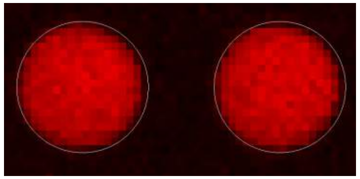

Grade IV_CH 18016_2000153791

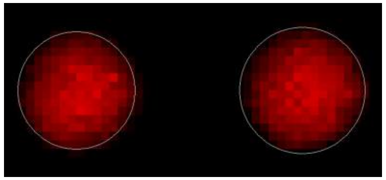

Grade IV_CJ 10048_2000155725

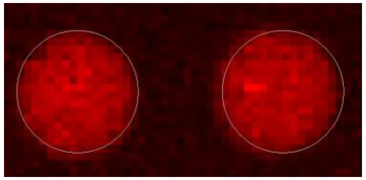

Grade IV_CJ 32705_2000153797

SVZn

BCO17810.1_TMOD4

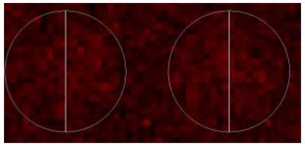
Grade IV_CF 7916_2000154021

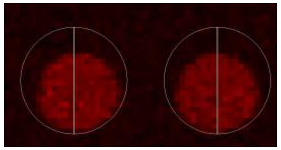
Grade IV_CJ 11291_2000154025

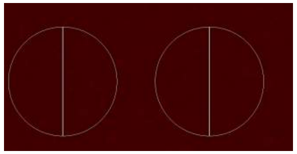
Grade IV_CJ 22776_2000153756

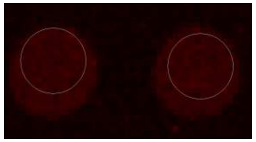
Grade IV_CJ 1519_2000153951

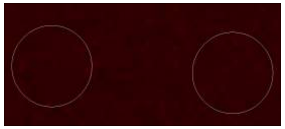
Grade IV_CH 28569_2000153799

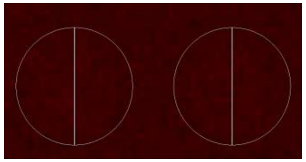
Grade IV_CF 7522_2000154010

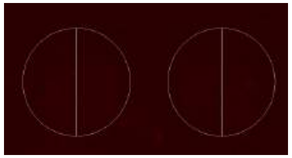
Grade IV_CH 9002_2000153954

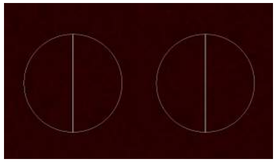
Grade IV_CH 21783_2000153785

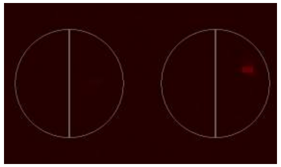
Grade IV_CH 3969_2000153950

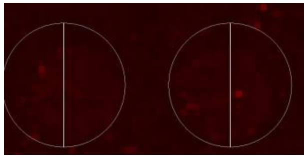
Grade IV_CH 4996_2000153933

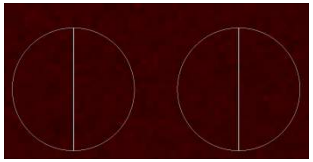
Grade IV_CF 11940_2000153779

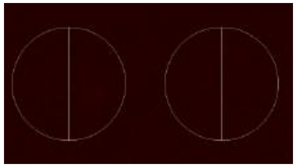
Grade IV_CH 14820_2000153796

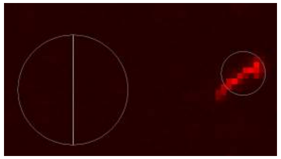
Grade IV_CH 18426_2000153757

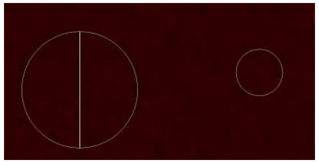
Grade IV_CH 6643_2000153758

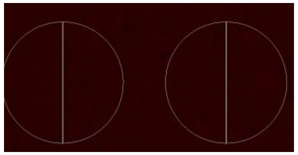
Grade IV_CH 29697_2000153759

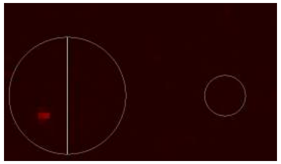
Grade IV_CF 12166_2000153934

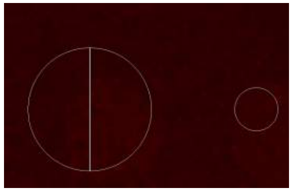
Grade IV_CH 30203_2000153793

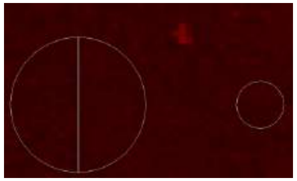
Grade IV_CH 29738_2000153798

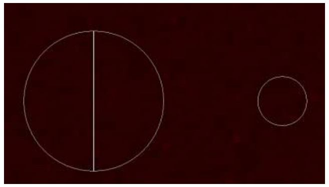
Grade IV_CJ 30014_2000153805

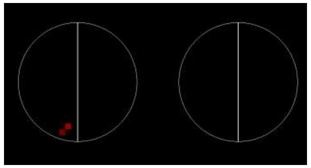
Grade IV_CH 22618_2000153782

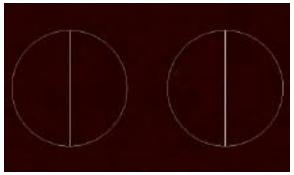
Grade IV_CJ 13655_2000153783

SVZp

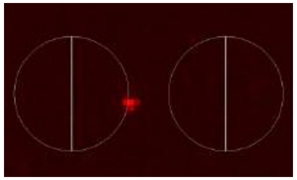
Grade IV_CJ 18704_2000153784

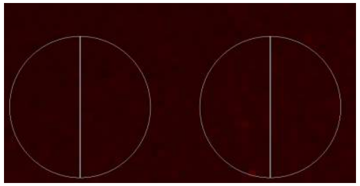
Grade IV_CF 27891_2000153776

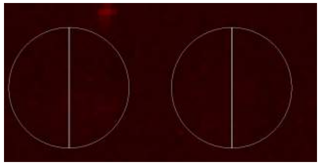
Grade IV_CJ 2041_2000154019

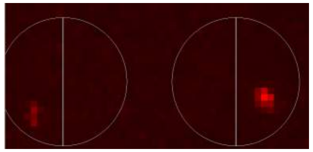
Grade IV_CH 8498_2000153777

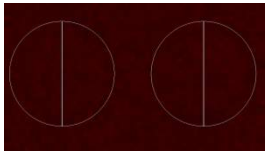
Grade IV_CJ 6254_2000153800

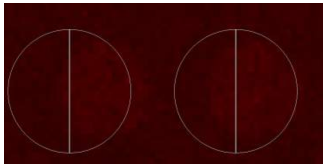
Grade IV_CH 12724_2000153778

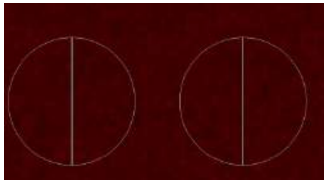
Grade IV_CJ 12441_2000153760

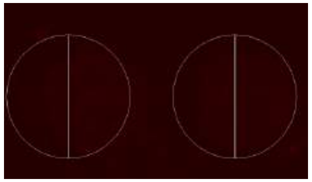
Grade IV_CF 11518_2000153942

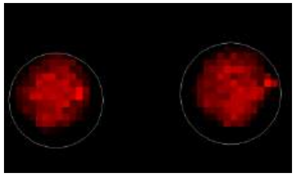
Grade IV_CH 5261_2000155722

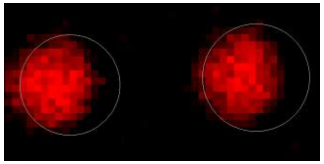
Grade IV_CF 19257_2000155723

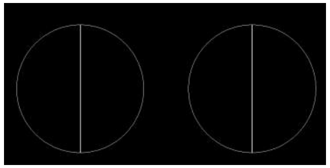
Grade IV_CH 18016_2000153791

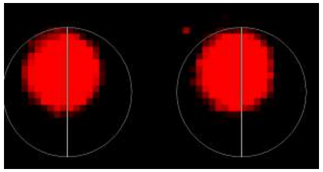
Grade IV_CJ 10048_2000155725

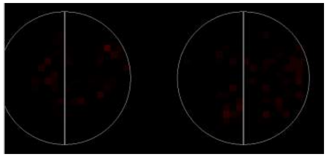
Grade IV_CJ 32705_2000153797

SVZn

BC094687.1_EEF1A1

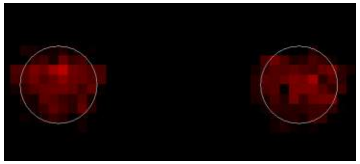
Grade IV_CF 7916_2000154021

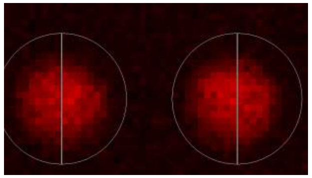
Grade IV_CJ 11291_2000154025

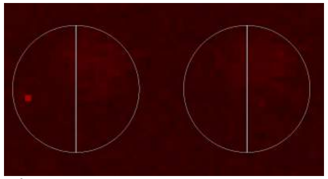
Grade IV_CJ 22776_2000153756

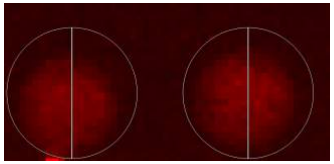
Grade IV_CJ 1519_2000153951

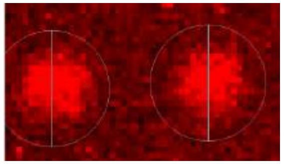
Grade IV_CH 28569_2000153799

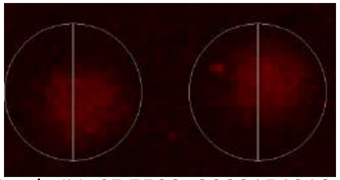
Grade IV_CF 7522_2000154010

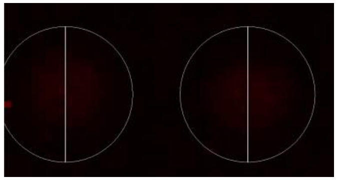
Grade IV_CH 9002_2000153954

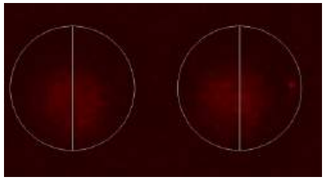
Grade IV_CH 21783_2000153785

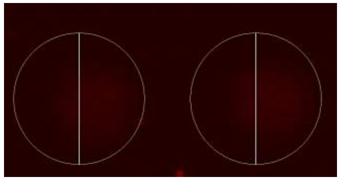
Grade IV_CH 3969_2000153950

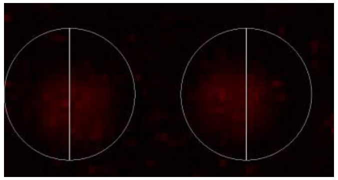
Grade IV_CH 4996_2000153933

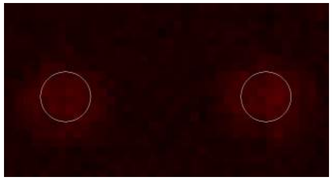
Grade IV_CF 11940_2000153779

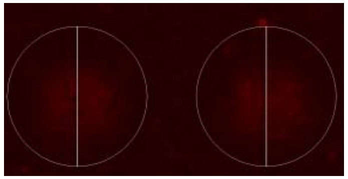
Grade IV_CH 14820_2000153796

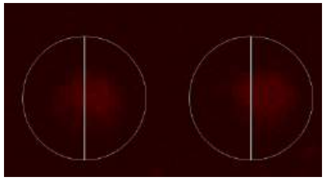
Grade IV_CH 18426_2000153757

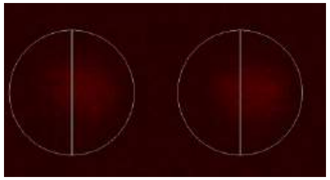
Grade IV_CH 6643_2000153758

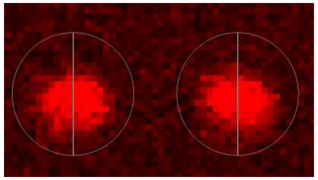
Grade IV_CH 29697_2000153759

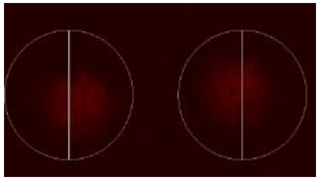
Grade IV_CF 12166_2000153934

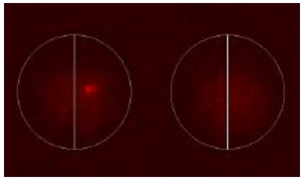
Grade IV_CH 30203_2000153793

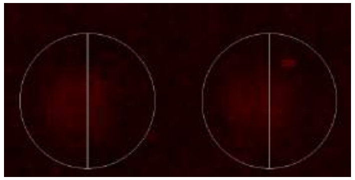
Grade IV_CH 29738_2000153798

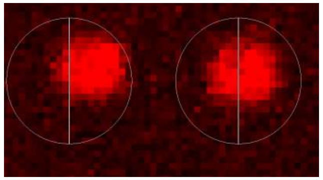
Grade IV_CJ 30014_2000153805

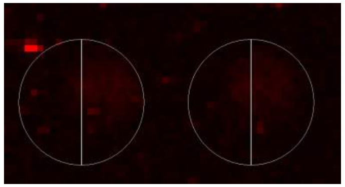
Grade IV_CH 22618_2000153782

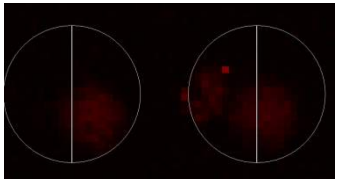
Grade IV_CJ 13655_2000153783

SVZp

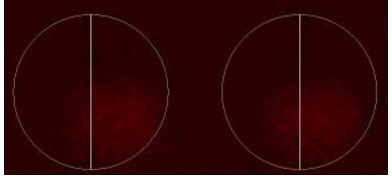
Grade IV_CJ 18704_2000153784

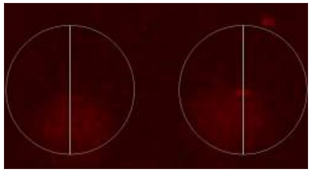
Grade IV_CF 27891_2000153776

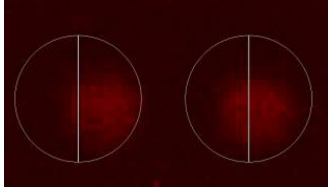
Grade IV_CJ 2041_2000154019

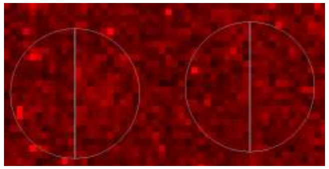
Grade IV_CH 8498_2000153777

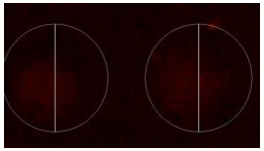
Grade IV_CJ 6254_2000153800

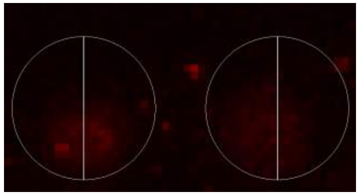
Grade IV_CH 12724_2000153778

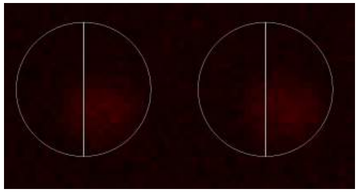
Grade IV_CJ 12441_2000153760

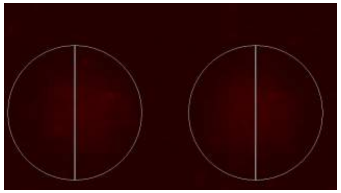
Grade IV_CF 11518_2000153942

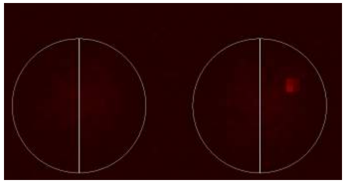
Grade IV_CH 5261_2000155722

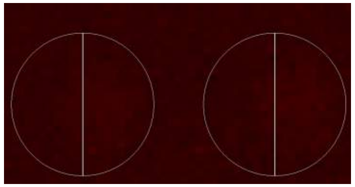
Grade IV_CF 19257_2000155723

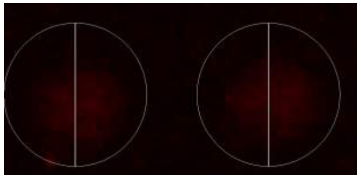
Grade IV_CH 18016_2000153791

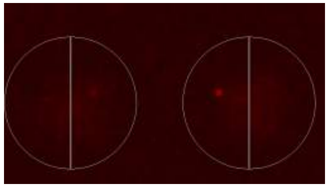
Grade IV_CJ 10048_2000155725

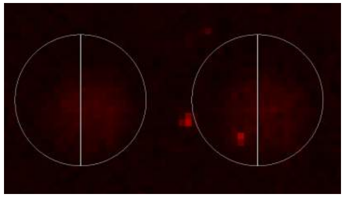
Grade IV_CJ 32705_2000153797

SVZn

XM_379060.1_LOC339685

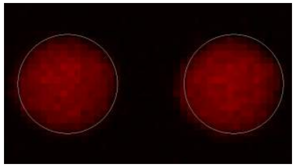
Grade IV_CF 7916_2000154021

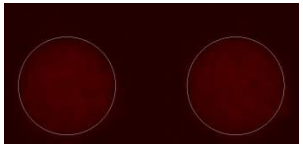
Grade IV_CJ 11291_2000154025

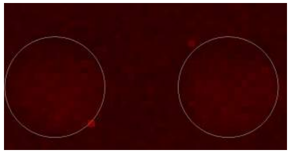
Grade IV_CJ 22776_2000153756

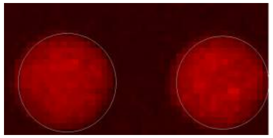
Grade IV_CJ 1519_2000153951

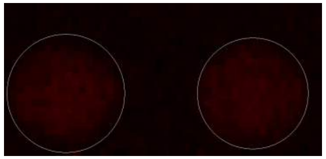
Grade IV_CH 28569_2000153799

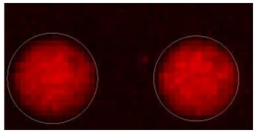
Grade IV_CF 7522_2000154010

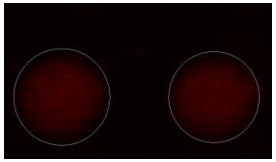
Grade IV_CH 9002_2000153954

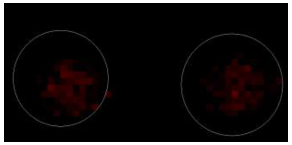
Grade IV_CH 21783_2000153785

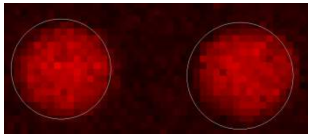
Grade IV_CH 3969_2000153950

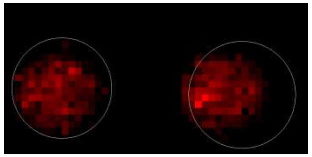
Grade IV_CH 4996_2000153933

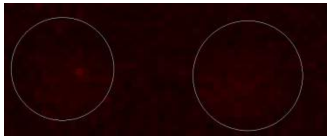
Grade IV_CF 11940_2000153779

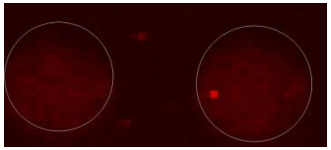
Grade IV_CH 14820_2000153796

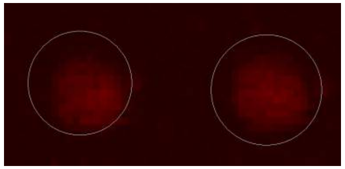
Grade IV_CH 18426_2000153757

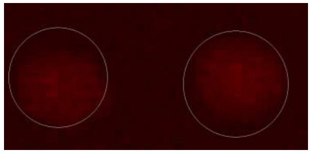
Grade IV_CH 6643_2000153758

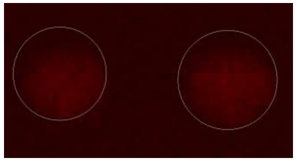
Grade IV_CH 29697_2000153759

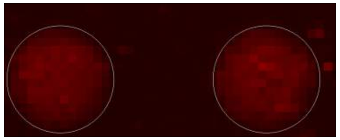
Grade IV_CF 12166_2000153934

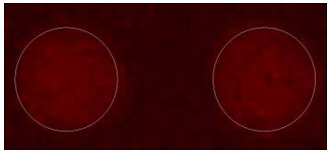
Grade IV_CH 30203_2000153793

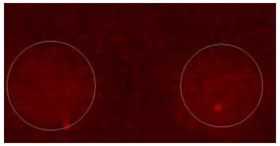
Grade IV_CH 29738_2000153798

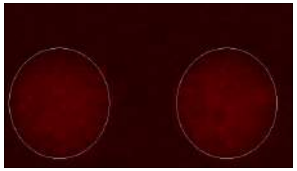
Grade IV_CJ 30014_2000153805

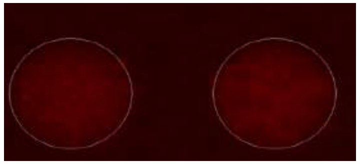
Grade IV_CH 22618_2000153782

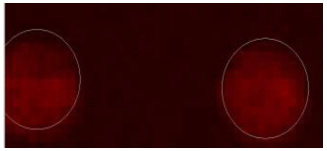
Grade IV_CJ 13655_2000153783

SVZp

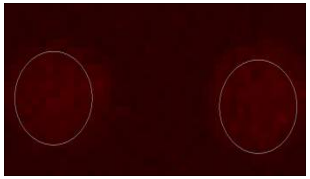

Grade IV_CJ 18704_2000153784

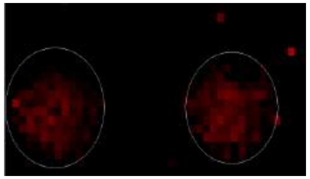

Grade IV_CF 27891_2000153776

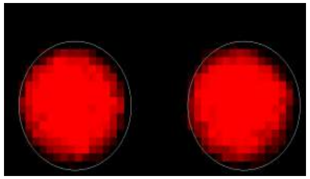

Grade IV_CJ 2041_2000154019

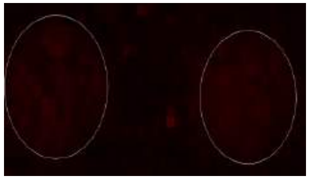

Grade IV_CH 8498_2000153777

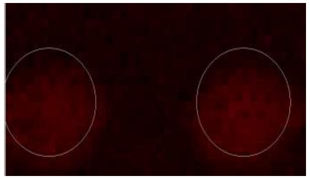

Grade IV_CJ 6254_2000153800

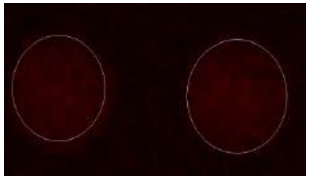

Grade IV_CH 12724_2000153778

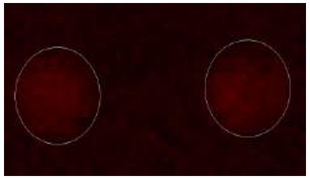

Grade IV_CJ 12441_2000153760

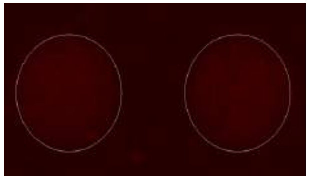

Grade IV_CF 11518_2000153942

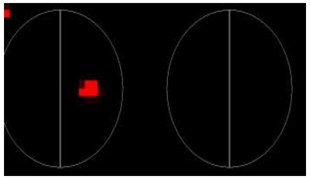

Grade IV_CH 5261_2000155722

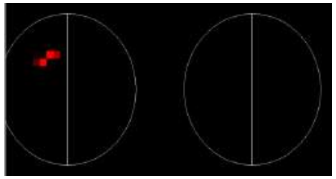

Grade IV_CF 19257_2000155723

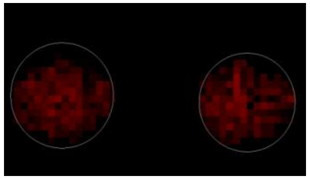

Grade IV_CH 18016_2000153791

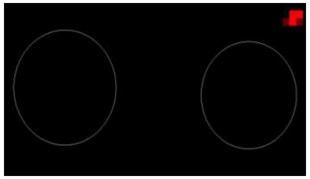

Grade IV_CJ 10048_2000155725

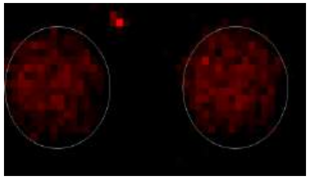

Grade IV_CJ 32705_2000153797

SVZn

Grade II_IDH1p Vs Grade II_Wild Type IDH1

BC000846.2 (CIB1) – IDH1p

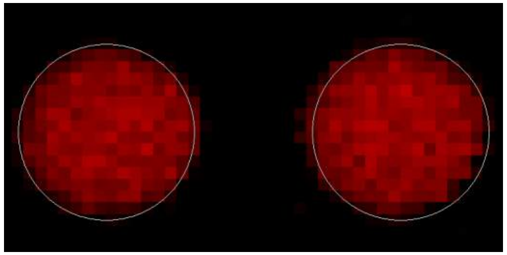

Grade II_CF 29538_2000154020

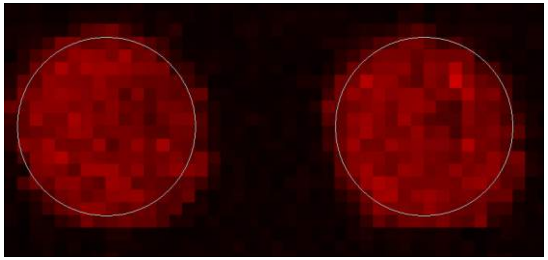

Grade II_CJ 33350_2000144452

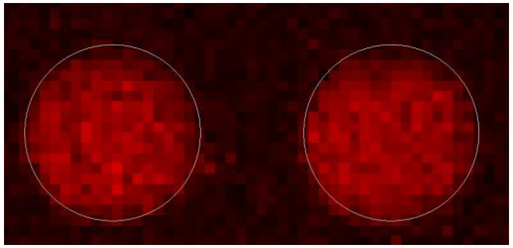

Grade II_CJ 4825_2000143981

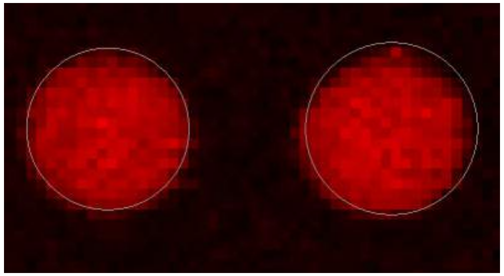

Grade II_CJ 7132_2000154028

BC000846.2 (CIB1) – Wild Type

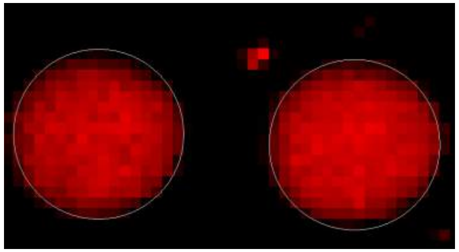

Grade II_CF 10729_2000154012

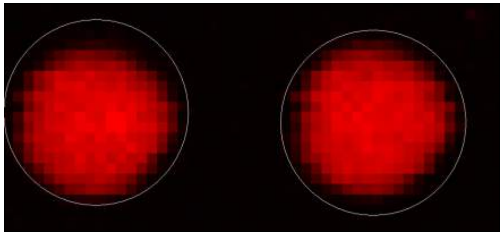

Grade II_CF 20468_2000153947

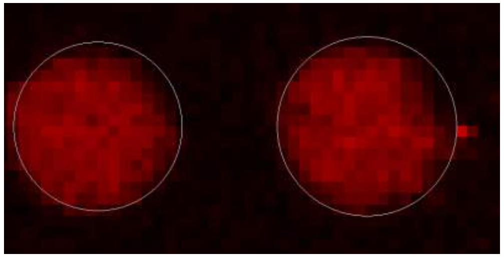

Grade II_CJ 10242_2000144449_2

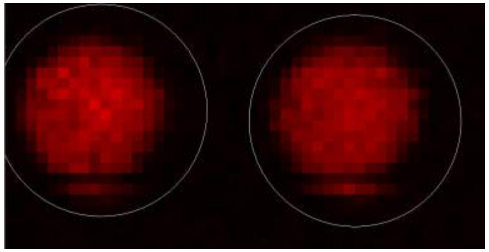

Grade II_CK 40_2000144445

NM_017735.3 (TTC27) - IDH1p

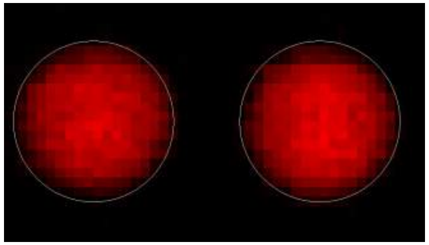

Grade II_CF 29538_2000154020

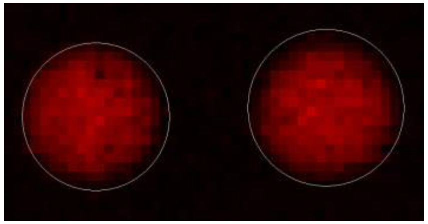

Grade II_CJ 33350_2000144452

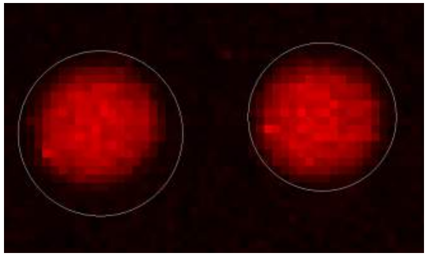

Grade II_CJ 4825_2000143981

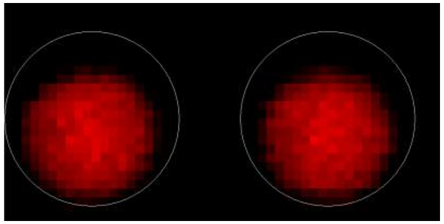

Grade II_CJ 7132_2000154028

NM_017735.3 (TTC27) – Wild Type

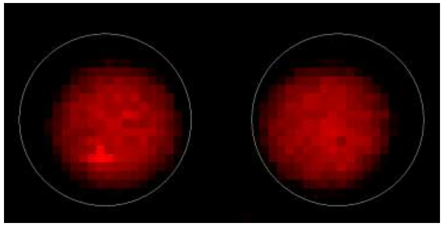

Grade II_CF 10729_2000154012

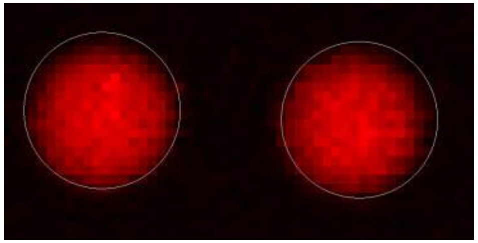

Grade II_CF 20468_2000153947

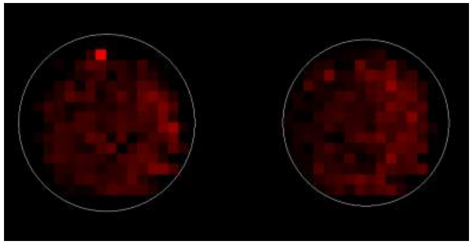

Grade II_CJ 10242_2000144449_2

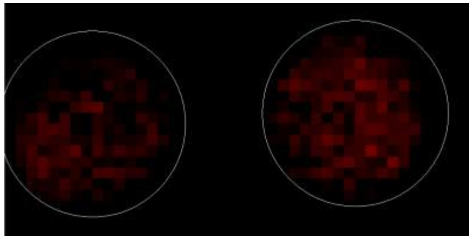

Grade II_CK 40_2000144445

BC034483.1 (HSPAIL) - IDH1p

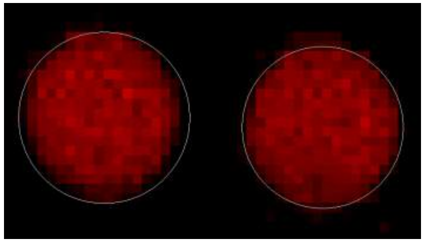

Grade II_CF 29538_2000154020

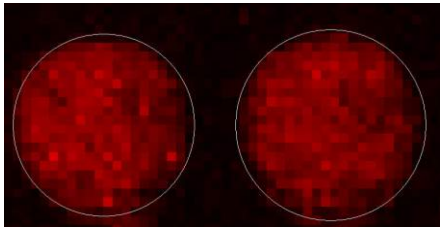

Grade II_CJ 33350_2000144452

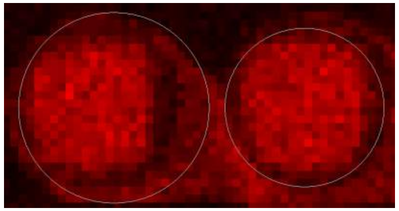

Grade II_CJ 4825_2000143981

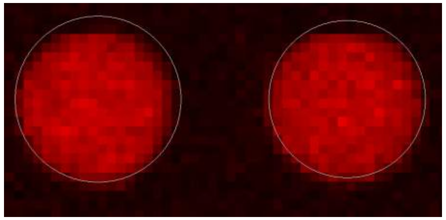

Grade II_CJ 7132_2000154028

BC034483.1 (HSPAIL) – Wild Type

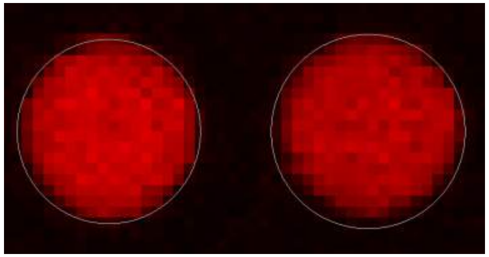

Grade II_CF 10729_2000154012

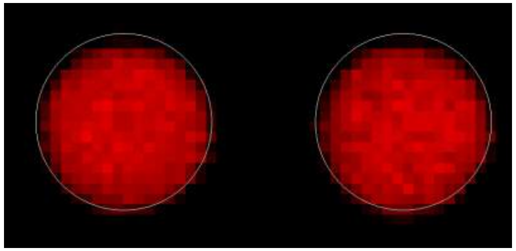

Grade II_CF 20468_2000153947

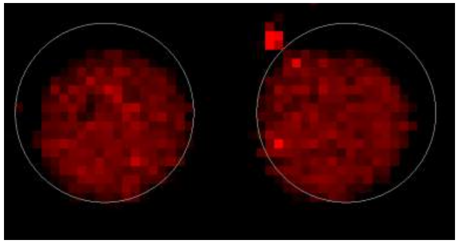

Grade II_CJ 10242_2000144449_2

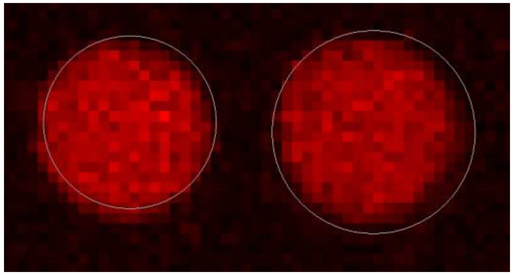

Grade II_CK 40_2000144445

ENST00000389266 (GARS) – IDH1p

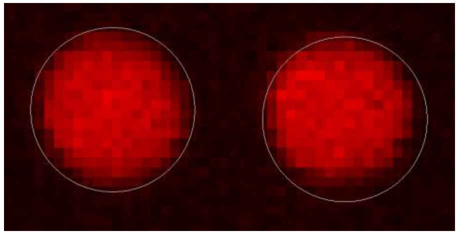

Grade II_CF 29538_2000154020

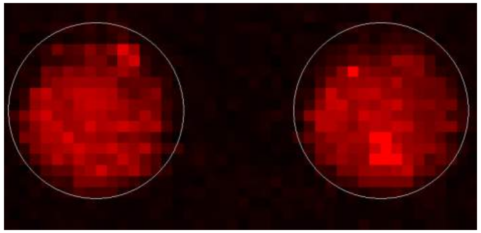

Grade II_CJ 33350_2000144452

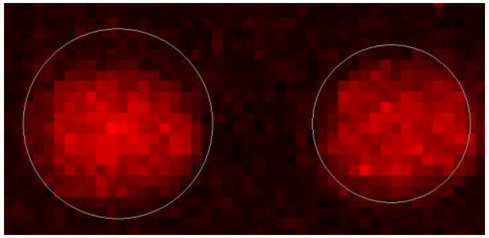

Grade II_CJ 4825_2000143981

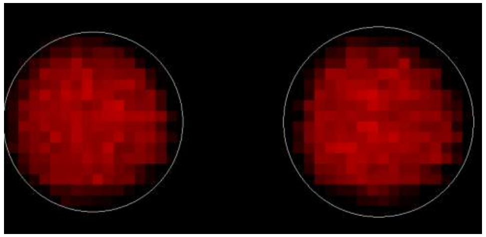

Grade II_CJ 7132_2000154028

ENST00000389266 (GARS) – Wild Type

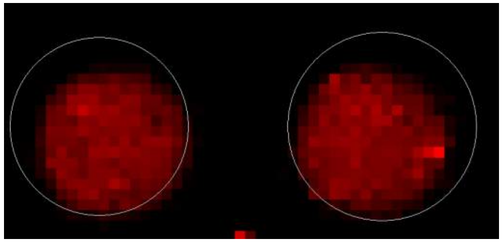

Grade II_CF 10729_2000154012

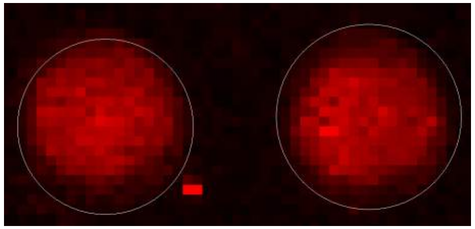

Grade II_CF 20468_2000153947

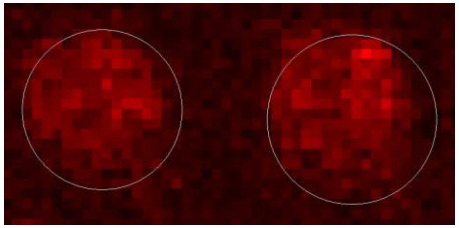

Grade II_CJ 10242_2000144449_2

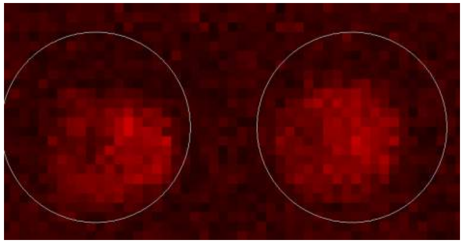

Grade II_CK 40_2000144445

BC026060.2 (CAMKK2) – IDH1p

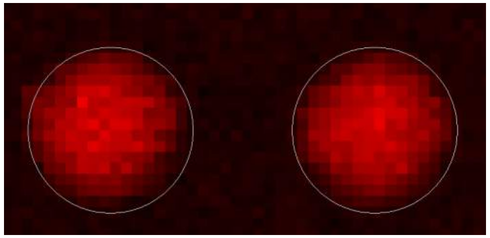

Grade II_CF 29538_2000154020

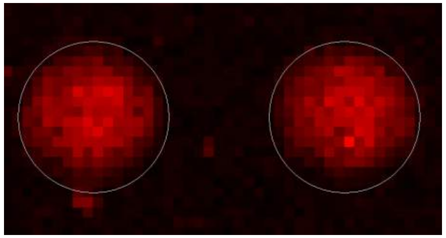

Grade II_CJ 33350_2000144452

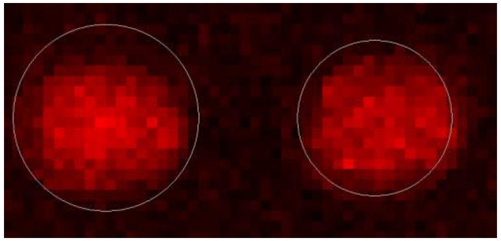

Grade II_CJ 4825_2000143981

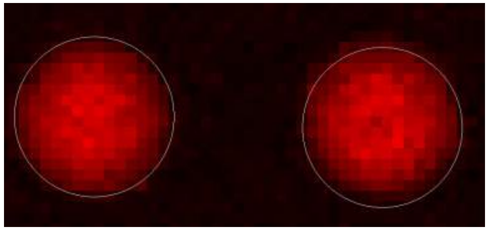

Grade II_CJ 7132_2000154028

BC026060.2 (CAMKK2) – Wild Type

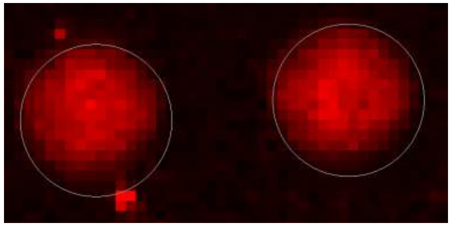

Grade II_CF 10729_2000154012

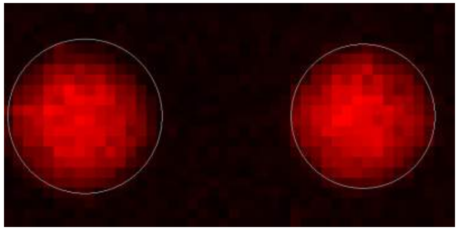

Grade II_CF 20468_2000153947

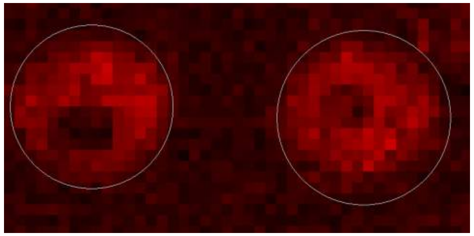

Grade II_CJ 10242_2000144449_2

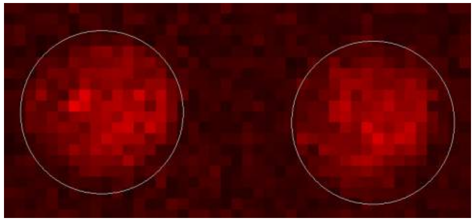

Grade II_CK 40_2000144445

NM_016401.2 (C11ORF73) – IDH1p

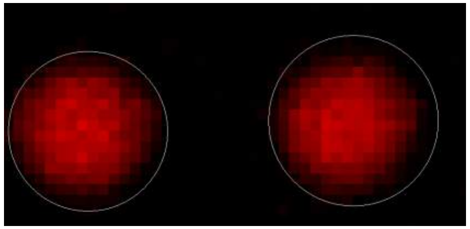

Grade II_CF 29538_2000154020

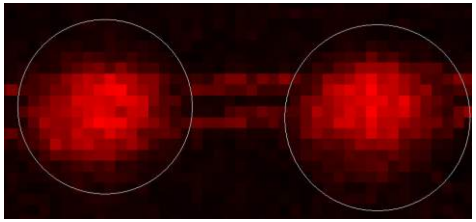

Grade II_CJ 33350_2000144452

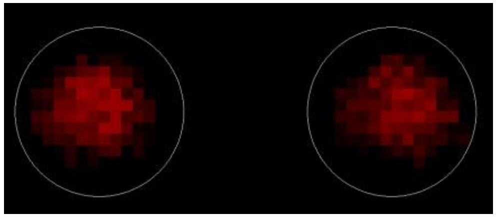

Grade II_CJ 4825_2000143981

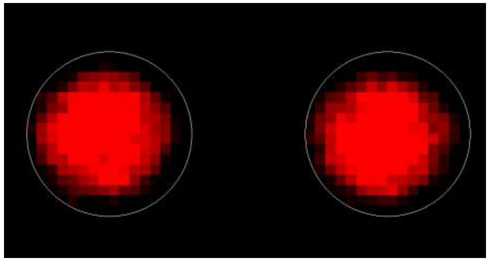

Grade II_CJ 7132_2000154028

NM_016401.2 (C11ORF73) – Wild Type

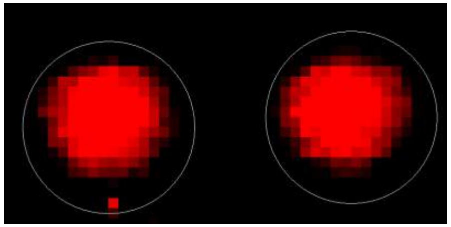

Grade II_CF 10729_2000154012

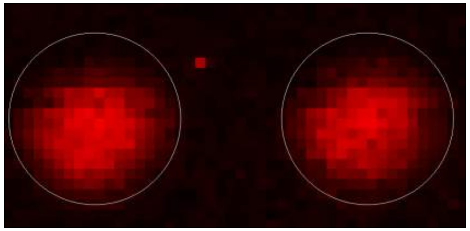

Grade II_CF 20468_2000153947

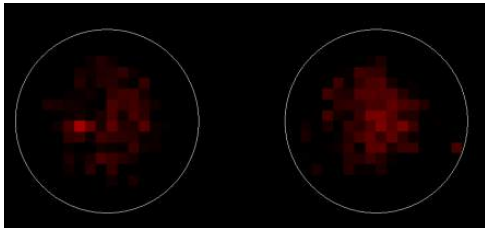

Grade II_CJ 10242_2000144449_2

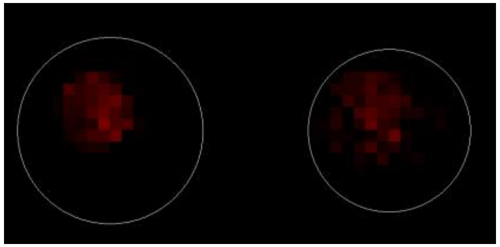

Grade II_CK 40_2000144445

NM_001888.2 (CRYM) – IDH1p

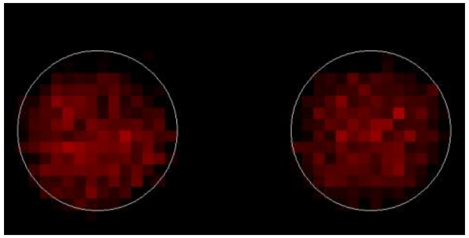

Grade II_CF 29538_2000154020

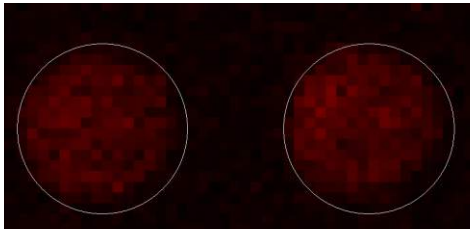

Grade II_CJ 33350_2000144452

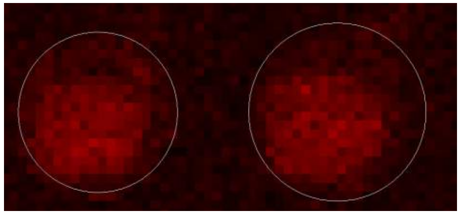

Grade II_CJ 4825_2000143981

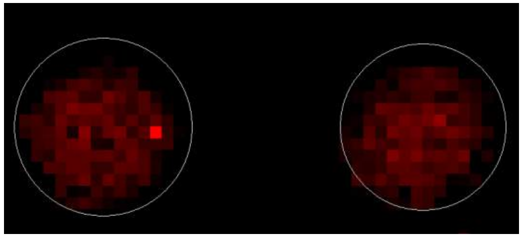

Grade II_CJ 7132_2000154028

NM_001888.2 (CRYM) – Wild Type

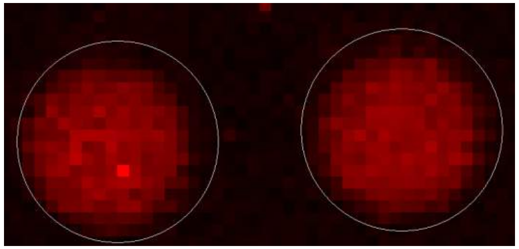
Grade II_CF 10729_2000154012

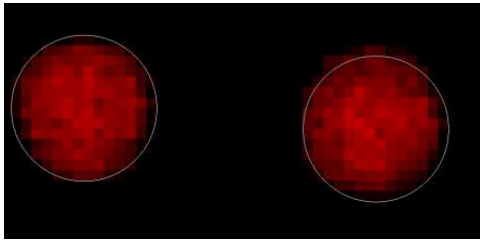
Grade II_CF 20468_2000153947

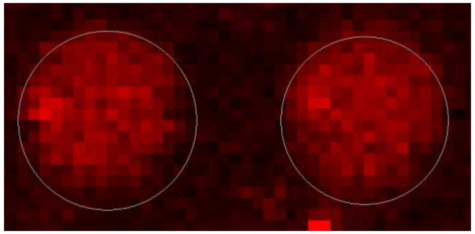
Grade II_CJ 10242_2000144449_2

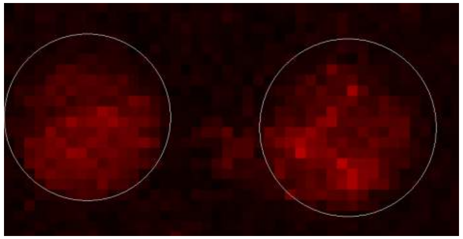
Grade II_CK 40_2000144445

NM_007026.1 (DUSP14) – IDH1p

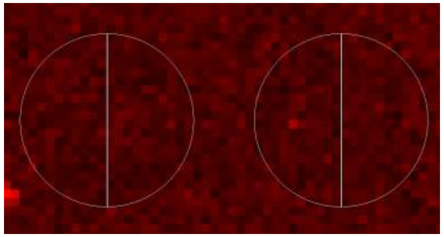

Grade II_CF 29538_2000154020

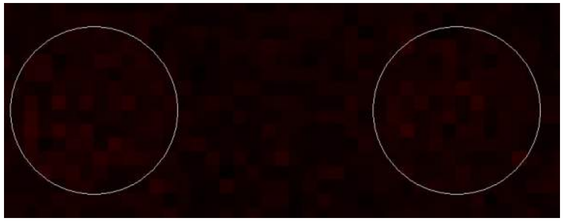

Grade II_CJ 33350_2000144452

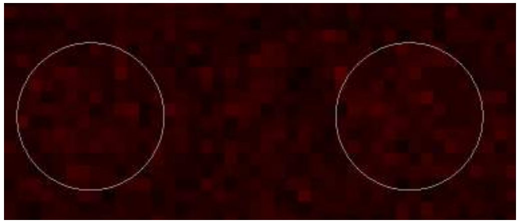

Grade II_CJ 4825_2000143981

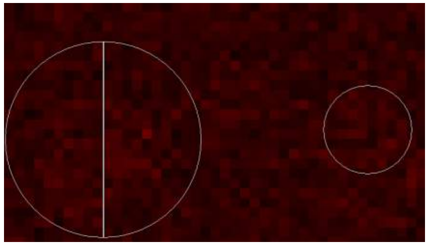

Grade II_CJ 7132_2000154028

NM_007026.1 (DUSP14) – Wild Type

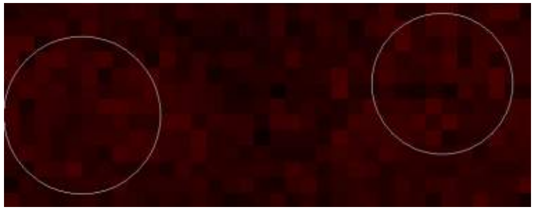

Grade II_CF 10729_2000154012

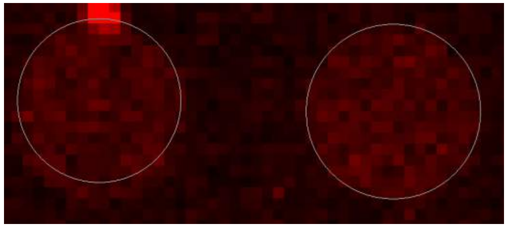

Grade II_CF 20468_2000153947

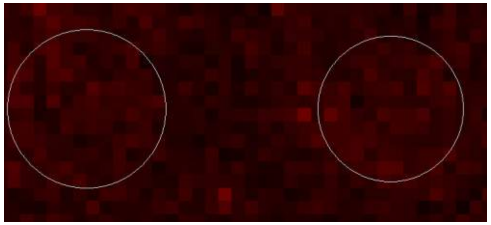

Grade II_CJ 10242_2000144449_2

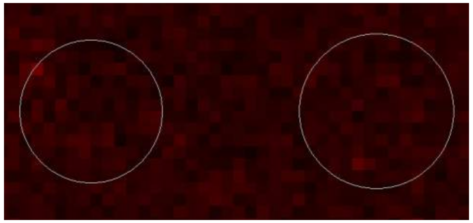

Grade II_CK 40_2000144445

NM_005861.2 (STUB1) – IDH1p

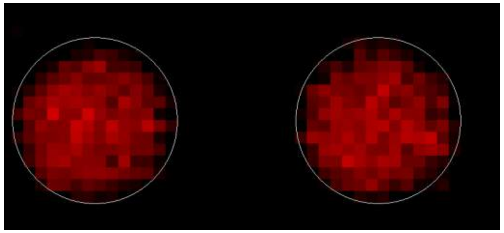

Grade II_CF 29538_2000154020

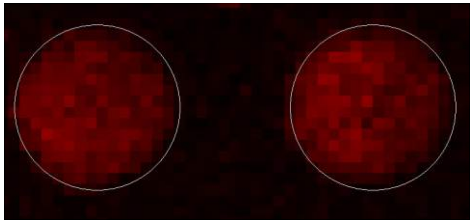

Grade II_CJ 33350_2000144452

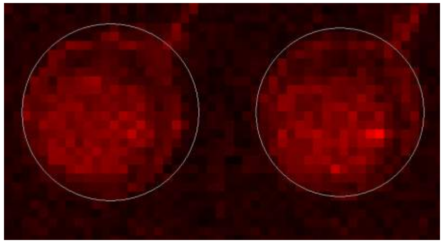

Grade II_CJ 4825_2000143981

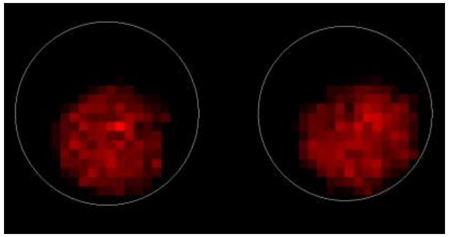

Grade II_CJ 7132_2000154028

NM_005861.2 (STUB1) – Wild Type

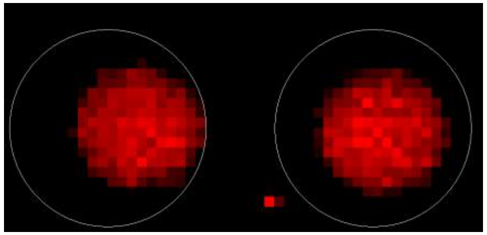

Grade II_CF 10729_2000154012

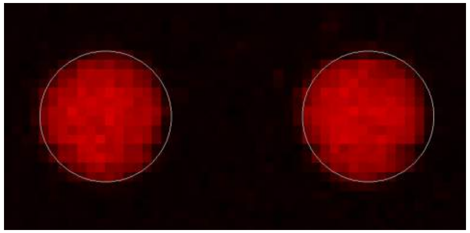

Grade II_CF 20468_2000153947

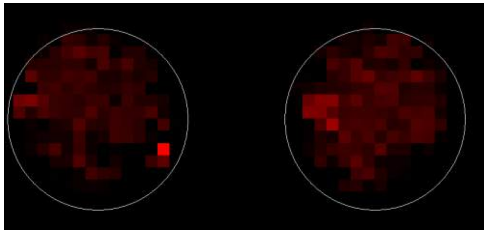

Grade II_CJ 10242_2000144449_2

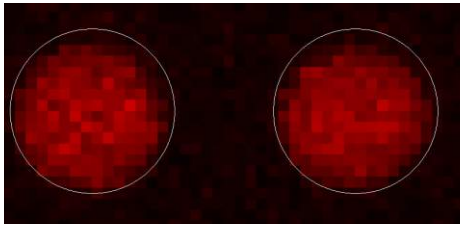

Grade II_CK 40_2000144445

NM_000519.3 (HBD) – IDH1p

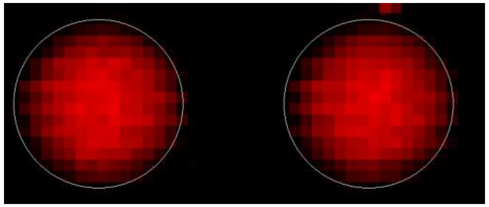

Grade II_CF 29538_2000154020

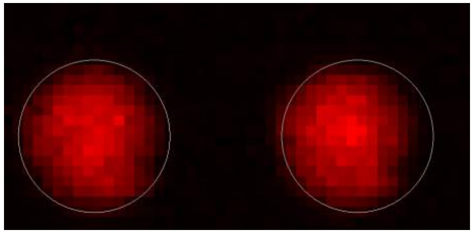

Grade II_CJ 33350_2000144452

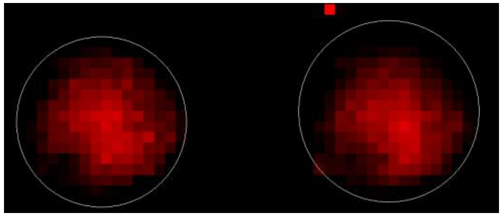

Grade II_CJ 4825_2000143981

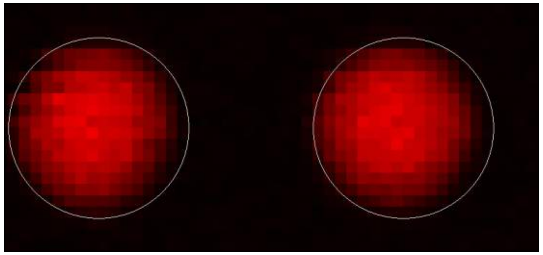

Grade II_CJ 7132_2000154028

NM_000519.3 (HBD) – Wild Type

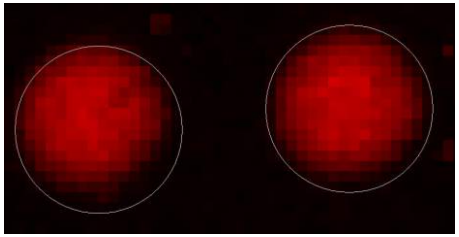

Grade II_CF 10729_2000154012

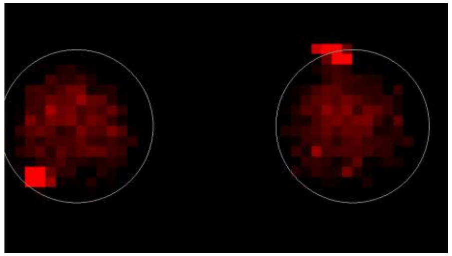

Grade II_CF 20468_2000153947

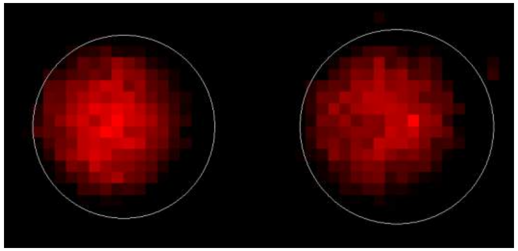

Grade II_CJ 10242_2000144449_2

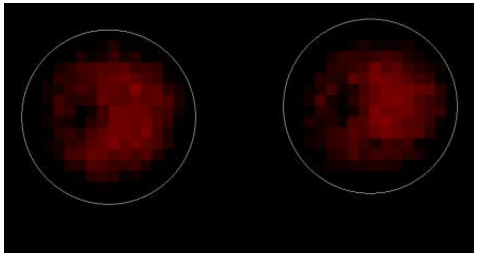

Grade II_CK 40_2000144445

NM_017863.2 (CXORF48) – IDH1p

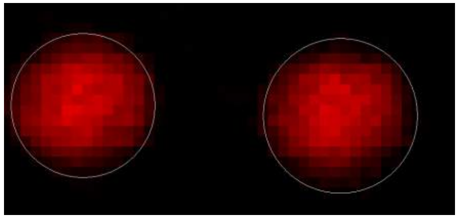

Grade II_CF 29538_2000154020

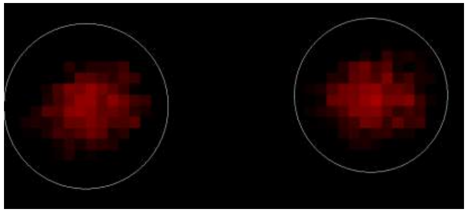

Grade II_CJ 33350_2000144452

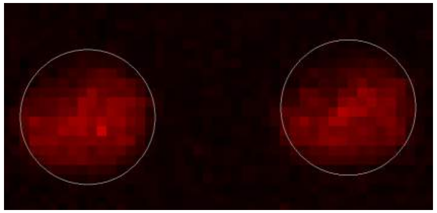

Grade II_CJ 4825_2000143981

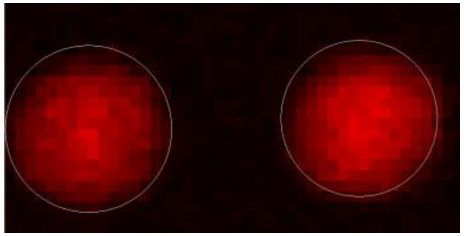

Grade II_CJ 7132_2000154028

NM_017863.2 (CXORF48) – Wild Type

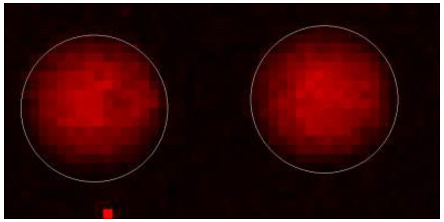
Grade II_CF 10729_2000154012

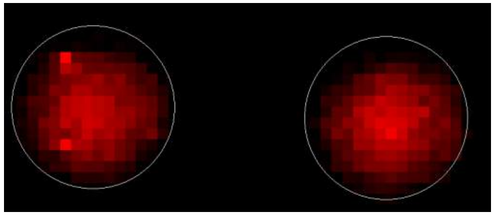
Grade II_CF 20468_2000153947

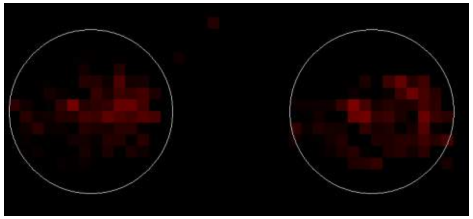
Grade II_CJ 10242_2000144449_2

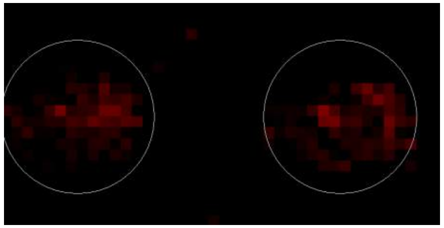
Grade II_CK 40_2000144445

BC089418.1 (IGLV3-25) – IDH1p

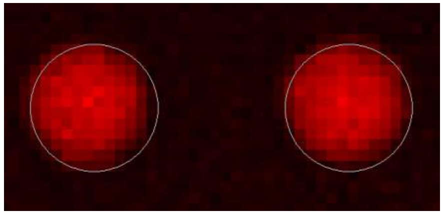

Grade II_CF 29538_2000154020

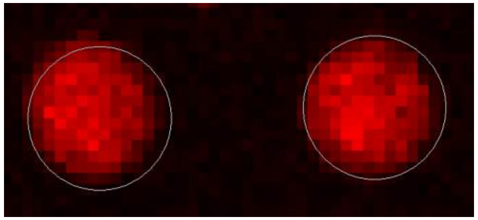

Grade II_CJ 33350_2000144452

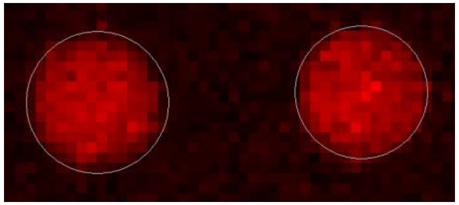

Grade II_CJ 4825_2000143981

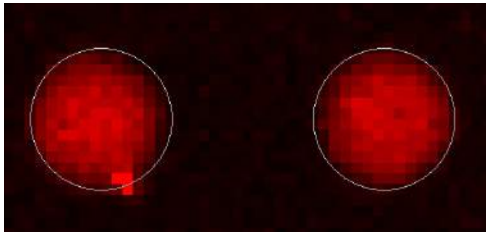

Grade II_CJ 7132_2000154028

BC089418.1 (IGLV3-25) – Wild Type

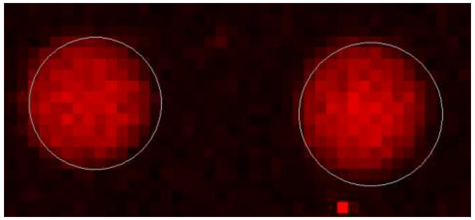

Grade II_CF 10729_2000154012

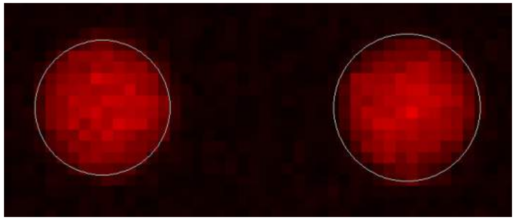

Grade II_CF 20468_2000153947

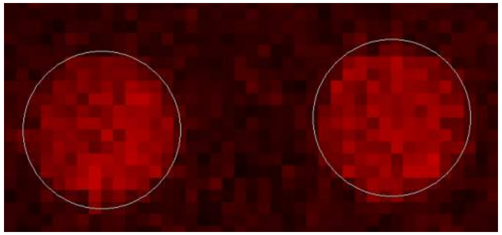

Grade II_CJ 10242_2000144449_2

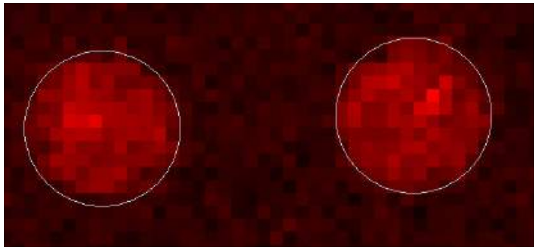

Grade II_CK 40_2000144445

NM_014440.1 (IL1F6) – IDH1p

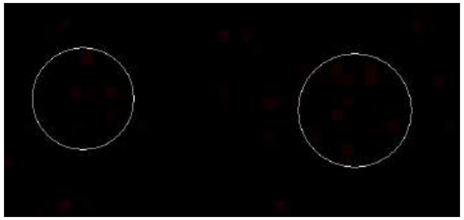

Grade II_CF 29538_2000154020

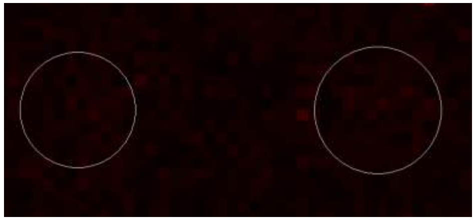

Grade II_CJ 33350_2000144452

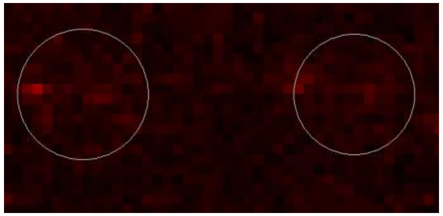

Grade II_CJ 4825_2000143981

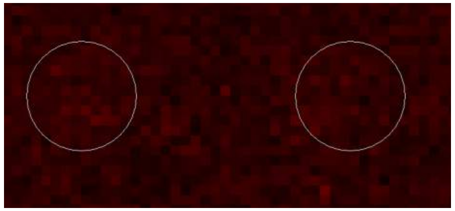

Grade II_CJ 7132_2000154028

NM_014440.1 (IL1F6) – Wild Type

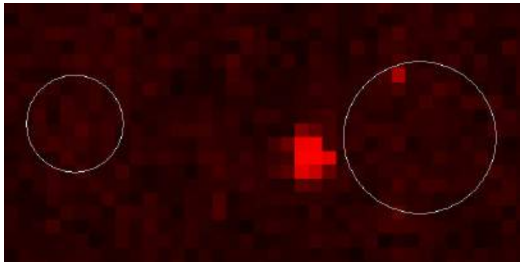

Grade II_CF 10729_2000154012

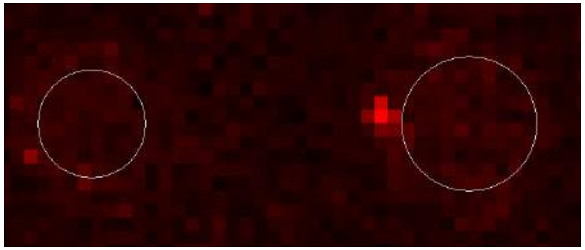

Grade II_CF 20468_2000153947

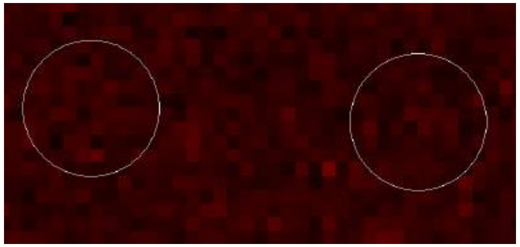

Grade II_CJ 10242_2000144449_2

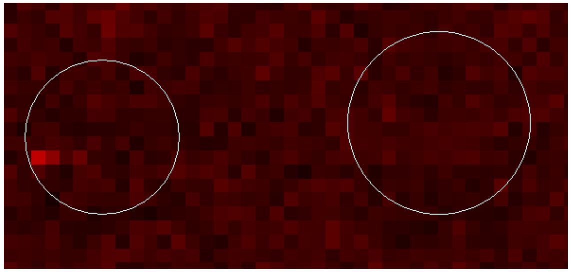

Grade II_CK 40_2000144445

NM_130897.1 (DYNLRB2) – IDH1p

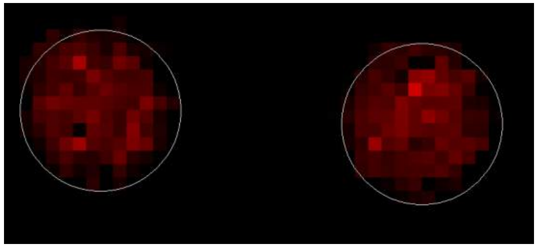

Grade II_CF 29538_2000154020

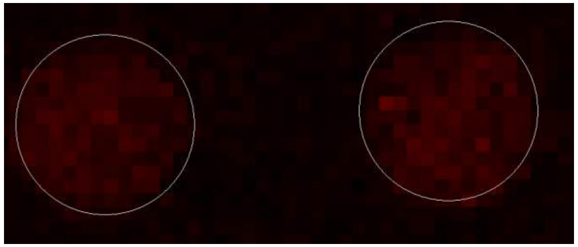

Grade II_CJ 33350_2000144452

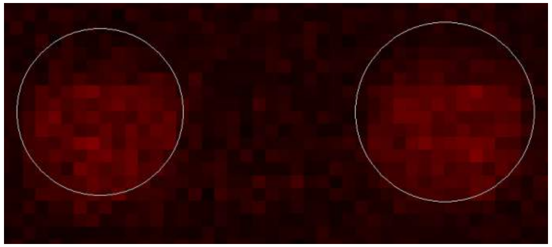

Grade II_CJ 4825_2000143981

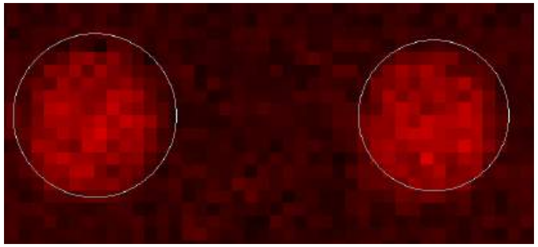

Grade II_CJ 7132_2000154028

NM_130897.1 (DYNLRB2) – Wild Type

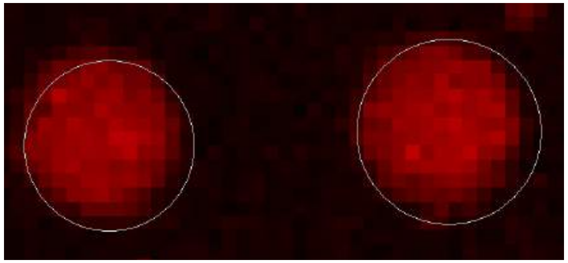

Grade II_CF 10729_2000154012

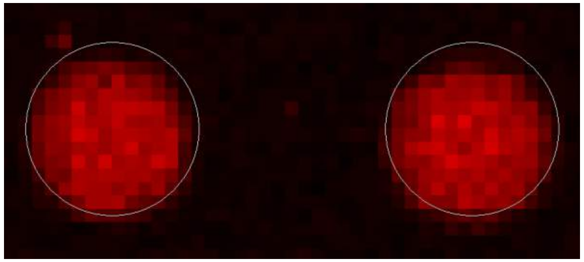

Grade II_CF 20468_2000153947

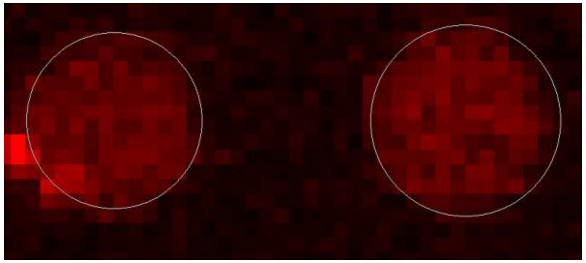

Grade II_CJ 10242_2000144449_2

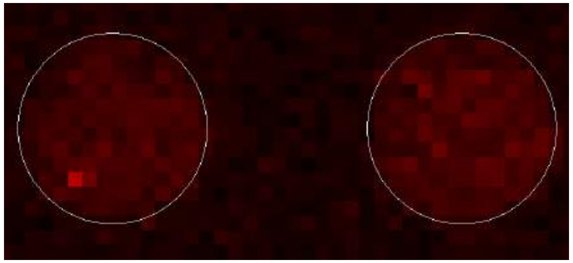

Grade II_CK 40_2000144445

BC001394.2 (SERPINB6) – IDH1p

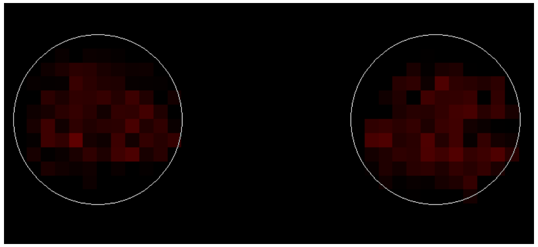

Grade II_CF 29538_2000154020

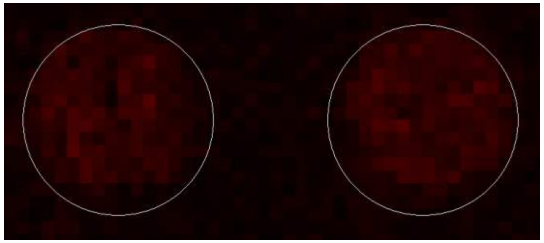

Grade II_CJ 33350_2000144452

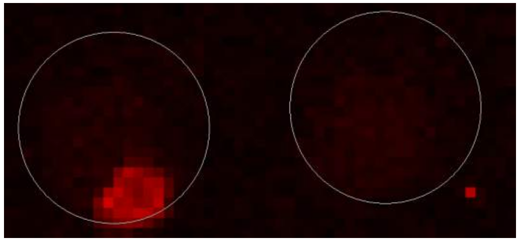

Grade II_CJ 4825_2000143981

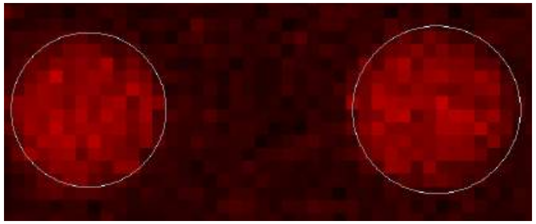

Grade II_CJ 7132_2000154028

BC001394.2 (SERPINB6) – Wild Type

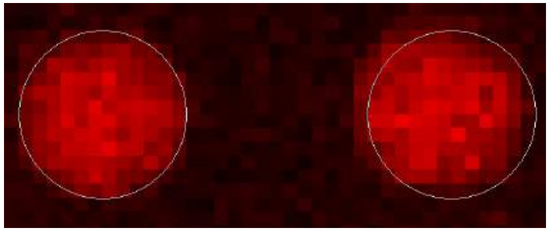

Grade II_CF 10729_2000154012

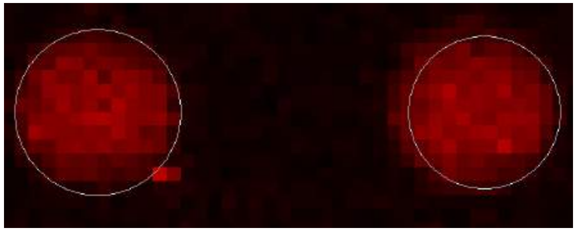

Grade II_CF 20468_2000153947

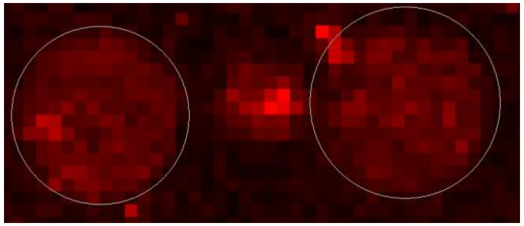

Grade II_CJ 10242_2000144449_2

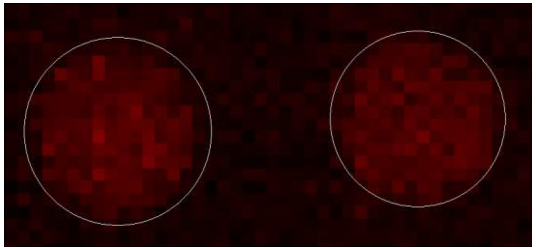

Grade II_CK 40_2000144445

BC000967.2 (NUDCD1) – IDH1p

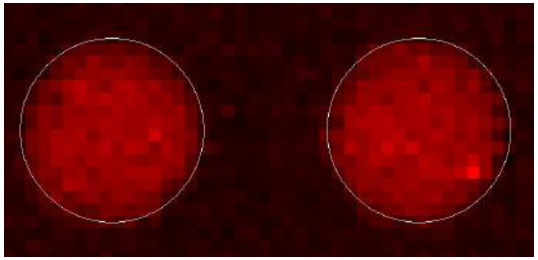

Grade II_CF 29538_2000154020

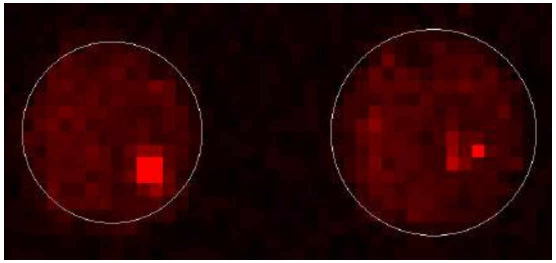

Grade II_CJ 33350_2000144452

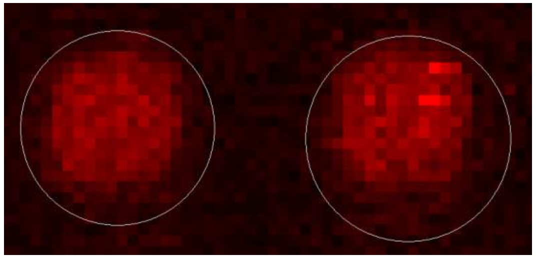

Grade II_CJ 4825_2000143981

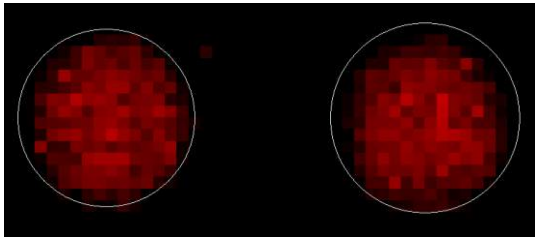

Grade II_CJ 7132_2000154028

BC000967.2 (NUDCD1) – Wild Type

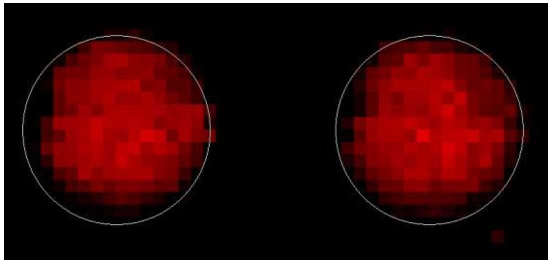

Grade II_CF 10729_2000154012

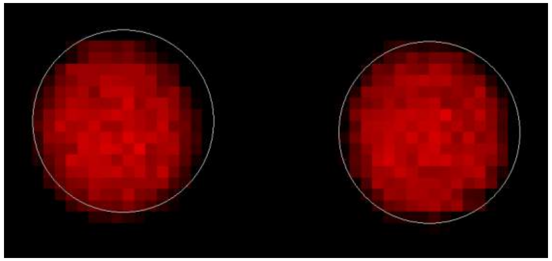

Grade II_CF 20468_2000153947

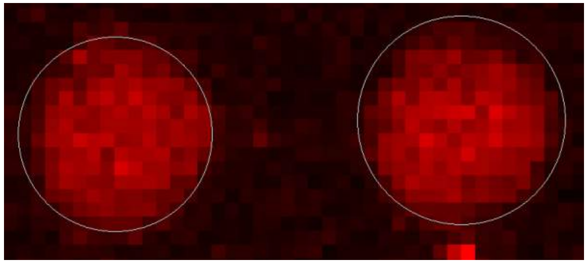

Grade II_CJ 10242_2000144449_2

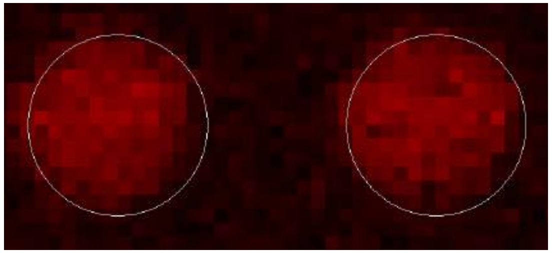

Grade II_CK 40_2000144445

BC064512.1 (UBR2) – IDH1p

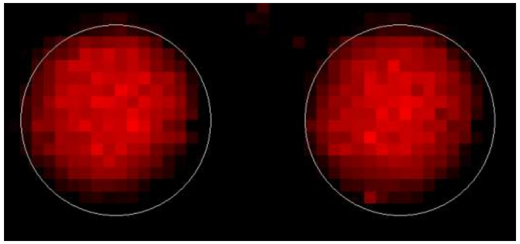

Grade II_CF 29538_2000154020

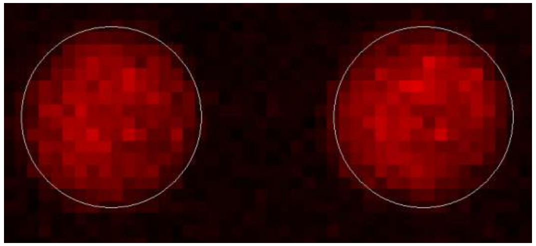

Grade II_CJ 33350_2000144452

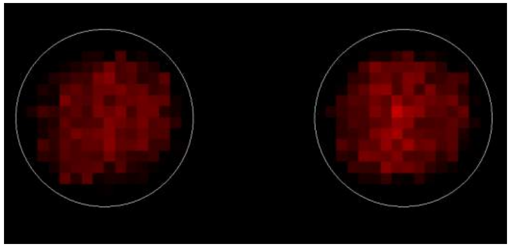

Grade II_CJ 4825_2000143981

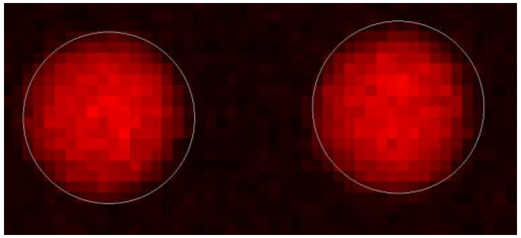

Grade II_CJ 7132_2000154028

BC064512.1 (UBR2) – Wild Type

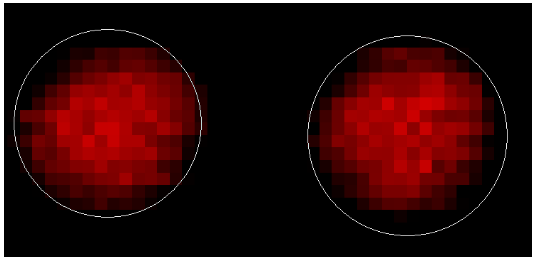

Grade II_CF 10729_2000154012

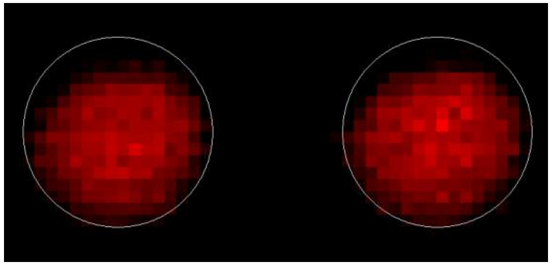

Grade II_CF 20468_2000153947

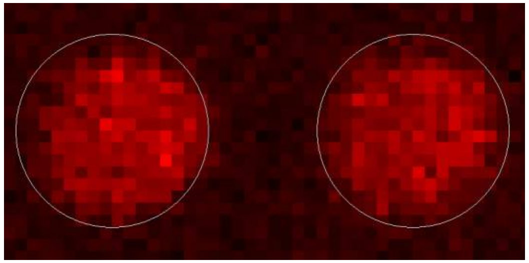

Grade II_CJ 10242_2000144449_2

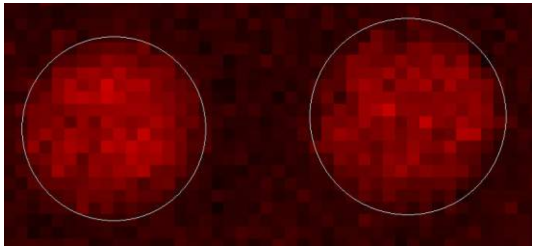

Grade II_CK 40_2000144445

NM_001106.3 (ACVR2B) – IDH1p

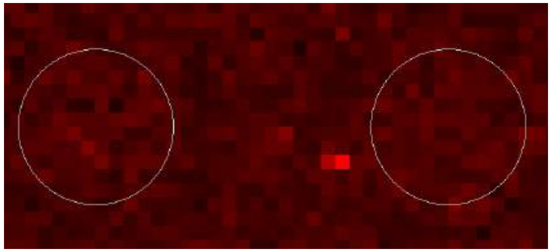

Grade II_CF 29538_2000154020

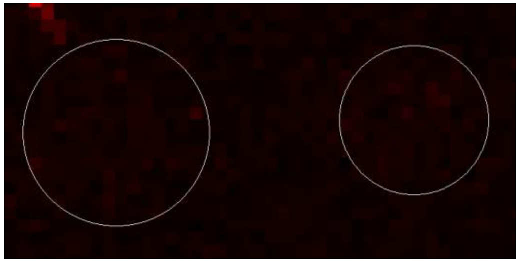

Grade II_CJ 33350_2000144452

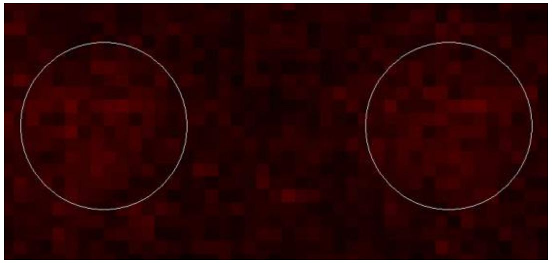

Grade II_CJ 4825_2000143981

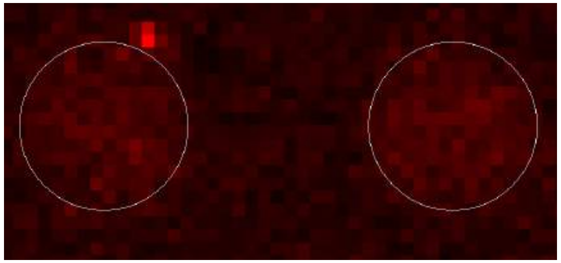

Grade II_CJ 7132_2000154028

NM_001106.3 (ACVR2B) – Wild Type

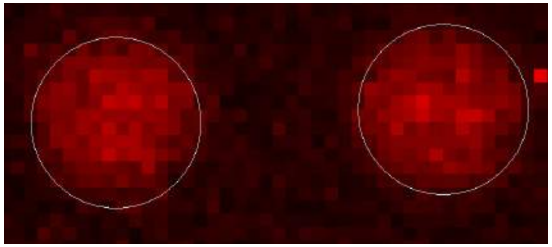

Grade II_CF 10729_2000154012

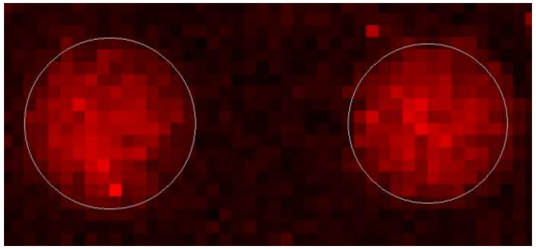

Grade II_CF 20468_2000153947

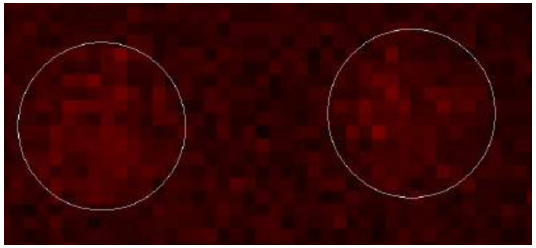

Grade II_CJ 10242_2000144449_2

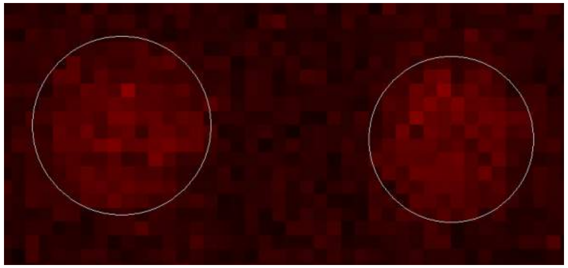

Grade II_CK 40_2000144445

BC075862.1 (CAPN1) – IDH1p

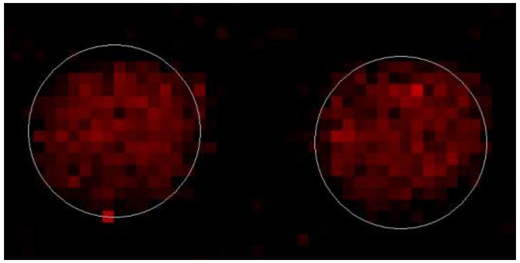

Grade II_CF 29538_2000154020

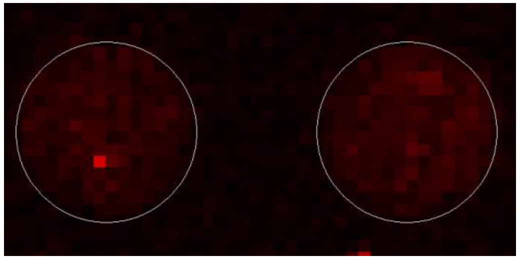

Grade II_CJ 33350_2000144452

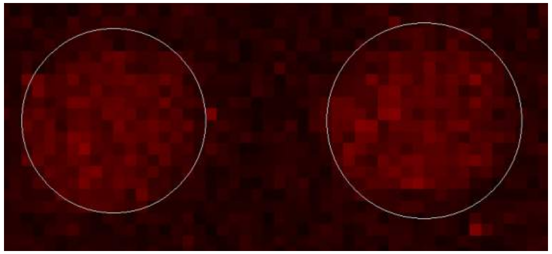

Grade II_CJ 4825_2000143981

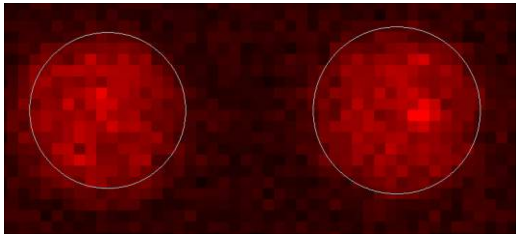

Grade II_CJ 7132_2000154028

BC075862.1 (CAPN1) – Wild Type

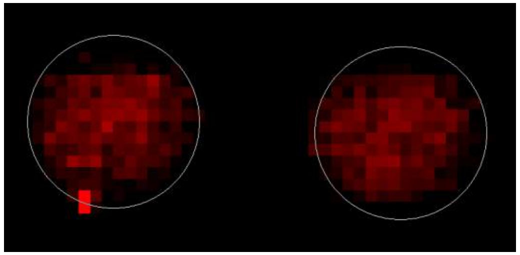

Grade II_CF 10729_2000154012

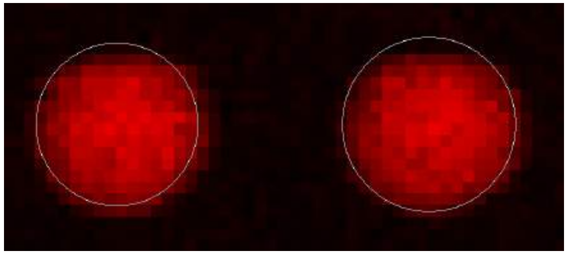

Grade II_CF 20468_2000153947

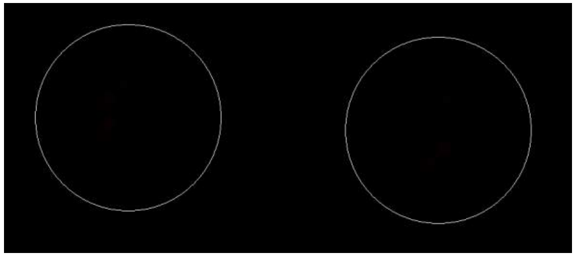

Grade II_CJ 10242_2000144449_2

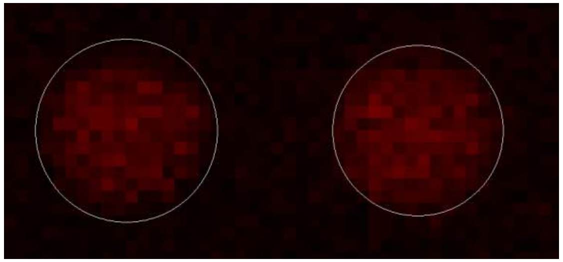

Grade II_CK 40_2000144445

BC002508.2 (ASMTL) – IDH1p

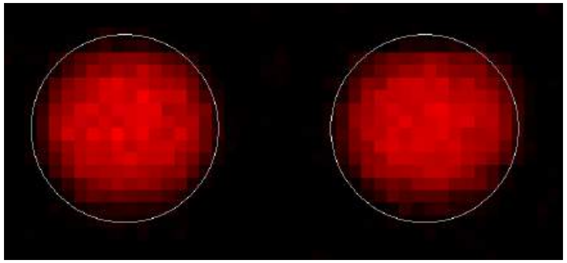

Grade II_CF 29538_2000154020

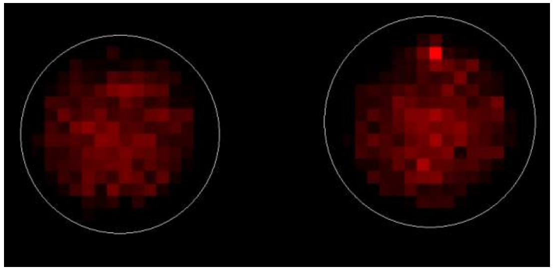

Grade II_CJ 33350_2000144452

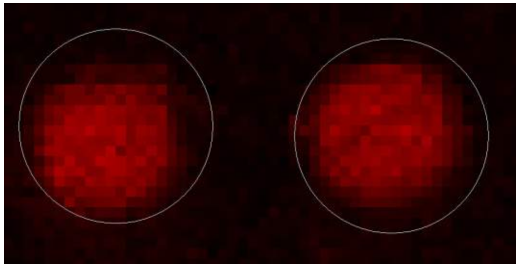

Grade II_CJ 4825_2000143981

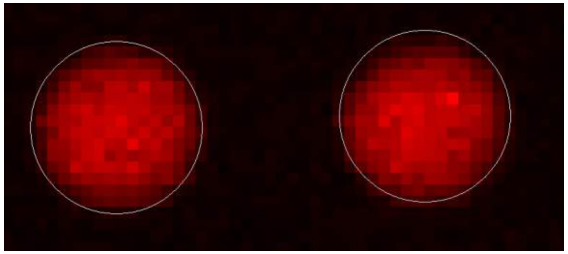

Grade II_CJ 7132_2000154028

BC002508.2 (ASMTL) – Wild Type

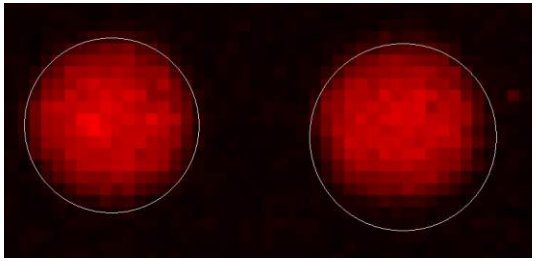

Grade II_CF 10729_2000154012

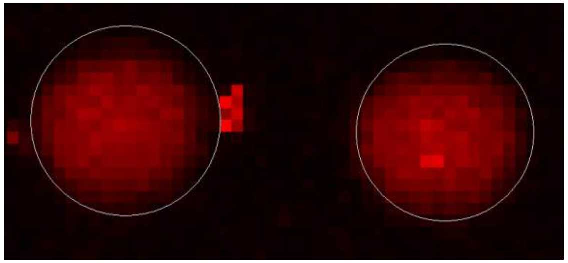

Grade II_CF 20468_2000153947

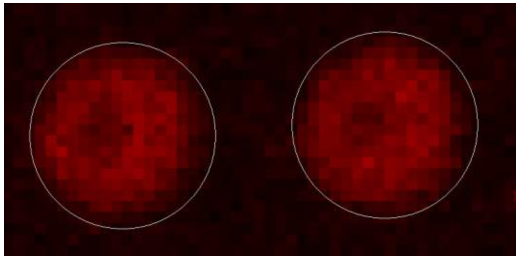

Grade II_CJ 10242_2000144449_2

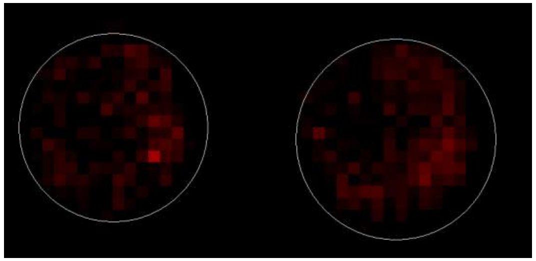

Grade II_CK 40_2000144445

NM_003405.3 (YWHAH) – IDH1p

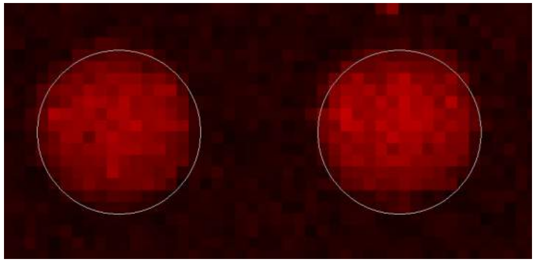

Grade II_CF 29538_2000154020

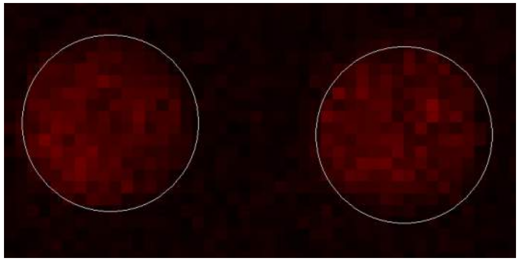

Grade II_CJ 33350_2000144452

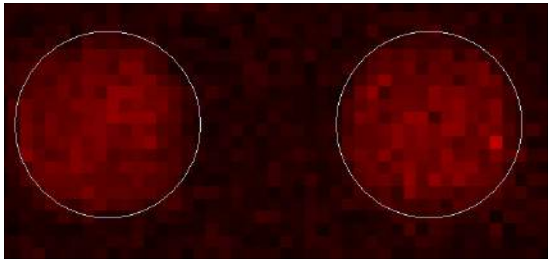

Grade II_CJ 4825_2000143981

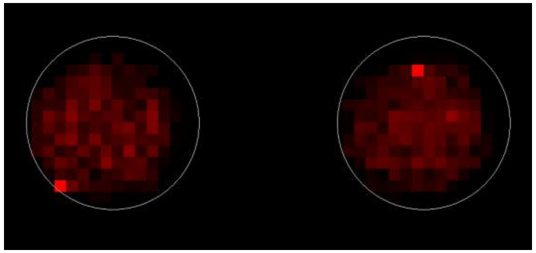

Grade II_CJ 7132_2000154028

NM_003405.3 (YWHAH) – Wild Type

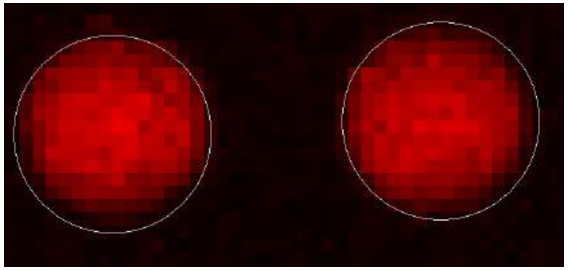

Grade II_CF 10729_2000154012

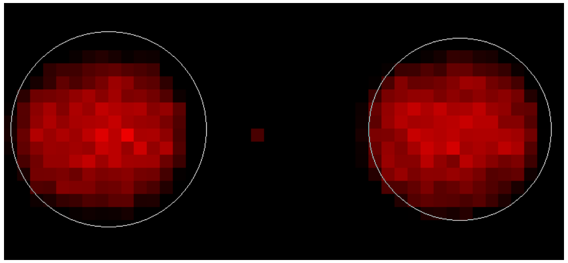

Grade II_CF 20468_2000153947

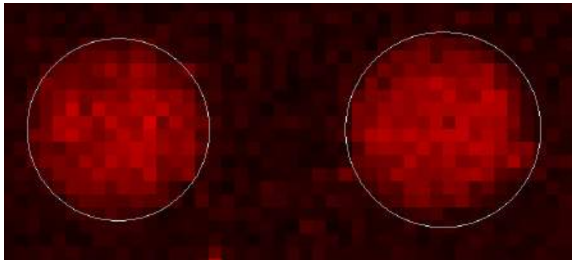

Grade II_CJ 10242_2000144449_2

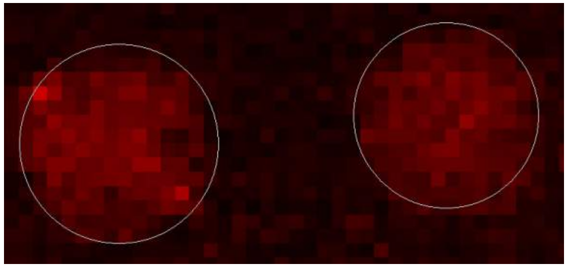

Grade II_CK 40_2000144445

NM_013330.3 (NME7) – IDH1p

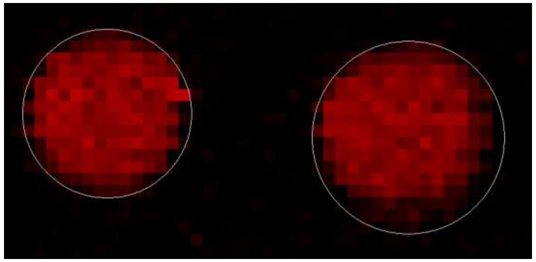

Grade II_CF 29538_2000154020

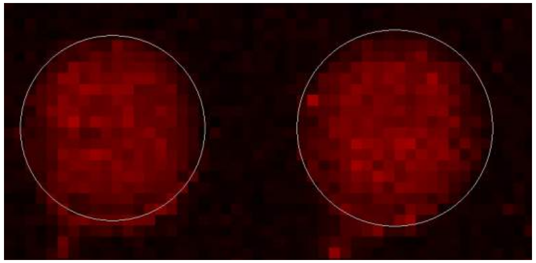

Grade II_CJ 33350_2000144452

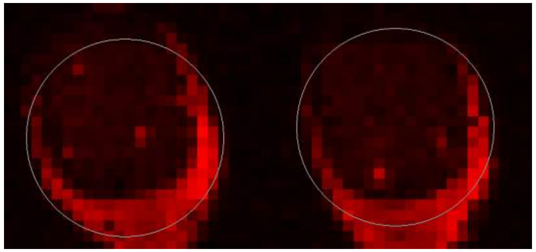

Grade II_CJ 4825_2000143981

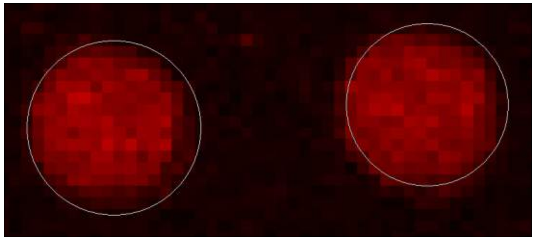

Grade II_CJ 7132_2000154028

NM_013330.3 (NME7) – Wild Type

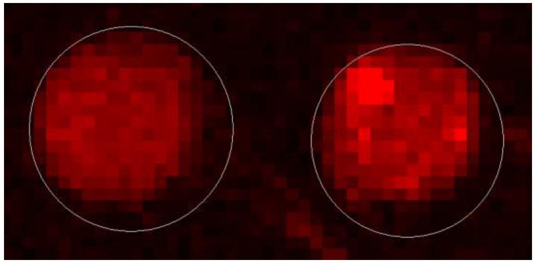

Grade II_CF 10729_2000154012

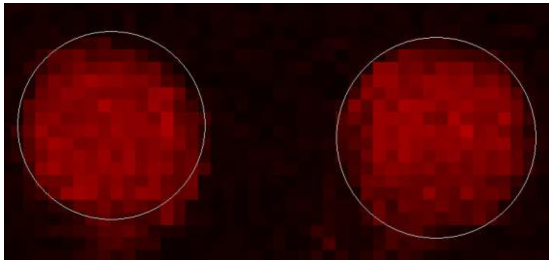

Grade II_CF 20468_2000153947

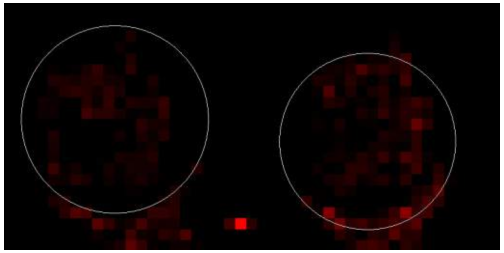

Grade II_CJ 10242_2000144449_2

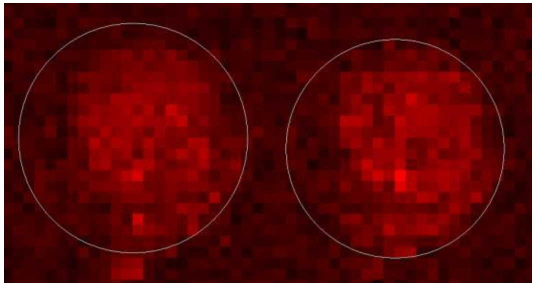

Grade II_CK 40_2000144445

Grade IV_IDH1p Vs Grade IV_Wild Type IDH1

NM_152734.3 (C6orf89) – IDH1p

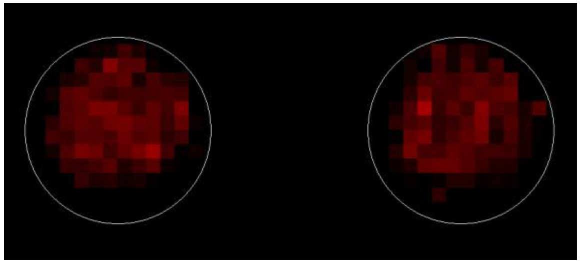

Grade IV_CF 12166_2000153934

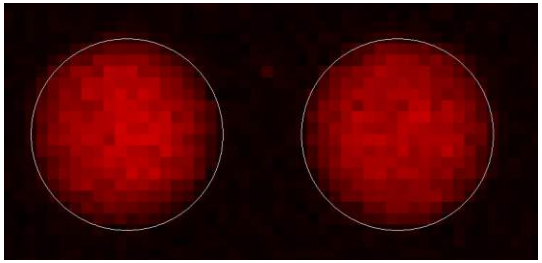

Grade IV_CF 7916_2000154021

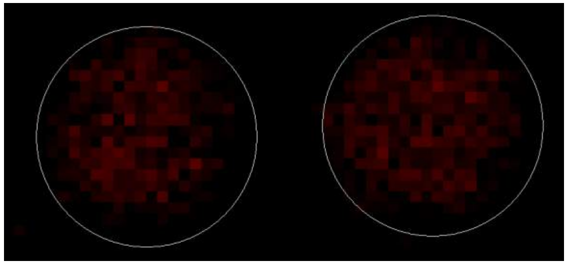

Grade IV_CJ 22776_2000153756

NM_152734.3 (C6orf89) – Wild Type

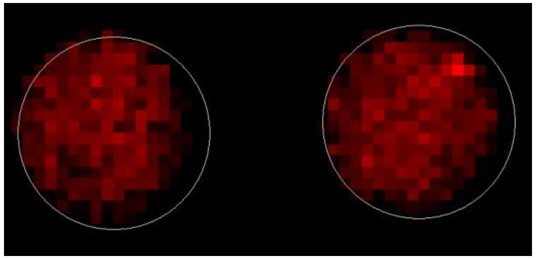

Grade IV_CH 18016_2000153791

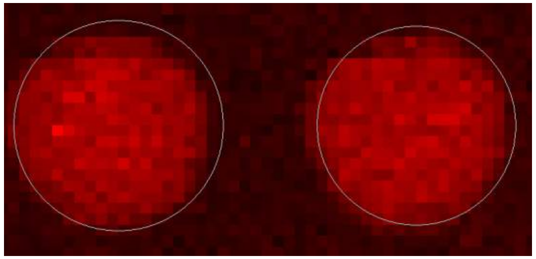

Grade IV_CJ 12441_2000153760

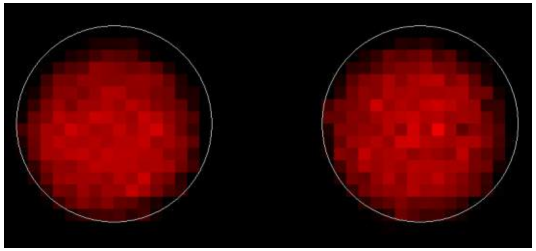

Grade IV_CJ 2041_2000154019

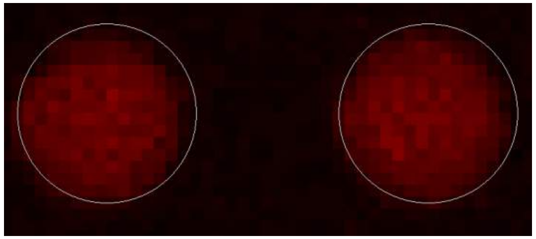

Grade IV_CH 3969_2000153950

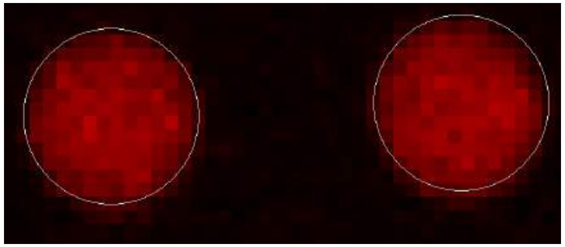

Grade IV_CJ 13655_2000153783

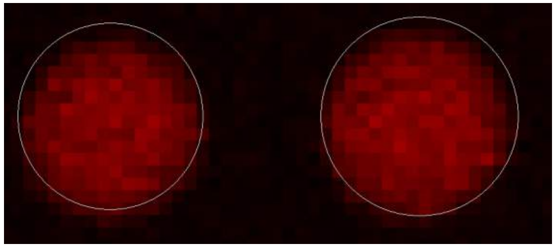

Grade IV_CJ 30014_2000153805
